# DMRforPairs: identifying Differentially Methylated Regions between unique samples using array based methylation profiles

**DOI:** 10.1186/1471-2105-15-141

**Published:** 2014-05-15

**Authors:** Martin A Rijlaarsdam, Yvonne G van der Zwan, Lambert CJ Dorssers, Leendert HJ Looijenga

**Affiliations:** 1 Department of Pathology, Laboratory for Experimental Patho-Oncology, Erasmus MC - University Medical Center Rotterdam, Be-432, P.O. Box 2040, Rotterdam 3000 CA, Netherlands

**Keywords:** DMR, Methylation, Illumina Infinium HumanMethylation450 BeadChip, Unique samples, Array

## Abstract

**Background:**

Array based methylation profiling is a cost-effective solution to study the association between genome methylation and human disease & development. Available tools to analyze the Illumina Infinium HumanMethylation450 BeadChip focus on comparing methylation levels per locus. Other tools combine multiple probes into a range, identifying differential methylated regions (DMRs). These tools all require groups of samples to compare. However, comparison of unique, individual samples is essential in situations where larger sample sizes are not possible.

**Results:**

DMRforPairs was designed to compare regional methylation status between unique samples. It identifies probe dense genomic regions and quantifies/tests their (difference in) methylation level between the samples. As a proof of concept, DMRforPairs is applied to public data from four human cell lines: two lymphoblastoid cell lines from healthy individuals and the cancer cell lines A431 and MCF7 (including 2 technical replicates each). DMRforPairs identified an increasing number of DMRs related to the sample phenotype when biological similarity of the samples decreased. DMRs identified by DMRforPairs were related to the biological origin of the cell lines.

**Conclusion:**

To our knowledge, DMRforPairs is the first tool to identify and visualize relevant and significant differentially methylated regions between unique samples.

## Background

Epigenetic (de)regulation, including DNA (CpG) methylation, is associated with development, differentiation and many human diseases [1-3] including the initiation and progression of various cancers [3-8]. While the primary DNA sequence is mostly stable during the lifetime of an individual, the epigenome is highly dynamic and responsive. Because of this, it provides valuable information about (past) (micro-)environmental conditions in the context of human disease and development [9,10].

DNA CpG methylation is routinely investigated on a genome wide scale [2,3]. The methylation profile can be assessed using micro-arrays or sequencing by applying (1) methylation-sensitive restriction enzymes or immunoprecipitation (anti-5mC) or (2) bisulfite-based treatment, which converts unmethylated cytosines into uracils [11]. The Illumina Infinium HumanMethylation450 BeadChip (450 K) is a bisulfite-based, cost-effective, two-color array querying over 480,000 independent genomic positions (99% Refseq genes, 96% CpG islands) [12-14]. Various tools are available to pre-process and analyze the 450 K data, but differential methylation is primarily detected per locus or by comparing differential patterns across regions using groups of samples [15]. The latter is implemented in IMA and bumphunter. Indeed, IMA offers region based analysis [16], but it does not work when using unique samples. Bumphunter identifies regional changes in the regression coefficient between methylation status and phenotype. Therefore, bumphunter (like IMA) requires groups of samples of sufficient size to estimate this coefficient for each probe [17]. However, when analyzing small numbers of samples with unique characteristics (e.g. normal and affected tissue of a clinically unique patient or a manipulated cell model), large series of samples are not available and current methods cannot be applied. Although larger series of samples are preferred (biological replicates or more patients), comparison of unique samples is desired in such a situation. DMRforPairs was designed to address this problem by comparing regional methylation status between unique samples.

### Implementation

**Figure 1:**
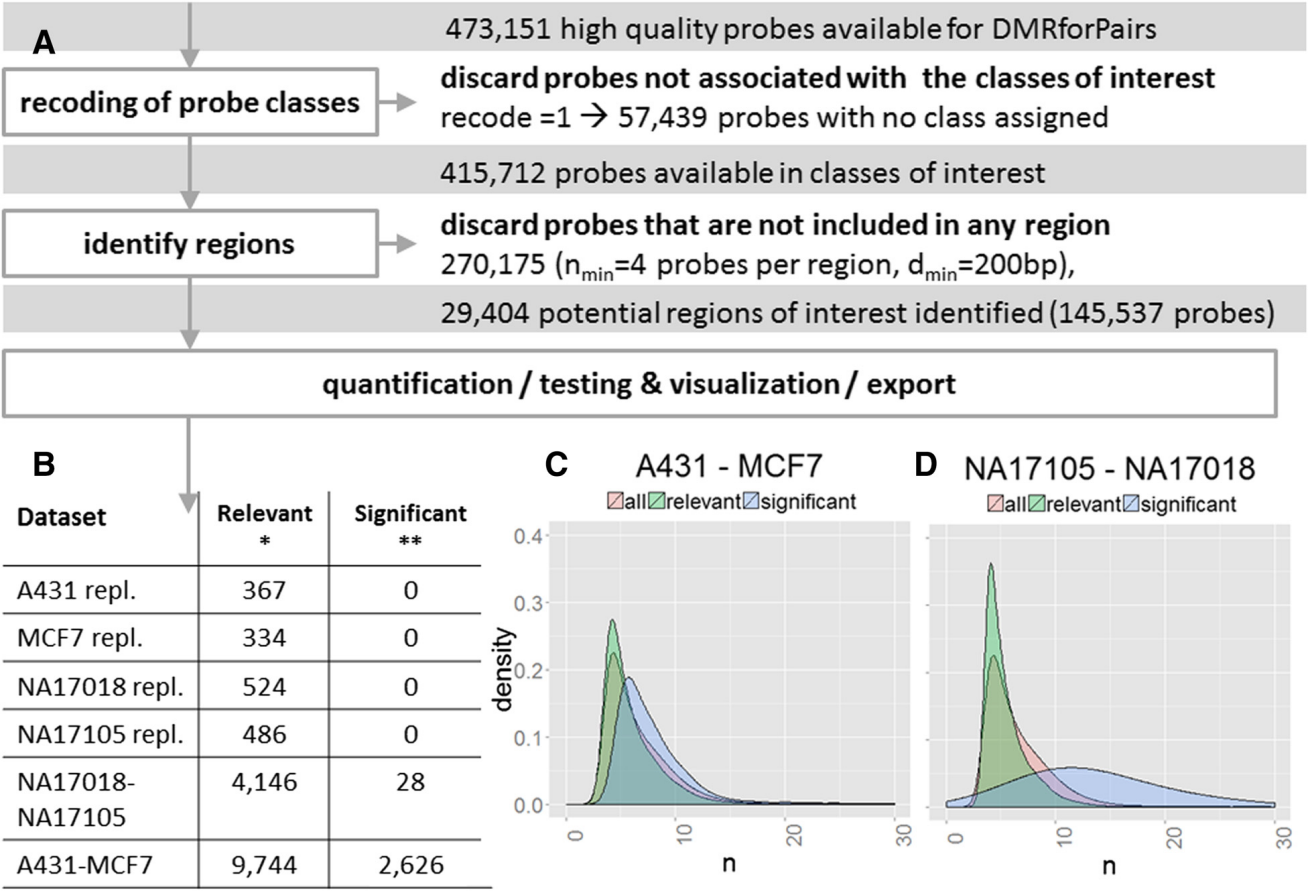
**Flowchart and overview of DMRforPairs results of the Illumina data. (A)** The subsequent steps of recoding probe classes, identifying regions, quantifying and testing methylation differences and exporting the results are described in detail in the main text. Briefly, 473,151 probes remained after quality control. Subsequently, probes not associated to any of the 11 classes or not included in any of the regions are discarded. 145,537 probes (35%) were included in 29,404 potential regions of interest. Finally, these were assessed for methylation differences. **(B)** Number of regions identified in the various pairwise analyses. * = relevant indicates regions with |ΔM| > 1.4, ** = significant indicates relevant + p_adjusted_ ≤ 0.05. “repl.” indicates technical replicates. **(C,D)** The density plots illustrate the distribution of all/the relevant/the significant regions with regard to the number of probes in each region. Only the comparisons of the two cancer cell lines **(C)** and the pair of lymphoblastoid cell lines **(D)** are depicted as the technical replicates yielded no significant DMRs.

The algorithm consists of a number of phases (Figure 1A) with fully customizable parameters which will be discussed below:

1) Recoding of the probe classes

2) Identification of regions with sufficient probe density

3) Quantification and testing of (difference in) methylation status.

### Data import and pre-processing

As input DMRforPairs requires the methylation percentage of each CpG site in each sample. It was originally designed for the 450 K array, but is applicable to any platform that generates a methylation percentage per CpG site and has sufficient coverage. For example, Additional file 1 illustrates the algorithm’s applicability to data generated using Nimblegen microarrays and the McrBC protocol (CHARM). DMRforPairs does not provide functions to import, filter (cross-hybrization, SNPs in probe sequencing) or pre-process 450 K data because of the existence of a number of excellent, well maintained pre-processing R-pipelines [11,15,16,18-22]. In the package documentation examples are provided on how to extract 450 K data for DMRforPairs using the lumi (http://www.bioconductor.org/packages/release/bioc/html/lumi.html), IMA (http://ima.r-forge.r-project.org/) and minfi (http://www.bioconductor.org/packages/2.12/bioc/html/minfi.html) pipelines. The output of these pipelines serves as input for DMRforPairs.

### Recoding of the probe classes

Illumina assigns the majority of probes to eleven specific classes according to their association to one or more functional regions (relation to gene: Body, 5'UTR, 3'UTR, 1^st^ exon, TSS1500, TSS200; relation to CpG island: Island, Northern/Southern Shelf & Shore [12]). Highly detailed classification may result in too low probe density per class as DMRforPairs investigates probes in close proximity to each other within each class individually. DMRforPairs therefore allows custom grouping and/or selection of classes. Three commonly used schemes are hard-coded in the software: (0) retain all 11 classes, (1) group on relation to gene/transcription start site/CpG island or (2) put all probes in one class. The last option ignores the assigned classes as it might be desirable to just let DMRforPairs identify DMRs without providing information about probes that belong to the same functional class. This option can also be used in case this functional classification is unknown.

### Identification of regions with sufficient probe density

A region of interest meets the following criteria:

1) Neighboring probes lay within d_min_ bp of each other (default = 200),

2) The number of probes per region ≥ n_min_ (default = 4), and

3) All probes are annotated to the same functional class (please see above).

**Figure 2:**
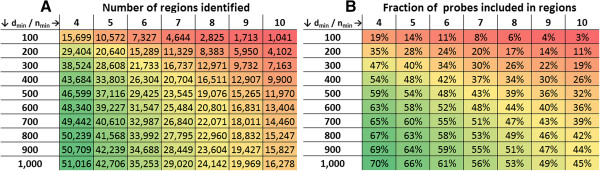
**Tuning of the of d**_**min **_**and n**_**min **_**parameters. (A)** Number of regions identified and **(B)** fraction of all probes included in these regions using different settings of d_min_ and n_min_. d_min_ denotes the maximal distance in bp allowed between two adjacent probes to be accepted in the same region. n_min_ denotes the minimal number of probes in a region (per sample). All runs of the algorithm were done using the 415,712 probes annotated to at least one Illumina class grouped according to gene/transcription start site/CpG island (recode parameter = 1). These benchmark statistics can be generated using the *tune_parameters* function in the algorithm (optional parallelization).

Default settings of d_min_ are based on decreasing correlation between methylation status of adjacent loci when evaluated at inter-locus distances between 0 and 1 kb (200 bp is reported to correlate well) [11,23]. The default value for n_min_ is based on the theoretical minimal number of 2×4 observations required for statistical testing using Mann–Whitney U test. Probes annotated to more than one class are included in multiple regions and fully identical regions from different classes are merged into one region with a combined class. Figure 2 illustrates the number of regions identified for various settings of d_min_ and n_min_ and the fraction of all probes included in the regions. A function is available in DMRforPairs to generate these benchmarking results for specific data sets and tune the settings of n_min_ and d_min_.

### Quantification and testing of methylation status

As recommended, the methylation percentage β and the M-values (logit_2_(β)) were used for visualization and statistical computations respectively [24]. Descriptive statistics are computed by DMRforPairs for all regions and samples (optional parallelization). These consist of median methylation levels (M and β values) and pairwise differences in median methylation level between all samples. If the median difference in M value between any pair of samples is sufficiently large in a specific region (> |ΔM|), the difference is formally tested using the Mann Whitney U or Kruskal-Wallis test. Pairwise testing is performed if indicated (n > 2 & p_Kruskal-Wallis_ ≤ 0.05). An α of 0.05 after adjustment for multiple testing (Bejamini & Hochberg (FDR) [25]) is used to select significant regions (default settings). α and the method to correct for multiple testing can be specified by the user.

Several issues need to be kept in mind when choosing the algorithm’s parameters and interpreting (test) results. In general, setting the algorithm’s parameter more stringently (|ΔM|↑,n_min_↑,d_min_↓)) reduces the amount of regions to be tested, but also discards potential DMRs that are less optimally covered by the probes on the array. Concerning the |ΔM| threshold it is important to be aware that the default setting (1.4) lies at the upper bound of the range (0.4-1.4) recommended by Du *et al*. A less stringent setting might result in a higher detection rate but reduces the true positive rate and increases the amount of multiple testing performed by DMRforPairs [24]. Also, correlation of methylation levels of CpG sites located closely together on the genome should be kept in mind. The potential presence of correlation warrants careful evaluation of statistical test results related to the independency assumption even though methylation levels at specific sites are technically (different probes) and biologically (different genomic positions) independent. Finally, comparisons with a higher number of probes per region have a higher power and are more likely to survive multiple testing. Therefore, the list of significant DMRs is theoretically biased towards regions with more probes (i.e. larger sample size). This bias was limited in a comparison of samples which are derived from a strongly biologically different origin (Figure 1B,C). When comparing the more similar samples there was some overrepresentation of regions with a high number of probes (28 DMRs, Figure 1B,D).

### Visualization, export and exploration

**Figure 3:**
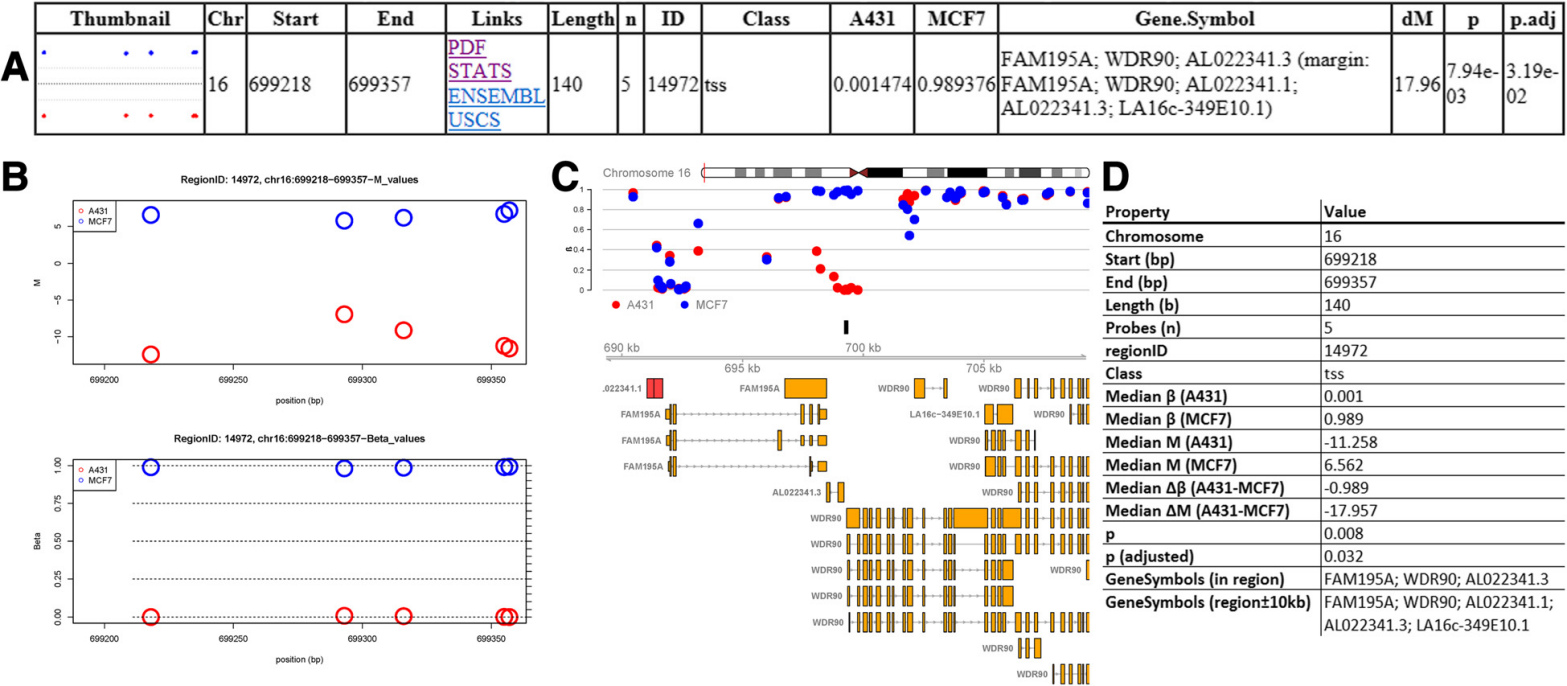
**DMRforPairs output. (A)** One row of the HTML table describing one DMR. Thumbnail, genomic annotation and descriptive statistics regarding (the difference between) the samples are presented as well as links to figures/tables illustrating the methylation patterns in the samples in detail. Direct links to the genomic region in two genome browsers are also provided (Ensembl & UCSC). Region IDs are generated on the fly by the *regionfinder* function and are specific to a dataset and to a set of DMRforPairs parameters. They are therefore not interchangeable between datasets/experiments and serve mainly as identifiers during exploration of the dataset. **(B)** Methylation level per probe (M and β values) plotted against its genomic position. These plots are generated for all relevant and significant regions. **(C)** Annotated visualization of DMRs (β values) ±10 kb. Black box indicates the DMR. Transcripts overlapping/near the region are retrieved from Ensembl. These plots are optionally generated for all relevant and significant regions. **(D)** Additional statistics (STATS link in table) as provided for each region.

HTML tables listing all, only relevant (median difference ≥ ΔM) and only significant regions are generated with links to genome browsers (Figure 3A, application of the R2HTML package [26]). Links are also provided to images depicting the observed methylation profiles and a text file with additional descriptive statistics (Figure 3). Pairwise plots are generated in case of more than two biological samples. For relevant and significant DMRs an extended output can be generated including thumbnails in the HTML tables and visualizations that also depict transcripts annotated (close) to the region (Figure 3C, application of the Gviz & GenomicRanges packages [27,28]). In addition, DMRforPairs includes a number of functions to further inquire the data. Methylation status of genes of interest, regions identified by DMRforPairs and custom genomic intervals can be visualized, annotated and quantified/tested.

## Results and discussion

### Dataset

As a proof of concept, DMRforPairs is applied to a public dataset including two commercially available EBV transfected lymphoblastoid cell lines from healthy individuals (NA17105 (African American male) and NA17018 (Chinese female), Coriell Institute for Medical Research (NJ, USA) (http://ccr.coriell.org/)). The dataset also includes the breast cancer cell line MCF7 [29] and HPV negative squamous-cell vulva carcinoma cell line A431 [30,31]. Data is available at Illumina’s website (http://support.illumina.com/downloads/genomestudio_software_20111.ilmn ) [12,13] and was processed in GenomeStudio V2011.1 and R 3.0.1 (Windows 7 × 64) and 2.15.2 (Redhat Linux × 64) using Illumina’s annotation manifest (v. 1.1, http://support.illumina.com/downloads/humanmethylation450_15017482_v12.ilmn). Import and pre-processing was carried out using the LUMI package (http://www.bioconductor.org/packages/release/bioc/html/lumi.html) [19] following the optimized “lumi: QN + BMIQ” pipeline [11]. This includes exclusion of poorly performing probes (p < 0.01, n = 713), color adjustment, quantile normalization and correction for probe type bias (Infinium I vs II) using the BMIQ algorithm [20]. Differentially methylated regions were identified by applying the DMRforPairs algorithm using the default settings (Figure 1, d_min_ = 200, n_min_ = 4, ΔM = 1.4, recode = 1, α = 0.05, correction for multiple testing = Benjamini Hochberg (FDR)). The networks/enrichment analyses were performed in IPA (Ingenuity® Systems, http://www.ingenuity.com, Core analysis; default settings).

## Results

In the Illumina manifest, 12% of the probes were not assigned to any of the 11 categories (discarded in this analysis with recode parameter set to 1). 35% of the remaining probes was included in one or more regions, leading to 29,404 potential regions of interest. Samples were compared pairwise in descending order of biological similarity: technical duplicates, lymphoblastoid cell lines and cancer cell lines (average of duplicates) (Figure 1, Additional file 2).

As expected, no DMRs were identified when comparing the pairs of technical replicates (Figure 1B). In the two lymphoblastoid cell lines, 28 DMRs were identified (Figure 1B,D). Fitting with the Chinese and African American origin of the cell lines, top DMRs were associated with regions encoding human leucocyte antigens involved in immune response and known to be differently methylated between populations [32] (e.g. HLA-DRB1 (rank 2), HCG27 (rank 4), HLA-K / HCG4B (rank 7)). Enrichment/network analysis in IPA showed significant overrepresentation of genes associated with immunological diseases. This concerned various auto-immune diseases and lymphoma (9 genes, p = 0.000271-0.0293 depending on the subcategory; ACTA1, CHST8, GABR1, HCG27, HLA-DRB1, IGF2-AS, POU5F1, ZNF165, VTRNA2-1).

Between A431 and MCF7 2,626 DMRs were identified (Figure 1B,C). On top of the list was FAM195A a gene with known low expression [33] and complete methylation in MCF7. In A431, the region showed complete demethylation, but no public expression data was available for this cell line. The rest of the top-5 consisted of homeobox genes which are frequently methylated in breast cancer and active in squamous cell carcinoma [34,35]. Cancer was by far the strongest overrepresented disease category in the enrichment/network analysis (989 genes, p = 1.31E-19 - 2.71E-4). Enriched subcategories included breast cancer (n = 234, p = 2.06E-10), head and neck (squamous cell) carcinoma (n = 131, p = 1.30E-7) and genital tumor (n = 192, p = 1.94E-7).

## Conclusions

DMRforPairs defines genomic regions using local probe density and optionally functional homogeneity. It quantifies, tests, annotates and visualizes (differential) methylation patterns between unique samples including pairwise comparison of samples if n > 2. Here, it is shown that in two lymphoblastoid cell lines from healthy individuals and cancer cell lines A431 and MCF7 (including 2 technical replicates each), DMRforPairs was able to identify an increasing number of DMRs related to the sample phenotype when biological similarity of the samples decreased. DMRs identified by DMRforPairs were related to the biological origin of the cell lines. In addition, DMRforPairs has been applied successfully in the analysis of integrated genome-wide epigenetic and expression profiles of germ cell cancer cell lines [36].

### Availability & requirements

**Project name:** DMRforPairs

**Project home page:**http://bioconductor.org/packages/release/bioc/html/DMRforPairs.htmlhttp://www.martinrijlaarsdam.nl/DMRforPairs/

**Operating system(s):** Platform independent

**Programming language:** R

**Other requirements:** R 2.15.2 or higher. Bioconductor packages: Gviz (> = 1.2.1) [27], R2HTML (> = 2.2.1) [26], GenomicRanges (> = 1.10.7) [28] and parallel. The lumi [19] package is suitable to import and pre-process 450 K data for use with DMRforPairs.

**License:** GPLv3

**Restrictions to use by non-academics:** none

## Abbreviations

DMR: Differentially methylated region. In this context a DMR is defined as a region with sufficiently large median difference in methylation between two or more samples which proved to be significant after correction for multiple testing.

## Competing interests

The authors report no conflicts of interest or conflicting financial issues. For a funding statement, please see below.

## Authors’ contributions

YZ and MR designed the algorithm. MR was responsible for the implementation in R. LL and LD supervised the study and contributed to its design. All authors read and approved the final manuscript.

## Supplementary Material

Additional file 1 R script illustrating the use of DMRforPairs with CHARM instead of 450 K data. Click here for file

Additional file 2 **DMRforPairs output for the comparison of A431-MCF7 and NA17018-NA17105.** Please start from the HTML files in each folder. Available via the BMC Bioinformatics website. Click here for file
