# Supplementary material for: DMRforPairs: identifying Differentially Methylated Regions between unique samples using array based methylation profiles
Source: BMC Bioinformatics. 2014 May 15;15:141. doi: 10.1186/1471-2105-15-141 (PMC4046028; doi:10.1186/1471-2105-15-141)
Supplement: Additional file 2 — DMRforPairs output for the comparison of A431-MCF7 and NA17018-NA17105. Please start from the HTML files in each folder. Available via the BMC Bioinformatics website. [file 1471-2105-15-141-S2.zip › 1394847754114233_MOESM2_ESM/A431_MCF7/all.html]

 
 
 DMRforPairs output generated on Tue Nov 19 17:07:20 2013. Identified regions were set to contain at least 4 probes with a maximum distance of 200 bp between individual probes (n=29404). Regions in which median methylation levels (M-values) between the samples differed at least |1.4| (n=9744) (=relevant) were tested for statistical significance (significant: p 2). p.adj denotes the multiple testing corrected p-value (method:fdr). Gene symbols of overlapping transcripts are listed for the exact region and within a margin of 10000 bp of the region. 

	  
	  
		 Chr   
		 Start   
		 End   
		 Links   
		 Length   
		 n   
		 ID   
		 Class   
		 A431   
		 MCF7   
		 Gene.Symbol   
		 dM   
		 p   
		 p.adj  
	  
  
 20
 
  57425979
 
  57428032
 
  PDF   STATS   ENSEMBL   UCSC 
 
 2054
 
 66
 
 25471
 
 gene;tss;island
 
 0.490360
 
 0.9503845
 
 GNAS; GNAS-AS1 (margin: GNAS; GNAS-AS1; RP1-309F20.3)
 
  4.0492925
 
 3.02e-20
 
 2.94e-16
  
 
  
 20
 
  57462798
 
  57464129
 
  PDF   STATS   ENSEMBL   UCSC 
 
 1332
 
 37
 
 25473
 
 gene;tss;island
 
 0.577175
 
 0.9789009
 
 GNAS; RP1-309F20.3 (margin: GNAS; RP1-309F20.3; RP1-309F20.4)
 
  5.2600685
 
 1.34e-17
 
 6.55e-14
  
 
  
 6
 
   3848634
 
   3849577
 
  PDF   STATS   ENSEMBL   UCSC 
 
  944
 
 26
 
 34602
 
 tss
 
 0.007421
 
 0.4980592
 
 RP11-420L9.4; FAM50B (margin: RP11-420L9.4; FAM50B)
 
  7.5471681
 
 4.03e-15
 
 1.31e-11
  
 
  
 12
 
 115134148
 
 115136308
 
  PDF   STATS   ENSEMBL   UCSC 
 
 2161
 
 54
 
 10850
 
 island
 
 0.614152
 
 0.9806930
 
  (margin: )
 
  4.0939924
 
 1.12e-13
 
 2.74e-10
  
 
  
 11
 
   2019436
 
   2020560
 
  PDF   STATS   ENSEMBL   UCSC 
 
 1125
 
 36
 
 7055
 
 tss;island
 
 0.601164
 
 0.0318317
 
 H19 (margin: AC051649.6; MRPL23-AS1; H19)
 
  5.5744045
 
 4.70e-13
 
 9.15e-10
  
 
  
 12
 
  21809973
 
  21811034
 
  PDF   STATS   ENSEMBL   UCSC 
 
 1062
 
 21
 
 10280
 
 island
 
 0.014872
 
 0.9658977
 
 LDHB (margin: RP11-59N23.3; LDHB)
 
 10.2595965
 
 3.72e-12
 
 5.17e-09
  
 
  
 7
 
  94285270
 
  94286955
 
  PDF   STATS   ENSEMBL   UCSC 
 
 1686
 
 61
 
 37093
 
 gene;tss;island
 
 0.816427
 
 0.4413271
 
 PEG10; SGCE (margin: PEG10; SGCE)
 
  2.2803055
 
 3.43e-12
 
 5.17e-09
  
 
  
 6
 
  28602543
 
  28603437
 
  PDF   STATS   ENSEMBL   UCSC 
 
  895
 
 35
 
 35593
 
 island
 
 0.554668
 
 0.9772244
 
 RP11-373N24.2 (margin: RP11-373N24.2)
 
  4.1149169
 
 5.28e-12
 
 6.43e-09
  
 
  
 7
 
  95025611
 
  95026248
 
  PDF   STATS   ENSEMBL   UCSC 
 
  638
 
 20
 
 38678
 
 island
 
 0.162573
 
 0.9712755
 
 PON1; PON3 (margin: PON1; PON3; PON2)
 
  7.2867998
 
 1.45e-11
 
 1.57e-08
  
 
  
 6
 
   3848634
 
   3849818
 
  PDF   STATS   ENSEMBL   UCSC 
 
 1185
 
 31
 
 35389
 
 island
 
 0.009897
 
 0.4968564
 
 RP11-420L9.4; FAM50B (margin: RP11-420L9.4; FAM50B)
 
  6.7384453
 
 2.03e-11
 
 1.98e-08
  
 
  
 7
 
  94284258
 
  94285004
 
  PDF   STATS   ENSEMBL   UCSC 
 
  747
 
 27
 
 37092
 
 gene;tss;island
 
 0.810916
 
 0.0827249
 
 PEG10; SGCE (margin: PEG10; SGCE)
 
  5.5632016
 
 2.94e-11
 
 2.60e-08
  
 
  
 6
 
 152128258
 
 152129036
 
  PDF   STATS   ENSEMBL   UCSC 
 
  779
 
 20
 
 34514
 
 gene;island
 
 0.487199
 
 0.0070514
 
 ESR1 (margin: ESR1; RP3-443C4.2)
 
  7.7307741
 
 5.80e-11
 
 4.35e-08
  
 
  
 7
 
  95025736
 
  95026248
 
  PDF   STATS   ENSEMBL   UCSC 
 
  513
 
 19
 
 37726
 
 tss
 
 0.169020
 
 0.9708057
 
 PON1; PON3 (margin: PON1; PON3; PON2)
 
  7.1723338
 
 5.66e-11
 
 4.35e-08
  
 
  
 17
 
   1956958
 
   1958478
 
  PDF   STATS   ENSEMBL   UCSC 
 
 1521
 
 45
 
 17001
 
 tss;island
 
 0.423165
 
 0.0202988
 
 HIC1 (margin: DPH1; OVCA2; HIC1; RP11-667K14.4; RP11-667K14.3; MIR132; MIR212; SMG6)
 
  4.0439949
 
 9.96e-11
 
 6.93e-08
  
 
  
 10
 
 134599783
 
 134600998
 
  PDF   STATS   ENSEMBL   UCSC 
 
 1216
 
 30
 
 5450
 
 tss;island
 
 0.849043
 
 0.9792833
 
 RP11-288G11.3; NKX6-2 (margin: INPP5A; RP11-288G11.3; NKX6-2)
 
  3.5120254
 
 1.09e-10
 
 7.05e-08
  
 
  
 10
 
 135050619
 
 135051351
 
  PDF   STATS   ENSEMBL   UCSC 
 
  733
 
 25
 
 5456
 
 tss
 
 0.548626
 
 0.9588739
 
 VENTX (margin: KNDC1; UTF1; VENTX; MIR202)
 
  3.8342431
 
 1.86e-10
 
 1.13e-07
  
 
  
 7
 
  27154262
 
  27155548
 
  PDF   STATS   ENSEMBL   UCSC 
 
 1287
 
 18
 
 36931
 
 gene;island
 
 0.005790
 
 0.9695520
 
 HOXA-AS2; HOXA3 (margin: HOXA-AS2; HOXA2; HOXA3)
 
 11.7091124
 
 2.20e-10
 
 1.16e-07
  
 
  
 7
 
  93519855
 
  93520566
 
  PDF   STATS   ENSEMBL   UCSC 
 
  712
 
 19
 
 38669
 
 island
 
 0.833285
 
 0.0391704
 
 GNGT1; AC002076.10; TFPI2 (margin: GNGT1; AC002076.10; TFPI2)
 
  6.7390501
 
 2.26e-10
 
 1.16e-07
  
 
  
 15
 
  83953690
 
  83954395
 
  PDF   STATS   ENSEMBL   UCSC 
 
  706
 
 18
 
 13865
 
 tss;island
 
 0.122262
 
 0.9269946
 
 RP11-382A20.4; BNC1 (margin: RP11-382A20.4; BNC1)
 
  6.3299373
 
 2.20e-10
 
 1.16e-07
  
 
  
 6
 
  30418494
 
  30419612
 
  PDF   STATS   ENSEMBL   UCSC 
 
 1119
 
 32
 
 35680
 
 island
 
 0.463818
 
 0.9128475
 
  (margin: )
 
  3.7441739
 
 3.51e-10
 
 1.71e-07
  
 
  
 7
 
  27204663
 
  27205658
 
  PDF   STATS   ENSEMBL   UCSC 
 
  996
 
 17
 
 38356
 
 island
 
 0.085212
 
 0.9647329
 
 HOXA9; RP1-170O19.20 (margin: HOXA-AS3; RP1-170O19.21; HOXA-AS4; HOXA7; HOXA9; RP1-170O19.20; MIR196B; HOXA10)
 
  8.0077838
 
 8.57e-10
 
 3.90e-07
  
 
  
 6
 
 152128258
 
 152128805
 
  PDF   STATS   ENSEMBL   UCSC 
 
  548
 
 18
 
 35305
 
 tss
 
 0.487199
 
 0.0087769
 
 ESR1 (margin: ESR1; RP3-443C4.2)
 
  7.7307741
 
 8.82e-10
 
 3.90e-07
  
 
  
 6
 
  32811495
 
  32812708
 
  PDF   STATS   ENSEMBL   UCSC 
 
 1214
 
 44
 
 34151
 
 gene;island
 
 0.007943
 
 0.3346682
 
 XXbac-BPG246D15.8; PSMB9; PSMB8; TAP1 (margin: XXbac-BPG246D15.8; PSMB9; TAP2; PSMB8; TAP1)
 
  4.9679151
 
 1.30e-09
 
 5.51e-07
  
 
  
 6
 
  29894619
 
  29895260
 
  PDF   STATS   ENSEMBL   UCSC 
 
  642
 
 25
 
 35642
 
 island
 
 0.080932
 
 0.9202888
 
 HLA-K; HCG4B (margin: HLA-K; HLA-U; HCG4B)
 
  6.5929154
 
 1.56e-09
 
 6.31e-07
  
 
  
 10
 
 135050619
 
 135051581
 
  PDF   STATS   ENSEMBL   UCSC 
 
  963
 
 28
 
 6348
 
 island
 
 0.550924
 
 0.9565341
 
 VENTX (margin: KNDC1; UTF1; VENTX; MIR202)
 
  3.6006036
 
 1.65e-09
 
 6.41e-07
  
 
  
 6
 
  33172817
 
  33173581
 
  PDF   STATS   ENSEMBL   UCSC 
 
  765
 
 17
 
 34228
 
 gene;island
 
 0.007682
 
 0.3879221
 
 SLC39A7; HSD17B8 (margin: RNY4P10; SLC39A7; HSD17B8; MIR219-1; RING1; ZNF70P1; RXRB)
 
  7.1111112
 
 1.71e-09
 
 6.42e-07
  
 
  
 11
 
   2292361
 
   2293309
 
  PDF   STATS   ENSEMBL   UCSC 
 
  949
 
 32
 
 7064
 
 tss;island
 
 0.760247
 
 0.5058157
 
 ASCL2 (margin: ASCL2)
 
  1.7188216
 
 1.90e-09
 
 6.85e-07
  
 
  
 1
 
  92949337
 
  92950836
 
  PDF   STATS   ENSEMBL   UCSC 
 
 1500
 
 31
 
 635
 
 gene;island
 
 0.245164
 
 0.9744740
 
 GFI1 (margin: GFI1)
 
  6.5945899
 
 2.50e-09
 
 8.71e-07
  
 
  
 18
 
  35145983
 
  35146770
 
  PDF   STATS   ENSEMBL   UCSC 
 
  788
 
 21
 
 19477
 
 island
 
 0.717615
 
 0.0084481
 
 CELF4 (margin: CELF4)
 
  6.6534429
 
 3.40e-09
 
 1.10e-06
  
 
  
 6
 
  31148332
 
  31148748
 
  PDF   STATS   ENSEMBL   UCSC 
 
  417
 
 16
 
 35754
 
 island
 
 0.973725
 
 0.5069406
 
 POU5F1 (margin: POU5F1; PSORS1C3)
 
  5.4270073
 
 3.33e-09
 
 1.10e-06
  
 
  
 6
 
  29893926
 
  29894341
 
  PDF   STATS   ENSEMBL   UCSC 
 
  416
 
 16
 
 34757
 
 tss;island
 
 0.060563
 
 0.9093362
 
 HLA-K; HCG4B (margin: HLA-K; HLA-U; HCG4B)
 
  7.5018779
 
 6.65e-09
 
 2.09e-06
  
 
  
 1
 
  92949447
 
  92950836
 
  PDF   STATS   ENSEMBL   UCSC 
 
 1390
 
 29
 
 1751
 
 tss
 
 0.243322
 
 0.9719233
 
 GFI1 (margin: GFI1)
 
  6.7291161
 
 1.05e-08
 
 3.21e-06
  
 
  
 5
 
 112073348
 
 112073769
 
  PDF   STATS   ENSEMBL   UCSC 
 
  422
 
 15
 
 31713
 
 gene
 
 0.006453
 
 0.4363533
 
 APC (margin: APC)
 
  7.1805135
 
 1.29e-08
 
 3.49e-06
  
 
  
 3
 
  73673893
 
  73674702
 
  PDF   STATS   ENSEMBL   UCSC 
 
  810
 
 15
 
 29286
 
 island
 
 0.642287
 
 0.0102182
 
 PDZRN3-AS1; PDZRN3 (margin: PDZRN3-AS1; PDZRN3)
 
  6.8880778
 
 1.29e-08
 
 3.49e-06
  
 
  
 19
 
  37329090
 
  37329859
 
  PDF   STATS   ENSEMBL   UCSC 
 
  770
 
 15
 
 21947
 
 island
 
 0.593467
 
 0.0085941
 
 ZNF790 (margin: CTD-2162K18.5; ZNF345; ZNF790)
 
  6.7002753
 
 1.29e-08
 
 3.49e-06
  
 
  
 6
 
  30431563
 
  30431860
 
  PDF   STATS   ENSEMBL   UCSC 
 
  298
 
 15
 
 35682
 
 island
 
 0.239452
 
 0.9453876
 
  (margin: TMPOP1; SUCLA2P1)
 
  5.8454414
 
 1.29e-08
 
 3.49e-06
  
 
  
 18
 
  35146001
 
  35146770
 
  PDF   STATS   ENSEMBL   UCSC 
 
  770
 
 20
 
 19246
 
 tss
 
 0.730509
 
 0.0079676
 
 CELF4 (margin: CELF4)
 
  6.5802797
 
 1.33e-08
 
 3.50e-06
  
 
  
 6
 
 133561614
 
 133562494
 
  PDF   STATS   ENSEMBL   UCSC 
 
  881
 
 38
 
 36486
 
 island
 
 0.829707
 
 0.9719626
 
 EYA4 (margin: EYA4)
 
  2.6386265
 
 1.52e-08
 
 3.90e-06
  
 
  
 6
 
  29910755
 
  29911558
 
  PDF   STATS   ENSEMBL   UCSC 
 
  804
 
 22
 
 33702
 
 gene;island
 
 0.052525
 
 0.8184463
 
 HLA-A; HCG4P5 (margin: HLA-U; HLA-A; HCG4P5)
 
  6.1289060
 
 2.17e-08
 
 5.43e-06
  
 
  
 5
 
 135415693
 
 135416613
 
  PDF   STATS   ENSEMBL   UCSC 
 
  921
 
 16
 
 33146
 
 island
 
 0.538887
 
 0.9708696
 
 VTRNA2-1 (margin: VTRNA2-1)
 
  4.5745207
 
 2.33e-08
 
 5.67e-06
  
 
  
 11
 
  12030088
 
  12031508
 
  PDF   STATS   ENSEMBL   UCSC 
 
 1421
 
 21
 
 8065
 
 island
 
 0.039606
 
 0.9715218
 
 DKK3 (margin: DKK3)
 
  9.2598276
 
 2.72e-08
 
 6.41e-06
  
 
  
 6
 
 133561614
 
 133562492
 
  PDF   STATS   ENSEMBL   UCSC 
 
  879
 
 37
 
 35231
 
 tss
 
 0.813407
 
 0.9704296
 
 EYA4 (margin: EYA4)
 
  2.6054610
 
 2.76e-08
 
 6.41e-06
  
 
  
 7
 
  27154262
 
  27155039
 
  PDF   STATS   ENSEMBL   UCSC 
 
  778
 
 14
 
 37490
 
 tss
 
 0.003101
 
 0.9695520
 
 HOXA-AS2; HOXA3 (margin: HOXA-AS2; HOXA2; HOXA3)
 
 12.8644193
 
 4.99e-08
 
 9.34e-06
  
 
  
 14
 
  75593334
 
  75594058
 
  PDF   STATS   ENSEMBL   UCSC 
 
  725
 
 14
 
 13013
 
 island
 
 0.006478
 
 0.9837457
 
 RP11-950C14.7; NEK9 (margin: RP11-950C14.7; NEK9; HIF1AP1; TMED10)
 
 12.4986705
 
 4.99e-08
 
 9.34e-06
  
 
  
 2
 
 178257176
 
 178257923
 
  PDF   STATS   ENSEMBL   UCSC 
 
  748
 
 14
 
 22943
 
 gene;tss;island
 
 0.011288
 
 0.9538975
 
 AGPS; NFE2L2; AC074286.1 (margin: AGPS; NFE2L2; AC074286.1)
 
 11.5650541
 
 4.99e-08
 
 9.34e-06
  
 
  
 19
 
  37406932
 
  37407486
 
  PDF   STATS   ENSEMBL   UCSC 
 
  555
 
 14
 
 20732
 
 tss;island
 
 0.825523
 
 0.0072321
 
 ZNF568; ZNF829 (margin: ZNF345; ZNF568; ZNF829)
 
  9.3080042
 
 4.99e-08
 
 9.34e-06
  
 
  
 1
 
 220921216
 
 220922217
 
  PDF   STATS   ENSEMBL   UCSC 
 
 1002
 
 14
 
 4219
 
 island
 
 0.534819
 
 0.0100635
 
 MARC2 (margin: MARC2)
 
  7.0115354
 
 4.99e-08
 
 9.34e-06
  
 
  
 6
 
  31126599
 
  31127379
 
  PDF   STATS   ENSEMBL   UCSC 
 
  781
 
 21
 
 34800
 
 tss
 
 0.007658
 
 0.6823610
 
 TCF19; CCHCR1 (margin: TCF19; CCHCR1; POU5F1)
 
  6.7650925
 
 4.34e-08
 
 9.34e-06
  
 
  
 17
 
   6899085
 
   6899888
 
  PDF   STATS   ENSEMBL   UCSC 
 
  804
 
 14
 
 17913
 
 island
 
 0.305123
 
 0.9793675
 
 ALOX12; AC027763.2; RP11-589P10.7; RP11-589P10.5 (margin: ALOX12; AC027763.2; RP11-589P10.7; RP11-589P10.5)
 
  6.6607890
 
 4.99e-08
 
 9.34e-06
  
 
  
 15
 
  79383385
 
  79383980
 
  PDF   STATS   ENSEMBL   UCSC 
 
  596
 
 14
 
 13842
 
 tss;island
 
 0.540917
 
 0.0088285
 
 RASGRF1 (margin: RASGRF1)
 
  6.3736953
 
 4.99e-08
 
 9.34e-06
  
 
  
 19
 
  11998457
 
  11999148
 
  PDF   STATS   ENSEMBL   UCSC 
 
  692
 
 14
 
 21586
 
 island
 
 0.170670
 
 0.9371514
 
 ZNF69 (margin: ZNF439; ZNF69)
 
  5.8950554
 
 4.99e-08
 
 9.34e-06
  
 
  
 6
 
  28601269
 
  28601519
 
  PDF   STATS   ENSEMBL   UCSC 
 
  251
 
 14
 
 35592
 
 island
 
 0.969866
 
 0.6265136
 
 RP11-373N24.2 (margin: RP11-373N24.2)
 
  4.2483461
 
 4.99e-08
 
 9.34e-06
  
 
  
 22
 
  27053195
 
  27053934
 
  PDF   STATS   ENSEMBL   UCSC 
 
  740
 
 15
 
 27379
 
 island
 
 0.211227
 
 0.9885787
 
 MIAT (margin: MIAT; ISCA2P1; CTA-373H7.7)
 
  8.3129972
 
 5.16e-08
 
 9.48e-06
  
 
  
 11
 
  14993378
 
  14995322
 
  PDF   STATS   ENSEMBL   UCSC 
 
 1945
 
 40
 
 8081
 
 island
 
 0.551579
 
 0.9542450
 
 CALCB; CALCA (margin: CALCB; CALCA)
 
  2.8218847
 
 5.34e-08
 
 9.64e-06
  
 
  
 6
 
  42927940
 
  42928546
 
  PDF   STATS   ENSEMBL   UCSC 
 
  607
 
 25
 
 36145
 
 island
 
 0.122307
 
 0.0146011
 
 GNMT (margin: RP3-475N16.1; GNMT; RPL24P4; PEX6)
 
  3.2707276
 
 5.98e-08
 
 1.06e-05
  
 
  
 12
 
  45270207
 
  45270896
 
  PDF   STATS   ENSEMBL   UCSC 
 
  690
 
 18
 
 9101
 
 gene;tss;island
 
 0.247954
 
 0.0197145
 
 NELL2 (margin: NELL2; Y_RNA)
 
  4.1774285
 
 8.22e-08
 
 1.43e-05
  
 
  
 11
 
  12030187
 
  12031508
 
  PDF   STATS   ENSEMBL   UCSC 
 
 1322
 
 20
 
 7153
 
 tss
 
 0.035832
 
 0.9709821
 
 DKK3 (margin: DKK3)
 
  9.1469378
 
 1.06e-07
 
 1.81e-05
  
 
  
 7
 
  37488162
 
  37489005
 
  PDF   STATS   ENSEMBL   UCSC 
 
  844
 
 15
 
 38416
 
 island
 
 0.858881
 
 0.0528746
 
 ELMO1 (margin: ELMO1)
 
  6.6085811
 
 1.55e-07
 
 2.60e-05
  
 
  
 18
 
  28622474
 
  28623159
 
  PDF   STATS   ENSEMBL   UCSC 
 
  686
 
 13
 
 19446
 
 island
 
 0.013858
 
 0.9699441
 
 DSC3 (margin: DSC3)
 
 11.2109042
 
 1.92e-07
 
 2.60e-05
  
 
  
 11
 
  12030088
 
  12030914
 
  PDF   STATS   ENSEMBL   UCSC 
 
  827
 
 13
 
 6511
 
 gene
 
 0.022862
 
 0.9715218
 
 DKK3 (margin: DKK3)
 
 10.0290852
 
 1.92e-07
 
 2.60e-05
  
 
  
 7
 
  99155673
 
  99156387
 
  PDF   STATS   ENSEMBL   UCSC 
 
  715
 
 13
 
 37760
 
 tss;island
 
 0.007404
 
 0.8961135
 
 ZNF655; FAM200A (margin: ZNF655; FAM200A)
 
  9.9687859
 
 1.92e-07
 
 2.60e-05
  
 
  
 4
 
 175750109
 
 175751001
 
  PDF   STATS   ENSEMBL   UCSC 
 
  893
 
 13
 
 31438
 
 island
 
 0.913758
 
 0.0115587
 
 ADAM29; GLRA3 (margin: ADAM29; GLRA3)
 
  9.6579944
 
 1.92e-07
 
 2.60e-05
  
 
  
 17
 
   7530871
 
   7531394
 
  PDF   STATS   ENSEMBL   UCSC 
 
  524
 
 13
 
 16406
 
 gene;tss;island
 
 0.003860
 
 0.4619718
 
 SHBG; SAT2 (margin: SHBG; SAT2)
 
  8.5872799
 
 1.92e-07
 
 2.60e-05
  
 
  
 19
 
   9434309
 
   9435206
 
  PDF   STATS   ENSEMBL   UCSC 
 
  898
 
 13
 
 21496
 
 island
 
 0.047853
 
 0.8727097
 
 ZNF559; ZNF177 (margin: ZNF559; ZNF177)
 
  8.3898881
 
 1.92e-07
 
 2.60e-05
  
 
  
 7
 
  93520074
 
  93520566
 
  PDF   STATS   ENSEMBL   UCSC 
 
  493
 
 13
 
 37715
 
 tss
 
 0.839425
 
 0.0310396
 
 GNGT1; AC002076.10; TFPI2 (margin: GNGT1; AC002076.10; TFPI2)
 
  8.0817362
 
 1.92e-07
 
 2.60e-05
  
 
  
 7
 
  45961772
 
  45962236
 
  PDF   STATS   ENSEMBL   UCSC 
 
  465
 
 13
 
 37588
 
 tss;island
 
 0.840260
 
 0.0314236
 
 IGFBP3 (margin: IGFBP3)
 
  7.9790720
 
 1.92e-07
 
 2.60e-05
  
 
  
 19
 
  54372324
 
  54373043
 
  PDF   STATS   ENSEMBL   UCSC 
 
  720
 
 13
 
 20209
 
 gene;island
 
 0.636065
 
 0.0068056
 
 MYADM; AC008753.6 (margin: AC008440.10; MYADM; PRKCG; AC008753.6; AC008440.5)
 
  7.5731122
 
 1.92e-07
 
 2.60e-05
  
 
  
 19
 
  37064156
 
  37064886
 
  PDF   STATS   ENSEMBL   UCSC 
 
  731
 
 13
 
 20003
 
 gene;island
 
 0.009136
 
 0.3961969
 
 AC092295.7; ZNF529 (margin: AC092295.7; ZNF529)
 
  6.4820869
 
 1.92e-07
 
 2.60e-05
  
 
  
 12
 
  81471311
 
  81472177
 
  PDF   STATS   ENSEMBL   UCSC 
 
  867
 
 13
 
 10650
 
 island
 
 0.536744
 
 0.0112548
 
 ACSS3 (margin: ACSS3)
 
  6.4588143
 
 1.92e-07
 
 2.60e-05
  
 
  
 19
 
  37329330
 
  37329859
 
  PDF   STATS   ENSEMBL   UCSC 
 
  530
 
 13
 
 20730
 
 tss
 
 0.558811
 
 0.0114197
 
 ZNF790 (margin: CTD-2162K18.5; ZNF345; ZNF790)
 
  6.2238295
 
 1.92e-07
 
 2.60e-05
  
 
  
 3
 
  44902933
 
  44903730
 
  PDF   STATS   ENSEMBL   UCSC 
 
  798
 
 13
 
 29044
 
 island
 
 0.021293
 
 0.4904979
 
 KIF15; TMEM42; MIR564 (margin: KIF15; TMEM42; MIR564)
 
  5.8110226
 
 1.92e-07
 
 2.60e-05
  
 
  
 11
 
   2444462
 
   2445442
 
  PDF   STATS   ENSEMBL   UCSC 
 
  981
 
 17
 
 7072
 
 tss;island
 
 0.281698
 
 0.8813573
 
 TRPM5 (margin: TRPM5)
 
  4.7768112
 
 1.67e-07
 
 2.60e-05
  
 
  
 6
 
  31126599
 
  31127527
 
  PDF   STATS   ENSEMBL   UCSC 
 
  929
 
 22
 
 33860
 
 gene;island
 
 0.009629
 
 0.7013902
 
 TCF19; CCHCR1 (margin: TCF19; CCHCR1; POU5F1)
 
  6.4853907
 
 1.96e-07
 
 2.62e-05
  
 
  
 17
 
  39968362
 
  39968871
 
  PDF   STATS   ENSEMBL   UCSC 
 
  510
 
 14
 
 17345
 
 tss;island
 
 0.509380
 
 0.0077893
 
 FKBP10; LEPREL4 (margin: FKBP10; LEPREL4)
 
  6.7938865
 
 1.99e-07
 
 2.63e-05
  
 
  
 6
 
  29855110
 
  29855636
 
  PDF   STATS   ENSEMBL   UCSC 
 
  527
 
 16
 
 35639
 
 island
 
 0.040919
 
 0.6616663
 
 HLA-H; HCG4P7 (margin: HLA-H; HLA-T; HCG4P7)
 
  6.7984857
 
 2.23e-07
 
 2.90e-05
  
 
  
 6
 
  42927940
 
  42928497
 
  PDF   STATS   ENSEMBL   UCSC 
 
  558
 
 22
 
 34985
 
 tss
 
 0.253324
 
 0.0159774
 
 GNMT (margin: RP3-475N16.1; GNMT; RPL24P4; PEX6)
 
  3.6557351
 
 2.35e-07
 
 3.02e-05
  
 
  
 4
 
 176986621
 
 176987577
 
  PDF   STATS   ENSEMBL   UCSC 
 
  957
 
 14
 
 31440
 
 island
 
 0.612494
 
 0.0080488
 
 WDR17 (margin: WDR17)
 
  7.7400502
 
 3.49e-07
 
 4.42e-05
  
 
  
 7
 
  12609907
 
  12610833
 
  PDF   STATS   ENSEMBL   UCSC 
 
  927
 
 15
 
 38267
 
 island
 
 0.431974
 
 0.0098562
 
 SCIN; AC005281.2 (margin: SCIN; AC005281.2)
 
  6.2754030
 
 3.87e-07
 
 4.83e-05
  
 
  
 11
 
   2481857
 
   2482929
 
  PDF   STATS   ENSEMBL   UCSC 
 
 1073
 
 17
 
 6460
 
 gene
 
 0.476189
 
 0.9546653
 
 KCNQ1 (margin: KCNQ1)
 
  4.9669740
 
 4.35e-07
 
 5.37e-05
  
 
  
 1
 
  50489240
 
  50489954
 
  PDF   STATS   ENSEMBL   UCSC 
 
  715
 
 15
 
 3249
 
 island
 
 0.891673
 
 0.0237983
 
 AGBL4 (margin: MTND2P29; AGBL4)
 
  8.9195347
 
 5.80e-07
 
 7.07e-05
  
 
  
 12
 
  21809973
 
  21810762
 
  PDF   STATS   ENSEMBL   UCSC 
 
  790
 
 12
 
 9080
 
 gene
 
 0.007273
 
 0.9760241
 
 LDHB (margin: RP11-59N23.3; LDHB)
 
 12.9187795
 
 7.40e-07
 
 7.21e-05
  
 
  
 2
 
  27603547
 
  27604131
 
  PDF   STATS   ENSEMBL   UCSC 
 
  585
 
 12
 
 24181
 
 island
 
 0.013656
 
 0.9738135
 
 ZNF513; PPM1G (margin: SNX17; EIF2B4; ZNF513; PPM1G; FTH1P3)
 
 12.0409855
 
 7.40e-07
 
 7.21e-05
  
 
  
 6
 
  28979210
 
  28979498
 
  PDF   STATS   ENSEMBL   UCSC 
 
  289
 
 12
 
 35608
 
 island
 
 0.018409
 
 0.9775782
 
  (margin: ZNF311)
 
 11.6081634
 
 7.40e-07
 
 7.21e-05
  
 
  
 7
 
  25219543
 
  25220162
 
  PDF   STATS   ENSEMBL   UCSC 
 
  620
 
 12
 
 38322
 
 island
 
 0.011678
 
 0.9656896
 
 C7orf31 (margin: AC004129.7; C7orf31)
 
 11.5554940
 
 7.40e-07
 
 7.21e-05
  
 
  
 7
 
 134143823
 
 134144288
 
  PDF   STATS   ENSEMBL   UCSC 
 
  466
 
 12
 
 38935
 
 island
 
 0.024656
 
 0.9781448
 
 AKR1B1 (margin: AKR1B1)
 
  9.6987272
 
 7.40e-07
 
 7.21e-05
  
 
  
 10
 
    530635
 
    531584
 
  PDF   STATS   ENSEMBL   UCSC 
 
  950
 
 12
 
 4441
 
 gene;island
 
 0.099031
 
 0.9743791
 
 DIP2C (margin: DIP2C)
 
  8.1243333
 
 7.40e-07
 
 7.21e-05
  
 
  
 6
 
  30653167
 
  30653799
 
  PDF   STATS   ENSEMBL   UCSC 
 
  633
 
 12
 
 33804
 
 gene;island
 
 0.045732
 
 0.8991163
 
 PPP1R18 (margin: PPP1R18; NRM; RPL7P4)
 
  7.6715318
 
 7.40e-07
 
 7.21e-05
  
 
  
 3
 
  73674074
 
  73674702
 
  PDF   STATS   ENSEMBL   UCSC 
 
  629
 
 12
 
 28446
 
 tss
 
 0.687794
 
 0.0108384
 
 PDZRN3-AS1; PDZRN3 (margin: PDZRN3-AS1; PDZRN3)
 
  7.4769376
 
 7.40e-07
 
 7.21e-05
  
 
  
 13
 
  43566114
 
  43566642
 
  PDF   STATS   ENSEMBL   UCSC 
 
  529
 
 12
 
 11698
 
 island
 
 0.127893
 
 0.9670586
 
 EPSTI1 (margin: EPSTI1)
 
  7.2060117
 
 7.40e-07
 
 7.21e-05
  
 
  
 4
 
 144480206
 
 144480740
 
  PDF   STATS   ENSEMBL   UCSC 
 
  535
 
 12
 
 31337
 
 island
 
 0.969658
 
 0.2442413
 
 SMARCA5; GUSBP5 (margin: SMARCA5; GUSBP5; RP11-481K16.2)
 
  7.1839418
 
 7.40e-07
 
 7.21e-05
  
 
  
 8
 
  12990213
 
  12991196
 
  PDF   STATS   ENSEMBL   UCSC 
 
  984
 
 12
 
 39212
 
 gene;island
 
 0.391183
 
 0.0051997
 
 DLC1 (margin: DLC1)
 
  7.1192942
 
 7.40e-07
 
 7.21e-05
  
 
  
 8
 
   1764878
 
   1765477
 
  PDF   STATS   ENSEMBL   UCSC 
 
  600
 
 12
 
 40038
 
 island
 
 0.377028
 
 0.9859677
 
 MIR596 (margin: MIR596; ARHGEF10)
 
  6.7246163
 
 7.40e-07
 
 7.21e-05
  
 
  
 19
 
  37663410
 
  37663856
 
  PDF   STATS   ENSEMBL   UCSC 
 
  447
 
 12
 
 20734
 
 tss
 
 0.409742
 
 0.0102753
 
 CTC-454I21.3; ZNF585A (margin: CTC-454I21.3; ZNF585A; ZNF585B)
 
  5.9655343
 
 7.40e-07
 
 7.21e-05
  
 
  
 2
 
  75426726
 
  75427699
 
  PDF   STATS   ENSEMBL   UCSC 
 
  974
 
 12
 
 23468
 
 tss;island
 
 0.387107
 
 0.0139468
 
 TACR1 (margin: RP11-355F16.1; TACR1)
 
  5.8814998
 
 7.40e-07
 
 7.21e-05
  
 
  
 20
 
  36793608
 
  36794002
 
  PDF   STATS   ENSEMBL   UCSC 
 
  395
 
 12
 
 26152
 
 island
 
 0.682876
 
 0.0253611
 
 TGM2 (margin: TGM2)
 
  5.7928387
 
 7.40e-07
 
 7.21e-05
  
 
  
 19
 
   9545862
 
   9546377
 
  PDF   STATS   ENSEMBL   UCSC 
 
  516
 
 12
 
 21498
 
 island
 
 0.013176
 
 0.3850013
 
 ZNF266 (margin: ZNF266)
 
  5.5456898
 
 7.40e-07
 
 7.21e-05
  
 
  
 7
 
 149570931
 
 149571373
 
  PDF   STATS   ENSEMBL   UCSC 
 
  443
 
 12
 
 37269
 
 gene;island
 
 0.503133
 
 0.0174614
 
 ATP6V0E2; ATP6V0E2-AS1 (margin: ZNF862; ATP6V0E2; ATP6V0E2-AS1)
 
  5.5084875
 
 7.40e-07
 
 7.21e-05
  
 
  
 4
 
 141489799
 
 141490428
 
  PDF   STATS   ENSEMBL   UCSC 
 
  630
 
 12
 
 31327
 
 island
 
 0.624971
 
 0.9776419
 
 UCP1 (margin: RN7SL152P; UCP1)
 
  4.6841453
 
 7.40e-07
 
 7.21e-05
  
 
  
 20
 
  13200685
 
  13200992
 
  PDF   STATS   ENSEMBL   UCSC 
 
  308
 
 12
 
 26007
 
 island
 
 0.847832
 
 0.9855137
 
 ISM1 (margin: ISM1)
 
  3.3086408
 
 7.40e-07
 
 7.21e-05
  
 
  
 11
 
  86383182
 
  86383585
 
  PDF   STATS   ENSEMBL   UCSC 
 
  404
 
 12
 
 6854
 
 gene
 
 0.796912
 
 0.9611276
 
 ME3 (margin: ME3)
 
  2.8505365
 
 7.40e-07
 
 7.21e-05
  
 
  
 3
 
 138665654
 
 138666473
 
  PDF   STATS   ENSEMBL   UCSC 
 
  820
 
 13
 
 28626
 
 tss;island
 
 0.445298
 
 0.9830046
 
 C3orf72; FOXL2 (margin: C3orf72; RP11-548O1.3; FOXL2)
 
  6.2481806
 
 7.69e-07
 
 7.42e-05
  
 
  
 11
 
  14993866
 
  14995322
 
  PDF   STATS   ENSEMBL   UCSC 
 
 1457
 
 34
 
 7167
 
 tss
 
 0.575779
 
 0.9612734
 
 CALCB; CALCA (margin: CALCB; CALCA)
 
  3.0486805
 
 7.89e-07
 
 7.54e-05
  
 
  
 6
 
  33084420
 
  33085063
 
  PDF   STATS   ENSEMBL   UCSC 
 
  644
 
 20
 
 34188
 
 gene;island
 
 0.090258
 
 0.9489495
 
 HLA-DPB2 (margin: HLA-DPB2; COL11A2P1)
 
  5.9935055
 
 9.25e-07
 
 8.75e-05
  
 
  
 7
 
  27225058
 
  27225897
 
  PDF   STATS   ENSEMBL   UCSC 
 
  840
 
 23
 
 36942
 
 gene
 
 0.833675
 
 0.9598710
 
 HOXA11-AS; HOXA11; RP1-170O19.14 (margin: HOXA11-AS; RP1-170O19.20; HOXA10; HOXA11; RP1-170O19.14; HOXA13)
 
  2.0400096
 
 1.01e-06
 
 9.49e-05
  
 
  
 13
 
  96296708
 
  96297338
 
  PDF   STATS   ENSEMBL   UCSC 
 
  631
 
 12
 
 11827
 
 island
 
 0.021332
 
 0.9846144
 
 DZIP1 (margin: DZIP1; DNAJC3-AS1)
 
 10.8876312
 
 1.48e-06
 
 1.37e-04
  
 
  
 6
 
  30070738
 
  30071612
 
  PDF   STATS   ENSEMBL   UCSC 
 
  875
 
 27
 
 33720
 
 gene;island
 
 0.932903
 
 0.0108320
 
 TRIM31-AS1; TRIM31 (margin: TRIM31-AS1; TRIM31)
 
  9.9356573
 
 1.95e-06
 
 1.79e-04
  
 
  
 6
 
  31759728
 
  31760825
 
  PDF   STATS   ENSEMBL   UCSC 
 
 1098
 
 20
 
 33957
 
 gene;island
 
 0.190099
 
 0.9412938
 
 VARS (margin: VARS; LSM2)
 
  5.7960161
 
 2.00e-06
 
 1.82e-04
  
 
  
 6
 
  27106988
 
  27107756
 
  PDF   STATS   ENSEMBL   UCSC 
 
  769
 
 11
 
 33617
 
 gene;island
 
 0.007674
 
 0.9718681
 
 HIST1H4I; HIST1H2BK (margin: HIST1H2AG; HIST1H4I; HIST1H2AH; MIR3143; HIST1H2BJ; HIST1H2BK)
 
 13.9322565
 
 2.84e-06
 
 1.96e-04
  
 
  
 2
 
  27603611
 
  27604131
 
  PDF   STATS   ENSEMBL   UCSC 
 
  521
 
 11
 
 23267
 
 tss
 
 0.009595
 
 0.9752453
 
 ZNF513; PPM1G (margin: SNX17; EIF2B4; ZNF513; PPM1G; FTH1P3)
 
 13.6257256
 
 2.84e-06
 
 1.96e-04
  
 
  
 7
 
  27195918
 
  27196555
 
  PDF   STATS   ENSEMBL   UCSC 
 
  638
 
 11
 
 38353
 
 island
 
 0.005620
 
 0.9797731
 
 HOXA-AS3; RP1-170O19.21; HOXA7 (margin: HOXA-AS3; RP1-170O19.21; HOXA-AS4; HOXA3; HOXA6; HOXA7; HOXA9; RP1-170O19.20)
 
 13.0324006
 
 2.84e-06
 
 1.96e-04
  
 
  
 12
 
  51566379
 
  51567112
 
  PDF   STATS   ENSEMBL   UCSC 
 
  734
 
 11
 
 10422
 
 island
 
 0.011744
 
 0.9731623
 
 TFCP2 (margin: RNU6-199P; TFCP2)
 
 11.6164717
 
 2.84e-06
 
 1.96e-04
  
 
  
 19
 
  37997675
 
  37998171
 
  PDF   STATS   ENSEMBL   UCSC 
 
  497
 
 11
 
 21957
 
 island
 
 0.884806
 
 0.0025733
 
 ZNF793; CTD-3064H18.1 (margin: ZNF793; CTD-3064H18.1; CTD-3064H18.2)
 
 11.2775408
 
 2.84e-06
 
 1.96e-04
  
 
  
 3
 
  50378191
 
  50378664
 
  PDF   STATS   ENSEMBL   UCSC 
 
  474
 
 11
 
 29153
 
 island
 
 0.015913
 
 0.9746159
 
 ZMYND10-AS1; RASSF1; ZMYND10 (margin: ZMYND10-AS1; CYB561D2; XXcos-LUCA11.5; RASSF1; ZMYND10; NPRL2)
 
 11.2133056
 
 2.84e-06
 
 1.96e-04
  
 
  
 6
 
  27840957
 
  27841423
 
  PDF   STATS   ENSEMBL   UCSC 
 
  467
 
 11
 
 34728
 
 tss;island
 
 0.036927
 
 0.9843284
 
 HIST1H3I; HIST1H4L (margin: HIST1H2AL; HIST1H2BPS2; HIST1H1B; HIST1H3I; HIST1H4L)
 
 11.2060771
 
 2.84e-06
 
 1.96e-04
  
 
  
 5
 
 114937535
 
 114938459
 
  PDF   STATS   ENSEMBL   UCSC 
 
  925
 
 11
 
 31721
 
 gene;island
 
 0.018747
 
 0.9826513
 
 AC010226.4; TICAM2; TMED7-TICAM2 (margin: AC010226.4; TICAM2; TMED7-TICAM2; TMED7)
 
 10.8324952
 
 2.84e-06
 
 1.96e-04
  
 
  
 17
 
  19648718
 
  19649293
 
  PDF   STATS   ENSEMBL   UCSC 
 
  576
 
 11
 
 16484
 
 gene;island
 
 0.013915
 
 0.9507600
 
 ALDH3A1 (margin: RP11-311F12.2; AC005722.4; ALDH3A1)
 
 10.7214365
 
 2.84e-06
 
 1.96e-04
  
 
  
 10
 
 118032872
 
 118033370
 
  PDF   STATS   ENSEMBL   UCSC 
 
  499
 
 11
 
 5382
 
 tss;island
 
 0.869783
 
 0.0060861
 
 GFRA1 (margin: GFRA1)
 
 10.2585551
 
 2.84e-06
 
 1.96e-04
  
 
  
 7
 
 134143906
 
 134144288
 
  PDF   STATS   ENSEMBL   UCSC 
 
  383
 
 11
 
 37911
 
 tss
 
 0.024920
 
 0.9823423
 
 AKR1B1 (margin: AKR1B1)
 
  9.9733978
 
 2.84e-06
 
 1.96e-04
  
 
  
 13
 
  29292888
 
  29293454
 
  PDF   STATS   ENSEMBL   UCSC 
 
  567
 
 11
 
 11642
 
 island
 
 0.929417
 
 0.0162202
 
 SLC46A3 (margin: SLC46A3; CYP51P2)
 
  9.8319088
 
 2.84e-06
 
 1.96e-04
  
 
  
 5
 
  89854293
 
  89854979
 
  PDF   STATS   ENSEMBL   UCSC 
 
  687
 
 11
 
 32955
 
 island
 
 0.692951
 
 0.0119135
 
 GPR98 (margin: GPR98)
 
  9.7010737
 
 2.84e-06
 
 1.96e-04
  
 
  
 3
 
  48632484
 
  48633144
 
  PDF   STATS   ENSEMBL   UCSC 
 
  661
 
 11
 
 29090
 
 island
 
 0.013916
 
 0.9653426
 
 COL7A1 (margin: COL7A1; UQCRC1)
 
  9.6268194
 
 2.84e-06
 
 1.96e-04
  
 
  
 15
 
  45421578
 
  45422095
 
  PDF   STATS   ENSEMBL   UCSC 
 
  518
 
 11
 
 13646
 
 tss;island
 
 0.121457
 
 0.9794764
 
 DUOX1; DUOXA1 (margin: DUOXA2; DUOX1; DUOXA1)
 
  9.5968154
 
 2.84e-06
 
 1.96e-04
  
 
  
 19
 
  37569236
 
  37569736
 
  PDF   STATS   ENSEMBL   UCSC 
 
  501
 
 11
 
 21950
 
 island
 
 0.802375
 
 0.0068591
 
 ZNF420; CTD-2293H3.2 (margin: ZNF420; CTD-2293H3.2)
 
  8.2317568
 
 2.84e-06
 
 1.96e-04
  
 
  
 8
 
  61193715
 
  61194292
 
  PDF   STATS   ENSEMBL   UCSC 
 
  578
 
 11
 
 40329
 
 island
 
 0.823630
 
 0.0139948
 
 CA8 (margin: CA8)
 
  8.2096237
 
 2.84e-06
 
 1.96e-04
  
 
  
 6
 
  31275551
 
  31275881
 
  PDF   STATS   ENSEMBL   UCSC 
 
  331
 
 11
 
 35762
 
 island
 
 0.689049
 
 0.0148081
 
 XXbac-BPG248L24.10 (margin: XXbac-BPG248L24.13; XXbac-BPG248L24.10)
 
  7.8097707
 
 2.84e-06
 
 1.96e-04
  
 
  
 4
 
 144480206
 
 144480561
 
  PDF   STATS   ENSEMBL   UCSC 
 
  356
 
 11
 
 30614
 
 tss
 
 0.978698
 
 0.2447930
 
 SMARCA5; GUSBP5 (margin: SMARCA5; GUSBP5; RP11-481K16.2)
 
  7.4459387
 
 2.84e-06
 
 1.96e-04
  
 
  
 5
 
 112073348
 
 112073544
 
  PDF   STATS   ENSEMBL   UCSC 
 
  197
 
 11
 
 32214
 
 tss
 
 0.006453
 
 0.4394419
 
 APC (margin: APC)
 
  7.1805135
 
 2.84e-06
 
 1.96e-04
  
 
  
 20
 
  44746392
 
  44747006
 
  PDF   STATS   ENSEMBL   UCSC 
 
  615
 
 11
 
 26215
 
 island
 
 0.129910
 
 0.9344710
 
 CD40 (margin: CD40)
 
  6.7001442
 
 2.84e-06
 
 1.96e-04
  
 
  
 2
 
  74730266
 
  74730948
 
  PDF   STATS   ENSEMBL   UCSC 
 
  683
 
 11
 
 22745
 
 gene;island
 
 0.020739
 
 0.5576626
 
 RP11-523H20.3; LBX2-AS1; LBX2; PCGF1 (margin: TTC31; RP11-523H20.3; LBX2-AS1; TLX2; LBX2; AC005041.17; PCGF1)
 
  6.6520810
 
 2.84e-06
 
 1.96e-04
  
 
  
 2
 
  17721431
 
  17722068
 
  PDF   STATS   ENSEMBL   UCSC 
 
  638
 
 11
 
 24094
 
 island
 
 0.032765
 
 0.6243980
 
 VSNL1 (margin: VSNL1)
 
  6.4647480
 
 2.84e-06
 
 1.96e-04
  
 
  
 4
 
  74964856
 
  74965278
 
  PDF   STATS   ENSEMBL   UCSC 
 
  423
 
 11
 
 31102
 
 island
 
 0.011064
 
 0.3637207
 
 CXCL2 (margin: CXCL2)
 
  6.2686749
 
 2.84e-06
 
 1.96e-04
  
 
  
 2
 
 201450323
 
 201450743
 
  PDF   STATS   ENSEMBL   UCSC 
 
  421
 
 11
 
 25003
 
 island
 
 0.413673
 
 0.9801815
 
 SGOL2; AOX1 (margin: SGOL2; AOX1)
 
  6.2013902
 
 2.84e-06
 
 1.96e-04
  
 
  
 7
 
 149570987
 
 149571373
 
  PDF   STATS   ENSEMBL   UCSC 
 
  387
 
 11
 
 37973
 
 tss
 
 0.503624
 
 0.0140687
 
 ATP6V0E2; ATP6V0E2-AS1 (margin: ZNF862; ATP6V0E2; ATP6V0E2-AS1)
 
  5.6748529
 
 2.84e-06
 
 1.96e-04
  
 
  
 2
 
  25391505
 
  25391911
 
  PDF   STATS   ENSEMBL   UCSC 
 
  407
 
 11
 
 24139
 
 island
 
 0.503666
 
 0.9670843
 
 POMC (margin: RP11-509E16.1; POMC)
 
  5.1379161
 
 2.84e-06
 
 1.96e-04
  
 
  
 1
 
  55007670
 
  55008516
 
  PDF   STATS   ENSEMBL   UCSC 
 
  847
 
 11
 
 3299
 
 island
 
 0.013112
 
 0.1057797
 
 ACOT11 (margin: HNRNPA1P63; ACOT11)
 
  4.5418798
 
 2.84e-06
 
 1.96e-04
  
 
  
 15
 
  45406327
 
  45406939
 
  PDF   STATS   ENSEMBL   UCSC 
 
  613
 
 13
 
 13645
 
 tss;island
 
 0.204804
 
 0.8482294
 
 DUOXA2; DUOX2 (margin: DUOXA2; DUOX2; DUOXA1)
 
  4.4420619
 
 2.31e-06
 
 1.96e-04
  
 
  
 11
 
  14913124
 
  14914012
 
  PDF   STATS   ENSEMBL   UCSC 
 
  889
 
 13
 
 8080
 
 island
 
 0.192657
 
 0.0076213
 
 CYP2R1 (margin: CYP2R1)
 
  4.4266301
 
 2.31e-06
 
 1.96e-04
  
 
  
 11
 
  31832710
 
  31833264
 
  PDF   STATS   ENSEMBL   UCSC 
 
  555
 
 11
 
 6562
 
 gene;island
 
 0.752034
 
 0.9732796
 
 RCN1; PAX6 (margin: RCN1; PAX6)
 
  3.7416583
 
 2.84e-06
 
 1.96e-04
  
 
  
 15
 
  45670865
 
  45671347
 
  PDF   STATS   ENSEMBL   UCSC 
 
  483
 
 13
 
 13333
 
 gene;tss;island
 
 0.869863
 
 0.9864906
 
 GATM (margin: GATM)
 
  3.7331970
 
 2.31e-06
 
 1.96e-04
  
 
  
 4
 
  76555547
 
  76556042
 
  PDF   STATS   ENSEMBL   UCSC 
 
  496
 
 11
 
 31107
 
 island
 
 0.079013
 
 0.4495094
 
 CDKL2 (margin: CDKL2; G3BP2)
 
  2.9849479
 
 2.84e-06
 
 1.96e-04
  
 
  
 6
 
  32063394
 
  32064258
 
  PDF   STATS   ENSEMBL   UCSC 
 
  865
 
 28
 
 34046
 
 gene;island
 
 0.783652
 
 0.9810936
 
 TNXB; ATF6B (margin: TNXB; ATF6B)
 
  2.7683296
 
 2.28e-06
 
 1.96e-04
  
 
  
 5
 
 101631990
 
 101632559
 
  PDF   STATS   ENSEMBL   UCSC 
 
  570
 
 12
 
 32986
 
 island
 
 0.953598
 
 0.0306345
 
 SLCO4C1 (margin: RN7SKP68; SLCO4C1)
 
  9.2449060
 
 2.96e-06
 
 2.02e-04
  
 
  
 19
 
  58446312
 
  58446988
 
  PDF   STATS   ENSEMBL   UCSC 
 
  677
 
 12
 
 22487
 
 island
 
 0.929211
 
 0.3685893
 
 ZNF418 (margin: ZNF418; ZNF256)
 
  4.5608702
 
 2.96e-06
 
 2.02e-04
  
 
  
 20
 
  21685834
 
  21686728
 
  PDF   STATS   ENSEMBL   UCSC 
 
  895
 
 14
 
 26042
 
 island
 
 0.614267
 
 0.9739677
 
 PAX1 (margin: PAX1)
 
  4.8828282
 
 3.34e-06
 
 2.26e-04
  
 
  
 12
 
  58012960
 
  58013687
 
  PDF   STATS   ENSEMBL   UCSC 
 
  728
 
 20
 
 9822
 
 tss;island
 
 0.714889
 
 0.9662860
 
 ARHGEF25; SLC26A10; AC025165.8 (margin: DTX3; ARHGEF25; SLC26A10; AC025165.8; B4GALNT1)
 
  2.5447558
 
 3.45e-06
 
 2.32e-04
  
 
  
 11
 
   2481857
 
   2482594
 
  PDF   STATS   ENSEMBL   UCSC 
 
  738
 
 15
 
 7076
 
 tss
 
 0.392645
 
 0.9436680
 
 KCNQ1 (margin: KCNQ1)
 
  4.1398550
 
 3.51e-06
 
 2.34e-04
  
 
  
 10
 
 135049991
 
 135050355
 
  PDF   STATS   ENSEMBL   UCSC 
 
  365
 
 13
 
 5455
 
 tss;island
 
 0.656817
 
 0.9843288
 
 VENTX (margin: KNDC1; UTF1; VENTX; MIR202)
 
  4.6206613
 
 3.65e-06
 
 2.42e-04
  
 
  
 11
 
  66034681
 
  66036138
 
  PDF   STATS   ENSEMBL   UCSC 
 
 1458
 
 58
 
 8500
 
 island
 
 0.282802
 
 0.0097424
 
 KLC2; RAB1B; RP11-867G23.1; RP11-867G23.2; RP11-867G23.3 (margin: KLC2; RAB1B; CNIH2; RP11-755F10.3; RP11-867G23.1; RP11-867G23.2; RP11-867G23.3; RP11-867G23.4)
 
  4.0813797
 
 4.18e-06
 
 2.75e-04
  
 
  
 6
 
  46702683
 
  46703670
 
  PDF   STATS   ENSEMBL   UCSC 
 
  988
 
 19
 
 36189
 
 island
 
 0.463165
 
 0.9300780
 
 PLA2G7 (margin: ANKRD66; PLA2G7)
 
  3.2340804
 
 5.23e-06
 
 3.42e-04
  
 
  
 6
 
  29894619
 
  29894908
 
  PDF   STATS   ENSEMBL   UCSC 
 
  290
 
 11
 
 34758
 
 tss
 
 0.038503
 
 0.9175490
 
 HLA-K; HCG4B (margin: HLA-K; HLA-U; HCG4B)
 
  8.1409963
 
 5.67e-06
 
 3.54e-04
  
 
  
 13
 
  92050675
 
  92051154
 
  PDF   STATS   ENSEMBL   UCSC 
 
  480
 
 11
 
 11814
 
 island
 
 0.586370
 
 0.0077824
 
 GPC5 (margin: GPC5)
 
  7.4680324
 
 5.67e-06
 
 3.54e-04
  
 
  
 7
 
  87563462
 
  87563931
 
  PDF   STATS   ENSEMBL   UCSC 
 
  470
 
 11
 
 38639
 
 island
 
 0.494824
 
 0.0107850
 
 ADAM22 (margin: ADAM22)
 
  6.8391143
 
 5.67e-06
 
 3.54e-04
  
 
  
 3
 
  44770851
 
  44771316
 
  PDF   STATS   ENSEMBL   UCSC 
 
  466
 
 11
 
 29041
 
 island
 
 0.273772
 
 0.9297942
 
 ZNF501 (margin: ZNF502; ZNF501; KIAA1143)
 
  6.4283335
 
 5.67e-06
 
 3.54e-04
  
 
  
 14
 
 102026086
 
 102026555
 
  PDF   STATS   ENSEMBL   UCSC 
 
  470
 
 11
 
 13158
 
 island
 
 0.518049
 
 0.9772537
 
 DIO3; DIO3OS (margin: DIO3; DIO3OS)
 
  4.8384081
 
 5.67e-06
 
 3.54e-04
  
 
  
 14
 
  96180319
 
  96181044
 
  PDF   STATS   ENSEMBL   UCSC 
 
  726
 
 11
 
 13113
 
 island
 
 0.587336
 
 0.9711114
 
 RP11-164H13.1; TCL1A (margin: RP11-164H13.1; TCL1A)
 
  3.2779835
 
 5.67e-06
 
 3.54e-04
  
 
  
 16
 
   3493336
 
   3494155
 
  PDF   STATS   ENSEMBL   UCSC 
 
  820
 
 11
 
 15056
 
 tss;island
 
 0.294576
 
 0.5275973
 
 LA16c-306E5.2; NAA60; ZNF597 (margin: LA16c-306E5.2; NAA60; ZNF597)
 
  1.4690836
 
 5.67e-06
 
 3.54e-04
  
 
  
 20
 
  61446962
 
  61448325
 
  PDF   STATS   ENSEMBL   UCSC 
 
 1364
 
 42
 
 25882
 
 tss;island
 
 0.564441
 
 0.8774159
 
 OGFR; COL9A3 (margin: OGFR; COL9A3; OGFR-AS1)
 
  1.7743296
 
 5.73e-06
 
 3.56e-04
  
 
  
 13
 
  20767498
 
  20768560
 
  PDF   STATS   ENSEMBL   UCSC 
 
 1063
 
 13
 
 11329
 
 tss;island
 
 0.238638
 
 0.9594946
 
 GJB2 (margin: LINC00556; GJB2)
 
  5.3150094
 
 5.77e-06
 
 3.56e-04
  
 
  
 11
 
  66034681
 
  66036038
 
  PDF   STATS   ENSEMBL   UCSC 
 
 1358
 
 55
 
 7460
 
 tss
 
 0.292714
 
 0.0096346
 
 KLC2; RAB1B; RP11-867G23.1; RP11-867G23.2; RP11-867G23.3 (margin: KLC2; RAB1B; CNIH2; RP11-755F10.3; RP11-867G23.1; RP11-867G23.2; RP11-867G23.3; RP11-867G23.4)
 
  4.2133468
 
 7.14e-06
 
 4.37e-04
  
 
  
 2
 
  29337946
 
  29338432
 
  PDF   STATS   ENSEMBL   UCSC 
 
  487
 
 10
 
 24198
 
 island
 
 0.008064
 
 0.9891859
 
 CLIP4 (margin: CLIP4)
 
 13.2602013
 
 1.08e-05
 
 4.82e-04
  
 
  
 22
 
  45608345
 
  45608713
 
  PDF   STATS   ENSEMBL   UCSC 
 
  369
 
 10
 
 26901
 
 gene
 
 0.010202
 
 0.9776878
 
 KIAA0930 (margin: KIAA0930; MIR1249)
 
 11.4542244
 
 1.08e-05
 
 4.82e-04
  
 
  
 3
 
 196065289
 
 196065688
 
  PDF   STATS   ENSEMBL   UCSC 
 
  400
 
 10
 
 28812
 
 tss
 
 0.008387
 
 0.9637754
 
 TM4SF19 (margin: TM4SF19; RNU6-910P; UBXN7)
 
 11.4540293
 
 1.08e-05
 
 4.82e-04
  
 
  
 7
 
  27209131
 
  27209582
 
  PDF   STATS   ENSEMBL   UCSC 
 
  452
 
 10
 
 38358
 
 island
 
 0.014704
 
 0.9774533
 
 HOXA-AS4; HOXA9; RP1-170O19.20; MIR196B; HOXA10 (margin: RP1-170O19.21; HOXA-AS4; HOXA7; HOXA9; RP1-170O19.20; MIR196B; HOXA10; HOXA11)
 
 11.2953470
 
 1.08e-05
 
 4.82e-04
  
 
  
 18
 
  28622811
 
  28623159
 
  PDF   STATS   ENSEMBL   UCSC 
 
  349
 
 10
 
 19220
 
 tss
 
 0.015294
 
 0.9709665
 
 DSC3 (margin: DSC3)
 
 11.1821010
 
 1.08e-05
 
 4.82e-04
  
 
  
 17
 
  19648846
 
  19649293
 
  PDF   STATS   ENSEMBL   UCSC 
 
  448
 
 10
 
 17194
 
 tss
 
 0.012939
 
 0.9391340
 
 ALDH3A1 (margin: RP11-311F12.2; AC005722.4; ALDH3A1)
 
 11.0549696
 
 1.08e-05
 
 4.82e-04
  
 
  
 3
 
 158390329
 
 158390821
 
  PDF   STATS   ENSEMBL   UCSC 
 
  493
 
 10
 
 28012
 
 gene
 
 0.957981
 
 0.0161807
 
 GFM1; LXN (margin: GFM1; LXN)
 
 10.6074694
 
 1.08e-05
 
 4.82e-04
  
 
  
 4
 
 175750507
 
 175751001
 
  PDF   STATS   ENSEMBL   UCSC 
 
  495
 
 10
 
 30686
 
 tss
 
 0.847449
 
 0.0139311
 
 ADAM29; GLRA3 (margin: ADAM29; GLRA3)
 
 10.2740578
 
 1.08e-05
 
 4.82e-04
  
 
  
 7
 
  27205159
 
  27205658
 
  PDF   STATS   ENSEMBL   UCSC 
 
  500
 
 10
 
 37496
 
 tss
 
 0.045967
 
 0.9660255
 
 HOXA9; RP1-170O19.20 (margin: HOXA-AS3; RP1-170O19.21; HOXA-AS4; HOXA7; HOXA9; RP1-170O19.20; MIR196B; HOXA10)
 
 10.0717523
 
 1.08e-05
 
 4.82e-04
  
 
  
 10
 
  17270087
 
  17270601
 
  PDF   STATS   ENSEMBL   UCSC 
 
  515
 
 10
 
 5578
 
 island
 
 0.136589
 
 0.9837831
 
 VIM; VIM-AS1 (margin: VIM; VIM-AS1; RP11-124N14.3)
 
  8.7755537
 
 1.08e-05
 
 4.82e-04
  
 
  
 22
 
  46481603
 
  46482023
 
  PDF   STATS   ENSEMBL   UCSC 
 
  421
 
 10
 
 27581
 
 island
 
 0.065337
 
 0.9648111
 
 FLJ27365 (margin: FLJ27365; MIR3619)
 
  8.7280251
 
 1.08e-05
 
 4.82e-04
  
 
  
 19
 
   9434309
 
   9434924
 
  PDF   STATS   ENSEMBL   UCSC 
 
  616
 
 10
 
 20422
 
 tss
 
 0.029063
 
 0.8000867
 
 ZNF559; ZNF177 (margin: ZNF559; ZNF177)
 
  8.5976496
 
 1.08e-05
 
 4.82e-04
  
 
  
 14
 
  24457893
 
  24458345
 
  PDF   STATS   ENSEMBL   UCSC 
 
  453
 
 10
 
 12723
 
 island
 
 0.010836
 
 0.7206862
 
 DHRS4L2; DHRS4-AS1 (margin: DHRS4L2; DHRS4-AS1)
 
  8.5764917
 
 1.08e-05
 
 4.82e-04
  
 
  
 8
 
  67940640
 
  67941178
 
  PDF   STATS   ENSEMBL   UCSC 
 
  539
 
 10
 
 40363
 
 island
 
 0.645954
 
 0.0071019
 
 PPP1R42 (margin: PPP1R42)
 
  8.5365471
 
 1.08e-05
 
 4.82e-04
  
 
  
 22
 
  27053195
 
  27053470
 
  PDF   STATS   ENSEMBL   UCSC 
 
  276
 
 10
 
 27037
 
 tss
 
 0.218240
 
 0.9883511
 
 MIAT (margin: MIAT; ISCA2P1; CTA-373H7.7)
 
  8.3953950
 
 1.08e-05
 
 4.82e-04
  
 
  
 1
 
  55266578
 
  55267293
 
  PDF   STATS   ENSEMBL   UCSC 
 
  716
 
 10
 
 3302
 
 island
 
 0.019140
 
 0.8959956
 
 TTC22 (margin: RP11-67L3.2; C1orf177; TTC22)
 
  8.1898610
 
 1.08e-05
 
 4.82e-04
  
 
  
 1
 
 153508511
 
 153508875
 
  PDF   STATS   ENSEMBL   UCSC 
 
  365
 
 10
 
 3763
 
 island
 
 0.005899
 
 0.6063518
 
 BX470102.3; S100A6; S100A5 (margin: BX470102.3; S100A6; S100A5; S100A4; S100A3)
 
  8.0849294
 
 1.08e-05
 
 4.82e-04
  
 
  
 2
 
 220083306
 
 220083951
 
  PDF   STATS   ENSEMBL   UCSC 
 
  646
 
 10
 
 25110
 
 island
 
 0.753240
 
 0.0101490
 
 ABCB6; ATG9A (margin: ZFAND2B; ANKZF1; ABCB6; ATG9A; AC068946.1)
 
  8.0737309
 
 1.08e-05
 
 4.82e-04
  
 
  
 6
 
  46138725
 
  46139019
 
  PDF   STATS   ENSEMBL   UCSC 
 
  295
 
 10
 
 35019
 
 tss
 
 0.532603
 
 0.0073672
 
 ENPP5 (margin: ENPP5)
 
  7.7724941
 
 1.08e-05
 
 4.82e-04
  
 
  
 3
 
  10206254
 
  10206731
 
  PDF   STATS   ENSEMBL   UCSC 
 
  478
 
 10
 
 28874
 
 island
 
 0.011123
 
 0.6296166
 
 IRAK2 (margin: IRAK2)
 
  7.4854207
 
 1.08e-05
 
 4.82e-04
  
 
  
 10
 
  73533035
 
  73533561
 
  PDF   STATS   ENSEMBL   UCSC 
 
  527
 
 10
 
 4624
 
 gene;island
 
 0.006436
 
 0.5471360
 
 CDH23; C10orf54 (margin: CDH23; C10orf54)
 
  7.2986971
 
 1.08e-05
 
 4.82e-04
  
 
  
 2
 
  12856626
 
  12857067
 
  PDF   STATS   ENSEMBL   UCSC 
 
  442
 
 10
 
 24085
 
 island
 
 0.186230
 
 0.0034520
 
 RP11-333O1.1; TRIB2 (margin: RP11-333O1.1; TRIB2)
 
  7.2799810
 
 1.08e-05
 
 4.82e-04
  
 
  
 19
 
  12175107
 
  12175719
 
  PDF   STATS   ENSEMBL   UCSC 
 
  613
 
 10
 
 21591
 
 island
 
 0.293095
 
 0.9372191
 
 ZNF844 (margin: ZNF844; RNA5SP466; CTD-2006C1.10; ZNF878)
 
  6.8891412
 
 1.08e-05
 
 4.82e-04
  
 
  
 6
 
  26188969
 
  26189597
 
  PDF   STATS   ENSEMBL   UCSC 
 
  629
 
 10
 
 35522
 
 island
 
 0.003306
 
 0.2791030
 
 HIST1H4D (margin: HIST1H2BE; HIST1H1PS1; HIST1H2BF; HIST1H4D; HIST1H3D; HIST1H2AD)
 
  6.8699069
 
 1.08e-05
 
 4.82e-04
  
 
  
 12
 
  89746101
 
  89746771
 
  PDF   STATS   ENSEMBL   UCSC 
 
  671
 
 10
 
 10664
 
 island
 
 0.002736
 
 0.2314672
 
 DUSP6 (margin: DUSP6)
 
  6.8384298
 
 1.08e-05
 
 4.82e-04
  
 
  
 20
 
  36793705
 
  36794002
 
  PDF   STATS   ENSEMBL   UCSC 
 
  298
 
 10
 
 25731
 
 tss
 
 0.638531
 
 0.0112265
 
 TGM2 (margin: TGM2)
 
  6.8371528
 
 1.08e-05
 
 4.82e-04
  
 
  
 15
 
  91497863
 
  91498497
 
  PDF   STATS   ENSEMBL   UCSC 
 
  635
 
 10
 
 14464
 
 island
 
 0.762567
 
 0.0267988
 
 UNC45A; RCCD1; AC068831.6 (margin: UNC45A; RCCD1; PRC1-AS1; AC068831.6; PRC1)
 
  6.8342377
 
 1.08e-05
 
 4.82e-04
  
 
  
 8
 
   1764878
 
   1765387
 
  PDF   STATS   ENSEMBL   UCSC 
 
  510
 
 10
 
 39535
 
 tss
 
 0.377028
 
 0.9859677
 
 MIR596 (margin: MIR596; ARHGEF10)
 
  6.7246163
 
 1.08e-05
 
 4.82e-04
  
 
  
 18
 
  43652172
 
  43652594
 
  PDF   STATS   ENSEMBL   UCSC 
 
  423
 
 10
 
 19484
 
 island
 
 0.744952
 
 0.0479561
 
 PSTPIP2 (margin: PSTPIP2; Y_RNA; ATP5A1)
 
  6.6242142
 
 1.08e-05
 
 4.82e-04
  
 
  
 13
 
  20805094
 
  20805679
 
  PDF   STATS   ENSEMBL   UCSC 
 
  586
 
 10
 
 11096
 
 gene;island
 
 0.152697
 
 0.9272125
 
 GJB6 (margin: GJB6)
 
  6.5247357
 
 1.08e-05
 
 4.82e-04
  
 
  
 3
 
 161089712
 
 161091175
 
  PDF   STATS   ENSEMBL   UCSC 
 
 1464
 
 15
 
 29638
 
 island
 
 0.487654
 
 0.0157366
 
 SPTSSB (margin: SPTSSB)
 
  6.2056414
 
 8.82e-06
 
 4.82e-04
  
 
  
 5
 
 139927007
 
 139927271
 
  PDF   STATS   ENSEMBL   UCSC 
 
  265
 
 10
 
 31785
 
 gene;island
 
 0.016379
 
 0.4741839
 
 ANKHD1; ANKHD1-EIF4EBP3; EIF4EBP3; SRA1 (margin: ANKHD1; ANKHD1-EIF4EBP3; EIF4EBP3; SRA1; APBB3)
 
  6.0731058
 
 1.08e-05
 
 4.82e-04
  
 
  
 19
 
  37064219
 
  37064886
 
  PDF   STATS   ENSEMBL   UCSC 
 
  668
 
 10
 
 20727
 
 tss
 
 0.014667
 
 0.4106843
 
 AC092295.7; ZNF529 (margin: AC092295.7; ZNF529)
 
  5.5658288
 
 1.08e-05
 
 4.82e-04
  
 
  
 14
 
  36003074
 
  36003528
 
  PDF   STATS   ENSEMBL   UCSC 
 
  455
 
 10
 
 12781
 
 island
 
 0.452536
 
 0.0138100
 
 INSM2 (margin: INSM2; RALGAPA1)
 
  5.5136322
 
 1.08e-05
 
 4.82e-04
  
 
  
 1
 
 115631930
 
 115632727
 
  PDF   STATS   ENSEMBL   UCSC 
 
  798
 
 10
 
 3628
 
 island
 
 0.015525
 
 0.5138926
 
 TSPAN2 (margin: RP4-666F24.3; TSPAN2)
 
  5.3242842
 
 1.08e-05
 
 4.82e-04
  
 
  
 14
 
 102026230
 
 102026555
 
  PDF   STATS   ENSEMBL   UCSC 
 
  326
 
 10
 
 12635
 
 tss
 
 0.517699
 
 0.9741354
 
 DIO3; DIO3OS (margin: DIO3; DIO3OS)
 
  5.0811134
 
 1.08e-05
 
 4.82e-04
  
 
  
 6
 
 134638742
 
 134639482
 
  PDF   STATS   ENSEMBL   UCSC 
 
  741
 
 10
 
 36495
 
 island
 
 0.425329
 
 0.9510374
 
 SGK1 (margin: SGK1)
 
  4.9670943
 
 1.08e-05
 
 4.82e-04
  
 
  
 5
 
 140624869
 
 140625389
 
  PDF   STATS   ENSEMBL   UCSC 
 
  521
 
 10
 
 33228
 
 island
 
 0.429667
 
 0.9586870
 
 PCDHB15 (margin: PCDHB18; PCDHB19P; PCDHB15)
 
  4.9074415
 
 1.08e-05
 
 4.82e-04
  
 
  
 4
 
 141489963
 
 141490428
 
  PDF   STATS   ENSEMBL   UCSC 
 
  466
 
 10
 
 30608
 
 tss
 
 0.624971
 
 0.9780354
 
 UCP1 (margin: RN7SL152P; UCP1)
 
  4.7542945
 
 1.08e-05
 
 4.82e-04
  
 
  
 15
 
  90039409
 
  90039913
 
  PDF   STATS   ENSEMBL   UCSC 
 
  505
 
 10
 
 14437
 
 island
 
 0.594427
 
 0.0561548
 
 RHCG (margin: RHCG; LINC00928)
 
  4.7024199
 
 1.08e-05
 
 4.82e-04
  
 
  
 2
 
 192015279
 
 192015918
 
  PDF   STATS   ENSEMBL   UCSC 
 
  640
 
 10
 
 22962
 
 gene
 
 0.294069
 
 0.0163572
 
 STAT4 (margin: STAT4)
 
  4.6282328
 
 1.08e-05
 
 4.82e-04
  
 
  
 11
 
  30607888
 
  30608198
 
  PDF   STATS   ENSEMBL   UCSC 
 
  311
 
 10
 
 8155
 
 island
 
 0.352776
 
 0.0194654
 
 RP5-1024C24.1; MPPED2 (margin: RP5-1024C24.1; MPPED2)
 
  4.6043398
 
 1.08e-05
 
 4.82e-04
  
 
  
 2
 
  25391656
 
  25391911
 
  PDF   STATS   ENSEMBL   UCSC 
 
  256
 
 10
 
 23231
 
 tss
 
 0.510038
 
 0.9699852
 
 POMC (margin: RP11-509E16.1; POMC)
 
  4.5965820
 
 1.08e-05
 
 4.82e-04
  
 
  
 16
 
  56622951
 
  56623549
 
  PDF   STATS   ENSEMBL   UCSC 
 
  599
 
 10
 
 15946
 
 island
 
 0.636101
 
 0.9839839
 
 MT3 (margin: MT3)
 
  4.5897708
 
 1.08e-05
 
 4.82e-04
  
 
  
 3
 
 134369339
 
 134370241
 
  PDF   STATS   ENSEMBL   UCSC 
 
  903
 
 13
 
 29500
 
 island
 
 0.415460
 
 0.8997911
 
 EPHB1; KY (margin: EPHB1; KY)
 
  4.5045837
 
 8.65e-06
 
 4.82e-04
  
 
  
 3
 
 173115252
 
 173116248
 
  PDF   STATS   ENSEMBL   UCSC 
 
  997
 
 13
 
 29671
 
 island
 
 0.545143
 
 0.9488451
 
 NLGN1 (margin: NLGN1)
 
  4.3833627
 
 8.65e-06
 
 4.82e-04
  
 
  
 5
 
 170736021
 
 170736572
 
  PDF   STATS   ENSEMBL   UCSC 
 
  552
 
 10
 
 33379
 
 island
 
 0.760427
 
 0.9810022
 
 TLX3 (margin: RANBP17; TLX3)
 
  4.3401126
 
 1.08e-05
 
 4.82e-04
  
 
  
 6
 
  30881112
 
  30881842
 
  PDF   STATS   ENSEMBL   UCSC 
 
  731
 
 23
 
 33835
 
 gene
 
 0.916063
 
 0.1066154
 
 GTF2H4; VARS2 (margin: RN7SL175P; GTF2H4; VARS2)
 
  4.3072001
 
 9.28e-06
 
 4.82e-04
  
 
  
 8
 
 144635260
 
 144635610
 
  PDF   STATS   ENSEMBL   UCSC 
 
  351
 
 10
 
 40668
 
 island
 
 0.546792
 
 0.9545885
 
 GSDMD (margin: 7SK; GSDMD; ZC3H3; RP11-661A12.5)
 
  4.1123408
 
 1.08e-05
 
 4.82e-04
  
 
  
 11
 
   8615356
 
   8616172
 
  PDF   STATS   ENSEMBL   UCSC 
 
  817
 
 16
 
 8033
 
 island
 
 0.817690
 
 0.9702997
 
 STK33 (margin: STK33)
 
  3.8905604
 
 8.94e-06
 
 4.82e-04
  
 
  
 6
 
  30923241
 
  30924004
 
  PDF   STATS   ENSEMBL   UCSC 
 
  764
 
 17
 
 35746
 
 island
 
 0.493822
 
 0.9093367
 
 DPCR1; HCG21 (margin: DPCR1; HCG21)
 
  3.8120138
 
 9.73e-06
 
 4.82e-04
  
 
  
 2
 
 177053274
 
 177053568
 
  PDF   STATS   ENSEMBL   UCSC 
 
  295
 
 10
 
 24915
 
 island
 
 0.074999
 
 0.4940114
 
 HOXD1; HOXD-AS1 (margin: AC009336.24; HOXD1; HOXD-AS1)
 
  3.6671171
 
 1.08e-05
 
 4.82e-04
  
 
  
 2
 
  65215361
 
  65215753
 
  PDF   STATS   ENSEMBL   UCSC 
 
  393
 
 10
 
 23393
 
 tss;island
 
 0.051784
 
 0.4305555
 
 SLC1A4 (margin: SLC1A4; RNU6-548P)
 
  3.6283989
 
 1.08e-05
 
 4.82e-04
  
 
  
 5
 
 178487123
 
 178487576
 
  PDF   STATS   ENSEMBL   UCSC 
 
  454
 
 10
 
 32548
 
 tss
 
 0.081366
 
 0.4853876
 
 ZNF354C (margin: ZNF354C)
 
  3.5974722
 
 1.08e-05
 
 4.82e-04
  
 
  
 20
 
  13200929
 
  13200992
 
  PDF   STATS   ENSEMBL   UCSC 
 
   64
 
 10
 
 25601
 
 tss
 
 0.841141
 
 0.9866767
 
 ISM1 (margin: ISM1)
 
  3.4801296
 
 1.08e-05
 
 4.82e-04
  
 
  
 5
 
 121414025
 
 121414425
 
  PDF   STATS   ENSEMBL   UCSC 
 
  401
 
 13
 
 33044
 
 island
 
 0.393905
 
 0.0773762
 
 LOX (margin: SRFBP1; LOX)
 
  3.4244587
 
 8.65e-06
 
 4.82e-04
  
 
  
 10
 
  82116203
 
  82116596
 
  PDF   STATS   ENSEMBL   UCSC 
 
  394
 
 10
 
 4654
 
 gene;tss;island
 
 0.856716
 
 0.9802598
 
 DYDC2; DYDC1 (margin: DYDC2; DYDC1)
 
  3.3994606
 
 1.08e-05
 
 4.82e-04
  
 
  
 7
 
  99517136
 
  99517509
 
  PDF   STATS   ENSEMBL   UCSC 
 
  374
 
 12
 
 38741
 
 island
 
 0.045404
 
 0.3374067
 
 TRIM4 (margin: RP4-604G5.1; TRIM4; GJC3)
 
  3.2165832
 
 8.88e-06
 
 4.82e-04
  
 
  
 8
 
  82192519
 
  82192931
 
  PDF   STATS   ENSEMBL   UCSC 
 
  413
 
 10
 
 40417
 
 island
 
 0.102944
 
 0.4334803
 
 FABP5; RP11-363E6.3 (margin: FABP5; RP11-363E6.4; RP11-363E6.3)
 
  2.9440294
 
 1.08e-05
 
 4.82e-04
  
 
  
 6
 
  32632565
 
  32633163
 
  PDF   STATS   ENSEMBL   UCSC 
 
  599
 
 22
 
 34126
 
 gene;island
 
 0.537709
 
 0.9360179
 
 HLA-DQB1 (margin: HLA-DQB1-AS1; HLA-DQB1; XXbac-BPG254F23.6)
 
  2.9004601
 
 1.03e-05
 
 4.82e-04
  
 
  
 15
 
  40544855
 
  40545511
 
  PDF   STATS   ENSEMBL   UCSC 
 
  657
 
 11
 
 13290
 
 gene;island
 
 0.794845
 
 0.9794130
 
 RP11-133K1.2; PAK6; C15orf56 (margin: RP11-133K1.2; PAK6; C15orf56)
 
  3.4832223
 
 1.13e-05
 
 5.02e-04
  
 
  
 7
 
   1250038
 
   1251125
 
  PDF   STATS   ENSEMBL   UCSC 
 
 1088
 
 17
 
 38089
 
 island
 
 0.112095
 
 0.7732560
 
  (margin: )
 
  4.6774887
 
 1.21e-05
 
 5.36e-04
  
 
  
 5
 
 134871462
 
 134872315
 
  PDF   STATS   ENSEMBL   UCSC 
 
  854
 
 21
 
 33138
 
 island
 
 0.641149
 
 0.9727417
 
 NEUROG1 (margin: NEUROG1)
 
  3.0226337
 
 1.23e-05
 
 5.38e-04
  
 
  
 6
 
  28984234
 
  28985069
 
  PDF   STATS   ENSEMBL   UCSC 
 
  836
 
 24
 
 35610
 
 island
 
 0.014891
 
 0.3252265
 
  (margin: ZNF311; OR2AD1P)
 
  4.5726378
 
 1.25e-05
 
 5.44e-04
  
 
  
 1
 
  39874802
 
  39875238
 
  PDF   STATS   ENSEMBL   UCSC 
 
  437
 
 13
 
 446
 
 gene;island
 
 0.749083
 
 0.0195854
 
 MACF1; KIAA0754 (margin: MACF1; KIAA0754)
 
  6.4618440
 
 1.29e-05
 
 5.58e-04
  
 
  
 17
 
  56832804
 
  56833425
 
  PDF   STATS   ENSEMBL   UCSC 
 
  622
 
 13
 
 18605
 
 island
 
 0.805132
 
 0.3756145
 
 PPM1E (margin: PPM1E)
 
  2.7354857
 
 1.29e-05
 
 5.58e-04
  
 
  
 11
 
   8615506
 
   8616172
 
  PDF   STATS   ENSEMBL   UCSC 
 
  667
 
 14
 
 7126
 
 tss
 
 0.802201
 
 0.9755193
 
 STK33 (margin: STK33)
 
  3.9481419
 
 1.36e-05
 
 5.85e-04
  
 
  
 12
 
   7282081
 
   7282931
 
  PDF   STATS   ENSEMBL   UCSC 
 
  851
 
 14
 
 9546
 
 tss
 
 0.661019
 
 0.0100009
 
 CLSTN3; RBP5; RP11-273B20.1 (margin: C1RL-AS1; RNU6-485P; CLSTN3; RBP5; RP11-273B20.1)
 
  7.9452698
 
 1.86e-05
 
 7.98e-04
  
 
  
 8
 
  38964784
 
  38965492
 
  PDF   STATS   ENSEMBL   UCSC 
 
  709
 
 11
 
 40239
 
 island
 
 0.987796
 
 0.0845960
 
 ADAM32 (margin: ADAM9; ADAM32)
 
  9.1104405
 
 1.98e-05
 
 8.44e-04
  
 
  
 11
 
  70507825
 
  70508659
 
  PDF   STATS   ENSEMBL   UCSC 
 
  835
 
 11
 
 6793
 
 gene;island
 
 0.620673
 
 0.9574276
 
 SHANK2 (margin: SHANK2)
 
  3.9912731
 
 1.98e-05
 
 8.44e-04
  
 
  
 13
 
  96296979
 
  96297338
 
  PDF   STATS   ENSEMBL   UCSC 
 
  360
 
 10
 
 11503
 
 tss
 
 0.033612
 
 0.9846144
 
 DZIP1 (margin: DZIP1; DNAJC3-AS1)
 
 10.8876312
 
 2.17e-05
 
 8.94e-04
  
 
  
 6
 
 150346721
 
 150347053
 
  PDF   STATS   ENSEMBL   UCSC 
 
  333
 
 10
 
 35294
 
 tss
 
 0.014398
 
 0.6850069
 
 RAET1L (margin: RAET1M; RAET1L)
 
  6.8704269
 
 2.17e-05
 
 8.94e-04
  
 
  
 6
 
  24911204
 
  24911615
 
  PDF   STATS   ENSEMBL   UCSC 
 
  412
 
 10
 
 34672
 
 tss
 
 0.662098
 
 0.0554352
 
 FAM65B (margin: FAM65B)
 
  5.9609205
 
 2.17e-05
 
 8.94e-04
  
 
  
 7
 
 112726396
 
 112726869
 
  PDF   STATS   ENSEMBL   UCSC 
 
  474
 
 10
 
 37191
 
 gene;tss
 
 0.365289
 
 0.9556943
 
 GPR85 (margin: GPR85)
 
  5.7893176
 
 2.17e-05
 
 8.94e-04
  
 
  
 12
 
  12502846
 
  12503544
 
  PDF   STATS   ENSEMBL   UCSC 
 
  699
 
 10
 
 10246
 
 island
 
 0.387336
 
 0.0192856
 
 MANSC1 (margin: RP11-757G14.1; LOH12CR1; MANSC1; LOH12CR2)
 
  5.1246918
 
 2.17e-05
 
 8.94e-04
  
 
  
 12
 
  72666976
 
  72667707
 
  PDF   STATS   ENSEMBL   UCSC 
 
  732
 
 10
 
 9218
 
 gene;island
 
 0.281286
 
 0.8547828
 
 TRHDE; TRHDE-AS1 (margin: TRHDE; TRHDE-AS1)
 
  3.9288612
 
 2.17e-05
 
 8.94e-04
  
 
  
 6
 
  32809263
 
  32809657
 
  PDF   STATS   ENSEMBL   UCSC 
 
  395
 
 10
 
 34148
 
 gene;island
 
 0.982997
 
 0.9511788
 
 PSMB8 (margin: XXbac-BPG246D15.8; PSMB9; TAP2; PSMB8; TAP1)
 
  1.4564412
 
 2.17e-05
 
 8.94e-04
  
 
  
 1
 
  39874802
 
  39875069
 
  PDF   STATS   ENSEMBL   UCSC 
 
  268
 
 12
 
 1521
 
 tss
 
 0.701414
 
 0.0195792
 
 MACF1; KIAA0754 (margin: MACF1; KIAA0754)
 
  6.7409221
 
 2.22e-05
 
 9.05e-04
  
 
  
 6
 
  46702683
 
  46703422
 
  PDF   STATS   ENSEMBL   UCSC 
 
  740
 
 12
 
 34360
 
 gene
 
 0.443560
 
 0.9666003
 
 PLA2G7 (margin: ANKRD66; PLA2G7)
 
  5.0675194
 
 2.22e-05
 
 9.05e-04
  
 
  
 17
 
   6679254
 
   6679781
 
  PDF   STATS   ENSEMBL   UCSC 
 
  528
 
 12
 
 17910
 
 island
 
 0.614425
 
 0.9769087
 
 XAF1; FBXO39 (margin: XAF1; FBXO39)
 
  4.4300689
 
 2.22e-05
 
 9.05e-04
  
 
  
 17
 
  46655164
 
  46656093
 
  PDF   STATS   ENSEMBL   UCSC 
 
  930
 
 18
 
 18480
 
 island
 
 0.861904
 
 0.9735898
 
 HOXB-AS3; HOXB3; HOXB4; MIR10A (margin: HOXB-AS3; HOXB3; HOXB4; MIR10A)
 
  1.8508593
 
 2.39e-05
 
 9.70e-04
  
 
  
 3
 
 161089871
 
 161091175
 
  PDF   STATS   ENSEMBL   UCSC 
 
 1305
 
 14
 
 28698
 
 tss
 
 0.529137
 
 0.0140962
 
 SPTSSB (margin: SPTSSB)
 
  6.5090714
 
 2.53e-05
 
 1.02e-03
  
 
  
 10
 
 124894943
 
 124895886
 
  PDF   STATS   ENSEMBL   UCSC 
 
  944
 
 13
 
 6215
 
 island
 
 0.540449
 
 0.9841326
 
 HMX3 (margin: HMX3; HMX2)
 
  5.8233934
 
 2.67e-05
 
 1.07e-03
  
 
  
 3
 
 125899370
 
 125900108
 
  PDF   STATS   ENSEMBL   UCSC 
 
  739
 
 13
 
 29427
 
 island
 
 0.771005
 
 0.9783004
 
 ALDH1L1-AS2; ALDH1L1 (margin: ALDH1L1-AS2; ALDH1L1)
 
  3.6570279
 
 2.67e-05
 
 1.07e-03
  
 
  
 11
 
  86383182
 
  86383940
 
  PDF   STATS   ENSEMBL   UCSC 
 
  759
 
 20
 
 8712
 
 island
 
 0.838721
 
 0.9635293
 
 ME3 (margin: ME3)
 
  2.1980145
 
 2.90e-05
 
 1.16e-03
  
 
  
 2
 
  29337946
 
  29338258
 
  PDF   STATS   ENSEMBL   UCSC 
 
  313
 
  9
 
 23282
 
 tss
 
 0.005635
 
 0.9888473
 
 CLIP4 (margin: CLIP4)
 
 13.3242456
 
 4.11e-05
 
 1.17e-03
  
 
  
 12
 
  51566731
 
  51567112
 
  PDF   STATS   ENSEMBL   UCSC 
 
  382
 
  9
 
 9712
 
 tss
 
 0.011744
 
 0.9776945
 
 TFCP2 (margin: RNU6-199P; TFCP2)
 
 12.6482367
 
 4.11e-05
 
 1.17e-03
  
 
  
 14
 
  75593812
 
  75594058
 
  PDF   STATS   ENSEMBL   UCSC 
 
  247
 
  9
 
 12516
 
 tss
 
 0.006636
 
 0.9826888
 
 RP11-950C14.7; NEK9 (margin: RP11-950C14.7; NEK9; HIF1AP1; TMED10)
 
 12.5167851
 
 4.11e-05
 
 1.17e-03
  
 
  
 19
 
   9649241
 
   9649542
 
  PDF   STATS   ENSEMBL   UCSC 
 
  302
 
  9
 
 21500
 
 island
 
 0.004427
 
 0.9659673
 
 ZNF426 (margin: ZNF426)
 
 12.3931158
 
 4.11e-05
 
 1.17e-03
  
 
  
 6
 
  30652202
 
  30652399
 
  PDF   STATS   ENSEMBL   UCSC 
 
  198
 
  9
 
 33802
 
 gene;island
 
 0.019670
 
 0.9822181
 
 PPP1R18 (margin: DHX16; PPP1R18; NRM)
 
 12.2577008
 
 4.11e-05
 
 1.17e-03
  
 
  
 1
 
 207224090
 
 207224740
 
  PDF   STATS   ENSEMBL   UCSC 
 
  651
 
  9
 
 4146
 
 island
 
 0.004897
 
 0.9547831
 
 PFKFB2; YOD1 (margin: PFKFB2; snoU13; YOD1)
 
 12.1164033
 
 4.11e-05
 
 1.17e-03
  
 
  
 22
 
  45608428
 
  45608713
 
  PDF   STATS   ENSEMBL   UCSC 
 
  286
 
  9
 
 27219
 
 tss
 
 0.011787
 
 0.9795075
 
 KIAA0930 (margin: KIAA0930; MIR1249)
 
 11.8454107
 
 4.11e-05
 
 1.17e-03
  
 
  
 3
 
  50378407
 
  50378664
 
  PDF   STATS   ENSEMBL   UCSC 
 
  258
 
  9
 
 28353
 
 tss
 
 0.010074
 
 0.9746159
 
 ZMYND10-AS1; RASSF1; ZMYND10 (margin: ZMYND10-AS1; CYB561D2; XXcos-LUCA11.5; RASSF1; ZMYND10; NPRL2)
 
 11.7341164
 
 4.11e-05
 
 1.17e-03
  
 
  
 6
 
  28367279
 
  28367898
 
  PDF   STATS   ENSEMBL   UCSC 
 
  620
 
  9
 
 35588
 
 island
 
 0.010310
 
 0.9401849
 
 ZSCAN12 (margin: RNU2-45P; ZSCAN12)
 
 11.3431464
 
 4.11e-05
 
 1.17e-03
  
 
  
 7
 
  27209195
 
  27209582
 
  PDF   STATS   ENSEMBL   UCSC 
 
  388
 
  9
 
 37497
 
 tss
 
 0.013288
 
 0.9777057
 
 HOXA-AS4; HOXA9; RP1-170O19.20; MIR196B; HOXA10 (margin: RP1-170O19.21; HOXA-AS4; HOXA7; HOXA9; RP1-170O19.20; MIR196B; HOXA10; HOXA11)
 
 11.1767334
 
 4.11e-05
 
 1.17e-03
  
 
  
 5
 
 140207460
 
 140208201
 
  PDF   STATS   ENSEMBL   UCSC 
 
  742
 
  9
 
 31797
 
 gene;island
 
 0.937397
 
 0.0137229
 
 PCDHA1; PCDHA2; PCDHA3; PCDHA4; PCDHA5; PCDHA6 (margin: PCDHA1; PCDHA2; PCDHA3; PCDHA4; PCDHA5; PCDHA6; PCDHA7)
 
 11.0161426
 
 4.11e-05
 
 1.17e-03
  
 
  
 7
 
  25219840
 
  25220162
 
  PDF   STATS   ENSEMBL   UCSC 
 
  323
 
  9
 
 37480
 
 tss
 
 0.011957
 
 0.9605954
 
 C7orf31 (margin: AC004129.7; C7orf31)
 
 10.8186454
 
 4.11e-05
 
 1.17e-03
  
 
  
 8
 
  98290148
 
  98290601
 
  PDF   STATS   ENSEMBL   UCSC 
 
  454
 
  9
 
 40476
 
 island
 
 0.964551
 
 0.0238116
 
 TSPYL5 (margin: TSPYL5)
 
 10.8014475
 
 4.11e-05
 
 1.17e-03
  
 
  
 13
 
  67804285
 
  67804744
 
  PDF   STATS   ENSEMBL   UCSC 
 
  460
 
  9
 
 11769
 
 island
 
 0.780391
 
 0.0048720
 
 PCDH9 (margin: PCDH9)
 
 10.7605078
 
 4.11e-05
 
 1.17e-03
  
 
  
 19
 
  17413767
 
  17414399
 
  PDF   STATS   ENSEMBL   UCSC 
 
  633
 
  9
 
 21734
 
 island
 
 0.014257
 
 0.9758769
 
 MRPL34; ABHD8 (margin: MRPL34; DDA1; ABHD8; CTD-2278I10.4)
 
 10.6142904
 
 4.11e-05
 
 1.17e-03
  
 
  
 8
 
 145024637
 
 145025178
 
  PDF   STATS   ENSEMBL   UCSC 
 
  542
 
  9
 
 39502
 
 gene;island
 
 0.018046
 
 0.9587871
 
 PLEC (margin: PLEC; MIR661)
 
 10.0831619
 
 4.11e-05
 
 1.17e-03
  
 
  
 13
 
  92050675
 
  92050843
 
  PDF   STATS   ENSEMBL   UCSC 
 
  169
 
  9
 
 11496
 
 tss
 
 0.586370
 
 0.0077824
 
 GPC5 (margin: GPC5)
 
 10.0027306
 
 4.11e-05
 
 1.17e-03
  
 
  
 12
 
  21810791
 
  21811034
 
  PDF   STATS   ENSEMBL   UCSC 
 
  244
 
  9
 
 9600
 
 tss
 
 0.018450
 
 0.9568697
 
 LDHB (margin: RP11-59N23.3; LDHB)
 
  9.9012810
 
 4.11e-05
 
 1.17e-03
  
 
  
 6
 
  28778091
 
  28778676
 
  PDF   STATS   ENSEMBL   UCSC 
 
  586
 
  9
 
 35595
 
 island
 
 0.951181
 
 0.0123133
 
  (margin: )
 
  9.8863541
 
 4.11e-05
 
 1.17e-03
  
 
  
 16
 
   4588414
 
   4589127
 
  PDF   STATS   ENSEMBL   UCSC 
 
  714
 
 11
 
 15671
 
 island
 
 0.019614
 
 0.9779079
 
 CDIP1 (margin: CDIP1)
 
  9.7222332
 
 3.40e-05
 
 1.17e-03
  
 
  
 7
 
 138720786
 
 138721065
 
  PDF   STATS   ENSEMBL   UCSC 
 
  280
 
  9
 
 37927
 
 tss;island
 
 0.030965
 
 0.9802515
 
 ZC3HAV1L (margin: ZC3HAV1L; ZC3HAV1)
 
  9.2728347
 
 4.11e-05
 
 1.17e-03
  
 
  
 5
 
  23507450
 
  23507656
 
  PDF   STATS   ENSEMBL   UCSC 
 
  207
 
  9
 
 32001
 
 tss
 
 0.846754
 
 0.0181593
 
 PRDM9 (margin: PRDM9)
 
  9.1312356
 
 4.11e-05
 
 1.17e-03
  
 
  
 6
 
 132128915
 
 132129103
 
  PDF   STATS   ENSEMBL   UCSC 
 
  189
 
  9
 
 35224
 
 tss;island
 
 0.608125
 
 0.0045050
 
 ENPP1 (margin: ENPP1; RP3-323K23.3)
 
  9.1127892
 
 4.11e-05
 
 1.17e-03
  
 
  
 4
 
 184826215
 
 184826479
 
  PDF   STATS   ENSEMBL   UCSC 
 
  265
 
  9
 
 30693
 
 tss;island
 
 0.874283
 
 0.0182415
 
 STOX2 (margin: STOX2)
 
  9.1025547
 
 4.11e-05
 
 1.17e-03
  
 
  
 2
 
  75788038
 
  75788552
 
  PDF   STATS   ENSEMBL   UCSC 
 
  515
 
  9
 
 22752
 
 gene;island
 
 0.133202
 
 0.9846943
 
 EVA1A (margin: EVA1A)
 
  9.0337909
 
 4.11e-05
 
 1.17e-03
  
 
  
 19
 
  16178091
 
  16178570
 
  PDF   STATS   ENSEMBL   UCSC 
 
  480
 
  9
 
 21712
 
 island
 
 0.004067
 
 0.6695707
 
 CTD-2231E14.4; TPM4 (margin: CTD-2231E14.4; TPM4)
 
  8.8282318
 
 4.11e-05
 
 1.17e-03
  
 
  
 12
 
 123215151
 
 123215684
 
  PDF   STATS   ENSEMBL   UCSC 
 
  534
 
  9
 
 10086
 
 tss
 
 0.945788
 
 0.0198039
 
 HCAR1 (margin: HCAR1; RP11-324E6.9)
 
  8.5020491
 
 4.11e-05
 
 1.17e-03
  
 
  
 4
 
 186456397
 
 186457012
 
  PDF   STATS   ENSEMBL   UCSC 
 
  616
 
  9
 
 31477
 
 island
 
 0.836308
 
 0.0093157
 
 PDLIM3 (margin: PDLIM3)
 
  8.4389742
 
 4.11e-05
 
 1.17e-03
  
 
  
 7
 
  94537584
 
  94537893
 
  PDF   STATS   ENSEMBL   UCSC 
 
  310
 
  9
 
 37094
 
 gene;island
 
 0.823709
 
 0.0110768
 
 PPP1R9A (margin: PPP1R9A)
 
  8.1983743
 
 4.11e-05
 
 1.17e-03
  
 
  
 13
 
  43566401
 
  43566642
 
  PDF   STATS   ENSEMBL   UCSC 
 
  242
 
  9
 
 11414
 
 tss
 
 0.085550
 
 0.9700302
 
 EPSTI1 (margin: EPSTI1)
 
  8.1701591
 
 4.11e-05
 
 1.17e-03
  
 
  
 8
 
  61193970
 
  61194292
 
  PDF   STATS   ENSEMBL   UCSC 
 
  323
 
  9
 
 39724
 
 tss
 
 0.823630
 
 0.0156382
 
 CA8 (margin: CA8)
 
  8.1002194
 
 4.11e-05
 
 1.17e-03
  
 
  
 11
 
  87908558
 
  87908938
 
  PDF   STATS   ENSEMBL   UCSC 
 
  381
 
  9
 
 8719
 
 island
 
 0.069801
 
 0.9644085
 
 MIR3166; RAB38 (margin: MIR3166; RAB38)
 
  8.0886652
 
 4.11e-05
 
 1.17e-03
  
 
  
 7
 
 138794401
 
 138794752
 
  PDF   STATS   ENSEMBL   UCSC 
 
  352
 
  9
 
 38949
 
 island
 
 0.005235
 
 0.6746008
 
 ZC3HAV1 (margin: ZC3HAV1)
 
  7.9031135
 
 4.11e-05
 
 1.17e-03
  
 
  
 12
 
  15373987
 
  15374609
 
  PDF   STATS   ENSEMBL   UCSC 
 
  623
 
  9
 
 10268
 
 island
 
 0.748996
 
 0.0122012
 
 RERG (margin: RERG)
 
  7.7787962
 
 4.11e-05
 
 1.17e-03
  
 
  
 14
 
  91580129
 
  91580613
 
  PDF   STATS   ENSEMBL   UCSC 
 
  485
 
  9
 
 12558
 
 tss
 
 0.034577
 
 0.9058400
 
 C14orf159 (margin: C14orf159)
 
  7.7418403
 
 4.11e-05
 
 1.17e-03
  
 
  
 2
 
  74730507
 
  74730948
 
  PDF   STATS   ENSEMBL   UCSC 
 
  442
 
  9
 
 23461
 
 tss
 
 0.024516
 
 0.5848811
 
 RP11-523H20.3; LBX2-AS1; LBX2; PCGF1 (margin: TTC31; RP11-523H20.3; LBX2-AS1; TLX2; LBX2; AC005041.17; PCGF1)
 
  7.5882654
 
 4.11e-05
 
 1.17e-03
  
 
  
 4
 
  44728525
 
  44728857
 
  PDF   STATS   ENSEMBL   UCSC 
 
  333
 
  9
 
 31018
 
 island
 
 0.005683
 
 0.5274100
 
 GNPDA2 (margin: GNPDA2)
 
  7.5789664
 
 4.11e-05
 
 1.17e-03
  
 
  
 19
 
  54372324
 
  54372713
 
  PDF   STATS   ENSEMBL   UCSC 
 
  390
 
  9
 
 21043
 
 tss
 
 0.617231
 
 0.0068056
 
 MYADM; AC008753.6 (margin: AC008440.10; MYADM; PRKCG; AC008753.6; AC008440.5)
 
  7.5731122
 
 4.11e-05
 
 1.17e-03
  
 
  
 11
 
  98891492
 
  98891820
 
  PDF   STATS   ENSEMBL   UCSC 
 
  329
 
  9
 
 7640
 
 tss;island
 
 0.807820
 
 0.0198226
 
 CNTN5 (margin: CNTN5)
 
  7.4976302
 
 4.11e-05
 
 1.17e-03
  
 
  
 6
 
  46097392
 
  46097784
 
  PDF   STATS   ENSEMBL   UCSC 
 
  393
 
  9
 
 36184
 
 island
 
 0.555134
 
 0.0049560
 
 ENPP4; RP1-8B1.4 (margin: ENPP4; RP1-8B1.4)
 
  7.4767017
 
 4.11e-05
 
 1.17e-03
  
 
  
 2
 
 214148856
 
 214149200
 
  PDF   STATS   ENSEMBL   UCSC 
 
  345
 
  9
 
 25064
 
 island
 
 0.252636
 
 0.9684672
 
 SPAG16; AC079610.2 (margin: SPAG16; RP11-105N14.2; AC079610.2)
 
  7.3751812
 
 4.11e-05
 
 1.17e-03
  
 
  
 11
 
  24518149
 
  24518448
 
  PDF   STATS   ENSEMBL   UCSC 
 
  300
 
  9
 
 7205
 
 tss;island
 
 0.678467
 
 0.0311192
 
 LUZP2 (margin: LUZP2)
 
  7.3712876
 
 4.11e-05
 
 1.17e-03
  
 
  
 14
 
 103989208
 
 103989789
 
  PDF   STATS   ENSEMBL   UCSC 
 
  582
 
  9
 
 12649
 
 tss;island
 
 0.074624
 
 0.9796697
 
 CKB; RP11-600F24.7 (margin: TRMT61A; CKB; RP11-600F24.7)
 
  7.2607653
 
 4.11e-05
 
 1.17e-03
  
 
  
 10
 
 134901199
 
 134901689
 
  PDF   STATS   ENSEMBL   UCSC 
 
  491
 
  9
 
 6332
 
 island
 
 0.866980
 
 0.0420193
 
 GPR123; RP13-439H18.4; AL445199.1 (margin: GPR123; RP13-439H18.4; AL445199.1)
 
  7.2181635
 
 4.11e-05
 
 1.17e-03
  
 
  
 4
 
 118006405
 
 118006832
 
  PDF   STATS   ENSEMBL   UCSC 
 
  428
 
  9
 
 31264
 
 island
 
 0.512939
 
 0.0079537
 
 TRAM1L1 (margin: TRAM1L1)
 
  7.1448933
 
 4.11e-05
 
 1.17e-03
  
 
  
 11
 
  62477052
 
  62477624
 
  PDF   STATS   ENSEMBL   UCSC 
 
  573
 
  9
 
 7361
 
 tss
 
 0.410492
 
 0.9880753
 
 GNG3; BSCL2; RP11-831H9.16 (margin: GNG3; BSCL2; RP11-831H9.16; HNRNPUL2)
 
  6.8843584
 
 4.11e-05
 
 1.17e-03
  
 
  
 14
 
  75535757
 
  75536561
 
  PDF   STATS   ENSEMBL   UCSC 
 
  805
 
  9
 
 13012
 
 island
 
 0.008778
 
 0.4980837
 
 ZC2HC1C; ACYP1 (margin: ZC2HC1C; ACYP1)
 
  6.8689267
 
 4.11e-05
 
 1.17e-03
  
 
  
 4
 
  57976431
 
  57976944
 
  PDF   STATS   ENSEMBL   UCSC 
 
  514
 
  9
 
 31076
 
 island
 
 0.013286
 
 0.6378675
 
 RP11-12A1.1; IGFBP7 (margin: RP11-12A1.1; IGFBP7; snoU13)
 
  6.8482152
 
 4.11e-05
 
 1.17e-03
  
 
  
 3
 
 132378605
 
 132379275
 
  PDF   STATS   ENSEMBL   UCSC 
 
  671
 
  9
 
 27950
 
 gene;tss;island
 
 0.005477
 
 0.4017249
 
 UBA5; ACAD11; NPHP3 (margin: HSPA8P19; UBA5; ACAD11; NPHP3)
 
  6.8396978
 
 4.11e-05
 
 1.17e-03
  
 
  
 10
 
 135340445
 
 135341025
 
  PDF   STATS   ENSEMBL   UCSC 
 
  581
 
  9
 
 6367
 
 island
 
 0.169381
 
 0.8924642
 
 CYP2E1; SPRN; AL161645.2 (margin: RP11-108K14.4; CYP2E1; SPRN; AL161645.2)
 
  6.7275304
 
 4.11e-05
 
 1.17e-03
  
 
  
 20
 
  44746392
 
  44746902
 
  PDF   STATS   ENSEMBL   UCSC 
 
  511
 
  9
 
 25789
 
 tss
 
 0.129910
 
 0.9344710
 
 CD40 (margin: CD40)
 
  6.7001442
 
 4.11e-05
 
 1.17e-03
  
 
  
 7
 
  37488564
 
  37489005
 
  PDF   STATS   ENSEMBL   UCSC 
 
  442
 
 11
 
 37541
 
 tss
 
 0.843908
 
 0.0528746
 
 ELMO1 (margin: ELMO1)
 
  6.6085811
 
 3.40e-05
 
 1.17e-03
  
 
  
 1
 
  23751134
 
  23751761
 
  PDF   STATS   ENSEMBL   UCSC 
 
  628
 
  9
 
 2835
 
 island
 
 0.700462
 
 0.0319162
 
 TCEA3 (margin: TCEA3; ASAP3)
 
  6.5050158
 
 4.11e-05
 
 1.17e-03
  
 
  
 6
 
  33160250
 
  33160604
 
  PDF   STATS   ENSEMBL   UCSC 
 
  355
 
  9
 
 34889
 
 tss
 
 0.282673
 
 0.9472364
 
 COL11A2; RXRB (margin: RNY4P10; SLC39A7; HSD17B8; COL11A2; RXRB)
 
  6.4509088
 
 4.11e-05
 
 1.17e-03
  
 
  
 5
 
 180230767
 
 180231194
 
  PDF   STATS   ENSEMBL   UCSC 
 
  428
 
  9
 
 31947
 
 gene;tss;island
 
 0.136342
 
 0.9728158
 
 MGAT1 (margin: MGAT1)
 
  6.4104153
 
 4.11e-05
 
 1.17e-03
  
 
  
 6
 
  42946962
 
  42947317
 
  PDF   STATS   ENSEMBL   UCSC 
 
  356
 
  9
 
 36146
 
 island
 
 0.014483
 
 0.6357615
 
 PEX6 (margin: PPP2R5D; PEX6)
 
  6.3896197
 
 4.11e-05
 
 1.17e-03
  
 
  
 2
 
  12856626
 
  12856970
 
  PDF   STATS   ENSEMBL   UCSC 
 
  345
 
  9
 
 23194
 
 tss
 
 0.180360
 
 0.0041553
 
 RP11-333O1.1; TRIB2 (margin: RP11-333O1.1; TRIB2)
 
  6.3754167
 
 4.11e-05
 
 1.17e-03
  
 
  
 17
 
   6899085
 
   6899380
 
  PDF   STATS   ENSEMBL   UCSC 
 
  296
 
  9
 
 17056
 
 tss
 
 0.325649
 
 0.9807883
 
 ALOX12; AC027763.2; RP11-589P10.7; RP11-589P10.5 (margin: ALOX12; AC027763.2; RP11-589P10.7; RP11-589P10.5)
 
  6.3648955
 
 4.11e-05
 
 1.17e-03
  
 
  
 7
 
  12609907
 
  12610196
 
  PDF   STATS   ENSEMBL   UCSC 
 
  290
 
  9
 
 37445
 
 tss
 
 0.228348
 
 0.0076617
 
 SCIN; AC005281.2 (margin: SCIN; AC005281.2)
 
  6.2754030
 
 4.11e-05
 
 1.17e-03
  
 
  
 4
 
  74965068
 
  74965278
 
  PDF   STATS   ENSEMBL   UCSC 
 
  211
 
  9
 
 30434
 
 tss
 
 0.011064
 
 0.3502603
 
 CXCL2 (margin: CXCL2)
 
  6.2686749
 
 4.11e-05
 
 1.17e-03
  
 
  
 2
 
 201450323
 
 201450690
 
  PDF   STATS   ENSEMBL   UCSC 
 
  368
 
  9
 
 23810
 
 tss
 
 0.457338
 
 0.9840320
 
 SGOL2; AOX1 (margin: SGOL2; AOX1)
 
  6.2013902
 
 4.11e-05
 
 1.17e-03
  
 
  
 5
 
  78985425
 
  78985592
 
  PDF   STATS   ENSEMBL   UCSC 
 
  168
 
  9
 
 32147
 
 tss
 
 0.544246
 
 0.0215677
 
 CMYA5 (margin: PAPD4; CMYA5)
 
  6.1219696
 
 4.11e-05
 
 1.17e-03
  
 
  
 1
 
  10056837
 
  10057312
 
  PDF   STATS   ENSEMBL   UCSC 
 
  476
 
  9
 
 2662
 
 island
 
 0.474104
 
 0.9820241
 
 RBP7 (margin: NMNAT1; RBP7)
 
  6.0782847
 
 4.11e-05
 
 1.17e-03
  
 
  
 2
 
 207139066
 
 207139471
 
  PDF   STATS   ENSEMBL   UCSC 
 
  406
 
  9
 
 23835
 
 tss;island
 
 0.010014
 
 0.4256223
 
 ZDBF2 (margin: ZDBF2)
 
  5.9419872
 
 4.11e-05
 
 1.17e-03
  
 
  
 4
 
  10459128
 
  10459435
 
  PDF   STATS   ENSEMBL   UCSC 
 
  308
 
  9
 
 30300
 
 tss;island
 
 0.209543
 
 0.9159577
 
 ZNF518B (margin: ZNF518B)
 
  5.9172831
 
 4.11e-05
 
 1.17e-03
  
 
  
 7
 
  93204046
 
  93204478
 
  PDF   STATS   ENSEMBL   UCSC 
 
  433
 
  9
 
 37713
 
 tss;island
 
 0.777357
 
 0.0751429
 
 CALCR (margin: CALCR)
 
  5.8747534
 
 4.11e-05
 
 1.17e-03
  
 
  
 1
 
 248020331
 
 248020812
 
  PDF   STATS   ENSEMBL   UCSC 
 
  482
 
  9
 
 4421
 
 island
 
 0.465363
 
 0.9764309
 
 TRIM58 (margin: TRIM58; OR2W3)
 
  5.8687225
 
 4.11e-05
 
 1.17e-03
  
 
  
 20
 
  30457834
 
  30458267
 
  PDF   STATS   ENSEMBL   UCSC 
 
  434
 
  9
 
 25384
 
 gene;tss;island
 
 0.522422
 
 0.0207159
 
 TTLL9; DUSP15 (margin: TTLL9; DUSP15)
 
  5.5859385
 
 4.11e-05
 
 1.17e-03
  
 
  
 8
 
  75896645
 
  75897310
 
  PDF   STATS   ENSEMBL   UCSC 
 
  666
 
  9
 
 40400
 
 island
 
 0.418945
 
 0.0133492
 
 CRISPLD1 (margin: CRISPLD1)
 
  5.4597035
 
 4.11e-05
 
 1.17e-03
  
 
  
 3
 
   9993731
 
   9994280
 
  PDF   STATS   ENSEMBL   UCSC 
 
  550
 
  9
 
 28868
 
 island
 
 0.381345
 
 0.0096619
 
 PRRT3-AS1; PRRT3 (margin: CRELD1; PRRT3-AS1; RP11-1020A11.2; PRRT3; EMC3)
 
  5.4542383
 
 4.11e-05
 
 1.17e-03
  
 
  
 2
 
  20866107
 
  20866500
 
  PDF   STATS   ENSEMBL   UCSC 
 
  394
 
  9
 
 24115
 
 island
 
 0.294031
 
 0.9835720
 
 GDF7 (margin: GDF7; AC012065.7; RP11-130L8.1)
 
  5.4413921
 
 4.11e-05
 
 1.17e-03
  
 
  
 6
 
 150311305
 
 150312368
 
  PDF   STATS   ENSEMBL   UCSC 
 
 1064
 
  9
 
 36564
 
 island
 
 0.184427
 
 0.9624256
 
  (margin: RP11-472G23.3; BTF3P10; RAET1K)
 
  5.2358471
 
 4.11e-05
 
 1.17e-03
  
 
  
 7
 
  94953653
 
  94954202
 
  PDF   STATS   ENSEMBL   UCSC 
 
  550
 
  9
 
 38677
 
 island
 
 0.491455
 
 0.9772265
 
 PON1 (margin: PON1)
 
  5.2214533
 
 4.11e-05
 
 1.17e-03
  
 
  
 10
 
 131354782
 
 131355302
 
  PDF   STATS   ENSEMBL   UCSC 
 
  521
 
  9
 
 4826
 
 gene
 
 0.712452
 
 0.9858174
 
 MGMT (margin: MGMT)
 
  5.1806811
 
 4.11e-05
 
 1.17e-03
  
 
  
 13
 
  27335082
 
  27335542
 
  PDF   STATS   ENSEMBL   UCSC 
 
  461
 
  9
 
 11356
 
 tss;island
 
 0.351129
 
 0.9552539
 
 GPR12 (margin: GPR12)
 
  5.1618483
 
 4.11e-05
 
 1.17e-03
  
 
  
 19
 
  52490101
 
  52490339
 
  PDF   STATS   ENSEMBL   UCSC 
 
  239
 
  9
 
 21002
 
 tss
 
 0.518917
 
 0.0140808
 
 ZNF350 (margin: HCCAT3; ZNF350; ZNF615)
 
  5.1105813
 
 4.11e-05
 
 1.17e-03
  
 
  
 2
 
 154335014
 
 154335386
 
  PDF   STATS   ENSEMBL   UCSC 
 
  373
 
  9
 
 24792
 
 island
 
 0.115355
 
 0.0110995
 
 AC012501.2; RPRM (margin: AC012501.2; RPRM)
 
  5.0737162
 
 4.11e-05
 
 1.17e-03
  
 
  
 7
 
 122526408
 
 122526940
 
  PDF   STATS   ENSEMBL   UCSC 
 
  533
 
  9
 
 38875
 
 island
 
 0.453150
 
 0.0208943
 
 CADPS2 (margin: CADPS2)
 
  5.0704432
 
 4.11e-05
 
 1.17e-03
  
 
  
 8
 
 144640306
 
 144640755
 
  PDF   STATS   ENSEMBL   UCSC 
 
  450
 
  9
 
 39483
 
 gene;island
 
 0.041254
 
 0.6025440
 
 GSDMD (margin: GSDMD; RP11-661A12.5; MROH6)
 
  5.0672224
 
 4.11e-05
 
 1.17e-03
  
 
  
 4
 
  40858965
 
  40859344
 
  PDF   STATS   ENSEMBL   UCSC 
 
  380
 
  9
 
 29990
 
 gene;island
 
 0.975364
 
 0.5986130
 
 APBB2 (margin: APBB2; snoU13)
 
  4.9656925
 
 4.11e-05
 
 1.17e-03
  
 
  
 13
 
  33589962
 
  33590495
 
  PDF   STATS   ENSEMBL   UCSC 
 
  534
 
 11
 
 11385
 
 tss;island
 
 0.485445
 
 0.9771101
 
 KL (margin: KL)
 
  4.9564747
 
 3.40e-05
 
 1.17e-03
  
 
  
 11
 
   2721207
 
   2722713
 
  PDF   STATS   ENSEMBL   UCSC 
 
 1507
 
 41
 
 6466
 
 gene;island
 
 0.467848
 
 0.0277332
 
 KCNQ1; KCNQ1OT1 (margin: KCNQ1; KCNQ1OT1)
 
  4.8320191
 
 3.24e-05
 
 1.17e-03
  
 
  
 3
 
 134369919
 
 134370241
 
  PDF   STATS   ENSEMBL   UCSC 
 
  323
 
  9
 
 28607
 
 tss
 
 0.274664
 
 0.8964001
 
 EPHB1; KY (margin: EPHB1; KY)
 
  4.7645897
 
 4.11e-05
 
 1.17e-03
  
 
  
 6
 
  43276478
 
  43276781
 
  PDF   STATS   ENSEMBL   UCSC 
 
  304
 
  9
 
 36161
 
 island
 
 0.419961
 
 0.9627229
 
 RP11-480N24.3; CRIP3; ZNF318 (margin: SLC22A7; RP11-480N24.3; CRIP3; ZNF318)
 
  4.7227317
 
 4.11e-05
 
 1.17e-03
  
 
  
 6
 
  24911001
 
  24911615
 
  PDF   STATS   ENSEMBL   UCSC 
 
  615
 
 12
 
 35497
 
 island
 
 0.522011
 
 0.0554352
 
 FAM65B (margin: FAM65B)
 
  4.6848182
 
 3.33e-05
 
 1.17e-03
  
 
  
 10
 
  95326033
 
  95326409
 
  PDF   STATS   ENSEMBL   UCSC 
 
  377
 
  9
 
 5238
 
 tss;island
 
 0.473957
 
 0.9649599
 
 FFAR4 (margin: FFAR4)
 
  4.4152762
 
 4.11e-05
 
 1.17e-03
  
 
  
 8
 
 144635260
 
 144635547
 
  PDF   STATS   ENSEMBL   UCSC 
 
  288
 
  9
 
 39950
 
 tss
 
 0.532168
 
 0.9708437
 
 GSDMD (margin: 7SK; GSDMD; ZC3H3; RP11-661A12.5)
 
  4.3556327
 
 4.11e-05
 
 1.17e-03
  
 
  
 11
 
  30607950
 
  30608198
 
  PDF   STATS   ENSEMBL   UCSC 
 
  249
 
  9
 
 7220
 
 tss
 
 0.343962
 
 0.0213749
 
 RP5-1024C24.1; MPPED2 (margin: RP5-1024C24.1; MPPED2)
 
  4.3504035
 
 4.11e-05
 
 1.17e-03
  
 
  
 3
 
 173115252
 
 173116238
 
  PDF   STATS   ENSEMBL   UCSC 
 
  987
 
 12
 
 28723
 
 tss
 
 0.547077
 
 0.9529222
 
 NLGN1 (margin: NLGN1)
 
  4.3008754
 
 3.33e-05
 
 1.17e-03
  
 
  
 11
 
   3663491
 
   3663842
 
  PDF   STATS   ENSEMBL   UCSC 
 
  352
 
  9
 
 7096
 
 tss
 
 0.320098
 
 0.0230179
 
 ART5 (margin: TRPC2; ART1; ART5)
 
  4.1858447
 
 4.11e-05
 
 1.17e-03
  
 
  
 14
 
  38068553
 
  38069206
 
  PDF   STATS   ENSEMBL   UCSC 
 
  654
 
  9
 
 12798
 
 island
 
 0.918635
 
 0.2868352
 
 TTC6; FOXA1 (margin: TTC6; FOXA1)
 
  4.1783811
 
 4.11e-05
 
 1.17e-03
  
 
  
 11
 
   2321770
 
   2322050
 
  PDF   STATS   ENSEMBL   UCSC 
 
  281
 
  9
 
 6453
 
 gene;tss
 
 0.599768
 
 0.9464693
 
 TSPAN32; C11orf21 (margin: TSPAN32; C11orf21)
 
  3.9349783
 
 4.11e-05
 
 1.17e-03
  
 
  
 10
 
  91295045
 
  91295421
 
  PDF   STATS   ENSEMBL   UCSC 
 
  377
 
  9
 
 5924
 
 island
 
 0.815173
 
 0.9863773
 
 SLC16A12 (margin: SLC16A12)
 
  3.6328215
 
 4.11e-05
 
 1.17e-03
  
 
  
 16
 
  66878426
 
  66878810
 
  PDF   STATS   ENSEMBL   UCSC 
 
  385
 
  9
 
 15286
 
 tss
 
 0.840890
 
 0.9815035
 
 CA7; NAE1; RP11-61A14.1 (margin: CA7; NAE1; RP11-61A14.1)
 
  3.5651164
 
 4.11e-05
 
 1.17e-03
  
 
  
 3
 
 136537607
 
 136537863
 
  PDF   STATS   ENSEMBL   UCSC 
 
  257
 
  9
 
 29508
 
 island
 
 0.735570
 
 0.9623064
 
 SLC35G2 (margin: SLC35G2; RP11-731C17.1)
 
  3.4436730
 
 4.11e-05
 
 1.17e-03
  
 
  
 5
 
 134871645
 
 134872315
 
  PDF   STATS   ENSEMBL   UCSC 
 
  671
 
 18
 
 32305
 
 tss
 
 0.681826
 
 0.9749165
 
 NEUROG1 (margin: NEUROG1)
 
  3.1641578
 
 3.44e-05
 
 1.17e-03
  
 
  
 12
 
  39299132
 
  39299844
 
  PDF   STATS   ENSEMBL   UCSC 
 
  713
 
  9
 
 10325
 
 island
 
 0.450830
 
 0.8970623
 
 RP11-396F22.1; CPNE8 (margin: RP11-396F22.1; CPNE8; AC067735.1)
 
  2.9509450
 
 4.11e-05
 
 1.17e-03
  
 
  
 6
 
  44186914
 
  44187674
 
  PDF   STATS   ENSEMBL   UCSC 
 
  761
 
 12
 
 36171
 
 island
 
 0.866913
 
 0.3605407
 
 RP1-302G2.5; SLC29A1 (margin: RP1-302G2.5; SLC29A1)
 
  2.9023101
 
 3.33e-05
 
 1.17e-03
  
 
  
 4
 
  76555772
 
  76556042
 
  PDF   STATS   ENSEMBL   UCSC 
 
  271
 
  9
 
 30439
 
 tss
 
 0.115174
 
 0.4533539
 
 CDKL2 (margin: CDKL2; G3BP2)
 
  2.6553084
 
 4.11e-05
 
 1.17e-03
  
 
  
 7
 
  27224092
 
  27225897
 
  PDF   STATS   ENSEMBL   UCSC 
 
 1806
 
 34
 
 37499
 
 tss;island
 
 0.809211
 
 0.9521657
 
 HOXA11-AS; HOXA11; RP1-170O19.14 (margin: HOXA11-AS; RP1-170O19.20; HOXA10; HOXA11; RP1-170O19.14; HOXA13)
 
  2.1096248
 
 3.93e-05
 
 1.17e-03
  
 
  
 11
 
  62476891
 
  62477624
 
  PDF   STATS   ENSEMBL   UCSC 
 
  734
 
 10
 
 8355
 
 island
 
 0.388633
 
 0.9867316
 
 GNG3; BSCL2; RP11-831H9.16 (margin: GNG3; BSCL2; RP11-831H9.16; HNRNPUL2)
 
  6.8336944
 
 4.33e-05
 
 1.22e-03
  
 
  
 6
 
  55443757
 
  55444488
 
  PDF   STATS   ENSEMBL   UCSC 
 
  732
 
 10
 
 36218
 
 island
 
 0.649865
 
 0.0377140
 
 HMGCLL1 (margin: HMGCLL1)
 
  6.3762181
 
 4.33e-05
 
 1.22e-03
  
 
  
 7
 
  27213893
 
  27214383
 
  PDF   STATS   ENSEMBL   UCSC 
 
  491
 
 10
 
 36939
 
 gene;island
 
 0.870379
 
 0.9879484
 
 RP1-170O19.20; HOXA10 (margin: HOXA-AS4; HOXA11-AS; HOXA9; RP1-170O19.20; MIR196B; HOXA10; HOXA11; RP1-170O19.14)
 
  4.4574483
 
 4.33e-05
 
 1.22e-03
  
 
  
 10
 
 131265059
 
 131265796
 
  PDF   STATS   ENSEMBL   UCSC 
 
  738
 
 15
 
 6258
 
 island
 
 0.406370
 
 0.0076123
 
 MGMT (margin: MGMT)
 
  5.5510671
 
 4.40e-05
 
 1.23e-03
  
 
  
 6
 
  33160067
 
  33160604
 
  PDF   STATS   ENSEMBL   UCSC 
 
  538
 
 15
 
 35969
 
 island
 
 0.471990
 
 0.9349884
 
 COL11A2; RXRB (margin: RNY4P10; SLC39A7; HSD17B8; COL11A2; RXRB)
 
  4.4745491
 
 4.40e-05
 
 1.23e-03
  
 
  
 6
 
  32811884
 
  32812708
 
  PDF   STATS   ENSEMBL   UCSC 
 
  825
 
 32
 
 34877
 
 tss
 
 0.007803
 
 0.0610363
 
 XXbac-BPG246D15.8; PSMB9; PSMB8; TAP1 (margin: XXbac-BPG246D15.8; PSMB9; TAP2; PSMB8; TAP1)
 
  2.1603276
 
 5.20e-05
 
 1.45e-03
  
 
  
 12
 
  85306323
 
  85307152
 
  PDF   STATS   ENSEMBL   UCSC 
 
  830
 
 13
 
 10656
 
 island
 
 0.357884
 
 0.9454598
 
 SLC6A15 (margin: SLC6A15)
 
  3.8717365
 
 5.23e-05
 
 1.45e-03
  
 
  
 20
 
  30639705
 
  30640256
 
  PDF   STATS   ENSEMBL   UCSC 
 
  552
 
 11
 
 26089
 
 island
 
 0.374674
 
 0.9459930
 
 RNA5SP482; HCK (margin: RNA5SP482; HCK)
 
  5.1154122
 
 5.39e-05
 
 1.47e-03
  
 
  
 11
 
    843897
 
    844686
 
  PDF   STATS   ENSEMBL   UCSC 
 
  790
 
 11
 
 6393
 
 gene;island
 
 0.450766
 
 0.0671055
 
 TSPAN4; POLR2L (margin: EFCAB4A; CD151; TSPAN4; AP006621.8; POLR2L)
 
  4.9715220
 
 5.39e-05
 
 1.47e-03
  
 
  
 3
 
 125899547
 
 125900108
 
  PDF   STATS   ENSEMBL   UCSC 
 
  562
 
 11
 
 28556
 
 tss
 
 0.771005
 
 0.9783004
 
 ALDH1L1-AS2; ALDH1L1 (margin: ALDH1L1-AS2; ALDH1L1)
 
  3.6570279
 
 5.39e-05
 
 1.47e-03
  
 
  
 5
 
 121414067
 
 121414425
 
  PDF   STATS   ENSEMBL   UCSC 
 
  359
 
 11
 
 32243
 
 tss
 
 0.408767
 
 0.0773762
 
 LOX (margin: SRFBP1; LOX)
 
  3.4244587
 
 5.39e-05
 
 1.47e-03
  
 
  
 1
 
   2344979
 
   2345475
 
  PDF   STATS   ENSEMBL   UCSC 
 
  497
 
 11
 
 1173
 
 tss;island
 
 0.151810
 
 0.0288236
 
 PEX10 (margin: RER1; PLCH2; PEX10)
 
  3.3797533
 
 5.39e-05
 
 1.47e-03
  
 
  
 15
 
  74218418
 
  74218921
 
  PDF   STATS   ENSEMBL   UCSC 
 
  504
 
 11
 
 14292
 
 island
 
 0.803470
 
 0.9465421
 
 LOXL1; LOXL1-AS1 (margin: LOXL1; LOXL1-AS1)
 
  2.1287304
 
 5.39e-05
 
 1.47e-03
  
 
  
 11
 
   2322286
 
   2323459
 
  PDF   STATS   ENSEMBL   UCSC 
 
 1174
 
 23
 
 6454
 
 gene;tss
 
 0.453420
 
 0.9043383
 
 TSPAN32; C11orf21 (margin: TSPAN32; C11orf21)
 
  3.0569059
 
 5.58e-05
 
 1.52e-03
  
 
  
 6
 
  29454557
 
  29455532
 
  PDF   STATS   ENSEMBL   UCSC 
 
  976
 
 15
 
 33659
 
 gene
 
 0.106273
 
 0.7456049
 
 MAS1L; RPS17P1 (margin: XXbac-BPG13B8.10; MAS1LP1; MAS1L; RPS17P1)
 
  4.5521319
 
 5.62e-05
 
 1.53e-03
  
 
  
 11
 
   2721243
 
   2722713
 
  PDF   STATS   ENSEMBL   UCSC 
 
 1471
 
 40
 
 7077
 
 tss
 
 0.477564
 
 0.0277398
 
 KCNQ1; KCNQ1OT1 (margin: KCNQ1; KCNQ1OT1)
 
  4.7463830
 
 5.98e-05
 
 1.62e-03
  
 
  
 11
 
  44331905
 
  44333192
 
  PDF   STATS   ENSEMBL   UCSC 
 
 1288
 
 40
 
 7261
 
 tss;island
 
 0.795993
 
 0.9611622
 
 ALX4 (margin: ALX4)
 
  1.8846424
 
 6.54e-05
 
 1.76e-03
  
 
  
 11
 
   3187271
 
   3188016
 
  PDF   STATS   ENSEMBL   UCSC 
 
  746
 
 24
 
 7092
 
 tss;island
 
 0.814123
 
 0.9342810
 
 OSBPL5 (margin: OSBPL5)
 
  2.0254009
 
 7.11e-05
 
 1.91e-03
  
 
  
 6
 
 117197952
 
 117198743
 
  PDF   STATS   ENSEMBL   UCSC 
 
  792
 
 12
 
 36434
 
 island
 
 0.496516
 
 0.0425366
 
 RFX6 (margin: RFX6)
 
  4.5413402
 
 7.17e-05
 
 1.92e-03
  
 
  
 2
 
 175199259
 
 175199828
 
  PDF   STATS   ENSEMBL   UCSC 
 
  570
 
 12
 
 24874
 
 island
 
 0.601333
 
 0.9768278
 
 SP9; AC018470.1 (margin: AC018470.4; SP9; AC018470.1)
 
  3.8768679
 
 7.17e-05
 
 1.92e-03
  
 
  
 1
 
 248100183
 
 248100614
 
  PDF   STATS   ENSEMBL   UCSC 
 
  432
 
 10
 
 4422
 
 island
 
 0.757900
 
 0.0185018
 
 OR2L13; OR2X1P; RP11-438H8.8 (margin: OR2AJ1; OR2L13; OR2X1P; OR2L8; RP11-438H8.8)
 
  7.7816397
 
 7.58e-05
 
 1.98e-03
  
 
  
 1
 
   6085799
 
   6086652
 
  PDF   STATS   ENSEMBL   UCSC 
 
  854
 
 10
 
 2589
 
 island
 
 0.049736
 
 0.5472285
 
 KCNAB2 (margin: KCNAB2)
 
  4.3178895
 
 7.58e-05
 
 1.98e-03
  
 
  
 5
 
 140305947
 
 140306458
 
  PDF   STATS   ENSEMBL   UCSC 
 
  512
 
 10
 
 31804
 
 gene;island
 
 0.957368
 
 0.5086992
 
 PCDHA1; PCDHA2; PCDHA3; PCDHA4; PCDHA5; PCDHA6; PCDHA7; PCDHA8; PCDHA9; PCDHA10; PCDHA11; PCDHA12; PCDHA13; PCDHAC1 (margin: PCDHA1; PCDHA2; PCDHA3; PCDHA4; PCDHA5; PCDHA6; PCDHA7; PCDHA8; PCDHA9; PCDHA10; PCDHA11; PCDHA12; PCDHA13; PCDHAC1)
 
  4.1644448
 
 7.58e-05
 
 1.98e-03
  
 
  
 11
 
  70508022
 
  70508659
 
  PDF   STATS   ENSEMBL   UCSC 
 
  638
 
 10
 
 7529
 
 tss
 
 0.600272
 
 0.9493446
 
 SHANK2 (margin: SHANK2)
 
  3.9998701
 
 7.58e-05
 
 1.98e-03
  
 
  
 12
 
  15475116
 
  15475767
 
  PDF   STATS   ENSEMBL   UCSC 
 
  652
 
 10
 
 10269
 
 island
 
 0.202319
 
 0.8044111
 
 PTPRO; RERG (margin: PTPRO; RERG)
 
  3.9134728
 
 7.58e-05
 
 1.98e-03
  
 
  
 17
 
  72931569
 
  72931857
 
  PDF   STATS   ENSEMBL   UCSC 
 
  289
 
 10
 
 17637
 
 tss
 
 0.514874
 
 0.8594905
 
 OTOP2; OTOP3 (margin: OTOP2; OTOP3)
 
  2.9007203
 
 7.58e-05
 
 1.98e-03
  
 
  
 1
 
 179711859
 
 179712361
 
  PDF   STATS   ENSEMBL   UCSC 
 
  503
 
 10
 
 4001
 
 island
 
 0.775828
 
 0.9617440
 
 RP11-12M5.1; FAM163A (margin: RP11-12M5.1; FAM163A)
 
  2.8807662
 
 7.58e-05
 
 1.98e-03
  
 
  
 1
 
  40782713
 
  40783264
 
  PDF   STATS   ENSEMBL   UCSC 
 
  552
 
 10
 
 3120
 
 island
 
 0.896823
 
 0.9781321
 
 COL9A2 (margin: COL9A2)
 
  2.5148239
 
 7.58e-05
 
 1.98e-03
  
 
  
 2
 
 219857793
 
 219858271
 
  PDF   STATS   ENSEMBL   UCSC 
 
  479
 
  9
 
 25100
 
 island
 
 0.253318
 
 0.9745469
 
 CRYBA2 (margin: AC097468.4; FEV; CRYBA2; MIR375; CCDC108)
 
  7.8995244
 
 8.23e-05
 
 2.06e-03
  
 
  
 4
 
   5712581
 
   5713100
 
  PDF   STATS   ENSEMBL   UCSC 
 
  520
 
  9
 
 30880
 
 island
 
 0.022459
 
 0.6702925
 
 EVC; EVC2 (margin: EVC; EVC2)
 
  6.9358204
 
 8.23e-05
 
 2.06e-03
  
 
  
 3
 
 181444368
 
 181445156
 
  PDF   STATS   ENSEMBL   UCSC 
 
  789
 
  9
 
 28045
 
 gene;island
 
 0.169176
 
 0.9710308
 
 SOX2-OT (margin: SOX2-OT)
 
  6.8280971
 
 8.23e-05
 
 2.06e-03
  
 
  
 3
 
  44770851
 
  44771113
 
  PDF   STATS   ENSEMBL   UCSC 
 
  263
 
  9
 
 28261
 
 tss
 
 0.321675
 
 0.9767697
 
 ZNF501 (margin: ZNF502; ZNF501; KIAA1143)
 
  6.4283335
 
 8.23e-05
 
 2.06e-03
  
 
  
 16
 
  68482518
 
  68483194
 
  PDF   STATS   ENSEMBL   UCSC 
 
  677
 
  9
 
 15322
 
 tss;island
 
 0.870023
 
 0.0396476
 
 SMPD3 (margin: SMPD3)
 
  6.0897315
 
 8.23e-05
 
 2.06e-03
  
 
  
 17
 
  16256937
 
  16257223
 
  PDF   STATS   ENSEMBL   UCSC 
 
  287
 
  9
 
 17156
 
 tss;island
 
 0.171990
 
 0.9367373
 
 CENPV (margin: PIGL; CENPV)
 
  5.5451189
 
 8.23e-05
 
 2.06e-03
  
 
  
 11
 
  69468789
 
  69469403
 
  PDF   STATS   ENSEMBL   UCSC 
 
  615
 
  9
 
 8593
 
 island
 
 0.895978
 
 0.3033873
 
 CCND1; ORAOV1 (margin: CCND1; ORAOV1)
 
  5.5100665
 
 8.23e-05
 
 2.06e-03
  
 
  
 2
 
 154333481
 
 154334077
 
  PDF   STATS   ENSEMBL   UCSC 
 
  597
 
  9
 
 24791
 
 island
 
 0.245991
 
 0.0051846
 
 AC012501.2; RPRM (margin: AC012501.2; RPRM)
 
  5.2620957
 
 8.23e-05
 
 2.06e-03
  
 
  
 19
 
  53662261
 
  53662723
 
  PDF   STATS   ENSEMBL   UCSC 
 
  463
 
  9
 
 22369
 
 island
 
 0.892812
 
 0.2818672
 
 ZNF347; ZNF665 (margin: CTD-2245F17.2; ZNF347; ZNF665)
 
  5.1989134
 
 8.23e-05
 
 2.06e-03
  
 
  
 20
 
  50418952
 
  50419348
 
  PDF   STATS   ENSEMBL   UCSC 
 
  397
 
  9
 
 26257
 
 island
 
 0.983176
 
 0.5604129
 
 SALL4 (margin: SALL4)
 
  5.0569645
 
 8.23e-05
 
 2.06e-03
  
 
  
 16
 
    215410
 
    216244
 
  PDF   STATS   ENSEMBL   UCSC 
 
  835
 
  9
 
 15443
 
 island
 
 0.673505
 
 0.9813736
 
 HBM; HBZP1 (margin: HBZ; HBM; HBZP1; Z84721.4; HBA2; HBA1)
 
  4.9381068
 
 8.23e-05
 
 2.06e-03
  
 
  
 5
 
 135416331
 
 135416613
 
  PDF   STATS   ENSEMBL   UCSC 
 
  283
 
  9
 
 32310
 
 tss
 
 0.536402
 
 0.9546425
 
 VTRNA2-1 (margin: VTRNA2-1)
 
  4.2757586
 
 8.23e-05
 
 2.06e-03
  
 
  
 7
 
  87229552
 
  87230344
 
  PDF   STATS   ENSEMBL   UCSC 
 
  793
 
  9
 
 37072
 
 gene;island
 
 0.376842
 
 0.9223944
 
 ABCB1 (margin: ABCB1)
 
  4.1556194
 
 8.23e-05
 
 2.06e-03
  
 
  
 21
 
  42688468
 
  42689051
 
  PDF   STATS   ENSEMBL   UCSC 
 
  584
 
  9
 
 26687
 
 island
 
 0.839102
 
 0.9869973
 
 FAM3B (margin: FAM3B)
 
  3.8379972
 
 8.23e-05
 
 2.06e-03
  
 
  
 8
 
  11561283
 
  11561620
 
  PDF   STATS   ENSEMBL   UCSC 
 
  338
 
  9
 
 39553
 
 tss
 
 0.759037
 
 0.9561329
 
 GATA4 (margin: GATA4)
 
  3.4905240
 
 8.23e-05
 
 2.06e-03
  
 
  
 14
 
  96180576
 
  96181044
 
  PDF   STATS   ENSEMBL   UCSC 
 
  469
 
  9
 
 12590
 
 tss
 
 0.728216
 
 0.9771808
 
 RP11-164H13.1; TCL1A (margin: RP11-164H13.1; TCL1A)
 
  2.8800449
 
 8.23e-05
 
 2.06e-03
  
 
  
 3
 
  68981852
 
  68982098
 
  PDF   STATS   ENSEMBL   UCSC 
 
  247
 
  9
 
 28437
 
 tss;island
 
 0.752155
 
 0.3924318
 
 FAM19A4 (margin: FAM19A4)
 
  2.7885604
 
 8.23e-05
 
 2.06e-03
  
 
  
 2
 
 219157083
 
 219157702
 
  PDF   STATS   ENSEMBL   UCSC 
 
  620
 
 11
 
 23014
 
 gene;island
 
 0.010717
 
 0.9429613
 
 PNKD; TMBIM1 (margin: PNKD; TMBIM1)
 
  9.0312299
 
 8.51e-05
 
 2.07e-03
  
 
  
 6
 
 146285286
 
 146285836
 
  PDF   STATS   ENSEMBL   UCSC 
 
  551
 
 11
 
 35274
 
 tss;island
 
 0.003789
 
 0.4024352
 
 SHPRH (margin: SHPRH)
 
  8.0283383
 
 8.51e-05
 
 2.07e-03
  
 
  
 2
 
 189156874
 
 189157566
 
  PDF   STATS   ENSEMBL   UCSC 
 
  693
 
 11
 
 24953
 
 island
 
 0.009169
 
 0.4411123
 
 GULP1 (margin: GULP1; MIR561; AC068718.1)
 
  6.2561123
 
 8.51e-05
 
 2.07e-03
  
 
  
 8
 
  63997977
 
  63998778
 
  PDF   STATS   ENSEMBL   UCSC 
 
  802
 
 11
 
 40337
 
 island
 
 0.447284
 
 0.0223178
 
 TTPA (margin: TTPA)
 
  6.2131459
 
 8.51e-05
 
 2.07e-03
  
 
  
 1
 
  19615303
 
  19615702
 
  PDF   STATS   ENSEMBL   UCSC 
 
  400
 
 11
 
 1302
 
 tss;island
 
 0.609638
 
 0.9773487
 
 AKR7A3 (margin: RP4-657E11.10; AKR7A3)
 
  5.1697243
 
 8.51e-05
 
 2.07e-03
  
 
  
 7
 
  27186993
 
  27187691
 
  PDF   STATS   ENSEMBL   UCSC 
 
  699
 
 11
 
 38349
 
 island
 
 0.454791
 
 0.9753813
 
 HOXA-AS3; HOXA3; HOXA6 (margin: HOXA-AS3; RP1-170O19.21; HOXA3; HOXA5; HOXA6; HOXA7)
 
  5.0409282
 
 8.51e-05
 
 2.07e-03
  
 
  
 17
 
  72889323
 
  72890001
 
  PDF   STATS   ENSEMBL   UCSC 
 
  679
 
 11
 
 18728
 
 island
 
 0.513969
 
 0.9530317
 
 FADS6 (margin: FADS6)
 
  4.6414284
 
 8.51e-05
 
 2.07e-03
  
 
  
 4
 
 174451141
 
 174451564
 
  PDF   STATS   ENSEMBL   UCSC 
 
  424
 
 11
 
 30682
 
 tss;island
 
 0.432426
 
 0.9544921
 
 HAND2-AS1; HAND2 (margin: HAND2-AS1; HAND2)
 
  3.8922235
 
 8.51e-05
 
 2.07e-03
  
 
  
 20
 
   2781122
 
   2781685
 
  PDF   STATS   ENSEMBL   UCSC 
 
  564
 
 11
 
 25954
 
 island
 
 0.740995
 
 0.9763788
 
 CPXM1 (margin: CPXM1)
 
  3.6853836
 
 8.51e-05
 
 2.07e-03
  
 
  
 7
 
   1022471
 
   1023156
 
  PDF   STATS   ENSEMBL   UCSC 
 
  686
 
 11
 
 38063
 
 island
 
 0.527375
 
 0.9551952
 
 CYP2W1 (margin: CYP2W1; COX19)
 
  3.6684859
 
 8.51e-05
 
 2.07e-03
  
 
  
 8
 
  11561283
 
  11561728
 
  PDF   STATS   ENSEMBL   UCSC 
 
  446
 
 11
 
 40087
 
 island
 
 0.781308
 
 0.9731732
 
 GATA4 (margin: GATA4)
 
  3.4905240
 
 8.51e-05
 
 2.07e-03
  
 
  
 17
 
  56832804
 
  56833201
 
  PDF   STATS   ENSEMBL   UCSC 
 
  398
 
 11
 
 17540
 
 tss
 
 0.807239
 
 0.3839881
 
 PPM1E (margin: PPM1E)
 
  2.7354857
 
 8.51e-05
 
 2.07e-03
  
 
  
 6
 
  46703193
 
  46703670
 
  PDF   STATS   ENSEMBL   UCSC 
 
  478
 
 15
 
 35022
 
 tss
 
 0.463165
 
 0.9300780
 
 PLA2G7 (margin: ANKRD66; PLA2G7)
 
  2.9459765
 
 8.98e-05
 
 2.18e-03
  
 
  
 1
 
 165204745
 
 165205506
 
  PDF   STATS   ENSEMBL   UCSC 
 
  762
 
 12
 
 863
 
 gene;island
 
 0.558146
 
 0.9569893
 
 LMX1A (margin: LMX1A)
 
  4.0562569
 
 1.03e-04
 
 2.47e-03
  
 
  
 7
 
  27143046
 
  27143806
 
  PDF   STATS   ENSEMBL   UCSC 
 
  761
 
 12
 
 37488
 
 tss;island
 
 0.959201
 
 0.6129512
 
 HOXA2; HOXA3 (margin: HOTAIRM1; HOXA-AS2; HOXA1; HOXA2; HOXA3)
 
  3.7404767
 
 1.03e-04
 
 2.47e-03
  
 
  
 2
 
 131512651
 
 131513216
 
  PDF   STATS   ENSEMBL   UCSC 
 
  566
 
 12
 
 23629
 
 tss;island
 
 0.582384
 
 0.9128807
 
 AMER3; AC140481.8 (margin: AMER3; AC140481.8)
 
  3.0413608
 
 1.03e-04
 
 2.47e-03
  
 
  
 17
 
  48911983
 
  48912556
 
  PDF   STATS   ENSEMBL   UCSC 
 
  574
 
 12
 
 17508
 
 tss
 
 0.843804
 
 0.9541893
 
 WFIKKN2 (margin: WFIKKN2; RP11-506D12.5)
 
  2.3429710
 
 1.03e-04
 
 2.47e-03
  
 
  
 6
 
  30068471
 
  30069319
 
  PDF   STATS   ENSEMBL   UCSC 
 
  849
 
 18
 
 35659
 
 island
 
 0.005996
 
 0.0552569
 
 TRIM31 (margin: TRIM31-AS1; AL669914.1; TRIM31)
 
  3.5029597
 
 1.13e-04
 
 2.70e-03
  
 
  
 19
 
  52074293
 
  52074501
 
  PDF   STATS   ENSEMBL   UCSC 
 
  209
 
  8
 
 20998
 
 tss;island
 
 0.005935
 
 0.9847649
 
 ZNF175 (margin: ZNF175; SIGLEC28P; SIGLEC29P)
 
 14.3777514
 
 1.55e-04
 
 2.71e-03
  
 
  
 22
 
  21368291
 
  21368765
 
  PDF   STATS   ENSEMBL   UCSC 
 
  475
 
  8
 
 26989
 
 tss;island
 
 0.003739
 
 0.9793876
 
 P2RX6; TUBA3FP (margin: THAP7-AS1; P2RX6; THAP7; TUBA3FP; AC002472.1)
 
 14.2853892
 
 1.55e-04
 
 2.71e-03
  
 
  
 12
 
  89744471
 
  89744877
 
  PDF   STATS   ENSEMBL   UCSC 
 
  407
 
  8
 
 9238
 
 gene;island
 
 0.004694
 
 0.9679356
 
 DUSP6 (margin: DUSP6)
 
 13.6886773
 
 1.55e-04
 
 2.71e-03
  
 
  
 1
 
  77332991
 
  77333229
 
  PDF   STATS   ENSEMBL   UCSC 
 
  239
 
  8
 
 3393
 
 island
 
 0.980715
 
 0.0086347
 
 ST6GALNAC5 (margin: ST6GALNAC5)
 
 12.8543572
 
 1.55e-04
 
 2.71e-03
  
 
  
 4
 
 165877875
 
 165878317
 
  PDF   STATS   ENSEMBL   UCSC 
 
  443
 
  8
 
 30171
 
 gene;island
 
 0.982038
 
 0.0201901
 
 RP11-366M4.8; FAM218A; TRIM61 (margin: RP11-366M4.8; FAM218A; TRIM61; RP11-366M4.11)
 
 12.6073618
 
 1.55e-04
 
 2.71e-03
  
 
  
 18
 
  44526430
 
  44527026
 
  PDF   STATS   ENSEMBL   UCSC 
 
  597
 
  8
 
 19493
 
 island
 
 0.960910
 
 0.0031573
 
 KATNAL2 (margin: KATNAL2)
 
 12.4532561
 
 1.55e-04
 
 2.71e-03
  
 
  
 19
 
  37997675
 
  37997831
 
  PDF   STATS   ENSEMBL   UCSC 
 
  157
 
  8
 
 20740
 
 tss
 
 0.899233
 
 0.0020897
 
 ZNF793; CTD-3064H18.1 (margin: ZNF793; CTD-3064H18.1; CTD-3064H18.2)
 
 11.6022125
 
 1.55e-04
 
 2.71e-03
  
 
  
 11
 
   6341717
 
   6341908
 
  PDF   STATS   ENSEMBL   UCSC 
 
  192
 
  8
 
 8013
 
 island
 
 0.018490
 
 0.9804994
 
 PRKCDBP (margin: PRKCDBP)
 
 11.5770794
 
 1.55e-04
 
 2.71e-03
  
 
  
 21
 
  34398085
 
  34398265
 
  PDF   STATS   ENSEMBL   UCSC 
 
  181
 
  8
 
 26642
 
 island
 
 0.938258
 
 0.0052505
 
 OLIG2; AP000282.2 (margin: OLIG2; AP000282.2)
 
 11.3344907
 
 1.55e-04
 
 2.71e-03
  
 
  
 4
 
 163085235
 
 163085411
 
  PDF   STATS   ENSEMBL   UCSC 
 
  177
 
  8
 
 30658
 
 tss
 
 0.954803
 
 0.0069198
 
 FSTL5 (margin: FSTL5)
 
 11.2389812
 
 1.55e-04
 
 2.71e-03
  
 
  
 12
 
  49393027
 
  49393408
 
  PDF   STATS   ENSEMBL   UCSC 
 
  382
 
  8
 
 10381
 
 island
 
 0.021250
 
 0.9084416
 
 RP11-386G11.5; DDN (margin: RP11-386G11.3; RP11-386G11.5; DDN; PRKAG1)
 
 11.0753583
 
 1.55e-04
 
 2.71e-03
  
 
  
 19
 
   1132256
 
   1132537
 
  PDF   STATS   ENSEMBL   UCSC 
 
  282
 
  8
 
 19644
 
 gene
 
 0.019989
 
 0.9854085
 
 SBNO2 (margin: SBNO2)
 
 10.8821625
 
 1.55e-04
 
 2.71e-03
  
 
  
 3
 
  49395574
 
  49395991
 
  PDF   STATS   ENSEMBL   UCSC 
 
  418
 
  8
 
 29116
 
 island
 
 0.012079
 
 0.9711581
 
 GPX1; RHOA (margin: GPX1; RHOA; RHOA-IT1)
 
 10.8723243
 
 1.55e-04
 
 2.71e-03
  
 
  
 3
 
  48632625
 
  48633144
 
  PDF   STATS   ENSEMBL   UCSC 
 
  520
 
  8
 
 28301
 
 tss
 
 0.011260
 
 0.9623150
 
 COL7A1 (margin: COL7A1; UQCRC1)
 
 10.8112114
 
 1.55e-04
 
 2.71e-03
  
 
  
 11
 
  22851321
 
  22851591
 
  PDF   STATS   ENSEMBL   UCSC 
 
  271
 
  8
 
 8137
 
 island
 
 0.924659
 
 0.0093297
 
 RP11-17A1.3; SVIP (margin: RP11-17A1.3; SVIP)
 
 10.2290087
 
 1.55e-04
 
 2.71e-03
  
 
  
 2
 
 202122309
 
 202122832
 
  PDF   STATS   ENSEMBL   UCSC 
 
  524
 
  8
 
 22988
 
 gene
 
 0.002760
 
 0.8755228
 
 CASP8 (margin: CASP8)
 
 10.1611439
 
 1.55e-04
 
 2.71e-03
  
 
  
 1
 
  51810337
 
  51811185
 
  PDF   STATS   ENSEMBL   UCSC 
 
  849
 
  8
 
 3261
 
 island
 
 0.029659
 
 0.9758068
 
 TTC39A (margin: RP11-275F13.1; TTC39A; EPS15)
 
  9.9683773
 
 1.55e-04
 
 2.71e-03
  
 
  
 17
 
  37123669
 
  37123949
 
  PDF   STATS   ENSEMBL   UCSC 
 
  281
 
  8
 
 17302
 
 tss
 
 0.913609
 
 0.0178865
 
 FBXO47 (margin: FBXO47)
 
  9.8417899
 
 1.55e-04
 
 2.71e-03
  
 
  
 8
 
  98290229
 
  98290601
 
  PDF   STATS   ENSEMBL   UCSC 
 
  373
 
  8
 
 39829
 
 tss
 
 0.961593
 
 0.0257387
 
 TSPYL5 (margin: TSPYL5)
 
  9.8272492
 
 1.55e-04
 
 2.71e-03
  
 
  
 1
 
  50489654
 
  50489954
 
  PDF   STATS   ENSEMBL   UCSC 
 
  301
 
  8
 
 1613
 
 tss
 
 0.835859
 
 0.0224351
 
 AGBL4 (margin: MTND2P29; AGBL4)
 
  9.5971456
 
 1.55e-04
 
 2.71e-03
  
 
  
 13
 
  29293196
 
  29293454
 
  PDF   STATS   ENSEMBL   UCSC 
 
  259
 
  8
 
 11370
 
 tss
 
 0.925900
 
 0.0174197
 
 SLC46A3 (margin: SLC46A3; CYP51P2)
 
  9.4247808
 
 1.55e-04
 
 2.71e-03
  
 
  
 6
 
  28092048
 
  28092370
 
  PDF   STATS   ENSEMBL   UCSC 
 
  323
 
  8
 
 34732
 
 tss
 
 0.880292
 
 0.0185271
 
 ZSCAN16; RP1-265C24.9 (margin: RP1-265C24.5; ZSCAN16; RP1-265C24.9)
 
  9.2392894
 
 1.55e-04
 
 2.71e-03
  
 
  
 17
 
  75315486
 
  75315868
 
  PDF   STATS   ENSEMBL   UCSC 
 
  383
 
  8
 
 16804
 
 gene
 
 0.015659
 
 0.8553305
 
 SEPT9 (margin: SEPT9)
 
  9.1589164
 
 1.55e-04
 
 2.71e-03
  
 
  
 16
 
  66638210
 
  66638438
 
  PDF   STATS   ENSEMBL   UCSC 
 
  229
 
  8
 
 15283
 
 tss
 
 0.836301
 
 0.0094402
 
 CMTM3 (margin: CMTM3; CMTM4)
 
  9.0992146
 
 1.55e-04
 
 2.71e-03
  
 
  
 5
 
   1882775
 
   1883011
 
  PDF   STATS   ENSEMBL   UCSC 
 
  237
 
  8
 
 32645
 
 island
 
 0.678166
 
 0.0279288
 
 CTD-2194D22.3; IRX4 (margin: CTD-2194D22.3; CTD-2194D22.4; IRX4)
 
  9.0814996
 
 1.55e-04
 
 2.71e-03
  
 
  
 2
 
  75788212
 
  75788552
 
  PDF   STATS   ENSEMBL   UCSC 
 
  341
 
  8
 
 23469
 
 tss
 
 0.128480
 
 0.9854770
 
 EVA1A (margin: EVA1A)
 
  9.0768124
 
 1.55e-04
 
 2.71e-03
  
 
  
 2
 
 191045309
 
 191045885
 
  PDF   STATS   ENSEMBL   UCSC 
 
  577
 
  8
 
 22960
 
 gene;island
 
 0.121313
 
 0.9788461
 
 C2orf88 (margin: C2orf88; HIBCH)
 
  8.9517912
 
 1.55e-04
 
 2.71e-03
  
 
  
 2
 
  69664562
 
  69664869
 
  PDF   STATS   ENSEMBL   UCSC 
 
  308
 
  8
 
 24369
 
 island
 
 0.003569
 
 0.5193702
 
 NFU1 (margin: NFU1)
 
  8.9438722
 
 1.55e-04
 
 2.71e-03
  
 
  
 10
 
  43892790
 
  43893015
 
  PDF   STATS   ENSEMBL   UCSC 
 
  226
 
  8
 
 4570
 
 gene;tss;island
 
 0.861726
 
 0.0157829
 
 HNRNPF (margin: HNRNPF)
 
  8.9168900
 
 1.55e-04
 
 2.71e-03
  
 
  
 11
 
  87908631
 
  87908938
 
  PDF   STATS   ENSEMBL   UCSC 
 
  308
 
  8
 
 7612
 
 tss
 
 0.102286
 
 0.9712194
 
 MIR3166; RAB38 (margin: MIR3166; RAB38)
 
  8.7599356
 
 1.55e-04
 
 2.71e-03
  
 
  
 18
 
  59560091
 
  59560495
 
  PDF   STATS   ENSEMBL   UCSC 
 
  405
 
  8
 
 19547
 
 island
 
 0.787620
 
 0.0109558
 
 RNF152 (margin: RNF152)
 
  8.6969522
 
 1.55e-04
 
 2.71e-03
  
 
  
 8
 
 143868013
 
 143868262
 
  PDF   STATS   ENSEMBL   UCSC 
 
  250
 
  8
 
 39939
 
 tss
 
 0.026861
 
 0.8841790
 
 RP11-706C16.8; LY6D (margin: RP11-706C16.8; LYNX1; LY6D)
 
  8.6679307
 
 1.55e-04
 
 2.71e-03
  
 
  
 12
 
 104609432
 
 104610064
 
  PDF   STATS   ENSEMBL   UCSC 
 
  633
 
  8
 
 10736
 
 island
 
 0.056908
 
 0.9827723
 
 TXNRD1 (margin: TXNRD1)
 
  8.6550857
 
 1.55e-04
 
 2.71e-03
  
 
  
 3
 
 139258658
 
 139258948
 
  PDF   STATS   ENSEMBL   UCSC 
 
  291
 
  8
 
 29539
 
 island
 
 0.093103
 
 0.9744209
 
 RP11-319G6.1; RBP1 (margin: RP11-319G6.1; RBP1)
 
  8.6161963
 
 1.55e-04
 
 2.71e-03
  
 
  
 22
 
  45596797
 
  45597035
 
  PDF   STATS   ENSEMBL   UCSC 
 
  239
 
  8
 
 26899
 
 gene;island
 
 0.153226
 
 0.9834902
 
 KIAA0930; MIR1249 (margin: KIAA0930; MIR1249)
 
  8.5336470
 
 1.55e-04
 
 2.71e-03
  
 
  
 11
 
  94278324
 
  94278603
 
  PDF   STATS   ENSEMBL   UCSC 
 
  280
 
  8
 
 6867
 
 gene;island
 
 0.118704
 
 0.9798691
 
 PIWIL4; FUT4; RP11-867G2.8 (margin: PIWIL4; FUT4; RP11-867G2.8)
 
  8.4337859
 
 1.55e-04
 
 2.71e-03
  
 
  
 1
 
  57111099
 
  57111511
 
  PDF   STATS   ENSEMBL   UCSC 
 
  413
 
  8
 
 553
 
 gene;island
 
 0.678122
 
 0.0097367
 
 PRKAA2; PPAP2B (margin: PRKAA2; PPAP2B)
 
  8.4048261
 
 1.55e-04
 
 2.71e-03
  
 
  
 19
 
  12267308
 
  12267796
 
  PDF   STATS   ENSEMBL   UCSC 
 
  489
 
  8
 
 21595
 
 island
 
 0.025189
 
 0.9067951
 
 ZNF625-ZNF20; ZNF625 (margin: ZNF136; ZNF625-ZNF20; ZNF625)
 
  8.2802062
 
 1.55e-04
 
 2.71e-03
  
 
  
 10
 
 119302050
 
 119302318
 
  PDF   STATS   ENSEMBL   UCSC 
 
  269
 
  8
 
 4785
 
 gene;island
 
 0.132328
 
 0.9797465
 
 EMX2; EMX2OS (margin: EMX2; EMX2OS)
 
  8.1046453
 
 1.55e-04
 
 2.71e-03
  
 
  
 2
 
 220083696
 
 220083951
 
  PDF   STATS   ENSEMBL   UCSC 
 
  256
 
  8
 
 23895
 
 tss
 
 0.770464
 
 0.0101490
 
 ABCB6; ATG9A (margin: ZFAND2B; ANKZF1; ABCB6; ATG9A; AC068946.1)
 
  8.0737309
 
 1.55e-04
 
 2.71e-03
  
 
  
 7
 
  94537716
 
  94537893
 
  PDF   STATS   ENSEMBL   UCSC 
 
  178
 
  8
 
 37723
 
 tss
 
 0.817289
 
 0.0109118
 
 PPP1R9A (margin: PPP1R9A)
 
  8.0299561
 
 1.55e-04
 
 2.71e-03
  
 
  
 1
 
 171810105
 
 171810570
 
  PDF   STATS   ENSEMBL   UCSC 
 
  466
 
  8
 
 2093
 
 tss;island
 
 0.039977
 
 0.8335154
 
 DNM3 (margin: DNM3)
 
  7.9035481
 
 1.55e-04
 
 2.71e-03
  
 
  
 3
 
  48700269
 
  48700498
 
  PDF   STATS   ENSEMBL   UCSC 
 
  230
 
  8
 
 29095
 
 island
 
 0.422085
 
 0.0058607
 
 RP11-148G20.1; CELSR3; NCKIPSD (margin: RP11-148G20.1; RP11-572O6.1; CELSR3; NCKIPSD)
 
  7.8997786
 
 1.55e-04
 
 2.71e-03
  
 
  
 2
 
 228029433
 
 228029856
 
  PDF   STATS   ENSEMBL   UCSC 
 
  424
 
  8
 
 23058
 
 gene;tss;island
 
 0.750292
 
 0.0148669
 
 COL4A3; COL4A4 (margin: COL4A3; COL4A4)
 
  7.8140285
 
 1.55e-04
 
 2.71e-03
  
 
  
 7
 
   5111488
 
   5111669
 
  PDF   STATS   ENSEMBL   UCSC 
 
  182
 
  8
 
 37420
 
 tss;island
 
 0.204464
 
 0.9746061
 
 RBAK (margin: RBAK)
 
  7.6761711
 
 1.55e-04
 
 2.71e-03
  
 
  
 5
 
  67583972
 
  67584380
 
  PDF   STATS   ENSEMBL   UCSC 
 
  409
 
  8
 
 31637
 
 gene;island
 
 0.637094
 
 0.0098041
 
 PIK3R1 (margin: PIK3R1)
 
  7.5051623
 
 1.55e-04
 
 2.71e-03
  
 
  
 6
 
  46097392
 
  46097695
 
  PDF   STATS   ENSEMBL   UCSC 
 
  304
 
  8
 
 35018
 
 tss
 
 0.547611
 
 0.0051924
 
 ENPP4; RP1-8B1.4 (margin: ENPP4; RP1-8B1.4)
 
  7.4712522
 
 1.55e-04
 
 2.71e-03
  
 
  
 3
 
  46448496
 
  46448963
 
  PDF   STATS   ENSEMBL   UCSC 
 
  468
 
  8
 
 28274
 
 tss
 
 0.040052
 
 0.8648563
 
 ACKR5; RP11-24F11.2 (margin: ACKR5; RP11-24F11.2)
 
  7.4311082
 
 1.55e-04
 
 2.71e-03
  
 
  
 10
 
  63422511
 
  63422841
 
  PDF   STATS   ENSEMBL   UCSC 
 
  331
 
  8
 
 5769
 
 island
 
 0.692062
 
 0.0212290
 
 C10orf107 (margin: C10orf107)
 
  7.4270808
 
 1.55e-04
 
 2.71e-03
  
 
  
 1
 
 220921216
 
 220921670
 
  PDF   STATS   ENSEMBL   UCSC 
 
  455
 
  8
 
 2262
 
 tss
 
 0.546819
 
 0.0090858
 
 MARC2 (margin: MARC2)
 
  7.2793054
 
 1.55e-04
 
 2.71e-03
  
 
  
 4
 
  55991683
 
  55991943
 
  PDF   STATS   ENSEMBL   UCSC 
 
  261
 
  8
 
 31057
 
 island
 
 0.720908
 
 0.0249785
 
 KDR (margin: KDR)
 
  7.2660773
 
 1.55e-04
 
 2.71e-03
  
 
  
 10
 
  75670653
 
  75670903
 
  PDF   STATS   ENSEMBL   UCSC 
 
  251
 
  8
 
 4638
 
 gene;island
 
 0.181772
 
 0.9713534
 
 PLAU; C10orf55 (margin: PLAU; C10orf55)
 
  7.2548476
 
 1.55e-04
 
 2.71e-03
  
 
  
 7
 
  45026259
 
  45026583
 
  PDF   STATS   ENSEMBL   UCSC 
 
  325
 
  8
 
 37576
 
 tss;island
 
 0.009017
 
 0.5106053
 
 SNHG15 (margin: MYO1G; SNHG15)
 
  7.1600897
 
 1.55e-04
 
 2.71e-03
  
 
  
 15
 
  45421578
 
  45422054
 
  PDF   STATS   ENSEMBL   UCSC 
 
  477
 
  8
 
 13327
 
 gene
 
 0.179751
 
 0.9389789
 
 DUOX1; DUOXA1 (margin: DUOXA2; DUOX1; DUOXA1)
 
  7.1564993
 
 1.55e-04
 
 2.71e-03
  
 
  
 4
 
  48491941
 
  48492344
 
  PDF   STATS   ENSEMBL   UCSC 
 
  404
 
  8
 
 31033
 
 island
 
 0.141045
 
 0.9554332
 
 SLC10A4; ZAR1 (margin: SLC10A4; ZAR1; FRYL)
 
  7.1178030
 
 1.55e-04
 
 2.71e-03
  
 
  
 11
 
  26353484
 
  26353811
 
  PDF   STATS   ENSEMBL   UCSC 
 
  328
 
  8
 
 8140
 
 island
 
 0.616941
 
 0.0202807
 
 ANO3 (margin: ANO3)
 
  7.1086205
 
 1.55e-04
 
 2.71e-03
  
 
  
 2
 
  70995349
 
  70995607
 
  PDF   STATS   ENSEMBL   UCSC 
 
  259
 
  8
 
 23420
 
 tss;island
 
 0.359977
 
 0.9850263
 
 ADD2 (margin: ADD2; FIGLA)
 
  6.9520370
 
 1.55e-04
 
 2.71e-03
  
 
  
 3
 
  44902933
 
  44903330
 
  PDF   STATS   ENSEMBL   UCSC 
 
  398
 
  8
 
 28264
 
 tss
 
 0.020158
 
 0.5103007
 
 KIF15; TMEM42; MIR564 (margin: KIF15; TMEM42; MIR564)
 
  6.9304119
 
 1.55e-04
 
 2.71e-03
  
 
  
 13
 
  20805380
 
  20805679
 
  PDF   STATS   ENSEMBL   UCSC 
 
  300
 
  8
 
 11330
 
 tss
 
 0.152697
 
 0.9272125
 
 GJB6 (margin: GJB6)
 
  6.8250944
 
 1.55e-04
 
 2.71e-03
  
 
  
 1
 
 150208519
 
 150208726
 
  PDF   STATS   ENSEMBL   UCSC 
 
  208
 
  8
 
 1903
 
 tss;island
 
 0.004783
 
 0.3160370
 
 RNU2-17P; ANP32E (margin: RNU2-17P; ANP32E)
 
  6.7932595
 
 1.55e-04
 
 2.71e-03
  
 
  
 10
 
 118765099
 
 118765335
 
  PDF   STATS   ENSEMBL   UCSC 
 
  237
 
  8
 
 5387
 
 tss;island
 
 0.584996
 
 0.0146998
 
 KIAA1598 (margin: KIAA1598)
 
  6.7646326
 
 1.55e-04
 
 2.71e-03
  
 
  
 11
 
 122852412
 
 122852698
 
  PDF   STATS   ENSEMBL   UCSC 
 
  287
 
  8
 
 7775
 
 tss
 
 0.917900
 
 0.1612890
 
 BSX (margin: BSX)
 
  6.7514139
 
 1.55e-04
 
 2.71e-03
  
 
  
 10
 
  25464008
 
  25464259
 
  PDF   STATS   ENSEMBL   UCSC 
 
  252
 
  8
 
 4991
 
 tss
 
 0.728679
 
 0.0269776
 
 GPR158; GPR158-AS1 (margin: GPR158; GPR158-AS1)
 
  6.7300838
 
 1.55e-04
 
 2.71e-03
  
 
  
 12
 
 101603835
 
 101604258
 
  PDF   STATS   ENSEMBL   UCSC 
 
  424
 
  8
 
 10717
 
 island
 
 0.701681
 
 0.0191870
 
 SLC5A8 (margin: SLC5A8; RNU6-768P)
 
  6.6518548
 
 1.55e-04
 
 2.71e-03
  
 
  
 18
 
  43652255
 
  43652594
 
  PDF   STATS   ENSEMBL   UCSC 
 
  340
 
  8
 
 19251
 
 tss
 
 0.897404
 
 0.0792793
 
 PSTPIP2 (margin: PSTPIP2; Y_RNA; ATP5A1)
 
  6.6241596
 
 1.55e-04
 
 2.71e-03
  
 
  
 6
 
  88875236
 
  88875844
 
  PDF   STATS   ENSEMBL   UCSC 
 
  609
 
  8
 
 36315
 
 island
 
 0.506668
 
 0.0267115
 
 CNR1 (margin: CNR1)
 
  6.5825788
 
 1.55e-04
 
 2.71e-03
  
 
  
 13
 
 113776873
 
 113777109
 
  PDF   STATS   ENSEMBL   UCSC 
 
  237
 
  8
 
 11548
 
 tss
 
 0.115509
 
 0.9330823
 
 F7; F10 (margin: F7; F10; KARSP2; F10-AS1)
 
  6.5359510
 
 1.55e-04
 
 2.71e-03
  
 
  
 3
 
 128336483
 
 128337044
 
  PDF   STATS   ENSEMBL   UCSC 
 
  562
 
  8
 
 29456
 
 island
 
 0.543072
 
 0.0157489
 
 RPN1 (margin: RPN1)
 
  6.4961333
 
 1.55e-04
 
 2.71e-03
  
 
  
 15
 
  52587751
 
  52588279
 
  PDF   STATS   ENSEMBL   UCSC 
 
  529
 
  8
 
 14149
 
 island
 
 0.476469
 
 0.0119506
 
 MYO5C (margin: MYO5C; MYO5A)
 
  6.3992655
 
 1.55e-04
 
 2.71e-03
  
 
  
 6
 
  71122912
 
  71123290
 
  PDF   STATS   ENSEMBL   UCSC 
 
  379
 
  8
 
 36238
 
 island
 
 0.011426
 
 0.5084320
 
 FAM135A; RP11-462G2.2 (margin: FAM135A; RP11-462G2.2)
 
  6.3699911
 
 1.55e-04
 
 2.71e-03
  
 
  
 11
 
  63438586
 
  63439313
 
  PDF   STATS   ENSEMBL   UCSC 
 
  728
 
 13
 
 8371
 
 island
 
 0.723416
 
 0.0134766
 
 ATL3 (margin: RTN3; ATL3)
 
  6.3572999
 
 1.30e-04
 
 2.71e-03
  
 
  
 5
 
 139927007
 
 139927242
 
  PDF   STATS   ENSEMBL   UCSC 
 
  236
 
  8
 
 32342
 
 tss
 
 0.010983
 
 0.4587885
 
 ANKHD1; ANKHD1-EIF4EBP3; EIF4EBP3; SRA1 (margin: ANKHD1; ANKHD1-EIF4EBP3; EIF4EBP3; SRA1; APBB3)
 
  6.3202216
 
 1.55e-04
 
 2.71e-03
  
 
  
 17
 
  42733527
 
  42733729
 
  PDF   STATS   ENSEMBL   UCSC 
 
  203
 
  8
 
 17412
 
 tss;island
 
 0.920764
 
 0.2210926
 
 C17orf104 (margin: RP11-1072C15.3; C17orf104; RP11-1072C15.2)
 
  6.3131820
 
 1.55e-04
 
 2.71e-03
  
 
  
 13
 
  36050788
 
  36051073
 
  PDF   STATS   ENSEMBL   UCSC 
 
  286
 
  8
 
 11127
 
 gene;island
 
 0.138508
 
 0.9020724
 
 NBEA; MAB21L1 (margin: NBEA; MAB21L1)
 
  6.2882485
 
 1.55e-04
 
 2.71e-03
  
 
  
 20
 
  62680970
 
  62681428
 
  PDF   STATS   ENSEMBL   UCSC 
 
  459
 
 11
 
 26380
 
 island
 
 0.815018
 
 0.0133988
 
 TCEA2; ZNF512B; SOX18 (margin: LINC00176; TCEA2; RP13-152O15.5; ZNF512B; SOX18)
 
  6.2709974
 
 1.28e-04
 
 2.71e-03
  
 
  
 5
 
 131592959
 
 131593413
 
  PDF   STATS   ENSEMBL   UCSC 
 
  455
 
 10
 
 33086
 
 island
 
 0.277509
 
 0.9571581
 
 PDLIM4; P4HA2 (margin: PDLIM4; P4HA2)
 
  6.1916056
 
 1.30e-04
 
 2.71e-03
  
 
  
 6
 
  42947051
 
  42947317
 
  PDF   STATS   ENSEMBL   UCSC 
 
  267
 
  8
 
 34986
 
 tss
 
 0.019655
 
 0.6177645
 
 PEX6 (margin: PPP2R5D; PEX6)
 
  6.1277245
 
 1.55e-04
 
 2.71e-03
  
 
  
 1
 
   9884565
 
   9884732
 
  PDF   STATS   ENSEMBL   UCSC 
 
  168
 
  8
 
 1230
 
 tss;island
 
 0.183542
 
 0.0079481
 
 CLSTN1 (margin: CLSTN1)
 
  6.0918472
 
 1.55e-04
 
 2.71e-03
  
 
  
 1
 
  84326462
 
  84326856
 
  PDF   STATS   ENSEMBL   UCSC 
 
  395
 
  8
 
 3406
 
 island
 
 0.506696
 
 0.0125745
 
 RP11-475O6.1 (margin: RP11-475O6.1; TTLL7)
 
  6.0713852
 
 1.55e-04
 
 2.71e-03
  
 
  
 6
 
 152125861
 
 152126441
 
  PDF   STATS   ENSEMBL   UCSC 
 
  581
 
  8
 
 34511
 
 gene;tss;island
 
 0.590549
 
 0.9911602
 
 ESR1 (margin: ESR1; RP3-443C4.2)
 
  6.0686287
 
 1.55e-04
 
 2.71e-03
  
 
  
 10
 
  54073047
 
  54073642
 
  PDF   STATS   ENSEMBL   UCSC 
 
  596
 
  8
 
 5086
 
 tss;island
 
 0.356181
 
 0.0131924
 
 DKK1; PRKG1-AS1 (margin: DKK1; PRKG1-AS1)
 
  6.0574530
 
 1.55e-04
 
 2.71e-03
  
 
  
 1
 
 115632236
 
 115632727
 
  PDF   STATS   ENSEMBL   UCSC 
 
  492
 
  8
 
 1857
 
 tss
 
 0.013920
 
 0.5138926
 
 TSPAN2 (margin: RP4-666F24.3; TSPAN2)
 
  6.0366140
 
 1.55e-04
 
 2.71e-03
  
 
  
 13
 
  28493913
 
  28494177
 
  PDF   STATS   ENSEMBL   UCSC 
 
  265
 
  8
 
 11633
 
 island
 
 0.392318
 
 0.9439982
 
 PDX1; PDX1-AS1 (margin: PDX1; PDX1-AS1)
 
  6.0038396
 
 1.55e-04
 
 2.71e-03
  
 
  
 1
 
  17445660
 
  17446162
 
  PDF   STATS   ENSEMBL   UCSC 
 
  503
 
  8
 
 2757
 
 island
 
 0.436290
 
 0.0146407
 
 PADI2 (margin: PADI2)
 
  5.9662542
 
 1.55e-04
 
 2.71e-03
  
 
  
 12
 
  89746328
 
  89746771
 
  PDF   STATS   ENSEMBL   UCSC 
 
  444
 
  8
 
 9906
 
 tss
 
 0.002736
 
 0.1990213
 
 DUSP6 (margin: DUSP6)
 
  5.9475197
 
 1.55e-04
 
 2.71e-03
  
 
  
 1
 
  26737005
 
  26737470
 
  PDF   STATS   ENSEMBL   UCSC 
 
  466
 
  8
 
 2895
 
 island
 
 0.279963
 
 0.9747428
 
 LIN28A (margin: LIN28A)
 
  5.9143631
 
 1.55e-04
 
 2.71e-03
  
 
  
 17
 
  15244887
 
  15245237
 
  PDF   STATS   ENSEMBL   UCSC 
 
  351
 
  8
 
 18040
 
 island
 
 0.435952
 
 0.9779506
 
 TEKT3 (margin: TEKT3)
 
  5.9044424
 
 1.55e-04
 
 2.71e-03
  
 
  
 22
 
  44576869
 
  44577240
 
  PDF   STATS   ENSEMBL   UCSC 
 
  372
 
  8
 
 27211
 
 tss
 
 0.226517
 
 0.9443940
 
 PARVG (margin: PARVB; PARVG)
 
  5.8809039
 
 1.55e-04
 
 2.71e-03
  
 
  
 1
 
  76262302
 
  76262984
 
  PDF   STATS   ENSEMBL   UCSC 
 
  683
 
  8
 
 3390
 
 island
 
 0.384534
 
 0.9802758
 
 RABGGTB; MSH4 (margin: ACADM; RABGGTB; SNORD45C; SNORD45A; SNORD45B; MSH4)
 
  5.8805666
 
 1.55e-04
 
 2.71e-03
  
 
  
 12
 
   7260546
 
   7260888
 
  PDF   STATS   ENSEMBL   UCSC 
 
  343
 
  8
 
 9044
 
 gene;tss
 
 0.019868
 
 0.9015680
 
 C1RL-AS1; C1RL (margin: C1RL-AS1; RNU6-485P; C1RL)
 
  5.8797826
 
 1.55e-04
 
 2.71e-03
  
 
  
 2
 
 113992762
 
 113993313
 
  PDF   STATS   ENSEMBL   UCSC 
 
  552
 
  8
 
 22834
 
 gene;tss;island
 
 0.986977
 
 0.5636940
 
 AC016683.6; PAX8 (margin: AC016683.6; RP11-65I12.1; PAX8)
 
  5.8773328
 
 1.55e-04
 
 2.71e-03
  
 
  
 5
 
 100238655
 
 100239071
 
  PDF   STATS   ENSEMBL   UCSC 
 
  417
 
  8
 
 32984
 
 island
 
 0.113773
 
 0.0051647
 
 ST8SIA4 (margin: ST8SIA4)
 
  5.8533304
 
 1.55e-04
 
 2.71e-03
  
 
  
 7
 
   8301971
 
   8302301
 
  PDF   STATS   ENSEMBL   UCSC 
 
  331
 
  8
 
 37438
 
 tss;island
 
 0.657986
 
 0.0405902
 
 AC007009.1; AC007128.1; ICA1 (margin: AC007009.1; AC007128.1; ICA1)
 
  5.8266820
 
 1.55e-04
 
 2.71e-03
  
 
  
 7
 
  27187372
 
  27187691
 
  PDF   STATS   ENSEMBL   UCSC 
 
  320
 
  8
 
 37493
 
 tss
 
 0.397123
 
 0.9772782
 
 HOXA-AS3; HOXA3; HOXA6 (margin: HOXA-AS3; RP1-170O19.21; HOXA3; HOXA5; HOXA6; HOXA7)
 
  5.8117806
 
 1.55e-04
 
 2.71e-03
  
 
  
 18
 
  47792708
 
  47793175
 
  PDF   STATS   ENSEMBL   UCSC 
 
  468
 
  8
 
 19508
 
 island
 
 0.281794
 
 0.0190631
 
 CCDC11; MBD1 (margin: CCDC11; MBD1)
 
  5.7467574
 
 1.55e-04
 
 2.71e-03
  
 
  
 17
 
  80797881
 
  80798078
 
  PDF   STATS   ENSEMBL   UCSC 
 
  198
 
  8
 
 16959
 
 gene
 
 0.368091
 
 0.0123377
 
 TBCD; ZNF750 (margin: TBCD; ZNF750)
 
  5.7167758
 
 1.55e-04
 
 2.71e-03
  
 
  
 11
 
  67806337
 
  67806668
 
  PDF   STATS   ENSEMBL   UCSC 
 
  332
 
  8
 
 8564
 
 island
 
 0.009860
 
 0.4123389
 
 TCIRG1 (margin: ALDH3B1; NDUFS8; MIR4691; TCIRG1; RP11-802E16.3; RP5-901A4.1)
 
  5.7126210
 
 1.55e-04
 
 2.71e-03
  
 
  
 14
 
  55034288
 
  55034666
 
  PDF   STATS   ENSEMBL   UCSC 
 
  379
 
  8
 
 12859
 
 island
 
 0.004207
 
 0.2308218
 
 SAMD4A (margin: SAMD4A)
 
  5.6717934
 
 1.55e-04
 
 2.71e-03
  
 
  
 4
 
  57976556
 
  57976944
 
  PDF   STATS   ENSEMBL   UCSC 
 
  389
 
  8
 
 30416
 
 tss
 
 0.146426
 
 0.6497719
 
 RP11-12A1.1; IGFBP7 (margin: RP11-12A1.1; IGFBP7; snoU13)
 
  5.6440123
 
 1.55e-04
 
 2.71e-03
  
 
  
 19
 
   9546252
 
   9546377
 
  PDF   STATS   ENSEMBL   UCSC 
 
  126
 
  8
 
 20424
 
 tss
 
 0.013176
 
 0.3850013
 
 ZNF266 (margin: ZNF266)
 
  5.5456898
 
 1.55e-04
 
 2.71e-03
  
 
  
 2
 
 160761085
 
 160761622
 
  PDF   STATS   ENSEMBL   UCSC 
 
  538
 
  8
 
 24812
 
 island
 
 0.357431
 
 0.9617505
 
 LY75; LY75-CD302 (margin: LY75; LY75-CD302)
 
  5.4565236
 
 1.55e-04
 
 2.71e-03
  
 
  
 2
 
 176971820
 
 176972113
 
  PDF   STATS   ENSEMBL   UCSC 
 
  294
 
  8
 
 24896
 
 island
 
 0.020965
 
 0.4737342
 
 HOXD11; AC009336.1; HOXD10 (margin: HOXD13; HOXD12; HOXD11; AC009336.1; HOXD10)
 
  5.4149073
 
 1.55e-04
 
 2.71e-03
  
 
  
 11
 
 119455041
 
 119455905
 
  PDF   STATS   ENSEMBL   UCSC 
 
  865
 
  8
 
 8885
 
 island
 
 0.012682
 
 0.3811394
 
  (margin: )
 
  5.3766826
 
 1.55e-04
 
 2.71e-03
  
 
  
 7
 
  87104724
 
  87105416
 
  PDF   STATS   ENSEMBL   UCSC 
 
  693
 
  8
 
 38635
 
 island
 
 0.471797
 
 0.9764397
 
 ABCB4 (margin: ABCB4)
 
  5.3513182
 
 1.55e-04
 
 2.71e-03
  
 
  
 19
 
  58446745
 
  58446988
 
  PDF   STATS   ENSEMBL   UCSC 
 
  244
 
  8
 
 21129
 
 tss
 
 0.952230
 
 0.3611145
 
 ZNF418 (margin: ZNF418; ZNF256)
 
  5.3257904
 
 1.55e-04
 
 2.71e-03
  
 
  
 5
 
  59189649
 
  59189934
 
  PDF   STATS   ENSEMBL   UCSC 
 
  286
 
  8
 
 32079
 
 tss
 
 0.726217
 
 0.0549849
 
 PDE4D (margin: PDE4D)
 
  5.3147381
 
 1.55e-04
 
 2.71e-03
  
 
  
 11
 
  46366643
 
  46367100
 
  PDF   STATS   ENSEMBL   UCSC 
 
  458
 
  8
 
 6598
 
 gene;island
 
 0.185126
 
 0.9014546
 
 DGKZ (margin: DGKZ)
 
  5.2247476
 
 1.55e-04
 
 2.71e-03
  
 
  
 11
 
    843897
 
    844400
 
  PDF   STATS   ENSEMBL   UCSC 
 
  504
 
  8
 
 7033
 
 tss
 
 0.676259
 
 0.0561188
 
 TSPAN4; POLR2L (margin: EFCAB4A; CD151; TSPAN4; AP006621.8; POLR2L)
 
  5.1931291
 
 1.55e-04
 
 2.71e-03
  
 
  
 6
 
 125684510
 
 125684960
 
  PDF   STATS   ENSEMBL   UCSC 
 
  451
 
  8
 
 36460
 
 island
 
 0.427742
 
 0.0230789
 
  (margin: RP11-735G4.1)
 
  5.1899493
 
 1.55e-04
 
 2.71e-03
  
 
  
 1
 
 111746873
 
 111747324
 
  PDF   STATS   ENSEMBL   UCSC 
 
  452
 
  8
 
 3589
 
 island
 
 0.440277
 
 0.0257893
 
 CHI3L2; DENND2D (margin: CHI3L2; DENND2D)
 
  5.1840508
 
 1.55e-04
 
 2.71e-03
  
 
  
 20
 
   1246703
 
   1247058
 
  PDF   STATS   ENSEMBL   UCSC 
 
  356
 
  8
 
 25935
 
 island
 
 0.317867
 
 0.9296874
 
 RAD21L1; SNPH (margin: RAD21L1; SNPH)
 
  5.1472050
 
 1.55e-04
 
 2.71e-03
  
 
  
 2
 
  20866107
 
  20866414
 
  PDF   STATS   ENSEMBL   UCSC 
 
  308
 
  8
 
 23215
 
 tss
 
 0.419811
 
 0.9834035
 
 GDF7 (margin: GDF7; AC012065.7; RP11-130L8.1)
 
  5.1441116
 
 1.55e-04
 
 2.71e-03
  
 
  
 12
 
  12503186
 
  12503544
 
  PDF   STATS   ENSEMBL   UCSC 
 
  359
 
  8
 
 9569
 
 tss
 
 0.365928
 
 0.0192856
 
 MANSC1 (margin: RP11-757G14.1; LOH12CR1; MANSC1; LOH12CR2)
 
  5.1246918
 
 1.55e-04
 
 2.71e-03
  
 
  
 3
 
  10857688
 
  10857800
 
  PDF   STATS   ENSEMBL   UCSC 
 
  113
 
  8
 
 28153
 
 tss;island
 
 0.410025
 
 0.9607461
 
 SLC6A11 (margin: SLC6A11)
 
  5.0821526
 
 1.55e-04
 
 2.71e-03
  
 
  
 6
 
 166139905
 
 166140706
 
  PDF   STATS   ENSEMBL   UCSC 
 
  802
 
  8
 
 36621
 
 island
 
 0.263519
 
 0.9482283
 
 PDE10A (margin: PDE10A; RNU6-730P)
 
  5.0766239
 
 1.55e-04
 
 2.71e-03
  
 
  
 8
 
  24772137
 
  24772513
 
  PDF   STATS   ENSEMBL   UCSC 
 
  377
 
  8
 
 39246
 
 gene;island
 
 0.673926
 
 0.9825675
 
 NEFM; GS1-72M22.1 (margin: NEFM; RP11-624C23.1; GS1-72M22.1)
 
  4.9951259
 
 1.55e-04
 
 2.71e-03
  
 
  
 12
 
 111284527
 
 111284806
 
  PDF   STATS   ENSEMBL   UCSC 
 
  280
 
  8
 
 10011
 
 tss
 
 0.324939
 
 0.9268785
 
 RP1-74B13.2; CCDC63 (margin: RP1-74B13.2; CCDC63; RPL29P25)
 
  4.9849673
 
 1.55e-04
 
 2.71e-03
  
 
  
 2
 
  17721431
 
  17721803
 
  PDF   STATS   ENSEMBL   UCSC 
 
  373
 
  8
 
 23200
 
 tss
 
 0.071875
 
 0.6378726
 
 VSNL1 (margin: VSNL1)
 
  4.9499881
 
 1.55e-04
 
 2.71e-03
  
 
  
 20
 
  21685834
 
  21686293
 
  PDF   STATS   ENSEMBL   UCSC 
 
  460
 
 10
 
 25631
 
 tss
 
 0.645460
 
 0.9810502
 
 PAX1 (margin: PAX1)
 
  4.8828282
 
 1.30e-04
 
 2.71e-03
  
 
  
 1
 
  24513473
 
  24514013
 
  PDF   STATS   ENSEMBL   UCSC 
 
  541
 
  8
 
 2856
 
 island
 
 0.027792
 
 0.5001676
 
 IFNLR1 (margin: IFNLR1)
 
  4.8122327
 
 1.55e-04
 
 2.71e-03
  
 
  
 8
 
  22014425
 
  22014724
 
  PDF   STATS   ENSEMBL   UCSC 
 
  300
 
  8
 
 39582
 
 tss;island
 
 0.462715
 
 0.0462170
 
 SFTPC; LGI3 (margin: SFTPC; BMP1; LGI3)
 
  4.7753862
 
 1.55e-04
 
 2.71e-03
  
 
  
 10
 
  31609882
 
  31610093
 
  PDF   STATS   ENSEMBL   UCSC 
 
  212
 
  8
 
 4554
 
 gene;island
 
 0.049984
 
 0.6425161
 
 ZEB1; ZEB1-AS1 (margin: ZEB1; ZEB1-AS1)
 
  4.7535223
 
 1.55e-04
 
 2.71e-03
  
 
  
 13
 
  20806477
 
  20806813
 
  PDF   STATS   ENSEMBL   UCSC 
 
  337
 
  8
 
 11331
 
 tss;island
 
 0.556864
 
 0.9843048
 
 GJB6 (margin: GJB6)
 
  4.7066448
 
 1.55e-04
 
 2.71e-03
  
 
  
 6
 
  43276570
 
  43276781
 
  PDF   STATS   ENSEMBL   UCSC 
 
  212
 
  8
 
 34996
 
 tss
 
 0.405866
 
 0.9559165
 
 RP11-480N24.3; CRIP3; ZNF318 (margin: SLC22A7; RP11-480N24.3; CRIP3; ZNF318)
 
  4.6604917
 
 1.55e-04
 
 2.71e-03
  
 
  
 5
 
 140305947
 
 140306249
 
  PDF   STATS   ENSEMBL   UCSC 
 
  303
 
  8
 
 32357
 
 tss
 
 0.957368
 
 0.5072553
 
 PCDHA1; PCDHA2; PCDHA3; PCDHA4; PCDHA5; PCDHA6; PCDHA7; PCDHA8; PCDHA9; PCDHA10; PCDHA11; PCDHA12; PCDHA13; PCDHAC1 (margin: PCDHA1; PCDHA2; PCDHA3; PCDHA4; PCDHA5; PCDHA6; PCDHA7; PCDHA8; PCDHA9; PCDHA10; PCDHA11; PCDHA12; PCDHA13; PCDHAC1)
 
  4.6217088
 
 1.55e-04
 
 2.71e-03
  
 
  
 8
 
  33457226
 
  33457822
 
  PDF   STATS   ENSEMBL   UCSC 
 
  597
 
  8
 
 40215
 
 island
 
 0.498978
 
 0.9651037
 
 DUSP26 (margin: RP11-317N12.1; DUSP26)
 
  4.6109352
 
 1.55e-04
 
 2.71e-03
  
 
  
 2
 
 223166909
 
 223167617
 
  PDF   STATS   ENSEMBL   UCSC 
 
  709
 
  8
 
 23050
 
 gene;island
 
 0.421325
 
 0.9518984
 
 CCDC140 (margin: CCDC140; PAX3)
 
  4.5126280
 
 1.55e-04
 
 2.71e-03
  
 
  
 1
 
 156338639
 
 156339286
 
  PDF   STATS   ENSEMBL   UCSC 
 
  648
 
  8
 
 3853
 
 island
 
 0.690837
 
 0.1101999
 
 RHBG; CCT3 (margin: RHBG; CCT3)
 
  4.4447383
 
 1.55e-04
 
 2.71e-03
  
 
  
 10
 
  88126089
 
  88126306
 
  PDF   STATS   ENSEMBL   UCSC 
 
  218
 
  8
 
 5902
 
 island
 
 0.562582
 
 0.0547924
 
 GRID1 (margin: GRID1)
 
  4.3880857
 
 1.55e-04
 
 2.71e-03
  
 
  
 1
 
  26233332
 
  26233709
 
  PDF   STATS   ENSEMBL   UCSC 
 
  378
 
 11
 
 1380
 
 tss;island
 
 0.503164
 
 0.9524453
 
 STMN1; MIR3917 (margin: AL033528.1; STMN1; MIR3917)
 
  4.2144275
 
 1.28e-04
 
 2.71e-03
  
 
  
 7
 
 120968877
 
 120969174
 
  PDF   STATS   ENSEMBL   UCSC 
 
  298
 
  8
 
 37203
 
 gene;island
 
 0.827127
 
 0.2168503
 
 WNT16 (margin: WNT16)
 
  4.0219477
 
 1.55e-04
 
 2.71e-03
  
 
  
 17
 
   7348316
 
   7348490
 
  PDF   STATS   ENSEMBL   UCSC 
 
  175
 
  8
 
 17950
 
 island
 
 0.957797
 
 0.6197443
 
 RP11-104H15.7; FGF11; CHRNB1; RP11-104H15.8 (margin: RP11-104H15.7; TMEM102; FGF11; CHRNB1; RP11-104H15.9; RP11-104H15.8)
 
  3.9825720
 
 1.55e-04
 
 2.71e-03
  
 
  
 19
 
  19648890
 
  19649144
 
  PDF   STATS   ENSEMBL   UCSC 
 
  255
 
  8
 
 21821
 
 island
 
 0.291595
 
 0.8186222
 
 YJEFN3; CILP2 (margin: NDUFA13; CTC-260F20.3; YJEFN3; CILP2)
 
  3.9039949
 
 1.55e-04
 
 2.71e-03
  
 
  
 6
 
 147829799
 
 147830172
 
  PDF   STATS   ENSEMBL   UCSC 
 
  374
 
  8
 
 36549
 
 island
 
 0.736613
 
 0.9772328
 
 SAMD5 (margin: SAMD5)
 
  3.8968760
 
 1.55e-04
 
 2.71e-03
  
 
  
 11
 
  69590113
 
  69590580
 
  PDF   STATS   ENSEMBL   UCSC 
 
  468
 
  8
 
 8597
 
 island
 
 0.518482
 
 0.9558930
 
 FGF4 (margin: AP001888.1; FGF4)
 
  3.8582257
 
 1.55e-04
 
 2.71e-03
  
 
  
 11
 
  93583509
 
  93583973
 
  PDF   STATS   ENSEMBL   UCSC 
 
  465
 
  8
 
 8733
 
 island
 
 0.048085
 
 0.3903767
 
 VSTM5 (margin: VSTM5)
 
  3.8289915
 
 1.55e-04
 
 2.71e-03
  
 
  
 3
 
 111717682
 
 111718245
 
  PDF   STATS   ENSEMBL   UCSC 
 
  564
 
  8
 
 27893
 
 gene
 
 0.520577
 
 0.9570795
 
 TAGLN3 (margin: ABHD10; TAGLN3)
 
  3.8232448
 
 1.55e-04
 
 2.71e-03
  
 
  
 4
 
 156587884
 
 156588387
 
  PDF   STATS   ENSEMBL   UCSC 
 
  504
 
  8
 
 30159
 
 gene;tss;island
 
 0.383802
 
 0.0230158
 
 GUCY1A3 (margin: GUCY1A3)
 
  3.8174096
 
 1.55e-04
 
 2.71e-03
  
 
  
 11
 
  31832879
 
  31833264
 
  PDF   STATS   ENSEMBL   UCSC 
 
  386
 
  8
 
 7223
 
 tss
 
 0.755876
 
 0.9747946
 
 RCN1; PAX6 (margin: RCN1; PAX6)
 
  3.6495121
 
 1.55e-04
 
 2.71e-03
  
 
  
 17
 
  44896017
 
  44896223
 
  PDF   STATS   ENSEMBL   UCSC 
 
  207
 
  8
 
 18447
 
 island
 
 0.333662
 
 0.0512876
 
 WNT3 (margin: WNT3)
 
  3.5834675
 
 1.55e-04
 
 2.71e-03
  
 
  
 3
 
 136537607
 
 136537856
 
  PDF   STATS   ENSEMBL   UCSC 
 
  250
 
  8
 
 28614
 
 tss
 
 0.735935
 
 0.9645070
 
 SLC35G2 (margin: SLC35G2; RP11-731C17.1)
 
  3.5433003
 
 1.55e-04
 
 2.71e-03
  
 
  
 2
 
  43019484
 
  43020457
 
  PDF   STATS   ENSEMBL   UCSC 
 
  974
 
 11
 
 24245
 
 island
 
 0.641145
 
 0.9596606
 
 HAAO (margin: HAAO; FTOP1)
 
  3.5149309
 
 1.28e-04
 
 2.71e-03
  
 
  
 1
 
 202830475
 
 202830826
 
  PDF   STATS   ENSEMBL   UCSC 
 
  352
 
  8
 
 2182
 
 tss;island
 
 0.154165
 
 0.0120969
 
 RP11-480I12.7; RP11-480I12.5 (margin: RP11-480I12.7; RP11-480I12.9; RP11-480I12.5)
 
  3.5007974
 
 1.55e-04
 
 2.71e-03
  
 
  
 2
 
  74875227
 
  74875548
 
  PDF   STATS   ENSEMBL   UCSC 
 
  322
 
  8
 
 23465
 
 tss;island
 
 0.881241
 
 0.9863651
 
 M1AP (margin: SEMA4F; M1AP)
 
  2.9987949
 
 1.55e-04
 
 2.71e-03
  
 
  
 14
 
  74705942
 
  74706336
 
  PDF   STATS   ENSEMBL   UCSC 
 
  395
 
  8
 
 12997
 
 island
 
 0.622617
 
 0.8768067
 
 VSX2 (margin: VSX2)
 
  2.9188836
 
 1.55e-04
 
 2.71e-03
  
 
  
 3
 
 138153127
 
 138153381
 
  PDF   STATS   ENSEMBL   UCSC 
 
  255
 
  8
 
 28623
 
 tss
 
 0.213739
 
 0.6631616
 
 ESYT3 (margin: ESYT3)
 
  2.8866404
 
 1.55e-04
 
 2.71e-03
  
 
  
 11
 
 115630531
 
 115631762
 
  PDF   STATS   ENSEMBL   UCSC 
 
 1232
 
 14
 
 8831
 
 island
 
 0.655795
 
 0.9713687
 
 AP000797.3; LINC00900 (margin: AP000797.3; LINC00900)
 
  2.8226803
 
 1.31e-04
 
 2.71e-03
  
 
  
 7
 
  99517192
 
  99517509
 
  PDF   STATS   ENSEMBL   UCSC 
 
  318
 
 10
 
 37762
 
 tss
 
 0.056448
 
 0.3374067
 
 TRIM4 (margin: RP4-604G5.1; TRIM4; GJC3)
 
  2.7381636
 
 1.30e-04
 
 2.71e-03
  
 
  
 19
 
  40732691
 
  40732902
 
  PDF   STATS   ENSEMBL   UCSC 
 
  212
 
  8
 
 20784
 
 tss
 
 0.416655
 
 0.0970250
 
 CNTD2 (margin: MAP3K10; TTC9B; CNTD2; AKT2)
 
  2.7006342
 
 1.55e-04
 
 2.71e-03
  
 
  
 4
 
 105411974
 
 105412536
 
  PDF   STATS   ENSEMBL   UCSC 
 
  563
 
  8
 
 31218
 
 island
 
 0.135523
 
 0.0276220
 
 AC093628.1; AC004053.1; CXXC4 (margin: AC093628.1; AC004053.1; CXXC4)
 
  2.6483936
 
 1.55e-04
 
 2.71e-03
  
 
  
 17
 
  46655164
 
  46655736
 
  PDF   STATS   ENSEMBL   UCSC 
 
  573
 
 11
 
 16653
 
 gene
 
 0.843091
 
 0.9778412
 
 HOXB-AS3; HOXB3; HOXB4; MIR10A (margin: HOXB-AS3; HOXB3; HOXB4; MIR10A)
 
  2.4353144
 
 1.28e-04
 
 2.71e-03
  
 
  
 20
 
  61051317
 
  61052060
 
  PDF   STATS   ENSEMBL   UCSC 
 
  744
 
 18
 
 25872
 
 tss;island
 
 0.721073
 
 0.9215948
 
 RP13-379O24.3; GATA5 (margin: RP13-379O24.3; GATA5)
 
  1.7973298
 
 1.32e-04
 
 2.71e-03
  
 
  
 5
 
 101632286
 
 101632559
 
  PDF   STATS   ENSEMBL   UCSC 
 
  274
 
  9
 
 32198
 
 tss
 
 0.967476
 
 0.0479951
 
 SLCO4C1 (margin: RN7SKP68; SLCO4C1)
 
  9.3519268
 
 1.65e-04
 
 2.80e-03
  
 
  
 1
 
   1141617
 
   1142405
 
  PDF   STATS   ENSEMBL   UCSC 
 
  789
 
  9
 
 2414
 
 island
 
 0.627937
 
 0.0326009
 
 TNFRSF18 (margin: TTLL10; TNFRSF18; TNFRSF4; SDF4)
 
  6.4676037
 
 1.65e-04
 
 2.80e-03
  
 
  
 4
 
  96468962
 
  96469634
 
  PDF   STATS   ENSEMBL   UCSC 
 
  673
 
  9
 
 30063
 
 gene;island
 
 0.669987
 
 0.0108804
 
 RP11-710C12.1; UNC5C (margin: RP11-710C12.1; UNC5C)
 
  6.2717984
 
 1.65e-04
 
 2.80e-03
  
 
  
 10
 
 124894943
 
 124895500
 
  PDF   STATS   ENSEMBL   UCSC 
 
  558
 
  9
 
 5415
 
 tss
 
 0.516571
 
 0.9862258
 
 HMX3 (margin: HMX3)
 
  5.9400191
 
 1.65e-04
 
 2.80e-03
  
 
  
 6
 
  29855401
 
  29855636
 
  PDF   STATS   ENSEMBL   UCSC 
 
  236
 
  9
 
 33699
 
 gene
 
 0.008569
 
 0.5349753
 
 HLA-H; HCG4P7 (margin: HLA-H; HLA-T; HCG4P7)
 
  5.8650470
 
 1.65e-04
 
 2.80e-03
  
 
  
 22
 
  44576869
 
  44577265
 
  PDF   STATS   ENSEMBL   UCSC 
 
  397
 
  9
 
 26891
 
 gene
 
 0.246176
 
 0.9455744
 
 PARVG (margin: PARVB; PARVG)
 
  5.7389699
 
 1.65e-04
 
 2.80e-03
  
 
  
 21
 
  43638893
 
  43639122
 
  PDF   STATS   ENSEMBL   UCSC 
 
  230
 
  9
 
 26437
 
 gene;tss;island
 
 0.475864
 
 0.0179861
 
 ABCG1 (margin: ABCG1; RNA5SP492)
 
  5.6342263
 
 1.65e-04
 
 2.80e-03
  
 
  
 5
 
 150284302
 
 150284796
 
  PDF   STATS   ENSEMBL   UCSC 
 
  495
 
  9
 
 33313
 
 island
 
 0.292191
 
 0.9004168
 
 ZNF300 (margin: IRGM; ZNF300)
 
  5.6212828
 
 1.65e-04
 
 2.80e-03
  
 
  
 7
 
  82792053
 
  82792411
 
  PDF   STATS   ENSEMBL   UCSC 
 
  359
 
  9
 
 38625
 
 island
 
 0.148853
 
 0.0104282
 
 PCLO (margin: PCLO)
 
  4.6309793
 
 1.65e-04
 
 2.80e-03
  
 
  
 7
 
   1748302
 
   1748766
 
  PDF   STATS   ENSEMBL   UCSC 
 
  465
 
  9
 
 37405
 
 tss
 
 0.449636
 
 0.9206681
 
 ELFN1 (margin: ELFN1)
 
  3.6040850
 
 1.65e-04
 
 2.80e-03
  
 
  
 3
 
 131080235
 
 131080927
 
  PDF   STATS   ENSEMBL   UCSC 
 
  693
 
  9
 
 29479
 
 island
 
 0.459429
 
 0.9156050
 
 NUDT16P; RP11-933H2.4 (margin: NEK11; NUDT16P; RP11-933H2.4)
 
  3.5440313
 
 1.65e-04
 
 2.80e-03
  
 
  
 18
 
   6414958
 
   6415252
 
  PDF   STATS   ENSEMBL   UCSC 
 
  295
 
  9
 
 19172
 
 tss;island
 
 0.527883
 
 0.9565002
 
 L3MBTL4 (margin: L3MBTL4)
 
  3.3032432
 
 1.65e-04
 
 2.80e-03
  
 
  
 15
 
  40545143
 
  40545511
 
  PDF   STATS   ENSEMBL   UCSC 
 
  369
 
  9
 
 13585
 
 tss
 
 0.794845
 
 0.9682490
 
 RP11-133K1.2; PAK6; C15orf56 (margin: RP11-133K1.2; PAK6; C15orf56)
 
  2.9806194
 
 1.65e-04
 
 2.80e-03
  
 
  
 6
 
  44186914
 
  44187226
 
  PDF   STATS   ENSEMBL   UCSC 
 
  313
 
  9
 
 35007
 
 tss
 
 0.844003
 
 0.3456163
 
 RP1-302G2.5; SLC29A1 (margin: RP1-302G2.5; SLC29A1)
 
  2.9748192
 
 1.65e-04
 
 2.80e-03
  
 
  
 17
 
  12569089
 
  12569487
 
  PDF   STATS   ENSEMBL   UCSC 
 
  399
 
 11
 
 18028
 
 island
 
 0.371737
 
 0.8610794
 
 MYOCD (margin: MYOCD; AC005358.3)
 
  3.8743684
 
 1.90e-04
 
 3.21e-03
  
 
  
 20
 
  56725695
 
  56726185
 
  PDF   STATS   ENSEMBL   UCSC 
 
  491
 
 11
 
 26286
 
 island
 
 0.732469
 
 0.9582621
 
 C20orf85 (margin: C20orf85)
 
  3.0743535
 
 1.90e-04
 
 3.21e-03
  
 
  
 7
 
  93204959
 
  93205295
 
  PDF   STATS   ENSEMBL   UCSC 
 
  337
 
 11
 
 37714
 
 tss;island
 
 0.025972
 
 0.1543914
 
 CALCR (margin: CALCR)
 
  2.6889657
 
 1.90e-04
 
 3.21e-03
  
 
  
 5
 
  57878745
 
  57879417
 
  PDF   STATS   ENSEMBL   UCSC 
 
  673
 
 12
 
 32817
 
 island
 
 0.681921
 
 0.0275055
 
 RAB3C (margin: RAB3C)
 
  5.4643526
 
 2.01e-04
 
 3.38e-03
  
 
  
 10
 
 105212056
 
 105212813
 
  PDF   STATS   ENSEMBL   UCSC 
 
  758
 
 12
 
 6102
 
 island
 
 0.598580
 
 0.1977769
 
 RP11-225H22.4; CALHM2; CALHM1 (margin: PDCD11; RP11-225H22.4; CALHM2; CALHM1)
 
  4.0881464
 
 2.01e-04
 
 3.38e-03
  
 
  
 2
 
 160918900
 
 160919532
 
  PDF   STATS   ENSEMBL   UCSC 
 
  633
 
 12
 
 24813
 
 island
 
 0.066208
 
 0.4228631
 
 PLA2R1 (margin: PLA2R1)
 
  3.4649965
 
 2.01e-04
 
 3.38e-03
  
 
  
 17
 
  72931569
 
  72932048
 
  PDF   STATS   ENSEMBL   UCSC 
 
  480
 
 12
 
 18731
 
 island
 
 0.527000
 
 0.8618091
 
 OTOP2; OTOP3 (margin: OTOP2; OTOP3)
 
  2.2336350
 
 2.01e-04
 
 3.38e-03
  
 
  
 3
 
  48885189
 
  48885665
 
  PDF   STATS   ENSEMBL   UCSC 
 
  477
 
 10
 
 29098
 
 island
 
 0.005880
 
 0.3863444
 
 PRKAR2A-AS1; PRKAR2A (margin: PRKAR2A-AS1; PRKAR2A; SLC25A20)
 
  6.5174539
 
 2.06e-04
 
 3.42e-03
  
 
  
 5
 
  63461216
 
  63461930
 
  PDF   STATS   ENSEMBL   UCSC 
 
  715
 
 10
 
 32828
 
 island
 
 0.665439
 
 0.0390218
 
 RNF180 (margin: RNF180)
 
  5.3446270
 
 2.06e-04
 
 3.42e-03
  
 
  
 6
 
  84742998
 
  84743364
 
  PDF   STATS   ENSEMBL   UCSC 
 
  367
 
 10
 
 35099
 
 tss;island
 
 0.282185
 
 0.8581279
 
 MRAP2 (margin: RP11-51G5.1; MRAP2)
 
  3.3938224
 
 2.06e-04
 
 3.42e-03
  
 
  
 2
 
 209271164
 
 209272057
 
  PDF   STATS   ENSEMBL   UCSC 
 
  894
 
 10
 
 25050
 
 island
 
 0.796132
 
 0.9682389
 
 PTH2R (margin: PTH2R)
 
  2.7980021
 
 2.06e-04
 
 3.42e-03
  
 
  
 1
 
   2984218
 
   2984869
 
  PDF   STATS   ENSEMBL   UCSC 
 
  652
 
 10
 
 2528
 
 island
 
 0.884607
 
 0.9746941
 
 PRDM16; LINC00982 (margin: PRDM16; LINC00982; AL008733.1)
 
  2.7883034
 
 2.06e-04
 
 3.42e-03
  
 
  
 2
 
  80531483
 
  80531898
 
  PDF   STATS   ENSEMBL   UCSC 
 
  416
 
 10
 
 22756
 
 gene;island
 
 0.828147
 
 0.4533058
 
 CTNNA2; LRRTM1 (margin: CTNNA2; LRRTM1)
 
  2.5935601
 
 2.06e-04
 
 3.42e-03
  
 
  
 7
 
 130125200
 
 130126637
 
  PDF   STATS   ENSEMBL   UCSC 
 
 1438
 
 18
 
 38921
 
 island
 
 0.512617
 
 0.0201612
 
 MEST; RP11-2E11.5 (margin: MEST; MIR335; RP11-2E11.5; hsa-mir-335)
 
  5.2911284
 
 2.11e-04
 
 3.49e-03
  
 
  
 11
 
  86383241
 
  86383940
 
  PDF   STATS   ENSEMBL   UCSC 
 
  700
 
 18
 
 7610
 
 tss
 
 0.848051
 
 0.9670690
 
 ME3 (margin: ME3)
 
  1.8373611
 
 2.11e-04
 
 3.49e-03
  
 
  
 1
 
  40420503
 
  40421002
 
  PDF   STATS   ENSEMBL   UCSC 
 
  500
 
 13
 
 3111
 
 island
 
 0.016041
 
 0.5369766
 
 MFSD2A (margin: MFSD2A; RP3-342P20.2; Y_RNA)
 
  7.7030761
 
 2.28e-04
 
 3.75e-03
  
 
  
 17
 
  46670933
 
  46671635
 
  PDF   STATS   ENSEMBL   UCSC 
 
  703
 
 13
 
 16655
 
 gene;island
 
 0.548156
 
 0.0290494
 
 HOXB-AS3; HOXB3; HOXB5; HOXB6 (margin: HOXB-AS3; HOXB3; MIR10A; HOXB5; HOXB6)
 
  6.2103653
 
 2.28e-04
 
 3.75e-03
  
 
  
 1
 
 185703201
 
 185703877
 
  PDF   STATS   ENSEMBL   UCSC 
 
  677
 
 13
 
 4045
 
 island
 
 0.158705
 
 0.0170500
 
 HMCN1 (margin: HMCN1)
 
  2.9540040
 
 2.28e-04
 
 3.75e-03
  
 
  
 13
 
  37004536
 
  37005582
 
  PDF   STATS   ENSEMBL   UCSC 
 
 1047
 
 29
 
 11396
 
 tss;island
 
 0.745720
 
 0.9346212
 
 CCNA1 (margin: CCNA1)
 
  2.4614760
 
 2.32e-04
 
 3.81e-03
  
 
  
 11
 
  66034681
 
  66035276
 
  PDF   STATS   ENSEMBL   UCSC 
 
  596
 
 17
 
 6727
 
 gene
 
 0.480074
 
 0.0507028
 
 KLC2; RAB1B; RP11-867G23.1; RP11-867G23.2 (margin: KLC2; RAB1B; CNIH2; RP11-755F10.3; RP11-867G23.1; RP11-867G23.2; RP11-867G23.3; RP11-867G23.4)
 
  4.6727081
 
 2.52e-04
 
 4.14e-03
  
 
  
 1
 
  68515788
 
  68516713
 
  PDF   STATS   ENSEMBL   UCSC 
 
  926
 
 17
 
 3369
 
 island
 
 0.614361
 
 0.9551843
 
 GNG12-AS1; DIRAS3; ARL5AP3 (margin: GNG12-AS1; RP4-609E1.2; AL157407.1; DIRAS3; ARL5AP3)
 
  4.0265159
 
 2.52e-04
 
 4.14e-03
  
 
  
 8
 
  49647579
 
  49648363
 
  PDF   STATS   ENSEMBL   UCSC 
 
  785
 
 11
 
 40284
 
 island
 
 0.139527
 
 0.9715595
 
 EFCAB1 (margin: EFCAB1)
 
  6.6491421
 
 2.75e-04
 
 4.46e-03
  
 
  
 15
 
  73735098
 
  73735694
 
  PDF   STATS   ENSEMBL   UCSC 
 
  597
 
 12
 
 14289
 
 island
 
 0.463388
 
 0.9573527
 
 C15orf60 (margin: C15orf60)
 
  4.1927367
 
 2.74e-04
 
 4.46e-03
  
 
  
 11
 
   3663198
 
   3663842
 
  PDF   STATS   ENSEMBL   UCSC 
 
  645
 
 11
 
 8000
 
 island
 
 0.327204
 
 0.0230179
 
 ART5 (margin: TRPC2; ART1; ART5)
 
  4.1858447
 
 2.75e-04
 
 4.46e-03
  
 
  
 11
 
  85521839
 
  85522637
 
  PDF   STATS   ENSEMBL   UCSC 
 
  799
 
 12
 
 8707
 
 island
 
 0.258890
 
 0.0448982
 
 SYTL2 (margin: SYTL2)
 
  3.7470264
 
 2.74e-04
 
 4.46e-03
  
 
  
 2
 
 175199259
 
 175199803
 
  PDF   STATS   ENSEMBL   UCSC 
 
  545
 
 11
 
 23723
 
 tss
 
 0.603495
 
 0.9756116
 
 SP9; AC018470.1 (margin: AC018470.4; SP9; AC018470.1)
 
  3.7337391
 
 2.75e-04
 
 4.46e-03
  
 
  
 16
 
  66878426
 
  66879016
 
  PDF   STATS   ENSEMBL   UCSC 
 
  591
 
 11
 
 14783
 
 gene;island
 
 0.813533
 
 0.9791660
 
 CA7; NAE1; RP11-61A14.1 (margin: CA7; NAE1; RP11-61A14.1)
 
  3.5651164
 
 2.75e-04
 
 4.46e-03
  
 
  
 19
 
  53073242
 
  53074012
 
  PDF   STATS   ENSEMBL   UCSC 
 
  771
 
 11
 
 22360
 
 island
 
 0.772057
 
 0.4388284
 
 ZNF701 (margin: ZNF808; ZNF701; RPL39P34; CTD-3099C6.7)
 
  1.9568156
 
 2.75e-04
 
 4.46e-03
  
 
  
 22
 
  45405621
 
  45405919
 
  PDF   STATS   ENSEMBL   UCSC 
 
  299
 
  9
 
 27216
 
 tss;island
 
 0.487334
 
 0.0094622
 
 PHF21B (margin: PHF21B; RP4-753M9.1)
 
  7.0008411
 
 2.88e-04
 
 4.58e-03
  
 
  
 11
 
 122852284
 
 122852698
 
  PDF   STATS   ENSEMBL   UCSC 
 
  415
 
  9
 
 8904
 
 island
 
 0.936536
 
 0.1717095
 
 BSX (margin: BSX)
 
  6.4968736
 
 2.88e-04
 
 4.58e-03
  
 
  
 7
 
 101005832
 
 101006089
 
  PDF   STATS   ENSEMBL   UCSC 
 
  258
 
  9
 
 37802
 
 tss;island
 
 0.583787
 
 0.9885915
 
 COL26A1 (margin: COL26A1)
 
  5.5342826
 
 2.88e-04
 
 4.58e-03
  
 
  
 5
 
 174151043
 
 174151779
 
  PDF   STATS   ENSEMBL   UCSC 
 
  737
 
  9
 
 33416
 
 island
 
 0.355032
 
 0.0060265
 
 MSX2 (margin: MSX2)
 
  4.9816466
 
 2.88e-04
 
 4.58e-03
  
 
  
 7
 
 128828120
 
 128828765
 
  PDF   STATS   ENSEMBL   UCSC 
 
  646
 
  9
 
 38907
 
 island
 
 0.537283
 
 0.9681938
 
 SMO (margin: SMO)
 
  4.7123566
 
 2.88e-04
 
 4.58e-03
  
 
  
 11
 
 134145889
 
 134146324
 
  PDF   STATS   ENSEMBL   UCSC 
 
  436
 
  9
 
 8981
 
 island
 
 0.496692
 
 0.9724474
 
 GLB1L3 (margin: ACAD8; GLB1L3)
 
  4.5932410
 
 2.88e-04
 
 4.58e-03
  
 
  
 17
 
   6679254
 
   6679541
 
  PDF   STATS   ENSEMBL   UCSC 
 
  288
 
  9
 
 17053
 
 tss
 
 0.613975
 
 0.9764448
 
 XAF1; FBXO39 (margin: XAF1; FBXO39)
 
  4.2115024
 
 2.88e-04
 
 4.58e-03
  
 
  
 7
 
 121784102
 
 121784596
 
  PDF   STATS   ENSEMBL   UCSC 
 
  495
 
  9
 
 38868
 
 island
 
 0.050096
 
 0.4931000
 
 AASS (margin: AASS)
 
  3.8009656
 
 2.88e-04
 
 4.58e-03
  
 
  
 1
 
  19600673
 
  19601069
 
  PDF   STATS   ENSEMBL   UCSC 
 
  397
 
  9
 
 1301
 
 tss;island
 
 0.862707
 
 0.9851077
 
 AKR7L (margin: AKR7L; AKR7A3)
 
  3.3999919
 
 2.88e-04
 
 4.58e-03
  
 
  
 3
 
 138153127
 
 138153439
 
  PDF   STATS   ENSEMBL   UCSC 
 
  313
 
  9
 
 29521
 
 island
 
 0.223319
 
 0.7008385
 
 ESYT3 (margin: ESYT3)
 
  3.3090583
 
 2.88e-04
 
 4.58e-03
  
 
  
 4
 
 166795260
 
 166795913
 
  PDF   STATS   ENSEMBL   UCSC 
 
  654
 
  9
 
 30173
 
 gene;island
 
 0.475516
 
 0.7998924
 
 TLL1 (margin: TLL1)
 
  2.1964926
 
 2.88e-04
 
 4.58e-03
  
 
  
 1
 
 186649153
 
 186649985
 
  PDF   STATS   ENSEMBL   UCSC 
 
  833
 
 13
 
 4047
 
 island
 
 0.188892
 
 0.0187634
 
 PTGS2 (margin: PTGS2)
 
  3.4748048
 
 2.97e-04
 
 4.72e-03
  
 
  
 2
 
 210636350
 
 210636844
 
  PDF   STATS   ENSEMBL   UCSC 
 
  495
 
  8
 
 25053
 
 island
 
 0.754735
 
 0.0361600
 
 UNC80 (margin: UNC80)
 
  6.5253158
 
 3.11e-04
 
 4.77e-03
  
 
  
 21
 
  39288661
 
  39289266
 
  PDF   STATS   ENSEMBL   UCSC 
 
  606
 
  8
 
 26681
 
 island
 
 0.536400
 
 0.0303954
 
 KCNJ6 (margin: KCNJ6)
 
  6.5110894
 
 3.11e-04
 
 4.77e-03
  
 
  
 17
 
  40346279
 
  40346798
 
  PDF   STATS   ENSEMBL   UCSC 
 
  520
 
  8
 
 17358
 
 tss
 
 0.054685
 
 0.7147151
 
 GHDC (margin: HCRT; GHDC; STAT5B)
 
  6.1466814
 
 3.11e-04
 
 4.77e-03
  
 
  
 11
 
  79151611
 
  79152112
 
  PDF   STATS   ENSEMBL   UCSC 
 
  502
 
  8
 
 8697
 
 island
 
 0.568076
 
 0.0339097
 
 TENM4 (margin: TENM4)
 
  5.7666478
 
 3.11e-04
 
 4.77e-03
  
 
  
 7
 
  29519297
 
  29519656
 
  PDF   STATS   ENSEMBL   UCSC 
 
  360
 
  8
 
 36949
 
 gene
 
 0.231188
 
 0.9078701
 
 CHN2 (margin: CHN2)
 
  5.5594929
 
 3.11e-04
 
 4.77e-03
  
 
  
 20
 
  55841869
 
  55842071
 
  PDF   STATS   ENSEMBL   UCSC 
 
  203
 
  8
 
 25840
 
 tss;island
 
 0.863226
 
 0.0757580
 
 RP4-813D12.3; BMP7 (margin: RP4-813D12.3; BMP7)
 
  5.4913348
 
 3.11e-04
 
 4.77e-03
  
 
  
 4
 
   5021084
 
   5021328
 
  PDF   STATS   ENSEMBL   UCSC 
 
  245
 
  8
 
 30875
 
 island
 
 0.385327
 
 0.9743116
 
 CYTL1 (margin: CYTL1)
 
  5.2492081
 
 3.11e-04
 
 4.77e-03
  
 
  
 4
 
 165304259
 
 165304540
 
  PDF   STATS   ENSEMBL   UCSC 
 
  282
 
  8
 
 31404
 
 island
 
 0.287069
 
 0.9527567
 
 MARCH1 (margin: MARCH1)
 
  5.2287906
 
 3.11e-04
 
 4.77e-03
  
 
  
 22
 
  45809596
 
  45810043
 
  PDF   STATS   ENSEMBL   UCSC 
 
  448
 
  8
 
 26904
 
 gene
 
 0.017408
 
 0.2887377
 
 RIBC2; SMC1B (margin: RIBC2; SMC1B)
 
  5.2073263
 
 3.11e-04
 
 4.77e-03
  
 
  
 20
 
   3776212
 
   3776483
 
  PDF   STATS   ENSEMBL   UCSC 
 
  272
 
  8
 
 25977
 
 island
 
 0.632599
 
 0.0504353
 
 CDC25B (margin: CDC25B; CENPB)
 
  5.0730296
 
 3.11e-04
 
 4.77e-03
  
 
  
 11
 
   2182457
 
   2182795
 
  PDF   STATS   ENSEMBL   UCSC 
 
  339
 
  8
 
 7061
 
 tss
 
 0.212626
 
 0.9384692
 
 INS-IGF2; INS (margin: MIR4686; IGF2; INS-IGF2; INS; TH)
 
  4.9928473
 
 3.11e-04
 
 4.77e-03
  
 
  
 20
 
  43374239
 
  43374406
 
  PDF   STATS   ENSEMBL   UCSC 
 
  168
 
  8
 
 25763
 
 tss;island
 
 0.418571
 
 0.0446456
 
 KCNK15; RP11-445H22.4 (margin: KCNK15; RP11-445H22.4; RIMS4)
 
  4.7790618
 
 3.11e-04
 
 4.77e-03
  
 
  
 19
 
  53662353
 
  53662723
 
  PDF   STATS   ENSEMBL   UCSC 
 
  371
 
  8
 
 21024
 
 tss
 
 0.857039
 
 0.2613673
 
 ZNF347; ZNF665 (margin: CTD-2245F17.2; ZNF347; ZNF665)
 
  4.5294054
 
 3.11e-04
 
 4.77e-03
  
 
  
 5
 
 140562562
 
 140563400
 
  PDF   STATS   ENSEMBL   UCSC 
 
  839
 
  8
 
 31813
 
 gene;island
 
 0.662667
 
 0.9771637
 
 PCDHB16 (margin: PCDHB7; PCDHB8; PCDHB16; PCDHB9; PCDHB10)
 
  4.3725301
 
 3.11e-04
 
 4.77e-03
  
 
  
 12
 
  49582842
 
  49583112
 
  PDF   STATS   ENSEMBL   UCSC 
 
  271
 
  8
 
 10388
 
 island
 
 0.128358
 
 0.0092178
 
 TUBA1C; TUBA1A (margin: TUBA1C; TUBA1A)
 
  4.1491985
 
 3.11e-04
 
 4.77e-03
  
 
  
 2
 
 176987161
 
 176987605
 
  PDF   STATS   ENSEMBL   UCSC 
 
  445
 
  8
 
 24902
 
 island
 
 0.843578
 
 0.9863528
 
 HOXD9; HOXD-AS2 (margin: HOXD10; HOXD9; HOXD8; HOXD-AS2)
 
  3.8681833
 
 3.11e-04
 
 4.77e-03
  
 
  
 20
 
   2781316
 
   2781685
 
  PDF   STATS   ENSEMBL   UCSC 
 
  370
 
  8
 
 25551
 
 tss
 
 0.736049
 
 0.9752814
 
 CPXM1 (margin: CPXM1)
 
  3.7995638
 
 3.11e-04
 
 4.77e-03
  
 
  
 2
 
  43019819
 
  43020457
 
  PDF   STATS   ENSEMBL   UCSC 
 
  639
 
  8
 
 23322
 
 tss
 
 0.660483
 
 0.9664717
 
 HAAO (margin: HAAO; FTOP1)
 
  3.5889281
 
 3.11e-04
 
 4.77e-03
  
 
  
 6
 
  32028831
 
  32029216
 
  PDF   STATS   ENSEMBL   UCSC 
 
  386
 
  8
 
 34025
 
 gene
 
 0.434914
 
 0.9241474
 
 TNXB (margin: TNXB)
 
  3.4940396
 
 3.11e-04
 
 4.77e-03
  
 
  
 10
 
  52750736
 
  52751341
 
  PDF   STATS   ENSEMBL   UCSC 
 
  606
 
  8
 
 5746
 
 island
 
 0.851085
 
 0.3484274
 
 PRKG1 (margin: PRKG1)
 
  3.0756263
 
 3.11e-04
 
 4.77e-03
  
 
  
 11
 
  67777618
 
  67777952
 
  PDF   STATS   ENSEMBL   UCSC 
 
  335
 
  8
 
 6766
 
 gene
 
 0.015284
 
 0.1119539
 
 ALDH3B1 (margin: ALDH3B1; UNC93B1)
 
  2.9259515
 
 3.11e-04
 
 4.77e-03
  
 
  
 19
 
  10046864
 
  10047420
 
  PDF   STATS   ENSEMBL   UCSC 
 
  557
 
  8
 
 21511
 
 island
 
 0.096399
 
 0.0210506
 
 OLFM2 (margin: OLFM2)
 
  2.6042463
 
 3.11e-04
 
 4.77e-03
  
 
  
 4
 
  13546111
 
  13546673
 
  PDF   STATS   ENSEMBL   UCSC 
 
  563
 
 10
 
 30933
 
 island
 
 0.676668
 
 0.0183347
 
 NKX3-2; AC006445.8 (margin: NKX3-2; AC006445.8)
 
  7.2440252
 
 3.25e-04
 
 4.91e-03
  
 
  
 11
 
   6440690
 
   6440968
 
  PDF   STATS   ENSEMBL   UCSC 
 
  279
 
 10
 
 7110
 
 tss;island
 
 0.578195
 
 0.0180453
 
 APBB1 (margin: APBB1; HPX)
 
  7.2186643
 
 3.25e-04
 
 4.91e-03
  
 
  
 5
 
 128300967
 
 128301488
 
  PDF   STATS   ENSEMBL   UCSC 
 
  522
 
 10
 
 33072
 
 island
 
 0.358096
 
 0.0129529
 
 SLC27A6 (margin: SLC27A6)
 
  5.6394285
 
 3.25e-04
 
 4.91e-03
  
 
  
 5
 
  59189375
 
  59189934
 
  PDF   STATS   ENSEMBL   UCSC 
 
  560
 
 10
 
 31625
 
 gene;island
 
 0.726217
 
 0.0693140
 
 PDE4D (margin: PDE4D)
 
  5.2274101
 
 3.25e-04
 
 4.91e-03
  
 
  
 6
 
  17282284
 
  17282859
 
  PDF   STATS   ENSEMBL   UCSC 
 
  576
 
 10
 
 33587
 
 gene;tss;island
 
 0.903507
 
 0.4877842
 
 RBM24 (margin: RBM24)
 
  4.8911582
 
 3.25e-04
 
 4.91e-03
  
 
  
 19
 
  22816896
 
  22817496
 
  PDF   STATS   ENSEMBL   UCSC 
 
  601
 
 10
 
 21843
 
 island
 
 0.484423
 
 0.0182757
 
 ZNF492 (margin: ZNF492; AC011516.2)
 
  4.1996294
 
 3.25e-04
 
 4.91e-03
  
 
  
 8
 
  65492528
 
  65492936
 
  PDF   STATS   ENSEMBL   UCSC 
 
  409
 
 10
 
 40347
 
 island
 
 0.175875
 
 0.7389047
 
 BHLHE22; RP11-21C4.1 (margin: BHLHE22; RP11-21C4.5; RP11-21C4.1; CYP7B1)
 
  3.7064367
 
 3.25e-04
 
 4.91e-03
  
 
  
 11
 
 120110544
 
 120111025
 
  PDF   STATS   ENSEMBL   UCSC 
 
  482
 
 10
 
 8893
 
 island
 
 0.508810
 
 0.9148430
 
 POU2F3 (margin: OAF; POU2F3; RP11-778O17.4)
 
  3.4062796
 
 3.25e-04
 
 4.91e-03
  
 
  
 19
 
  40005557
 
  40005836
 
  PDF   STATS   ENSEMBL   UCSC 
 
  280
 
 10
 
 22001
 
 island
 
 0.532278
 
 0.8534067
 
 SELV (margin: DLL3; SELV)
 
  3.2496973
 
 3.25e-04
 
 4.91e-03
  
 
  
 11
 
 107798913
 
 107799287
 
  PDF   STATS   ENSEMBL   UCSC 
 
  375
 
 10
 
 8777
 
 island
 
 0.304828
 
 0.0265297
 
 RAB39A; SLC35F2 (margin: RAB39A; SLC35F2)
 
  3.1218716
 
 3.25e-04
 
 4.91e-03
  
 
  
 6
 
  29394438
 
  29395425
 
  PDF   STATS   ENSEMBL   UCSC 
 
  988
 
 12
 
 33655
 
 gene
 
 0.083188
 
 0.7047189
 
 OR5V1; OR11A1 (margin: OR12D1P; OR10C1; OR5V1; OR11A1)
 
  4.1034908
 
 3.71e-04
 
 5.59e-03
  
 
  
 1
 
  78511140
 
  78512129
 
  PDF   STATS   ENSEMBL   UCSC 
 
  990
 
 12
 
 3402
 
 island
 
 0.860338
 
 0.9805205
 
 GIPC2; RP11-386I14.2 (margin: GIPC2; RP11-386I14.2)
 
  2.6635935
 
 3.71e-04
 
 5.59e-03
  
 
  
 4
 
 187476326
 
 187477065
 
  PDF   STATS   ENSEMBL   UCSC 
 
  740
 
 11
 
 31483
 
 island
 
 0.810564
 
 0.0103418
 
 RP11-215A19.2; MTNR1A (margin: RP11-215A19.2; MTNR1A)
 
  8.4808711
 
 3.94e-04
 
 5.86e-03
  
 
  
 19
 
  19280969
 
  19281559
 
  PDF   STATS   ENSEMBL   UCSC 
 
  591
 
 11
 
 19947
 
 gene;island
 
 0.518657
 
 0.0120118
 
 MEF2BNB-MEF2B; MEF2B (margin: MEF2BNB-MEF2B; MEF2B; MEF2BNB)
 
  6.6398701
 
 3.94e-04
 
 5.86e-03
  
 
  
 19
 
  39522418
 
  39523626
 
  PDF   STATS   ENSEMBL   UCSC 
 
 1209
 
 11
 
 21986
 
 island
 
 0.494865
 
 0.0061657
 
 CTB-189B5.3; FBXO27 (margin: CTB-189B5.3; FBXO27)
 
  6.6163996
 
 3.94e-04
 
 5.86e-03
  
 
  
 2
 
  27805536
 
  27806117
 
  PDF   STATS   ENSEMBL   UCSC 
 
  582
 
 11
 
 24189
 
 island
 
 0.012305
 
 0.3797408
 
 C2orf16; ZNF512; RP11-158I13.2; AC074091.1 (margin: C2orf16; ZNF512; RP11-158I13.2; AC074091.1)
 
  5.8190802
 
 3.94e-04
 
 5.86e-03
  
 
  
 17
 
  40713676
 
  40714406
 
  PDF   STATS   ENSEMBL   UCSC 
 
  731
 
 11
 
 18340
 
 island
 
 0.019914
 
 0.3739203
 
 RP11-400F19.8; COASY (margin: RP11-400F19.8; HSD17B1; COASY; MLX; RP11-400F19.6; PSMC3IP)
 
  4.9115169
 
 3.94e-04
 
 5.86e-03
  
 
  
 4
 
 141347993
 
 141349096
 
  PDF   STATS   ENSEMBL   UCSC 
 
 1104
 
 16
 
 31325
 
 island
 
 0.175649
 
 0.0060703
 
 CLGN (margin: CLGN)
 
  4.6789194
 
 3.92e-04
 
 5.86e-03
  
 
  
 10
 
 105212236
 
 105212813
 
  PDF   STATS   ENSEMBL   UCSC 
 
  578
 
 11
 
 5338
 
 tss
 
 0.609000
 
 0.3479701
 
 RP11-225H22.4; CALHM2; CALHM1 (margin: PDCD11; RP11-225H22.4; CALHM2; CALHM1)
 
  3.7175163
 
 3.94e-04
 
 5.86e-03
  
 
  
 6
 
  32728786
 
  32730299
 
  PDF   STATS   ENSEMBL   UCSC 
 
 1514
 
 40
 
 34132
 
 gene;island
 
 0.744104
 
 0.9434472
 
 HLA-DQB2 (margin: MIR3135B; HLA-DQB2)
 
  2.4915564
 
 3.94e-04
 
 5.86e-03
  
 
  
 6
 
 122931154
 
 122931702
 
  PDF   STATS   ENSEMBL   UCSC 
 
  549
 
 11
 
 34463
 
 gene;island
 
 0.073470
 
 0.0198058
 
 PKIB (margin: PKIB)
 
  1.9842102
 
 3.94e-04
 
 5.86e-03
  
 
  
 7
 
  27153580
 
  27153944
 
  PDF   STATS   ENSEMBL   UCSC 
 
  365
 
  7
 
 36930
 
 gene;island
 
 0.003833
 
 0.9842564
 
 HOXA-AS2; HOXA3 (margin: HOXA-AS2; HOXA2; HOXA3)
 
 15.0336902
 
 5.83e-04
 
 6.47e-03
  
 
  
 11
 
 121986961
 
 121987203
 
  PDF   STATS   ENSEMBL   UCSC 
 
  243
 
  7
 
 6948
 
 gene;tss
 
 0.981970
 
 0.0123627
 
 RP11-166D19.1; BLID (margin: RP11-166D19.1; BLID)
 
 13.0480625
 
 5.83e-04
 
 6.47e-03
  
 
  
 7
 
  27196296
 
  27196555
 
  PDF   STATS   ENSEMBL   UCSC 
 
  260
 
  7
 
 37494
 
 tss
 
 0.005620
 
 0.9792464
 
 HOXA-AS3; RP1-170O19.21; HOXA7 (margin: HOXA-AS3; RP1-170O19.21; HOXA-AS4; HOXA3; HOXA6; HOXA7; HOXA9; RP1-170O19.20)
 
 13.0324006
 
 5.83e-04
 
 6.47e-03
  
 
  
 16
 
  56659336
 
  56659724
 
  PDF   STATS   ENSEMBL   UCSC 
 
  389
 
  7
 
 15949
 
 island
 
 0.007345
 
 0.9700851
 
 MT1E (margin: MT1L; MT1E; MT1M; MT1JP; AC026461.1)
 
 12.9327921
 
 5.83e-04
 
 6.47e-03
  
 
  
 11
 
  33037397
 
  33037916
 
  PDF   STATS   ENSEMBL   UCSC 
 
  520
 
  7
 
 8185
 
 island
 
 0.011832
 
 0.9785054
 
 DEPDC7 (margin: DEPDC7; Y_RNA)
 
 12.8015680
 
 5.83e-04
 
 6.47e-03
  
 
  
 4
 
 176987009
 
 176987577
 
  PDF   STATS   ENSEMBL   UCSC 
 
  569
 
  7
 
 30186
 
 gene
 
 0.740243
 
 0.0047212
 
 WDR17 (margin: WDR17)
 
 12.7896861
 
 5.83e-04
 
 6.47e-03
  
 
  
 19
 
   9649310
 
   9649542
 
  PDF   STATS   ENSEMBL   UCSC 
 
  233
 
  7
 
 20426
 
 tss
 
 0.008980
 
 0.9659673
 
 ZNF426 (margin: ZNF426)
 
 12.3784585
 
 5.83e-04
 
 6.47e-03
  
 
  
 11
 
   6341842
 
   6341908
 
  PDF   STATS   ENSEMBL   UCSC 
 
   67
 
  7
 
 7106
 
 tss
 
 0.016418
 
 0.9810317
 
 PRKCDBP (margin: PRKCDBP)
 
 11.8097341
 
 5.83e-04
 
 6.47e-03
  
 
  
 20
 
  57581903
 
  57582371
 
  PDF   STATS   ENSEMBL   UCSC 
 
  469
 
  7
 
 26299
 
 island
 
 0.010674
 
 0.9824445
 
 CTSZ (margin: NELFCD; TUBB1; CTSZ)
 
 11.8027803
 
 5.83e-04
 
 6.47e-03
  
 
  
 15
 
  79382548
 
  79383167
 
  PDF   STATS   ENSEMBL   UCSC 
 
  620
 
  7
 
 13447
 
 gene;island
 
 0.944524
 
 0.0092832
 
 RASGRF1 (margin: RASGRF1)
 
 11.6980490
 
 5.83e-04
 
 6.47e-03
  
 
  
 4
 
  44728642
 
  44728857
 
  PDF   STATS   ENSEMBL   UCSC 
 
  216
 
  7
 
 30370
 
 tss
 
 0.002233
 
 0.5601860
 
 GNPDA2 (margin: GNPDA2)
 
 11.3929658
 
 5.83e-04
 
 6.47e-03
  
 
  
 2
 
 220117599
 
 220117945
 
  PDF   STATS   ENSEMBL   UCSC 
 
  347
 
  7
 
 23029
 
 gene;tss;island
 
 0.010294
 
 0.9769421
 
 TUBA4B; TUBA4A (margin: STK16; TUBA4B; GLB1L; TUBA4A)
 
 11.3660712
 
 5.83e-04
 
 6.47e-03
  
 
  
 1
 
 209825672
 
 209825943
 
  PDF   STATS   ENSEMBL   UCSC 
 
  272
 
  7
 
 2230
 
 tss
 
 0.004107
 
 0.8964726
 
 LAMB3 (margin: LAMB3; RP1-28O10.1)
 
 11.2604158
 
 5.83e-04
 
 6.47e-03
  
 
  
 3
 
 192445388
 
 192445594
 
  PDF   STATS   ENSEMBL   UCSC 
 
  207
 
  7
 
 28792
 
 tss
 
 0.916534
 
 0.0065870
 
 FGF12; RNU1-20P (margin: FGF12; RNU1-20P)
 
 11.2183804
 
 5.83e-04
 
 6.47e-03
  
 
  
 2
 
  71503484
 
  71503883
 
  PDF   STATS   ENSEMBL   UCSC 
 
  400
 
  7
 
 24396
 
 island
 
 0.788977
 
 0.0053147
 
 ZNF638 (margin: ZNF638)
 
 11.1444020
 
 5.83e-04
 
 6.47e-03
  
 
  
 17
 
  49008322
 
  49008925
 
  PDF   STATS   ENSEMBL   UCSC 
 
  604
 
  7
 
 18563
 
 island
 
 0.018188
 
 0.9641402
 
  (margin: RP11-700H6.1)
 
 11.0937572
 
 5.83e-04
 
 6.47e-03
  
 
  
 12
 
  49393166
 
  49393408
 
  PDF   STATS   ENSEMBL   UCSC 
 
  243
 
  7
 
 9680
 
 tss
 
 0.021603
 
 0.9843356
 
 RP11-386G11.5; DDN (margin: RP11-386G11.3; RP11-386G11.5; DDN; PRKAG1)
 
 11.0869903
 
 5.83e-04
 
 6.47e-03
  
 
  
 7
 
  87257356
 
  87257786
 
  PDF   STATS   ENSEMBL   UCSC 
 
  431
 
  7
 
 37073
 
 gene;island
 
 0.668913
 
 0.0036127
 
 RUNDC3B; ABCB1 (margin: snoU13; RUNDC3B; ABCB1)
 
 10.9893948
 
 5.83e-04
 
 6.47e-03
  
 
  
 13
 
  67804509
 
  67804744
 
  PDF   STATS   ENSEMBL   UCSC 
 
  236
 
  7
 
 11471
 
 tss
 
 0.794527
 
 0.0048720
 
 PCDH9 (margin: PCDH9)
 
 10.7605078
 
 5.83e-04
 
 6.47e-03
  
 
  
 3
 
 149374761
 
 149375293
 
  PDF   STATS   ENSEMBL   UCSC 
 
  533
 
  7
 
 27992
 
 gene;island
 
 0.007860
 
 0.9732876
 
 WWTR1-AS1; WWTR1 (margin: WWTR1-AS1; WWTR1; WWTR1-IT1)
 
 10.6266910
 
 5.83e-04
 
 6.47e-03
  
 
  
 1
 
  32041507
 
  32042037
 
  PDF   STATS   ENSEMBL   UCSC 
 
  531
 
  7
 
 1448
 
 tss
 
 0.009848
 
 0.9489900
 
 TINAGL1; RP11-73M7.1 (margin: TINAGL1; RP11-73M7.1)
 
 10.5902161
 
 5.83e-04
 
 6.47e-03
  
 
  
 20
 
  13976093
 
  13976190
 
  PDF   STATS   ENSEMBL   UCSC 
 
   98
 
  7
 
 26012
 
 island
 
 0.855699
 
 0.0046728
 
 MACROD2; SEL1L2 (margin: MACROD2; RPS3P1; SEL1L2)
 
 10.5586491
 
 5.83e-04
 
 6.47e-03
  
 
  
 20
 
  48099248
 
  48099479
 
  PDF   STATS   ENSEMBL   UCSC 
 
  232
 
  7
 
 25808
 
 tss
 
 0.898502
 
 0.0102120
 
 KCNB1 (margin: KCNB1)
 
 10.5221884
 
 5.83e-04
 
 6.47e-03
  
 
  
 11
 
  22851383
 
  22851591
 
  PDF   STATS   ENSEMBL   UCSC 
 
  209
 
  7
 
 7204
 
 tss
 
 0.933412
 
 0.0042923
 
 RP11-17A1.3; SVIP (margin: RP11-17A1.3; SVIP)
 
 10.5022141
 
 5.83e-04
 
 6.47e-03
  
 
  
 15
 
  39871808
 
  39872186
 
  PDF   STATS   ENSEMBL   UCSC 
 
  379
 
  7
 
 13576
 
 tss;island
 
 0.003617
 
 0.7705225
 
 THBS1 (margin: THBS1)
 
 10.3728438
 
 5.83e-04
 
 6.47e-03
  
 
  
 8
 
  10586678
 
  10587055
 
  PDF   STATS   ENSEMBL   UCSC 
 
  378
 
  7
 
 39199
 
 gene;island
 
 0.011931
 
 0.9667253
 
 CTD-2135J3.3; SOX7 (margin: CTD-2135J3.3; SOX7)
 
 10.3615807
 
 5.83e-04
 
 6.47e-03
  
 
  
 10
 
 118032872
 
 118033115
 
  PDF   STATS   ENSEMBL   UCSC 
 
  244
 
  7
 
 4774
 
 gene
 
 0.941245
 
 0.0060861
 
 GFRA1 (margin: GFRA1)
 
 10.2585551
 
 5.83e-04
 
 6.47e-03
  
 
  
 19
 
  59066570
 
  59066700
 
  PDF   STATS   ENSEMBL   UCSC 
 
  131
 
  7
 
 21150
 
 tss;island
 
 0.002624
 
 0.5830275
 
 CHMP2A; UBE2M (margin: TRIM28; AC016629.8; RN7SL525P; CHMP2A; UBE2M; MZF1)
 
 10.2214645
 
 5.83e-04
 
 6.47e-03
  
 
  
 11
 
  66103823
 
  66104174
 
  PDF   STATS   ENSEMBL   UCSC 
 
  352
 
  7
 
 8509
 
 island
 
 0.004602
 
 0.8303603
 
 RP11-867G23.12; RIN1; BRMS1 (margin: RP11-867G23.12; RP11-867G23.8; RIN1; BRMS1; B3GNT1)
 
 10.1333799
 
 5.83e-04
 
 6.47e-03
  
 
  
 1
 
  85527430
 
  85527943
 
  PDF   STATS   ENSEMBL   UCSC 
 
  514
 
  7
 
 1721
 
 tss
 
 0.008497
 
 0.9756858
 
 WDR63 (margin: WDR63)
 
 10.1293670
 
 5.83e-04
 
 6.47e-03
  
 
  
 19
 
  11998686
 
  11999148
 
  PDF   STATS   ENSEMBL   UCSC 
 
  463
 
  7
 
 19850
 
 gene
 
 0.088674
 
 0.9562620
 
 ZNF69 (margin: ZNF439; ZNF69)
 
  9.9245531
 
 5.83e-04
 
 6.47e-03
  
 
  
 11
 
 117069780
 
 117069966
 
  PDF   STATS   ENSEMBL   UCSC 
 
  187
 
  7
 
 7717
 
 tss
 
 0.028597
 
 0.9608695
 
 SIDT2; TAGLN (margin: SIDT2; TAGLN; PCSK7)
 
  9.8321031
 
 5.83e-04
 
 6.47e-03
  
 
  
 1
 
 204183337
 
 204183655
 
  PDF   STATS   ENSEMBL   UCSC 
 
  319
 
  7
 
 2197
 
 tss
 
 0.954737
 
 0.0111833
 
 GOLT1A (margin: GOLT1A; PLEKHA6)
 
  9.7417746
 
 5.83e-04
 
 6.47e-03
  
 
  
 1
 
  43613433
 
  43613627
 
  PDF   STATS   ENSEMBL   UCSC 
 
  195
 
  7
 
 3158
 
 island
 
 0.913609
 
 0.0124518
 
 FAM183A (margin: FAM183A)
 
  9.7152611
 
 5.83e-04
 
 6.47e-03
  
 
  
 19
 
  52430190
 
  52430632
 
  PDF   STATS   ENSEMBL   UCSC 
 
  443
 
  7
 
 21001
 
 tss
 
 0.001913
 
 0.4802642
 
 ZNF613 (margin: ZNF613)
 
  9.6987560
 
 5.83e-04
 
 6.47e-03
  
 
  
 19
 
   1132294
 
   1132537
 
  PDF   STATS   ENSEMBL   UCSC 
 
  244
 
  7
 
 20293
 
 tss
 
 0.021376
 
 0.9821913
 
 SBNO2 (margin: SBNO2)
 
  9.6633528
 
 5.83e-04
 
 6.47e-03
  
 
  
 7
 
 138794512
 
 138794752
 
  PDF   STATS   ENSEMBL   UCSC 
 
  241
 
  7
 
 37928
 
 tss
 
 0.005235
 
 0.6976922
 
 ZC3HAV1 (margin: ZC3HAV1)
 
  9.4073460
 
 5.83e-04
 
 6.47e-03
  
 
  
 22
 
  46481603
 
  46481822
 
  PDF   STATS   ENSEMBL   UCSC 
 
  220
 
  7
 
 27228
 
 tss
 
 0.025801
 
 0.9619560
 
 FLJ27365 (margin: FLJ27365; MIR3619)
 
  9.3641317
 
 5.83e-04
 
 6.47e-03
  
 
  
 11
 
  34535357
 
  34535579
 
  PDF   STATS   ENSEMBL   UCSC 
 
  223
 
  7
 
 7242
 
 tss
 
 0.959613
 
 0.0377423
 
 ELF5 (margin: ELF5)
 
  9.2439790
 
 5.83e-04
 
 6.47e-03
  
 
  
 6
 
  28129313
 
  28129498
 
  PDF   STATS   ENSEMBL   UCSC 
 
  186
 
  7
 
 34734
 
 tss
 
 0.038744
 
 0.8628174
 
 ZNF192P1 (margin: ZKSCAN8; ZNF192P1)
 
  9.0910603
 
 5.83e-04
 
 6.47e-03
  
 
  
 4
 
  37455280
 
  37455499
 
  PDF   STATS   ENSEMBL   UCSC 
 
  220
 
  7
 
 30341
 
 tss;island
 
 0.877526
 
 0.0260667
 
 C4orf19 (margin: KIAA1239; C4orf19)
 
  9.0779084
 
 5.83e-04
 
 6.47e-03
  
 
  
 19
 
  37701476
 
  37701738
 
  PDF   STATS   ENSEMBL   UCSC 
 
  263
 
  7
 
 20735
 
 tss
 
 0.384546
 
 0.0026787
 
 CTC-454I21.3; ZNF585B (margin: ZNF383; CTC-454I21.3; ZNF585B)
 
  9.0220682
 
 5.83e-04
 
 6.47e-03
  
 
  
 6
 
  29855110
 
  29855347
 
  PDF   STATS   ENSEMBL   UCSC 
 
  238
 
  7
 
 34756
 
 tss
 
 0.072403
 
 0.9662191
 
 HLA-H; HCG4P7 (margin: HLA-H; HLA-T; HCG4P7)
 
  8.9793998
 
 5.83e-04
 
 6.47e-03
  
 
  
 19
 
  20150159
 
  20150505
 
  PDF   STATS   ENSEMBL   UCSC 
 
  347
 
  7
 
 20655
 
 tss;island
 
 0.665355
 
 0.0040905
 
 ZNF682 (margin: AC006539.3; ZNF682)
 
  8.9612124
 
 5.83e-04
 
 6.47e-03
  
 
  
 1
 
 155043322
 
 155043967
 
  PDF   STATS   ENSEMBL   UCSC 
 
  646
 
  7
 
 3806
 
 island
 
 0.031025
 
 0.9805229
 
 EFNA4; EFNA3 (margin: ADAM15; EFNA4; EFNA3)
 
  8.8577595
 
 5.83e-04
 
 6.47e-03
  
 
  
 2
 
  11679845
 
  11680144
 
  PDF   STATS   ENSEMBL   UCSC 
 
  300
 
  7
 
 22571
 
 gene
 
 0.588034
 
 0.0285357
 
 GREB1; MIR4429 (margin: GREB1; MIR4429)
 
  8.8458014
 
 5.83e-04
 
 6.47e-03
  
 
  
 17
 
   9479910
 
   9479965
 
  PDF   STATS   ENSEMBL   UCSC 
 
   56
 
  7
 
 17127
 
 tss;island
 
 0.387108
 
 0.0037007
 
 WDR16; STX8 (margin: WDR16; STX8)
 
  8.6975227
 
 5.83e-04
 
 6.47e-03
  
 
  
 3
 
 139258700
 
 139258948
 
  PDF   STATS   ENSEMBL   UCSC 
 
  249
 
  7
 
 28633
 
 tss
 
 0.093914
 
 0.9743836
 
 RP11-319G6.1; RBP1 (margin: RP11-319G6.1; RBP1)
 
  8.6524239
 
 5.83e-04
 
 6.47e-03
  
 
  
 16
 
  69141250
 
  69141478
 
  PDF   STATS   ENSEMBL   UCSC 
 
  229
 
  7
 
 14817
 
 gene;island
 
 0.181953
 
 0.9843593
 
 HAS3 (margin: HAS3; CHTF8)
 
  8.5378020
 
 5.83e-04
 
 6.47e-03
  
 
  
 22
 
  45596948
 
  45597035
 
  PDF   STATS   ENSEMBL   UCSC 
 
   88
 
  7
 
 27218
 
 tss
 
 0.140214
 
 0.9843147
 
 KIAA0930; MIR1249 (margin: KIAA0930; MIR1249)
 
  8.5346014
 
 5.83e-04
 
 6.47e-03
  
 
  
 12
 
 107714673
 
 107715146
 
  PDF   STATS   ENSEMBL   UCSC 
 
  474
 
  7
 
 9283
 
 gene;island
 
 0.076962
 
 0.9641064
 
 BTBD11 (margin: BTBD11)
 
  8.4468873
 
 5.83e-04
 
 6.47e-03
  
 
  
 13
 
  78272372
 
  78272639
 
  PDF   STATS   ENSEMBL   UCSC 
 
  268
 
  7
 
 11789
 
 island
 
 0.656925
 
 0.0072077
 
 SLAIN1; MIR3665 (margin: SLAIN1; MIR3665)
 
  8.4226226
 
 5.83e-04
 
 6.47e-03
  
 
  
 1
 
  55267006
 
  55267293
 
  PDF   STATS   ENSEMBL   UCSC 
 
  288
 
  7
 
 1648
 
 tss
 
 0.009138
 
 0.8571750
 
 TTC22 (margin: RP11-67L3.2; C1orf177; TTC22)
 
  8.3688052
 
 5.83e-04
 
 6.47e-03
  
 
  
 14
 
  61116048
 
  61116506
 
  PDF   STATS   ENSEMBL   UCSC 
 
  459
 
  7
 
 12907
 
 island
 
 0.004193
 
 0.5483567
 
 SIX1 (margin: RP11-1042B17.5; SIX1)
 
  8.3524061
 
 5.83e-04
 
 6.47e-03
  
 
  
 10
 
 128994297
 
 128994702
 
  PDF   STATS   ENSEMBL   UCSC 
 
  406
 
  7
 
 4818
 
 gene;island
 
 0.736439
 
 0.0082290
 
 DOCK1; FAM196A (margin: DOCK1; FAM196A)
 
  8.3448385
 
 5.83e-04
 
 6.47e-03
  
 
  
 18
 
  55862577
 
  55862872
 
  PDF   STATS   ENSEMBL   UCSC 
 
  296
 
  7
 
 19120
 
 gene;island
 
 0.115909
 
 0.9769799
 
 NEDD4L; RP11-718I15.1 (margin: NEDD4L; RP11-718I15.1)
 
  8.3079484
 
 5.83e-04
 
 6.47e-03
  
 
  
 12
 
   6560689
 
   6561007
 
  PDF   STATS   ENSEMBL   UCSC 
 
  319
 
  7
 
 9511
 
 tss
 
 0.015785
 
 0.8980304
 
 CD27; TAPBPL; CD27-AS1 (margin: CD27; TAPBPL; CD27-AS1; VAMP1)
 
  8.2697920
 
 5.83e-04
 
 6.47e-03
  
 
  
 19
 
  37569236
 
  37569352
 
  PDF   STATS   ENSEMBL   UCSC 
 
  117
 
  7
 
 20733
 
 tss
 
 0.802375
 
 0.0068591
 
 ZNF420; CTD-2293H3.2 (margin: ZNF420; CTD-2293H3.2)
 
  8.2317568
 
 5.83e-04
 
 6.47e-03
  
 
  
 4
 
   8582101
 
   8582300
 
  PDF   STATS   ENSEMBL   UCSC 
 
  200
 
  7
 
 30922
 
 island
 
 0.657911
 
 0.0104737
 
 GPR78 (margin: GPR78)
 
  8.1325935
 
 5.83e-04
 
 6.47e-03
  
 
  
 15
 
  89438611
 
  89438964
 
  PDF   STATS   ENSEMBL   UCSC 
 
  354
 
  7
 
 14423
 
 island
 
 0.107684
 
 0.9656085
 
 HAPLN3 (margin: HAPLN3; MFGE8)
 
  8.1295556
 
 5.83e-04
 
 6.47e-03
  
 
  
 19
 
  35645556
 
  35645712
 
  PDF   STATS   ENSEMBL   UCSC 
 
  157
 
  7
 
 20701
 
 tss;island
 
 0.002698
 
 0.5944597
 
 FXYD7; FXYD5 (margin: FXYD1; CTD-2527I21.4; FXYD7; FXYD5)
 
  8.1270656
 
 5.83e-04
 
 6.47e-03
  
 
  
 19
 
  50706387
 
  50706874
 
  PDF   STATS   ENSEMBL   UCSC 
 
  488
 
  7
 
 20973
 
 tss;island
 
 0.918338
 
 0.0235255
 
 MYH14 (margin: MYH14)
 
  8.0324892
 
 5.83e-04
 
 6.47e-03
  
 
  
 1
 
   3607097
 
   3607425
 
  PDF   STATS   ENSEMBL   UCSC 
 
  329
 
  7
 
 138
 
 gene;island
 
 0.130280
 
 0.9794866
 
 TP73 (margin: TP73)
 
  8.0087000
 
 5.83e-04
 
 6.47e-03
  
 
  
 2
 
  97760606
 
  97760749
 
  PDF   STATS   ENSEMBL   UCSC 
 
  144
 
  7
 
 23527
 
 tss;island
 
 0.008109
 
 0.5888977
 
 FAHD2B (margin: AC018892.9; RN7SL313P; FAHD2B)
 
  8.0060616
 
 5.83e-04
 
 6.47e-03
  
 
  
 16
 
  81526954
 
  81527504
 
  PDF   STATS   ENSEMBL   UCSC 
 
  551
 
  7
 
 14854
 
 gene
 
 0.016507
 
 0.8682323
 
 CMIP (margin: CMIP)
 
  7.8861101
 
 5.83e-04
 
 6.47e-03
  
 
  
 5
 
  57878188
 
  57878526
 
  PDF   STATS   ENSEMBL   UCSC 
 
  339
 
  7
 
 32077
 
 tss;island
 
 0.785558
 
 0.0127281
 
 RAB3C (margin: RAB3C)
 
  7.8540139
 
 5.83e-04
 
 6.47e-03
  
 
  
 22
 
  30901532
 
  30901886
 
  PDF   STATS   ENSEMBL   UCSC 
 
  355
 
  7
 
 27413
 
 island
 
 0.052521
 
 0.9162924
 
 SEC14L4 (margin: SEC14L4)
 
  7.8047902
 
 5.83e-04
 
 6.47e-03
  
 
  
 13
 
  37006063
 
  37006340
 
  PDF   STATS   ENSEMBL   UCSC 
 
  278
 
  7
 
 11132
 
 gene;tss;island
 
 0.785718
 
 0.0197204
 
 CCNA1 (margin: CCNA1)
 
  7.8000398
 
 5.83e-04
 
 6.47e-03
  
 
  
 2
 
 214148856
 
 214149035
 
  PDF   STATS   ENSEMBL   UCSC 
 
  180
 
  7
 
 23859
 
 tss
 
 0.240302
 
 0.9764423
 
 SPAG16; AC079610.2 (margin: SPAG16; RP11-105N14.2; AC079610.2)
 
  7.7491975
 
 5.83e-04
 
 6.47e-03
  
 
  
 11
 
 119993722
 
 119994008
 
  PDF   STATS   ENSEMBL   UCSC 
 
  287
 
  7
 
 6942
 
 gene
 
 0.052219
 
 0.9437517
 
 TRIM29 (margin: TRIM29)
 
  7.7054383
 
 5.83e-04
 
 6.47e-03
  
 
  
 14
 
  37051693
 
  37051949
 
  PDF   STATS   ENSEMBL   UCSC 
 
  257
 
  7
 
 12787
 
 island
 
 0.006821
 
 0.5584706
 
 NKX2-8 (margin: RPL29P3; NKX2-8; RN7SKP257)
 
  7.6753456
 
 5.83e-04
 
 6.47e-03
  
 
  
 4
 
  96470053
 
  96470349
 
  PDF   STATS   ENSEMBL   UCSC 
 
  297
 
  7
 
 31191
 
 island
 
 0.640816
 
 0.0078895
 
 RP11-710C12.1; UNC5C (margin: RP11-710C12.1; UNC5C)
 
  7.5664401
 
 5.83e-04
 
 6.47e-03
  
 
  
 10
 
  52833902
 
  52834189
 
  PDF   STATS   ENSEMBL   UCSC 
 
  288
 
  7
 
 4590
 
 gene;tss;island
 
 0.777780
 
 0.0138165
 
 PRKG1 (margin: PRKG1; RP11-96B5.3)
 
  7.5083656
 
 5.83e-04
 
 6.47e-03
  
 
  
 11
 
  63439115
 
  63439313
 
  PDF   STATS   ENSEMBL   UCSC 
 
  199
 
  7
 
 7377
 
 tss
 
 0.805798
 
 0.0158978
 
 ATL3 (margin: RTN3; ATL3)
 
  7.4969048
 
 5.83e-04
 
 6.47e-03
  
 
  
 3
 
 158390510
 
 158390821
 
  PDF   STATS   ENSEMBL   UCSC 
 
  312
 
  7
 
 28686
 
 tss
 
 0.967124
 
 0.0302078
 
 GFM1; LXN (margin: GFM1; LXN)
 
  7.4891964
 
 5.83e-04
 
 6.47e-03
  
 
  
 5
 
 156886816
 
 156887129
 
  PDF   STATS   ENSEMBL   UCSC 
 
  314
 
  7
 
 33342
 
 island
 
 0.151132
 
 0.9337445
 
 NIPAL4; CTB-109A12.1; ADAM19 (margin: NIPAL4; CTB-109A12.1; ADAM19)
 
  7.4723943
 
 5.83e-04
 
 6.47e-03
  
 
  
 10
 
 135340445
 
 135340850
 
  PDF   STATS   ENSEMBL   UCSC 
 
  406
 
  7
 
 5465
 
 tss
 
 0.041826
 
 0.8924642
 
 CYP2E1; SPRN; AL161645.2 (margin: RP11-108K14.4; CYP2E1; SPRN; AL161645.2)
 
  7.3897338
 
 5.83e-04
 
 6.47e-03
  
 
  
 10
 
 134901199
 
 134901305
 
  PDF   STATS   ENSEMBL   UCSC 
 
  107
 
  7
 
 5452
 
 tss
 
 0.867132
 
 0.0420193
 
 GPR123; RP13-439H18.4; AL445199.1 (margin: GPR123; RP13-439H18.4; AL445199.1)
 
  7.3514472
 
 5.83e-04
 
 6.47e-03
  
 
  
 10
 
  63422511
 
  63422668
 
  PDF   STATS   ENSEMBL   UCSC 
 
  158
 
  7
 
 5098
 
 tss
 
 0.667549
 
 0.0267022
 
 C10orf107 (margin: C10orf107)
 
  7.3027335
 
 5.83e-04
 
 6.47e-03
  
 
  
 10
 
  44880542
 
  44880819
 
  PDF   STATS   ENSEMBL   UCSC 
 
  278
 
  7
 
 5055
 
 tss;island
 
 0.814968
 
 0.0280983
 
 AL137026.1; CXCL12 (margin: AL137026.1; CXCL12)
 
  7.2514288
 
 5.83e-04
 
 6.47e-03
  
 
  
 19
 
  12175107
 
  12175540
 
  PDF   STATS   ENSEMBL   UCSC 
 
  434
 
  7
 
 20488
 
 tss
 
 0.174627
 
 0.9338730
 
 ZNF844 (margin: ZNF844; RNA5SP466; CTD-2006C1.10; ZNF878)
 
  7.2491389
 
 5.83e-04
 
 6.47e-03
  
 
  
 19
 
  20162642
 
  20163138
 
  PDF   STATS   ENSEMBL   UCSC 
 
  497
 
  7
 
 21833
 
 island
 
 0.311244
 
 0.0039436
 
  (margin: AC006539.4)
 
  7.2142210
 
 5.83e-04
 
 6.47e-03
  
 
  
 10
 
  94833520
 
  94833658
 
  PDF   STATS   ENSEMBL   UCSC 
 
  139
 
  7
 
 4680
 
 gene;island
 
 0.469482
 
 0.0072777
 
 CYP26A1 (margin: CYP26C1; CYP26A1)
 
  7.1446747
 
 5.83e-04
 
 6.47e-03
  
 
  
 5
 
  87971794
 
  87972265
 
  PDF   STATS   ENSEMBL   UCSC 
 
  472
 
  7
 
 31679
 
 gene;island
 
 0.459419
 
 0.0080968
 
 CTC-467M3.1; LINC00461 (margin: CTC-467M3.1; LINC00461)
 
  7.1035229
 
 5.83e-04
 
 6.47e-03
  
 
  
 3
 
 187457128
 
 187457732
 
  PDF   STATS   ENSEMBL   UCSC 
 
  605
 
  7
 
 28079
 
 gene;island
 
 0.087885
 
 0.8679600
 
 BCL6 (margin: RP11-211G3.3; RP11-211G3.2; BCL6)
 
  7.0654315
 
 5.83e-04
 
 6.47e-03
  
 
  
 4
 
 164264796
 
 164265012
 
  PDF   STATS   ENSEMBL   UCSC 
 
  217
 
  7
 
 30661
 
 tss;island
 
 0.680610
 
 0.0225595
 
 NPY5R; NPY1R (margin: NPY5R; NPY1R)
 
  7.0477065
 
 5.83e-04
 
 6.47e-03
  
 
  
 4
 
  48491941
 
  48492304
 
  PDF   STATS   ENSEMBL   UCSC 
 
  364
 
  7
 
 30383
 
 tss
 
 0.172506
 
 0.9464711
 
 SLC10A4; ZAR1 (margin: SLC10A4; ZAR1; FRYL)
 
  7.0423123
 
 5.83e-04
 
 6.47e-03
  
 
  
 10
 
  73533407
 
  73533561
 
  PDF   STATS   ENSEMBL   UCSC 
 
  155
 
  7
 
 5145
 
 tss
 
 0.008946
 
 0.5733102
 
 CDH23; C10orf54 (margin: CDH23; C10orf54)
 
  6.9799622
 
 5.83e-04
 
 6.47e-03
  
 
  
 4
 
  94749865
 
  94750205
 
  PDF   STATS   ENSEMBL   UCSC 
 
  341
 
  7
 
 31186
 
 island
 
 0.541368
 
 0.0095737
 
 ATOH1 (margin: RNA5SP164; ATOH1)
 
  6.9585284
 
 5.83e-04
 
 6.47e-03
  
 
  
 8
 
  67940823
 
  67941178
 
  PDF   STATS   ENSEMBL   UCSC 
 
  356
 
  7
 
 39749
 
 tss
 
 0.624074
 
 0.0118566
 
 PPP1R42 (margin: PPP1R42)
 
  6.9550709
 
 5.83e-04
 
 6.47e-03
  
 
  
 6
 
  80657412
 
  80657555
 
  PDF   STATS   ENSEMBL   UCSC 
 
  144
 
  7
 
 35083
 
 tss;island
 
 0.579808
 
 0.0112031
 
 ELOVL4 (margin: ELOVL4; GAPDHP63)
 
  6.9549662
 
 5.83e-04
 
 6.47e-03
  
 
  
 10
 
  75670653
 
  75670793
 
  PDF   STATS   ENSEMBL   UCSC 
 
  141
 
  7
 
 5166
 
 tss
 
 0.208011
 
 0.9603496
 
 PLAU; C10orf55 (margin: PLAU; C10orf55)
 
  6.8469119
 
 5.83e-04
 
 6.47e-03
  
 
  
 7
 
  56160717
 
  56161020
 
  PDF   STATS   ENSEMBL   UCSC 
 
  304
 
  7
 
 37609
 
 tss
 
 0.714170
 
 0.0153108
 
 PHKG1 (margin: snoU13; PHKG1; CHCHD2)
 
  6.8086452
 
 5.83e-04
 
 6.47e-03
  
 
  
 6
 
  34433676
 
  34433789
 
  PDF   STATS   ENSEMBL   UCSC 
 
  114
 
  7
 
 34919
 
 tss;island
 
 0.698268
 
 0.0086130
 
 PACSIN1 (margin: PACSIN1; BX255972.1)
 
  6.8001901
 
 5.83e-04
 
 6.47e-03
  
 
  
 5
 
  43020161
 
  43020716
 
  PDF   STATS   ENSEMBL   UCSC 
 
  556
 
  7
 
 32765
 
 island
 
 0.004369
 
 0.4238735
 
 CTD-2035E11.3; CTD-2201E18.3 (margin: CTD-2035E11.3; CTD-2201E18.3)
 
  6.7980423
 
 5.83e-04
 
 6.47e-03
  
 
  
 18
 
  18822579
 
  18823249
 
  PDF   STATS   ENSEMBL   UCSC 
 
  671
 
  7
 
 19066
 
 gene;island
 
 0.927544
 
 0.1162182
 
 GREB1L; RP11-699A5.2 (margin: GREB1L; RP11-699A5.2)
 
  6.7885639
 
 5.83e-04
 
 6.47e-03
  
 
  
 3
 
 186857284
 
 186857593
 
  PDF   STATS   ENSEMBL   UCSC 
 
  310
 
  7
 
 28778
 
 tss;island
 
 0.004173
 
 0.4018382
 
 RPL39L (margin: RPL39L)
 
  6.7541404
 
 5.83e-04
 
 6.47e-03
  
 
  
 1
 
 156675322
 
 156675562
 
  PDF   STATS   ENSEMBL   UCSC 
 
  241
 
  7
 
 3863
 
 island
 
 0.650819
 
 0.0203913
 
 CRABP2 (margin: CRABP2; RP11-66D17.5)
 
  6.7016593
 
 5.83e-04
 
 6.47e-03
  
 
  
 13
 
  36050844
 
  36051073
 
  PDF   STATS   ENSEMBL   UCSC 
 
  230
 
  7
 
 11390
 
 tss
 
 0.074358
 
 0.8818161
 
 NBEA; MAB21L1 (margin: NBEA; MAB21L1)
 
  6.6757277
 
 5.83e-04
 
 6.47e-03
  
 
  
 1
 
  10056837
 
  10057168
 
  PDF   STATS   ENSEMBL   UCSC 
 
  332
 
  7
 
 1233
 
 tss
 
 0.361523
 
 0.9820241
 
 RBP7 (margin: NMNAT1; RBP7)
 
  6.6657123
 
 5.83e-04
 
 6.47e-03
  
 
  
 19
 
  11877661
 
  11878036
 
  PDF   STATS   ENSEMBL   UCSC 
 
  376
 
  7
 
 21583
 
 island
 
 0.012621
 
 0.5520662
 
 ZNF441 (margin: ZNF441; CTC-499B15.1)
 
  6.6421794
 
 5.83e-04
 
 6.47e-03
  
 
  
 1
 
  13840381
 
  13840712
 
  PDF   STATS   ENSEMBL   UCSC 
 
  332
 
  7
 
 2703
 
 island
 
 0.424862
 
 0.9684898
 
 RP4-597A16.2; LRRC38 (margin: RP4-597A16.2; LRRC38)
 
  6.6327193
 
 5.83e-04
 
 6.47e-03
  
 
  
 20
 
  37230326
 
  37230741
 
  PDF   STATS   ENSEMBL   UCSC 
 
  416
 
  7
 
 26158
 
 island
 
 0.037851
 
 0.8354356
 
 ARHGAP40 (margin: ARHGAP40)
 
  6.5283003
 
 5.83e-04
 
 6.47e-03
  
 
  
 10
 
  44069847
 
  44070200
 
  PDF   STATS   ENSEMBL   UCSC 
 
  354
 
  7
 
 5700
 
 island
 
 0.480529
 
 0.0106859
 
 ZNF239 (margin: ZNF239)
 
  6.5042041
 
 5.83e-04
 
 6.47e-03
  
 
  
 3
 
  49941376
 
  49941613
 
  PDF   STATS   ENSEMBL   UCSC 
 
  238
 
  7
 
 28339
 
 tss;island
 
 0.011266
 
 0.4536348
 
 CTD-2330K9.2; CTD-2330K9.3; MST1R (margin: CTD-2330K9.2; CTD-2330K9.3; MST1R; MON1A)
 
  6.4968236
 
 5.83e-04
 
 6.47e-03
  
 
  
 7
 
  27204663
 
  27205114
 
  PDF   STATS   ENSEMBL   UCSC 
 
  452
 
  7
 
 36938
 
 gene
 
 0.293685
 
 0.9515906
 
 HOXA9; RP1-170O19.20 (margin: HOXA-AS3; RP1-170O19.21; HOXA-AS4; HOXA7; HOXA9; RP1-170O19.20; MIR196B; HOXA10)
 
  6.3849726
 
 5.83e-04
 
 6.47e-03
  
 
  
 4
 
 122302226
 
 122302421
 
  PDF   STATS   ENSEMBL   UCSC 
 
  196
 
  7
 
 30570
 
 tss;island
 
 0.315206
 
 0.9781084
 
 QRFPR (margin: QRFPR; RP11-364P2.2)
 
  6.3792622
 
 5.83e-04
 
 6.47e-03
  
 
  
 8
 
  67344553
 
  67344720
 
  PDF   STATS   ENSEMBL   UCSC 
 
  168
 
  7
 
 40354
 
 island
 
 0.435886
 
 0.9845239
 
 RRS1; ADHFE1 (margin: RRS1; ADHFE1; RP11-346I3.4)
 
  6.3009068
 
 5.83e-04
 
 6.47e-03
  
 
  
 3
 
 168863860
 
 168864313
 
  PDF   STATS   ENSEMBL   UCSC 
 
  454
 
  7
 
 28020
 
 gene;island
 
 0.009253
 
 0.2489342
 
 MECOM (margin: MECOM)
 
  6.2658445
 
 5.83e-04
 
 6.47e-03
  
 
  
 19
 
   4769531
 
   4769690
 
  PDF   STATS   ENSEMBL   UCSC 
 
  160
 
  7
 
 19739
 
 gene;tss
 
 0.407219
 
 0.0055522
 
 MIR7-3HG; MIR7-3 (margin: MIR7-3HG; MIR7-3)
 
  6.1824601
 
 5.83e-04
 
 6.47e-03
  
 
  
 17
 
  15244970
 
  15245237
 
  PDF   STATS   ENSEMBL   UCSC 
 
  268
 
  7
 
 17150
 
 tss
 
 0.349483
 
 0.9807795
 
 TEKT3 (margin: TEKT3)
 
  6.1640356
 
 5.83e-04
 
 6.47e-03
  
 
  
 17
 
  71161157
 
  71161502
 
  PDF   STATS   ENSEMBL   UCSC 
 
  346
 
  7
 
 18699
 
 island
 
 0.694264
 
 0.0314279
 
 RP11-143K11.5; SSTR2; POLR3KP2 (margin: RP11-143K11.5; SSTR2; RP11-143K11.1; POLR3KP2)
 
  6.1343026
 
 5.83e-04
 
 6.47e-03
  
 
  
 3
 
  10206254
 
  10206537
 
  PDF   STATS   ENSEMBL   UCSC 
 
  284
 
  7
 
 28145
 
 tss
 
 0.028970
 
 0.6734771
 
 IRAK2 (margin: IRAK2)
 
  6.1342401
 
 5.83e-04
 
 6.47e-03
  
 
  
 4
 
 155664941
 
 155665249
 
  PDF   STATS   ENSEMBL   UCSC 
 
  309
 
  7
 
 31387
 
 island
 
 0.895922
 
 0.0957878
 
 LRAT (margin: LRAT; RP11-21G20.3)
 
  5.9814872
 
 5.83e-04
 
 6.47e-03
  
 
  
 3
 
  36422059
 
  36422406
 
  PDF   STATS   ENSEMBL   UCSC 
 
  348
 
  7
 
 28978
 
 island
 
 0.041633
 
 0.6191327
 
 STAC (margin: STAC)
 
  5.9664419
 
 5.83e-04
 
 6.47e-03
  
 
  
 11
 
  79151719
 
  79152112
 
  PDF   STATS   ENSEMBL   UCSC 
 
  394
 
  7
 
 7593
 
 tss
 
 0.630142
 
 0.0305186
 
 TENM4 (margin: TENM4)
 
  5.9348672
 
 5.83e-04
 
 6.47e-03
  
 
  
 2
 
 136875897
 
 136876188
 
  PDF   STATS   ENSEMBL   UCSC 
 
  292
 
  7
 
 23649
 
 tss;island
 
 0.589683
 
 0.0087081
 
 CXCR4 (margin: CXCR4)
 
  5.9196518
 
 5.83e-04
 
 6.47e-03
  
 
  
 17
 
   2699021
 
   2699718
 
  PDF   STATS   ENSEMBL   UCSC 
 
  698
 
 10
 
 17009
 
 tss
 
 0.308136
 
 0.9602316
 
 RAP1GAP2 (margin: RAP1GAP2)
 
  5.9062179
 
 4.87e-04
 
 6.47e-03
  
 
  
 2
 
 161126295
 
 161126857
 
  PDF   STATS   ENSEMBL   UCSC 
 
  563
 
  7
 
 24814
 
 island
 
 0.013273
 
 0.4457909
 
 ITGB6; RBMS1 (margin: ITGB6; RBMS1)
 
  5.9014897
 
 5.83e-04
 
 6.47e-03
  
 
  
 12
 
  26986758
 
  26987164
 
  PDF   STATS   ENSEMBL   UCSC 
 
  407
 
 11
 
 9615
 
 tss;island
 
 0.808322
 
 0.0988291
 
 ITPR2 (margin: ITPR2)
 
  5.8886802
 
 5.53e-04
 
 6.47e-03
  
 
  
 5
 
 131592959
 
 131593287
 
  PDF   STATS   ENSEMBL   UCSC 
 
  329
 
  9
 
 32268
 
 tss
 
 0.223401
 
 0.9504618
 
 PDLIM4; P4HA2 (margin: PDLIM4; P4HA2)
 
  5.8460066
 
 4.94e-04
 
 6.47e-03
  
 
  
 11
 
  10472001
 
  10472161
 
  PDF   STATS   ENSEMBL   UCSC 
 
  161
 
  7
 
 7144
 
 tss;island
 
 0.026589
 
 0.5763446
 
 AMPD3 (margin: AMPD3)
 
  5.8341761
 
 5.83e-04
 
 6.47e-03
  
 
  
 15
 
  99644973
 
  99645231
 
  PDF   STATS   ENSEMBL   UCSC 
 
  259
 
  7
 
 13923
 
 tss;island
 
 0.014917
 
 0.3759823
 
 SYNM; RP11-654A16.3 (margin: SYNM; RP11-654A16.3)
 
  5.8002412
 
 5.83e-04
 
 6.47e-03
  
 
  
 11
 
  65547778
 
  65548028
 
  PDF   STATS   ENSEMBL   UCSC 
 
  251
 
  7
 
 8478
 
 island
 
 0.016321
 
 0.5673250
 
 AP5B1; AP001266.1 (margin: RP11-770G2.2; OVOL1; AP5B1; AP001266.1; RP11-770G2.4)
 
  5.7999383
 
 5.83e-04
 
 6.47e-03
  
 
  
 22
 
  18593294
 
  18593609
 
  PDF   STATS   ENSEMBL   UCSC 
 
  316
 
  7
 
 27284
 
 island
 
 0.513269
 
 0.0165961
 
 PEX26; TUBA8 (margin: PEX26; XXbac-B476C20.10; TUBA8; ARL2BPP10)
 
  5.7527451
 
 5.83e-04
 
 6.47e-03
  
 
  
 13
 
  97646639
 
  97646811
 
  PDF   STATS   ENSEMBL   UCSC 
 
  173
 
  7
 
 11506
 
 tss;island
 
 0.340892
 
 0.0137378
 
 OXGR1 (margin: LINC00359; OXGR1)
 
  5.7418192
 
 5.83e-04
 
 6.47e-03
  
 
  
 10
 
 119304379
 
 119304719
 
  PDF   STATS   ENSEMBL   UCSC 
 
  341
 
  7
 
 4786
 
 gene;island
 
 0.458746
 
 0.9843142
 
 EMX2; EMX2OS (margin: EMX2; EMX2OS)
 
  5.6995766
 
 5.83e-04
 
 6.47e-03
  
 
  
 2
 
 176971820
 
 176972068
 
  PDF   STATS   ENSEMBL   UCSC 
 
  249
 
  7
 
 23734
 
 tss
 
 0.017044
 
 0.3842794
 
 HOXD11; AC009336.1; HOXD10 (margin: HOXD13; HOXD12; HOXD11; AC009336.1; HOXD10)
 
  5.6666601
 
 5.83e-04
 
 6.47e-03
  
 
  
 10
 
 103603563
 
 103604019
 
  PDF   STATS   ENSEMBL   UCSC 
 
  457
 
  7
 
 6074
 
 island
 
 0.940615
 
 0.2150244
 
 KCNIP2; C10orf76 (margin: KCNIP2; C10orf76)
 
  5.5991167
 
 5.83e-04
 
 6.47e-03
  
 
  
 15
 
  86313834
 
  86313973
 
  PDF   STATS   ENSEMBL   UCSC 
 
  140
 
  7
 
 13463
 
 gene;tss;island
 
 0.452417
 
 0.9785376
 
 RP11-158M2.6; KLHL25; MIR1276 (margin: RP11-158M2.6; KLHL25; MIR1276)
 
  5.5853424
 
 5.83e-04
 
 6.47e-03
  
 
  
 7
 
 156802150
 
 156802502
 
  PDF   STATS   ENSEMBL   UCSC 
 
  353
 
  7
 
 37317
 
 gene;tss;island
 
 0.671111
 
 0.0366290
 
 RP5-1121A15.1; MNX1 (margin: RP5-1121A15.4; RP5-1121A15.1; MNX1)
 
  5.4977459
 
 5.83e-04
 
 6.47e-03
  
 
  
 14
 
  69951211
 
  69951495
 
  PDF   STATS   ENSEMBL   UCSC 
 
  285
 
  7
 
 12967
 
 island
 
 0.527078
 
 0.0184843
 
 PLEKHD1 (margin: PLEKHD1)
 
  5.4975147
 
 5.83e-04
 
 6.47e-03
  
 
  
 16
 
    215410
 
    215960
 
  PDF   STATS   ENSEMBL   UCSC 
 
  551
 
  7
 
 14962
 
 tss
 
 0.544603
 
 0.9813736
 
 HBM; HBZP1 (margin: HBZ; HBM; HBZP1; Z84721.4; HBA2; HBA1)
 
  5.4402320
 
 5.83e-04
 
 6.47e-03
  
 
  
 3
 
  61547104
 
  61547287
 
  PDF   STATS   ENSEMBL   UCSC 
 
  184
 
  7
 
 29243
 
 island
 
 0.431322
 
 0.0192095
 
 PTPRG (margin: PTPRG)
 
  5.4140578
 
 5.83e-04
 
 6.47e-03
  
 
  
 4
 
  90757351
 
  90757814
 
  PDF   STATS   ENSEMBL   UCSC 
 
  464
 
  7
 
 30057
 
 gene;island
 
 0.264788
 
 0.9623277
 
 RP11-67M1.1; SNCA (margin: RP11-67M1.1; SNCA)
 
  5.3840751
 
 5.83e-04
 
 6.47e-03
  
 
  
 3
 
 192958383
 
 192958968
 
  PDF   STATS   ENSEMBL   UCSC 
 
  586
 
  7
 
 28794
 
 tss;island
 
 0.413652
 
 0.9727717
 
 HRASLS (margin: HRASLS)
 
  5.3767626
 
 5.83e-04
 
 6.47e-03
  
 
  
 20
 
  47896769
 
  47897451
 
  PDF   STATS   ENSEMBL   UCSC 
 
  683
 
  7
 
 25447
 
 gene;island
 
 0.009393
 
 0.3818281
 
 ZFAS1; SNORD12C; SNORD12B; SNORD12; ZNFX1 (margin: ZFAS1; SNORD12C; SNORD12B; SNORD12; ZNFX1)
 
  5.3722028
 
 5.83e-04
 
 6.47e-03
  
 
  
 3
 
 187870621
 
 187871034
 
  PDF   STATS   ENSEMBL   UCSC 
 
  414
 
  7
 
 28780
 
 tss;island
 
 0.195855
 
 0.0029315
 
 LPP; LPP-AS2 (margin: RP11-430L16.1; LPP; LPP-AS2)
 
  5.3412340
 
 5.83e-04
 
 6.47e-03
  
 
  
 11
 
  65405268
 
  65405903
 
  PDF   STATS   ENSEMBL   UCSC 
 
  636
 
 12
 
 8472
 
 island
 
 0.029097
 
 0.5385847
 
 PCNXL3; MIR4690; SIPA1 (margin: PCNXL3; MIR4690; SIPA1; MIR4489)
 
  5.3079289
 
 4.96e-04
 
 6.47e-03
  
 
  
 4
 
   8271196
 
   8271507
 
  PDF   STATS   ENSEMBL   UCSC 
 
  312
 
  7
 
 30917
 
 island
 
 0.451292
 
 0.9645153
 
 HTRA3 (margin: HTRA3)
 
  5.2964071
 
 5.83e-04
 
 6.47e-03
  
 
  
 11
 
 128775513
 
 128775750
 
  PDF   STATS   ENSEMBL   UCSC 
 
  238
 
  7
 
 6969
 
 gene
 
 0.007703
 
 0.1581079
 
 KCNJ5; C11orf45 (margin: KCNJ5; C11orf45)
 
  5.2958536
 
 5.83e-04
 
 6.47e-03
  
 
  
 11
 
    826837
 
    827613
 
  PDF   STATS   ENSEMBL   UCSC 
 
  777
 
  7
 
 7885
 
 island
 
 0.434926
 
 0.0197041
 
 PNPLA2; EFCAB4A; AP006621.8 (margin: PNPLA2; EFCAB4A; CD151; AP006621.8; POLR2L)
 
  5.2609051
 
 5.83e-04
 
 6.47e-03
  
 
  
 8
 
  24772137
 
  24772435
 
  PDF   STATS   ENSEMBL   UCSC 
 
  299
 
  7
 
 39605
 
 tss
 
 0.667441
 
 0.9816032
 
 NEFM; GS1-72M22.1 (margin: NEFM; RP11-624C23.1; GS1-72M22.1)
 
  5.2335158
 
 5.83e-04
 
 6.47e-03
  
 
  
 17
 
  80797948
 
  80798078
 
  PDF   STATS   ENSEMBL   UCSC 
 
  131
 
  7
 
 17781
 
 tss
 
 0.336913
 
 0.0158649
 
 TBCD; ZNF750 (margin: TBCD; ZNF750)
 
  5.2266563
 
 5.83e-04
 
 6.47e-03
  
 
  
 20
 
  50419082
 
  50419348
 
  PDF   STATS   ENSEMBL   UCSC 
 
  267
 
  7
 
 25826
 
 tss
 
 0.972177
 
 0.5235106
 
 SALL4 (margin: SALL4)
 
  5.1742695
 
 5.83e-04
 
 6.47e-03
  
 
  
 4
 
 108852496
 
 108852651
 
  PDF   STATS   ENSEMBL   UCSC 
 
  156
 
  7
 
 30535
 
 tss;island
 
 0.227892
 
 0.0114914
 
 CYP2U1; RP11-286E11.1 (margin: CYP2U1; RP11-286E11.1; RP11-286E11.2)
 
  5.1359401
 
 5.83e-04
 
 6.47e-03
  
 
  
 6
 
  74161911
 
  74162142
 
  PDF   STATS   ENSEMBL   UCSC 
 
  232
 
  7
 
 36256
 
 island
 
 0.023750
 
 0.4124424
 
 MB21D1 (margin: MTO1; RNU6-975P; MB21D1)
 
  5.1337959
 
 5.83e-04
 
 6.47e-03
  
 
  
 7
 
  51384371
 
  51384676
 
  PDF   STATS   ENSEMBL   UCSC 
 
  306
 
  7
 
 38495
 
 island
 
 0.374087
 
 0.0176542
 
 COBL (margin: COBL)
 
  5.0824096
 
 5.83e-04
 
 6.47e-03
  
 
  
 8
 
  12990898
 
  12991196
 
  PDF   STATS   ENSEMBL   UCSC 
 
  299
 
  7
 
 39560
 
 tss
 
 0.162281
 
 0.0067569
 
 DLC1 (margin: DLC1)
 
  5.0127749
 
 5.83e-04
 
 6.47e-03
  
 
  
 5
 
 180018465
 
 180018735
 
  PDF   STATS   ENSEMBL   UCSC 
 
  271
 
  7
 
 33504
 
 island
 
 0.381737
 
 0.9769426
 
 SCGB3A1 (margin: AC122714.1; SCGB3A1; FLT4)
 
  5.0025712
 
 5.83e-04
 
 6.47e-03
  
 
  
 5
 
 140624869
 
 140625130
 
  PDF   STATS   ENSEMBL   UCSC 
 
  262
 
  7
 
 32369
 
 tss
 
 0.428592
 
 0.9620285
 
 PCDHB15 (margin: PCDHB18; PCDHB19P; PCDHB15)
 
  4.9843689
 
 5.83e-04
 
 6.47e-03
  
 
  
 20
 
  44098223
 
  44098409
 
  PDF   STATS   ENSEMBL   UCSC 
 
  187
 
  7
 
 26200
 
 island
 
 0.019264
 
 0.3984748
 
 WFDC2; AL031663.1 (margin: WFDC2; AL031663.1)
 
  4.9730303
 
 5.83e-04
 
 6.47e-03
  
 
  
 8
 
  40755165
 
  40755559
 
  PDF   STATS   ENSEMBL   UCSC 
 
  395
 
  7
 
 40240
 
 island
 
 0.494995
 
 0.0174080
 
 ZMAT4; RP11-465K16.1 (margin: ZMAT4; RP11-465K16.1)
 
  4.9723053
 
 5.83e-04
 
 6.47e-03
  
 
  
 1
 
   6085799
 
   6086275
 
  PDF   STATS   ENSEMBL   UCSC 
 
  477
 
  7
 
 1196
 
 tss
 
 0.027194
 
 0.5104381
 
 KCNAB2 (margin: KCNAB2)
 
  4.8934652
 
 5.83e-04
 
 6.47e-03
  
 
  
 6
 
  31685392
 
  31685575
 
  PDF   STATS   ENSEMBL   UCSC 
 
  184
 
  7
 
 33940
 
 gene
 
 0.607218
 
 0.0278658
 
 LY6G6F; MEGT1; LY6G6D; C6orf25; LY6G6C (margin: LY6G6F; MEGT1; LY6G6D; C6orf25; XXbac-BPG32J3.20; LY6G6E; LY6G6C; DDAH2)
 
  4.8733815
 
 5.83e-04
 
 6.47e-03
  
 
  
 15
 
  83378614
 
  83378895
 
  PDF   STATS   ENSEMBL   UCSC 
 
  282
 
  9
 
 14385
 
 island
 
 0.101681
 
 0.0072371
 
 AP3B2; AC105339.1 (margin: AP3B2; AC105339.1)
 
  4.7789055
 
 4.94e-04
 
 6.47e-03
  
 
  
 12
 
  12848977
 
  12849748
 
  PDF   STATS   ENSEMBL   UCSC 
 
  772
 
 11
 
 10250
 
 island
 
 0.074570
 
 0.0083179
 
 GPR19 (margin: RP11-180M15.3; GPR19)
 
  4.7312214
 
 5.53e-04
 
 6.47e-03
  
 
  
 2
 
 233497957
 
 233498223
 
  PDF   STATS   ENSEMBL   UCSC 
 
  267
 
  7
 
 23068
 
 gene;island
 
 0.369572
 
 0.0266633
 
 EFHD1 (margin: EFHD1)
 
  4.7047910
 
 5.83e-04
 
 6.47e-03
  
 
  
 6
 
  36237982
 
  36238230
 
  PDF   STATS   ENSEMBL   UCSC 
 
  249
 
  7
 
 34935
 
 tss
 
 0.032196
 
 0.4590459
 
 PNPLA1 (margin: PNPLA1)
 
  4.6740998
 
 5.83e-04
 
 6.47e-03
  
 
  
 15
 
  41056021
 
  41056369
 
  PDF   STATS   ENSEMBL   UCSC 
 
  349
 
  7
 
 14045
 
 island
 
 0.223257
 
 0.0162463
 
 GCHFR (margin: RP11-532F12.6; GCHFR; C15orf62; RMDN3; DNAJC17)
 
  4.6055942
 
 5.83e-04
 
 6.47e-03
  
 
  
 4
 
 114683176
 
 114683375
 
  PDF   STATS   ENSEMBL   UCSC 
 
  200
 
  7
 
 30556
 
 tss;island
 
 0.054651
 
 0.5827425
 
 CAMK2D (margin: CAMK2D)
 
  4.5879208
 
 5.83e-04
 
 6.47e-03
  
 
  
 3
 
  32859377
 
  32859587
 
  PDF   STATS   ENSEMBL   UCSC 
 
  211
 
  7
 
 28968
 
 island
 
 0.550407
 
 0.9780749
 
 TRIM71 (margin: TRIM71)
 
  4.5784161
 
 5.83e-04
 
 6.47e-03
  
 
  
 3
 
 183959000
 
 183959171
 
  PDF   STATS   ENSEMBL   UCSC 
 
  172
 
  7
 
 28057
 
 gene;tss;island
 
 0.516725
 
 0.9258241
 
 EIF2B5; VWA5B2; MIR1224; ALG3 (margin: EIF2B5; VWA5B2; MIR1224; ECE2; ALG3)
 
  4.4828383
 
 5.83e-04
 
 6.47e-03
  
 
  
 12
 
 114843188
 
 114843955
 
  PDF   STATS   ENSEMBL   UCSC 
 
  768
 
 10
 
 9326
 
 gene;island
 
 0.365114
 
 0.8509800
 
 TBX5 (margin: TBX5-AS1; TBX5)
 
  4.4418350
 
 4.87e-04
 
 6.47e-03
  
 
  
 3
 
 180319597
 
 180320159
 
  PDF   STATS   ENSEMBL   UCSC 
 
  563
 
  9
 
 29684
 
 island
 
 0.025245
 
 0.3314089
 
 TTC14; RP11-496B10.3; CCDC39 (margin: TTC14; RP11-496B10.3; RP11-496B10.1; CCDC39)
 
  4.4140386
 
 4.94e-04
 
 6.47e-03
  
 
  
 10
 
  26726877
 
  26727261
 
  PDF   STATS   ENSEMBL   UCSC 
 
  385
 
  7
 
 4994
 
 tss
 
 0.489592
 
 0.9020204
 
 APBB1IP (margin: APBB1IP)
 
  4.3715963
 
 5.83e-04
 
 6.47e-03
  
 
  
 15
 
  83240550
 
  83240791
 
  PDF   STATS   ENSEMBL   UCSC 
 
  242
 
  7
 
 13453
 
 gene
 
 0.487301
 
 0.8441474
 
 CPEB1 (margin: RP11-379H8.1; CPEB1)
 
  4.3171501
 
 5.83e-04
 
 6.47e-03
  
 
  
 5
 
 170736021
 
 170736277
 
  PDF   STATS   ENSEMBL   UCSC 
 
  257
 
  7
 
 32486
 
 tss
 
 0.775874
 
 0.9758764
 
 TLX3 (margin: RANBP17; TLX3)
 
  4.3005498
 
 5.83e-04
 
 6.47e-03
  
 
  
 10
 
  28034669
 
  28034856
 
  PDF   STATS   ENSEMBL   UCSC 
 
  188
 
  7
 
 5634
 
 island
 
 0.739197
 
 0.9774543
 
 RP11-360I20.2; MKX (margin: RP11-360I20.2; MKX)
 
  4.2929031
 
 5.83e-04
 
 6.47e-03
  
 
  
 4
 
 186732837
 
 186733060
 
  PDF   STATS   ENSEMBL   UCSC 
 
  224
 
  7
 
 30212
 
 gene;tss
 
 0.755083
 
 0.9832416
 
 SORBS2 (margin: SORBS2)
 
  4.2920419
 
 5.83e-04
 
 6.47e-03
  
 
  
 12
 
 114846313
 
 114846503
 
  PDF   STATS   ENSEMBL   UCSC 
 
  191
 
  7
 
 10033
 
 tss
 
 0.638526
 
 0.9698051
 
 TBX5-AS1; TBX5 (margin: TBX5-AS1; TBX5)
 
  4.2919613
 
 5.83e-04
 
 6.47e-03
  
 
  
 11
 
  69590282
 
  69590580
 
  PDF   STATS   ENSEMBL   UCSC 
 
  299
 
  7
 
 7522
 
 tss
 
 0.517153
 
 0.9544217
 
 FGF4 (margin: AP001888.1; FGF4)
 
  4.2759978
 
 5.83e-04
 
 6.47e-03
  
 
  
 10
 
 133508246
 
 133508866
 
  PDF   STATS   ENSEMBL   UCSC 
 
  621
 
  7
 
 6278
 
 island
 
 0.297049
 
 0.9447354
 
  (margin: )
 
  4.2724848
 
 5.83e-04
 
 6.47e-03
  
 
  
 7
 
  82073339
 
  82073810
 
  PDF   STATS   ENSEMBL   UCSC 
 
  472
 
  7
 
 37677
 
 tss;island
 
 0.438566
 
 0.0268650
 
 CACNA2D1 (margin: CACNA2D1)
 
  4.2571453
 
 5.83e-04
 
 6.47e-03
  
 
  
 11
 
  85565852
 
  85565989
 
  PDF   STATS   ENSEMBL   UCSC 
 
  138
 
  7
 
 7606
 
 tss;island
 
 0.006200
 
 0.1141509
 
 CCDC83; AP000974.1 (margin: CCDC83; AP000974.1)
 
  4.1930021
 
 5.83e-04
 
 6.47e-03
  
 
  
 5
 
   1225074
 
   1225719
 
  PDF   STATS   ENSEMBL   UCSC 
 
  646
 
 10
 
 32616
 
 island
 
 0.253550
 
 0.9002765
 
 SLC6A19; SLC6A18 (margin: SLC6A19; SLC6A18)
 
  4.1724857
 
 4.87e-04
 
 6.47e-03
  
 
  
 7
 
 120968877
 
 120969079
 
  PDF   STATS   ENSEMBL   UCSC 
 
  203
 
  7
 
 37861
 
 tss
 
 0.839002
 
 0.1985319
 
 WNT16 (margin: WNT16)
 
  4.1592921
 
 5.83e-04
 
 6.47e-03
  
 
  
 5
 
  41510325
 
  41510851
 
  PDF   STATS   ENSEMBL   UCSC 
 
  527
 
  7
 
 32754
 
 island
 
 0.401197
 
 0.0368686
 
 PLCXD3 (margin: PLCXD3)
 
  4.1288015
 
 5.83e-04
 
 6.47e-03
  
 
  
 6
 
 147829799
 
 147830055
 
  PDF   STATS   ENSEMBL   UCSC 
 
  257
 
  7
 
 35279
 
 tss
 
 0.724120
 
 0.9846343
 
 SAMD5 (margin: SAMD5)
 
  4.1168399
 
 5.83e-04
 
 6.47e-03
  
 
  
 22
 
  38071455
 
  38071677
 
  PDF   STATS   ENSEMBL   UCSC 
 
  223
 
  7
 
 27468
 
 island
 
 0.019314
 
 0.2254494
 
 LGALS1 (margin: SH3BP1; PDXP; LGALS1; NOL12; RP1-37E16.12; RN7SL385P)
 
  4.0989901
 
 5.83e-04
 
 6.47e-03
  
 
  
 14
 
  54421381
 
  54421578
 
  PDF   STATS   ENSEMBL   UCSC 
 
  198
 
  7
 
 12083
 
 gene;tss;island
 
 0.612948
 
 0.9559227
 
 BMP4 (margin: MIR5580; BMP4)
 
  4.0026774
 
 5.83e-04
 
 6.47e-03
  
 
  
 3
 
   9811714
 
   9811990
 
  PDF   STATS   ENSEMBL   UCSC 
 
  277
 
  7
 
 28130
 
 tss;island
 
 0.702927
 
 0.1645705
 
 OGG1; CAMK1 (margin: OGG1; CAMK1; TADA3)
 
  3.9631630
 
 5.83e-04
 
 6.47e-03
  
 
  
 19
 
  19648890
 
  19649054
 
  PDF   STATS   ENSEMBL   UCSC 
 
  165
 
  7
 
 20645
 
 tss
 
 0.283654
 
 0.7852041
 
 YJEFN3; CILP2 (margin: NDUFA13; CTC-260F20.3; YJEFN3; CILP2)
 
  3.9342525
 
 5.83e-04
 
 6.47e-03
  
 
  
 15
 
  50474069
 
  50474388
 
  PDF   STATS   ENSEMBL   UCSC 
 
  320
 
  7
 
 13665
 
 tss;island
 
 0.839302
 
 0.2916339
 
 SLC27A2; ATP8B4 (margin: SLC27A2; ATP8B4)
 
  3.9174294
 
 5.83e-04
 
 6.47e-03
  
 
  
 20
 
  45985495
 
  45985741
 
  PDF   STATS   ENSEMBL   UCSC 
 
  247
 
  7
 
 25799
 
 tss
 
 0.097296
 
 0.6144122
 
 ZMYND8 (margin: RP4-569M23.2; ZMYND8)
 
  3.8876542
 
 5.83e-04
 
 6.47e-03
  
 
  
 11
 
  75379432
 
  75379624
 
  PDF   STATS   ENSEMBL   UCSC 
 
  193
 
  7
 
 8665
 
 island
 
 0.559345
 
 0.9410024
 
 MAP6 (margin: MAP6)
 
  3.8814881
 
 5.83e-04
 
 6.47e-03
  
 
  
 17
 
  12569089
 
  12569204
 
  PDF   STATS   ENSEMBL   UCSC 
 
  116
 
  7
 
 17141
 
 tss
 
 0.579174
 
 0.9281741
 
 MYOCD (margin: MYOCD; AC005358.3)
 
  3.8743684
 
 5.83e-04
 
 6.47e-03
  
 
  
 16
 
   2261069
 
   2261237
 
  PDF   STATS   ENSEMBL   UCSC 
 
  169
 
  7
 
 15021
 
 tss
 
 0.603656
 
 0.9546712
 
 MLST8; RP11-304L19.8; BRICD5; PGP (margin: MLST8; RP11-304L19.8; AC009065.3; BRICD5; PGP)
 
  3.7924449
 
 5.83e-04
 
 6.47e-03
  
 
  
 15
 
 100882020
 
 100882647
 
  PDF   STATS   ENSEMBL   UCSC 
 
  628
 
 11
 
 14507
 
 island
 
 0.230900
 
 0.0154381
 
 ADAMTS17 (margin: ADAMTS17; SPATA41)
 
  3.7823255
 
 5.53e-04
 
 6.47e-03
  
 
  
 19
 
  11998457
 
  11998660
 
  PDF   STATS   ENSEMBL   UCSC 
 
  204
 
  7
 
 20484
 
 tss
 
 0.407806
 
 0.8684481
 
 ZNF69 (margin: ZNF439; ZNF69)
 
  3.7234313
 
 5.83e-04
 
 6.47e-03
  
 
  
 12
 
    322514
 
    322983
 
  PDF   STATS   ENSEMBL   UCSC 
 
  470
 
 10
 
 8991
 
 gene;tss
 
 0.337324
 
 0.8694782
 
 SLC6A12 (margin: RP11-283I3.2; SLC6A12; SLC6A13)
 
  3.7089270
 
 4.87e-04
 
 6.47e-03
  
 
  
 19
 
  12075601
 
  12076348
 
  PDF   STATS   ENSEMBL   UCSC 
 
  748
 
 11
 
 21588
 
 island
 
 0.497687
 
 0.8401420
 
 ZNF763 (margin: ZNF763; CTD-2006C1.12)
 
  3.5113888
 
 5.53e-04
 
 6.47e-03
  
 
  
 6
 
  32185818
 
  32186244
 
  PDF   STATS   ENSEMBL   UCSC 
 
  427
 
  9
 
 34097
 
 gene
 
 0.239205
 
 0.7288425
 
 NOTCH4 (margin: NOTCH4)
 
  3.4740531
 
 4.94e-04
 
 6.47e-03
  
 
  
 7
 
   4347499
 
   4348107
 
  PDF   STATS   ENSEMBL   UCSC 
 
  609
 
  7
 
 38187
 
 island
 
 0.447791
 
 0.8971555
 
  (margin: )
 
  3.4287279
 
 5.83e-04
 
 6.47e-03
  
 
  
 11
 
 120110544
 
 120110887
 
  PDF   STATS   ENSEMBL   UCSC 
 
  344
 
  9
 
 7761
 
 tss
 
 0.509804
 
 0.9244239
 
 POU2F3 (margin: OAF; POU2F3; RP11-778O17.4)
 
  3.4101855
 
 4.94e-04
 
 6.47e-03
  
 
  
 7
 
 116139180
 
 116139425
 
  PDF   STATS   ENSEMBL   UCSC 
 
  246
 
  7
 
 37849
 
 tss
 
 0.110220
 
 0.3631043
 
 CAV2; AC002066.1 (margin: CAV2; AC002066.1)
 
  3.3914233
 
 5.83e-04
 
 6.47e-03
  
 
  
 16
 
   2260770
 
   2261237
 
  PDF   STATS   ENSEMBL   UCSC 
 
  468
 
 10
 
 15593
 
 island
 
 0.655808
 
 0.9510204
 
 MLST8; RP11-304L19.8; BRICD5; PGP (margin: MLST8; RP11-304L19.8; AC009065.3; BRICD5; PGP)
 
  3.3717026
 
 4.87e-04
 
 6.47e-03
  
 
  
 4
 
  42399699
 
  42399858
 
  PDF   STATS   ENSEMBL   UCSC 
 
  160
 
  7
 
 31014
 
 island
 
 0.833178
 
 0.9823425
 
 SHISA3 (margin: SHISA3; RP11-63A11.1; ATP8A1)
 
  3.3186975
 
 5.83e-04
 
 6.47e-03
  
 
  
 6
 
 167275778
 
 167275999
 
  PDF   STATS   ENSEMBL   UCSC 
 
  222
 
  7
 
 35338
 
 tss
 
 0.751792
 
 0.1840614
 
 RPS6KA2; RP11-514O12.4 (margin: RPS6KA2; RP11-514O12.4)
 
  3.2483990
 
 5.83e-04
 
 6.47e-03
  
 
  
 3
 
  42947263
 
  42947771
 
  PDF   STATS   ENSEMBL   UCSC 
 
  509
 
  7
 
 29023
 
 island
 
 0.898288
 
 0.9871415
 
 ACKR2; KRBOX1; ZNF662 (margin: ACKR2; KRBOX1; ZNF662)
 
  3.2184323
 
 5.83e-04
 
 6.47e-03
  
 
  
 12
 
  81471311
 
  81471757
 
  PDF   STATS   ENSEMBL   UCSC 
 
  447
 
  7
 
 9894
 
 tss
 
 0.093108
 
 0.0171431
 
 ACSS3 (margin: ACSS3)
 
  3.2165771
 
 5.83e-04
 
 6.47e-03
  
 
  
 18
 
  42259977
 
  42260234
 
  PDF   STATS   ENSEMBL   UCSC 
 
  258
 
  7
 
 19249
 
 tss;island
 
 0.107152
 
 0.0150718
 
 SETBP1; RP11-456K23.1 (margin: SETBP1; RP11-456K23.1)
 
  3.2072146
 
 5.83e-04
 
 6.47e-03
  
 
  
 8
 
  82192519
 
  82192777
 
  PDF   STATS   ENSEMBL   UCSC 
 
  259
 
  7
 
 39784
 
 tss
 
 0.074019
 
 0.4500747
 
 FABP5; RP11-363E6.3 (margin: FABP5; RP11-363E6.4; RP11-363E6.3)
 
  3.1698717
 
 5.83e-04
 
 6.47e-03
  
 
  
 12
 
  72667326
 
  72667707
 
  PDF   STATS   ENSEMBL   UCSC 
 
  382
 
  7
 
 9877
 
 tss
 
 0.221887
 
 0.6987639
 
 TRHDE; TRHDE-AS1 (margin: TRHDE; TRHDE-AS1)
 
  2.9941492
 
 5.83e-04
 
 6.47e-03
  
 
  
 17
 
  74533845
 
  74534090
 
  PDF   STATS   ENSEMBL   UCSC 
 
  246
 
  7
 
 17681
 
 tss;island
 
 0.919732
 
 0.9829892
 
 PRCD; CYGB (margin: PRCD; RP11-666A8.7; CYGB; RP11-666A8.8)
 
  2.9773337
 
 5.83e-04
 
 6.47e-03
  
 
  
 1
 
 185703201
 
 185703659
 
  PDF   STATS   ENSEMBL   UCSC 
 
  459
 
 11
 
 2144
 
 tss
 
 0.158705
 
 0.0170500
 
 HMCN1 (margin: HMCN1)
 
  2.9540040
 
 5.53e-04
 
 6.47e-03
  
 
  
 17
 
   8230130
 
   8230940
 
  PDF   STATS   ENSEMBL   UCSC 
 
  811
 
  7
 
 18001
 
 island
 
 0.816762
 
 0.9718441
 
 SNORA69 (margin: ARHGEF15; SNORA69; RP11-849F2.4; AC135178.7)
 
  2.8609101
 
 5.83e-04
 
 6.47e-03
  
 
  
 17
 
  79304498
 
  79304683
 
  PDF   STATS   ENSEMBL   UCSC 
 
  186
 
  7
 
 17737
 
 tss
 
 0.910714
 
 0.6508686
 
 TMEM105 (margin: TMEM105)
 
  2.7020522
 
 5.83e-04
 
 6.47e-03
  
 
  
 14
 
  96505296
 
  96505874
 
  PDF   STATS   ENSEMBL   UCSC 
 
  579
 
 12
 
 13114
 
 island
 
 0.130796
 
 0.0189269
 
 C14orf132 (margin: C14orf132)
 
  2.6631797
 
 4.96e-04
 
 6.47e-03
  
 
  
 1
 
  45792540
 
  45792733
 
  PDF   STATS   ENSEMBL   UCSC 
 
  194
 
  7
 
 3198
 
 island
 
 0.375137
 
 0.1130303
 
 HPDL (margin: HPDL; MUTYH)
 
  2.6506991
 
 5.83e-04
 
 6.47e-03
  
 
  
 2
 
  24232603
 
  24232974
 
  PDF   STATS   ENSEMBL   UCSC 
 
  372
 
 11
 
 24125
 
 island
 
 0.918705
 
 0.9845189
 
 MFSD2B (margin: UBXN2A; MFSD2B)
 
  2.5643079
 
 5.53e-04
 
 6.47e-03
  
 
  
 14
 
 101521409
 
 101521809
 
  PDF   STATS   ENSEMBL   UCSC 
 
  401
 
 10
 
 12629
 
 tss
 
 0.495308
 
 0.8654124
 
 MIR134; MIR485; MIR323B; MIR382 (margin: MIR1185-2; MIR381HG; MIR381; MIR487B; MIR539; MIR889; MIR544A; MIR655; AL132709.3; MIR487A; MIR134; MIR485; MIR323B; MIR154; MIR496; MIR377; MIR541; MIR409; MIR412; MIR369; MIR410; MIR656; MIR382)
 
  2.5233364
 
 4.87e-04
 
 6.47e-03
  
 
  
 3
 
 111717534
 
 111717969
 
  PDF   STATS   ENSEMBL   UCSC 
 
  436
 
  7
 
 28498
 
 tss
 
 0.548037
 
 0.8274762
 
 TAGLN3 (margin: ABHD10; TAGLN3)
 
  2.4846327
 
 5.83e-04
 
 6.47e-03
  
 
  
 10
 
 126138415
 
 126138879
 
  PDF   STATS   ENSEMBL   UCSC 
 
  465
 
  7
 
 6228
 
 island
 
 0.147673
 
 0.4876840
 
 RP13-238F13.3; NKX1-2 (margin: RP13-238F13.3; LHPP; RP13-238F13.5; NKX1-2)
 
  2.4661753
 
 5.83e-04
 
 6.47e-03
  
 
  
 4
 
 156680007
 
 156680518
 
  PDF   STATS   ENSEMBL   UCSC 
 
  512
 
  7
 
 31392
 
 island
 
 0.837231
 
 0.4481213
 
 GUCY1B3 (margin: GUCY1B3)
 
  2.3382275
 
 5.83e-04
 
 6.47e-03
  
 
  
 10
 
  93392644
 
  93393509
 
  PDF   STATS   ENSEMBL   UCSC 
 
  866
 
 14
 
 5932
 
 island
 
 0.504912
 
 0.9011407
 
 PPP1R3C (margin: PPP1R3C)
 
  2.2794772
 
 5.23e-04
 
 6.47e-03
  
 
  
 8
 
  48675647
 
  48676092
 
  PDF   STATS   ENSEMBL   UCSC 
 
  446
 
  7
 
 40273
 
 island
 
 0.892919
 
 0.9702679
 
  (margin: PRKDC)
 
  2.2351058
 
 5.83e-04
 
 6.47e-03
  
 
  
 7
 
  73894573
 
  73895278
 
  PDF   STATS   ENSEMBL   UCSC 
 
  706
 
  7
 
 37044
 
 gene;island
 
 0.790658
 
 0.9489686
 
 GTF2IRD1 (margin: GTF2IRD1; RNA5SP233)
 
  2.1000289
 
 5.83e-04
 
 6.47e-03
  
 
  
 6
 
  31762353
 
  31762901
 
  PDF   STATS   ENSEMBL   UCSC 
 
  549
 
 15
 
 33958
 
 gene;island
 
 0.009670
 
 0.0317057
 
 VARS (margin: VARS; LSM2)
 
  1.8712794
 
 5.78e-04
 
 6.47e-03
  
 
  
 6
 
  31865522
 
  31866286
 
  PDF   STATS   ENSEMBL   UCSC 
 
  765
 
 26
 
 34849
 
 tss;island
 
 0.003202
 
 0.0105474
 
 C2; EHMT2; ZBTB12 (margin: C2; EHMT2; ZBTB12)
 
  1.6630273
 
 5.18e-04
 
 6.47e-03
  
 
  
 19
 
    291986
 
    292498
 
  PDF   STATS   ENSEMBL   UCSC 
 
  513
 
 12
 
 20270
 
 tss;island
 
 0.925731
 
 0.9732563
 
 PPAP2C (margin: VN2R11P; PPAP2C)
 
  1.5566789
 
 4.96e-04
 
 6.47e-03
  
 
  
 14
 
  23834710
 
  23834995
 
  PDF   STATS   ENSEMBL   UCSC 
 
  286
 
  8
 
 12718
 
 island
 
 0.012988
 
 0.9746902
 
 EFS (margin: RP11-124D2.3; IL25; CMTM5; EFS)
 
 10.8096687
 
 6.22e-04
 
 6.71e-03
  
 
  
 10
 
   6622279
 
   6622566
 
  PDF   STATS   ENSEMBL   UCSC 
 
  288
 
  8
 
 4935
 
 tss;island
 
 0.012952
 
 0.8470744
 
 PRKCQ-AS1; PRKCQ (margin: PRKCQ-AS1; RP11-5N23.3; PRKCQ)
 
  7.1685243
 
 6.22e-04
 
 6.71e-03
  
 
  
 17
 
  45056626
 
  45056976
 
  PDF   STATS   ENSEMBL   UCSC 
 
  351
 
  8
 
 17438
 
 tss;island
 
 0.495075
 
 0.0083221
 
 GOSR2; RP11-156P1.2; LRRC37A17P; RPRML (margin: GOSR2; RP11-156P1.2; MIR5089; LRRC37A17P; RPRML; RP11-156P1.3)
 
  6.7787969
 
 6.22e-04
 
 6.71e-03
  
 
  
 5
 
 113697861
 
 113698506
 
  PDF   STATS   ENSEMBL   UCSC 
 
  646
 
  8
 
 33017
 
 island
 
 0.918513
 
 0.0455418
 
 KCNN2 (margin: KCNN2)
 
  6.4832717
 
 6.22e-04
 
 6.71e-03
  
 
  
 5
 
 150284416
 
 150284796
 
  PDF   STATS   ENSEMBL   UCSC 
 
  381
 
  8
 
 32435
 
 tss
 
 0.332093
 
 0.9247521
 
 ZNF300 (margin: IRGM; ZNF300)
 
  5.6141326
 
 6.22e-04
 
 6.71e-03
  
 
  
 15
 
  83378637
 
  83378895
 
  PDF   STATS   ENSEMBL   UCSC 
 
  259
 
  8
 
 13859
 
 tss
 
 0.136691
 
 0.0063408
 
 AP3B2; AC105339.1 (margin: AP3B2; AC105339.1)
 
  5.4946768
 
 6.22e-04
 
 6.71e-03
  
 
  
 17
 
  72889708
 
  72890001
 
  PDF   STATS   ENSEMBL   UCSC 
 
  294
 
  8
 
 17634
 
 tss
 
 0.468382
 
 0.9546383
 
 FADS6 (margin: FADS6)
 
  5.4930984
 
 6.22e-04
 
 6.71e-03
  
 
  
 2
 
  30454146
 
  30454643
 
  PDF   STATS   ENSEMBL   UCSC 
 
  498
 
  8
 
 24201
 
 island
 
 0.211085
 
 0.7157188
 
 LBH (margin: LBH)
 
  5.4567422
 
 6.22e-04
 
 6.71e-03
  
 
  
 19
 
  39989234
 
  39989592
 
  PDF   STATS   ENSEMBL   UCSC 
 
  359
 
  8
 
 21999
 
 island
 
 0.070824
 
 0.6105802
 
 DLL3 (margin: TIMM50; DLL3)
 
  5.2076217
 
 6.22e-04
 
 6.71e-03
  
 
  
 5
 
  65891878
 
  65892187
 
  PDF   STATS   ENSEMBL   UCSC 
 
  310
 
  8
 
 32843
 
 island
 
 0.285027
 
 0.0158711
 
 MAST4 (margin: MAST4)
 
  5.0979582
 
 6.22e-04
 
 6.71e-03
  
 
  
 19
 
  37341052
 
  37341373
 
  PDF   STATS   ENSEMBL   UCSC 
 
  322
 
  8
 
 21948
 
 island
 
 0.390930
 
 0.0193650
 
 ZNF345; ZNF790 (margin: ZNF345; ZNF790; Y_RNA)
 
  5.0107061
 
 6.22e-04
 
 6.71e-03
  
 
  
 2
 
 223164459
 
 223164925
 
  PDF   STATS   ENSEMBL   UCSC 
 
  467
 
  8
 
 23049
 
 gene;tss;island
 
 0.696724
 
 0.9806264
 
 CCDC140; PAX3 (margin: CCDC140; PAX3)
 
  4.9709602
 
 6.22e-04
 
 6.71e-03
  
 
  
 1
 
  44870946
 
  44871634
 
  PDF   STATS   ENSEMBL   UCSC 
 
  689
 
  8
 
 3181
 
 island
 
 0.219642
 
 0.0091744
 
 RNF220 (margin: RNF220)
 
  4.8576910
 
 6.22e-04
 
 6.71e-03
  
 
  
 13
 
 113633379
 
 113634042
 
  PDF   STATS   ENSEMBL   UCSC 
 
  664
 
  8
 
 11263
 
 gene;island
 
 0.208671
 
 0.8966254
 
 MCF2L (margin: MCF2L; MCF2L-AS1)
 
  4.6857766
 
 6.22e-04
 
 6.71e-03
  
 
  
 10
 
 115938729
 
 115939284
 
  PDF   STATS   ENSEMBL   UCSC 
 
  556
 
  8
 
 6151
 
 island
 
 0.959261
 
 0.5831036
 
 TDRD1 (margin: TDRD1; C10orf118; MIR2110)
 
  4.4288130
 
 6.22e-04
 
 6.71e-03
  
 
  
 13
 
  98829311
 
  98829666
 
  PDF   STATS   ENSEMBL   UCSC 
 
  356
 
  8
 
 11197
 
 gene;island
 
 0.669895
 
 0.9788841
 
 FARP1; RNF113B (margin: FARP1; RNF113B)
 
  4.3314678
 
 6.22e-04
 
 6.71e-03
  
 
  
 6
 
  21666391
 
  21666939
 
  PDF   STATS   ENSEMBL   UCSC 
 
  549
 
  8
 
 35486
 
 island
 
 0.477496
 
 0.9216157
 
 LINC00340 (margin: LINC00340)
 
  4.3258023
 
 6.22e-04
 
 6.71e-03
  
 
  
 10
 
  26726877
 
  26727318
 
  PDF   STATS   ENSEMBL   UCSC 
 
  442
 
  8
 
 5619
 
 island
 
 0.491990
 
 0.9332860
 
 APBB1IP (margin: APBB1IP)
 
  4.0499172
 
 6.22e-04
 
 6.71e-03
  
 
  
 7
 
  27213966
 
  27214383
 
  PDF   STATS   ENSEMBL   UCSC 
 
  418
 
  8
 
 37498
 
 tss
 
 0.889836
 
 0.9893164
 
 RP1-170O19.20; HOXA10 (margin: HOXA-AS4; HOXA11-AS; HOXA9; RP1-170O19.20; MIR196B; HOXA10; HOXA11; RP1-170O19.14)
 
  4.0015183
 
 6.22e-04
 
 6.71e-03
  
 
  
 12
 
   2800055
 
   2800500
 
  PDF   STATS   ENSEMBL   UCSC 
 
  446
 
  8
 
 9006
 
 gene;island
 
 0.648254
 
 0.9267918
 
 CACNA1C; CACNA1C-AS1 (margin: CACNA1C; CACNA1C-AS1)
 
  3.9057912
 
 6.22e-04
 
 6.71e-03
  
 
  
 1
 
 220101698
 
 220102043
 
  PDF   STATS   ENSEMBL   UCSC 
 
  346
 
  8
 
 4211
 
 island
 
 0.589472
 
 0.9491381
 
 SLC30A10 (margin: SLC30A10)
 
  3.8130696
 
 6.22e-04
 
 6.71e-03
  
 
  
 6
 
  32171786
 
  32172151
 
  PDF   STATS   ENSEMBL   UCSC 
 
  366
 
  8
 
 34088
 
 gene
 
 0.173519
 
 0.6745407
 
 NOTCH4 (margin: GPSM3; NOTCH4)
 
  3.8042780
 
 6.22e-04
 
 6.71e-03
  
 
  
 12
 
 114846162
 
 114846503
 
  PDF   STATS   ENSEMBL   UCSC 
 
  342
 
  8
 
 10841
 
 island
 
 0.585134
 
 0.9427640
 
 TBX5-AS1; TBX5 (margin: TBX5-AS1; TBX5)
 
  3.7389951
 
 6.22e-04
 
 6.71e-03
  
 
  
 8
 
  65492528
 
  65492792
 
  PDF   STATS   ENSEMBL   UCSC 
 
  265
 
  8
 
 39736
 
 tss
 
 0.164728
 
 0.7389047
 
 BHLHE22; RP11-21C4.1 (margin: BHLHE22; RP11-21C4.5; RP11-21C4.1; CYP7B1)
 
  3.7064367
 
 6.22e-04
 
 6.71e-03
  
 
  
 20
 
  29611652
 
  29611924
 
  PDF   STATS   ENSEMBL   UCSC 
 
  273
 
  8
 
 26069
 
 island
 
 0.591716
 
 0.2378722
 
 FRG1B (margin: FRG1B)
 
  2.0651053
 
 6.22e-04
 
 6.71e-03
  
 
  
 17
 
   6947288
 
   6947464
 
  PDF   STATS   ENSEMBL   UCSC 
 
  177
 
  8
 
 17060
 
 tss
 
 0.852850
 
 0.9159955
 
 SLC16A11 (margin: SLC16A13; SLC16A11)
 
  1.9872230
 
 6.22e-04
 
 6.71e-03
  
 
  
 1
 
 160039847
 
 160040667
 
  PDF   STATS   ENSEMBL   UCSC 
 
  821
 
 13
 
 3887
 
 island
 
 0.389606
 
 0.8538831
 
 RP11-536C5.2; KCNJ10 (margin: RP11-536C5.2; KCNJ9; KCNJ10)
 
  2.8295444
 
 6.28e-04
 
 6.77e-03
  
 
  
 6
 
  30881112
 
  30882094
 
  PDF   STATS   ENSEMBL   UCSC 
 
  983
 
 28
 
 34793
 
 tss
 
 0.877260
 
 0.0688374
 
 GTF2H4; VARS2 (margin: RN7SL175P; GTF2H4; VARS2)
 
  3.4158615
 
 6.32e-04
 
 6.81e-03
  
 
  
 6
 
  32805142
 
  32806489
 
  PDF   STATS   ENSEMBL   UCSC 
 
 1348
 
 25
 
 34146
 
 gene
 
 0.011911
 
 0.0657534
 
 TAP2 (margin: XXbac-BPG246D15.8; PSMB9; TAP2; PSMB8; TAP1)
 
  2.3344739
 
 6.49e-04
 
 6.98e-03
  
 
  
 14
 
 101192190
 
 101193038
 
  PDF   STATS   ENSEMBL   UCSC 
 
  849
 
 16
 
 12607
 
 tss;island
 
 0.889818
 
 0.9674755
 
 DLK1 (margin: DLK1)
 
  2.1059052
 
 6.53e-04
 
 7.01e-03
  
 
  
 12
 
  54392734
 
  54393725
 
  PDF   STATS   ENSEMBL   UCSC 
 
  992
 
 16
 
 9751
 
 tss;island
 
 0.880733
 
 0.6527668
 
 HOXC5; HOXC6; HOXC9; HOXC-AS1 (margin: HOXC10; HOXC5; HOXC6; MIR196A2; HOXC9; HOXC8; HOXC-AS2; HOXC-AS1)
 
  1.7735420
 
 6.53e-04
 
 7.01e-03
  
 
  
 3
 
  44753865
 
  44754587
 
  PDF   STATS   ENSEMBL   UCSC 
 
  723
 
 12
 
 29040
 
 island
 
 0.395013
 
 0.9762943
 
 ZNF502; SOCS5P3 (margin: ZNF502; SOCS5P3)
 
  6.0042473
 
 6.56e-04
 
 7.02e-03
  
 
  
 8
 
  11560299
 
  11560851
 
  PDF   STATS   ENSEMBL   UCSC 
 
  553
 
 12
 
 39552
 
 tss;island
 
 0.607764
 
 0.9647620
 
 GATA4 (margin: GATA4)
 
  4.0358663
 
 6.56e-04
 
 7.02e-03
  
 
  
 15
 
  81426347
 
  81426820
 
  PDF   STATS   ENSEMBL   UCSC 
 
  474
 
 10
 
 14378
 
 island
 
 0.830843
 
 0.9806614
 
 C15orf26 (margin: C15orf26)
 
  4.0267666
 
 7.25e-04
 
 7.74e-03
  
 
  
 5
 
 178017571
 
 178018186
 
  PDF   STATS   ENSEMBL   UCSC 
 
  616
 
 10
 
 32540
 
 tss
 
 0.713695
 
 0.9514401
 
 COL23A1 (margin: COL23A1; CLK4)
 
  3.5247105
 
 7.25e-04
 
 7.74e-03
  
 
  
 17
 
  65040527
 
  65040983
 
  PDF   STATS   ENSEMBL   UCSC 
 
  457
 
 10
 
 18677
 
 island
 
 0.666991
 
 0.9691731
 
 CACNG1 (margin: CACNG4; CACNG1; RP11-74H8.1)
 
  3.2149846
 
 7.25e-04
 
 7.74e-03
  
 
  
 19
 
   4534986
 
   4535582
 
  PDF   STATS   ENSEMBL   UCSC 
 
  597
 
 11
 
 21379
 
 island
 
 0.143006
 
 0.0099404
 
 PLIN5; CTB-50L17.14; LRG1 (margin: RN7SL121P; PLIN5; CTB-50L17.14; LRG1; SEMA6B)
 
  4.8463561
 
 7.65e-04
 
 8.13e-03
  
 
  
 10
 
 102821427
 
 102822508
 
  PDF   STATS   ENSEMBL   UCSC 
 
 1082
 
 11
 
 4722
 
 gene;island
 
 0.356010
 
 0.0405256
 
 KAZALD1 (margin: KAZALD1)
 
  3.6757117
 
 7.65e-04
 
 8.13e-03
  
 
  
 1
 
 156211409
 
 156212005
 
  PDF   STATS   ENSEMBL   UCSC 
 
  597
 
 11
 
 3848
 
 island
 
 0.448341
 
 0.8930823
 
 PMF1-BGLAP; PMF1; BGLAP; PAQR6 (margin: PMF1-BGLAP; PMF1; BGLAP; PAQR6; SMG5)
 
  2.8811946
 
 7.65e-04
 
 8.13e-03
  
 
  
 3
 
  49314370
 
  49314920
 
  PDF   STATS   ENSEMBL   UCSC 
 
  551
 
 11
 
 29113
 
 island
 
 0.948245
 
 0.9819486
 
 C3orf62; USP4 (margin: Y_RNA; MIR4271; C3orf62; USP4)
 
  1.5789868
 
 7.65e-04
 
 8.13e-03
  
 
  
 4
 
  13546145
 
  13546673
 
  PDF   STATS   ENSEMBL   UCSC 
 
  529
 
  9
 
 30304
 
 tss
 
 0.739958
 
 0.0170808
 
 NKX3-2; AC006445.8 (margin: NKX3-2; AC006445.8)
 
  8.1751491
 
 7.82e-04
 
 8.17e-03
  
 
  
 1
 
 215256254
 
 215256978
 
  PDF   STATS   ENSEMBL   UCSC 
 
  725
 
  9
 
 1009
 
 gene;island
 
 0.688374
 
 0.0197387
 
 KCNK2 (margin: KCNK2)
 
  7.7614381
 
 7.82e-04
 
 8.17e-03
  
 
  
 3
 
  48885314
 
  48885665
 
  PDF   STATS   ENSEMBL   UCSC 
 
  352
 
  9
 
 28308
 
 tss
 
 0.005971
 
 0.3737598
 
 PRKAR2A-AS1; PRKAR2A (margin: PRKAR2A-AS1; PRKAR2A; SLC25A20)
 
  6.2954724
 
 7.82e-04
 
 8.17e-03
  
 
  
 20
 
  62680986
 
  62681428
 
  PDF   STATS   ENSEMBL   UCSC 
 
  443
 
  9
 
 25909
 
 tss
 
 0.818539
 
 0.0133988
 
 TCEA2; ZNF512B; SOX18 (margin: LINC00176; TCEA2; RP13-152O15.5; ZNF512B; SOX18)
 
  6.2709974
 
 7.82e-04
 
 8.17e-03
  
 
  
 2
 
 189156874
 
 189157361
 
  PDF   STATS   ENSEMBL   UCSC 
 
  488
 
  9
 
 23777
 
 tss
 
 0.009169
 
 0.4616616
 
 GULP1 (margin: GULP1; MIR561; AC068718.1)
 
  6.2561123
 
 7.82e-04
 
 8.17e-03
  
 
  
 4
 
 154125208
 
 154125720
 
  PDF   STATS   ENSEMBL   UCSC 
 
  513
 
  9
 
 30152
 
 gene
 
 0.970704
 
 0.3660644
 
 TRIM2 (margin: TRIM2)
 
  5.2021548
 
 7.82e-04
 
 8.17e-03
  
 
  
 6
 
 168197410
 
 168198163
 
  PDF   STATS   ENSEMBL   UCSC 
 
  754
 
  9
 
 36648
 
 island
 
 0.573375
 
 0.9764320
 
 AL009178.1; C6orf123 (margin: AL009178.1; C6orf123)
 
  4.6306349
 
 7.82e-04
 
 8.17e-03
  
 
  
 11
 
  67418140
 
  67418405
 
  PDF   STATS   ENSEMBL   UCSC 
 
  266
 
  9
 
 7500
 
 tss
 
 0.073856
 
 0.6669093
 
 ACY3; AP003385.2 (margin: TBX10; ACY3; AP003385.2; ALDH3B2)
 
  3.6614251
 
 7.82e-04
 
 8.17e-03
  
 
  
 7
 
  75157345
 
  75157765
 
  PDF   STATS   ENSEMBL   UCSC 
 
  421
 
  9
 
 38585
 
 island
 
 0.008794
 
 0.0424094
 
 PMS2P3 (margin: PMS2P3; HIP1)
 
  3.0806746
 
 7.82e-04
 
 8.17e-03
  
 
  
 13
 
  27131484
 
  27131780
 
  PDF   STATS   ENSEMBL   UCSC 
 
  297
 
  9
 
 11355
 
 tss;island
 
 0.174481
 
 0.0117236
 
 WASF3 (margin: WASF3)
 
  3.0205005
 
 7.82e-04
 
 8.17e-03
  
 
  
 8
 
 101661767
 
 101662246
 
  PDF   STATS   ENSEMBL   UCSC 
 
  480
 
  9
 
 40498
 
 island
 
 0.879867
 
 0.9789012
 
 SNX31 (margin: SNX31)
 
  2.9506782
 
 7.82e-04
 
 8.17e-03
  
 
  
 1
 
   2984245
 
   2984869
 
  PDF   STATS   ENSEMBL   UCSC 
 
  625
 
  9
 
 1178
 
 tss
 
 0.904245
 
 0.9747394
 
 PRDM16; LINC00982 (margin: PRDM16; LINC00982; AL008733.1)
 
  2.6923439
 
 7.82e-04
 
 8.17e-03
  
 
  
 6
 
  31831340
 
  31831890
 
  PDF   STATS   ENSEMBL   UCSC 
 
  551
 
 16
 
 33975
 
 gene;tss;island
 
 0.083374
 
 0.0119657
 
 NEU1; SLC44A4 (margin: NEU1; SLC44A4)
 
  2.6828840
 
 7.70e-04
 
 8.17e-03
  
 
  
 2
 
  80531500
 
  80531898
 
  PDF   STATS   ENSEMBL   UCSC 
 
  399
 
  9
 
 23473
 
 tss
 
 0.805138
 
 0.4595089
 
 CTNNA2; LRRTM1 (margin: CTNNA2; LRRTM1)
 
  2.4155408
 
 7.82e-04
 
 8.17e-03
  
 
  
 15
 
  74218418
 
  74218780
 
  PDF   STATS   ENSEMBL   UCSC 
 
  363
 
  9
 
 13785
 
 tss
 
 0.881955
 
 0.9672099
 
 LOXL1; LOXL1-AS1 (margin: LOXL1; LOXL1-AS1)
 
  1.9535238
 
 7.82e-04
 
 8.17e-03
  
 
  
 6
 
  31744339
 
  31744962
 
  PDF   STATS   ENSEMBL   UCSC 
 
  624
 
 14
 
 33954
 
 gene
 
 0.010125
 
 0.6155159
 
 VWA7; VARS; Y_RNA (margin: MSH5; MSH5-SAPCD1; SAPCD1; SAPCD1-AS1; VWA7; VARS; Y_RNA)
 
  6.1139169
 
 7.94e-04
 
 8.28e-03
  
 
  
 7
 
 149569715
 
 149570367
 
  PDF   STATS   ENSEMBL   UCSC 
 
  653
 
 13
 
 37268
 
 gene;island
 
 0.508493
 
 0.0407944
 
 ATP6V0E2; ATP6V0E2-AS1 (margin: ZNF862; ATP6V0E2; ATP6V0E2-AS1)
 
  4.4254885
 
 7.95e-04
 
 8.28e-03
  
 
  
 6
 
  32974843
 
  32975360
 
  PDF   STATS   ENSEMBL   UCSC 
 
  518
 
 13
 
 34176
 
 gene;island
 
 0.181833
 
 0.7767439
 
 HLA-DOA (margin: HLA-DOA)
 
  3.5338658
 
 7.95e-04
 
 8.28e-03
  
 
  
 11
 
   2017362
 
   2018247
 
  PDF   STATS   ENSEMBL   UCSC 
 
  886
 
 13
 
 6443
 
 gene;island
 
 0.591597
 
 0.9376737
 
 H19 (margin: MRPL23; AC051649.6; MRPL23-AS1; H19)
 
  2.0081552
 
 7.95e-04
 
 8.28e-03
  
 
  
 6
 
  32805142
 
  32806699
 
  PDF   STATS   ENSEMBL   UCSC 
 
 1558
 
 31
 
 35928
 
 island
 
 0.013101
 
 0.0376443
 
 TAP2; PSMB8 (margin: XXbac-BPG246D15.8; PSMB9; TAP2; PSMB8; TAP1)
 
  1.7443138
 
 9.55e-04
 
 9.93e-03
  
 
  
 7
 
 150778724
 
 150779390
 
  PDF   STATS   ENSEMBL   UCSC 
 
  667
 
 14
 
 37287
 
 gene;tss;island
 
 0.156903
 
 0.6204752
 
 FASTK; TMUB1 (margin: SLC4A2; AGAP3; RP11-148K1.12; FASTK; TMUB1)
 
  3.5965996
 
 9.71e-04
 
 1.01e-02
  
 
  
 2
 
 239139911
 
 239141043
 
  PDF   STATS   ENSEMBL   UCSC 
 
 1133
 
 17
 
 23972
 
 tss;island
 
 0.764399
 
 0.9696189
 
 AC096574.4; AC016757.3 (margin: AC096574.4; AC016757.3; HES6; PER2)
 
  2.4241389
 
 1.00e-03
 
 1.04e-02
  
 
  
 1
 
  68515788
 
  68516453
 
  PDF   STATS   ENSEMBL   UCSC 
 
  666
 
 11
 
 574
 
 gene
 
 0.674010
 
 0.9782434
 
 GNG12-AS1; DIRAS3; ARL5AP3 (margin: GNG12-AS1; RP4-609E1.2; AL157407.1; DIRAS3; ARL5AP3)
 
  4.6684225
 
 1.04e-03
 
 1.08e-02
  
 
  
 8
 
 121824218
 
 121824929
 
  PDF   STATS   ENSEMBL   UCSC 
 
  712
 
 10
 
 40565
 
 island
 
 0.726989
 
 0.0076004
 
 RP11-713M15.2; SNTB1 (margin: RP11-713M15.2; SNTB1)
 
  8.1695590
 
 1.05e-03
 
 1.08e-02
  
 
  
 16
 
  66638210
 
  66638702
 
  PDF   STATS   ENSEMBL   UCSC 
 
  493
 
 10
 
 14782
 
 gene;island
 
 0.731925
 
 0.0094402
 
 CMTM3 (margin: CMTM3; CMTM4)
 
  6.8638786
 
 1.05e-03
 
 1.08e-02
  
 
  
 11
 
  94134446
 
  94135029
 
  PDF   STATS   ENSEMBL   UCSC 
 
  584
 
 10
 
 8737
 
 island
 
 0.593387
 
 0.9816101
 
 GPR83 (margin: GPR83)
 
  4.7090482
 
 1.05e-03
 
 1.08e-02
  
 
  
 21
 
  45705175
 
  45705742
 
  PDF   STATS   ENSEMBL   UCSC 
 
  568
 
 10
 
 26582
 
 tss;island
 
 0.569780
 
 0.9849700
 
 AIRE (margin: AIRE)
 
  4.6670543
 
 1.05e-03
 
 1.08e-02
  
 
  
 1
 
 156211409
 
 156211896
 
  PDF   STATS   ENSEMBL   UCSC 
 
  488
 
 10
 
 2011
 
 tss
 
 0.427327
 
 0.8915046
 
 PMF1-BGLAP; PMF1; BGLAP; PAQR6 (margin: PMF1-BGLAP; PMF1; BGLAP; PAQR6; SMG5)
 
  4.1823206
 
 1.05e-03
 
 1.08e-02
  
 
  
 10
 
  61122307
 
  61122716
 
  PDF   STATS   ENSEMBL   UCSC 
 
  410
 
 10
 
 5762
 
 island
 
 0.112351
 
 0.0065518
 
 FAM13C (margin: FAM13C)
 
  4.1062354
 
 1.05e-03
 
 1.08e-02
  
 
  
 19
 
  52408259
 
  52408520
 
  PDF   STATS   ENSEMBL   UCSC 
 
  262
 
  8
 
 21000
 
 tss
 
 0.039673
 
 0.9716082
 
 ZNF649 (margin: CTC-429C10.2; ZNF649)
 
  9.7552213
 
 1.09e-03
 
 1.10e-02
  
 
  
 3
 
  50604613
 
  50605269
 
  PDF   STATS   ENSEMBL   UCSC 
 
  657
 
  8
 
 29160
 
 island
 
 0.051019
 
 0.9641206
 
 HEMK1; C3orf18 (margin: HEMK1; C3orf18)
 
  8.7028634
 
 1.09e-03
 
 1.10e-02
  
 
  
 12
 
 116996773
 
 116997095
 
  PDF   STATS   ENSEMBL   UCSC 
 
  323
 
  8
 
 10038
 
 tss
 
 0.161729
 
 0.9691150
 
 MAP1LC3B2 (margin: RP11-809C9.2; MAP1LC3B2)
 
  7.0642926
 
 1.09e-03
 
 1.10e-02
  
 
  
 16
 
  58548873
 
  58549320
 
  PDF   STATS   ENSEMBL   UCSC 
 
  448
 
  8
 
 15275
 
 tss;island
 
 0.327962
 
 0.0138060
 
 NDRG4; SETD6 (margin: NDRG4; SETD6; CNOT1)
 
  6.5983735
 
 1.09e-03
 
 1.10e-02
  
 
  
 20
 
   1206682
 
   1206830
 
  PDF   STATS   ENSEMBL   UCSC 
 
  149
 
  8
 
 25934
 
 island
 
 0.232500
 
 0.0065549
 
 RAD21L1 (margin: RAD21L1)
 
  5.5791163
 
 1.09e-03
 
 1.10e-02
  
 
  
 11
 
  35160330
 
  35160892
 
  PDF   STATS   ENSEMBL   UCSC 
 
  563
 
  8
 
 8205
 
 island
 
 0.008193
 
 0.3351365
 
 CD44; RP4-607I7.1 (margin: CD44; AL356215.1; RP4-607I7.1)
 
  5.4132080
 
 1.09e-03
 
 1.10e-02
  
 
  
 2
 
 109745210
 
 109745841
 
  PDF   STATS   ENSEMBL   UCSC 
 
  632
 
  8
 
 22822
 
 gene;tss;island
 
 0.268846
 
 0.9219153
 
 SH3RF3; SH3RF3-AS1 (margin: SH3RF3; SH3RF3-AS1)
 
  5.2999522
 
 1.09e-03
 
 1.10e-02
  
 
  
 20
 
  30639705
 
  30640041
 
  PDF   STATS   ENSEMBL   UCSC 
 
  337
 
  8
 
 25671
 
 tss
 
 0.355977
 
 0.9478283
 
 RNA5SP482; HCK (margin: RNA5SP482; HCK)
 
  5.2951389
 
 1.09e-03
 
 1.10e-02
  
 
  
 11
 
  14913752
 
  14914012
 
  PDF   STATS   ENSEMBL   UCSC 
 
  261
 
  8
 
 7166
 
 tss
 
 0.149965
 
 0.0054751
 
 CYP2R1 (margin: CYP2R1)
 
  4.7214472
 
 1.09e-03
 
 1.10e-02
  
 
  
 7
 
 128828120
 
 128828599
 
  PDF   STATS   ENSEMBL   UCSC 
 
  480
 
  8
 
 37891
 
 tss
 
 0.541563
 
 0.9663942
 
 SMO (margin: SMO)
 
  4.5681574
 
 1.09e-03
 
 1.10e-02
  
 
  
 16
 
   8806359
 
   8806756
 
  PDF   STATS   ENSEMBL   UCSC 
 
  398
 
  8
 
 14652
 
 gene;tss
 
 0.358623
 
 0.9818402
 
 ABAT (margin: ABAT; RNU7-63P)
 
  4.4457766
 
 1.09e-03
 
 1.10e-02
  
 
  
 11
 
 134145889
 
 134146266
 
  PDF   STATS   ENSEMBL   UCSC 
 
  378
 
  8
 
 7832
 
 tss
 
 0.501605
 
 0.9746170
 
 GLB1L3 (margin: ACAD8; GLB1L3)
 
  4.3904018
 
 1.09e-03
 
 1.10e-02
  
 
  
 5
 
 133451237
 
 133451625
 
  PDF   STATS   ENSEMBL   UCSC 
 
  389
 
  8
 
 31750
 
 gene;island
 
 0.191375
 
 0.8483647
 
 TCF7 (margin: TCF7)
 
  4.2625115
 
 1.09e-03
 
 1.10e-02
  
 
  
 11
 
  69519088
 
  69519437
 
  PDF   STATS   ENSEMBL   UCSC 
 
  350
 
  8
 
 8596
 
 island
 
 0.284265
 
 0.6738857
 
 FGF19 (margin: FGF19)
 
  3.9288822
 
 1.09e-03
 
 1.10e-02
  
 
  
 20
 
    982623
 
    983130
 
  PDF   STATS   ENSEMBL   UCSC 
 
  508
 
  8
 
 25931
 
 island
 
 0.722470
 
 0.9609961
 
 RSPO4 (margin: RSPO4)
 
  3.4485928
 
 1.09e-03
 
 1.10e-02
  
 
  
 2
 
 222436947
 
 222437130
 
  PDF   STATS   ENSEMBL   UCSC 
 
  184
 
  8
 
 25140
 
 island
 
 0.052708
 
 0.0070246
 
 EPHA4 (margin: EPHA4)
 
  3.3081730
 
 1.09e-03
 
 1.10e-02
  
 
  
 6
 
 122931154
 
 122931308
 
  PDF   STATS   ENSEMBL   UCSC 
 
  155
 
  8
 
 35206
 
 tss
 
 0.112124
 
 0.0146302
 
 PKIB (margin: PKIB)
 
  2.8700904
 
 1.09e-03
 
 1.10e-02
  
 
  
 1
 
 179711859
 
 179712281
 
  PDF   STATS   ENSEMBL   UCSC 
 
  423
 
  8
 
 2120
 
 tss
 
 0.767768
 
 0.9468408
 
 RP11-12M5.1; FAM163A (margin: RP11-12M5.1; FAM163A)
 
  2.4880758
 
 1.09e-03
 
 1.10e-02
  
 
  
 10
 
 102893925
 
 102894639
 
  PDF   STATS   ENSEMBL   UCSC 
 
  715
 
  8
 
 4724
 
 gene;island
 
 0.901336
 
 0.9777325
 
 TLX1; RP11-31L23.3 (margin: HUG1; TLX1; TLX1NB; RP11-31L23.3)
 
  2.4263353
 
 1.09e-03
 
 1.10e-02
  
 
  
 10
 
 119000927
 
 119001590
 
  PDF   STATS   ENSEMBL   UCSC 
 
  664
 
  8
 
 4778
 
 gene;island
 
 0.773323
 
 0.4115058
 
 SLC18A2; RP11-501J20.5 (margin: SLC18A2; RP11-501J20.5)
 
  2.2312833
 
 1.09e-03
 
 1.10e-02
  
 
  
 2
 
 162283434
 
 162284206
 
  PDF   STATS   ENSEMBL   UCSC 
 
  773
 
  8
 
 24823
 
 island
 
 0.902938
 
 0.9760359
 
 TBR1; AC009487.5; RP11-334E15.2 (margin: TBR1; AC009487.5; RP11-334E15.2; AC009487.4)
 
  1.9732583
 
 1.09e-03
 
 1.10e-02
  
 
  
 19
 
  54023732
 
  54024435
 
  PDF   STATS   ENSEMBL   UCSC 
 
  704
 
 12
 
 22380
 
 island
 
 0.727632
 
 0.0056978
 
 ZNF331 (margin: CTD-2224J9.8; ZNF331)
 
  6.5935356
 
 1.11e-03
 
 1.12e-02
  
 
  
 19
 
  11689488
 
  11689997
 
  PDF   STATS   ENSEMBL   UCSC 
 
  510
 
 12
 
 20477
 
 tss;island
 
 0.876010
 
 0.9815943
 
 ZNF627; ACP5 (margin: ZNF627; AC020947.2; ACP5)
 
  2.6006833
 
 1.11e-03
 
 1.12e-02
  
 
  
 17
 
  35290307
 
  35291580
 
  PDF   STATS   ENSEMBL   UCSC 
 
 1274
 
 12
 
 18235
 
 island
 
 0.896785
 
 0.9805246
 
 RP11-445F12.1 (margin: LHX1; RP11-445F12.1; RP11-445F12.2)
 
  2.4688491
 
 1.11e-03
 
 1.12e-02
  
 
  
 8
 
  41167660
 
  41168481
 
  PDF   STATS   ENSEMBL   UCSC 
 
  822
 
 12
 
 39668
 
 tss;island
 
 0.618888
 
 0.9265213
 
 SFRP1 (margin: RNU6-895P; SFRP1)
 
  2.3925175
 
 1.11e-03
 
 1.12e-02
  
 
  
 11
 
 106889318
 
 106889745
 
  PDF   STATS   ENSEMBL   UCSC 
 
  428
 
  7
 
 7659
 
 tss;island
 
 0.386764
 
 0.0047994
 
 GUCY1A2 (margin: GUCY1A2)
 
  8.7604311
 
 1.17e-03
 
 1.13e-02
  
 
  
 8
 
  22926785
 
  22926911
 
  PDF   STATS   ENSEMBL   UCSC 
 
  127
 
  7
 
 39595
 
 tss
 
 0.015330
 
 0.4967274
 
 RP11-875O11.2; TNFRSF10B; RP11-875O11.3 (margin: RP11-875O11.2; TNFRSF10B; RP11-875O11.3)
 
  7.8708313
 
 1.17e-03
 
 1.13e-02
  
 
  
 8
 
  70747067
 
  70747646
 
  PDF   STATS   ENSEMBL   UCSC 
 
  580
 
  7
 
 40372
 
 island
 
 0.394780
 
 0.0040725
 
 RP11-159H10.3; SLCO5A1 (margin: RP11-159H10.3; SLCO5A1)
 
  7.3185943
 
 1.17e-03
 
 1.13e-02
  
 
  
 12
 
  51818350
 
  51818768
 
  PDF   STATS   ENSEMBL   UCSC 
 
  419
 
  7
 
 10427
 
 island
 
 0.466417
 
 0.0086969
 
 SLC4A8; RP11-607P23.1 (margin: SLC4A8; RP11-607P23.1)
 
  7.0790064
 
 1.17e-03
 
 1.13e-02
  
 
  
 5
 
 174151043
 
 174151567
 
  PDF   STATS   ENSEMBL   UCSC 
 
  525
 
  7
 
 32505
 
 tss
 
 0.355032
 
 0.0035988
 
 MSX2 (margin: MSX2)
 
  6.8645741
 
 1.17e-03
 
 1.13e-02
  
 
  
 6
 
  55444061
 
  55444488
 
  PDF   STATS   ENSEMBL   UCSC 
 
  428
 
  7
 
 35043
 
 tss
 
 0.680507
 
 0.0529852
 
 HMGCLL1 (margin: HMGCLL1)
 
  6.6212719
 
 1.17e-03
 
 1.13e-02
  
 
  
 21
 
  39288720
 
  39289266
 
  PDF   STATS   ENSEMBL   UCSC 
 
  547
 
  7
 
 26544
 
 tss
 
 0.575433
 
 0.0334113
 
 KCNJ6 (margin: KCNJ6)
 
  6.2479699
 
 1.17e-03
 
 1.13e-02
  
 
  
 16
 
  30022548
 
  30022725
 
  PDF   STATS   ENSEMBL   UCSC 
 
  178
 
  7
 
 15175
 
 tss;island
 
 0.279049
 
 0.0021616
 
 DOC2A (margin: INO80E; C16orf92; DOC2A)
 
  6.2467449
 
 1.17e-03
 
 1.13e-02
  
 
  
 7
 
   1607146
 
   1607868
 
  PDF   STATS   ENSEMBL   UCSC 
 
  723
 
  7
 
 36762
 
 gene;island
 
 0.340174
 
 0.9605404
 
 PSMG3-AS1; PSMG3 (margin: PSMG3-AS1; TMEM184A; PSMG3)
 
  6.1331925
 
 1.17e-03
 
 1.13e-02
  
 
  
 2
 
 119532041
 
 119532574
 
  PDF   STATS   ENSEMBL   UCSC 
 
  534
 
  7
 
 24643
 
 island
 
 0.008394
 
 0.2969521
 
  (margin: )
 
  5.7296002
 
 1.17e-03
 
 1.13e-02
  
 
  
 17
 
  45772333
 
  45772800
 
  PDF   STATS   ENSEMBL   UCSC 
 
  468
 
  7
 
 18457
 
 island
 
 0.345098
 
 0.9724947
 
 TBKBP1 (margin: KPNB1; TBKBP1)
 
  5.3759122
 
 1.17e-03
 
 1.13e-02
  
 
  
 1
 
 201915757
 
 201915999
 
  PDF   STATS   ENSEMBL   UCSC 
 
  243
 
  7
 
 2170
 
 tss
 
 0.453973
 
 0.0211864
 
 LMOD1 (margin: TIMM17A; LMOD1)
 
  5.1933885
 
 1.17e-03
 
 1.13e-02
  
 
  
 5
 
  65891878
 
  65892109
 
  PDF   STATS   ENSEMBL   UCSC 
 
  232
 
  7
 
 32101
 
 tss
 
 0.316111
 
 0.0094492
 
 MAST4 (margin: MAST4)
 
  5.1578905
 
 1.17e-03
 
 1.13e-02
  
 
  
 7
 
  35077704
 
  35077807
 
  PDF   STATS   ENSEMBL   UCSC 
 
  104
 
  7
 
 37534
 
 tss
 
 0.198062
 
 0.0059631
 
 DPY19L1 (margin: DPY19L1)
 
  5.0201509
 
 1.17e-03
 
 1.13e-02
  
 
  
 10
 
 122216135
 
 122216378
 
  PDF   STATS   ENSEMBL   UCSC 
 
  244
 
  7
 
 5403
 
 tss;island
 
 0.279159
 
 0.8982329
 
 PPAPDC1A (margin: PPAPDC1A; AC073587.1)
 
  4.9948440
 
 1.17e-03
 
 1.13e-02
  
 
  
 20
 
  57464970
 
  57465175
 
  PDF   STATS   ENSEMBL   UCSC 
 
  206
 
  7
 
 25474
 
 gene;tss;island
 
 0.573327
 
 0.9695203
 
 GNAS; RP1-309F20.3 (margin: GNAS; RP1-309F20.3)
 
  4.9547162
 
 1.17e-03
 
 1.13e-02
  
 
  
 11
 
  45687263
 
  45687494
 
  PDF   STATS   ENSEMBL   UCSC 
 
  232
 
  7
 
 7265
 
 tss;island
 
 0.256939
 
 0.0130351
 
 CHST1 (margin: CHST1)
 
  4.8790166
 
 1.17e-03
 
 1.13e-02
  
 
  
 1
 
 224804053
 
 224804396
 
  PDF   STATS   ENSEMBL   UCSC 
 
  344
 
  7
 
 4244
 
 island
 
 0.569051
 
 0.0285007
 
 CNIH3; RP11-100E13.1 (margin: CNIH3; RP11-100E13.1)
 
  4.8773004
 
 1.17e-03
 
 1.13e-02
  
 
  
 7
 
  64734093
 
  64734749
 
  PDF   STATS   ENSEMBL   UCSC 
 
  657
 
  7
 
 38527
 
 island
 
 0.275941
 
 0.0133190
 
  (margin: AC092685.1)
 
  4.8416096
 
 1.17e-03
 
 1.13e-02
  
 
  
 3
 
  46853850
 
  46854311
 
  PDF   STATS   ENSEMBL   UCSC 
 
  462
 
  7
 
 29061
 
 island
 
 0.361665
 
 0.9669160
 
 PRSS50; PRSS45; PRSS44 (margin: PRSS50; PRSS45; PRSS44)
 
  4.7731569
 
 1.17e-03
 
 1.13e-02
  
 
  
 5
 
 133451237
 
 133451474
 
  PDF   STATS   ENSEMBL   UCSC 
 
  238
 
  7
 
 32287
 
 tss
 
 0.090258
 
 0.8148762
 
 TCF7 (margin: TCF7)
 
  4.7164784
 
 1.17e-03
 
 1.13e-02
  
 
  
 6
 
  10886946
 
  10887047
 
  PDF   STATS   ENSEMBL   UCSC 
 
  102
 
  7
 
 34633
 
 tss
 
 0.351650
 
 0.9718615
 
 RP11-637O19.3; SYCP2L (margin: RP11-637O19.3; SYCP2L; RP11-637O19.2; GCM2)
 
  4.6261817
 
 1.17e-03
 
 1.13e-02
  
 
  
 20
 
   3776212
 
   3776357
 
  PDF   STATS   ENSEMBL   UCSC 
 
  146
 
  7
 
 25568
 
 tss
 
 0.618304
 
 0.0632335
 
 CDC25B (margin: CDC25B; CENPB)
 
  4.5871402
 
 1.17e-03
 
 1.13e-02
  
 
  
 18
 
  32556626
 
  32557027
 
  PDF   STATS   ENSEMBL   UCSC 
 
  402
 
  7
 
 19231
 
 tss;island
 
 0.147140
 
 0.0091926
 
 MAPRE2 (margin: MAPRE2)
 
  4.5345244
 
 1.17e-03
 
 1.13e-02
  
 
  
 7
 
 107302255
 
 107302510
 
  PDF   STATS   ENSEMBL   UCSC 
 
  256
 
  7
 
 37183
 
 gene;tss;island
 
 0.434857
 
 0.0256667
 
 SLC26A4; SLC26A4-AS1 (margin: SLC26A4; SLC26A4-AS1)
 
  4.5321668
 
 1.17e-03
 
 1.13e-02
  
 
  
 11
 
 110583377
 
 110583662
 
  PDF   STATS   ENSEMBL   UCSC 
 
  286
 
  7
 
 8794
 
 island
 
 0.458990
 
 0.9068867
 
 ARHGAP20 (margin: ARHGAP20)
 
  3.9103233
 
 1.17e-03
 
 1.13e-02
  
 
  
 4
 
   5053085
 
   5053596
 
  PDF   STATS   ENSEMBL   UCSC 
 
  512
 
  7
 
 30877
 
 island
 
 0.486009
 
 0.9755138
 
 STK32B (margin: STK32B)
 
  3.7674644
 
 1.17e-03
 
 1.13e-02
  
 
  
 1
 
   3056542
 
   3056951
 
  PDF   STATS   ENSEMBL   UCSC 
 
  410
 
  7
 
 102
 
 gene;island
 
 0.133823
 
 0.7320669
 
 PRDM16 (margin: PRDM16; RP1-163G9.2)
 
  3.6944645
 
 1.17e-03
 
 1.13e-02
  
 
  
 8
 
  24771114
 
  24771645
 
  PDF   STATS   ENSEMBL   UCSC 
 
  532
 
  7
 
 39604
 
 tss;island
 
 0.365215
 
 0.8692471
 
 NEFM; RP11-624C23.1; GS1-72M22.1 (margin: NEFM; RP11-624C23.1; GS1-72M22.1)
 
  3.6367626
 
 1.17e-03
 
 1.13e-02
  
 
  
 1
 
  50889128
 
  50889510
 
  PDF   STATS   ENSEMBL   UCSC 
 
  383
 
  7
 
 3253
 
 island
 
 0.678901
 
 0.9769999
 
 DMRTA2 (margin: DMRTA2)
 
  3.6187851
 
 1.17e-03
 
 1.13e-02
  
 
  
 11
 
   7695165
 
   7695528
 
  PDF   STATS   ENSEMBL   UCSC 
 
  364
 
  7
 
 7121
 
 tss;island
 
 0.527611
 
 0.9578135
 
 CYB5R2 (margin: CYB5R2)
 
  3.5767226
 
 1.17e-03
 
 1.13e-02
  
 
  
 3
 
 131080235
 
 131080697
 
  PDF   STATS   ENSEMBL   UCSC 
 
  463
 
  7
 
 28593
 
 tss
 
 0.456731
 
 0.8881090
 
 NUDT16P; RP11-933H2.4 (margin: NEK11; NUDT16P; RP11-933H2.4)
 
  3.5440313
 
 1.17e-03
 
 1.13e-02
  
 
  
 7
 
   1248693
 
   1249199
 
  PDF   STATS   ENSEMBL   UCSC 
 
  507
 
  7
 
 38088
 
 island
 
 0.591919
 
 0.9799957
 
  (margin: )
 
  3.5405806
 
 1.17e-03
 
 1.13e-02
  
 
  
 12
 
  49582874
 
  49583112
 
  PDF   STATS   ENSEMBL   UCSC 
 
  239
 
  7
 
 9684
 
 tss
 
 0.091980
 
 0.0081497
 
 TUBA1C; TUBA1A (margin: TUBA1C; TUBA1A)
 
  3.5038815
 
 1.17e-03
 
 1.13e-02
  
 
  
 1
 
 182361258
 
 182361557
 
  PDF   STATS   ENSEMBL   UCSC 
 
  300
 
  7
 
 2131
 
 tss;island
 
 0.311984
 
 0.7256653
 
 GLUL (margin: GLUL; TEDDM1)
 
  2.9075941
 
 1.17e-03
 
 1.13e-02
  
 
  
 11
 
  60623415
 
  60623918
 
  PDF   STATS   ENSEMBL   UCSC 
 
  504
 
  7
 
 8314
 
 island
 
 0.584662
 
 0.8571711
 
 PTGDR2 (margin: CCDC86; ZP1; RP11-804A23.4; PTGDR2)
 
  1.8936389
 
 1.17e-03
 
 1.13e-02
  
 
  
 2
 
  61371880
 
  61372226
 
  PDF   STATS   ENSEMBL   UCSC 
 
  347
 
  7
 
 23377
 
 tss
 
 0.413036
 
 0.6706098
 
 KIAA1841; C2orf74; AC016747.3 (margin: KIAA1841; C2orf74; AC016747.3; RP11-493E12.1)
 
  1.6921036
 
 1.17e-03
 
 1.13e-02
  
 
  
 6
 
  33180841
 
  33181565
 
  PDF   STATS   ENSEMBL   UCSC 
 
  725
 
 14
 
 35985
 
 island
 
 0.101880
 
 0.4363605
 
 RING1; ZNF70P1 (margin: SLC39A7; HSD17B8; MIR219-1; RING1; ZNF70P1)
 
  3.0166835
 
 1.18e-03
 
 1.14e-02
  
 
  
 17
 
  46671131
 
  46671635
 
  PDF   STATS   ENSEMBL   UCSC 
 
  505
 
  9
 
 17464
 
 tss
 
 0.493760
 
 0.0132634
 
 HOXB-AS3; HOXB3; HOXB5; HOXB6 (margin: HOXB-AS3; HOXB3; MIR10A; HOXB5; HOXB6)
 
  6.6870025
 
 1.23e-03
 
 1.18e-02
  
 
  
 8
 
  17433926
 
  17434541
 
  PDF   STATS   ENSEMBL   UCSC 
 
  616
 
  9
 
 39567
 
 tss;island
 
 0.559855
 
 0.0115972
 
 PDGFRL (margin: SLC7A2; PDGFRL)
 
  6.4864178
 
 1.23e-03
 
 1.18e-02
  
 
  
 17
 
   7197775
 
   7198087
 
  PDF   STATS   ENSEMBL   UCSC 
 
  313
 
  9
 
 17933
 
 island
 
 0.691478
 
 0.0080068
 
 YBX2 (margin: SLC2A4; RP1-4G17.2; YBX2)
 
  5.8227323
 
 1.23e-03
 
 1.18e-02
  
 
  
 6
 
  33140660
 
  33141305
 
  PDF   STATS   ENSEMBL   UCSC 
 
  646
 
  9
 
 34201
 
 gene
 
 0.029186
 
 0.6116818
 
 COL11A2 (margin: COL11A2)
 
  5.5793089
 
 1.23e-03
 
 1.18e-02
  
 
  
 4
 
  25031951
 
  25032564
 
  PDF   STATS   ENSEMBL   UCSC 
 
  614
 
  9
 
 30957
 
 island
 
 0.406595
 
 0.0107021
 
 LGI2 (margin: LGI2)
 
  4.5868587
 
 1.23e-03
 
 1.18e-02
  
 
  
 20
 
  42875645
 
  42876091
 
  PDF   STATS   ENSEMBL   UCSC 
 
  447
 
  9
 
 26180
 
 island
 
 0.579934
 
 0.9127644
 
 GDAP1L1 (margin: GDAP1L1)
 
  4.0687582
 
 1.23e-03
 
 1.18e-02
  
 
  
 1
 
   2410885
 
   2411643
 
  PDF   STATS   ENSEMBL   UCSC 
 
  759
 
  9
 
 85
 
 gene;island
 
 0.600498
 
 0.9657119
 
 PLCH2 (margin: PLCH2)
 
  3.1503116
 
 1.23e-03
 
 1.18e-02
  
 
  
 11
 
 107798913
 
 107799237
 
  PDF   STATS   ENSEMBL   UCSC 
 
  325
 
  9
 
 7666
 
 tss
 
 0.311595
 
 0.0290982
 
 RAB39A; SLC35F2 (margin: RAB39A; SLC35F2)
 
  2.9680118
 
 1.23e-03
 
 1.18e-02
  
 
  
 11
 
  30038615
 
  30038797
 
  PDF   STATS   ENSEMBL   UCSC 
 
  183
 
  9
 
 7218
 
 tss;island
 
 0.870632
 
 0.5149750
 
 KCNA4 (margin: KCNA4)
 
  2.8774709
 
 1.23e-03
 
 1.18e-02
  
 
  
 1
 
  24645802
 
  24646392
 
  PDF   STATS   ENSEMBL   UCSC 
 
  591
 
 13
 
 2858
 
 island
 
 0.068427
 
 0.0060429
 
 GRHL3; RP11-10N16.3 (margin: GRHL3; RP11-10N16.3)
 
  3.3994135
 
 1.25e-03
 
 1.19e-02
  
 
  
 14
 
 101359793
 
 101360236
 
  PDF   STATS   ENSEMBL   UCSC 
 
  444
 
 11
 
 12615
 
 tss
 
 0.395317
 
 0.9595553
 
 AL117190.3; MEG8 (margin: MIR433; MIR127; MIR432; MIR136; AL117190.3; MEG8; AL117190.1; RTL1)
 
  4.9032720
 
 1.40e-03
 
 1.33e-02
  
 
  
 12
 
 103889516
 
 103890274
 
  PDF   STATS   ENSEMBL   UCSC 
 
  759
 
 11
 
 10731
 
 island
 
 0.443048
 
 0.0464859
 
 C12orf42 (margin: RP11-626I20.1; C12orf42; RP11-626I20.2)
 
  3.9264646
 
 1.40e-03
 
 1.33e-02
  
 
  
 10
 
 102976327
 
 102977342
 
  PDF   STATS   ENSEMBL   UCSC 
 
 1016
 
 11
 
 6056
 
 island
 
 0.880302
 
 0.9740146
 
  (margin: LBX1)
 
  2.1384022
 
 1.40e-03
 
 1.33e-02
  
 
  
 3
 
  10805956
 
  10806569
 
  PDF   STATS   ENSEMBL   UCSC 
 
  614
 
 11
 
 28152
 
 tss
 
 0.754395
 
 0.9415629
 
 LINC00606 (margin: AC018495.3; LINC00606)
 
  2.1346560
 
 1.40e-03
 
 1.33e-02
  
 
  
 10
 
 130339134
 
 130339927
 
  PDF   STATS   ENSEMBL   UCSC 
 
  794
 
 12
 
 6256
 
 island
 
 0.886813
 
 0.0308424
 
  (margin: )
 
  2.4391954
 
 1.43e-03
 
 1.36e-02
  
 
  
 1
 
 229569608
 
 229570288
 
  PDF   STATS   ENSEMBL   UCSC 
 
  681
 
 14
 
 4310
 
 island
 
 0.774712
 
 0.9306026
 
 ACTA1 (margin: RP5-1068B5.1; ACTA1; RP5-1068B5.3; NUP133)
 
  2.2620388
 
 1.43e-03
 
 1.36e-02
  
 
  
 16
 
  82660206
 
  82660873
 
  PDF   STATS   ENSEMBL   UCSC 
 
  668
 
 14
 
 16129
 
 island
 
 0.195635
 
 0.4330187
 
 CDH13 (margin: CDH13)
 
  1.4481609
 
 1.43e-03
 
 1.36e-02
  
 
  
 5
 
  35617687
 
  35618055
 
  PDF   STATS   ENSEMBL   UCSC 
 
  369
 
 10
 
 32728
 
 island
 
 0.507961
 
 0.0060583
 
 SPEF2 (margin: SPEF2)
 
  7.3631445
 
 1.50e-03
 
 1.41e-02
  
 
  
 4
 
 110223830
 
 110224288
 
  PDF   STATS   ENSEMBL   UCSC 
 
  459
 
 10
 
 30542
 
 tss
 
 0.765990
 
 0.0597673
 
 AC004051.2; COL25A1 (margin: AC004051.2; COL25A1)
 
  5.7217420
 
 1.50e-03
 
 1.41e-02
  
 
  
 6
 
  94129234
 
  94129697
 
  PDF   STATS   ENSEMBL   UCSC 
 
  464
 
 10
 
 36331
 
 island
 
 0.865728
 
 0.0776282
 
 EPHA7 (margin: EPHA7)
 
  4.9533761
 
 1.50e-03
 
 1.41e-02
  
 
  
 3
 
  45837898
 
  45838338
 
  PDF   STATS   ENSEMBL   UCSC 
 
  441
 
 10
 
 29051
 
 island
 
 0.671566
 
 0.9812578
 
 SLC6A20 (margin: SLC6A20)
 
  4.6098937
 
 1.50e-03
 
 1.41e-02
  
 
  
 6
 
  30043727
 
  30044259
 
  PDF   STATS   ENSEMBL   UCSC 
 
  533
 
 10
 
 34763
 
 tss
 
 0.044666
 
 0.5319958
 
 RNF39 (margin: ZNRD1; PPP1R11; RNF39)
 
  4.2215702
 
 1.50e-03
 
 1.41e-02
  
 
  
 11
 
  46354562
 
  46355283
 
  PDF   STATS   ENSEMBL   UCSC 
 
  722
 
 10
 
 8246
 
 island
 
 0.771264
 
 0.9841608
 
 DGKZ (margin: CREB3L1; DGKZ)
 
  3.7025008
 
 1.50e-03
 
 1.41e-02
  
 
  
 1
 
   3663164
 
   3664084
 
  PDF   STATS   ENSEMBL   UCSC 
 
  921
 
 10
 
 2570
 
 island
 
 0.629121
 
 0.9583716
 
 TP73-AS1 (margin: TP73; CCDC27; TP73-AS1)
 
  3.5424774
 
 1.50e-03
 
 1.41e-02
  
 
  
 6
 
 130339410
 
 130339998
 
  PDF   STATS   ENSEMBL   UCSC 
 
  589
 
 10
 
 36475
 
 island
 
 0.112041
 
 0.5343909
 
 L3MBTL3 (margin: L3MBTL3)
 
  3.1993005
 
 1.50e-03
 
 1.41e-02
  
 
  
 2
 
 160919147
 
 160919532
 
  PDF   STATS   ENSEMBL   UCSC 
 
  386
 
 10
 
 23684
 
 tss
 
 0.082234
 
 0.4301947
 
 PLA2R1 (margin: PLA2R1)
 
  3.0141029
 
 1.50e-03
 
 1.41e-02
  
 
  
 2
 
  24232603
 
  24232891
 
  PDF   STATS   ENSEMBL   UCSC 
 
  289
 
 10
 
 23220
 
 tss
 
 0.923183
 
 0.9855031
 
 MFSD2B (margin: UBXN2A; MFSD2B)
 
  2.5561301
 
 1.50e-03
 
 1.41e-02
  
 
  
 1
 
 153650884
 
 153651325
 
  PDF   STATS   ENSEMBL   UCSC 
 
  442
 
 10
 
 3767
 
 island
 
 0.092802
 
 0.4494201
 
 NPR1 (margin: NPR1; ILF2)
 
  2.2375957
 
 1.50e-03
 
 1.41e-02
  
 
  
 8
 
  55370192
 
  55370579
 
  PDF   STATS   ENSEMBL   UCSC 
 
  388
 
 10
 
 40302
 
 island
 
 0.862163
 
 0.9723743
 
 SOX17 (margin: SOX17)
 
  1.8975168
 
 1.50e-03
 
 1.41e-02
  
 
  
 16
 
    699218
 
    699500
 
  PDF   STATS   ENSEMBL   UCSC 
 
  283
 
  6
 
 15468
 
 island
 
 0.003816
 
 0.9878054
 
 FAM195A; WDR90; AL022341.3 (margin: FAM195A; WDR90; AL022341.1; AL022341.3; LA16c-349E10.1)
 
 16.6350052
 
 2.16e-03
 
 1.45e-02
  
 
  
 17
 
  46682308
 
  46682413
 
  PDF   STATS   ENSEMBL   UCSC 
 
  106
 
  6
 
 16657
 
 gene;island
 
 0.001299
 
 0.9855877
 
 HOXB-AS3; HOXB3; HOXB6 (margin: HOXB-AS3; HOXB3; HOXB5; HOXB6; HOXB7; HOXB8)
 
 15.6182810
 
 2.16e-03
 
 1.45e-02
  
 
  
 1
 
  77332991
 
  77333159
 
  PDF   STATS   ENSEMBL   UCSC 
 
  169
 
  6
 
 1703
 
 tss
 
 0.977517
 
 0.0063733
 
 ST6GALNAC5 (margin: ST6GALNAC5)
 
 14.2419421
 
 2.16e-03
 
 1.45e-02
  
 
  
 15
 
  41061984
 
  41062273
 
  PDF   STATS   ENSEMBL   UCSC 
 
  290
 
  6
 
 13296
 
 gene
 
 0.001681
 
 0.9630811
 
 C15orf62; DNAJC17 (margin: RP11-532F12.6; GCHFR; C15orf62; DNAJC17)
 
 14.1106710
 
 2.16e-03
 
 1.45e-02
  
 
  
 2
 
  85640762
 
  85641254
 
  PDF   STATS   ENSEMBL   UCSC 
 
  493
 
  6
 
 24478
 
 island
 
 0.001772
 
 0.9841504
 
 CAPG (margin: SH2D6; CAPG)
 
 13.8414291
 
 2.16e-03
 
 1.45e-02
  
 
  
 16
 
  57836242
 
  57836706
 
  PDF   STATS   ENSEMBL   UCSC 
 
  465
 
  6
 
 15969
 
 island
 
 0.008506
 
 0.9793310
 
 CTD-2600O9.1; KIFC3 (margin: CTD-2600O9.1; KIFC3)
 
 13.6520524
 
 2.16e-03
 
 1.45e-02
  
 
  
 5
 
    912383
 
    912860
 
  PDF   STATS   ENSEMBL   UCSC 
 
  478
 
  6
 
 31516
 
 gene;island
 
 0.008493
 
 0.9849223
 
 TRIP13 (margin: TRIP13)
 
 13.2945196
 
 2.16e-03
 
 1.45e-02
  
 
  
 18
 
  44526430
 
  44526756
 
  PDF   STATS   ENSEMBL   UCSC 
 
  327
 
  6
 
 19260
 
 tss
 
 0.960910
 
 0.0028311
 
 KATNAL2 (margin: KATNAL2)
 
 13.0292034
 
 2.16e-03
 
 1.45e-02
  
 
  
 21
 
  26934197
 
  26934682
 
  PDF   STATS   ENSEMBL   UCSC 
 
  486
 
  6
 
 26615
 
 island
 
 0.005765
 
 0.9756968
 
 MIR155HG (margin: MIR155HG)
 
 12.8661086
 
 2.16e-03
 
 1.45e-02
  
 
  
 11
 
  33037530
 
  33037916
 
  PDF   STATS   ENSEMBL   UCSC 
 
  387
 
  6
 
 6572
 
 gene
 
 0.013750
 
 0.9839290
 
 DEPDC7 (margin: DEPDC7; Y_RNA)
 
 12.5193040
 
 2.16e-03
 
 1.45e-02
  
 
  
 6
 
 125284203
 
 125284359
 
  PDF   STATS   ENSEMBL   UCSC 
 
  157
 
  6
 
 35208
 
 tss;island
 
 0.010135
 
 0.9854241
 
 RNF217; RP11-510H23.1 (margin: RNF217; RP11-510H23.1)
 
 12.4331053
 
 2.16e-03
 
 1.45e-02
  
 
  
 20
 
  57581903
 
  57582292
 
  PDF   STATS   ENSEMBL   UCSC 
 
  390
 
  6
 
 25478
 
 gene
 
 0.008945
 
 0.9825091
 
 CTSZ (margin: NELFCD; CTSZ)
 
 12.4127193
 
 2.16e-03
 
 1.45e-02
  
 
  
 7
 
  92466078
 
  92466386
 
  PDF   STATS   ENSEMBL   UCSC 
 
  309
 
  6
 
 37711
 
 tss;island
 
 0.007206
 
 0.9649786
 
 AC002454.1; CDK6 (margin: AC002454.1; CDK6)
 
 11.9814073
 
 2.16e-03
 
 1.45e-02
  
 
  
 5
 
  11904015
 
  11904127
 
  PDF   STATS   ENSEMBL   UCSC 
 
  113
 
  6
 
 32695
 
 island
 
 0.949890
 
 0.0160610
 
 CTNND2 (margin: CTNND2)
 
 11.7775888
 
 2.16e-03
 
 1.45e-02
  
 
  
 3
 
  48507354
 
  48507618
 
  PDF   STATS   ENSEMBL   UCSC 
 
  265
 
  6
 
 27767
 
 gene
 
 0.000825
 
 0.7963948
 
 ATRIP; TREX1; SHISA5 (margin: ATRIP; TREX1; SHISA5)
 
 11.7725526
 
 2.16e-03
 
 1.45e-02
  
 
  
 22
 
  50968250
 
  50968516
 
  PDF   STATS   ENSEMBL   UCSC 
 
  267
 
  6
 
 27629
 
 island
 
 0.012538
 
 0.9759185
 
 TYMP; ODF3B (margin: NCAPH2; SCO2; TYMP; ODF3B)
 
 11.4475702
 
 2.16e-03
 
 1.45e-02
  
 
  
 14
 
  23834860
 
  23834995
 
  PDF   STATS   ENSEMBL   UCSC 
 
  136
 
  6
 
 12302
 
 tss
 
 0.012711
 
 0.9796467
 
 EFS (margin: RP11-124D2.3; IL25; CMTM5; EFS)
 
 11.4331031
 
 2.16e-03
 
 1.45e-02
  
 
  
 2
 
 202122309
 
 202122669
 
  PDF   STATS   ENSEMBL   UCSC 
 
  361
 
  6
 
 23817
 
 tss
 
 0.004098
 
 0.9039781
 
 CASP8 (margin: CASP8)
 
 11.4303743
 
 2.16e-03
 
 1.45e-02
  
 
  
 21
 
  42797847
 
  42798386
 
  PDF   STATS   ENSEMBL   UCSC 
 
  540
 
  6
 
 26430
 
 gene;island
 
 0.018467
 
 0.9735600
 
 MX1 (margin: MX1)
 
 11.2787617
 
 2.16e-03
 
 1.45e-02
  
 
  
 1
 
  89664260
 
  89664582
 
  PDF   STATS   ENSEMBL   UCSC 
 
  323
 
  6
 
 619
 
 gene
 
 0.018831
 
 0.9785379
 
 GBP4 (margin: GBP4)
 
 11.2573383
 
 2.16e-03
 
 1.45e-02
  
 
  
 7
 
  44152996
 
  44153500
 
  PDF   STATS   ENSEMBL   UCSC 
 
  505
 
  6
 
 36990
 
 gene;island
 
 0.978531
 
 0.0164788
 
 AEBP1; POLD2 (margin: AEBP1; MIR4649; POLD2; RNA5SP230)
 
 11.2434857
 
 2.16e-03
 
 1.45e-02
  
 
  
 6
 
  28367544
 
  28367898
 
  PDF   STATS   ENSEMBL   UCSC 
 
  355
 
  6
 
 34741
 
 tss
 
 0.013260
 
 0.9464397
 
 ZSCAN12 (margin: RNU2-45P; ZSCAN12)
 
 11.1121097
 
 2.16e-03
 
 1.45e-02
  
 
  
 19
 
  57702917
 
  57703364
 
  PDF   STATS   ENSEMBL   UCSC 
 
  448
 
  6
 
 20244
 
 gene
 
 0.007256
 
 0.9513954
 
 ZNF264 (margin: ZNF264)
 
 10.9859555
 
 2.16e-03
 
 1.45e-02
  
 
  
 1
 
  25256939
 
  25257191
 
  PDF   STATS   ENSEMBL   UCSC 
 
  253
 
  6
 
 330
 
 gene;tss;island
 
 0.030781
 
 0.9370643
 
 RUNX3 (margin: RUNX3)
 
 10.7447881
 
 2.16e-03
 
 1.45e-02
  
 
  
 2
 
 128158438
 
 128159058
 
  PDF   STATS   ENSEMBL   UCSC 
 
  621
 
  6
 
 24688
 
 island
 
 0.022968
 
 0.9439485
 
 AC068282.3 (margin: AC068282.3)
 
 10.7442647
 
 2.16e-03
 
 1.45e-02
  
 
  
 21
 
  34398085
 
  34398226
 
  PDF   STATS   ENSEMBL   UCSC 
 
  142
 
  6
 
 26516
 
 tss
 
 0.791043
 
 0.0052505
 
 OLIG2; AP000282.2 (margin: OLIG2; AP000282.2)
 
 10.6792855
 
 2.16e-03
 
 1.45e-02
  
 
  
 19
 
  50979561
 
  50979755
 
  PDF   STATS   ENSEMBL   UCSC 
 
  195
 
  6
 
 20177
 
 gene;island
 
 0.015864
 
 0.9650280
 
 EMC10; FAM71E1 (margin: MYBPC2; EMC10; FAM71E1; CTD-2545M3.2; CTD-2545M3.8)
 
 10.6546529
 
 2.16e-03
 
 1.45e-02
  
 
  
 7
 
  92237896
 
  92238364
 
  PDF   STATS   ENSEMBL   UCSC 
 
  469
 
  6
 
 37086
 
 gene
 
 0.975002
 
 0.0191165
 
 CDK6 (margin: CDK6)
 
 10.6250484
 
 2.16e-03
 
 1.45e-02
  
 
  
 19
 
  17414284
 
  17414399
 
  PDF   STATS   ENSEMBL   UCSC 
 
  116
 
  6
 
 20585
 
 tss
 
 0.012743
 
 0.9496120
 
 MRPL34; ABHD8 (margin: MRPL34; DDA1; ABHD8; CTD-2278I10.4)
 
 10.6057855
 
 2.16e-03
 
 1.45e-02
  
 
  
 19
 
  50832552
 
  50832861
 
  PDF   STATS   ENSEMBL   UCSC 
 
  310
 
  6
 
 22304
 
 island
 
 0.848330
 
 0.0114210
 
 NR1H2; KCNC3 (margin: NR1H2; KCNC3; NAPSB)
 
 10.5445466
 
 2.16e-03
 
 1.45e-02
  
 
  
 6
 
  26044204
 
  26044460
 
  PDF   STATS   ENSEMBL   UCSC 
 
  257
 
  6
 
 34683
 
 tss;island
 
 0.054543
 
 0.9856547
 
 U91328.2; HIST1H3C; HIST1H2BB (margin: U91328.2; HIST1H3C; HIST1H3B; HIST1H2AB; HIST1H2BB; HIST1H1C)
 
 10.4415439
 
 2.16e-03
 
 1.45e-02
  
 
  
 3
 
 197183271
 
 197183662
 
  PDF   STATS   ENSEMBL   UCSC 
 
  392
 
  6
 
 29817
 
 island
 
 0.025600
 
 0.9601856
 
 AC128709.2; AC128709.3; AC128709.4 (margin: AC128709.2; AC128709.3; AC128709.4)
 
 10.4251591
 
 2.16e-03
 
 1.45e-02
  
 
  
 19
 
  37407257
 
  37407486
 
  PDF   STATS   ENSEMBL   UCSC 
 
  230
 
  6
 
 20006
 
 gene
 
 0.909618
 
 0.0060311
 
 ZNF568; ZNF829 (margin: ZNF345; ZNF568; ZNF829)
 
 10.3867967
 
 2.16e-03
 
 1.45e-02
  
 
  
 3
 
  53032638
 
  53033167
 
  PDF   STATS   ENSEMBL   UCSC 
 
  530
 
  6
 
 27827
 
 gene;island
 
 0.029937
 
 0.9757820
 
 SFMBT1; RP11-894J14.5 (margin: SFMBT1; RP11-894J14.5)
 
 10.3284062
 
 2.16e-03
 
 1.45e-02
  
 
  
 6
 
  31025933
 
  31026287
 
  PDF   STATS   ENSEMBL   UCSC 
 
  355
 
  6
 
 33849
 
 gene
 
 0.001543
 
 0.1455433
 
 HCG22 (margin: HCG22)
 
 10.0920033
 
 2.16e-03
 
 1.45e-02
  
 
  
 20
 
  13976093
 
  13976143
 
  PDF   STATS   ENSEMBL   UCSC 
 
   51
 
  6
 
 25605
 
 tss
 
 0.876097
 
 0.0059560
 
 MACROD2; SEL1L2 (margin: MACROD2; RPS3P1; SEL1L2)
 
 10.0786322
 
 2.16e-03
 
 1.45e-02
  
 
  
 6
 
 132272342
 
 132272790
 
  PDF   STATS   ENSEMBL   UCSC 
 
  449
 
  6
 
 36481
 
 island
 
 0.564627
 
 0.0065621
 
 RP11-69I8.3; CTGF (margin: RP11-69I8.3; CTGF)
 
  9.9647102
 
 2.16e-03
 
 1.45e-02
  
 
  
 19
 
  52004992
 
  52005188
 
  PDF   STATS   ENSEMBL   UCSC 
 
  197
 
  6
 
 20996
 
 tss
 
 0.009491
 
 0.9109864
 
 SIGLEC12 (margin: CEACAM18; SIGLEC27P; SIGLEC12; SIGLEC26P)
 
  9.8896013
 
 2.16e-03
 
 1.45e-02
  
 
  
 10
 
 116164598
 
 116164955
 
  PDF   STATS   ENSEMBL   UCSC 
 
  358
 
  6
 
 5377
 
 tss;island
 
 0.034999
 
 0.9738634
 
 AFAP1L2 (margin: AFAP1L2)
 
  9.8635164
 
 2.16e-03
 
 1.45e-02
  
 
  
 1
 
  13910555
 
  13910796
 
  PDF   STATS   ENSEMBL   UCSC 
 
  242
 
  6
 
 235
 
 gene;tss;island
 
 0.049612
 
 0.9794611
 
 PDPN (margin: PDPN)
 
  9.8015297
 
 2.16e-03
 
 1.45e-02
  
 
  
 7
 
  38670804
 
  38671001
 
  PDF   STATS   ENSEMBL   UCSC 
 
  198
 
  6
 
 36972
 
 gene;island
 
 0.725317
 
 0.0078441
 
 AMPH (margin: AMPH)
 
  9.6041036
 
 2.16e-03
 
 1.45e-02
  
 
  
 2
 
 219858132
 
 219858271
 
  PDF   STATS   ENSEMBL   UCSC 
 
  140
 
  6
 
 23887
 
 tss
 
 0.047441
 
 0.9675989
 
 CRYBA2 (margin: AC097468.4; FEV; CRYBA2; MIR375; CCDC108)
 
  9.5451214
 
 2.16e-03
 
 1.45e-02
  
 
  
 2
 
 191045309
 
 191045668
 
  PDF   STATS   ENSEMBL   UCSC 
 
  360
 
  6
 
 23783
 
 tss
 
 0.087126
 
 0.9823493
 
 C2orf88 (margin: C2orf88; HIBCH)
 
  9.4527693
 
 2.16e-03
 
 1.45e-02
  
 
  
 22
 
  19710880
 
  19711051
 
  PDF   STATS   ENSEMBL   UCSC 
 
  172
 
  6
 
 26788
 
 gene;tss;island
 
 0.054825
 
 0.9844560
 
 SEPT5; GP1BB (margin: SEPT5; GP1BB)
 
  9.4200591
 
 2.16e-03
 
 1.45e-02
  
 
  
 20
 
  19915716
 
  19915874
 
  PDF   STATS   ENSEMBL   UCSC 
 
  159
 
  6
 
 25369
 
 gene
 
 0.058450
 
 0.9764447
 
 RIN2 (margin: RIN2)
 
  9.3459442
 
 2.16e-03
 
 1.45e-02
  
 
  
 3
 
  32442928
 
  32443496
 
  PDF   STATS   ENSEMBL   UCSC 
 
  569
 
  6
 
 27709
 
 gene;island
 
 0.128735
 
 0.9787877
 
 CMTM7 (margin: CMTM7)
 
  9.3369602
 
 2.16e-03
 
 1.45e-02
  
 
  
 12
 
 133022423
 
 133022955
 
  PDF   STATS   ENSEMBL   UCSC 
 
  533
 
  6
 
 11058
 
 island
 
 0.174085
 
 0.9622008
 
  (margin: RP11-503G7.1)
 
  9.3340928
 
 2.16e-03
 
 1.45e-02
  
 
  
 4
 
  91048416
 
  91048584
 
  PDF   STATS   ENSEMBL   UCSC 
 
  169
 
  6
 
 30498
 
 tss;island
 
 0.628345
 
 0.0058654
 
 CCSER1 (margin: CCSER1)
 
  9.2845555
 
 2.16e-03
 
 1.45e-02
  
 
  
 12
 
  56325715
 
  56325867
 
  PDF   STATS   ENSEMBL   UCSC 
 
  153
 
  6
 
 9169
 
 gene;island
 
 0.001860
 
 0.4586420
 
 DGKA; WIBG (margin: DGKA; WIBG)
 
  9.1769037
 
 2.16e-03
 
 1.45e-02
  
 
  
 19
 
  12267592
 
  12267796
 
  PDF   STATS   ENSEMBL   UCSC 
 
  205
 
  6
 
 20490
 
 tss
 
 0.018638
 
 0.9067951
 
 ZNF625-ZNF20; ZNF625 (margin: ZNF136; ZNF625-ZNF20; ZNF625)
 
  9.1102992
 
 2.16e-03
 
 1.45e-02
  
 
  
 5
 
   1882885
 
   1883011
 
  PDF   STATS   ENSEMBL   UCSC 
 
  127
 
  6
 
 31974
 
 tss
 
 0.726794
 
 0.0327835
 
 CTD-2194D22.3; IRX4 (margin: CTD-2194D22.3; CTD-2194D22.4; IRX4)
 
  9.0814996
 
 2.16e-03
 
 1.45e-02
  
 
  
 8
 
  58907018
 
  58907387
 
  PDF   STATS   ENSEMBL   UCSC 
 
  370
 
  6
 
 40321
 
 island
 
 0.843875
 
 0.0158443
 
 FAM110B (margin: RP11-1112C15.1; FAM110B)
 
  9.0539954
 
 2.16e-03
 
 1.45e-02
  
 
  
 1
 
  57110686
 
  57110878
 
  PDF   STATS   ENSEMBL   UCSC 
 
  193
 
  6
 
 1652
 
 tss;island
 
 0.684087
 
 0.0086618
 
 PRKAA2; PPAP2B (margin: PRKAA2; PPAP2B)
 
  9.0106775
 
 2.16e-03
 
 1.45e-02
  
 
  
 19
 
  16178091
 
  16178253
 
  PDF   STATS   ENSEMBL   UCSC 
 
  163
 
  6
 
 20566
 
 tss
 
 0.002484
 
 0.6072858
 
 CTD-2231E14.4; TPM4 (margin: CTD-2231E14.4; TPM4)
 
  9.0064436
 
 2.16e-03
 
 1.45e-02
  
 
  
 12
 
 106533667
 
 106533903
 
  PDF   STATS   ENSEMBL   UCSC 
 
  237
 
  6
 
 10747
 
 island
 
 0.054368
 
 0.9729925
 
 NUAK1 (margin: NUAK1)
 
  9.0042863
 
 2.16e-03
 
 1.45e-02
  
 
  
 5
 
 118691415
 
 118691794
 
  PDF   STATS   ENSEMBL   UCSC 
 
  380
 
  6
 
 31725
 
 gene;island
 
 0.003070
 
 0.3436751
 
 TNFAIP8 (margin: TNFAIP8)
 
  8.9874967
 
 2.16e-03
 
 1.45e-02
  
 
  
 10
 
  30723892
 
  30724374
 
  PDF   STATS   ENSEMBL   UCSC 
 
  483
 
  6
 
 4550
 
 gene;island
 
 0.008516
 
 0.8208121
 
 MAP3K8 (margin: MAP3K8)
 
  8.9348439
 
 2.16e-03
 
 1.45e-02
  
 
  
 14
 
  24457893
 
  24458014
 
  PDF   STATS   ENSEMBL   UCSC 
 
  122
 
  6
 
 12308
 
 tss
 
 0.013160
 
 0.7372064
 
 DHRS4L2; DHRS4-AS1 (margin: DHRS4L2; DHRS4-AS1)
 
  8.9155687
 
 2.16e-03
 
 1.45e-02
  
 
  
 5
 
  89854293
 
  89854539
 
  PDF   STATS   ENSEMBL   UCSC 
 
  247
 
  6
 
 32174
 
 tss
 
 0.635778
 
 0.0132259
 
 GPR98 (margin: GPR98)
 
  8.8532947
 
 2.16e-03
 
 1.45e-02
  
 
  
 3
 
  48700375
 
  48700498
 
  PDF   STATS   ENSEMBL   UCSC 
 
  124
 
  6
 
 28305
 
 tss
 
 0.422085
 
 0.0030524
 
 RP11-148G20.1; CELSR3; NCKIPSD (margin: RP11-148G20.1; RP11-572O6.1; CELSR3; NCKIPSD)
 
  8.8082270
 
 2.16e-03
 
 1.45e-02
  
 
  
 1
 
  42384310
 
  42384647
 
  PDF   STATS   ENSEMBL   UCSC 
 
  338
 
  6
 
 3142
 
 island
 
 0.604034
 
 0.0168155
 
 HIVEP3 (margin: HIVEP3)
 
  8.7883408
 
 2.16e-03
 
 1.45e-02
  
 
  
 1
 
 153508511
 
 153508713
 
  PDF   STATS   ENSEMBL   UCSC 
 
  203
 
  6
 
 760
 
 gene
 
 0.003945
 
 0.6063518
 
 BX470102.3; S100A6; S100A5 (margin: BX470102.3; S100A6; S100A5; S100A4; S100A3)
 
  8.7542668
 
 2.16e-03
 
 1.45e-02
  
 
  
 5
 
 140105626
 
 140105729
 
  PDF   STATS   ENSEMBL   UCSC 
 
  104
 
  6
 
 32351
 
 tss
 
 0.001389
 
 0.3778140
 
 VTRNA1-3 (margin: VTRNA1-2; VTRNA1-3; RP11-515C16.7)
 
  8.7344887
 
 2.16e-03
 
 1.45e-02
  
 
  
 16
 
   3096477
 
   3096711
 
  PDF   STATS   ENSEMBL   UCSC 
 
  235
 
  6
 
 15635
 
 island
 
 0.057479
 
 0.9310677
 
 MMP25 (margin: RP11-473M20.5; MMP25; CCDC64B; RP11-473M20.7)
 
  8.6939988
 
 2.16e-03
 
 1.45e-02
  
 
  
 17
 
  78161467
 
  78161706
 
  PDF   STATS   ENSEMBL   UCSC 
 
  240
 
  6
 
 16859
 
 gene;island
 
 0.014000
 
 0.8784141
 
 CARD14 (margin: CARD14)
 
  8.6850681
 
 2.16e-03
 
 1.45e-02
  
 
  
 19
 
   6740812
 
   6741181
 
  PDF   STATS   ENSEMBL   UCSC 
 
  370
 
  6
 
 19771
 
 gene;island
 
 0.191525
 
 0.9689831
 
 TRIP10 (margin: TRIP10; C3; GPR108; CTD-3128G10.6; SH2D3A)
 
  8.6641342
 
 2.16e-03
 
 1.45e-02
  
 
  
 20
 
  44540777
 
  44541153
 
  PDF   STATS   ENSEMBL   UCSC 
 
  377
 
  6
 
 26209
 
 island
 
 0.202694
 
 0.9888702
 
 PLTP (margin: PLTP)
 
  8.6498974
 
 2.16e-03
 
 1.45e-02
  
 
  
 19
 
  38794554
 
  38794845
 
  PDF   STATS   ENSEMBL   UCSC 
 
  292
 
  6
 
 20014
 
 gene
 
 0.003595
 
 0.5431034
 
 CTB-102L5.4; C19orf33; YIF1B (margin: SPINT2; CTB-102L5.4; Y_RNA; C19orf33; YIF1B)
 
  8.6029002
 
 2.16e-03
 
 1.45e-02
  
 
  
 8
 
  49647579
 
  49647833
 
  PDF   STATS   ENSEMBL   UCSC 
 
  255
 
  6
 
 39301
 
 gene
 
 0.079211
 
 0.9791698
 
 EFCAB1 (margin: EFCAB1)
 
  8.6005012
 
 2.16e-03
 
 1.45e-02
  
 
  
 10
 
    729479
 
    729956
 
  PDF   STATS   ENSEMBL   UCSC 
 
  478
 
  6
 
 4451
 
 gene
 
 0.052854
 
 0.9782908
 
 DIP2C (margin: DIP2C)
 
  8.5988036
 
 2.16e-03
 
 1.45e-02
  
 
  
 14
 
 100626084
 
 100626205
 
  PDF   STATS   ENSEMBL   UCSC 
 
  122
 
  6
 
 12602
 
 tss;island
 
 0.827248
 
 0.0153922
 
 DEGS2 (margin: DEGS2)
 
  8.5970206
 
 2.16e-03
 
 1.45e-02
  
 
  
 19
 
  38085706
 
  38085810
 
  PDF   STATS   ENSEMBL   UCSC 
 
  105
 
  6
 
 20008
 
 gene;tss;island
 
 0.401397
 
 0.0015035
 
 ZNF540; ZNF571 (margin: ZNF571-AS1; ZNF540; ZNF571)
 
  8.5681167
 
 2.16e-03
 
 1.45e-02
  
 
  
 4
 
  47839876
 
  47840162
 
  PDF   STATS   ENSEMBL   UCSC 
 
  287
 
  6
 
 31025
 
 island
 
 0.151099
 
 0.9731888
 
 CORIN; RP11-121C2.2 (margin: CORIN; RP11-121C2.2; NFXL1)
 
  8.5220719
 
 2.16e-03
 
 1.45e-02
  
 
  
 5
 
  42949542
 
  42950152
 
  PDF   STATS   ENSEMBL   UCSC 
 
  611
 
  6
 
 32761
 
 island
 
 0.216285
 
 0.9858768
 
  (margin: )
 
  8.5147621
 
 2.16e-03
 
 1.45e-02
  
 
  
 10
 
 128994432
 
 128994702
 
  PDF   STATS   ENSEMBL   UCSC 
 
  271
 
  6
 
 5434
 
 tss
 
 0.739762
 
 0.0072433
 
 DOCK1; FAM196A (margin: DOCK1; FAM196A)
 
  8.4313480
 
 2.16e-03
 
 1.45e-02
  
 
  
 19
 
  48896922
 
  48897279
 
  PDF   STATS   ENSEMBL   UCSC 
 
  358
 
  6
 
 20914
 
 tss;island
 
 0.834172
 
 0.0085994
 
 GRIN2D (margin: GRIN2D; KDELR1)
 
  8.4305242
 
 2.16e-03
 
 1.45e-02
  
 
  
 12
 
  81471867
 
  81472177
 
  PDF   STATS   ENSEMBL   UCSC 
 
  311
 
  6
 
 9228
 
 gene
 
 0.676889
 
 0.0072133
 
 ACSS3 (margin: ACSS3)
 
  8.4189073
 
 2.16e-03
 
 1.45e-02
  
 
  
 8
 
  23021325
 
  23021690
 
  PDF   STATS   ENSEMBL   UCSC 
 
  366
 
  6
 
 40146
 
 island
 
 0.136673
 
 0.9760923
 
 TNFRSF10D (margin: TNFRSF10D)
 
  8.2730741
 
 2.16e-03
 
 1.45e-02
  
 
  
 6
 
   5996893
 
   5997375
 
  PDF   STATS   ENSEMBL   UCSC 
 
  483
 
  6
 
 35404
 
 island
 
 0.023421
 
 0.8363634
 
 NRN1 (margin: NRN1)
 
  8.2628032
 
 2.16e-03
 
 1.45e-02
  
 
  
 11
 
   2170831
 
   2171184
 
  PDF   STATS   ENSEMBL   UCSC 
 
  354
 
  6
 
 6449
 
 gene
 
 0.037514
 
 0.7493195
 
 IGF2-AS; IGF2; INS-IGF2 (margin: IGF2-AS; IGF2; INS-IGF2; INS)
 
  8.2409050
 
 2.16e-03
 
 1.45e-02
  
 
  
 16
 
  88943191
 
  88943641
 
  PDF   STATS   ENSEMBL   UCSC 
 
  451
 
  6
 
 14920
 
 gene;island
 
 0.178678
 
 0.9720561
 
 CBFA2T3 (margin: RP11-830F9.5; PABPN1L; CBFA2T3)
 
  8.1980218
 
 2.16e-03
 
 1.45e-02
  
 
  
 12
 
  15374457
 
  15374609
 
  PDF   STATS   ENSEMBL   UCSC 
 
  153
 
  6
 
 9590
 
 tss
 
 0.687846
 
 0.0120080
 
 RERG (margin: RERG)
 
  8.1652076
 
 2.16e-03
 
 1.45e-02
  
 
  
 6
 
  28411279
 
  28411423
 
  PDF   STATS   ENSEMBL   UCSC 
 
  145
 
  6
 
 34742
 
 tss
 
 0.100601
 
 0.9449737
 
 ZSCAN23 (margin: COX11P1; ZSCAN23)
 
  8.1346679
 
 2.16e-03
 
 1.45e-02
  
 
  
 14
 
  91580380
 
  91580613
 
  PDF   STATS   ENSEMBL   UCSC 
 
  234
 
  6
 
 12176
 
 gene
 
 0.033793
 
 0.8922496
 
 C14orf159 (margin: C14orf159)
 
  8.1196339
 
 2.16e-03
 
 1.45e-02
  
 
  
 16
 
    604802
 
    605347
 
  PDF   STATS   ENSEMBL   UCSC 
 
  546
 
  6
 
 15462
 
 island
 
 0.127057
 
 0.9723118
 
 CAPN15; LA16c-366D1.3 (margin: CAPN15; MIR3176; C16orf11; PIGQ; NHLRC4; LA16c-366D1.3)
 
  8.0476031
 
 2.16e-03
 
 1.45e-02
  
 
  
 11
 
 101000436
 
 101000717
 
  PDF   STATS   ENSEMBL   UCSC 
 
  282
 
  6
 
 8755
 
 island
 
 0.312186
 
 0.0131263
 
 PGR (margin: PGR)
 
  8.0348842
 
 2.16e-03
 
 1.45e-02
  
 
  
 5
 
 140220686
 
 140221106
 
  PDF   STATS   ENSEMBL   UCSC 
 
  421
 
  6
 
 31799
 
 gene;island
 
 0.841240
 
 0.0186792
 
 PCDHA1; PCDHA2; PCDHA3; PCDHA4; PCDHA5; PCDHA6; PCDHA7; PCDHA8 (margin: PCDHA1; PCDHA2; PCDHA3; PCDHA4; PCDHA5; PCDHA6; PCDHA7; PCDHA8; PCDHA9)
 
  8.0235278
 
 2.16e-03
 
 1.45e-02
  
 
  
 16
 
  67918484
 
  67918965
 
  PDF   STATS   ENSEMBL   UCSC 
 
  482
 
  8
 
 16033
 
 island
 
 0.902547
 
 0.0310864
 
 EDC4; CTC-479C5.10; NRN1L (margin: EDC4; AC040162.1; CTC-479C5.10; NRN1L; PSKH1)
 
  8.0154458
 
 1.86e-03
 
 1.45e-02
  
 
  
 8
 
 110703888
 
 110704289
 
  PDF   STATS   ENSEMBL   UCSC 
 
  402
 
  6
 
 40541
 
 island
 
 0.681649
 
 0.0084202
 
 SYBU (margin: SYBU)
 
  7.9929109
 
 2.16e-03
 
 1.45e-02
  
 
  
 1
 
  35220224
 
  35220710
 
  PDF   STATS   ENSEMBL   UCSC 
 
  487
 
  6
 
 1478
 
 tss
 
 0.080881
 
 0.9614388
 
 GJB5; SMIM12 (margin: GJB5; GJB4; SMIM12; RP1-34M23.5)
 
  7.9783803
 
 2.16e-03
 
 1.45e-02
  
 
  
 2
 
 227700458
 
 227700568
 
  PDF   STATS   ENSEMBL   UCSC 
 
  111
 
  6
 
 23928
 
 tss;island
 
 0.731950
 
 0.0219031
 
 RHBDD1 (margin: RHBDD1)
 
  7.9574454
 
 2.16e-03
 
 1.45e-02
  
 
  
 11
 
   1471410
 
   1471699
 
  PDF   STATS   ENSEMBL   UCSC 
 
  290
 
  6
 
 6420
 
 gene;island
 
 0.145169
 
 0.9685869
 
 BRSK2 (margin: BRSK2)
 
  7.9396529
 
 2.16e-03
 
 1.45e-02
  
 
  
 13
 
  99135543
 
  99135804
 
  PDF   STATS   ENSEMBL   UCSC 
 
  262
 
  6
 
 11201
 
 gene
 
 0.116801
 
 0.9320179
 
 STK24 (margin: STK24)
 
  7.8764828
 
 2.16e-03
 
 1.45e-02
  
 
  
 1
 
 179560700
 
 179561111
 
  PDF   STATS   ENSEMBL   UCSC 
 
  412
 
  6
 
 4000
 
 island
 
 0.631001
 
 0.0096398
 
 RP11-545A16.4; TDRD5 (margin: RP11-545A16.3; RP11-545A16.4; TDRD5)
 
  7.8741291
 
 2.16e-03
 
 1.45e-02
  
 
  
 13
 
  78272372
 
  78272443
 
  PDF   STATS   ENSEMBL   UCSC 
 
   72
 
  6
 
 11486
 
 tss
 
 0.656605
 
 0.0077207
 
 SLAIN1; MIR3665 (margin: SLAIN1; MIR3665)
 
  7.8376445
 
 2.16e-03
 
 1.45e-02
  
 
  
 11
 
 120008025
 
 120008607
 
  PDF   STATS   ENSEMBL   UCSC 
 
  583
 
  6
 
 6943
 
 gene;island
 
 0.062986
 
 0.9285541
 
 TRIM29 (margin: TRIM29)
 
  7.8302893
 
 2.16e-03
 
 1.45e-02
  
 
  
 10
 
  77163316
 
  77163401
 
  PDF   STATS   ENSEMBL   UCSC 
 
   86
 
  6
 
 4643
 
 gene;tss;island
 
 0.009462
 
 0.5683886
 
 ZNF503-AS2; ZNF503 (margin: ZNF503-AS2; RP11-399K21.11; ZNF503)
 
  7.8177442
 
 2.16e-03
 
 1.45e-02
  
 
  
 17
 
  14204310
 
  14204593
 
  PDF   STATS   ENSEMBL   UCSC 
 
  284
 
  6
 
 18034
 
 island
 
 0.510526
 
 0.0050110
 
 HS3ST3B1 (margin: HS3ST3B1; RP11-214O1.2; RP11-214O1.1)
 
  7.7484229
 
 2.16e-03
 
 1.45e-02
  
 
  
 4
 
   8582101
 
   8582287
 
  PDF   STATS   ENSEMBL   UCSC 
 
  187
 
  6
 
 30298
 
 tss
 
 0.652664
 
 0.0118088
 
 GPR78 (margin: GPR78)
 
  7.7279544
 
 2.16e-03
 
 1.45e-02
  
 
  
 19
 
  18118666
 
  18119069
 
  PDF   STATS   ENSEMBL   UCSC 
 
  404
 
  6
 
 19926
 
 gene;island
 
 0.171320
 
 0.9799799
 
 ARRDC2 (margin: CTB-52I2.8; ARRDC2; CTB-52I2.5)
 
  7.7251967
 
 2.16e-03
 
 1.45e-02
  
 
  
 12
 
 108733253
 
 108733370
 
  PDF   STATS   ENSEMBL   UCSC 
 
  118
 
  6
 
 9980
 
 tss
 
 0.077269
 
 0.9258097
 
 CMKLR1 (margin: CMKLR1)
 
  7.7147375
 
 2.16e-03
 
 1.45e-02
  
 
  
 7
 
  75932840
 
  75932977
 
  PDF   STATS   ENSEMBL   UCSC 
 
  138
 
  6
 
 37053
 
 gene;island
 
 0.397926
 
 0.0038307
 
 HSPB1 (margin: HSPB1)
 
  7.6432360
 
 2.16e-03
 
 1.45e-02
  
 
  
 7
 
  87563462
 
  87563649
 
  PDF   STATS   ENSEMBL   UCSC 
 
  188
 
  6
 
 37689
 
 tss
 
 0.490881
 
 0.0053348
 
 ADAM22 (margin: ADAM22)
 
  7.6377574
 
 2.16e-03
 
 1.45e-02
  
 
  
 20
 
   1206682
 
   1206758
 
  PDF   STATS   ENSEMBL   UCSC 
 
   77
 
  6
 
 25535
 
 tss
 
 0.163230
 
 0.0037844
 
 RAD21L1 (margin: RAD21L1)
 
  7.6231171
 
 2.16e-03
 
 1.45e-02
  
 
  
 14
 
  23305548
 
  23305957
 
  PDF   STATS   ENSEMBL   UCSC 
 
  410
 
  6
 
 12703
 
 island
 
 0.041398
 
 0.8902814
 
 MRPL52; MMP14 (margin: MRPL52; MMP14; SLC7A7)
 
  7.6190887
 
 2.16e-03
 
 1.45e-02
  
 
  
 15
 
  93199018
 
  93199180
 
  PDF   STATS   ENSEMBL   UCSC 
 
  163
 
  6
 
 14473
 
 island
 
 0.439713
 
 0.0084372
 
 FAM174B (margin: FAM174B)
 
  7.5413871
 
 2.16e-03
 
 1.45e-02
  
 
  
 6
 
  26189333
 
  26189597
 
  PDF   STATS   ENSEMBL   UCSC 
 
  265
 
  6
 
 34692
 
 tss
 
 0.002518
 
 0.2593597
 
 HIST1H4D (margin: HIST1H2BE; HIST1H1PS1; HIST1H2BF; HIST1H4D; HIST1H3D; HIST1H2AD)
 
  7.5379996
 
 2.16e-03
 
 1.45e-02
  
 
  
 18
 
  20714217
 
  20714496
 
  PDF   STATS   ENSEMBL   UCSC 
 
  280
 
  6
 
 19203
 
 tss;island
 
 0.494748
 
 0.0055299
 
 CABLES1; AC105247.1 (margin: CABLES1; AC105247.1)
 
  7.5354126
 
 2.16e-03
 
 1.45e-02
  
 
  
 11
 
 119020099
 
 119020168
 
  PDF   STATS   ENSEMBL   UCSC 
 
   70
 
  6
 
 6937
 
 gene;tss;island
 
 0.245700
 
 0.0050855
 
 ABCG4 (margin: ABCG4; AP002956.1)
 
  7.5335157
 
 2.16e-03
 
 1.45e-02
  
 
  
 5
 
  67583972
 
  67584222
 
  PDF   STATS   ENSEMBL   UCSC 
 
  251
 
  6
 
 32103
 
 tss
 
 0.637094
 
 0.0098041
 
 PIK3R1 (margin: PIK3R1)
 
  7.5051623
 
 2.16e-03
 
 1.45e-02
  
 
  
 7
 
 122526824
 
 122526940
 
  PDF   STATS   ENSEMBL   UCSC 
 
  117
 
  6
 
 37867
 
 tss
 
 0.479181
 
 0.0074916
 
 CADPS2 (margin: CADPS2)
 
  7.5049131
 
 2.16e-03
 
 1.45e-02
  
 
  
 18
 
  70211774
 
  70211974
 
  PDF   STATS   ENSEMBL   UCSC 
 
  201
 
  6
 
 19318
 
 tss;island
 
 0.667853
 
 0.0143937
 
 CBLN2 (margin: CBLN2)
 
  7.4988983
 
 2.16e-03
 
 1.45e-02
  
 
  
 12
 
  98850691
 
  98851021
 
  PDF   STATS   ENSEMBL   UCSC 
 
  331
 
  6
 
 10703
 
 island
 
 0.260487
 
 0.9769877
 
 SLC9A7P1 (margin: SLC9A7P1)
 
  7.4551427
 
 2.16e-03
 
 1.45e-02
  
 
  
 11
 
 121970496
 
 121970768
 
  PDF   STATS   ENSEMBL   UCSC 
 
  273
 
  6
 
 6946
 
 gene
 
 0.971004
 
 0.1794332
 
 RP11-166D19.1; MIR125B1 (margin: RP11-166D19.1; MIR125B1)
 
  7.4345054
 
 2.16e-03
 
 1.45e-02
  
 
  
 7
 
   8473234
 
   8473578
 
  PDF   STATS   ENSEMBL   UCSC 
 
  345
 
  6
 
 37439
 
 tss;island
 
 0.778704
 
 0.0254412
 
 NXPH1 (margin: NXPH1)
 
  7.4068730
 
 2.16e-03
 
 1.45e-02
  
 
  
 15
 
  78423938
 
  78424152
 
  PDF   STATS   ENSEMBL   UCSC 
 
  215
 
  6
 
 13829
 
 tss
 
 0.522790
 
 0.0165245
 
 IDH3A; CIB2 (margin: IDH3A; RP11-285A1.1; CIB2)
 
  7.3905405
 
 2.16e-03
 
 1.45e-02
  
 
  
 4
 
  55991782
 
  55991943
 
  PDF   STATS   ENSEMBL   UCSC 
 
  162
 
  6
 
 30402
 
 tss
 
 0.643531
 
 0.0244791
 
 KDR (margin: KDR)
 
  7.3873816
 
 2.16e-03
 
 1.45e-02
  
 
  
 12
 
 100750473
 
 100750811
 
  PDF   STATS   ENSEMBL   UCSC 
 
  339
 
  6
 
 9941
 
 tss
 
 0.114190
 
 0.9331340
 
 SLC17A8 (margin: SLC17A8)
 
  7.3602428
 
 2.16e-03
 
 1.45e-02
  
 
  
 5
 
 134824531
 
 134824947
 
  PDF   STATS   ENSEMBL   UCSC 
 
  417
 
  6
 
 33136
 
 island
 
 0.516441
 
 0.0150870
 
  (margin: )
 
  7.3288954
 
 2.16e-03
 
 1.45e-02
  
 
  
 4
 
  94749865
 
  94750061
 
  PDF   STATS   ENSEMBL   UCSC 
 
  197
 
  6
 
 30501
 
 tss
 
 0.560383
 
 0.0082569
 
 ATOH1 (margin: RNA5SP164; ATOH1)
 
  7.3061981
 
 2.16e-03
 
 1.45e-02
  
 
  
 11
 
  92931250
 
  92931416
 
  PDF   STATS   ENSEMBL   UCSC 
 
  167
 
  6
 
 7618
 
 tss;island
 
 0.395396
 
 0.0062886
 
 SLC36A4 (margin: SLC36A4)
 
  7.3058145
 
 2.16e-03
 
 1.45e-02
  
 
  
 10
 
  94833520
 
  94833632
 
  PDF   STATS   ENSEMBL   UCSC 
 
  113
 
  6
 
 5235
 
 tss
 
 0.467561
 
 0.0069444
 
 CYP26A1 (margin: CYP26C1; CYP26A1)
 
  7.3050710
 
 2.16e-03
 
 1.45e-02
  
 
  
 6
 
  29429909
 
  29430506
 
  PDF   STATS   ENSEMBL   UCSC 
 
  598
 
  6
 
 33658
 
 gene
 
 0.003507
 
 0.4708638
 
 OR2H1; UBDP1 (margin: OR2H1; OR11A1; UBDP1)
 
  7.2720927
 
 2.16e-03
 
 1.45e-02
  
 
  
 11
 
 133938676
 
 133939064
 
  PDF   STATS   ENSEMBL   UCSC 
 
  389
 
  6
 
 8974
 
 island
 
 0.218973
 
 0.9796876
 
 JAM3 (margin: JAM3)
 
  7.2651772
 
 2.16e-03
 
 1.45e-02
  
 
  
 11
 
   8832817
 
   8833095
 
  PDF   STATS   ENSEMBL   UCSC 
 
  279
 
  6
 
 6500
 
 gene;tss
 
 0.111682
 
 0.9467558
 
 RP11-318C2.1; ST5 (margin: RP11-318C2.1; ST5)
 
  7.2435961
 
 2.16e-03
 
 1.45e-02
  
 
  
 12
 
  54366760
 
  54367008
 
  PDF   STATS   ENSEMBL   UCSC 
 
  249
 
  6
 
 10484
 
 island
 
 0.004694
 
 0.3876561
 
 HOXC11; HOTAIR (margin: HOXC11; HOXC10; HOTAIR; HOXC-AS3)
 
  7.1593076
 
 2.16e-03
 
 1.45e-02
  
 
  
 7
 
  50633725
 
  50634252
 
  PDF   STATS   ENSEMBL   UCSC 
 
  528
 
  6
 
 37595
 
 tss
 
 0.050865
 
 0.8404515
 
 DDC (margin: DDC)
 
  7.1483892
 
 2.16e-03
 
 1.45e-02
  
 
  
 8
 
  63998640
 
  63998778
 
  PDF   STATS   ENSEMBL   UCSC 
 
  139
 
  6
 
 39731
 
 tss
 
 0.526332
 
 0.0097061
 
 TTPA (margin: TTPA)
 
  7.1122091
 
 2.16e-03
 
 1.45e-02
  
 
  
 10
 
  97666894
 
  97667244
 
  PDF   STATS   ENSEMBL   UCSC 
 
  351
 
  6
 
 5250
 
 tss;island
 
 0.474981
 
 0.0063948
 
 RP11-248J23.7; C10orf131; ENTPD1-AS1 (margin: RP11-248J23.7; C10orf131; ENTPD1-AS1)
 
  7.1030486
 
 2.16e-03
 
 1.45e-02
  
 
  
 19
 
  37288291
 
  37288793
 
  PDF   STATS   ENSEMBL   UCSC 
 
  503
 
  6
 
 21946
 
 island
 
 0.673186
 
 0.0182970
 
 CTD-2162K18.5 (margin: CTD-2162K18.5)
 
  7.0410280
 
 2.16e-03
 
 1.45e-02
  
 
  
 4
 
   6202379
 
   6202609
 
  PDF   STATS   ENSEMBL   UCSC 
 
  231
 
  6
 
 30278
 
 tss;island
 
 0.623207
 
 0.0137139
 
 RP11-586D19.1; RP11-586D19.2; JAKMIP1 (margin: RP11-586D19.1; RP11-586D19.2; JAKMIP1)
 
  7.0386240
 
 2.16e-03
 
 1.45e-02
  
 
  
 5
 
 156886816
 
 156887005
 
  PDF   STATS   ENSEMBL   UCSC 
 
  190
 
  6
 
 32456
 
 tss
 
 0.163736
 
 0.9514387
 
 NIPAL4; CTB-109A12.1; ADAM19 (margin: NIPAL4; CTB-109A12.1; ADAM19)
 
  7.0373457
 
 2.16e-03
 
 1.45e-02
  
 
  
 19
 
  50004152
 
  50004625
 
  PDF   STATS   ENSEMBL   UCSC 
 
  474
 
  6
 
 20952
 
 tss
 
 0.281142
 
 0.9360570
 
 RPS11; hsa-mir-150; MIR150; CTD-3148I10.13 (margin: RPL13A; SNORD32A; SNORD33; SNORD34; SNORD35A; RPS11; SNORD35B; hsa-mir-150; FCGRT; MIR150; CTD-3148I10.13)
 
  7.0334316
 
 2.16e-03
 
 1.45e-02
  
 
  
 17
 
   8869099
 
   8869285
 
  PDF   STATS   ENSEMBL   UCSC 
 
  187
 
  6
 
 17124
 
 tss;island
 
 0.777673
 
 0.0231897
 
 CTB-41I6.1; CTB-41I6.2; PIK3R5 (margin: CTB-41I6.1; CTB-41I6.2; PIK3R5)
 
  6.9980445
 
 2.16e-03
 
 1.45e-02
  
 
  
 17
 
  38599271
 
  38599763
 
  PDF   STATS   ENSEMBL   UCSC 
 
  493
 
  6
 
 18306
 
 island
 
 0.827302
 
 0.0228525
 
 IGFBP4 (margin: IGFBP4; RP11-58O9.1)
 
  6.9936926
 
 2.16e-03
 
 1.45e-02
  
 
  
 3
 
 186649165
 
 186649855
 
  PDF   STATS   ENSEMBL   UCSC 
 
  691
 
  6
 
 28076
 
 gene;island
 
 0.637317
 
 0.0141809
 
 ST6GAL1; AC007690.1 (margin: ST6GAL1; AC007690.1; RP11-42D20.1)
 
  6.9823239
 
 2.16e-03
 
 1.45e-02
  
 
  
 5
 
 154091976
 
 154092426
 
  PDF   STATS   ENSEMBL   UCSC 
 
  451
 
  6
 
 32450
 
 tss
 
 0.630488
 
 0.0125109
 
 LARP1 (margin: LARP1; RN7SL439P; Metazoa_SRP)
 
  6.9737118
 
 2.16e-03
 
 1.45e-02
  
 
  
 5
 
 150399781
 
 150400672
 
  PDF   STATS   ENSEMBL   UCSC 
 
  892
 
  9
 
 33315
 
 island
 
 0.956282
 
 0.1068098
 
 GPX3 (margin: GPX3; TNIP1)
 
  6.9219707
 
 1.85e-03
 
 1.45e-02
  
 
  
 11
 
 128761146
 
 128761211
 
  PDF   STATS   ENSEMBL   UCSC 
 
   66
 
  6
 
 7807
 
 tss;island
 
 0.023015
 
 0.6408832
 
 KCNJ5 (margin: KCNJ5; RP11-740D6.3; C11orf45)
 
  6.8218122
 
 2.16e-03
 
 1.45e-02
  
 
  
 6
 
  41620951
 
  41621322
 
  PDF   STATS   ENSEMBL   UCSC 
 
  372
 
  6
 
 34331
 
 gene;island
 
 0.862050
 
 0.0476304
 
 MDFI (margin: MDFI)
 
  6.7660160
 
 2.16e-03
 
 1.45e-02
  
 
  
 12
 
   5541220
 
   5541403
 
  PDF   STATS   ENSEMBL   UCSC 
 
  184
 
  6
 
 10182
 
 island
 
 0.077432
 
 0.8709769
 
 NTF3 (margin: NTF3)
 
  6.7648892
 
 2.16e-03
 
 1.45e-02
  
 
  
 4
 
 186435443
 
 186435667
 
  PDF   STATS   ENSEMBL   UCSC 
 
  225
 
  6
 
 30210
 
 gene
 
 0.976491
 
 0.2844882
 
 PDLIM3 (margin: PDLIM3)
 
  6.7492592
 
 2.16e-03
 
 1.45e-02
  
 
  
 14
 
  75535757
 
  75536217
 
  PDF   STATS   ENSEMBL   UCSC 
 
  461
 
  6
 
 12515
 
 tss
 
 0.010809
 
 0.5589423
 
 ZC2HC1C; ACYP1 (margin: ZC2HC1C; ACYP1)
 
  6.7383027
 
 2.16e-03
 
 1.45e-02
  
 
  
 15
 
  91497863
 
  91498080
 
  PDF   STATS   ENSEMBL   UCSC 
 
  218
 
  6
 
 13908
 
 tss
 
 0.783728
 
 0.0370683
 
 UNC45A; RCCD1; AC068831.6 (margin: UNC45A; RCCD1; PRC1-AS1; AC068831.6; PRC1)
 
  6.7233587
 
 2.16e-03
 
 1.45e-02
  
 
  
 13
 
 113759771
 
 113760286
 
  PDF   STATS   ENSEMBL   UCSC 
 
  516
 
  6
 
 11956
 
 island
 
 0.559401
 
 0.0194431
 
 F7 (margin: MCF2L; F7; AL137002.1)
 
  6.6822449
 
 2.16e-03
 
 1.45e-02
  
 
  
 1
 
 156675388
 
 156675562
 
  PDF   STATS   ENSEMBL   UCSC 
 
  175
 
  6
 
 2020
 
 tss
 
 0.632415
 
 0.0219552
 
 CRABP2 (margin: CRABP2; RP11-66D17.5)
 
  6.6491008
 
 2.16e-03
 
 1.45e-02
  
 
  
 19
 
  37406932
 
  37407152
 
  PDF   STATS   ENSEMBL   UCSC 
 
  221
 
  6
 
 20005
 
 gene
 
 0.482800
 
 0.0083643
 
 ZNF568; ZNF829 (margin: ZNF345; ZNF568; ZNF829)
 
  6.6412834
 
 2.16e-03
 
 1.45e-02
  
 
  
 15
 
  63674108
 
  63674276
 
  PDF   STATS   ENSEMBL   UCSC 
 
  169
 
  6
 
 13722
 
 tss;island
 
 0.488175
 
 0.0196455
 
 CA12 (margin: RP11-321G12.1; CA12)
 
  6.6350141
 
 2.16e-03
 
 1.45e-02
  
 
  
 14
 
  36003074
 
  36003211
 
  PDF   STATS   ENSEMBL   UCSC 
 
  138
 
  6
 
 12354
 
 tss
 
 0.523529
 
 0.0138044
 
 INSM2 (margin: INSM2; RALGAPA1)
 
  6.6286022
 
 2.16e-03
 
 1.45e-02
  
 
  
 11
 
   6440279
 
   6440482
 
  PDF   STATS   ENSEMBL   UCSC 
 
  204
 
  6
 
 6490
 
 gene;island
 
 0.581947
 
 0.0170675
 
 APBB1 (margin: APBB1; HPX)
 
  6.5910893
 
 2.16e-03
 
 1.45e-02
  
 
  
 7
 
   3341524
 
   3342048
 
  PDF   STATS   ENSEMBL   UCSC 
 
  525
 
  6
 
 36823
 
 gene;island
 
 0.547954
 
 0.0345279
 
 SDK1; AC073316.1 (margin: SDK1; AC073316.1)
 
  6.5899559
 
 2.16e-03
 
 1.45e-02
  
 
  
 6
 
 146136083
 
 146136749
 
  PDF   STATS   ENSEMBL   UCSC 
 
  667
 
 10
 
 35273
 
 tss;island
 
 0.011085
 
 0.5034272
 
 RP11-545I5.3; FBXO30 (margin: RP11-545I5.3; FBXO30)
 
  6.5875054
 
 2.09e-03
 
 1.45e-02
  
 
  
 17
 
  75471193
 
  75471491
 
  PDF   STATS   ENSEMBL   UCSC 
 
  299
 
  6
 
 16811
 
 gene
 
 0.347778
 
 0.9798225
 
 SEPT9 (margin: SEPT9; RP11-75C10.7; RP11-75C10.9)
 
  6.5709414
 
 2.16e-03
 
 1.45e-02
  
 
  
 1
 
 203598573
 
 203599089
 
  PDF   STATS   ENSEMBL   UCSC 
 
  517
 
  6
 
 953
 
 gene;island
 
 0.262317
 
 0.9815181
 
 ATP2B4 (margin: ATP2B4)
 
  6.5402924
 
 2.16e-03
 
 1.45e-02
  
 
  
 20
 
   9819697
 
   9819820
 
  PDF   STATS   ENSEMBL   UCSC 
 
  124
 
  6
 
 25593
 
 tss;island
 
 0.309722
 
 0.0063629
 
 PAK7 (margin: PAK7)
 
  6.5265253
 
 2.16e-03
 
 1.45e-02
  
 
  
 1
 
   2461820
 
   2461929
 
  PDF   STATS   ENSEMBL   UCSC 
 
  110
 
  6
 
 1175
 
 tss;island
 
 0.557598
 
 0.9892281
 
 HES5 (margin: PANK4; HES5)
 
  6.5156565
 
 2.16e-03
 
 1.45e-02
  
 
  
 2
 
 197457277
 
 197457430
 
  PDF   STATS   ENSEMBL   UCSC 
 
  154
 
  6
 
 24975
 
 island
 
 0.594782
 
 0.0166415
 
 HECW2 (margin: HECW2)
 
  6.5012762
 
 2.16e-03
 
 1.45e-02
  
 
  
 16
 
  90114063
 
  90114307
 
  PDF   STATS   ENSEMBL   UCSC 
 
  245
 
  6
 
 15437
 
 tss
 
 0.476131
 
 0.0150253
 
 URAHP (margin: GAS8; URAHP; PRDM7)
 
  6.4822936
 
 2.16e-03
 
 1.45e-02
  
 
  
 16
 
  86370962
 
  86371442
 
  PDF   STATS   ENSEMBL   UCSC 
 
  481
 
  6
 
 14883
 
 gene
 
 0.085211
 
 0.9014852
 
 LINC00917 (margin: RP11-158I3.3; LINC00917)
 
  6.4571255
 
 2.16e-03
 
 1.45e-02
  
 
  
 3
 
  47620439
 
  47620545
 
  PDF   STATS   ENSEMBL   UCSC 
 
  107
 
  6
 
 28288
 
 tss;island
 
 0.761374
 
 0.0259596
 
 CSPG5 (margin: CSPG5; SMARCC1)
 
  6.4542814
 
 2.16e-03
 
 1.45e-02
  
 
  
 5
 
  52083947
 
  52084055
 
  PDF   STATS   ENSEMBL   UCSC 
 
  109
 
  6
 
 31610
 
 gene
 
 0.012348
 
 0.4217590
 
 ITGA1; PELO; CTD-2288O8.1 (margin: ITGA1; PELO; CTD-2288O8.1)
 
  6.4454161
 
 2.16e-03
 
 1.45e-02
  
 
  
 4
 
  25090198
 
  25090665
 
  PDF   STATS   ENSEMBL   UCSC 
 
  468
 
  6
 
 30958
 
 island
 
 0.331168
 
 0.9720687
 
  (margin: )
 
  6.4224069
 
 2.16e-03
 
 1.45e-02
  
 
  
 2
 
 228582688
 
 228582820
 
  PDF   STATS   ENSEMBL   UCSC 
 
  133
 
  6
 
 25168
 
 island
 
 0.309152
 
 0.0019382
 
 SLC19A3 (margin: SLC19A3)
 
  6.3750607
 
 2.16e-03
 
 1.45e-02
  
 
  
 20
 
    749264
 
    749620
 
  PDF   STATS   ENSEMBL   UCSC 
 
  357
 
  6
 
 25529
 
 tss
 
 0.013551
 
 0.6270626
 
 SLC52A3 (margin: SLC52A3)
 
  6.3589425
 
 2.16e-03
 
 1.45e-02
  
 
  
 6
 
  33172442
 
  33172572
 
  PDF   STATS   ENSEMBL   UCSC 
 
  131
 
  6
 
 34227
 
 gene
 
 0.010851
 
 0.4551949
 
 SLC39A7; HSD17B8 (margin: RNY4P10; SLC39A7; HSD17B8; MIR219-1; RING1; ZNF70P1; RXRB)
 
  6.3586262
 
 2.16e-03
 
 1.45e-02
  
 
  
 8
 
  67344553
 
  67344665
 
  PDF   STATS   ENSEMBL   UCSC 
 
  113
 
  6
 
 39742
 
 tss
 
 0.413016
 
 0.9817923
 
 RRS1; ADHFE1 (margin: RRS1; ADHFE1; RP11-346I3.4)
 
  6.3425057
 
 2.16e-03
 
 1.45e-02
  
 
  
 13
 
 114880761
 
 114881194
 
  PDF   STATS   ENSEMBL   UCSC 
 
  434
 
  6
 
 11316
 
 gene;island
 
 0.296170
 
 0.9700362
 
 RASA3 (margin: RASA3; RASA3-IT1)
 
  6.3401390
 
 2.16e-03
 
 1.45e-02
  
 
  
 1
 
  99729757
 
  99729807
 
  PDF   STATS   ENSEMBL   UCSC 
 
   51
 
  6
 
 1773
 
 tss;island
 
 0.794715
 
 0.0677533
 
 LPPR4 (margin: LPPR4)
 
  6.3352053
 
 2.16e-03
 
 1.45e-02
  
 
  
 7
 
  12610355
 
  12610833
 
  PDF   STATS   ENSEMBL   UCSC 
 
  479
 
  6
 
 36896
 
 gene
 
 0.684784
 
 0.0460062
 
 SCIN; AC005281.2 (margin: SCIN; AC005281.2)
 
  6.3248578
 
 2.16e-03
 
 1.45e-02
  
 
  
 7
 
 134576049
 
 134576386
 
  PDF   STATS   ENSEMBL   UCSC 
 
  338
 
  6
 
 37233
 
 gene
 
 0.011827
 
 0.4747369
 
 CALD1 (margin: CALD1)
 
  6.3125814
 
 2.16e-03
 
 1.45e-02
  
 
  
 3
 
  44753865
 
  44754131
 
  PDF   STATS   ENSEMBL   UCSC 
 
  267
 
  8
 
 28260
 
 tss
 
 0.395013
 
 0.9765951
 
 ZNF502; SOCS5P3 (margin: ZNF502; SOCS5P3)
 
  6.3109588
 
 1.86e-03
 
 1.45e-02
  
 
  
 10
 
  97667581
 
  97667923
 
  PDF   STATS   ENSEMBL   UCSC 
 
  343
 
  6
 
 5969
 
 island
 
 0.546975
 
 0.0191001
 
 RP11-248J23.7; C10orf131; ENTPD1-AS1 (margin: RP11-248J23.7; C10orf131; ENTPD1-AS1)
 
  6.2966201
 
 2.16e-03
 
 1.45e-02
  
 
  
 18
 
  47792917
 
  47793175
 
  PDF   STATS   ENSEMBL   UCSC 
 
  259
 
  6
 
 19274
 
 tss
 
 0.281794
 
 0.0071494
 
 CCDC11; MBD1 (margin: CCDC11; MBD1)
 
  6.2886068
 
 2.16e-03
 
 1.45e-02
  
 
  
 11
 
  65779192
 
  65779343
 
  PDF   STATS   ENSEMBL   UCSC 
 
  152
 
  6
 
 7456
 
 tss
 
 0.237062
 
 0.9507591
 
 CST6 (margin: BANF1; CST6; EIF1AD; CATSPER1)
 
  6.2625451
 
 2.16e-03
 
 1.45e-02
  
 
  
 5
 
 100238977
 
 100239071
 
  PDF   STATS   ENSEMBL   UCSC 
 
   95
 
  6
 
 32196
 
 tss
 
 0.107932
 
 0.0044653
 
 ST8SIA4 (margin: ST8SIA4)
 
  6.2560124
 
 2.16e-03
 
 1.45e-02
  
 
  
 1
 
 239549577
 
 239549853
 
  PDF   STATS   ENSEMBL   UCSC 
 
  277
 
  6
 
 4370
 
 island
 
 0.786841
 
 0.0169963
 
 CHRM3 (margin: CHRM3)
 
  6.2450445
 
 2.16e-03
 
 1.45e-02
  
 
  
 10
 
  94833042
 
  94833214
 
  PDF   STATS   ENSEMBL   UCSC 
 
  173
 
  6
 
 5234
 
 tss;island
 
 0.865288
 
 0.0508479
 
 CYP26A1 (margin: CYP26C1; CYP26A1)
 
  6.1833534
 
 2.16e-03
 
 1.45e-02
  
 
  
 11
 
  69468789
 
  69469175
 
  PDF   STATS   ENSEMBL   UCSC 
 
  387
 
  6
 
 6782
 
 gene
 
 0.932932
 
 0.3164531
 
 CCND1; ORAOV1 (margin: CCND1; ORAOV1)
 
  6.1690509
 
 2.16e-03
 
 1.45e-02
  
 
  
 19
 
  45996372
 
  45996644
 
  PDF   STATS   ENSEMBL   UCSC 
 
  273
 
  6
 
 20099
 
 gene;island
 
 0.587822
 
 0.0301701
 
 PPM1N; RTN2 (margin: PPM1N; RTN2)
 
  6.1609092
 
 2.16e-03
 
 1.45e-02
  
 
  
 1
 
 220921855
 
 220922217
 
  PDF   STATS   ENSEMBL   UCSC 
 
  363
 
  6
 
 1020
 
 gene
 
 0.456907
 
 0.0115957
 
 MARC2 (margin: MARC2)
 
  6.1430165
 
 2.16e-03
 
 1.45e-02
  
 
  
 15
 
  52588020
 
  52588279
 
  PDF   STATS   ENSEMBL   UCSC 
 
  260
 
  6
 
 13679
 
 tss
 
 0.449042
 
 0.0127651
 
 MYO5C (margin: MYO5C; MYO5A)
 
  6.1215085
 
 2.16e-03
 
 1.45e-02
  
 
  
 17
 
  21454528
 
  21455116
 
  PDF   STATS   ENSEMBL   UCSC 
 
  589
 
  6
 
 18125
 
 island
 
 0.015745
 
 0.4603936
 
 RP11-822E23.3; C17orf51 (margin: RP11-822E23.2; RP11-822E23.3; C17orf51)
 
  6.1214063
 
 2.16e-03
 
 1.45e-02
  
 
  
 14
 
  89878562
 
  89878733
 
  PDF   STATS   ENSEMBL   UCSC 
 
  172
 
  6
 
 12170
 
 gene;island
 
 0.163694
 
 0.9238812
 
 FOXN3; RP11-33N16.3 (margin: RP11-33N16.2; FOXN3-AS1; FOXN3; RP11-33N16.3)
 
  6.1132098
 
 2.16e-03
 
 1.45e-02
  
 
  
 4
 
 118006747
 
 118006832
 
  PDF   STATS   ENSEMBL   UCSC 
 
   86
 
  6
 
 30559
 
 tss
 
 0.518175
 
 0.0225544
 
 TRAM1L1 (margin: TRAM1L1)
 
  6.1042897
 
 2.16e-03
 
 1.45e-02
  
 
  
 12
 
  16758692
 
  16759029
 
  PDF   STATS   ENSEMBL   UCSC 
 
  338
 
  6
 
 9072
 
 gene
 
 0.637440
 
 0.0490199
 
 MGST1; LMO3 (margin: MGST1; LMO3)
 
  6.1020982
 
 2.16e-03
 
 1.45e-02
  
 
  
 8
 
 144660395
 
 144660772
 
  PDF   STATS   ENSEMBL   UCSC 
 
  378
 
  6
 
 40673
 
 island
 
 0.546836
 
 0.9903039
 
 RP11-661A12.9; RP11-661A12.7; NAPRT1; EEF1D (margin: RP11-661A12.9; RP11-661A12.7; MROH6; NAPRT1; EEF1D)
 
  6.0724020
 
 2.16e-03
 
 1.45e-02
  
 
  
 6
 
 111408745
 
 111408840
 
  PDF   STATS   ENSEMBL   UCSC 
 
   96
 
  6
 
 36418
 
 island
 
 0.470901
 
 0.0138620
 
 SLC16A10 (margin: SLC16A10; RNU6-960P)
 
  6.0533150
 
 2.16e-03
 
 1.45e-02
  
 
  
 2
 
  33359198
 
  33359688
 
  PDF   STATS   ENSEMBL   UCSC 
 
  491
 
  6
 
 22641
 
 gene
 
 0.109078
 
 0.9186882
 
 LTBP1 (margin: LTBP1)
 
  6.0377588
 
 2.16e-03
 
 1.45e-02
  
 
  
 5
 
  88179235
 
  88179539
 
  PDF   STATS   ENSEMBL   UCSC 
 
  305
 
  6
 
 31682
 
 gene;island
 
 0.331203
 
 0.0090862
 
 MEF2C-AS1; MEF2C (margin: MEF2C-AS1; MEF2C)
 
  6.0101299
 
 2.16e-03
 
 1.45e-02
  
 
  
 13
 
  28493913
 
  28494161
 
  PDF   STATS   ENSEMBL   UCSC 
 
  249
 
  6
 
 11365
 
 tss
 
 0.487884
 
 0.9439982
 
 PDX1; PDX1-AS1 (margin: PDX1; PDX1-AS1)
 
  6.0038396
 
 2.16e-03
 
 1.45e-02
  
 
  
 11
 
    826837
 
    827509
 
  PDF   STATS   ENSEMBL   UCSC 
 
  673
 
  6
 
 7031
 
 tss
 
 0.496828
 
 0.0188474
 
 PNPLA2; EFCAB4A; AP006621.8 (margin: PNPLA2; EFCAB4A; CD151; AP006621.8; POLR2L)
 
  5.9819708
 
 2.16e-03
 
 1.45e-02
  
 
  
 19
 
  13915583
 
  13915877
 
  PDF   STATS   ENSEMBL   UCSC 
 
  295
 
  6
 
 19883
 
 gene;island
 
 0.977923
 
 0.4378045
 
 ZSWIM4 (margin: ZSWIM4; CTD-3252C9.2; RN7SL619P)
 
  5.9801198
 
 2.16e-03
 
 1.45e-02
  
 
  
 10
 
  49893336
 
  49893463
 
  PDF   STATS   ENSEMBL   UCSC 
 
  128
 
  6
 
 5069
 
 tss
 
 0.312916
 
 0.9329553
 
 WDFY4 (margin: WDFY4)
 
  5.9783643
 
 2.16e-03
 
 1.45e-02
  
 
  
 7
 
   4872926
 
   4873549
 
  PDF   STATS   ENSEMBL   UCSC 
 
  624
 
  6
 
 36860
 
 gene;island
 
 0.267219
 
 0.9580844
 
 RADIL (margin: RADIL)
 
  5.9771387
 
 2.16e-03
 
 1.45e-02
  
 
  
 2
 
 182521874
 
 182521926
 
  PDF   STATS   ENSEMBL   UCSC 
 
   53
 
  6
 
 23762
 
 tss;island
 
 0.459963
 
 0.0168135
 
 CERKL (margin: CERKL)
 
  5.9293708
 
 2.16e-03
 
 1.45e-02
  
 
  
 5
 
  43037123
 
  43037666
 
  PDF   STATS   ENSEMBL   UCSC 
 
  544
 
  6
 
 32766
 
 island
 
 0.509784
 
 0.9847336
 
 CTD-2201E18.3; ANXA2R (margin: CTD-2035E11.4; AC025171.1; CTD-2201E18.3; ANXA2R)
 
  5.9180307
 
 2.16e-03
 
 1.45e-02
  
 
  
 14
 
 101525784
 
 101526140
 
  PDF   STATS   ENSEMBL   UCSC 
 
  357
 
  6
 
 12630
 
 tss
 
 0.072769
 
 0.8823186
 
 MIR154; MIR496 (margin: MIR381HG; MIR889; MIR544A; MIR655; AL132709.3; MIR487A; MIR134; MIR485; MIR323B; MIR154; MIR496; MIR377; MIR541; MIR409; MIR412; MIR369; MIR410; MIR656; MEG9; MIR382)
 
  5.8957405
 
 2.16e-03
 
 1.45e-02
  
 
  
 17
 
  21279561
 
  21279874
 
  PDF   STATS   ENSEMBL   UCSC 
 
  314
 
  6
 
 18118
 
 island
 
 0.547441
 
 0.9840273
 
 KCNJ12 (margin: KCNJ12)
 
  5.8564446
 
 2.16e-03
 
 1.45e-02
  
 
  
 5
 
 180018495
 
 180018735
 
  PDF   STATS   ENSEMBL   UCSC 
 
  241
 
  6
 
 32560
 
 tss
 
 0.371413
 
 0.9682548
 
 SCGB3A1 (margin: AC122714.1; SCGB3A1; FLT4)
 
  5.8556413
 
 2.16e-03
 
 1.45e-02
  
 
  
 2
 
 179278332
 
 179278855
 
  PDF   STATS   ENSEMBL   UCSC 
 
  524
 
  6
 
 22945
 
 gene;island
 
 0.013720
 
 0.3506432
 
 AC009948.5 (margin: RP11-65L3.1; AC009948.5)
 
  5.8386142
 
 2.16e-03
 
 1.45e-02
  
 
  
 3
 
  96533511
 
  96534005
 
  PDF   STATS   ENSEMBL   UCSC 
 
  495
 
  6
 
 27871
 
 gene;island
 
 0.920773
 
 0.2526366
 
 EPHA6 (margin: EPHA6)
 
  5.8350087
 
 2.16e-03
 
 1.45e-02
  
 
  
 17
 
  71161160
 
  71161502
 
  PDF   STATS   ENSEMBL   UCSC 
 
  343
 
  6
 
 16745
 
 gene
 
 0.588200
 
 0.0204805
 
 RP11-143K11.5; SSTR2; POLR3KP2 (margin: RP11-143K11.5; SSTR2; RP11-143K11.1; POLR3KP2)
 
  5.8342201
 
 2.16e-03
 
 1.45e-02
  
 
  
 13
 
  36920644
 
  36921174
 
  PDF   STATS   ENSEMBL   UCSC 
 
  531
 
 13
 
 11131
 
 gene;island
 
 0.117488
 
 0.0059811
 
 SPG20OS; SPG20 (margin: SPG20OS; AL139377.1; SPG20)
 
  5.8294794
 
 1.91e-03
 
 1.45e-02
  
 
  
 4
 
 155664941
 
 155665106
 
  PDF   STATS   ENSEMBL   UCSC 
 
  166
 
  6
 
 30647
 
 tss
 
 0.885266
 
 0.1074596
 
 LRAT (margin: LRAT; RP11-21G20.3)
 
  5.8032628
 
 2.16e-03
 
 1.45e-02
  
 
  
 2
 
 131513608
 
 131513927
 
  PDF   STATS   ENSEMBL   UCSC 
 
  320
 
  6
 
 22867
 
 gene;island
 
 0.470951
 
 0.0299294
 
 AMER3; AC140481.8 (margin: AMER3; AC140481.8)
 
  5.8024510
 
 2.16e-03
 
 1.45e-02
  
 
  
 17
 
   1958851
 
   1959132
 
  PDF   STATS   ENSEMBL   UCSC 
 
  282
 
  6
 
 16327
 
 gene;tss;island
 
 0.208448
 
 0.9622385
 
 HIC1 (margin: HIC1; RP11-667K14.3; MIR132; MIR212; SMG6)
 
  5.7902367
 
 2.16e-03
 
 1.45e-02
  
 
  
 1
 
 239550177
 
 239550578
 
  PDF   STATS   ENSEMBL   UCSC 
 
  402
 
  6
 
 4371
 
 island
 
 0.780983
 
 0.1009708
 
 CHRM3 (margin: CHRM3)
 
  5.7865212
 
 2.16e-03
 
 1.45e-02
  
 
  
 21
 
  46424715
 
  46424976
 
  PDF   STATS   ENSEMBL   UCSC 
 
  262
 
  6
 
 26591
 
 tss;island
 
 0.024753
 
 0.5728399
 
 LINC00162 (margin: LINC00163; AP001505.9; LINC00162)
 
  5.7768355
 
 2.16e-03
 
 1.45e-02
  
 
  
 14
 
 104583632
 
 104584279
 
  PDF   STATS   ENSEMBL   UCSC 
 
  648
 
  6
 
 13202
 
 island
 
 0.726304
 
 0.0357608
 
 MIR203 (margin: ASPG; MIR203)
 
  5.7643754
 
 2.16e-03
 
 1.45e-02
  
 
  
 10
 
  35930436
 
  35930534
 
  PDF   STATS   ENSEMBL   UCSC 
 
   99
 
  6
 
 5029
 
 tss;island
 
 0.364800
 
 0.0087612
 
 FZD8; MIR4683 (margin: FZD8; MIR4683)
 
  5.7423468
 
 2.16e-03
 
 1.45e-02
  
 
  
 7
 
  31232532
 
  31232861
 
  PDF   STATS   ENSEMBL   UCSC 
 
  330
 
  6
 
 38391
 
 island
 
 0.469134
 
 0.0082102
 
  (margin: )
 
  5.7402678
 
 2.16e-03
 
 1.45e-02
  
 
  
 6
 
 108487063
 
 108487183
 
  PDF   STATS   ENSEMBL   UCSC 
 
  121
 
  6
 
 35158
 
 tss;island
 
 0.381778
 
 0.9693834
 
 NR2E1; OSTM1 (margin: OSTM1-AS1; NR2E1; OSTM1)
 
  5.7207931
 
 2.16e-03
 
 1.45e-02
  
 
  
 11
 
  67806337
 
  67806437
 
  PDF   STATS   ENSEMBL   UCSC 
 
  101
 
  6
 
 7505
 
 tss
 
 0.009860
 
 0.3734378
 
 TCIRG1 (margin: ALDH3B1; NDUFS8; MIR4691; TCIRG1; RP11-802E16.3; RP5-901A4.1)
 
  5.7126210
 
 2.16e-03
 
 1.45e-02
  
 
  
 15
 
  60296138
 
  60296469
 
  PDF   STATS   ENSEMBL   UCSC 
 
  332
 
  6
 
 14189
 
 island
 
 0.621428
 
 0.0272041
 
 FOXB1 (margin: FOXB1)
 
  5.6975075
 
 2.16e-03
 
 1.45e-02
  
 
  
 8
 
  41166990
 
  41167278
 
  PDF   STATS   ENSEMBL   UCSC 
 
  289
 
  6
 
 39667
 
 tss;island
 
 0.599309
 
 0.9866917
 
 SFRP1 (margin: RNU6-895P; SFRP1)
 
  5.6863371
 
 2.16e-03
 
 1.45e-02
  
 
  
 13
 
 113676726
 
 113677325
 
  PDF   STATS   ENSEMBL   UCSC 
 
  600
 
  6
 
 11269
 
 gene;island
 
 0.331854
 
 0.9615491
 
 MCF2L (margin: MCF2L)
 
  5.6845892
 
 2.16e-03
 
 1.45e-02
  
 
  
 5
 
 142065539
 
 142065819
 
  PDF   STATS   ENSEMBL   UCSC 
 
  281
 
  6
 
 31843
 
 gene
 
 0.036642
 
 0.5778664
 
 FGF1 (margin: FGF1)
 
  5.6691166
 
 2.16e-03
 
 1.45e-02
  
 
  
 19
 
  19976569
 
  19976679
 
  PDF   STATS   ENSEMBL   UCSC 
 
  111
 
  6
 
 20654
 
 tss
 
 0.684678
 
 0.0203324
 
 ZNF253 (margin: CTC-559E9.12; ZNF253; CTC-559E9.10)
 
  5.6680162
 
 2.16e-03
 
 1.45e-02
  
 
  
 6
 
 166401464
 
 166401700
 
  PDF   STATS   ENSEMBL   UCSC 
 
  237
 
  6
 
 34542
 
 gene;island
 
 0.294165
 
 0.0032170
 
 PDE10A; LINC00473 (margin: PDE10A; LINC00473)
 
  5.6620011
 
 2.16e-03
 
 1.45e-02
  
 
  
 8
 
 144640306
 
 144640403
 
  PDF   STATS   ENSEMBL   UCSC 
 
   98
 
  6
 
 39952
 
 tss
 
 0.032462
 
 0.6116509
 
 GSDMD (margin: GSDMD; RP11-661A12.5; MROH6)
 
  5.5843194
 
 2.16e-03
 
 1.45e-02
  
 
  
 6
 
  32410690
 
  32411185
 
  PDF   STATS   ENSEMBL   UCSC 
 
  496
 
  6
 
 34114
 
 gene
 
 0.031762
 
 0.6043268
 
 HLA-DRA (margin: HLA-DRA)
 
  5.5839821
 
 2.16e-03
 
 1.45e-02
  
 
  
 12
 
  85673860
 
  85674022
 
  PDF   STATS   ENSEMBL   UCSC 
 
  163
 
  6
 
 9901
 
 tss
 
 0.561979
 
 0.9831254
 
 ALX1 (margin: ALX1)
 
  5.5742365
 
 2.16e-03
 
 1.45e-02
  
 
  
 3
 
 170746029
 
 170746291
 
  PDF   STATS   ENSEMBL   UCSC 
 
  263
 
  6
 
 29661
 
 island
 
 0.379421
 
 0.9728515
 
 SLC2A2 (margin: SLC2A2)
 
  5.5287798
 
 2.16e-03
 
 1.45e-02
  
 
  
 1
 
   3044741
 
   3045166
 
  PDF   STATS   ENSEMBL   UCSC 
 
  426
 
  6
 
 101
 
 gene
 
 0.308099
 
 0.9681457
 
 PRDM16 (margin: PRDM16; RP1-163G9.2)
 
  5.5281118
 
 2.16e-03
 
 1.45e-02
  
 
  
 11
 
  65547881
 
  65548028
 
  PDF   STATS   ENSEMBL   UCSC 
 
  148
 
  6
 
 7440
 
 tss
 
 0.018850
 
 0.5318193
 
 AP5B1; AP001266.1 (margin: RP11-770G2.2; OVOL1; AP5B1; AP001266.1; RP11-770G2.4)
 
  5.5233220
 
 2.16e-03
 
 1.45e-02
  
 
  
 1
 
 244624521
 
 244624883
 
  PDF   STATS   ENSEMBL   UCSC 
 
  363
 
  6
 
 4394
 
 island
 
 0.418953
 
 0.0110950
 
 C1orf101 (margin: C1orf101; ADSS)
 
  5.4736883
 
 2.16e-03
 
 1.45e-02
  
 
  
 6
 
   6007259
 
   6007769
 
  PDF   STATS   ENSEMBL   UCSC 
 
  511
 
  6
 
 35407
 
 island
 
 0.401251
 
 0.9761190
 
 NRN1 (margin: NRN1)
 
  5.4655342
 
 2.16e-03
 
 1.45e-02
  
 
  
 17
 
  43922122
 
  43922423
 
  PDF   STATS   ENSEMBL   UCSC 
 
  302
 
  6
 
 16638
 
 gene
 
 0.091335
 
 0.8289965
 
 SPPL2C; MAPT-AS1 (margin: CRHR1; SPPL2C; MAPT-AS1)
 
  5.4415403
 
 2.16e-03
 
 1.45e-02
  
 
  
 6
 
 112375186
 
 112375333
 
  PDF   STATS   ENSEMBL   UCSC 
 
  148
 
  6
 
 35180
 
 tss
 
 0.152571
 
 0.9276259
 
 WISP3 (margin: WISP3)
 
  5.4283714
 
 2.16e-03
 
 1.45e-02
  
 
  
 22
 
  18593294
 
  18593441
 
  PDF   STATS   ENSEMBL   UCSC 
 
  148
 
  6
 
 26948
 
 tss
 
 0.551085
 
 0.0179325
 
 PEX26; TUBA8 (margin: PEX26; XXbac-B476C20.10; TUBA8; ARL2BPP10)
 
  5.4246725
 
 2.16e-03
 
 1.45e-02
  
 
  
 8
 
  60031823
 
  60031944
 
  PDF   STATS   ENSEMBL   UCSC 
 
  122
 
  6
 
 39722
 
 tss;island
 
 0.284781
 
 0.9251340
 
 RP11-25K19.1; TOX (margin: RP11-25K19.1; TOX)
 
  5.4203426
 
 2.16e-03
 
 1.45e-02
  
 
  
 5
 
   1930801
 
   1931781
 
  PDF   STATS   ENSEMBL   UCSC 
 
  981
 
  8
 
 32646
 
 island
 
 0.428493
 
 0.9384376
 
  (margin: RP11-259O2.1)
 
  5.3879200
 
 1.86e-03
 
 1.45e-02
  
 
  
 21
 
  22369802
 
  22370444
 
  PDF   STATS   ENSEMBL   UCSC 
 
  643
 
  6
 
 26495
 
 tss;island
 
 0.817345
 
 0.2793395
 
 NCAM2 (margin: NCAM2)
 
  5.3781715
 
 2.16e-03
 
 1.45e-02
  
 
  
 6
 
  29601398
 
  29601705
 
  PDF   STATS   ENSEMBL   UCSC 
 
  308
 
  6
 
 34750
 
 tss;island
 
 0.940755
 
 0.2279017
 
 GABBR1 (margin: GABBR1; SUMO2P1)
 
  5.3753062
 
 2.16e-03
 
 1.45e-02
  
 
  
 17
 
  54670875
 
  54671575
 
  PDF   STATS   ENSEMBL   UCSC 
 
  701
 
  6
 
 18577
 
 island
 
 0.485699
 
 0.0249184
 
 NOG (margin: NOG)
 
  5.3721704
 
 2.16e-03
 
 1.45e-02
  
 
  
 11
 
  46366643
 
  46366884
 
  PDF   STATS   ENSEMBL   UCSC 
 
  242
 
  6
 
 7276
 
 tss
 
 0.169284
 
 0.9014546
 
 DGKZ (margin: DGKZ)
 
  5.3658601
 
 2.16e-03
 
 1.45e-02
  
 
  
 17
 
  27045043
 
  27045302
 
  PDF   STATS   ENSEMBL   UCSC 
 
  260
 
  6
 
 17218
 
 tss;island
 
 0.008018
 
 0.3385586
 
 RPL23A; RAB34 (margin: RPL23A; SNORD42B; SNORD4A; SNORD42A; SNORD4B; NEK8; AC010761.14; PROCA1; RAB34; AC010761.8; TLCD1)
 
  5.3365961
 
 2.16e-03
 
 1.45e-02
  
 
  
 2
 
 160761262
 
 160761622
 
  PDF   STATS   ENSEMBL   UCSC 
 
  361
 
  6
 
 23683
 
 tss
 
 0.357431
 
 0.9609495
 
 LY75; LY75-CD302 (margin: LY75; LY75-CD302)
 
  5.3338455
 
 2.16e-03
 
 1.45e-02
  
 
  
 10
 
  18629497
 
  18629677
 
  PDF   STATS   ENSEMBL   UCSC 
 
  181
 
  6
 
 4522
 
 gene
 
 0.440669
 
 0.9595235
 
 CACNB2 (margin: CACNB2)
 
  5.3223243
 
 2.16e-03
 
 1.45e-02
  
 
  
 19
 
  37825307
 
  37825446
 
  PDF   STATS   ENSEMBL   UCSC 
 
  140
 
  6
 
 20736
 
 tss;island
 
 0.883959
 
 0.4653724
 
 HKR1 (margin: HKR1; CTD-3220F14.2)
 
  5.3180138
 
 2.16e-03
 
 1.45e-02
  
 
  
 11
 
  64937570
 
  64937697
 
  PDF   STATS   ENSEMBL   UCSC 
 
  128
 
  6
 
 7419
 
 tss
 
 0.935295
 
 0.2432610
 
 SPDYC (margin: SPDYC; CAPN1; AP003068.17; AP003068.18; PGAM1P8; AP003068.23)
 
  5.3128914
 
 2.16e-03
 
 1.45e-02
  
 
  
 6
 
 127664674
 
 127664950
 
  PDF   STATS   ENSEMBL   UCSC 
 
  277
 
  6
 
 35216
 
 tss;island
 
 0.027107
 
 0.4641958
 
 ECHDC1 (margin: YWHAZP4; ECHDC1)
 
  5.2924141
 
 2.16e-03
 
 1.45e-02
  
 
  
 11
 
   3239953
 
   3240399
 
  PDF   STATS   ENSEMBL   UCSC 
 
  447
 
  6
 
 6478
 
 gene;island
 
 0.046947
 
 0.7947594
 
 MRGPRG-AS1; MRGPRG (margin: MRGPRG-AS1; AC109309.4; MRGPRG; MRGPRE)
 
  5.2816058
 
 2.16e-03
 
 1.45e-02
  
 
  
 4
 
 165304443
 
 165304540
 
  PDF   STATS   ENSEMBL   UCSC 
 
   98
 
  6
 
 30663
 
 tss
 
 0.318970
 
 0.9527567
 
 MARCH1 (margin: MARCH1)
 
  5.2287906
 
 2.16e-03
 
 1.45e-02
  
 
  
 7
 
  98972104
 
  98972197
 
  PDF   STATS   ENSEMBL   UCSC 
 
   94
 
  6
 
 37752
 
 tss;island
 
 0.442184
 
 0.0300379
 
 ARPC1A; ARPC1B (margin: ARPC1A; ARPC1B)
 
  5.2168867
 
 2.16e-03
 
 1.45e-02
  
 
  
 15
 
  65203834
 
  65204146
 
  PDF   STATS   ENSEMBL   UCSC 
 
  313
 
  6
 
 14222
 
 island
 
 0.409145
 
 0.0197010
 
 AC069368.3; ANKDD1A (margin: AC069368.3; ANKDD1A; RN7SL348P)
 
  5.2069816
 
 2.16e-03
 
 1.45e-02
  
 
  
 10
 
   5566543
 
   5566908
 
  PDF   STATS   ENSEMBL   UCSC 
 
  366
 
  6
 
 4926
 
 tss;island
 
 0.197042
 
 0.8860647
 
 CALML3; CALML3-AS1; RP11-116G8.5 (margin: CALML3; CALML3-AS1; RP11-116G8.5)
 
  5.2052790
 
 2.16e-03
 
 1.45e-02
  
 
  
 14
 
  69951211
 
  69951422
 
  PDF   STATS   ENSEMBL   UCSC 
 
  212
 
  6
 
 12481
 
 tss
 
 0.480368
 
 0.0241340
 
 PLEKHD1 (margin: PLEKHD1)
 
  5.1897031
 
 2.16e-03
 
 1.45e-02
  
 
  
 19
 
   7852051
 
   7852238
 
  PDF   STATS   ENSEMBL   UCSC 
 
  188
 
  6
 
 20407
 
 tss;island
 
 0.169891
 
 0.9314352
 
 CLEC4GP1 (margin: CLEC4GP1)
 
  5.1558756
 
 2.16e-03
 
 1.45e-02
  
 
  
 20
 
   1246703
 
   1246934
 
  PDF   STATS   ENSEMBL   UCSC 
 
  232
 
  6
 
 25536
 
 tss
 
 0.429381
 
 0.9352164
 
 RAD21L1; SNPH (margin: RAD21L1; SNPH)
 
  5.1472050
 
 2.16e-03
 
 1.45e-02
  
 
  
 10
 
 128077307
 
 128077376
 
  PDF   STATS   ENSEMBL   UCSC 
 
   70
 
  6
 
 5433
 
 tss;island
 
 0.587569
 
 0.9795670
 
 ADAM12 (margin: ADAM12)
 
  5.1387765
 
 2.16e-03
 
 1.45e-02
  
 
  
 8
 
  33457483
 
  33457822
 
  PDF   STATS   ENSEMBL   UCSC 
 
  340
 
  6
 
 39642
 
 tss
 
 0.394121
 
 0.9651037
 
 DUSP26 (margin: RP11-317N12.1; DUSP26)
 
  5.1282795
 
 2.16e-03
 
 1.45e-02
  
 
  
 19
 
  12624679
 
  12624832
 
  PDF   STATS   ENSEMBL   UCSC 
 
  154
 
  6
 
 20498
 
 tss
 
 0.579203
 
 0.9829155
 
 ZNF709 (margin: CTD-3105H18.8; CTD-3105H18.10; CTD-3105H18.9; CTD-3105H18.11; PPIAP20; ZNF709; ZNF564; CTD-2192J16.20)
 
  5.1072164
 
 2.16e-03
 
 1.45e-02
  
 
  
 15
 
  75470777
 
  75471218
 
  PDF   STATS   ENSEMBL   UCSC 
 
  442
 
  6
 
 14325
 
 island
 
 0.967945
 
 0.6436655
 
  (margin: RPL36AP45)
 
  5.0968689
 
 2.16e-03
 
 1.45e-02
  
 
  
 20
 
  44098223
 
  44098387
 
  PDF   STATS   ENSEMBL   UCSC 
 
  165
 
  6
 
 25776
 
 tss
 
 0.016406
 
 0.3897111
 
 WFDC2; AL031663.1 (margin: WFDC2; AL031663.1)
 
  5.0421770
 
 2.16e-03
 
 1.45e-02
  
 
  
 19
 
  51225848
 
  51226557
 
  PDF   STATS   ENSEMBL   UCSC 
 
  710
 
  6
 
 20984
 
 tss;island
 
 0.522912
 
 0.9601491
 
 CLEC11A (margin: CLEC11A; SHANK1)
 
  5.0415357
 
 2.16e-03
 
 1.45e-02
  
 
  
 7
 
   1022471
 
   1022806
 
  PDF   STATS   ENSEMBL   UCSC 
 
  336
 
  6
 
 37392
 
 tss
 
 0.489814
 
 0.9715587
 
 CYP2W1 (margin: CYP2W1; COX19)
 
  5.0383605
 
 2.16e-03
 
 1.45e-02
  
 
  
 11
 
 128775679
 
 128775750
 
  PDF   STATS   ENSEMBL   UCSC 
 
   72
 
  6
 
 7808
 
 tss
 
 0.006616
 
 0.1279501
 
 KCNJ5; C11orf45 (margin: KCNJ5; C11orf45)
 
  5.0271774
 
 2.16e-03
 
 1.45e-02
  
 
  
 1
 
 236228598
 
 236228837
 
  PDF   STATS   ENSEMBL   UCSC 
 
  240
 
  6
 
 2339
 
 tss;island
 
 0.737854
 
 0.9820009
 
 NID1 (margin: Y_RNA; NID1)
 
  5.0241333
 
 2.16e-03
 
 1.45e-02
  
 
  
 3
 
 112359488
 
 112359652
 
  PDF   STATS   ENSEMBL   UCSC 
 
  165
 
  6
 
 27896
 
 gene
 
 0.008889
 
 0.1962534
 
 CCDC80 (margin: CCDC80)
 
  4.9666453
 
 2.16e-03
 
 1.45e-02
  
 
  
 14
 
  55034288
 
  55034616
 
  PDF   STATS   ENSEMBL   UCSC 
 
  329
 
  6
 
 12409
 
 tss
 
 0.004361
 
 0.1632108
 
 SAMD4A (margin: SAMD4A)
 
  4.9606204
 
 2.16e-03
 
 1.45e-02
  
 
  
 6
 
 152702330
 
 152702660
 
  PDF   STATS   ENSEMBL   UCSC 
 
  331
 
  6
 
 34516
 
 gene
 
 0.340864
 
 0.9208742
 
 SYNE1-AS1; SYNE1 (margin: SYNE1-AS1; SYNE1)
 
  4.9494065
 
 2.16e-03
 
 1.45e-02
  
 
  
 10
 
  22726610
 
  22726885
 
  PDF   STATS   ENSEMBL   UCSC 
 
  276
 
  6
 
 5605
 
 island
 
 0.008728
 
 0.2469091
 
 SPAG6 (margin: SPAG6)
 
  4.9021070
 
 2.16e-03
 
 1.45e-02
  
 
  
 4
 
   4387697
 
   4387917
 
  PDF   STATS   ENSEMBL   UCSC 
 
  221
 
  6
 
 30267
 
 tss;island
 
 0.660379
 
 0.9642717
 
 NSG1 (margin: NSG1)
 
  4.8724785
 
 2.16e-03
 
 1.45e-02
  
 
  
 2
 
 119599459
 
 119600002
 
  PDF   STATS   ENSEMBL   UCSC 
 
  544
 
  6
 
 24644
 
 island
 
 0.636856
 
 0.9793290
 
 EN1 (margin: RP11-19E11.1; EN1)
 
  4.8603069
 
 2.16e-03
 
 1.45e-02
  
 
  
 1
 
 182584068
 
 182584578
 
  PDF   STATS   ENSEMBL   UCSC 
 
  511
 
  6
 
 4024
 
 island
 
 0.661253
 
 0.0595130
 
 RP11-317P15.4 (margin: RP11-317P15.4; RGS16)
 
  4.8464269
 
 2.16e-03
 
 1.45e-02
  
 
  
 8
 
  99076526
 
  99076837
 
  PDF   STATS   ENSEMBL   UCSC 
 
  312
 
  6
 
 40483
 
 island
 
 0.610609
 
 0.9653727
 
 C8orf47 (margin: C8orf47)
 
  4.8351216
 
 2.16e-03
 
 1.45e-02
  
 
  
 6
 
  74162051
 
  74162142
 
  PDF   STATS   ENSEMBL   UCSC 
 
   92
 
  6
 
 35070
 
 tss
 
 0.032792
 
 0.4048150
 
 MB21D1 (margin: MTO1; RNU6-975P; MB21D1)
 
  4.8256634
 
 2.16e-03
 
 1.45e-02
  
 
  
 4
 
 175443243
 
 175443867
 
  PDF   STATS   ENSEMBL   UCSC 
 
  625
 
  6
 
 30184
 
 gene;island
 
 0.244689
 
 0.0061252
 
 HPGD; RP11-440I14.2 (margin: HPGD; RP11-440I14.2)
 
  4.8233435
 
 2.16e-03
 
 1.45e-02
  
 
  
 13
 
  79183424
 
  79184101
 
  PDF   STATS   ENSEMBL   UCSC 
 
  678
 
  6
 
 11796
 
 island
 
 0.567422
 
 0.9710209
 
 RNF219-AS1 (margin: RNF219-AS1; RP11-52L5.6; POU4F1; RNF219)
 
  4.8070315
 
 2.16e-03
 
 1.45e-02
  
 
  
 7
 
  51384519
 
  51384676
 
  PDF   STATS   ENSEMBL   UCSC 
 
  158
 
  6
 
 37599
 
 tss
 
 0.338481
 
 0.0230038
 
 COBL (margin: COBL)
 
  4.7947089
 
 2.16e-03
 
 1.45e-02
  
 
  
 7
 
 140773422
 
 140774161
 
  PDF   STATS   ENSEMBL   UCSC 
 
  740
 
  6
 
 38967
 
 island
 
 0.700704
 
 0.0495421
 
 TMEM178B (margin: TMEM178B)
 
  4.7700693
 
 2.16e-03
 
 1.45e-02
  
 
  
 13
 
  74708078
 
  74708194
 
  PDF   STATS   ENSEMBL   UCSC 
 
  117
 
  6
 
 11477
 
 tss
 
 0.116825
 
 0.0069483
 
 KLF12 (margin: KLF12)
 
  4.7559222
 
 2.16e-03
 
 1.45e-02
  
 
  
 6
 
  27840105
 
  27840257
 
  PDF   STATS   ENSEMBL   UCSC 
 
  153
 
  6
 
 34727
 
 tss
 
 0.014174
 
 0.0005156
 
 HIST1H3I; HIST1H4L (margin: HIST1H2AL; HIST1H2BPS2; HIST1H1B; HIST1H3I; HIST1H4L)
 
  4.7534397
 
 2.16e-03
 
 1.45e-02
  
 
  
 6
 
 167263025
 
 167263454
 
  PDF   STATS   ENSEMBL   UCSC 
 
  430
 
  6
 
 34555
 
 gene
 
 0.308054
 
 0.9061350
 
 RPS6KA2 (margin: RPS6KA2; RP11-514O12.4)
 
  4.7343063
 
 2.16e-03
 
 1.45e-02
  
 
  
 13
 
 111972854
 
 111972997
 
  PDF   STATS   ENSEMBL   UCSC 
 
  144
 
  6
 
 11542
 
 tss
 
 0.583758
 
 0.9539720
 
 TEX29 (margin: TEX29)
 
  4.7296202
 
 2.16e-03
 
 1.45e-02
  
 
  
 8
 
  49468684
 
  49468988
 
  PDF   STATS   ENSEMBL   UCSC 
 
  305
 
  6
 
 40283
 
 island
 
 0.552412
 
 0.9698921
 
 RP11-770E5.1; RP11-567J20.2 (margin: RP11-770E5.1; RP11-567J20.2)
 
  4.7246926
 
 2.16e-03
 
 1.45e-02
  
 
  
 2
 
 233497957
 
 233498164
 
  PDF   STATS   ENSEMBL   UCSC 
 
  208
 
  6
 
 23953
 
 tss
 
 0.378354
 
 0.0249245
 
 EFHD1 (margin: EFHD1)
 
  4.7142957
 
 2.16e-03
 
 1.45e-02
  
 
  
 15
 
  90039805
 
  90039913
 
  PDF   STATS   ENSEMBL   UCSC 
 
  109
 
  6
 
 13888
 
 tss
 
 0.617174
 
 0.0584574
 
 RHCG (margin: RHCG; LINC00928)
 
  4.7024199
 
 2.16e-03
 
 1.45e-02
  
 
  
 17
 
  62777648
 
  62777777
 
  PDF   STATS   ENSEMBL   UCSC 
 
  130
 
  6
 
 16734
 
 gene;tss;island
 
 0.060473
 
 0.6091347
 
 hsa-mir-6080; PLEKHM1P (margin: hsa-mir-6080; RP11-927P21.4; PLEKHM1P)
 
  4.6964100
 
 2.16e-03
 
 1.45e-02
  
 
  
 5
 
 128300967
 
 128301185
 
  PDF   STATS   ENSEMBL   UCSC 
 
  219
 
  6
 
 32260
 
 tss
 
 0.155037
 
 0.0115851
 
 SLC27A6 (margin: SLC27A6)
 
  4.6883769
 
 2.16e-03
 
 1.45e-02
  
 
  
 4
 
   8271196
 
   8271414
 
  PDF   STATS   ENSEMBL   UCSC 
 
  219
 
  6
 
 30296
 
 tss
 
 0.430225
 
 0.9462976
 
 HTRA3 (margin: HTRA3)
 
  4.6828812
 
 2.16e-03
 
 1.45e-02
  
 
  
 1
 
 197880384
 
 197880923
 
  PDF   STATS   ENSEMBL   UCSC 
 
  540
 
  6
 
 2156
 
 tss;island
 
 0.418197
 
 0.8979913
 
 LHX9 (margin: C1orf53; LHX9)
 
  4.6812740
 
 2.16e-03
 
 1.45e-02
  
 
  
 3
 
 120626881
 
 120627170
 
  PDF   STATS   ENSEMBL   UCSC 
 
  290
 
  6
 
 29392
 
 island
 
 0.616596
 
 0.0464757
 
 STXBP5L (margin: STXBP5L)
 
  4.6741585
 
 2.16e-03
 
 1.45e-02
  
 
  
 4
 
  46391259
 
  46391929
 
  PDF   STATS   ENSEMBL   UCSC 
 
  671
 
 13
 
 29995
 
 gene;island
 
 0.910914
 
 0.2150730
 
 RP11-436F23.1; GABRA2 (margin: RP11-436F23.1; GABRA2)
 
  4.6655454
 
 1.91e-03
 
 1.45e-02
  
 
  
 11
 
  46354562
 
  46354635
 
  PDF   STATS   ENSEMBL   UCSC 
 
   74
 
  6
 
 7275
 
 tss
 
 0.771264
 
 0.9879434
 
 DGKZ (margin: CREB3L1; DGKZ)
 
  4.6622588
 
 2.16e-03
 
 1.45e-02
  
 
  
 10
 
 114135847
 
 114136073
 
  PDF   STATS   ENSEMBL   UCSC 
 
  227
 
  6
 
 4762
 
 gene
 
 0.157526
 
 0.7940793
 
 ACSL5 (margin: ACSL5)
 
  4.6518538
 
 2.16e-03
 
 1.45e-02
  
 
  
 6
 
 150463775
 
 150464265
 
  PDF   STATS   ENSEMBL   UCSC 
 
  491
 
  8
 
 36568
 
 island
 
 0.040524
 
 0.5505686
 
 PPP1R14C (margin: PPP1R14C)
 
  4.6473092
 
 1.86e-03
 
 1.45e-02
  
 
  
 10
 
 123357993
 
 123358317
 
  PDF   STATS   ENSEMBL   UCSC 
 
  325
 
  6
 
 5406
 
 tss;island
 
 0.043592
 
 0.4863748
 
 FGFR2 (margin: FGFR2)
 
  4.6434262
 
 2.16e-03
 
 1.45e-02
  
 
  
 7
 
  93519855
 
  93520036
 
  PDF   STATS   ENSEMBL   UCSC 
 
  182
 
  6
 
 37091
 
 gene
 
 0.787939
 
 0.2182080
 
 GNGT1; AC002076.10; TFPI2 (margin: GNGT1; AC002076.10; TFPI2)
 
  4.6206397
 
 2.16e-03
 
 1.45e-02
  
 
  
 12
 
 130646022
 
 130646497
 
  PDF   STATS   ENSEMBL   UCSC 
 
  476
 
  6
 
 10118
 
 tss;island
 
 0.108050
 
 0.5945637
 
 FZD10; FZD10-AS1 (margin: FZD10; FZD10-AS1)
 
  4.5802397
 
 2.16e-03
 
 1.45e-02
  
 
  
 12
 
  24715478
 
  24715564
 
  PDF   STATS   ENSEMBL   UCSC 
 
   87
 
  6
 
 9605
 
 tss;island
 
 0.529605
 
 0.0418054
 
 RP11-444D3.1 (margin: RP11-444D3.1; LINC00477)
 
  4.5783086
 
 2.16e-03
 
 1.45e-02
  
 
  
 4
 
    619162
 
    619392
 
  PDF   STATS   ENSEMBL   UCSC 
 
  231
 
  6
 
 30723
 
 island
 
 0.257760
 
 0.8742244
 
 PDE6B (margin: PDE6B)
 
  4.5706219
 
 2.16e-03
 
 1.45e-02
  
 
  
 10
 
  88126273
 
  88126306
 
  PDF   STATS   ENSEMBL   UCSC 
 
   34
 
  6
 
 5197
 
 tss
 
 0.562582
 
 0.0500006
 
 GRID1 (margin: GRID1)
 
  4.5656880
 
 2.16e-03
 
 1.45e-02
  
 
  
 17
 
  43662488
 
  43662979
 
  PDF   STATS   ENSEMBL   UCSC 
 
  492
 
  6
 
 18441
 
 island
 
 0.033586
 
 0.4112254
 
 DND1P1 (margin: DND1P1)
 
  4.5263290
 
 2.16e-03
 
 1.45e-02
  
 
  
 3
 
 100120235
 
 100120961
 
  PDF   STATS   ENSEMBL   UCSC 
 
  727
 
 11
 
 27878
 
 gene;tss;island
 
 0.111505
 
 0.0101826
 
 LNP1; TOMM70A (margin: LNP1; TOMM70A)
 
  4.4775811
 
 1.86e-03
 
 1.45e-02
  
 
  
 3
 
 145879430
 
 145879710
 
  PDF   STATS   ENSEMBL   UCSC 
 
  281
 
  6
 
 28647
 
 tss
 
 0.048617
 
 0.5412533
 
 PLOD2 (margin: PLOD2)
 
  4.4731987
 
 2.16e-03
 
 1.45e-02
  
 
  
 19
 
  18496756
 
  18497201
 
  PDF   STATS   ENSEMBL   UCSC 
 
  446
 
  8
 
 21778
 
 island
 
 0.017220
 
 0.1903055
 
 GDF15; MIR3189 (margin: GDF15; MIR3189; LRRC25)
 
  4.4654031
 
 1.86e-03
 
 1.45e-02
  
 
  
 8
 
  39695788
 
  39696062
 
  PDF   STATS   ENSEMBL   UCSC 
 
  275
 
  6
 
 39665
 
 tss
 
 0.646368
 
 0.9647476
 
 ADAM2 (margin: ADAM2)
 
  4.4555104
 
 2.16e-03
 
 1.45e-02
  
 
  
 5
 
   8457548
 
   8458089
 
  PDF   STATS   ENSEMBL   UCSC 
 
  542
 
  6
 
 32684
 
 island
 
 0.043443
 
 0.3485895
 
 MIR4458; RP11-480D4.1; RP11-480D4.2; RP11-480D4.4 (margin: MIR4458; RP11-480D4.1; RP11-480D4.2; RP11-480D4.6; RP11-480D4.4)
 
  4.4240203
 
 2.16e-03
 
 1.45e-02
  
 
  
 6
 
 112575705
 
 112576119
 
  PDF   STATS   ENSEMBL   UCSC 
 
  415
 
  6
 
 36423
 
 island
 
 0.482311
 
 0.9589687
 
 RP11-506B6.6; LAMA4 (margin: RP11-506B6.6; LAMA4)
 
  4.4023594
 
 2.16e-03
 
 1.45e-02
  
 
  
 10
 
  31609882
 
  31609960
 
  PDF   STATS   ENSEMBL   UCSC 
 
   79
 
  6
 
 5018
 
 tss
 
 0.082845
 
 0.6582600
 
 ZEB1; ZEB1-AS1 (margin: ZEB1; ZEB1-AS1)
 
  4.3979545
 
 2.16e-03
 
 1.45e-02
  
 
  
 17
 
  75446431
 
  75446661
 
  PDF   STATS   ENSEMBL   UCSC 
 
  231
 
  6
 
 16808
 
 gene;island
 
 0.156153
 
 0.8014304
 
 SEPT9 (margin: SEPT9; Y_RNA)
 
  4.3976001
 
 2.16e-03
 
 1.45e-02
  
 
  
 11
 
 114069864
 
 114070210
 
  PDF   STATS   ENSEMBL   UCSC 
 
  347
 
  6
 
 6911
 
 gene;island
 
 0.589264
 
 0.9720917
 
 ZBTB16 (margin: ZBTB16; RP11-64D24.2)
 
  4.3695832
 
 2.16e-03
 
 1.45e-02
  
 
  
 5
 
  80529067
 
  80529340
 
  PDF   STATS   ENSEMBL   UCSC 
 
  274
 
  6
 
 31666
 
 gene
 
 0.974666
 
 0.6636922
 
 CKMT2; CTC-281B15.1 (margin: RASGRF2; CKMT2; CTC-281B15.1; CTD-2248H3.1)
 
  4.3425455
 
 2.16e-03
 
 1.45e-02
  
 
  
 12
 
   6420020
 
   6420052
 
  PDF   STATS   ENSEMBL   UCSC 
 
   33
 
  6
 
 9023
 
 gene;tss;island
 
 0.938658
 
 0.4353919
 
 PLEKHG6 (margin: PLEKHG6)
 
  4.3416400
 
 2.16e-03
 
 1.45e-02
  
 
  
 12
 
  15475116
 
  15475342
 
  PDF   STATS   ENSEMBL   UCSC 
 
  227
 
  6
 
 9591
 
 tss
 
 0.200676
 
 0.8044111
 
 PTPRO; RERG (margin: PTPRO; RERG)
 
  4.3398406
 
 2.16e-03
 
 1.45e-02
  
 
  
 5
 
   1445354
 
   1445593
 
  PDF   STATS   ENSEMBL   UCSC 
 
  240
 
  6
 
 32629
 
 island
 
 0.442529
 
 0.9668690
 
 SLC6A3 (margin: SLC6A3; LPCAT1)
 
  4.3086010
 
 2.16e-03
 
 1.45e-02
  
 
  
 14
 
 101347044
 
 101347358
 
  PDF   STATS   ENSEMBL   UCSC 
 
  315
 
  6
 
 12210
 
 gene;tss;island
 
 0.161891
 
 0.8602137
 
 MIR431; MIR433; MIR127; RTL1 (margin: MIR493; MIR337; MIR665; MIR431; MIR433; MIR127; MIR432; MIR136; AL117190.3; RTL1)
 
  4.2899372
 
 2.16e-03
 
 1.45e-02
  
 
  
 4
 
  93224895
 
  93225565
 
  PDF   STATS   ENSEMBL   UCSC 
 
  671
 
  8
 
 31183
 
 island
 
 0.493817
 
 0.0476417
 
 GRID2; RP11-9B6.1 (margin: GRID2; RP11-9B6.1)
 
  4.2834746
 
 1.86e-03
 
 1.45e-02
  
 
  
 3
 
 112931126
 
 112931496
 
  PDF   STATS   ENSEMBL   UCSC 
 
  371
 
  8
 
 29363
 
 island
 
 0.410678
 
 0.0420926
 
 BOC (margin: BOC)
 
  4.2798912
 
 1.86e-03
 
 1.45e-02
  
 
  
 10
 
  23216637
 
  23216847
 
  PDF   STATS   ENSEMBL   UCSC 
 
  211
 
  6
 
 4986
 
 tss
 
 0.647070
 
 0.1066417
 
 ARMC3 (margin: ARMC3)
 
  4.2759778
 
 2.16e-03
 
 1.45e-02
  
 
  
 20
 
  61151391
 
  61151480
 
  PDF   STATS   ENSEMBL   UCSC 
 
   90
 
  6
 
 25490
 
 gene;tss;island
 
 0.116732
 
 0.7281271
 
 C20orf166; MIR1-1 (margin: C20orf166; MIR1-1; MIR133A2; C20orf166-AS1)
 
  4.2694486
 
 2.16e-03
 
 1.45e-02
  
 
  
 4
 
  40859249
 
  40859344
 
  PDF   STATS   ENSEMBL   UCSC 
 
   96
 
  6
 
 30361
 
 tss
 
 0.967730
 
 0.6059840
 
 APBB2 (margin: APBB2; snoU13)
 
  4.2611206
 
 2.16e-03
 
 1.45e-02
  
 
  
 1
 
  43533796
 
  43534324
 
  PDF   STATS   ENSEMBL   UCSC 
 
  529
 
  6
 
 3157
 
 island
 
 0.955390
 
 0.5345781
 
  (margin: )
 
  4.2542715
 
 2.16e-03
 
 1.45e-02
  
 
  
 8
 
  25902284
 
  25902611
 
  PDF   STATS   ENSEMBL   UCSC 
 
  328
 
  6
 
 40170
 
 island
 
 0.511699
 
 0.0870655
 
 EBF2 (margin: EBF2)
 
  4.2238394
 
 2.16e-03
 
 1.45e-02
  
 
  
 1
 
 201857840
 
 201857955
 
  PDF   STATS   ENSEMBL   UCSC 
 
  116
 
  6
 
 942
 
 gene
 
 0.437680
 
 0.0440046
 
 SHISA4 (margin: IPO9; SHISA4; RP11-307B6.3; LMOD1)
 
  4.2091723
 
 2.16e-03
 
 1.45e-02
  
 
  
 10
 
  28034792
 
  28034856
 
  PDF   STATS   ENSEMBL   UCSC 
 
   65
 
  6
 
 5004
 
 tss
 
 0.720930
 
 0.9752192
 
 RP11-360I20.2; MKX (margin: RP11-360I20.2; MKX)
 
  4.1178726
 
 2.16e-03
 
 1.45e-02
  
 
  
 19
 
  18548845
 
  18549274
 
  PDF   STATS   ENSEMBL   UCSC 
 
  430
 
  8
 
 21779
 
 island
 
 0.461350
 
 0.0449945
 
 ISYNA1 (margin: SSBP4; CTD-3137H5.1; ISYNA1; ELL; AC010335.1)
 
  4.1139997
 
 1.86e-03
 
 1.45e-02
  
 
  
 7
 
 145813413
 
 145813439
 
  PDF   STATS   ENSEMBL   UCSC 
 
   27
 
  6
 
 37959
 
 tss
 
 0.946067
 
 0.5182275
 
 CNTNAP2 (margin: CNTNAP2)
 
  4.1122155
 
 2.16e-03
 
 1.45e-02
  
 
  
 20
 
  33146263
 
  33146543
 
  PDF   STATS   ENSEMBL   UCSC 
 
  281
 
  6
 
 25400
 
 gene;island
 
 0.714251
 
 0.9752206
 
 MAP1LC3A; PIGU (margin: MAP1LC3A; PIGU)
 
  4.1058226
 
 2.16e-03
 
 1.45e-02
  
 
  
 1
 
  27113508
 
  27113753
 
  PDF   STATS   ENSEMBL   UCSC 
 
  246
 
  6
 
 1396
 
 tss;island
 
 0.025221
 
 0.3191601
 
 PIGV (margin: ARID1A; PIGV)
 
  4.1048016
 
 2.16e-03
 
 1.45e-02
  
 
  
 13
 
 112760898
 
 112761328
 
  PDF   STATS   ENSEMBL   UCSC 
 
  431
 
  6
 
 11916
 
 island
 
 0.566720
 
 0.9695521
 
 LINC00403; LINC00404 (margin: LINC00403; LINC00404)
 
  4.1024757
 
 2.16e-03
 
 1.45e-02
  
 
  
 22
 
  39712956
 
  39713086
 
  PDF   STATS   ENSEMBL   UCSC 
 
  131
 
  6
 
 26876
 
 gene;tss;island
 
 0.063532
 
 0.5309069
 
 RPL3; SNORD83A; SNORD43 (margin: RPL3; SNORD83B; SNORD83A; SNORD43)
 
  4.0765055
 
 2.16e-03
 
 1.45e-02
  
 
  
 1
 
  23504146
 
  23504632
 
  PDF   STATS   ENSEMBL   UCSC 
 
  487
 
  6
 
 2830
 
 island
 
 0.086265
 
 0.4786039
 
 LUZP1 (margin: LUZP1)
 
  4.0703481
 
 2.16e-03
 
 1.45e-02
  
 
  
 2
 
  37571677
 
  37571901
 
  PDF   STATS   ENSEMBL   UCSC 
 
  225
 
  6
 
 24223
 
 island
 
 0.973969
 
 0.6532795
 
 QPCT (margin: QPCT)
 
  4.0621579
 
 2.16e-03
 
 1.45e-02
  
 
  
 1
 
   1935048
 
   1935561
 
  PDF   STATS   ENSEMBL   UCSC 
 
  514
 
  6
 
 2467
 
 island
 
 0.521313
 
 0.9572980
 
 C1orf222 (margin: C1orf222; RP11-547D24.1)
 
  4.0238980
 
 2.16e-03
 
 1.45e-02
  
 
  
 7
 
  30029717
 
  30029808
 
  PDF   STATS   ENSEMBL   UCSC 
 
   92
 
  6
 
 36952
 
 gene
 
 0.080215
 
 0.5068099
 
 AC007285.6; SCRN1 (margin: AC007285.6; SCRN1)
 
  4.0236055
 
 2.16e-03
 
 1.45e-02
  
 
  
 11
 
  93583672
 
  93583973
 
  PDF   STATS   ENSEMBL   UCSC 
 
  302
 
  6
 
 7626
 
 tss
 
 0.042885
 
 0.4186877
 
 VSTM5 (margin: VSTM5)
 
  4.0065392
 
 2.16e-03
 
 1.45e-02
  
 
  
 6
 
  33171765
 
  33172572
 
  PDF   STATS   ENSEMBL   UCSC 
 
  808
 
 16
 
 35978
 
 island
 
 0.042213
 
 0.4272579
 
 SLC39A7; HSD17B8 (margin: RNY4P10; SLC39A7; HSD17B8; MIR219-1; RING1; ZNF70P1; COL11A2; RXRB)
 
  4.0064658
 
 1.94e-03
 
 1.45e-02
  
 
  
 8
 
 140716495
 
 140716766
 
  PDF   STATS   ENSEMBL   UCSC 
 
  272
 
  6
 
 39924
 
 tss;island
 
 0.319875
 
 0.0391380
 
 KCNK9 (margin: KCNK9)
 
  4.0059654
 
 2.16e-03
 
 1.45e-02
  
 
  
 7
 
   4264980
 
   4265548
 
  PDF   STATS   ENSEMBL   UCSC 
 
  569
 
  6
 
 36836
 
 gene
 
 0.433500
 
 0.9502536
 
 SDK1 (margin: SDK1)
 
  3.9956122
 
 2.16e-03
 
 1.45e-02
  
 
  
 17
 
   7348316
 
   7348399
 
  PDF   STATS   ENSEMBL   UCSC 
 
   84
 
  6
 
 17084
 
 tss
 
 0.957797
 
 0.6197443
 
 RP11-104H15.7; FGF11; CHRNB1; RP11-104H15.8 (margin: RP11-104H15.7; TMEM102; FGF11; CHRNB1; RP11-104H15.9; RP11-104H15.8)
 
  3.9825720
 
 2.16e-03
 
 1.45e-02
  
 
  
 17
 
  65040527
 
  65040616
 
  PDF   STATS   ENSEMBL   UCSC 
 
   90
 
  6
 
 17596
 
 tss
 
 0.595661
 
 0.9723212
 
 CACNG1 (margin: CACNG4; CACNG1; RP11-74H8.1)
 
  3.9621324
 
 2.16e-03
 
 1.45e-02
  
 
  
 16
 
  67184918
 
  67185194
 
  PDF   STATS   ENSEMBL   UCSC 
 
  277
 
  6
 
 15292
 
 tss;island
 
 0.672650
 
 0.9477831
 
 B3GNT9 (margin: C16orf70; FBXL8; B3GNT9; TRADD)
 
  3.9075948
 
 2.16e-03
 
 1.45e-02
  
 
  
 3
 
 179754483
 
 179754761
 
  PDF   STATS   ENSEMBL   UCSC 
 
  279
 
  8
 
 29683
 
 island
 
 0.906395
 
 0.3876031
 
 PEX5L (margin: PEX5L)
 
  3.8422245
 
 1.86e-03
 
 1.45e-02
  
 
  
 11
 
  75379491
 
  75379624
 
  PDF   STATS   ENSEMBL   UCSC 
 
  134
 
  6
 
 7570
 
 tss
 
 0.551989
 
 0.9229103
 
 MAP6 (margin: MAP6)
 
  3.8243396
 
 2.16e-03
 
 1.45e-02
  
 
  
 1
 
 220101698
 
 220101962
 
  PDF   STATS   ENSEMBL   UCSC 
 
  265
 
  6
 
 1014
 
 gene
 
 0.656775
 
 0.9564318
 
 SLC30A10 (margin: SLC30A10)
 
  3.8130696
 
 2.16e-03
 
 1.45e-02
  
 
  
 17
 
  44896147
 
  44896223
 
  PDF   STATS   ENSEMBL   UCSC 
 
   77
 
  6
 
 17435
 
 tss
 
 0.267941
 
 0.0392367
 
 WNT3 (margin: WNT3)
 
  3.8114463
 
 2.16e-03
 
 1.45e-02
  
 
  
 1
 
   3568669
 
   3568722
 
  PDF   STATS   ENSEMBL   UCSC 
 
   54
 
  6
 
 1183
 
 tss;island
 
 0.356578
 
 0.0395754
 
 TP73; WRAP73 (margin: TP73; WRAP73; RP5-1092A11.5)
 
  3.8005680
 
 2.16e-03
 
 1.45e-02
  
 
  
 15
 
  78556834
 
  78557094
 
  PDF   STATS   ENSEMBL   UCSC 
 
  261
 
  6
 
 13437
 
 gene;tss;island
 
 0.079483
 
 0.5423108
 
 DNAJA4; RP11-762H8.3 (margin: DNAJA4; RP11-762H8.3)
 
  3.7888905
 
 2.16e-03
 
 1.45e-02
  
 
  
 8
 
 104310606
 
 104310886
 
  PDF   STATS   ENSEMBL   UCSC 
 
  281
 
  6
 
 39857
 
 tss
 
 0.004542
 
 0.0361681
 
 FZD6; RP11-318M2.2 (margin: FZD6; RP11-318M2.2)
 
  3.7682394
 
 2.16e-03
 
 1.45e-02
  
 
  
 22
 
  24180995
 
  24181270
 
  PDF   STATS   ENSEMBL   UCSC 
 
  276
 
  6
 
 27354
 
 island
 
 0.789792
 
 0.9832129
 
 DERL3 (margin: SMARCB1; DERL3)
 
  3.7662262
 
 2.16e-03
 
 1.45e-02
  
 
  
 20
 
  42875645
 
  42875779
 
  PDF   STATS   ENSEMBL   UCSC 
 
  135
 
  6
 
 25756
 
 tss
 
 0.375155
 
 0.8806327
 
 GDAP1L1 (margin: GDAP1L1)
 
  3.7318099
 
 2.16e-03
 
 1.45e-02
  
 
  
 10
 
  91295317
 
  91295421
 
  PDF   STATS   ENSEMBL   UCSC 
 
  105
 
  6
 
 5215
 
 tss
 
 0.832077
 
 0.9874646
 
 SLC16A12 (margin: SLC16A12)
 
  3.7256486
 
 2.16e-03
 
 1.45e-02
  
 
  
 7
 
  50132541
 
  50133212
 
  PDF   STATS   ENSEMBL   UCSC 
 
  672
 
 12
 
 38489
 
 island
 
 0.765775
 
 0.9676109
 
 ZPBP (margin: C7orf72; ZPBP)
 
  3.7181416
 
 1.83e-03
 
 1.45e-02
  
 
  
 10
 
 131761150
 
 131761386
 
  PDF   STATS   ENSEMBL   UCSC 
 
  237
 
  6
 
 4838
 
 gene;island
 
 0.679958
 
 0.9626847
 
 EBF3 (margin: EBF3)
 
  3.7091212
 
 2.16e-03
 
 1.45e-02
  
 
  
 17
 
  33700745
 
  33700817
 
  PDF   STATS   ENSEMBL   UCSC 
 
   73
 
  6
 
 17267
 
 tss;island
 
 0.107058
 
 0.5302478
 
 SLFN11 (margin: SLFN11; RP11-686D22.6)
 
  3.6951192
 
 2.16e-03
 
 1.45e-02
  
 
  
 13
 
 112721712
 
 112721789
 
  PDF   STATS   ENSEMBL   UCSC 
 
   78
 
  6
 
 11543
 
 tss
 
 0.634855
 
 0.9495515
 
 SOX1 (margin: SOX1)
 
  3.6847228
 
 2.16e-03
 
 1.45e-02
  
 
  
 12
 
 122356316
 
 122356852
 
  PDF   STATS   ENSEMBL   UCSC 
 
  537
 
  6
 
 10925
 
 island
 
 0.958229
 
 0.6379166
 
 PSMD9; RP11-87C12.2; WDR66 (margin: PSMD9; RP11-87C12.2; RNU7-170P; WDR66)
 
  3.6689537
 
 2.16e-03
 
 1.45e-02
  
 
  
 14
 
  77737495
 
  77737800
 
  PDF   STATS   ENSEMBL   UCSC 
 
  306
 
  6
 
 13032
 
 island
 
 0.667932
 
 0.9651554
 
 NGB (margin: TMEM63C; MIR1260A; NGB; POMT2)
 
  3.6379368
 
 2.16e-03
 
 1.45e-02
  
 
  
 15
 
  83240578
 
  83240791
 
  PDF   STATS   ENSEMBL   UCSC 
 
  214
 
  6
 
 13857
 
 tss
 
 0.495213
 
 0.8750092
 
 CPEB1 (margin: RP11-379H8.1; CPEB1)
 
  3.6346110
 
 2.16e-03
 
 1.45e-02
  
 
  
 1
 
   1266468
 
   1266781
 
  PDF   STATS   ENSEMBL   UCSC 
 
  314
 
  6
 
 2426
 
 island
 
 0.348044
 
 0.8075355
 
 TAS1R3 (margin: GLTPD1; TAS1R3; CPSF3L; DVL1)
 
  3.6207124
 
 2.16e-03
 
 1.45e-02
  
 
  
 2
 
 177053274
 
 177053297
 
  PDF   STATS   ENSEMBL   UCSC 
 
   24
 
  6
 
 23744
 
 tss
 
 0.074999
 
 0.4796419
 
 HOXD1; HOXD-AS1 (margin: AC009336.24; HOXD1; HOXD-AS1)
 
  3.5982128
 
 2.16e-03
 
 1.45e-02
  
 
  
 6
 
 100054469
 
 100054615
 
  PDF   STATS   ENSEMBL   UCSC 
 
  147
 
  6
 
 35137
 
 tss
 
 0.811298
 
 0.9854314
 
 PRDM13 (margin: PRDM13)
 
  3.5611818
 
 2.16e-03
 
 1.45e-02
  
 
  
 17
 
   7165831
 
   7165909
 
  PDF   STATS   ENSEMBL   UCSC 
 
   79
 
  6
 
 17069
 
 tss;island
 
 0.077430
 
 0.0091601
 
 RP1-4G17.5; CLDN7 (margin: ELP5; CTDNEP1; RP1-4G17.5; CLDN7; Y_RNA)
 
  3.5412391
 
 2.16e-03
 
 1.45e-02
  
 
  
 4
 
  42399699
 
  42399851
 
  PDF   STATS   ENSEMBL   UCSC 
 
  153
 
  6
 
 30367
 
 tss
 
 0.821382
 
 0.9837941
 
 SHISA3 (margin: SHISA3; RP11-63A11.1; ATP8A1)
 
  3.5192345
 
 2.16e-03
 
 1.45e-02
  
 
  
 12
 
   6419409
 
   6419750
 
  PDF   STATS   ENSEMBL   UCSC 
 
  342
 
  6
 
 9506
 
 tss;island
 
 0.946476
 
 0.5454705
 
 PLEKHG6 (margin: PLEKHG6)
 
  3.4879664
 
 2.16e-03
 
 1.45e-02
  
 
  
 6
 
 168389700
 
 168390347
 
  PDF   STATS   ENSEMBL   UCSC 
 
  648
 
  6
 
 36652
 
 island
 
 0.634189
 
 0.9559250
 
  (margin: KIF25; RP3-470B24.5; KIF25-AS1)
 
  3.4754920
 
 2.16e-03
 
 1.45e-02
  
 
  
 13
 
 100643858
 
 100644657
 
  PDF   STATS   ENSEMBL   UCSC 
 
  800
 
  8
 
 11850
 
 island
 
 0.841986
 
 0.9713373
 
  (margin: ZIC2; LINC00554)
 
  3.4705756
 
 1.86e-03
 
 1.45e-02
  
 
  
 6
 
  32188695
 
  32189126
 
  PDF   STATS   ENSEMBL   UCSC 
 
  432
 
  6
 
 34099
 
 gene
 
 0.205905
 
 0.7816745
 
 NOTCH4 (margin: NOTCH4)
 
  3.4531152
 
 2.16e-03
 
 1.45e-02
  
 
  
 20
 
  57428282
 
  57428473
 
  PDF   STATS   ENSEMBL   UCSC 
 
  192
 
  6
 
 25472
 
 gene;island
 
 0.697752
 
 0.9624504
 
 GNAS (margin: GNAS; GNAS-AS1; RP1-309F20.3)
 
  3.4448400
 
 2.16e-03
 
 1.45e-02
  
 
  
 11
 
  64512829
 
  64513289
 
  PDF   STATS   ENSEMBL   UCSC 
 
  461
 
  6
 
 7402
 
 tss;island
 
 0.515863
 
 0.9118921
 
 RASGRP2; PYGM (margin: RASGRP2; PYGM)
 
  3.4385757
 
 2.16e-03
 
 1.45e-02
  
 
  
 15
 
  45406534
 
  45406939
 
  PDF   STATS   ENSEMBL   UCSC 
 
  406
 
  6
 
 13326
 
 gene
 
 0.413827
 
 0.8357450
 
 DUOXA2; DUOX2 (margin: DUOXA2; DUOX2; DUOXA1)
 
  3.3810891
 
 2.16e-03
 
 1.45e-02
  
 
  
 5
 
 169930841
 
 169930938
 
  PDF   STATS   ENSEMBL   UCSC 
 
   98
 
  6
 
 32484
 
 tss
 
 0.517026
 
 0.9473656
 
 KCNIP1 (margin: KCNIP1)
 
  3.3462379
 
 2.16e-03
 
 1.45e-02
  
 
  
 6
 
  84562778
 
  84563098
 
  PDF   STATS   ENSEMBL   UCSC 
 
  321
 
  9
 
 36292
 
 island
 
 0.657288
 
 0.9712561
 
 RIPPLY2 (margin: RIPPLY2; CYB5R4)
 
  3.3268848
 
 1.85e-03
 
 1.45e-02
  
 
  
 13
 
 113642457
 
 113642784
 
  PDF   STATS   ENSEMBL   UCSC 
 
  328
 
  6
 
 11265
 
 gene
 
 0.747506
 
 0.9700272
 
 MCF2L (margin: MCF2L)
 
  3.2744564
 
 2.16e-03
 
 1.45e-02
  
 
  
 4
 
  41753439
 
  41754125
 
  PDF   STATS   ENSEMBL   UCSC 
 
  687
 
  8
 
 31004
 
 island
 
 0.503217
 
 0.8273458
 
 RP11-227F19.1; RP11-227F19.2 (margin: RP11-227F19.1; RP11-227F19.2; PHOX2B)
 
  3.2237912
 
 1.86e-03
 
 1.45e-02
  
 
  
 12
 
 132689907
 
 132690782
 
  PDF   STATS   ENSEMBL   UCSC 
 
  876
 
 11
 
 9434
 
 gene;island
 
 0.101196
 
 0.7576034
 
 GALNT9 (margin: GALNT9)
 
  3.2215664
 
 1.86e-03
 
 1.45e-02
  
 
  
 5
 
 169930841
 
 169931363
 
  PDF   STATS   ENSEMBL   UCSC 
 
  523
 
  9
 
 31890
 
 gene;island
 
 0.583777
 
 0.9335790
 
 KCNIP1 (margin: KCNIP1)
 
  3.2076584
 
 1.85e-03
 
 1.45e-02
  
 
  
 1
 
 160039962
 
 160040667
 
  PDF   STATS   ENSEMBL   UCSC 
 
  706
 
 11
 
 2035
 
 tss
 
 0.362764
 
 0.8538831
 
 RP11-536C5.2; KCNJ10 (margin: RP11-536C5.2; KCNJ9; KCNJ10)
 
  3.1998096
 
 1.86e-03
 
 1.45e-02
  
 
  
 6
 
  32363875
 
  32364075
 
  PDF   STATS   ENSEMBL   UCSC 
 
  201
 
  6
 
 34106
 
 gene
 
 0.722917
 
 0.9672831
 
 BTNL2 (margin: HCG23; BTNL2)
 
  3.1563938
 
 2.16e-03
 
 1.45e-02
  
 
  
 12
 
  52626814
 
  52627047
 
  PDF   STATS   ENSEMBL   UCSC 
 
  234
 
  6
 
 10437
 
 island
 
 0.847509
 
 0.9820642
 
 KRT7 (margin: LINC00592; KRT7; METTL7AP1; RP3-416H24.1)
 
  3.1547038
 
 2.16e-03
 
 1.45e-02
  
 
  
 1
 
  13910138
 
  13910224
 
  PDF   STATS   ENSEMBL   UCSC 
 
   87
 
  6
 
 1261
 
 tss;island
 
 0.410967
 
 0.8581603
 
 PDPN (margin: PDPN)
 
  3.0982414
 
 2.16e-03
 
 1.45e-02
  
 
  
 10
 
   8094431
 
   8094860
 
  PDF   STATS   ENSEMBL   UCSC 
 
  430
 
  6
 
 4487
 
 gene;island
 
 0.928300
 
 0.5969752
 
 RP11-379F12.4; GATA3; GATA3-AS1; RP11-379F12.3 (margin: RP11-379F12.4; GATA3; GATA3-AS1; RP11-379F12.3)
 
  3.0975656
 
 2.16e-03
 
 1.45e-02
  
 
  
 12
 
 114841536
 
 114842031
 
  PDF   STATS   ENSEMBL   UCSC 
 
  496
 
  8
 
 9325
 
 gene;island
 
 0.706190
 
 0.9787814
 
 TBX5 (margin: TBX5-AS1; TBX5)
 
  3.0296095
 
 1.86e-03
 
 1.45e-02
  
 
  
 5
 
 126365232
 
 126365876
 
  PDF   STATS   ENSEMBL   UCSC 
 
  645
 
  6
 
 31736
 
 gene;island
 
 0.109818
 
 0.0120948
 
 MARCH3 (margin: MARCH3)
 
  3.0144010
 
 2.16e-03
 
 1.45e-02
  
 
  
 1
 
  15271830
 
  15272326
 
  PDF   STATS   ENSEMBL   UCSC 
 
  497
 
  6
 
 241
 
 gene;tss
 
 0.548713
 
 0.9408368
 
 KAZN (margin: KAZN)
 
  2.9739468
 
 2.16e-03
 
 1.45e-02
  
 
  
 19
 
  54041163
 
  54041398
 
  PDF   STATS   ENSEMBL   UCSC 
 
  236
 
  6
 
 20206
 
 gene;island
 
 0.802056
 
 0.4296791
 
 ZNF331 (margin: ZNF331)
 
  2.9699222
 
 2.16e-03
 
 1.45e-02
  
 
  
 19
 
  21106002
 
  21106053
 
  PDF   STATS   ENSEMBL   UCSC 
 
   52
 
  6
 
 20657
 
 tss
 
 0.752598
 
 0.2853009
 
 ZNF85 (margin: ZNF85)
 
  2.9613138
 
 2.16e-03
 
 1.45e-02
  
 
  
 14
 
  74705942
 
  74706113
 
  PDF   STATS   ENSEMBL   UCSC 
 
  172
 
  6
 
 12505
 
 tss
 
 0.630797
 
 0.8620925
 
 VSX2 (margin: VSX2)
 
  2.9188836
 
 2.16e-03
 
 1.45e-02
  
 
  
 10
 
  49892741
 
  49893026
 
  PDF   STATS   ENSEMBL   UCSC 
 
  286
 
  8
 
 5068
 
 tss
 
 0.564010
 
 0.9218539
 
 WDFY4 (margin: WDFY4)
 
  2.9067916
 
 1.86e-03
 
 1.45e-02
  
 
  
 10
 
  27547536
 
  27547965
 
  PDF   STATS   ENSEMBL   UCSC 
 
  430
 
  6
 
 5628
 
 island
 
 0.799525
 
 0.9779250
 
 ARMC4P1; LRRC37A6P (margin: RP11-85G18.6; ARMC4P1; LRRC37A6P)
 
  2.8280895
 
 2.16e-03
 
 1.45e-02
  
 
  
 7
 
  92464881
 
  92465342
 
  PDF   STATS   ENSEMBL   UCSC 
 
  462
 
  8
 
 37089
 
 gene;island
 
 0.010133
 
 0.0713812
 
 AC002454.1; CDK6 (margin: AC002454.1; CDK6)
 
  2.8164717
 
 1.86e-03
 
 1.45e-02
  
 
  
 10
 
 126138593
 
 126138879
 
  PDF   STATS   ENSEMBL   UCSC 
 
  287
 
  6
 
 5422
 
 tss
 
 0.107762
 
 0.4634467
 
 RP13-238F13.3; NKX1-2 (margin: RP13-238F13.3; LHPP; RP13-238F13.5; NKX1-2)
 
  2.7167135
 
 2.16e-03
 
 1.45e-02
  
 
  
 14
 
  70655686
 
  70656116
 
  PDF   STATS   ENSEMBL   UCSC 
 
  431
 
  9
 
 12976
 
 island
 
 0.620561
 
 0.2073824
 
 RP11-486O13.2; SLC8A3 (margin: RP11-486O13.2; SLC8A3)
 
  2.6885986
 
 1.85e-03
 
 1.45e-02
  
 
  
 12
 
  57588031
 
  57588350
 
  PDF   STATS   ENSEMBL   UCSC 
 
  320
 
  8
 
 9187
 
 gene
 
 0.970962
 
 0.8060020
 
 LRP1; MIR1228 (margin: LRP1; MIR1228)
 
  2.6617524
 
 1.86e-03
 
 1.45e-02
  
 
  
 4
 
 154680808
 
 154681323
 
  PDF   STATS   ENSEMBL   UCSC 
 
  516
 
  6
 
 30155
 
 gene;island
 
 0.885930
 
 0.9755379
 
 RNF175 (margin: RNF175)
 
  2.6315581
 
 2.16e-03
 
 1.45e-02
  
 
  
 5
 
 178450457
 
 178450763
 
  PDF   STATS   ENSEMBL   UCSC 
 
  307
 
  6
 
 32547
 
 tss
 
 0.074159
 
 0.3059243
 
 ZNF879 (margin: ZNF879)
 
  2.6003212
 
 2.16e-03
 
 1.45e-02
  
 
  
 16
 
  56622951
 
  56623215
 
  PDF   STATS   ENSEMBL   UCSC 
 
  265
 
  6
 
 15246
 
 tss
 
 0.908559
 
 0.9839839
 
 MT3 (margin: MT3)
 
  2.5962805
 
 2.16e-03
 
 1.45e-02
  
 
  
 14
 
  85996101
 
  85996499
 
  PDF   STATS   ENSEMBL   UCSC 
 
  399
 
 10
 
 13053
 
 island
 
 0.217010
 
 0.5801306
 
 RP11-497E19.2; FLRT2; RP11-497E19.1 (margin: RP11-497E19.2; FLRT2; RP11-497E19.1)
 
  2.5452943
 
 2.09e-03
 
 1.45e-02
  
 
  
 20
 
  61583537
 
  61584159
 
  PDF   STATS   ENSEMBL   UCSC 
 
  623
 
 11
 
 26338
 
 island
 
 0.749194
 
 0.9565417
 
 SLC17A9 (margin: GID8; SLC17A9)
 
  2.5355101
 
 1.86e-03
 
 1.45e-02
  
 
  
 1
 
  31845923
 
  31846110
 
  PDF   STATS   ENSEMBL   UCSC 
 
  188
 
  6
 
 1445
 
 tss
 
 0.899896
 
 0.9738864
 
 FABP3 (margin: ZCCHC17; FABP3)
 
  2.4781542
 
 2.16e-03
 
 1.45e-02
  
 
  
 3
 
 169530268
 
 169530621
 
  PDF   STATS   ENSEMBL   UCSC 
 
  354
 
  6
 
 29650
 
 island
 
 0.597743
 
 0.9117818
 
 LRRC34 (margin: LRRIQ4; LRRC34)
 
  2.4407134
 
 2.16e-03
 
 1.45e-02
  
 
  
 7
 
   4210949
 
   4211755
 
  PDF   STATS   ENSEMBL   UCSC 
 
  807
 
  8
 
 36831
 
 gene
 
 0.704586
 
 0.9191498
 
 SDK1 (margin: SDK1)
 
  2.3988220
 
 1.86e-03
 
 1.45e-02
  
 
  
 11
 
  61584319
 
  61584773
 
  PDF   STATS   ENSEMBL   UCSC 
 
  455
 
  8
 
 8335
 
 island
 
 0.507546
 
 0.1630558
 
 FADS2; FADS1; MIR1908 (margin: FADS2; FADS1; MIR1908)
 
  2.3704117
 
 1.86e-03
 
 1.45e-02
  
 
  
 2
 
 102091195
 
 102091321
 
  PDF   STATS   ENSEMBL   UCSC 
 
  127
 
  6
 
 23548
 
 tss
 
 0.767366
 
 0.9370724
 
 RFX8 (margin: AC092570.2; RFX8)
 
  2.3401958
 
 2.16e-03
 
 1.45e-02
  
 
  
 6
 
  33288180
 
  33289280
 
  PDF   STATS   ENSEMBL   UCSC 
 
 1101
 
 36
 
 34268
 
 gene;island
 
 0.786933
 
 0.9018939
 
 DAXX (margin: TAPBP; ZBTB22; DAXX)
 
  2.3265239
 
 1.64e-03
 
 1.45e-02
  
 
  
 1
 
 229569877
 
 229570288
 
  PDF   STATS   ENSEMBL   UCSC 
 
  412
 
 11
 
 2311
 
 tss
 
 0.768394
 
 0.9247575
 
 ACTA1 (margin: RP5-1068B5.1; ACTA1; RP5-1068B5.3; NUP133)
 
  2.3204149
 
 1.86e-03
 
 1.45e-02
  
 
  
 12
 
  39299501
 
  39299844
 
  PDF   STATS   ENSEMBL   UCSC 
 
  344
 
  6
 
 9636
 
 tss
 
 0.453800
 
 0.7601641
 
 RP11-396F22.1; CPNE8 (margin: RP11-396F22.1; CPNE8; AC067735.1)
 
  2.2939566
 
 2.16e-03
 
 1.45e-02
  
 
  
 6
 
 118228382
 
 118228650
 
  PDF   STATS   ENSEMBL   UCSC 
 
  269
 
  6
 
 35199
 
 tss;island
 
 0.691474
 
 0.3183170
 
 SLC35F1 (margin: SLC35F1)
 
  2.2770549
 
 2.16e-03
 
 1.45e-02
  
 
  
 1
 
 107682504
 
 107682963
 
  PDF   STATS   ENSEMBL   UCSC 
 
  460
 
  6
 
 3537
 
 island
 
 0.353124
 
 0.7190323
 
 NTNG1 (margin: NTNG1)
 
  2.2441181
 
 2.16e-03
 
 1.45e-02
  
 
  
 10
 
  18429643
 
  18429760
 
  PDF   STATS   ENSEMBL   UCSC 
 
  118
 
  6
 
 4520
 
 gene;island
 
 0.733489
 
 0.4243196
 
 CACNB2 (margin: CACNB2)
 
  2.2410824
 
 2.16e-03
 
 1.45e-02
  
 
  
 5
 
  37835506
 
  37836392
 
  PDF   STATS   ENSEMBL   UCSC 
 
  887
 
 11
 
 31591
 
 gene;tss;island
 
 0.931966
 
 0.9888281
 
 GDNF (margin: GDNF-AS1; GDNF)
 
  2.1864195
 
 1.86e-03
 
 1.45e-02
  
 
  
 6
 
  31628050
 
  31628440
 
  PDF   STATS   ENSEMBL   UCSC 
 
  391
 
 15
 
 33929
 
 gene
 
 0.002256
 
 0.0202209
 
 C6orf47-AS1; C6orf47; GPANK1 (margin: APOM; C6orf47-AS1; Y_RNA; CSNK2B; CSNK2B-LY6G5B-1181; LY6G5B; BAG6; C6orf47; GPANK1)
 
  2.1371489
 
 1.67e-03
 
 1.45e-02
  
 
  
 7
 
  23720292
 
  23720838
 
  PDF   STATS   ENSEMBL   UCSC 
 
  547
 
  6
 
 36920
 
 gene;island
 
 0.616283
 
 0.3098603
 
 FAM221A; AC006026.13 (margin: FAM221A; AC006026.1; AC006026.13)
 
  2.0873132
 
 2.16e-03
 
 1.45e-02
  
 
  
 4
 
  89618324
 
  89619573
 
  PDF   STATS   ENSEMBL   UCSC 
 
 1250
 
 20
 
 30055
 
 gene
 
 0.916552
 
 0.7277144
 
 HERC3; NAP1L5 (margin: HERC3; FAM13A-AS1; NAP1L5)
 
  2.0623712
 
 1.77e-03
 
 1.45e-02
  
 
  
 19
 
  58038573
 
  58038689
 
  PDF   STATS   ENSEMBL   UCSC 
 
  117
 
  6
 
 21118
 
 tss
 
 0.661445
 
 0.3560098
 
 ZNF549 (margin: ZNF773; ZNF549; ZNF550)
 
  2.0211679
 
 2.16e-03
 
 1.45e-02
  
 
  
 1
 
  46951291
 
  46951683
 
  PDF   STATS   ENSEMBL   UCSC 
 
  393
 
  6
 
 3219
 
 island
 
 0.936853
 
 0.9814498
 
  (margin: LINC00505)
 
  1.9850517
 
 2.16e-03
 
 1.45e-02
  
 
  
 16
 
  80966033
 
  80966491
 
  PDF   STATS   ENSEMBL   UCSC 
 
  459
 
  6
 
 16118
 
 island
 
 0.848212
 
 0.5832170
 
  (margin: )
 
  1.8566158
 
 2.16e-03
 
 1.45e-02
  
 
  
 13
 
 100641646
 
 100642106
 
  PDF   STATS   ENSEMBL   UCSC 
 
  461
 
  6
 
 11849
 
 island
 
 0.929146
 
 0.9777170
 
  (margin: ZIC2; LINC00554)
 
  1.8232425
 
 2.16e-03
 
 1.45e-02
  
 
  
 6
 
  32817662
 
  32818477
 
  PDF   STATS   ENSEMBL   UCSC 
 
  816
 
 12
 
 34154
 
 gene;island
 
 0.985170
 
 0.9487470
 
 PSMB9; TAP1 (margin: XXbac-BPG246D15.8; PSMB9; TAP2; PSMB8; TAP1)
 
  1.7954429
 
 1.83e-03
 
 1.45e-02
  
 
  
 11
 
  64360724
 
  64361020
 
  PDF   STATS   ENSEMBL   UCSC 
 
  297
 
  6
 
 6690
 
 gene
 
 0.573100
 
 0.8398903
 
 SLC22A12 (margin: SLC22A12)
 
  1.7810246
 
 2.16e-03
 
 1.45e-02
  
 
  
 10
 
 129845375
 
 129845766
 
  PDF   STATS   ENSEMBL   UCSC 
 
  392
 
  6
 
 5438
 
 tss
 
 0.088373
 
 0.2466843
 
 PTPRE (margin: PTPRE)
 
  1.7651991
 
 2.16e-03
 
 1.45e-02
  
 
  
 14
 
 101291068
 
 101292392
 
  PDF   STATS   ENSEMBL   UCSC 
 
 1325
 
 22
 
 12608
 
 tss;island
 
 0.817094
 
 0.9420187
 
 MEG3 (margin: MEG3; MIR2392; AL117190.2; RP11-123M6.2)
 
  1.7639504
 
 1.69e-03
 
 1.45e-02
  
 
  
 5
 
  88179867
 
  88180330
 
  PDF   STATS   ENSEMBL   UCSC 
 
  464
 
  6
 
 31683
 
 gene;tss;island
 
 0.654113
 
 0.3971121
 
 MEF2C-AS1; MEF2C (margin: MEF2C-AS1; MEF2C)
 
  1.6522974
 
 2.16e-03
 
 1.45e-02
  
 
  
 18
 
  76740088
 
  76740262
 
  PDF   STATS   ENSEMBL   UCSC 
 
  175
 
  6
 
 19332
 
 tss
 
 0.957786
 
 0.9878716
 
 SALL3; RP11-849I19.1 (margin: SALL3; RP11-849I19.1)
 
  1.6507524
 
 2.16e-03
 
 1.45e-02
  
 
  
 11
 
   2293552
 
   2293665
 
  PDF   STATS   ENSEMBL   UCSC 
 
  114
 
  6
 
 7065
 
 tss;island
 
 0.874190
 
 0.9524090
 
 ASCL2 (margin: ASCL2)
 
  1.6507261
 
 2.16e-03
 
 1.45e-02
  
 
  
 1
 
 158083159
 
 158083569
 
  PDF   STATS   ENSEMBL   UCSC 
 
  411
 
  6
 
 3876
 
 island
 
 0.319420
 
 0.6221044
 
  (margin: )
 
  1.6325772
 
 2.16e-03
 
 1.45e-02
  
 
  
 19
 
  58220295
 
  58220837
 
  PDF   STATS   ENSEMBL   UCSC 
 
  543
 
 10
 
 22480
 
 island
 
 0.951825
 
 0.9856940
 
 AC003006.7; ZNF154 (margin: AC003006.7; ZNF154; ZNF671)
 
  1.4979229
 
 2.09e-03
 
 1.45e-02
  
 
  
 14
 
 102027514
 
 102027797
 
  PDF   STATS   ENSEMBL   UCSC 
 
  284
 
  6
 
 12636
 
 tss;island
 
 0.686037
 
 0.4548857
 
 DIO3; DIO3OS (margin: DIO3; DIO3OS)
 
  1.4137093
 
 2.16e-03
 
 1.45e-02
  
 
  
 8
 
  38964784
 
  38965026
 
  PDF   STATS   ENSEMBL   UCSC 
 
  243
 
  7
 
 39662
 
 tss
 
 0.987257
 
 0.0845960
 
 ADAM32 (margin: ADAM9; ADAM32)
 
  9.1104405
 
 2.33e-03
 
 1.52e-02
  
 
  
 17
 
   7197878
 
   7198087
 
  PDF   STATS   ENSEMBL   UCSC 
 
  210
 
  7
 
 17072
 
 tss
 
 0.683375
 
 0.0041107
 
 YBX2 (margin: SLC2A4; RP1-4G17.2; YBX2)
 
  7.5565915
 
 2.33e-03
 
 1.52e-02
  
 
  
 2
 
 223916502
 
 223916952
 
  PDF   STATS   ENSEMBL   UCSC 
 
  451
 
  7
 
 25157
 
 island
 
 0.266617
 
 0.9817981
 
 KCNE4 (margin: KCNE4)
 
  6.5001592
 
 2.33e-03
 
 1.52e-02
  
 
  
 6
 
  36355343
 
  36355825
 
  PDF   STATS   ENSEMBL   UCSC 
 
  483
 
  7
 
 36081
 
 island
 
 0.129236
 
 0.9579471
 
 RP1-50J22.4; ETV7 (margin: RP1-50J22.4; ETV7; PXT1)
 
  6.1289668
 
 2.33e-03
 
 1.52e-02
  
 
  
 6
 
  39016262
 
  39016776
 
  PDF   STATS   ENSEMBL   UCSC 
 
  515
 
  7
 
 36105
 
 island
 
 0.471043
 
 0.9617406
 
 GLP1R (margin: GLP1R; RP1-202I21.3)
 
  6.0434446
 
 2.33e-03
 
 1.52e-02
  
 
  
 17
 
   6659164
 
   6659562
 
  PDF   STATS   ENSEMBL   UCSC 
 
  399
 
  7
 
 16377
 
 gene
 
 0.182400
 
 0.8932931
 
 XAF1 (margin: RPL23AP73; XAF1; CTC-281F24.2)
 
  6.0415748
 
 2.33e-03
 
 1.52e-02
  
 
  
 10
 
 134859687
 
 134860210
 
  PDF   STATS   ENSEMBL   UCSC 
 
  524
 
  7
 
 6329
 
 island
 
 0.312062
 
 0.8311340
 
  (margin: )
 
  5.6328969
 
 2.33e-03
 
 1.52e-02
  
 
  
 4
 
 176986621
 
 176986950
 
  PDF   STATS   ENSEMBL   UCSC 
 
  330
 
  7
 
 30687
 
 tss
 
 0.493372
 
 0.0102845
 
 WDR17 (margin: WDR17)
 
  5.1238094
 
 2.33e-03
 
 1.52e-02
  
 
  
 4
 
  93224895
 
  93225466
 
  PDF   STATS   ENSEMBL   UCSC 
 
  572
 
  7
 
 30499
 
 tss
 
 0.525546
 
 0.0256232
 
 GRID2; RP11-9B6.1 (margin: GRID2; RP11-9B6.1)
 
  5.0768300
 
 2.33e-03
 
 1.52e-02
  
 
  
 6
 
 167535764
 
 167536184
 
  PDF   STATS   ENSEMBL   UCSC 
 
  421
 
  7
 
 34556
 
 gene;tss
 
 0.522759
 
 0.9529102
 
 CCR6 (margin: CCR6)
 
  4.9735103
 
 2.33e-03
 
 1.52e-02
  
 
  
 18
 
  48086162
 
  48086680
 
  PDF   STATS   ENSEMBL   UCSC 
 
  519
 
  7
 
 19514
 
 island
 
 0.371146
 
 0.0293444
 
 MAPK4 (margin: MAPK4)
 
  4.9700773
 
 2.33e-03
 
 1.52e-02
  
 
  
 8
 
  97505593
 
  97505868
 
  PDF   STATS   ENSEMBL   UCSC 
 
  276
 
  7
 
 39827
 
 tss;island
 
 0.549832
 
 0.0323197
 
 SDC2 (margin: SDC2)
 
  4.8788651
 
 2.33e-03
 
 1.52e-02
  
 
  
 18
 
    580188
 
    580435
 
  PDF   STATS   ENSEMBL   UCSC 
 
  248
 
  7
 
 19347
 
 island
 
 0.480626
 
 0.9636747
 
 CETN1 (margin: CETN1; RN7SKP146)
 
  4.6895918
 
 2.33e-03
 
 1.52e-02
  
 
  
 17
 
  73874443
 
  73874790
 
  PDF   STATS   ENSEMBL   UCSC 
 
  348
 
  7
 
 18765
 
 island
 
 0.012213
 
 0.3893657
 
 RP11-552F3.9; TRIM47; TRIM65 (margin: RP11-552F3.9; TRIM47; TRIM65)
 
  4.6397732
 
 2.33e-03
 
 1.52e-02
  
 
  
 4
 
 156297854
 
 156298427
 
  PDF   STATS   ENSEMBL   UCSC 
 
  574
 
 12
 
 31390
 
 island
 
 0.736511
 
 0.0398933
 
 AC097467.2; MAP9 (margin: AC097467.2; RP11-27G13.5; MAP9; RP11-27G13.3; RP11-27G13.4)
 
  4.6366054
 
 2.32e-03
 
 1.52e-02
  
 
  
 4
 
  66536186
 
  66536772
 
  PDF   STATS   ENSEMBL   UCSC 
 
  587
 
  7
 
 30417
 
 tss;island
 
 0.780437
 
 0.2405901
 
 RP11-807H7.1; EPHA5 (margin: RP11-807H7.1; EPHA5)
 
  4.5166718
 
 2.33e-03
 
 1.52e-02
  
 
  
 19
 
  37341052
 
  37341261
 
  PDF   STATS   ENSEMBL   UCSC 
 
  210
 
  7
 
 20731
 
 tss
 
 0.436574
 
 0.0207818
 
 ZNF345; ZNF790 (margin: ZNF345; ZNF790; Y_RNA)
 
  4.4984089
 
 2.33e-03
 
 1.52e-02
  
 
  
 7
 
 130125200
 
 130126024
 
  PDF   STATS   ENSEMBL   UCSC 
 
  825
 
 12
 
 37900
 
 tss
 
 0.815743
 
 0.1020613
 
 MEST; RP11-2E11.5 (margin: MEST; MIR335; RP11-2E11.5; hsa-mir-335)
 
  4.3242942
 
 2.32e-03
 
 1.52e-02
  
 
  
 13
 
 108922032
 
 108922504
 
  PDF   STATS   ENSEMBL   UCSC 
 
  473
 
  7
 
 11224
 
 gene
 
 0.096754
 
 0.6564768
 
 TNFSF13B (margin: TNFSF13B)
 
  4.3052139
 
 2.33e-03
 
 1.52e-02
  
 
  
 2
 
  71205052
 
  71205685
 
  PDF   STATS   ENSEMBL   UCSC 
 
  634
 
  7
 
 24391
 
 island
 
 0.389157
 
 0.9202473
 
 ANKRD53; AC007040.11 (margin: ANKRD53; AC007040.11; TEX261)
 
  4.2994208
 
 2.33e-03
 
 1.52e-02
  
 
  
 3
 
 118753610
 
 118753871
 
  PDF   STATS   ENSEMBL   UCSC 
 
  262
 
  7
 
 27904
 
 gene;island
 
 0.154896
 
 0.0071733
 
 IGSF11 (margin: IGSF11)
 
  4.2253782
 
 2.33e-03
 
 1.52e-02
  
 
  
 11
 
  76381687
 
  76382149
 
  PDF   STATS   ENSEMBL   UCSC 
 
  463
 
  7
 
 7577
 
 tss;island
 
 0.584332
 
 0.9609839
 
 LRRC32 (margin: AP001189.4; RP11-672A2.3; LRRC32)
 
  4.1632308
 
 2.33e-03
 
 1.52e-02
  
 
  
 5
 
  87974369
 
  87974547
 
  PDF   STATS   ENSEMBL   UCSC 
 
  179
 
  7
 
 31680
 
 gene;tss;island
 
 0.816910
 
 0.9892670
 
 CTC-467M3.1; LINC00461 (margin: CTC-467M3.1; LINC00461)
 
  4.0051290
 
 2.33e-03
 
 1.52e-02
  
 
  
 7
 
  82792239
 
  82792411
 
  PDF   STATS   ENSEMBL   UCSC 
 
  173
 
  7
 
 37678
 
 tss
 
 0.146606
 
 0.0074790
 
 PCLO (margin: PCLO)
 
  3.9692367
 
 2.33e-03
 
 1.52e-02
  
 
  
 1
 
  44870976
 
  44871634
 
  PDF   STATS   ENSEMBL   UCSC 
 
  659
 
  7
 
 489
 
 gene
 
 0.266203
 
 0.0150929
 
 RNF220 (margin: RNF220)
 
  3.8815264
 
 2.33e-03
 
 1.52e-02
  
 
  
 7
 
  75157456
 
  75157765
 
  PDF   STATS   ENSEMBL   UCSC 
 
  310
 
  7
 
 37654
 
 tss
 
 0.007502
 
 0.0470124
 
 PMS2P3 (margin: PMS2P3; HIP1)
 
  3.8319285
 
 2.33e-03
 
 1.52e-02
  
 
  
 11
 
  69519128
 
  69519437
 
  PDF   STATS   ENSEMBL   UCSC 
 
  310
 
  7
 
 7521
 
 tss
 
 0.330090
 
 0.6914312
 
 FGF19 (margin: FGF19)
 
  3.6673354
 
 2.33e-03
 
 1.52e-02
  
 
  
 21
 
  37914924
 
  37915391
 
  PDF   STATS   ENSEMBL   UCSC 
 
  468
 
  7
 
 26418
 
 gene;tss
 
 0.646491
 
 0.9773228
 
 CLDN14 (margin: AP000695.4; CLDN14)
 
  3.5693596
 
 2.33e-03
 
 1.52e-02
  
 
  
 19
 
  46999224
 
  46999444
 
  PDF   STATS   ENSEMBL   UCSC 
 
  221
 
  7
 
 20893
 
 tss
 
 0.651436
 
 0.9585077
 
 AC011484.1; PPP5D1; PNMAL2 (margin: AC011484.1; PPP5D1; PNMAL2)
 
  3.2849627
 
 2.33e-03
 
 1.52e-02
  
 
  
 11
 
  32454718
 
  32455735
 
  PDF   STATS   ENSEMBL   UCSC 
 
 1018
 
 12
 
 6567
 
 gene;island
 
 0.566199
 
 0.9579996
 
 WT1-AS; WT1 (margin: WT1-AS; WT1)
 
  2.5748314
 
 2.32e-03
 
 1.52e-02
  
 
  
 12
 
  57588031
 
  57588243
 
  PDF   STATS   ENSEMBL   UCSC 
 
  213
 
  7
 
 9811
 
 tss
 
 0.972239
 
 0.8325115
 
 LRP1; MIR1228 (margin: LRP1; MIR1228)
 
  2.4559678
 
 2.33e-03
 
 1.52e-02
  
 
  
 2
 
 239139911
 
 239140296
 
  PDF   STATS   ENSEMBL   UCSC 
 
  386
 
  7
 
 23096
 
 gene
 
 0.937505
 
 0.9834787
 
 AC096574.4; AC016757.3 (margin: AC096574.4; AC016757.3; HES6)
 
  2.0064362
 
 2.33e-03
 
 1.52e-02
  
 
  
 18
 
  76740088
 
  76740284
 
  PDF   STATS   ENSEMBL   UCSC 
 
  197
 
  7
 
 19593
 
 island
 
 0.950974
 
 0.9872025
 
 SALL3; RP11-849I19.1 (margin: SALL3; RP11-849I19.1)
 
  1.6563731
 
 2.33e-03
 
 1.52e-02
  
 
  
 5
 
  52083512
 
  52084055
 
  PDF   STATS   ENSEMBL   UCSC 
 
  544
 
 13
 
 32058
 
 tss;island
 
 0.016522
 
 0.1147280
 
 ITGA1; PELO; CTD-2288O8.1 (margin: ITGA1; PELO; CTD-2288O8.1)
 
  3.0180679
 
 2.35e-03
 
 1.53e-02
  
 
  
 4
 
  46391414
 
  46391929
 
  PDF   STATS   ENSEMBL   UCSC 
 
  516
 
 11
 
 30372
 
 tss
 
 0.910914
 
 0.1509597
 
 RP11-436F23.1; GABRA2 (margin: RP11-436F23.1; GABRA2)
 
  4.8855929
 
 2.45e-03
 
 1.59e-02
  
 
  
 7
 
  75931606
 
  75932007
 
  PDF   STATS   ENSEMBL   UCSC 
 
  402
 
 11
 
 38597
 
 island
 
 0.163115
 
 0.0158432
 
 HSPB1 (margin: HSPB1)
 
  3.0693241
 
 2.45e-03
 
 1.59e-02
  
 
  
 4
 
 154073371
 
 154074387
 
  PDF   STATS   ENSEMBL   UCSC 
 
 1017
 
 15
 
 31370
 
 island
 
 0.422430
 
 0.0252470
 
 TRIM2 (margin: TRIM2)
 
  3.2619128
 
 2.70e-03
 
 1.76e-02
  
 
  
 2
 
 176994142
 
 176994764
 
  PDF   STATS   ENSEMBL   UCSC 
 
  623
 
  9
 
 24905
 
 island
 
 0.010562
 
 0.9328017
 
 HOXD8; HOXD-AS2 (margin: HOXD10; HOXD9; HOXD8; HOXD3; HOXD-AS2)
 
  9.5884667
 
 2.76e-03
 
 1.78e-02
  
 
  
 4
 
 187476543
 
 187477065
 
  PDF   STATS   ENSEMBL   UCSC 
 
  523
 
  9
 
 30707
 
 tss
 
 0.810564
 
 0.0103418
 
 RP11-215A19.2; MTNR1A (margin: RP11-215A19.2; MTNR1A)
 
  8.4808711
 
 2.76e-03
 
 1.78e-02
  
 
  
 1
 
 179557438
 
 179558114
 
  PDF   STATS   ENSEMBL   UCSC 
 
  677
 
  9
 
 3999
 
 island
 
 0.099654
 
 0.9318891
 
 RP11-545A16.3; RP11-545A16.4 (margin: RNU5F-2P; RP11-545A16.3; RP11-545A16.4; TDRD5)
 
  8.3135200
 
 2.76e-03
 
 1.78e-02
  
 
  
 19
 
  19281140
 
  19281559
 
  PDF   STATS   ENSEMBL   UCSC 
 
  420
 
  9
 
 20636
 
 tss
 
 0.518657
 
 0.0059051
 
 MEF2BNB-MEF2B; MEF2B (margin: MEF2BNB-MEF2B; MEF2B; MEF2BNB)
 
  7.6092661
 
 2.76e-03
 
 1.78e-02
  
 
  
 2
 
 178937477
 
 178937882
 
  PDF   STATS   ENSEMBL   UCSC 
 
  406
 
  9
 
 22944
 
 gene;island
 
 0.262155
 
 0.0113589
 
 PDE11A (margin: PDE11A)
 
  6.1292187
 
 2.76e-03
 
 1.78e-02
  
 
  
 6
 
  33095664
 
  33096501
 
  PDF   STATS   ENSEMBL   UCSC 
 
  838
 
  9
 
 34192
 
 gene
 
 0.021820
 
 0.5961358
 
 HLA-DPB2 (margin: HLA-DPB2; HLA-DPA3)
 
  5.9831777
 
 2.76e-03
 
 1.78e-02
  
 
  
 6
 
 151646540
 
 151647133
 
  PDF   STATS   ENSEMBL   UCSC 
 
  594
 
  9
 
 34509
 
 gene;island
 
 0.483203
 
 0.0208525
 
 AKAP12 (margin: AKAP12; RN7SKP268)
 
  5.7366611
 
 2.76e-03
 
 1.78e-02
  
 
  
 10
 
  61122307
 
  61122570
 
  PDF   STATS   ENSEMBL   UCSC 
 
  264
 
  9
 
 4596
 
 gene
 
 0.142931
 
 0.0061563
 
 FAM13C (margin: FAM13C)
 
  4.8040232
 
 2.76e-03
 
 1.78e-02
  
 
  
 16
 
  15595854
 
  15596423
 
  PDF   STATS   ENSEMBL   UCSC 
 
  570
 
  9
 
 14675
 
 gene
 
 0.395222
 
 0.9386682
 
 RP11-1021N1.1; C16orf45 (margin: RP11-1021N1.1; C16orf45)
 
  4.6045599
 
 2.76e-03
 
 1.78e-02
  
 
  
 1
 
  11919018
 
  11919699
 
  PDF   STATS   ENSEMBL   UCSC 
 
  682
 
  9
 
 1252
 
 tss
 
 0.126322
 
 0.0349865
 
 NPPB (margin: NPPA-AS1; NPPA; NPPB)
 
  4.0319373
 
 2.76e-03
 
 1.78e-02
  
 
  
 13
 
  84456127
 
  84456722
 
  PDF   STATS   ENSEMBL   UCSC 
 
  596
 
  9
 
 11806
 
 island
 
 0.894082
 
 0.2576876
 
 SLITRK1 (margin: SLITRK1)
 
  3.8203958
 
 2.76e-03
 
 1.78e-02
  
 
  
 4
 
  11430353
 
  11430908
 
  PDF   STATS   ENSEMBL   UCSC 
 
  556
 
  9
 
 30928
 
 island
 
 0.010810
 
 0.0427209
 
 HS3ST1 (margin: HS3ST1)
 
  1.7947585
 
 2.76e-03
 
 1.78e-02
  
 
  
 16
 
  88717374
 
  88717989
 
  PDF   STATS   ENSEMBL   UCSC 
 
  616
 
 13
 
 16212
 
 island
 
 0.617213
 
 0.2393905
 
 CYBA; MVD (margin: IL17C; SNAI3-AS1; CYBA; MVD)
 
  1.7001647
 
 2.87e-03
 
 1.85e-02
  
 
  
 21
 
  34442160
 
  34442674
 
  PDF   STATS   ENSEMBL   UCSC 
 
  515
 
 10
 
 26643
 
 island
 
 0.625716
 
 0.9762216
 
 OLIG1; AP000282.2 (margin: LINC00945; OLIG1; AP000282.2)
 
  3.2421429
 
 2.88e-03
 
 1.86e-02
  
 
  
 20
 
  23402051
 
  23402404
 
  PDF   STATS   ENSEMBL   UCSC 
 
  354
 
 10
 
 26051
 
 island
 
 0.001629
 
 0.0047478
 
 NAPB (margin: NAPB)
 
  2.9509981
 
 2.88e-03
 
 1.86e-02
  
 
  
 2
 
 128422113
 
 128422717
 
  PDF   STATS   ENSEMBL   UCSC 
 
  605
 
 10
 
 22862
 
 gene;island
 
 0.863115
 
 0.9526668
 
 LIMS2 (margin: GPR17; LIMS2)
 
  1.8554757
 
 2.88e-03
 
 1.86e-02
  
 
  
 17
 
  33775295
 
  33776116
 
  PDF   STATS   ENSEMBL   UCSC 
 
  822
 
 12
 
 18214
 
 island
 
 0.648494
 
 0.9535070
 
 SLFN13 (margin: SLFN13; RP11-1094M14.7)
 
  2.0976968
 
 2.91e-03
 
 1.88e-02
  
 
  
 20
 
  48099146
 
  48099479
 
  PDF   STATS   ENSEMBL   UCSC 
 
  334
 
  8
 
 26234
 
 island
 
 0.909396
 
 0.0106026
 
 KCNB1 (margin: KCNB1)
 
 10.3271610
 
 2.95e-03
 
 1.88e-02
  
 
  
 4
 
  48484997
 
  48485328
 
  PDF   STATS   ENSEMBL   UCSC 
 
  332
 
  8
 
 30382
 
 tss;island
 
 0.206271
 
 0.9775453
 
 SLC10A4 (margin: SLC10A4; ZAR1)
 
  6.6563024
 
 2.95e-03
 
 1.88e-02
  
 
  
 1
 
  49242359
 
  49242934
 
  PDF   STATS   ENSEMBL   UCSC 
 
  576
 
  8
 
 520
 
 gene;island
 
 0.786971
 
 0.0532768
 
 AGBL4; BEND5 (margin: AGBL4; BEND5)
 
  5.7178456
 
 2.95e-03
 
 1.88e-02
  
 
  
 10
 
  99531645
 
  99531934
 
  PDF   STATS   ENSEMBL   UCSC 
 
  290
 
  8
 
 5996
 
 island
 
 0.223994
 
 0.9459047
 
 SFRP5 (margin: ZFYVE27; SFRP5)
 
  5.3944431
 
 2.95e-03
 
 1.88e-02
  
 
  
 6
 
 166876490
 
 166877038
 
  PDF   STATS   ENSEMBL   UCSC 
 
  549
 
  8
 
 34545
 
 gene
 
 0.434865
 
 0.9768698
 
 RPS6KA2; RPS6KA2-IT1 (margin: RPS6KA2; RPS6KA2-IT1)
 
  4.9347771
 
 2.95e-03
 
 1.88e-02
  
 
  
 5
 
   1268644
 
   1269309
 
  PDF   STATS   ENSEMBL   UCSC 
 
  666
 
  8
 
 31531
 
 gene;island
 
 0.401198
 
 0.9778647
 
 TERT (margin: TERT)
 
  4.8558529
 
 2.95e-03
 
 1.88e-02
  
 
  
 21
 
  42218932
 
  42219299
 
  PDF   STATS   ENSEMBL   UCSC 
 
  368
 
  8
 
 26686
 
 island
 
 0.591361
 
 0.9862668
 
 DSCAM (margin: DSCAM)
 
  4.6986543
 
 2.95e-03
 
 1.88e-02
  
 
  
 15
 
  81426347
 
  81426610
 
  PDF   STATS   ENSEMBL   UCSC 
 
  264
 
  8
 
 13852
 
 tss
 
 0.831292
 
 0.9812865
 
 C15orf26 (margin: C15orf26)
 
  4.0267666
 
 2.95e-03
 
 1.88e-02
  
 
  
 3
 
 196366191
 
 196366669
 
  PDF   STATS   ENSEMBL   UCSC 
 
  479
 
  8
 
 29810
 
 island
 
 0.638609
 
 0.9425163
 
 NRROS; PIGX (margin: NRROS; PIGX; AC023797.1)
 
  3.8447485
 
 2.95e-03
 
 1.88e-02
  
 
  
 7
 
  50132903
 
  50133212
 
  PDF   STATS   ENSEMBL   UCSC 
 
  310
 
  8
 
 37593
 
 tss
 
 0.765775
 
 0.9676109
 
 ZPBP (margin: C7orf72; ZPBP)
 
  3.7888323
 
 2.95e-03
 
 1.88e-02
  
 
  
 11
 
  67141628
 
  67142030
 
  PDF   STATS   ENSEMBL   UCSC 
 
  403
 
  8
 
 6749
 
 gene;tss;island
 
 0.006607
 
 0.0984167
 
 AP003419.11; CLCF1 (margin: RN7SKP239; AP003419.11; CLCF1)
 
  3.7873273
 
 2.95e-03
 
 1.88e-02
  
 
  
 11
 
  85522279
 
  85522637
 
  PDF   STATS   ENSEMBL   UCSC 
 
  359
 
  8
 
 7605
 
 tss
 
 0.520712
 
 0.0914915
 
 SYTL2 (margin: SYTL2)
 
  3.7823961
 
 2.95e-03
 
 1.88e-02
  
 
  
 5
 
  63461216
 
  63461654
 
  PDF   STATS   ENSEMBL   UCSC 
 
  439
 
  8
 
 32088
 
 tss
 
 0.665439
 
 0.0975523
 
 RNF180 (margin: RNF180)
 
  3.7194629
 
 2.95e-03
 
 1.88e-02
  
 
  
 8
 
  54164051
 
  54164442
 
  PDF   STATS   ENSEMBL   UCSC 
 
  392
 
  8
 
 40296
 
 island
 
 0.712925
 
 0.9717494
 
 OPRK1 (margin: RP11-162D9.3; OPRK1)
 
  3.6707932
 
 2.95e-03
 
 1.88e-02
  
 
  
 16
 
   1030383
 
   1030619
 
  PDF   STATS   ENSEMBL   UCSC 
 
  237
 
  8
 
 14986
 
 tss;island
 
 0.828697
 
 0.9847632
 
 AC009041.2; SOX8; LMF1; RP11-161M6.2 (margin: AC009041.2; SOX8; LMF1; RP11-161M6.2; RP11-161M6.3)
 
  3.0529980
 
 2.95e-03
 
 1.88e-02
  
 
  
 19
 
  19050971
 
  19051482
 
  PDF   STATS   ENSEMBL   UCSC 
 
  512
 
  8
 
 19944
 
 gene;tss;island
 
 0.013192
 
 0.0836710
 
 AC005932.1; HOMER3 (margin: DDX49; AC005932.1; AC002985.3; HOMER3)
 
  2.8717782
 
 2.95e-03
 
 1.88e-02
  
 
  
 20
 
  10199210
 
  10199536
 
  PDF   STATS   ENSEMBL   UCSC 
 
  327
 
  8
 
 25999
 
 island
 
 0.041065
 
 0.0058438
 
 SNAP25; SNAP25-AS1 (margin: SNAP25; SNAP25-AS1)
 
  2.7632788
 
 2.95e-03
 
 1.88e-02
  
 
  
 13
 
  36872048
 
  36872346
 
  PDF   STATS   ENSEMBL   UCSC 
 
  299
 
  8
 
 11393
 
 tss
 
 0.439499
 
 0.7312730
 
 SOHLH2; CCDC169-SOHLH2; CCDC169 (margin: SOHLH2; CCDC169-SOHLH2; CCDC169; SPG20)
 
  2.6847657
 
 2.95e-03
 
 1.88e-02
  
 
  
 3
 
  25469392
 
  25469720
 
  PDF   STATS   ENSEMBL   UCSC 
 
  329
 
  8
 
 28190
 
 tss
 
 0.155647
 
 0.5470668
 
 RARB (margin: RARB)
 
  2.6439199
 
 2.95e-03
 
 1.88e-02
  
 
  
 14
 
  85996101
 
  85996393
 
  PDF   STATS   ENSEMBL   UCSC 
 
  293
 
  8
 
 12542
 
 tss
 
 0.175518
 
 0.4877925
 
 RP11-497E19.2; FLRT2; RP11-497E19.1 (margin: RP11-497E19.2; FLRT2; RP11-497E19.1)
 
  2.3313965
 
 2.95e-03
 
 1.88e-02
  
 
  
 4
 
 154073371
 
 154074259
 
  PDF   STATS   ENSEMBL   UCSC 
 
  889
 
 14
 
 30639
 
 tss
 
 0.447257
 
 0.0234930
 
 TRIM2 (margin: TRIM2)
 
  3.2750373
 
 2.96e-03
 
 1.88e-02
  
 
  
 5
 
 178487123
 
 178488100
 
  PDF   STATS   ENSEMBL   UCSC 
 
  978
 
 14
 
 33479
 
 island
 
 0.116434
 
 0.5210139
 
 ZNF354C (margin: ZNF354C)
 
  3.0197812
 
 2.96e-03
 
 1.88e-02
  
 
  
 20
 
  32307885
 
  32308529
 
  PDF   STATS   ENSEMBL   UCSC 
 
  645
 
 11
 
 26102
 
 island
 
 0.513200
 
 0.0061858
 
 PXMP4 (margin: ZNF341; PXMP4; RP4-553F4.2)
 
  6.7610648
 
 3.18e-03
 
 2.01e-02
  
 
  
 10
 
 131265059
 
 131265435
 
  PDF   STATS   ENSEMBL   UCSC 
 
  377
 
 11
 
 5440
 
 tss
 
 0.495232
 
 0.0189894
 
 MGMT (margin: MGMT)
 
  4.7984818
 
 3.18e-03
 
 2.01e-02
  
 
  
 11
 
  69634240
 
  69634709
 
  PDF   STATS   ENSEMBL   UCSC 
 
  470
 
 11
 
 7523
 
 tss;island
 
 0.572511
 
 0.9450471
 
 FGF3 (margin: FGF3)
 
  3.1541279
 
 3.18e-03
 
 2.01e-02
  
 
  
 3
 
  50382952
 
  50383485
 
  PDF   STATS   ENSEMBL   UCSC 
 
  534
 
 11
 
 29154
 
 island
 
 0.315713
 
 0.5222781
 
 ZMYND10-AS1; ZMYND10; NPRL2 (margin: ZMYND10-AS1; CYB561D2; XXcos-LUCA11.5; RASSF1; ZMYND10; NPRL2; TMEM115)
 
  1.6104679
 
 3.18e-03
 
 2.01e-02
  
 
  
 7
 
 150754936
 
 150755981
 
  PDF   STATS   ENSEMBL   UCSC 
 
 1046
 
 23
 
 39022
 
 island
 
 0.013311
 
 0.0476503
 
 SLC4A2; CDK5 (margin: ABCB8; ASIC3; SLC4A2; CDK5)
 
  1.8199500
 
 3.35e-03
 
 2.12e-02
  
 
  
 1
 
 209979111
 
 209979779
 
  PDF   STATS   ENSEMBL   UCSC 
 
  669
 
 13
 
 4167
 
 island
 
 0.056728
 
 0.0093928
 
 IRF6 (margin: IRF6)
 
  2.6965141
 
 3.49e-03
 
 2.20e-02
  
 
  
 19
 
  52391078
 
  52391789
 
  PDF   STATS   ENSEMBL   UCSC 
 
  712
 
 13
 
 22344
 
 island
 
 0.968067
 
 0.8365985
 
 CTC-429C10.2; ZNF577; ZNF649 (margin: CTC-429C10.2; ZNF577; ZNF649)
 
  2.2314026
 
 3.49e-03
 
 2.20e-02
  
 
  
 7
 
  24796981
 
  24797884
 
  PDF   STATS   ENSEMBL   UCSC 
 
  904
 
 12
 
 38318
 
 island
 
 0.245013
 
 0.9652738
 
 DFNA5 (margin: DFNA5)
 
  6.8848042
 
 3.64e-03
 
 2.29e-02
  
 
  
 13
 
  36920660
 
  36921174
 
  PDF   STATS   ENSEMBL   UCSC 
 
  515
 
 12
 
 11394
 
 tss
 
 0.223460
 
 0.0051040
 
 SPG20OS; SPG20 (margin: SPG20OS; AL139377.1; SPG20)
 
  6.5227752
 
 3.64e-03
 
 2.29e-02
  
 
  
 19
 
  52511285
 
  52511900
 
  PDF   STATS   ENSEMBL   UCSC 
 
  616
 
 12
 
 22346
 
 island
 
 0.009672
 
 0.2901162
 
 ZNF615 (margin: ZNF615; ZNF614)
 
  4.8360989
 
 3.64e-03
 
 2.29e-02
  
 
  
 17
 
  41277694
 
  41279022
 
  PDF   STATS   ENSEMBL   UCSC 
 
 1329
 
 28
 
 16595
 
 gene
 
 0.009157
 
 0.1370682
 
 NBR2; BRCA1 (margin: NBR2; BRCA1)
 
  2.9216806
 
 3.69e-03
 
 2.33e-02
  
 
  
 15
 
  48470205
 
  48470973
 
  PDF   STATS   ENSEMBL   UCSC 
 
  769
 
 10
 
 14119
 
 island
 
 0.926747
 
 0.0256239
 
 MYEF2 (margin: MYEF2; RP11-605F22.2)
 
  5.6731736
 
 3.89e-03
 
 2.43e-02
  
 
  
 8
 
  22926478
 
  22926911
 
  PDF   STATS   ENSEMBL   UCSC 
 
  434
 
 10
 
 40144
 
 island
 
 0.010998
 
 0.3816369
 
 RP11-875O11.2; TNFRSF10B; RP11-875O11.3 (margin: RP11-875O11.2; TNFRSF10B; RP11-875O11.3)
 
  4.8232305
 
 3.89e-03
 
 2.43e-02
  
 
  
 6
 
  32942063
 
  32942808
 
  PDF   STATS   ENSEMBL   UCSC 
 
  746
 
 10
 
 34171
 
 gene;island
 
 0.004822
 
 0.0589707
 
 BRD2; XXbac-BPG181M17.6 (margin: BRD2; BRD2-IT1; HLA-DMA; XXbac-BPG181M17.6)
 
  3.8460356
 
 3.89e-03
 
 2.43e-02
  
 
  
 10
 
  50887472
 
  50887934
 
  PDF   STATS   ENSEMBL   UCSC 
 
  463
 
 10
 
 5738
 
 island
 
 0.497170
 
 0.9460125
 
 CHAT; C10orf53 (margin: CHAT; C10orf53)
 
  3.7147896
 
 3.89e-03
 
 2.43e-02
  
 
  
 14
 
 101489985
 
 101490253
 
  PDF   STATS   ENSEMBL   UCSC 
 
  269
 
 10
 
 12620
 
 tss
 
 0.434178
 
 0.8979051
 
 MIR379; MIR411; MIR299; MIR1197; MIR323A; MIR380 (margin: MIR379; MIR411; MIR299; MIR1197; MIR323A; MIR758; MIR329-1; MIR329-2; MIR494; MIR1193; MIR543; MIR495; MIR380)
 
  3.5976947
 
 3.89e-03
 
 2.43e-02
  
 
  
 17
 
  79099711
 
  79100287
 
  PDF   STATS   ENSEMBL   UCSC 
 
  577
 
 10
 
 16894
 
 gene;tss;island
 
 0.690632
 
 0.9779997
 
 MIR338; AATK; MIR657 (margin: BAIAP2; MIR338; RP11-149I9.2; AATK; MIR657; MIR1250)
 
  3.2378865
 
 3.89e-03
 
 2.43e-02
  
 
  
 15
 
  40762807
 
  40763522
 
  PDF   STATS   ENSEMBL   UCSC 
 
  716
 
 10
 
 14038
 
 island
 
 0.010622
 
 0.0533472
 
 CHST14 (margin: BAHD1; CHST14; RP11-64K12.8; RP11-64K12.1)
 
  2.6180935
 
 3.89e-03
 
 2.43e-02
  
 
  
 10
 
  15130387
 
  15131036
 
  PDF   STATS   ENSEMBL   UCSC 
 
  650
 
 10
 
 5567
 
 island
 
 0.140784
 
 0.0089637
 
 DCLRE1CP1; ACBD7 (margin: RPP38; DCLRE1CP1; ACBD7; GAPDHP45; C10orf111)
 
  2.2021735
 
 3.89e-03
 
 2.43e-02
  
 
  
 7
 
  50861418
 
  50862266
 
  PDF   STATS   ENSEMBL   UCSC 
 
  849
 
 20
 
 37598
 
 tss;island
 
 0.655393
 
 0.9298661
 
 GRB10 (margin: GRB10)
 
  1.7671723
 
 3.89e-03
 
 2.43e-02
  
 
  
 17
 
   6947032
 
   6947464
 
  PDF   STATS   ENSEMBL   UCSC 
 
  433
 
 10
 
 17918
 
 island
 
 0.842418
 
 0.9152133
 
 SLC16A11 (margin: SLC16A13; SLC16A11)
 
  1.7157799
 
 3.89e-03
 
 2.43e-02
  
 
  
 11
 
   2920052
 
   2920819
 
  PDF   STATS   ENSEMBL   UCSC 
 
  768
 
 16
 
 6469
 
 gene;tss;island
 
 0.394485
 
 0.7994970
 
 SLC22A18; SLC22A18AS (margin: SLC22A18; SLC22A18AS)
 
  2.5588350
 
 3.92e-03
 
 2.46e-02
  
 
  
 16
 
   4588826
 
   4589127
 
  PDF   STATS   ENSEMBL   UCSC 
 
  302
 
  7
 
 15071
 
 tss
 
 0.008563
 
 0.9779079
 
 CDIP1 (margin: CDIP1)
 
 10.6061293
 
 4.08e-03
 
 2.47e-02
  
 
  
 1
 
  50489240
 
  50489596
 
  PDF   STATS   ENSEMBL   UCSC 
 
  357
 
  7
 
 522
 
 gene
 
 0.891673
 
 0.0326185
 
 AGBL4 (margin: MTND2P29; AGBL4)
 
  7.6851519
 
 4.08e-03
 
 2.47e-02
  
 
  
 7
 
  43797839
 
  43798207
 
  PDF   STATS   ENSEMBL   UCSC 
 
  369
 
  7
 
 37558
 
 tss;island
 
 0.563564
 
 0.0083039
 
 BLVRA (margin: BLVRA; AC005189.6)
 
  7.5471577
 
 4.08e-03
 
 2.47e-02
  
 
  
 8
 
 121824330
 
 121824929
 
  PDF   STATS   ENSEMBL   UCSC 
 
  600
 
  9
 
 39891
 
 tss
 
 0.726566
 
 0.0076390
 
 RP11-713M15.2; SNTB1 (margin: RP11-713M15.2; SNTB1)
 
  7.3424354
 
 3.99e-03
 
 2.47e-02
  
 
  
 7
 
  98099806
 
  98100419
 
  PDF   STATS   ENSEMBL   UCSC 
 
  614
 
  7
 
 38713
 
 island
 
 0.885376
 
 0.0317237
 
  (margin: AC074121.4)
 
  7.1964904
 
 4.08e-03
 
 2.47e-02
  
 
  
 1
 
 121260494
 
 121261146
 
  PDF   STATS   ENSEMBL   UCSC 
 
  653
 
  7
 
 3668
 
 island
 
 0.028271
 
 0.7329921
 
 EMBP1 (margin: EMBP1; RP11-344P13.4)
 
  7.1929154
 
 4.08e-03
 
 2.47e-02
  
 
  
 10
 
  86001241
 
  86001560
 
  PDF   STATS   ENSEMBL   UCSC 
 
  320
 
  7
 
 5194
 
 tss
 
 0.024339
 
 0.9167049
 
 LRIT1 (margin: RGR; LRIT1)
 
  6.8856604
 
 4.08e-03
 
 2.47e-02
  
 
  
 14
 
  79744991
 
  79745592
 
  PDF   STATS   ENSEMBL   UCSC 
 
  602
 
  7
 
 12159
 
 gene;tss;island
 
 0.602879
 
 0.0127651
 
 NRXN3 (margin: NRXN3)
 
  6.5670309
 
 4.08e-03
 
 2.47e-02
  
 
  
 6
 
  39290342
 
  39290877
 
  PDF   STATS   ENSEMBL   UCSC 
 
  536
 
  7
 
 34956
 
 tss
 
 0.026917
 
 0.7331221
 
 KCNK16 (margin: KCNK17; KCNK16; KIF6)
 
  5.8923858
 
 4.08e-03
 
 2.47e-02
  
 
  
 4
 
 141347993
 
 141348737
 
  PDF   STATS   ENSEMBL   UCSC 
 
  745
 
  9
 
 30138
 
 gene
 
 0.334080
 
 0.0067713
 
 CLGN (margin: CLGN)
 
  5.8880081
 
 3.99e-03
 
 2.47e-02
  
 
  
 11
 
  65405268
 
  65405525
 
  PDF   STATS   ENSEMBL   UCSC 
 
  258
 
  9
 
 7437
 
 tss
 
 0.016034
 
 0.5212912
 
 PCNXL3; MIR4690; SIPA1 (margin: PCNXL3; MIR4690; SIPA1; MIR4489)
 
  5.8158714
 
 3.99e-03
 
 2.47e-02
  
 
  
 16
 
  67312928
 
  67313043
 
  PDF   STATS   ENSEMBL   UCSC 
 
  116
 
  7
 
 14797
 
 gene;tss;island
 
 0.372309
 
 0.0342004
 
 PLEKHG4 (margin: SLC9A5; PLEKHG4; KCTD19)
 
  5.3769636
 
 4.08e-03
 
 2.47e-02
  
 
  
 4
 
  52917271
 
  52917567
 
  PDF   STATS   ENSEMBL   UCSC 
 
  297
 
  9
 
 30390
 
 tss
 
 0.017628
 
 0.3523604
 
 SPATA18 (margin: RP11-535C7.1; SPATA18)
 
  4.9718284
 
 3.99e-03
 
 2.47e-02
  
 
  
 17
 
  47307954
 
  47308460
 
  PDF   STATS   ENSEMBL   UCSC 
 
  507
 
  7
 
 18521
 
 island
 
 0.287656
 
 0.0074783
 
 PHOSPHO1 (margin: ABI3; PHOSPHO1)
 
  4.8097747
 
 4.08e-03
 
 2.47e-02
  
 
  
 13
 
  36944433
 
  36944649
 
  PDF   STATS   ENSEMBL   UCSC 
 
  217
 
  7
 
 11395
 
 tss
 
 0.734431
 
 0.9849033
 
 SPG20OS; SPG20 (margin: SPG20OS; SPG20)
 
  4.5730145
 
 4.08e-03
 
 2.47e-02
  
 
  
 17
 
  71640181
 
  71640369
 
  PDF   STATS   ENSEMBL   UCSC 
 
  189
 
  7
 
 18706
 
 island
 
 0.408269
 
 0.0240025
 
 RP11-277J6.2; SDK2 (margin: RP11-277J6.2; SDK2)
 
  4.4634114
 
 4.08e-03
 
 2.47e-02
  
 
  
 7
 
 100493365
 
 100493849
 
  PDF   STATS   ENSEMBL   UCSC 
 
  485
 
  7
 
 38785
 
 island
 
 0.216535
 
 0.0087562
 
 ACHE (margin: SRRT; UFSP1; ACHE; RN7SL549P)
 
  4.3616362
 
 4.08e-03
 
 2.47e-02
  
 
  
 1
 
  45082704
 
  45083278
 
  PDF   STATS   ENSEMBL   UCSC 
 
  575
 
  7
 
 491
 
 gene;island
 
 0.846733
 
 0.9839866
 
 RNF220 (margin: RNF220)
 
  4.1890567
 
 4.08e-03
 
 2.47e-02
  
 
  
 6
 
 150463775
 
 150464124
 
  PDF   STATS   ENSEMBL   UCSC 
 
  350
 
  7
 
 35296
 
 tss
 
 0.050007
 
 0.5666280
 
 PPP1R14C (margin: PPP1R14C)
 
  4.1277560
 
 4.08e-03
 
 2.47e-02
  
 
  
 1
 
 204966000
 
 204966512
 
  PDF   STATS   ENSEMBL   UCSC 
 
  513
 
  7
 
 962
 
 gene
 
 0.498554
 
 0.9434845
 
 NFASC (margin: NFASC)
 
  3.9999686
 
 4.08e-03
 
 2.47e-02
  
 
  
 2
 
    496713
 
    497417
 
  PDF   STATS   ENSEMBL   UCSC 
 
  705
 
  7
 
 24004
 
 island
 
 0.561086
 
 0.9090610
 
  (margin: AC093326.1)
 
  3.9685584
 
 4.08e-03
 
 2.47e-02
  
 
  
 3
 
 152552410
 
 152552615
 
  PDF   STATS   ENSEMBL   UCSC 
 
  206
 
  7
 
 28665
 
 tss;island
 
 0.223008
 
 0.0177340
 
 P2RY1 (margin: P2RY1; RP11-38P22.2)
 
  3.9231851
 
 4.08e-03
 
 2.47e-02
  
 
  
 1
 
  32827707
 
  32828191
 
  PDF   STATS   ENSEMBL   UCSC 
 
  485
 
  9
 
 395
 
 gene;tss;island
 
 0.660730
 
 0.1154674
 
 TSSK3; FAM229A (margin: TSSK3; RP4-811H24.9; FAM229A; BSDC1)
 
  3.8964352
 
 3.99e-03
 
 2.47e-02
  
 
  
 12
 
  85306648
 
  85307152
 
  PDF   STATS   ENSEMBL   UCSC 
 
  505
 
  9
 
 9898
 
 tss
 
 0.357884
 
 0.8801732
 
 SLC6A15 (margin: SLC6A15)
 
  3.8280493
 
 3.99e-03
 
 2.47e-02
  
 
  
 6
 
  39693159
 
  39693479
 
  PDF   STATS   ENSEMBL   UCSC 
 
  321
 
  7
 
 36110
 
 island
 
 0.140376
 
 0.0122924
 
 KIF6 (margin: KIF6)
 
  3.7218975
 
 4.08e-03
 
 2.47e-02
  
 
  
 3
 
 192126584
 
 192126996
 
  PDF   STATS   ENSEMBL   UCSC 
 
  413
 
  7
 
 28085
 
 gene;island
 
 0.662388
 
 0.9565586
 
 FGF12 (margin: FGF12)
 
  3.6117073
 
 4.08e-03
 
 2.47e-02
  
 
  
 14
 
  56232650
 
  56233178
 
  PDF   STATS   ENSEMBL   UCSC 
 
  529
 
  7
 
 12871
 
 island
 
 0.576525
 
 0.9355912
 
 RP11-813I20.2; RPL13AP3 (margin: RP11-813I20.2; RPL13AP3)
 
  3.5640497
 
 4.08e-03
 
 2.47e-02
  
 
  
 12
 
  48398320
 
  48398730
 
  PDF   STATS   ENSEMBL   UCSC 
 
  411
 
  7
 
 9665
 
 tss;island
 
 0.663298
 
 0.9597108
 
 COL2A1; RP1-228P16.3 (margin: RP1-228P16.4; COL2A1; RP1-228P16.3)
 
  3.5221275
 
 4.08e-03
 
 2.47e-02
  
 
  
 4
 
  74734714
 
  74735101
 
  PDF   STATS   ENSEMBL   UCSC 
 
  388
 
  9
 
 30430
 
 tss
 
 0.170937
 
 0.4332985
 
 CXCL1 (margin: CXCL1)
 
  3.4840891
 
 3.99e-03
 
 2.47e-02
  
 
  
 20
 
  39995539
 
  39995747
 
  PDF   STATS   ENSEMBL   UCSC 
 
  209
 
  7
 
 25746
 
 tss;island
 
 0.379364
 
 0.9389697
 
 EMILIN3 (margin: LPIN3; EMILIN3)
 
  3.4693905
 
 4.08e-03
 
 2.47e-02
  
 
  
 15
 
  90792609
 
  90793056
 
  PDF   STATS   ENSEMBL   UCSC 
 
  448
 
  7
 
 14450
 
 island
 
 0.455505
 
 0.0222432
 
 TTLL13; RP11-697E2.6 (margin: GDPGP1; RP11-697E2.4; TTLL13; RP11-697E2.6)
 
  3.2954702
 
 4.08e-03
 
 2.47e-02
  
 
  
 6
 
 167275684
 
 167275999
 
  PDF   STATS   ENSEMBL   UCSC 
 
  316
 
  9
 
 36637
 
 island
 
 0.751792
 
 0.1840614
 
 RPS6KA2; RP11-514O12.4 (margin: RPS6KA2; RP11-514O12.4)
 
  3.1265092
 
 3.99e-03
 
 2.47e-02
  
 
  
 1
 
   1395945
 
   1396296
 
  PDF   STATS   ENSEMBL   UCSC 
 
  352
 
  7
 
 37
 
 gene;island
 
 0.669091
 
 0.9795902
 
 ATAD3C (margin: ATAD3C; ATAD3B)
 
  3.0592128
 
 4.08e-03
 
 2.47e-02
  
 
  
 3
 
 122399230
 
 122399601
 
  PDF   STATS   ENSEMBL   UCSC 
 
  372
 
  7
 
 28540
 
 tss
 
 0.014170
 
 0.1501274
 
 PARP14 (margin: PARP14)
 
  3.0513082
 
 4.08e-03
 
 2.47e-02
  
 
  
 8
 
 102504447
 
 102504859
 
  PDF   STATS   ENSEMBL   UCSC 
 
  413
 
  9
 
 40506
 
 island
 
 0.043483
 
 0.0056691
 
 GRHL2; KB-1562D12.1 (margin: GRHL2; KB-1562D12.1)
 
  2.9260033
 
 3.99e-03
 
 2.47e-02
  
 
  
 7
 
 121784351
 
 121784596
 
  PDF   STATS   ENSEMBL   UCSC 
 
  246
 
  7
 
 37864
 
 tss
 
 0.074548
 
 0.3785709
 
 AASS (margin: AASS)
 
  2.9190328
 
 4.08e-03
 
 2.47e-02
  
 
  
 19
 
  46526333
 
  46526675
 
  PDF   STATS   ENSEMBL   UCSC 
 
  343
 
  7
 
 20889
 
 tss
 
 0.600238
 
 0.9409812
 
 CCDC61; PGLYRP1 (margin: CCDC61; MIR769; CTC-344H19.4; PGLYRP1)
 
  2.8254312
 
 4.08e-03
 
 2.47e-02
  
 
  
 5
 
   2112109
 
   2112561
 
  PDF   STATS   ENSEMBL   UCSC 
 
  453
 
  7
 
 32652
 
 island
 
 0.640088
 
 0.9344770
 
  (margin: )
 
  2.6181007
 
 4.08e-03
 
 2.47e-02
  
 
  
 2
 
 223162666
 
 223163326
 
  PDF   STATS   ENSEMBL   UCSC 
 
  661
 
  9
 
 23047
 
 gene;island
 
 0.810193
 
 0.9791638
 
 CCDC140; PAX3 (margin: CCDC140; PAX3)
 
  2.4316050
 
 3.99e-03
 
 2.47e-02
  
 
  
 1
 
  40782991
 
  40783264
 
  PDF   STATS   ENSEMBL   UCSC 
 
  274
 
  7
 
 1535
 
 tss
 
 0.900811
 
 0.9775087
 
 COL9A2 (margin: COL9A2)
 
  2.4282367
 
 4.08e-03
 
 2.47e-02
  
 
  
 17
 
  57184330
 
  57184624
 
  PDF   STATS   ENSEMBL   UCSC 
 
  295
 
  9
 
 17541
 
 tss
 
 0.007384
 
 0.0217556
 
 AC099850.1; TRIM37 (margin: AC099850.1; TRIM37; SKA2)
 
  2.2767955
 
 3.99e-03
 
 2.47e-02
  
 
  
 12
 
   7055452
 
   7056008
 
  PDF   STATS   ENSEMBL   UCSC 
 
  557
 
  7
 
 10221
 
 island
 
 0.066742
 
 0.0222652
 
 C12orf57; PTPN6; U47924.31 (margin: ATN1; C12orf57; RNU7-1; PTPN6; U47924.31)
 
  2.2533259
 
 4.08e-03
 
 2.47e-02
  
 
  
 5
 
 178450457
 
 178451176
 
  PDF   STATS   ENSEMBL   UCSC 
 
  720
 
  9
 
 33478
 
 island
 
 0.076743
 
 0.3091348
 
 ZNF879 (margin: ZNF879)
 
  2.2429811
 
 3.99e-03
 
 2.47e-02
  
 
  
 19
 
  53073242
 
  53073420
 
  PDF   STATS   ENSEMBL   UCSC 
 
  179
 
  7
 
 21016
 
 tss
 
 0.834115
 
 0.4772594
 
 ZNF701 (margin: ZNF808; ZNF701; RPL39P34; CTD-3099C6.7)
 
  2.1743443
 
 4.08e-03
 
 2.47e-02
  
 
  
 22
 
  39640835
 
  39641154
 
  PDF   STATS   ENSEMBL   UCSC 
 
  320
 
  7
 
 27500
 
 island
 
 0.107085
 
 0.0273878
 
 PDGFB (margin: PDGFB)
 
  2.1336210
 
 4.08e-03
 
 2.47e-02
  
 
  
 4
 
  11430552
 
  11430908
 
  PDF   STATS   ENSEMBL   UCSC 
 
  357
 
  7
 
 30302
 
 tss
 
 0.010992
 
 0.0518103
 
 HS3ST1 (margin: HS3ST1)
 
  1.7947585
 
 4.08e-03
 
 2.47e-02
  
 
  
 3
 
  49314539
 
  49314920
 
  PDF   STATS   ENSEMBL   UCSC 
 
  382
 
  9
 
 28325
 
 tss
 
 0.952964
 
 0.9819486
 
 C3orf62; USP4 (margin: Y_RNA; MIR4271; C3orf62; USP4)
 
  1.5789868
 
 3.99e-03
 
 2.47e-02
  
 
  
 6
 
  33561181
 
  33561449
 
  PDF   STATS   ENSEMBL   UCSC 
 
  269
 
  7
 
 34910
 
 tss
 
 0.971292
 
 0.9336733
 
 LINC00336 (margin: GGNBP1; LINC00336)
 
  1.4592800
 
 4.08e-03
 
 2.47e-02
  
 
  
 7
 
  83824255
 
  83824481
 
  PDF   STATS   ENSEMBL   UCSC 
 
  227
 
  7
 
 37679
 
 tss
 
 0.014877
 
 0.1000268
 
 SEMA3A (margin: SEMA3A)
 
  1.4289127
 
 4.08e-03
 
 2.47e-02
  
 
  
 7
 
  12443478
 
  12444290
 
  PDF   STATS   ENSEMBL   UCSC 
 
  813
 
 11
 
 38266
 
 island
 
 0.705256
 
 0.0662933
 
 VWDE (margin: VWDE)
 
  6.6192820
 
 4.10e-03
 
 2.48e-02
  
 
  
 13
 
  25745946
 
  25746644
 
  PDF   STATS   ENSEMBL   UCSC 
 
  699
 
 11
 
 11350
 
 tss
 
 0.657428
 
 0.9175573
 
 AMER2; RP11-165I9.4 (margin: RP11-165I9.8; AMER2; RP11-165I9.4)
 
  2.8529965
 
 4.10e-03
 
 2.48e-02
  
 
  
 19
 
  37157565
 
  37157995
 
  PDF   STATS   ENSEMBL   UCSC 
 
  431
 
 11
 
 21944
 
 island
 
 0.039552
 
 0.0058397
 
 ZNF461 (margin: ZNF461)
 
  2.0041857
 
 4.10e-03
 
 2.48e-02
  
 
  
 2
 
 219866424
 
 219866628
 
  PDF   STATS   ENSEMBL   UCSC 
 
  205
 
  6
 
 25102
 
 island
 
 0.507048
 
 0.0079614
 
 AC097468.4; MIR375; CCDC108 (margin: AC097468.4; CRYBA2; MIR375; CCDC108)
 
  9.8429573
 
 4.33e-03
 
 2.52e-02
  
 
  
 6
 
  76059450
 
  76059773
 
  PDF   STATS   ENSEMBL   UCSC 
 
  324
 
  6
 
 34396
 
 gene
 
 0.741377
 
 0.0296176
 
 FILIP1 (margin: RP11-415D17.3; FILIP1)
 
  8.4268277
 
 4.33e-03
 
 2.52e-02
  
 
  
 4
 
 172734266
 
 172734514
 
  PDF   STATS   ENSEMBL   UCSC 
 
  249
 
  6
 
 30678
 
 tss;island
 
 0.712600
 
 0.0120230
 
 GALNTL6 (margin: GALNTL6)
 
  7.4372328
 
 4.33e-03
 
 2.52e-02
  
 
  
 17
 
  46681111
 
  46681401
 
  PDF   STATS   ENSEMBL   UCSC 
 
  291
 
  6
 
 16656
 
 gene
 
 0.003590
 
 0.6286722
 
 HOXB-AS3; HOXB3; HOXB6 (margin: HOXB-AS3; HOXB3; HOXB5; HOXB6; HOXB7; HOXB8)
 
  7.2951932
 
 4.33e-03
 
 2.52e-02
  
 
  
 4
 
  77172617
 
  77172841
 
  PDF   STATS   ENSEMBL   UCSC 
 
  225
 
  6
 
 30447
 
 tss
 
 0.104825
 
 0.0020151
 
 FAM47E; FAM47E-STBD1 (margin: FAM47E; FAM47E-STBD1)
 
  7.1917866
 
 4.33e-03
 
 2.52e-02
  
 
  
 11
 
  35441012
 
  35441311
 
  PDF   STATS   ENSEMBL   UCSC 
 
  300
 
  6
 
 8207
 
 island
 
 0.602157
 
 0.0166108
 
 RP4-683L5.1; SLC1A2 (margin: RP4-683L5.1; SLC1A2)
 
  7.1664719
 
 4.33e-03
 
 2.52e-02
  
 
  
 6
 
 166419479
 
 166419943
 
  PDF   STATS   ENSEMBL   UCSC 
 
  465
 
  6
 
 36625
 
 island
 
 0.304983
 
 0.0043978
 
  (margin: )
 
  6.8754352
 
 4.33e-03
 
 2.52e-02
  
 
  
 4
 
 113970617
 
 113970935
 
  PDF   STATS   ENSEMBL   UCSC 
 
  319
 
  6
 
 30103
 
 gene
 
 0.041525
 
 0.8236436
 
 ANK2; RP11-650J17.1 (margin: ANK2; RP11-650J17.1)
 
  6.3554281
 
 4.33e-03
 
 2.52e-02
  
 
  
 10
 
 118084109
 
 118084587
 
  PDF   STATS   ENSEMBL   UCSC 
 
  479
 
  6
 
 4775
 
 gene
 
 0.087577
 
 0.9122135
 
 CCDC172 (margin: CCDC172)
 
  6.1451008
 
 4.33e-03
 
 2.52e-02
  
 
  
 7
 
  28319343
 
  28319676
 
  PDF   STATS   ENSEMBL   UCSC 
 
  334
 
  6
 
 37504
 
 tss
 
 0.105150
 
 0.9239470
 
 AC005017.2 (margin: AC005017.2)
 
  6.1207416
 
 4.33e-03
 
 2.52e-02
  
 
  
 11
 
  35639492
 
  35639686
 
  PDF   STATS   ENSEMBL   UCSC 
 
  195
 
  6
 
 7247
 
 tss;island
 
 0.018668
 
 0.6015756
 
 FJX1 (margin: FJX1)
 
  6.0199824
 
 4.33e-03
 
 2.52e-02
  
 
  
 1
 
 186649153
 
 186649530
 
  PDF   STATS   ENSEMBL   UCSC 
 
  378
 
  6
 
 925
 
 gene
 
 0.157196
 
 0.0036518
 
 PTGS2 (margin: PTGS2)
 
  5.8703641
 
 4.33e-03
 
 2.52e-02
  
 
  
 4
 
   5712581
 
   5712884
 
  PDF   STATS   ENSEMBL   UCSC 
 
  304
 
  6
 
 30276
 
 tss
 
 0.031829
 
 0.6309700
 
 EVC; EVC2 (margin: EVC; EVC2)
 
  5.7586559
 
 4.33e-03
 
 2.52e-02
  
 
  
 1
 
  60539362
 
  60539675
 
  PDF   STATS   ENSEMBL   UCSC 
 
  314
 
  6
 
 3327
 
 island
 
 0.636488
 
 0.9820838
 
 C1orf87 (margin: C1orf87)
 
  5.6817953
 
 4.33e-03
 
 2.52e-02
  
 
  
 4
 
 154125208
 
 154125594
 
  PDF   STATS   ENSEMBL   UCSC 
 
  387
 
  6
 
 30640
 
 tss
 
 0.878636
 
 0.2769437
 
 TRIM2 (margin: TRIM2)
 
  5.5917120
 
 4.33e-03
 
 2.52e-02
  
 
  
 11
 
    615945
 
    616112
 
  PDF   STATS   ENSEMBL   UCSC 
 
  168
 
  6
 
 7020
 
 tss;island
 
 0.479860
 
 0.0116634
 
 IRF7; CDHR5 (margin: PHRF1; IRF7; CDHR5; SCT)
 
  5.4720401
 
 4.33e-03
 
 2.52e-02
  
 
  
 2
 
  30454146
 
  30454363
 
  PDF   STATS   ENSEMBL   UCSC 
 
  218
 
  6
 
 23285
 
 tss
 
 0.213201
 
 0.7157188
 
 LBH (margin: LBH)
 
  5.4567422
 
 4.33e-03
 
 2.52e-02
  
 
  
 17
 
  71640253
 
  71640369
 
  PDF   STATS   ENSEMBL   UCSC 
 
  117
 
  6
 
 17618
 
 tss
 
 0.419848
 
 0.0165482
 
 RP11-277J6.2; SDK2 (margin: RP11-277J6.2; SDK2)
 
  5.4020184
 
 4.33e-03
 
 2.52e-02
  
 
  
 15
 
  73661642
 
  73662002
 
  PDF   STATS   ENSEMBL   UCSC 
 
  361
 
  6
 
 13781
 
 tss
 
 0.413589
 
 0.9603015
 
 HCN4 (margin: HCN4)
 
  5.1754344
 
 4.33e-03
 
 2.52e-02
  
 
  
 1
 
  77747875
 
  77748221
 
  PDF   STATS   ENSEMBL   UCSC 
 
  347
 
  6
 
 1705
 
 tss
 
 0.459902
 
 0.0207547
 
 AK5 (margin: AK5)
 
  5.1472867
 
 4.33e-03
 
 2.52e-02
  
 
  
 1
 
 236227410
 
 236227686
 
  PDF   STATS   ENSEMBL   UCSC 
 
  277
 
  6
 
 1096
 
 gene;island
 
 0.219113
 
 0.9702027
 
 NID1 (margin: Y_RNA; NID1)
 
  4.9006514
 
 4.33e-03
 
 2.52e-02
  
 
  
 17
 
  46703854
 
  46704004
 
  PDF   STATS   ENSEMBL   UCSC 
 
  151
 
  6
 
 17468
 
 tss;island
 
 0.008080
 
 0.1313161
 
 HOXB7; HOXB9 (margin: HOXB-AS4; HOXB7; HOXB8; HOXB9; MIR196A1)
 
  4.8564904
 
 4.33e-03
 
 2.52e-02
  
 
  
 5
 
 153857789
 
 153858102
 
  PDF   STATS   ENSEMBL   UCSC 
 
  314
 
  6
 
 33329
 
 island
 
 0.401517
 
 0.9504249
 
 HAND1 (margin: HAND1; CTB-158E9.1)
 
  4.8435640
 
 4.33e-03
 
 2.52e-02
  
 
  
 18
 
    580188
 
    580334
 
  PDF   STATS   ENSEMBL   UCSC 
 
  147
 
  6
 
 19151
 
 tss
 
 0.466927
 
 0.9638346
 
 CETN1 (margin: CETN1; RN7SKP146)
 
  4.7761131
 
 4.33e-03
 
 2.52e-02
  
 
  
 2
 
  71205052
 
  71205564
 
  PDF   STATS   ENSEMBL   UCSC 
 
  513
 
  6
 
 23424
 
 tss
 
 0.228566
 
 0.8868975
 
 ANKRD53; AC007040.11 (margin: ANKRD53; AC007040.11; TEX261)
 
  4.4380786
 
 4.33e-03
 
 2.52e-02
  
 
  
 20
 
  50158996
 
  50159653
 
  PDF   STATS   ENSEMBL   UCSC 
 
  658
 
  6
 
 25454
 
 gene;island
 
 0.817214
 
 0.1927072
 
 NFATC2 (margin: NFATC2)
 
  4.4107641
 
 4.33e-03
 
 2.52e-02
  
 
  
 2
 
    264120
 
    264204
 
  PDF   STATS   ENSEMBL   UCSC 
 
   85
 
  6
 
 23152
 
 tss;island
 
 0.109763
 
 0.0049111
 
 ACP1; SH3YL1 (margin: ACP1; SH3YL1)
 
  4.3792487
 
 4.33e-03
 
 2.52e-02
  
 
  
 12
 
  26348331
 
  26348529
 
  PDF   STATS   ENSEMBL   UCSC 
 
  199
 
  6
 
 9086
 
 gene;island
 
 0.282266
 
 0.0218685
 
 SSPN (margin: SSPN)
 
  4.2935477
 
 4.33e-03
 
 2.52e-02
  
 
  
 11
 
 134201447
 
 134201640
 
  PDF   STATS   ENSEMBL   UCSC 
 
  194
 
  6
 
 7833
 
 tss;island
 
 0.319516
 
 0.0280154
 
 GLB1L2 (margin: GLB1L3; GLB1L2)
 
  4.2494927
 
 4.33e-03
 
 2.52e-02
  
 
  
 8
 
 144069457
 
 144069888
 
  PDF   STATS   ENSEMBL   UCSC 
 
  432
 
  6
 
 39470
 
 gene
 
 0.820375
 
 0.1378141
 
 RP11-273G15.2 (margin: CDC42P3; RP11-273G15.2)
 
  4.1838555
 
 4.33e-03
 
 2.52e-02
  
 
  
 1
 
   2537547
 
   2537838
 
  PDF   STATS   ENSEMBL   UCSC 
 
  292
 
  6
 
 92
 
 gene;island
 
 0.456664
 
 0.9445793
 
 MMEL1 (margin: MMEL1)
 
  4.1404305
 
 4.33e-03
 
 2.52e-02
  
 
  
 8
 
  10261641
 
  10262221
 
  PDF   STATS   ENSEMBL   UCSC 
 
  581
 
  6
 
 39195
 
 gene;island
 
 0.470947
 
 0.9330135
 
 MSRA (margin: MSRA)
 
  4.1305032
 
 4.33e-03
 
 2.52e-02
  
 
  
 20
 
  30073209
 
  30073576
 
  PDF   STATS   ENSEMBL   UCSC 
 
  368
 
  6
 
 25660
 
 tss;island
 
 0.496932
 
 0.8520254
 
 REM1; LINC00028 (margin: REM1; LINC00028; DEFB124)
 
  4.0709373
 
 4.33e-03
 
 2.52e-02
  
 
  
 12
 
  79257496
 
  79257853
 
  PDF   STATS   ENSEMBL   UCSC 
 
  358
 
  6
 
 9890
 
 tss
 
 0.284925
 
 0.0166485
 
 SYT1 (margin: SYT1)
 
  4.0469344
 
 4.33e-03
 
 2.52e-02
  
 
  
 7
 
  18535072
 
  18535499
 
  PDF   STATS   ENSEMBL   UCSC 
 
  428
 
  6
 
 37458
 
 tss
 
 0.597630
 
 0.1977708
 
 HDAC9 (margin: HDAC9)
 
  4.0412956
 
 4.33e-03
 
 2.52e-02
  
 
  
 4
 
   1504989
 
   1505559
 
  PDF   STATS   ENSEMBL   UCSC 
 
  571
 
  6
 
 30788
 
 island
 
 0.438145
 
 0.9210637
 
  (margin: )
 
  3.9228388
 
 4.33e-03
 
 2.52e-02
  
 
  
 2
 
  39893078
 
  39893520
 
  PDF   STATS   ENSEMBL   UCSC 
 
  443
 
  6
 
 22654
 
 gene;island
 
 0.782332
 
 0.9868423
 
 AC007246.3; TMEM178A (margin: AC007246.3; TMEM178A)
 
  3.7968574
 
 4.33e-03
 
 2.52e-02
  
 
  
 10
 
  14051636
 
  14052028
 
  PDF   STATS   ENSEMBL   UCSC 
 
  393
 
  6
 
 4508
 
 gene
 
 0.603613
 
 0.9650231
 
 FRMD4A (margin: RP11-142M10.2; FRMD4A)
 
  3.7568786
 
 4.33e-03
 
 2.52e-02
  
 
  
 4
 
 122301573
 
 122302007
 
  PDF   STATS   ENSEMBL   UCSC 
 
  435
 
  6
 
 30112
 
 gene;island
 
 0.646415
 
 0.9677485
 
 QRFPR (margin: QRFPR; RP11-364P2.2)
 
  3.7207898
 
 4.33e-03
 
 2.52e-02
  
 
  
 1
 
  50889182
 
  50889510
 
  PDF   STATS   ENSEMBL   UCSC 
 
  329
 
  6
 
 1616
 
 tss
 
 0.678095
 
 0.9769040
 
 DMRTA2 (margin: DMRTA2)
 
  3.6333313
 
 4.33e-03
 
 2.52e-02
  
 
  
 19
 
  49993125
 
  49993217
 
  PDF   STATS   ENSEMBL   UCSC 
 
   93
 
  6
 
 20154
 
 gene;tss;island
 
 0.115124
 
 0.6077468
 
 RPL13A; SNORD32A; SNORD33; SNORD34; SNORD35A (margin: CTD-3148I10.9; FLT3LG; RPL13A; SNORD32A; SNORD33; SNORD34; SNORD35A; RPS11; SNORD35B; hsa-mir-150; MIR150)
 
  3.5721938
 
 4.33e-03
 
 2.52e-02
  
 
  
 11
 
 110583543
 
 110583662
 
  PDF   STATS   ENSEMBL   UCSC 
 
  120
 
  6
 
 7679
 
 tss
 
 0.454362
 
 0.9038837
 
 ARHGAP20 (margin: ARHGAP20)
 
  3.5356334
 
 4.33e-03
 
 2.52e-02
  
 
  
 1
 
  91191583
 
  91192162
 
  PDF   STATS   ENSEMBL   UCSC 
 
  580
 
  6
 
 3463
 
 island
 
 0.688544
 
 0.9761543
 
  (margin: BARHL2)
 
  3.5338820
 
 4.33e-03
 
 2.52e-02
  
 
  
 6
 
  33053576
 
  33054001
 
  PDF   STATS   ENSEMBL   UCSC 
 
  426
 
  6
 
 34187
 
 gene
 
 0.209625
 
 0.6034706
 
 HLA-DPB1 (margin: HLA-DPB1; RPL32P1; HLA-DPA1; HLA-DPA2)
 
  3.4968812
 
 4.33e-03
 
 2.52e-02
  
 
  
 7
 
 158886098
 
 158886532
 
  PDF   STATS   ENSEMBL   UCSC 
 
  435
 
  6
 
 37384
 
 gene;island
 
 0.611297
 
 0.9578905
 
 VIPR2 (margin: VIPR2)
 
  3.4950960
 
 4.33e-03
 
 2.52e-02
  
 
  
 7
 
  14028690
 
  14029085
 
  PDF   STATS   ENSEMBL   UCSC 
 
  396
 
  6
 
 36898
 
 gene;island
 
 0.537135
 
 0.0795487
 
 ETV1 (margin: ETV1)
 
  3.4760116
 
 4.33e-03
 
 2.52e-02
  
 
  
 7
 
 129425330
 
 129425932
 
  PDF   STATS   ENSEMBL   UCSC 
 
  603
 
  6
 
 38913
 
 island
 
 0.613647
 
 0.9516694
 
 RP11-738B7.1 (margin: MIR96; MIR183; RP11-738B7.1)
 
  3.4219352
 
 4.33e-03
 
 2.52e-02
  
 
  
 6
 
  31508106
 
  31508318
 
  PDF   STATS   ENSEMBL   UCSC 
 
  213
 
  6
 
 33894
 
 gene;island
 
 0.154720
 
 0.5505411
 
 DDX39B-AS1; DDX39B; ATP6V1G2-DDX39B; SNORD84 (margin: RPL15P4; MCCD1; DDX39B-AS1; NFKBIL1; DDX39B; ATP6V1G2-DDX39B; SNORD117; SNORD84; ATP6V1G2)
 
  3.3171841
 
 4.33e-03
 
 2.52e-02
  
 
  
 8
 
  21644953
 
  21645524
 
  PDF   STATS   ENSEMBL   UCSC 
 
  572
 
  6
 
 39224
 
 gene;island
 
 0.833643
 
 0.9514623
 
 GFRA2 (margin: GFRA2)
 
  3.1960225
 
 4.33e-03
 
 2.52e-02
  
 
  
 1
 
   1177703
 
   1178245
 
  PDF   STATS   ENSEMBL   UCSC 
 
  543
 
  6
 
 2417
 
 island
 
 0.753598
 
 0.9728451
 
 FAM132A (margin: B3GALT6; SDF4; FAM132A; RP5-902P8.12; UBE2J2)
 
  3.1682410
 
 4.33e-03
 
 2.52e-02
  
 
  
 1
 
 201617847
 
 201618284
 
  PDF   STATS   ENSEMBL   UCSC 
 
  438
 
  6
 
 940
 
 gene;island
 
 0.603554
 
 0.1464076
 
 NAV1 (margin: NAV1)
 
  3.0724479
 
 4.33e-03
 
 2.52e-02
  
 
  
 2
 
 242801896
 
 242802192
 
  PDF   STATS   ENSEMBL   UCSC 
 
  297
 
  6
 
 23995
 
 tss;island
 
 0.661121
 
 0.9672036
 
 PDCD1 (margin: CXXC11; PDCD1)
 
  3.0202322
 
 4.33e-03
 
 2.52e-02
  
 
  
 11
 
   1083178
 
   1083509
 
  PDF   STATS   ENSEMBL   UCSC 
 
  332
 
  6
 
 6402
 
 gene;island
 
 0.760257
 
 0.9586873
 
 MUC2 (margin: MUC2)
 
  2.9499201
 
 4.33e-03
 
 2.52e-02
  
 
  
 11
 
  17373103
 
  17373302
 
  PDF   STATS   ENSEMBL   UCSC 
 
  200
 
  6
 
 7174
 
 tss;island
 
 0.098264
 
 0.0181081
 
 NUCB2; NCR3LG1 (margin: NUCB2; NCR3LG1)
 
  2.6697510
 
 4.33e-03
 
 2.52e-02
  
 
  
 1
 
  47696212
 
  47696788
 
  PDF   STATS   ENSEMBL   UCSC 
 
  577
 
  6
 
 1607
 
 tss;island
 
 0.855688
 
 0.9780241
 
 RP1-18D14.7; TAL1 (margin: RP1-18D14.7; TAL1)
 
  2.6673070
 
 4.33e-03
 
 2.52e-02
  
 
  
 1
 
 200011684
 
 200011988
 
  PDF   STATS   ENSEMBL   UCSC 
 
  305
 
  6
 
 934
 
 gene;island
 
 0.913987
 
 0.9770294
 
 NR5A2 (margin: NR5A2; RNU6-570P)
 
  2.5721434
 
 4.33e-03
 
 2.52e-02
  
 
  
 6
 
 106959535
 
 106959651
 
  PDF   STATS   ENSEMBL   UCSC 
 
  117
 
  6
 
 35151
 
 tss;island
 
 0.015386
 
 0.0771104
 
 AIM1 (margin: AIM1)
 
  2.5418777
 
 4.33e-03
 
 2.52e-02
  
 
  
 8
 
 143459774
 
 143460209
 
  PDF   STATS   ENSEMBL   UCSC 
 
  436
 
  6
 
 39462
 
 gene
 
 0.863331
 
 0.9675119
 
 TSNARE1 (margin: TSNARE1)
 
  2.4716472
 
 4.33e-03
 
 2.52e-02
  
 
  
 2
 
 198669113
 
 198669339
 
  PDF   STATS   ENSEMBL   UCSC 
 
  227
 
  6
 
 23804
 
 tss;island
 
 0.122686
 
 0.0147450
 
 PLCL1 (margin: PLCL1)
 
  2.4370508
 
 4.33e-03
 
 2.52e-02
  
 
  
 15
 
  50979137
 
  50979486
 
  PDF   STATS   ENSEMBL   UCSC 
 
  350
 
  6
 
 13670
 
 tss;island
 
 0.014115
 
 0.0364647
 
 RN7SL354P; TRPM7 (margin: RN7SL354P; TRPM7)
 
  2.2090607
 
 4.33e-03
 
 2.52e-02
  
 
  
 6
 
  32908466
 
  32908794
 
  PDF   STATS   ENSEMBL   UCSC 
 
  329
 
  6
 
 34163
 
 gene
 
 0.009984
 
 0.0454600
 
 HLA-DMB; XXbac-BPG181M17.5 (margin: HLA-DMB; AL645941.1; XXbac-BPG181M17.5; HLA-DMA)
 
  2.1807621
 
 4.33e-03
 
 2.52e-02
  
 
  
 17
 
  15165908
 
  15166153
 
  PDF   STATS   ENSEMBL   UCSC 
 
  246
 
  6
 
 17149
 
 tss
 
 0.068811
 
 0.0159189
 
 RP11-849N15.1; PMP22 (margin: RP11-849N15.1; PMP22; MIR4731; AC005703.3)
 
  2.1157802
 
 4.33e-03
 
 2.52e-02
  
 
  
 11
 
  67777618
 
  67777770
 
  PDF   STATS   ENSEMBL   UCSC 
 
  153
 
  6
 
 7503
 
 tss
 
 0.021765
 
 0.0870191
 
 ALDH3B1 (margin: ALDH3B1; UNC93B1)
 
  1.9564591
 
 4.33e-03
 
 2.52e-02
  
 
  
 11
 
  60623504
 
  60623918
 
  PDF   STATS   ENSEMBL   UCSC 
 
  415
 
  6
 
 7323
 
 tss
 
 0.598316
 
 0.8737915
 
 PTGDR2 (margin: CCDC86; ZP1; RP11-804A23.4; PTGDR2)
 
  1.8651459
 
 4.33e-03
 
 2.52e-02
  
 
  
 22
 
  39640959
 
  39641154
 
  PDF   STATS   ENSEMBL   UCSC 
 
  196
 
  6
 
 27150
 
 tss
 
 0.101303
 
 0.0271727
 
 PDGFB (margin: PDGFB)
 
  1.8194497
 
 4.33e-03
 
 2.52e-02
  
 
  
 2
 
  44065259
 
  44065893
 
  PDF   STATS   ENSEMBL   UCSC 
 
  635
 
 13
 
 22661
 
 gene
 
 0.769590
 
 0.8885374
 
 ABCG8; ABCG5 (margin: ABCG8; ABCG5)
 
  1.5712910
 
 4.22e-03
 
 2.52e-02
  
 
  
 15
 
  26108612
 
  26108948
 
  PDF   STATS   ENSEMBL   UCSC 
 
  337
 
 12
 
 13539
 
 tss;island
 
 0.751221
 
 0.9568451
 
 RP11-2C7.1; ATP10A (margin: RP11-2C7.1; ATP10A)
 
  2.3634525
 
 4.51e-03
 
 2.62e-02
  
 
  
 6
 
  31628050
 
  31629199
 
  PDF   STATS   ENSEMBL   UCSC 
 
 1150
 
 25
 
 35813
 
 island
 
 0.005318
 
 0.0272504
 
 C6orf47-AS1; Y_RNA; C6orf47; GPANK1 (margin: APOM; C6orf47-AS1; Y_RNA; CSNK2B; CSNK2B-LY6G5B-1181; LY6G5B; BAG6; C6orf47; GPANK1)
 
  1.9165594
 
 4.53e-03
 
 2.63e-02
  
 
  
 2
 
 178937517
 
 178937882
 
  PDF   STATS   ENSEMBL   UCSC 
 
  366
 
  8
 
 23751
 
 tss
 
 0.241980
 
 0.0110302
 
 PDE11A (margin: PDE11A)
 
  6.2481182
 
 4.66e-03
 
 2.68e-02
  
 
  
 7
 
  50800837
 
  50801423
 
  PDF   STATS   ENSEMBL   UCSC 
 
  587
 
  8
 
 37014
 
 gene;tss
 
 0.158946
 
 0.9376946
 
 GRB10 (margin: GRB10)
 
  5.6128407
 
 4.66e-03
 
 2.68e-02
  
 
  
 5
 
 172661803
 
 172662463
 
  PDF   STATS   ENSEMBL   UCSC 
 
  661
 
  8
 
 33407
 
 island
 
 0.279337
 
 0.9349527
 
 NKX2-5 (margin: RPL7AP33; NKX2-5)
 
  5.4087356
 
 4.66e-03
 
 2.68e-02
  
 
  
 1
 
  43919949
 
  43920264
 
  PDF   STATS   ENSEMBL   UCSC 
 
  316
 
  8
 
 1563
 
 tss
 
 0.440478
 
 0.9442858
 
 SZT2; HYI-AS1; HYI (margin: SZT2; HYI-AS1; SZT2-AS1; HYI)
 
  4.9894251
 
 4.66e-03
 
 2.68e-02
  
 
  
 13
 
  74707860
 
  74708194
 
  PDF   STATS   ENSEMBL   UCSC 
 
  335
 
  8
 
 11779
 
 island
 
 0.143541
 
 0.0069483
 
 KLF12 (margin: KLF12)
 
  4.6824803
 
 4.66e-03
 
 2.68e-02
  
 
  
 3
 
 123813191
 
 123813504
 
  PDF   STATS   ENSEMBL   UCSC 
 
  314
 
  8
 
 28548
 
 tss
 
 0.391695
 
 0.9231001
 
 KALRN (margin: KALRN)
 
  4.5417590
 
 4.66e-03
 
 2.68e-02
  
 
  
 11
 
  94134646
 
  94135029
 
  PDF   STATS   ENSEMBL   UCSC 
 
  384
 
  8
 
 7629
 
 tss
 
 0.684159
 
 0.9842663
 
 GPR83 (margin: GPR83)
 
  4.5030821
 
 4.66e-03
 
 2.68e-02
  
 
  
 19
 
  12624466
 
  12624832
 
  PDF   STATS   ENSEMBL   UCSC 
 
  367
 
  8
 
 21604
 
 island
 
 0.579203
 
 0.9810008
 
 ZNF709 (margin: CTD-3105H18.8; CTD-3105H18.10; CTD-3105H18.9; CTD-3105H18.11; PPIAP20; ZNF709; ZNF564; CTD-2192J16.20)
 
  4.4378241
 
 4.66e-03
 
 2.68e-02
  
 
  
 4
 
 142054660
 
 142054878
 
  PDF   STATS   ENSEMBL   UCSC 
 
  219
 
  8
 
 30609
 
 tss;island
 
 0.137613
 
 0.0053988
 
 RNF150 (margin: RNF150)
 
  4.2717618
 
 4.66e-03
 
 2.68e-02
  
 
  
 3
 
  48697409
 
  48697823
 
  PDF   STATS   ENSEMBL   UCSC 
 
  415
 
  8
 
 27769
 
 gene;island
 
 0.667706
 
 0.9399871
 
 CELSR3 (margin: RP11-148G20.1; CELSR3; NCKIPSD)
 
  3.8532859
 
 4.66e-03
 
 2.68e-02
  
 
  
 6
 
  29523592
 
  29524117
 
  PDF   STATS   ENSEMBL   UCSC 
 
  526
 
  8
 
 33660
 
 gene;island
 
 0.206994
 
 0.6803436
 
 OR2I1P; UBD; GABBR1 (margin: OR2I1P; UBD; GABBR1)
 
  3.6229516
 
 4.66e-03
 
 2.68e-02
  
 
  
 1
 
 205818956
 
 205819609
 
  PDF   STATS   ENSEMBL   UCSC 
 
  654
 
  8
 
 4138
 
 island
 
 0.815614
 
 0.9846418
 
 PM20D1 (margin: PM20D1)
 
  3.3297243
 
 4.66e-03
 
 2.68e-02
  
 
  
 3
 
  42814705
 
  42814948
 
  PDF   STATS   ENSEMBL   UCSC 
 
  244
 
  8
 
 29020
 
 island
 
 0.870840
 
 0.9855173
 
 RP11-70C1.1; CCDC13; HIGD1A (margin: RP11-70C1.1; CCDC13; HIGD1A)
 
  3.2490932
 
 4.66e-03
 
 2.68e-02
  
 
  
 20
 
  56725695
 
  56725899
 
  PDF   STATS   ENSEMBL   UCSC 
 
  205
 
  8
 
 25847
 
 tss
 
 0.757011
 
 0.9620708
 
 C20orf85 (margin: C20orf85)
 
  3.0941402
 
 4.66e-03
 
 2.68e-02
  
 
  
 17
 
   6735063
 
   6735316
 
  PDF   STATS   ENSEMBL   UCSC 
 
  254
 
  8
 
 17054
 
 tss
 
 0.139902
 
 0.0203220
 
 TEKT1 (margin: TEKT1)
 
  2.9386947
 
 4.66e-03
 
 2.68e-02
  
 
  
 5
 
 169532686
 
 169533120
 
  PDF   STATS   ENSEMBL   UCSC 
 
  435
 
  8
 
 33376
 
 island
 
 0.170298
 
 0.6367436
 
 FOXI1 (margin: FOXI1)
 
  2.6523120
 
 4.66e-03
 
 2.68e-02
  
 
  
 17
 
   6616644
 
   6617192
 
  PDF   STATS   ENSEMBL   UCSC 
 
  549
 
  8
 
 17909
 
 island
 
 0.867168
 
 0.9634886
 
 SLC13A5 (margin: SLC13A5)
 
  2.1816801
 
 4.66e-03
 
 2.68e-02
  
 
  
 8
 
  79427993
 
  79428725
 
  PDF   STATS   ENSEMBL   UCSC 
 
  733
 
  8
 
 40406
 
 island
 
 0.221891
 
 0.6628969
 
 PKIA; RP11-594N15.2 (margin: PKIA; RP11-594N15.2)
 
  2.1193045
 
 4.66e-03
 
 2.68e-02
  
 
  
 14
 
  88792913
 
  88793311
 
  PDF   STATS   ENSEMBL   UCSC 
 
  399
 
  8
 
 13057
 
 island
 
 0.297270
 
 0.5243838
 
 KCNK10 (margin: KCNK10)
 
  1.5703019
 
 4.66e-03
 
 2.68e-02
  
 
  
 12
 
  56511647
 
  56511953
 
  PDF   STATS   ENSEMBL   UCSC 
 
  307
 
  8
 
 9784
 
 tss
 
 0.004145
 
 0.0070927
 
 RPL41; ZC3H10; ESYT1; RP11-603J24.6; RP11-603J24.5 (margin: RP11-603J24.9; PA2G4; RPL41; ZC3H10; ESYT1; RP11-603J24.17; RP11-603J24.6; RP11-603J24.5)
 
  1.4365956
 
 4.66e-03
 
 2.68e-02
  
 
  
 6
 
  33130918
 
  33132727
 
  PDF   STATS   ENSEMBL   UCSC 
 
 1810
 
 30
 
 34193
 
 gene;island
 
 0.498822
 
 0.7309101
 
 COL11A2 (margin: COL11A2)
 
  1.5357417
 
 4.76e-03
 
 2.73e-02
  
 
  
 19
 
  11784246
 
  11785337
 
  PDF   STATS   ENSEMBL   UCSC 
 
 1092
 
 15
 
 21580
 
 island
 
 0.690615
 
 0.9579471
 
 ZNF833P (margin: ZNF833P; CTC-499B15.4; HNRNPA1P10)
 
  2.1471249
 
 4.94e-03
 
 2.83e-02
  
 
  
 14
 
 101531403
 
 101532352
 
  PDF   STATS   ENSEMBL   UCSC 
 
  950
 
 20
 
 12634
 
 tss;island
 
 0.565064
 
 0.9227843
 
 MIR541; MIR409; MIR412; MIR369; MIR410; MIR656 (margin: MIR134; MIR485; MIR323B; MIR154; MIR496; MIR377; MIR541; MIR409; MIR412; MIR369; MIR410; MIR656; MEG9; MIR382; AL132709.1)
 
  2.4518477
 
 5.13e-03
 
 2.94e-02
  
 
  
 1
 
 247275326
 
 247276096
 
  PDF   STATS   ENSEMBL   UCSC 
 
  771
 
 10
 
 4414
 
 island
 
 0.694229
 
 0.0490187
 
 C1orf229 (margin: FGFR3P6; ZNF669; C1orf229; ZNF124)
 
  5.0834866
 
 5.20e-03
 
 2.96e-02
  
 
  
 11
 
  65779192
 
  65779847
 
  PDF   STATS   ENSEMBL   UCSC 
 
  656
 
 10
 
 8495
 
 island
 
 0.439260
 
 0.9588249
 
 CST6 (margin: BANF1; CST6; EIF1AD; CATSPER1)
 
  4.9397024
 
 5.20e-03
 
 2.96e-02
  
 
  
 4
 
 110223040
 
 110223795
 
  PDF   STATS   ENSEMBL   UCSC 
 
  756
 
 10
 
 30089
 
 gene
 
 0.661326
 
 0.9621028
 
 AC004051.2; COL25A1 (margin: AC004051.2; COL25A1)
 
  3.5579413
 
 5.20e-03
 
 2.96e-02
  
 
  
 11
 
  69924046
 
  69924390
 
  PDF   STATS   ENSEMBL   UCSC 
 
  345
 
 10
 
 7524
 
 tss;island
 
 0.155892
 
 0.8378570
 
 ANO1 (margin: RNU6-1175P; ANO1; ANO1-AS2)
 
  3.4140192
 
 5.20e-03
 
 2.96e-02
  
 
  
 4
 
   3464653
 
   3465064
 
  PDF   STATS   ENSEMBL   UCSC 
 
  412
 
 10
 
 30839
 
 island
 
 0.371364
 
 0.0282593
 
 DOK7 (margin: DOK7)
 
  3.3831037
 
 5.20e-03
 
 2.96e-02
  
 
  
 4
 
  74734714
 
  74735149
 
  PDF   STATS   ENSEMBL   UCSC 
 
  436
 
 10
 
 31097
 
 island
 
 0.109363
 
 0.4216879
 
 CXCL1 (margin: CXCL1)
 
  3.1891968
 
 5.20e-03
 
 2.96e-02
  
 
  
 19
 
  52391234
 
  52391789
 
  PDF   STATS   ENSEMBL   UCSC 
 
  556
 
 10
 
 20999
 
 tss
 
 0.964228
 
 0.7934613
 
 CTC-429C10.2; ZNF577; ZNF649 (margin: CTC-429C10.2; ZNF577; ZNF649)
 
  2.4107060
 
 5.20e-03
 
 2.96e-02
  
 
  
 11
 
  65601167
 
  65601427
 
  PDF   STATS   ENSEMBL   UCSC 
 
  261
 
 10
 
 8481
 
 island
 
 0.817298
 
 0.9686324
 
 SNX32; CFL1 (margin: SNX32; CFL1)
 
  2.0622832
 
 5.20e-03
 
 2.96e-02
  
 
  
 14
 
 106938234
 
 106938621
 
  PDF   STATS   ENSEMBL   UCSC 
 
  388
 
 10
 
 13248
 
 island
 
 0.870637
 
 0.9666093
 
 LINC00221 (margin: LINC00221; IGHV3-43; IGHVII-43-1; IGHVIII-44; IGHVIV-44-1; IGHVII-44-2)
 
  2.0512274
 
 5.20e-03
 
 2.96e-02
  
 
  
 14
 
 101505891
 
 101506579
 
  PDF   STATS   ENSEMBL   UCSC 
 
  689
 
 11
 
 12625
 
 tss
 
 0.322106
 
 0.9587518
 
 AL132709.2; MIR376C; MIR654; MIR376A1; MIR300 (margin: MIR494; MIR1193; MIR543; MIR495; AL132709.2; MIR376C; MIR654; MIR376A1; MIR300; MIR1185-1; MIR1185-2; MIR381HG; MIR381; MIR487B; MIR539; MIR889; MIR544A; MIR655; AL132709.3)
 
  4.9662410
 
 5.24e-03
 
 2.98e-02
  
 
  
 12
 
  85673860
 
  85674694
 
  PDF   STATS   ENSEMBL   UCSC 
 
  835
 
 11
 
 10658
 
 island
 
 0.630367
 
 0.9731450
 
 ALX1 (margin: ALX1)
 
  4.2347584
 
 5.24e-03
 
 2.98e-02
  
 
  
 4
 
 164253207
 
 164254230
 
  PDF   STATS   ENSEMBL   UCSC 
 
 1024
 
 11
 
 31400
 
 island
 
 0.054112
 
 0.0065403
 
 NPY1R (margin: NPY5R; NPY1R)
 
  4.2312996
 
 5.24e-03
 
 2.98e-02
  
 
  
 16
 
  57318603
 
  57319093
 
  PDF   STATS   ENSEMBL   UCSC 
 
  491
 
 11
 
 15260
 
 tss;island
 
 0.008169
 
 0.0666749
 
 PLLP (margin: PLLP)
 
  3.6945123
 
 5.24e-03
 
 2.98e-02
  
 
  
 1
 
 161067636
 
 161068387
 
  PDF   STATS   ENSEMBL   UCSC 
 
  752
 
 12
 
 3898
 
 island
 
 0.318415
 
 0.0065829
 
 KLHDC9; PFDN2 (margin: RP11-544M22.8; KLHDC9; PVRL4; PFDN2)
 
  4.6968809
 
 5.56e-03
 
 3.16e-02
  
 
  
 10
 
  25464008
 
  25464513
 
  PDF   STATS   ENSEMBL   UCSC 
 
  506
 
 12
 
 4533
 
 gene;island
 
 0.728679
 
 0.0972949
 
 GPR158; GPR158-AS1 (margin: GPR158; GPR158-AS1)
 
  4.0341157
 
 5.56e-03
 
 3.16e-02
  
 
  
 17
 
  57184151
 
  57184624
 
  PDF   STATS   ENSEMBL   UCSC 
 
  474
 
 12
 
 18606
 
 island
 
 0.007404
 
 0.0218102
 
 AC099850.1; TRIM37 (margin: AC099850.1; TRIM37; SKA2)
 
  2.2504942
 
 5.56e-03
 
 3.16e-02
  
 
  
 22
 
  45680498
 
  45681036
 
  PDF   STATS   ENSEMBL   UCSC 
 
  539
 
 12
 
 27573
 
 island
 
 0.762491
 
 0.5221186
 
 UPK3A (margin: CTA-268H5.9; UPK3A)
 
  1.7712518
 
 5.56e-03
 
 3.16e-02
  
 
  
 4
 
 101111147
 
 101111927
 
  PDF   STATS   ENSEMBL   UCSC 
 
  781
 
  9
 
 31203
 
 island
 
 0.742314
 
 0.0171944
 
 RP11-15B17.1; RP11-588P8.1; DDIT4L (margin: RP11-15B17.1; RP11-588P8.1; DDIT4L)
 
  7.7193462
 
 5.64e-03
 
 3.18e-02
  
 
  
 17
 
   1082526
 
   1083491
 
  PDF   STATS   ENSEMBL   UCSC 
 
  966
 
  9
 
 16310
 
 gene;island
 
 0.085581
 
 0.0094073
 
 AC016292.1; ABR (margin: AC016292.1; ABR)
 
  4.3622429
 
 5.64e-03
 
 3.18e-02
  
 
  
 10
 
  61469599
 
  61469980
 
  PDF   STATS   ENSEMBL   UCSC 
 
  382
 
  9
 
 5764
 
 island
 
 0.593549
 
 0.0499418
 
 SLC16A9 (margin: SLC16A9)
 
  3.6839994
 
 5.64e-03
 
 3.18e-02
  
 
  
 10
 
 127464165
 
 127464762
 
  PDF   STATS   ENSEMBL   UCSC 
 
  598
 
  9
 
 6238
 
 island
 
 0.575454
 
 0.8975653
 
 MMP21 (margin: C10orf137; MMP21)
 
  3.6231355
 
 5.64e-03
 
 3.18e-02
  
 
  
 5
 
 131346985
 
 131347773
 
  PDF   STATS   ENSEMBL   UCSC 
 
  789
 
  9
 
 33083
 
 island
 
 0.611266
 
 0.1341127
 
 AC034228.2; ACSL6 (margin: AC034228.3; AC034228.2; ACSL6)
 
  3.4619630
 
 5.64e-03
 
 3.18e-02
  
 
  
 19
 
  40732518
 
  40732902
 
  PDF   STATS   ENSEMBL   UCSC 
 
  385
 
  9
 
 22017
 
 island
 
 0.410154
 
 0.0988252
 
 CNTD2 (margin: MAP3K10; TTC9B; CNTD2; AKT2)
 
  2.5696919
 
 5.64e-03
 
 3.18e-02
  
 
  
 4
 
   3464653
 
   3465004
 
  PDF   STATS   ENSEMBL   UCSC 
 
  352
 
  9
 
 30262
 
 tss
 
 0.409553
 
 0.0300367
 
 DOK7 (margin: DOK7)
 
  2.2888918
 
 5.64e-03
 
 3.18e-02
  
 
  
 7
 
 150755072
 
 150755981
 
  PDF   STATS   ENSEMBL   UCSC 
 
  910
 
 20
 
 37987
 
 tss
 
 0.012987
 
 0.0507835
 
 SLC4A2; CDK5 (margin: ABCB8; ASIC3; SLC4A2; CDK5)
 
  2.0738488
 
 5.62e-03
 
 3.18e-02
  
 
  
 16
 
    699218
 
    699357
 
  PDF   STATS   ENSEMBL   UCSC 
 
  140
 
  5
 
 14972
 
 tss
 
 0.001474
 
 0.9893758
 
 FAM195A; WDR90; AL022341.3 (margin: FAM195A; WDR90; AL022341.1; AL022341.3; LA16c-349E10.1)
 
 17.9568458
 
 7.94e-03
 
 3.19e-02
  
 
  
 7
 
  27153636
 
  27153944
 
  PDF   STATS   ENSEMBL   UCSC 
 
  309
 
  5
 
 37489
 
 tss
 
 0.000914
 
 0.9844781
 
 HOXA-AS2; HOXA3 (margin: HOXA-AS2; HOXA2; HOXA3)
 
 15.9654595
 
 7.94e-03
 
 3.19e-02
  
 
  
 6
 
  26045532
 
  26045987
 
  PDF   STATS   ENSEMBL   UCSC 
 
  456
 
  5
 
 35512
 
 island
 
 0.001452
 
 0.9395841
 
 U91328.2; HIST1H3C; HIST1H2BB (margin: U91328.2; HIST1H3C; HIST1H2AB; HIST1H2BB; HIST1H1C)
 
 14.3191129
 
 7.94e-03
 
 3.19e-02
  
 
  
 3
 
  37902229
 
  37902741
 
  PDF   STATS   ENSEMBL   UCSC 
 
  513
 
  5
 
 28223
 
 tss;island
 
 0.004721
 
 0.9656721
 
 CTDSPL; AC093415.2 (margin: CTDSPL; AC093415.2)
 
 14.3166570
 
 7.94e-03
 
 3.19e-02
  
 
  
 1
 
  90309205
 
  90309605
 
  PDF   STATS   ENSEMBL   UCSC 
 
  401
 
  5
 
 622
 
 gene;island
 
 0.004863
 
 0.9848243
 
 RP11-302M6.4; LRRC8D (margin: RP11-302M6.4; LRRC8D)
 
 14.2036411
 
 7.94e-03
 
 3.19e-02
  
 
  
 16
 
  31483137
 
  31483534
 
  PDF   STATS   ENSEMBL   UCSC 
 
  398
 
  5
 
 15212
 
 tss;island
 
 0.006349
 
 0.9816653
 
 TGFB1I1 (margin: ARMC5; TGFB1I1; SLC5A2)
 
 13.8511987
 
 7.94e-03
 
 3.19e-02
  
 
  
 10
 
  17270300
 
  17270601
 
  PDF   STATS   ENSEMBL   UCSC 
 
  302
 
  5
 
 4517
 
 gene
 
 0.007815
 
 0.9891572
 
 VIM; VIM-AS1 (margin: VIM; VIM-AS1; RP11-124N14.3)
 
 13.6101871
 
 7.94e-03
 
 3.19e-02
  
 
  
 1
 
  24307153
 
  24307535
 
  PDF   STATS   ENSEMBL   UCSC 
 
  383
 
  5
 
 1354
 
 tss;island
 
 0.001968
 
 0.9714716
 
 SRSF10 (margin: SRSF10)
 
 13.6012334
 
 7.94e-03
 
 3.19e-02
  
 
  
 4
 
  40517938
 
  40518143
 
  PDF   STATS   ENSEMBL   UCSC 
 
  206
 
  5
 
 29987
 
 gene
 
 0.011117
 
 0.9731692
 
 RBM47 (margin: RBM47)
 
 13.4247717
 
 7.94e-03
 
 3.19e-02
  
 
  
 11
 
  33037397
 
  33037626
 
  PDF   STATS   ENSEMBL   UCSC 
 
  230
 
  5
 
 7230
 
 tss
 
 0.009358
 
 0.9893526
 
 DEPDC7 (margin: DEPDC7; Y_RNA)
 
 13.3455743
 
 7.94e-03
 
 3.19e-02
  
 
  
 13
 
  88323266
 
  88323607
 
  PDF   STATS   ENSEMBL   UCSC 
 
  342
 
  5
 
 11807
 
 island
 
 0.284860
 
 0.0027557
 
 SLITRK5; MIR4500HG (margin: SLITRK5; MIR4500HG)
 
 12.9615703
 
 7.94e-03
 
 3.19e-02
  
 
  
 12
 
  30948636
 
  30948794
 
  PDF   STATS   ENSEMBL   UCSC 
 
  159
 
  5
 
 10308
 
 island
 
 0.007858
 
 0.9808008
 
 LINC00941 (margin: LINC00941)
 
 12.6474489
 
 7.94e-03
 
 3.19e-02
  
 
  
 7
 
  92672812
 
  92673176
 
  PDF   STATS   ENSEMBL   UCSC 
 
  365
 
  5
 
 38665
 
 island
 
 0.008107
 
 0.9600798
 
  (margin: )
 
 12.6220948
 
 7.94e-03
 
 3.19e-02
  
 
  
 6
 
 138893673
 
 138893718
 
  PDF   STATS   ENSEMBL   UCSC 
 
   46
 
  5
 
 35253
 
 tss
 
 0.985415
 
 0.0142395
 
 NHSL1 (margin: NHSL1)
 
 12.5105400
 
 7.94e-03
 
 3.19e-02
  
 
  
 14
 
  75593334
 
  75593738
 
  PDF   STATS   ENSEMBL   UCSC 
 
  405
 
  5
 
 12144
 
 gene
 
 0.005556
 
 0.9846942
 
 RP11-950C14.7; NEK9 (margin: RP11-950C14.7; NEK9; HIF1AP1; TMED10)
 
 12.4805559
 
 7.94e-03
 
 3.19e-02
  
 
  
 14
 
  69256677
 
  69256977
 
  PDF   STATS   ENSEMBL   UCSC 
 
  301
 
  5
 
 12121
 
 gene;island
 
 0.010901
 
 0.9786508
 
 ZFP36L1 (margin: ZFP36L1)
 
 12.3131729
 
 7.94e-03
 
 3.19e-02
  
 
  
 1
 
  25566331
 
  25566753
 
  PDF   STATS   ENSEMBL   UCSC 
 
  423
 
  5
 
 2868
 
 island
 
 0.002736
 
 0.9543479
 
 C1orf63 (margin: RP3-465N24.6; SYF2; C1orf63)
 
 12.1428432
 
 7.94e-03
 
 3.19e-02
  
 
  
 17
 
  79881468
 
  79881543
 
  PDF   STATS   ENSEMBL   UCSC 
 
   76
 
  5
 
 16929
 
 gene;tss;island
 
 0.009827
 
 0.9874887
 
 RP11-498C9.12; MAFG (margin: RP11-498C9.12; MAFG-AS1; RP11-498C9.13; SIRT7; MAFG; PYCR1)
 
 11.9669612
 
 7.94e-03
 
 3.19e-02
  
 
  
 2
 
  79220029
 
  79220528
 
  PDF   STATS   ENSEMBL   UCSC 
 
  500
 
  5
 
 24460
 
 island
 
 0.936069
 
 0.0110135
 
  (margin: )
 
 11.9542874
 
 7.94e-03
 
 3.19e-02
  
 
  
 8
 
 145025059
 
 145025178
 
  PDF   STATS   ENSEMBL   UCSC 
 
  120
 
  5
 
 39966
 
 tss
 
 0.007933
 
 0.9673927
 
 PLEC (margin: PLEC; MIR661)
 
 11.8683844
 
 7.94e-03
 
 3.19e-02
  
 
  
 5
 
 137225191
 
 137225509
 
  PDF   STATS   ENSEMBL   UCSC 
 
  319
 
  5
 
 31763
 
 gene;island
 
 0.002456
 
 0.9605017
 
 MYOT; PKD2L2; RP11-381K20.2 (margin: MYOT; PKD2L2; RP11-381K20.2)
 
 11.7597102
 
 7.94e-03
 
 3.19e-02
  
 
  
 7
 
  24796981
 
  24797486
 
  PDF   STATS   ENSEMBL   UCSC 
 
  506
 
  5
 
 36922
 
 gene
 
 0.008012
 
 0.9513217
 
 DFNA5 (margin: DFNA5)
 
 11.6414798
 
 7.94e-03
 
 3.19e-02
  
 
  
 2
 
 176994142
 
 176994448
 
  PDF   STATS   ENSEMBL   UCSC 
 
  307
 
  5
 
 23739
 
 tss
 
 0.012218
 
 0.9708036
 
 HOXD8; HOXD-AS2 (margin: HOXD10; HOXD9; HOXD8; HOXD3; HOXD-AS2)
 
 11.6172467
 
 7.94e-03
 
 3.19e-02
  
 
  
 1
 
 150254280
 
 150254546
 
  PDF   STATS   ENSEMBL   UCSC 
 
  267
 
  5
 
 1905
 
 tss;island
 
 0.879954
 
 0.0050452
 
 C1orf54; C1orf51 (margin: C1orf54; C1orf51; MRPS21)
 
 11.6120245
 
 7.94e-03
 
 3.19e-02
  
 
  
 11
 
    796361
 
    796607
 
  PDF   STATS   ENSEMBL   UCSC 
 
  247
 
  5
 
 7028
 
 tss;island
 
 0.007354
 
 0.9589390
 
 SLC25A22 (margin: CEND1; SLC25A22; PIDD)
 
 11.5819908
 
 7.94e-03
 
 3.19e-02
  
 
  
 22
 
  50968250
 
  50968343
 
  PDF   STATS   ENSEMBL   UCSC 
 
   94
 
  5
 
 26931
 
 gene
 
 0.012995
 
 0.9769053
 
 TYMP; ODF3B (margin: NCAPH2; SCO2; TYMP; ODF3B)
 
 11.5426179
 
 7.94e-03
 
 3.19e-02
  
 
  
 22
 
  50968297
 
  50968516
 
  PDF   STATS   ENSEMBL   UCSC 
 
  220
 
  5
 
 27258
 
 tss
 
 0.012080
 
 0.9769053
 
 TYMP; ODF3B (margin: NCAPH2; SCO2; TYMP; ODF3B)
 
 11.5426179
 
 7.94e-03
 
 3.19e-02
  
 
  
 6
 
 105584551
 
 105584780
 
  PDF   STATS   ENSEMBL   UCSC 
 
  230
 
  5
 
 35145
 
 tss;island
 
 0.013436
 
 0.9670871
 
 BVES-AS1; BVES (margin: BVES-AS1; BVES)
 
 11.5387936
 
 7.94e-03
 
 3.19e-02
  
 
  
 3
 
  11178593
 
  11178758
 
  PDF   STATS   ENSEMBL   UCSC 
 
  166
 
  5
 
 28154
 
 tss
 
 0.004118
 
 0.8822373
 
 HRH1 (margin: HRH1)
 
 11.5308468
 
 7.94e-03
 
 3.19e-02
  
 
  
 1
 
  21616619
 
  21617100
 
  PDF   STATS   ENSEMBL   UCSC 
 
  482
 
  5
 
 295
 
 gene;island
 
 0.012588
 
 0.9767680
 
 ECE1 (margin: RP5-1071N3.1; ECE1)
 
 11.3397599
 
 7.94e-03
 
 3.19e-02
  
 
  
 7
 
  48129797
 
  48129992
 
  PDF   STATS   ENSEMBL   UCSC 
 
  196
 
  5
 
 37006
 
 gene;island
 
 0.005170
 
 0.9258725
 
 UPP1 (margin: UPP1)
 
 11.3206262
 
 7.94e-03
 
 3.19e-02
  
 
  
 4
 
 165877875
 
 165878091
 
  PDF   STATS   ENSEMBL   UCSC 
 
  217
 
  5
 
 30665
 
 tss
 
 0.976683
 
 0.0332061
 
 RP11-366M4.8; FAM218A; TRIM61 (margin: RP11-366M4.8; FAM218A; TRIM61; RP11-366M4.11)
 
 11.3094782
 
 7.94e-03
 
 3.19e-02
  
 
  
 6
 
 106546704
 
 106546824
 
  PDF   STATS   ENSEMBL   UCSC 
 
  121
 
  5
 
 34434
 
 gene
 
 0.004456
 
 0.9651344
 
 PRDM1; RP1-134E15.3 (margin: PRDM1; RP1-134E15.3)
 
 11.2935180
 
 7.94e-03
 
 3.19e-02
  
 
  
 5
 
 140207460
 
 140207636
 
  PDF   STATS   ENSEMBL   UCSC 
 
  177
 
  5
 
 32352
 
 tss
 
 0.957192
 
 0.0137229
 
 PCDHA1; PCDHA2; PCDHA3; PCDHA4; PCDHA5; PCDHA6 (margin: PCDHA1; PCDHA2; PCDHA3; PCDHA4; PCDHA5; PCDHA6; PCDHA7)
 
 11.2935011
 
 7.94e-03
 
 3.19e-02
  
 
  
 6
 
  27840957
 
  27841230
 
  PDF   STATS   ENSEMBL   UCSC 
 
  274
 
  5
 
 33624
 
 gene
 
 0.047288
 
 0.9780759
 
 HIST1H3I; HIST1H4L (margin: HIST1H2AL; HIST1H2BPS2; HIST1H1B; HIST1H3I; HIST1H4L)
 
 11.2060771
 
 7.94e-03
 
 3.19e-02
  
 
  
 15
 
  84047906
 
  84048308
 
  PDF   STATS   ENSEMBL   UCSC 
 
  403
 
  5
 
 14399
 
 island
 
 0.007696
 
 0.9650349
 
 RP11-382A20.4 (margin: RP11-382A20.4)
 
 11.1948298
 
 7.94e-03
 
 3.19e-02
  
 
  
 19
 
    571107
 
    571416
 
  PDF   STATS   ENSEMBL   UCSC 
 
  310
 
  5
 
 20274
 
 tss;island
 
 0.908118
 
 0.0094937
 
 BSG; AC009005.2 (margin: BSG; AC009005.2)
 
 11.1124610
 
 7.94e-03
 
 3.19e-02
  
 
  
 11
 
  63655748
 
  63656090
 
  PDF   STATS   ENSEMBL   UCSC 
 
  343
 
  5
 
 6655
 
 gene
 
 0.009720
 
 0.9273905
 
 MARK2 (margin: MARK2; RNU6-1306P)
 
 11.0398515
 
 7.94e-03
 
 3.19e-02
  
 
  
 10
 
 118031864
 
 118032081
 
  PDF   STATS   ENSEMBL   UCSC 
 
  218
 
  5
 
 4773
 
 gene;tss;island
 
 0.817448
 
 0.0118907
 
 GFRA1 (margin: GFRA1)
 
 11.0288695
 
 7.94e-03
 
 3.19e-02
  
 
  
 8
 
  67454546
 
  67454892
 
  PDF   STATS   ENSEMBL   UCSC 
 
  347
 
  5
 
 40356
 
 island
 
 0.963815
 
 0.0132727
 
  (margin: )
 
 10.9929856
 
 7.94e-03
 
 3.19e-02
  
 
  
 7
 
  87257356
 
  87257673
 
  PDF   STATS   ENSEMBL   UCSC 
 
  318
 
  5
 
 37687
 
 tss
 
 0.858652
 
 0.0092591
 
 RUNDC3B; ABCB1 (margin: snoU13; RUNDC3B; ABCB1)
 
 10.9893948
 
 7.94e-03
 
 3.19e-02
  
 
  
 6
 
 152011103
 
 152011415
 
  PDF   STATS   ENSEMBL   UCSC 
 
  313
 
  5
 
 35302
 
 tss
 
 0.900167
 
 0.0099869
 
 ESR1 (margin: ESR1)
 
 10.9327970
 
 7.94e-03
 
 3.19e-02
  
 
  
 17
 
   4981403
 
   4981610
 
  PDF   STATS   ENSEMBL   UCSC 
 
  208
 
  5
 
 17040
 
 tss;island
 
 0.908852
 
 0.0062884
 
 ZFP3; RP11-46I8.3 (margin: ZFP3; RP11-46I8.3)
 
 10.8578355
 
 7.94e-03
 
 3.19e-02
  
 
  
 4
 
 158141449
 
 158141570
 
  PDF   STATS   ENSEMBL   UCSC 
 
  122
 
  5
 
 30654
 
 tss;island
 
 0.979948
 
 0.0299280
 
 GRIA2 (margin: GRIA2)
 
 10.8357062
 
 7.94e-03
 
 3.19e-02
  
 
  
 3
 
  11610138
 
  11610338
 
  PDF   STATS   ENSEMBL   UCSC 
 
  201
 
  5
 
 27668
 
 gene;island
 
 0.009809
 
 0.9756045
 
 VGLL4 (margin: ATG7; VGLL4)
 
 10.7703361
 
 7.94e-03
 
 3.19e-02
  
 
  
 15
 
  83952722
 
  83953068
 
  PDF   STATS   ENSEMBL   UCSC 
 
  347
 
  5
 
 13459
 
 gene;island
 
 0.025098
 
 0.9797996
 
 RP11-382A20.4; BNC1 (margin: RP11-382A20.4; BNC1)
 
 10.7559138
 
 7.94e-03
 
 3.19e-02
  
 
  
 13
 
  20392406
 
  20392706
 
  PDF   STATS   ENSEMBL   UCSC 
 
  301
 
  5
 
 11562
 
 island
 
 0.978530
 
 0.0299560
 
 ST6GALNAC4P1 (margin: ST6GALNAC4P1; ZMYM5)
 
 10.5817086
 
 7.94e-03
 
 3.19e-02
  
 
  
 19
 
  40314862
 
  40315143
 
  PDF   STATS   ENSEMBL   UCSC 
 
  282
 
  5
 
 22005
 
 island
 
 0.982789
 
 0.0502869
 
 DYRK1B (margin: DYRK1B; FBL)
 
 10.5758439
 
 7.94e-03
 
 3.19e-02
  
 
  
 7
 
  83277941
 
  83278138
 
  PDF   STATS   ENSEMBL   UCSC 
 
  198
 
  5
 
 37068
 
 gene
 
 0.776585
 
 0.0027332
 
 SEMA3E (margin: SEMA3E)
 
 10.4913903
 
 7.94e-03
 
 3.19e-02
  
 
  
 1
 
  51810823
 
  51811185
 
  PDF   STATS   ENSEMBL   UCSC 
 
  363
 
  5
 
 1622
 
 tss
 
 0.030217
 
 0.9757689
 
 TTC39A (margin: RP11-275F13.1; TTC39A; EPS15)
 
 10.3757618
 
 7.94e-03
 
 3.19e-02
  
 
  
 4
 
 186456837
 
 186457012
 
  PDF   STATS   ENSEMBL   UCSC 
 
  176
 
  5
 
 30702
 
 tss
 
 0.925963
 
 0.0110995
 
 PDLIM3 (margin: PDLIM3)
 
 10.2807657
 
 7.94e-03
 
 3.19e-02
  
 
  
 17
 
  72855943
 
  72856181
 
  PDF   STATS   ENSEMBL   UCSC 
 
  239
 
  5
 
 18726
 
 island
 
 0.045745
 
 0.9905716
 
 GRIN2C (margin: GRIN2C; FDXR)
 
 10.1395251
 
 7.94e-03
 
 3.19e-02
  
 
  
 19
 
  49223814
 
  49224165
 
  PDF   STATS   ENSEMBL   UCSC 
 
  352
 
  5
 
 20925
 
 tss;island
 
 0.987176
 
 0.0348285
 
 MAMSTR; RASIP1 (margin: MAMSTR; RASIP1)
 
 10.1038203
 
 7.94e-03
 
 3.19e-02
  
 
  
 11
 
  10476494
 
  10476662
 
  PDF   STATS   ENSEMBL   UCSC 
 
  169
 
  5
 
 6506
 
 gene;tss;island
 
 0.009191
 
 0.8970407
 
 AMPD3 (margin: AMPD3)
 
 10.0901623
 
 7.94e-03
 
 3.19e-02
  
 
  
 3
 
  72704324
 
  72704701
 
  PDF   STATS   ENSEMBL   UCSC 
 
  378
 
  5
 
 29281
 
 island
 
 0.938345
 
 0.0110333
 
  (margin: )
 
 10.0556694
 
 7.94e-03
 
 3.19e-02
  
 
  
 1
 
  21877524
 
  21877781
 
  PDF   STATS   ENSEMBL   UCSC 
 
  258
 
  5
 
 298
 
 gene;tss
 
 0.973677
 
 0.0258809
 
 ALPL (margin: ALPL)
 
 10.0345739
 
 7.94e-03
 
 3.19e-02
  
 
  
 7
 
  25019747
 
  25019943
 
  PDF   STATS   ENSEMBL   UCSC 
 
  197
 
  5
 
 38320
 
 island
 
 0.019260
 
 0.9589232
 
 OSBPL3 (margin: OSBPL3)
 
  9.9827205
 
 7.94e-03
 
 3.19e-02
  
 
  
 1
 
 228225533
 
 228225886
 
  PDF   STATS   ENSEMBL   UCSC 
 
  354
 
  5
 
 1045
 
 gene;island
 
 0.047340
 
 0.9784386
 
 WNT3A (margin: WNT3A)
 
  9.9641901
 
 7.94e-03
 
 3.19e-02
  
 
  
 3
 
 141087187
 
 141087363
 
  PDF   STATS   ENSEMBL   UCSC 
 
  177
 
  5
 
 27973
 
 gene
 
 0.007575
 
 0.8122344
 
 ZBTB38; RP11-438D8.2 (margin: ZBTB38; RP11-438D8.2)
 
  9.7822798
 
 7.94e-03
 
 3.19e-02
  
 
  
 16
 
   3096477
 
   3096662
 
  PDF   STATS   ENSEMBL   UCSC 
 
  186
 
  5
 
 15049
 
 tss
 
 0.033886
 
 0.9067693
 
 MMP25 (margin: RP11-473M20.5; MMP25; CCDC64B; RP11-473M20.7)
 
  9.7740635
 
 7.94e-03
 
 3.19e-02
  
 
  
 19
 
  18335182
 
  18335429
 
  PDF   STATS   ENSEMBL   UCSC 
 
  248
 
  5
 
 19931
 
 gene;island
 
 0.637108
 
 0.0029624
 
 PDE4C (margin: AC068499.10; PDE4C)
 
  9.7111723
 
 7.94e-03
 
 3.19e-02
  
 
  
 5
 
  89854632
 
  89854979
 
  PDF   STATS   ENSEMBL   UCSC 
 
  348
 
  5
 
 31687
 
 gene
 
 0.918695
 
 0.0119135
 
 GPR98 (margin: GPR98)
 
  9.7010737
 
 7.94e-03
 
 3.19e-02
  
 
  
 19
 
  50832651
 
  50832861
 
  PDF   STATS   ENSEMBL   UCSC 
 
  211
 
  5
 
 20974
 
 tss
 
 0.788778
 
 0.0168283
 
 NR1H2; KCNC3 (margin: NR1H2; KCNC3; NAPSB)
 
  9.6859521
 
 7.94e-03
 
 3.19e-02
  
 
  
 21
 
  42733729
 
  42733894
 
  PDF   STATS   ENSEMBL   UCSC 
 
  166
 
  5
 
 26556
 
 tss
 
 0.022285
 
 0.8428770
 
 MX2 (margin: FAM3B; MX2)
 
  9.6639145
 
 7.94e-03
 
 3.19e-02
  
 
  
 11
 
  13689411
 
  13689686
 
  PDF   STATS   ENSEMBL   UCSC 
 
  276
 
  5
 
 7158
 
 tss;island
 
 0.411502
 
 0.0115158
 
 FAR1; FAR1-IT1 (margin: FAR1; FAR1-IT1)
 
  9.6463176
 
 7.94e-03
 
 3.19e-02
  
 
  
 17
 
  75315486
 
  75315567
 
  PDF   STATS   ENSEMBL   UCSC 
 
   82
 
  5
 
 17696
 
 tss
 
 0.014663
 
 0.8518537
 
 SEPT9 (margin: SEPT9)
 
  9.6363617
 
 7.94e-03
 
 3.19e-02
  
 
  
 12
 
 106533840
 
 106533903
 
  PDF   STATS   ENSEMBL   UCSC 
 
   64
 
  5
 
 9966
 
 tss
 
 0.058686
 
 0.9796691
 
 NUAK1 (margin: NUAK1)
 
  9.6315270
 
 7.94e-03
 
 3.19e-02
  
 
  
 11
 
 124587993
 
 124588484
 
  PDF   STATS   ENSEMBL   UCSC 
 
  492
 
  5
 
 8914
 
 island
 
 0.010895
 
 0.9668032
 
  (margin: )
 
  9.6081672
 
 7.94e-03
 
 3.19e-02
  
 
  
 20
 
  52199520
 
  52199778
 
  PDF   STATS   ENSEMBL   UCSC 
 
  259
 
  5
 
 26263
 
 island
 
 0.004588
 
 0.8533248
 
 ZNF217 (margin: RP4-724E16.2; ZNF217)
 
  9.5739538
 
 7.94e-03
 
 3.19e-02
  
 
  
 1
 
  43613433
 
  43613567
 
  PDF   STATS   ENSEMBL   UCSC 
 
  135
 
  5
 
 1556
 
 tss
 
 0.917510
 
 0.0291883
 
 FAM183A (margin: FAM183A)
 
  9.5629223
 
 7.94e-03
 
 3.19e-02
  
 
  
 18
 
  53257019
 
  53257340
 
  PDF   STATS   ENSEMBL   UCSC 
 
  322
 
  5
 
 19286
 
 tss;island
 
 0.042610
 
 0.9631121
 
 TCF4 (margin: TCF4)
 
  9.5585631
 
 7.94e-03
 
 3.19e-02
  
 
  
 1
 
  24126017
 
  24126230
 
  PDF   STATS   ENSEMBL   UCSC 
 
  214
 
  5
 
 320
 
 gene;island
 
 0.004854
 
 0.6858252
 
 GALE (margin: PITHD1; LYPLA2; GALE; HMGCL)
 
  9.5277472
 
 7.94e-03
 
 3.19e-02
  
 
  
 2
 
  69664772
 
  69664869
 
  PDF   STATS   ENSEMBL   UCSC 
 
   98
 
  5
 
 23406
 
 tss
 
 0.003137
 
 0.5771487
 
 NFU1 (margin: NFU1)
 
  9.4920539
 
 7.94e-03
 
 3.19e-02
  
 
  
 1
 
   2063799
 
   2064216
 
  PDF   STATS   ENSEMBL   UCSC 
 
  418
 
  5
 
 63
 
 gene;island
 
 0.028678
 
 0.9758133
 
 PRKCZ (margin: PRKCZ; RP5-892K4.1)
 
  9.4656822
 
 7.94e-03
 
 3.19e-02
  
 
  
 1
 
   3615390
 
   3615762
 
  PDF   STATS   ENSEMBL   UCSC 
 
  373
 
  5
 
 139
 
 gene
 
 0.096132
 
 0.9855319
 
 TP73 (margin: TP73)
 
  9.4420171
 
 7.94e-03
 
 3.19e-02
  
 
  
 1
 
 190447232
 
 190447640
 
  PDF   STATS   ENSEMBL   UCSC 
 
  409
 
  5
 
 2147
 
 tss
 
 0.460310
 
 0.0100990
 
 RP11-547I7.2; RP11-161I10.1; FAM5C (margin: RP11-547I7.2; RP11-161I10.1; FAM5C)
 
  9.3454254
 
 7.94e-03
 
 3.19e-02
  
 
  
 1
 
 202183264
 
 202183605
 
  PDF   STATS   ENSEMBL   UCSC 
 
  342
 
  5
 
 945
 
 gene;island
 
 0.046696
 
 0.9606753
 
 LGR6 (margin: LGR6)
 
  9.3215628
 
 7.94e-03
 
 3.19e-02
  
 
  
 1
 
 207224090
 
 207224388
 
  PDF   STATS   ENSEMBL   UCSC 
 
  299
 
  5
 
 975
 
 gene
 
 0.007758
 
 0.9580892
 
 PFKFB2; YOD1 (margin: PFKFB2; snoU13; YOD1)
 
  9.2884870
 
 7.94e-03
 
 3.19e-02
  
 
  
 18
 
  59560365
 
  59560495
 
  PDF   STATS   ENSEMBL   UCSC 
 
  131
 
  5
 
 19301
 
 tss
 
 0.762207
 
 0.0053392
 
 RNF152 (margin: RNF152)
 
  9.2459805
 
 7.94e-03
 
 3.19e-02
  
 
  
 12
 
   6560689
 
   6560851
 
  PDF   STATS   ENSEMBL   UCSC 
 
  163
 
  5
 
 9028
 
 gene
 
 0.015785
 
 0.8990481
 
 CD27; TAPBPL; CD27-AS1 (margin: CD27; TAPBPL; CD27-AS1; VAMP1)
 
  9.2012007
 
 7.94e-03
 
 3.19e-02
  
 
  
 2
 
  97171137
 
  97171449
 
  PDF   STATS   ENSEMBL   UCSC 
 
  313
 
  5
 
 22781
 
 gene;island
 
 0.004061
 
 0.6976759
 
 NEURL3 (margin: NEURL3)
 
  9.1609648
 
 7.94e-03
 
 3.19e-02
  
 
  
 12
 
  54388841
 
  54389264
 
  PDF   STATS   ENSEMBL   UCSC 
 
  424
 
  5
 
 10488
 
 island
 
 0.024892
 
 0.9694346
 
 HOXC5; HOXC6; HOXC9; HOXC-AS2 (margin: HOXC10; HOXC5; HOXC6; MIR196A2; HOXC9; HOXC-AS3; HOXC-AS2; HOXC-AS1)
 
  9.1121318
 
 7.94e-03
 
 3.19e-02
  
 
  
 2
 
  11679845
 
  11680057
 
  PDF   STATS   ENSEMBL   UCSC 
 
  213
 
  5
 
 23191
 
 tss
 
 0.588034
 
 0.0093264
 
 GREB1; MIR4429 (margin: GREB1; MIR4429)
 
  8.9423812
 
 7.94e-03
 
 3.19e-02
  
 
  
 2
 
 238395764
 
 238395947
 
  PDF   STATS   ENSEMBL   UCSC 
 
  184
 
  5
 
 25230
 
 island
 
 0.772013
 
 0.0084101
 
 MLPH (margin: MLPH)
 
  8.9420423
 
 7.94e-03
 
 3.19e-02
  
 
  
 17
 
  41446167
 
  41446521
 
  PDF   STATS   ENSEMBL   UCSC 
 
  355
 
  5
 
 18369
 
 island
 
 0.004838
 
 0.5319861
 
 LINC00910 (margin: LINC00910)
 
  8.9112644
 
 7.94e-03
 
 3.19e-02
  
 
  
 19
 
   1401118
 
   1401492
 
  PDF   STATS   ENSEMBL   UCSC 
 
  375
 
  5
 
 19654
 
 gene
 
 0.675243
 
 0.0048163
 
 GAMT (margin: NDUFS7; DAZAP1; AC005329.7; GAMT)
 
  8.8673237
 
 7.94e-03
 
 3.19e-02
  
 
  
 6
 
 166400871
 
 166401051
 
  PDF   STATS   ENSEMBL   UCSC 
 
  181
 
  5
 
 34541
 
 gene;island
 
 0.457473
 
 0.0033101
 
 PDE10A; LINC00473 (margin: PDE10A; LINC00473)
 
  8.8550786
 
 7.94e-03
 
 3.19e-02
  
 
  
 5
 
 118690955
 
 118691126
 
  PDF   STATS   ENSEMBL   UCSC 
 
  172
 
  5
 
 31724
 
 gene;tss;island
 
 0.004970
 
 0.8208736
 
 TNFAIP8 (margin: TNFAIP8)
 
  8.8065075
 
 7.94e-03
 
 3.19e-02
  
 
  
 19
 
  49127327
 
  49127660
 
  PDF   STATS   ENSEMBL   UCSC 
 
  334
 
  5
 
 20132
 
 gene;island
 
 0.959783
 
 0.0172842
 
 SPHK2; AC022154.7 (margin: SPHK2; FAM83E; RPL18; AC022154.7; DBP)
 
  8.8050989
 
 7.94e-03
 
 3.19e-02
  
 
  
 17
 
  78161467
 
  78161572
 
  PDF   STATS   ENSEMBL   UCSC 
 
  106
 
  5
 
 17722
 
 tss
 
 0.011574
 
 0.8648938
 
 CARD14 (margin: CARD14)
 
  8.7593436
 
 7.94e-03
 
 3.19e-02
  
 
  
 3
 
 157824148
 
 157824283
 
  PDF   STATS   ENSEMBL   UCSC 
 
  136
 
  5
 
 28682
 
 tss;island
 
 0.336309
 
 0.0021935
 
 RSRC1; SHOX2 (margin: RSRC1; SHOX2)
 
  8.7477536
 
 7.94e-03
 
 3.19e-02
  
 
  
 4
 
 156297854
 
 156298050
 
  PDF   STATS   ENSEMBL   UCSC 
 
  197
 
  5
 
 30158
 
 gene
 
 0.860690
 
 0.0156267
 
 AC097467.2; MAP9 (margin: AC097467.2; RP11-27G13.5; MAP9; RP11-27G13.3; RP11-27G13.4)
 
  8.7155901
 
 7.94e-03
 
 3.19e-02
  
 
  
 20
 
   3220565
 
   3220985
 
  PDF   STATS   ENSEMBL   UCSC 
 
  421
 
  5
 
 25969
 
 island
 
 0.040605
 
 0.9564412
 
 SLC4A11 (margin: SLC4A11; C20orf194)
 
  8.6870427
 
 7.94e-03
 
 3.19e-02
  
 
  
 6
 
  30652647
 
  30652907
 
  PDF   STATS   ENSEMBL   UCSC 
 
  261
 
  5
 
 33803
 
 gene;island
 
 0.083283
 
 0.9679755
 
 PPP1R18 (margin: DHX16; PPP1R18; NRM; RPL7P4)
 
  8.6819705
 
 7.94e-03
 
 3.19e-02
  
 
  
 20
 
  44540794
 
  44541153
 
  PDF   STATS   ENSEMBL   UCSC 
 
  360
 
  5
 
 25783
 
 tss
 
 0.192369
 
 0.9883776
 
 PLTP (margin: PLTP)
 
  8.6612073
 
 7.94e-03
 
 3.19e-02
  
 
  
 7
 
 107205042
 
 107205392
 
  PDF   STATS   ENSEMBL   UCSC 
 
  351
 
  5
 
 37180
 
 gene;tss;island
 
 0.003187
 
 0.6730959
 
 DUS4L; COG5 (margin: DUS4L; COG5)
 
  8.6424122
 
 7.94e-03
 
 3.19e-02
  
 
  
 17
 
   8771003
 
   8771331
 
  PDF   STATS   ENSEMBL   UCSC 
 
  329
 
  5
 
 17123
 
 tss
 
 0.008925
 
 0.5408919
 
 PIK3R6 (margin: PIK3R6; PIK3R5)
 
  8.6153141
 
 7.94e-03
 
 3.19e-02
  
 
  
 13
 
  88324432
 
  88324879
 
  PDF   STATS   ENSEMBL   UCSC 
 
  448
 
  5
 
 11809
 
 island
 
 0.867562
 
 0.0211597
 
 SLITRK5; MIR4500HG (margin: SLITRK5; MIR4500HG)
 
  8.6146314
 
 7.94e-03
 
 3.19e-02
  
 
  
 1
 
 159046773
 
 159047163
 
  PDF   STATS   ENSEMBL   UCSC 
 
  391
 
  5
 
 2028
 
 tss
 
 0.018595
 
 0.8773162
 
 AIM2 (margin: AIM2; RP11-520H16.4)
 
  8.5790774
 
 7.94e-03
 
 3.19e-02
  
 
  
 16
 
  69141250
 
  69141437
 
  PDF   STATS   ENSEMBL   UCSC 
 
  188
 
  5
 
 15326
 
 tss
 
 0.106971
 
 0.9779223
 
 HAS3 (margin: HAS3; CHTF8)
 
  8.5378020
 
 7.94e-03
 
 3.19e-02
  
 
  
 19
 
   2427708
 
   2428122
 
  PDF   STATS   ENSEMBL   UCSC 
 
  415
 
  5
 
 21284
 
 island
 
 0.008211
 
 0.5132677
 
 TMPRSS9; TIMM13; LMNB2 (margin: TMPRSS9; TIMM13; LMNB2)
 
  8.5147865
 
 7.94e-03
 
 3.19e-02
  
 
  
 19
 
  31640642
 
  31640711
 
  PDF   STATS   ENSEMBL   UCSC 
 
   70
 
  5
 
 20673
 
 tss
 
 0.178352
 
 0.9840156
 
 AC020952.1 (margin: AC020952.1)
 
  8.5078910
 
 7.94e-03
 
 3.19e-02
  
 
  
 2
 
 119603383
 
 119603969
 
  PDF   STATS   ENSEMBL   UCSC 
 
  587
 
  5
 
 22841
 
 gene;island
 
 0.059361
 
 0.9766843
 
 EN1 (margin: RP11-19E11.1; EN1)
 
  8.4283894
 
 7.94e-03
 
 3.19e-02
  
 
  
 5
 
  63802106
 
  63802412
 
  PDF   STATS   ENSEMBL   UCSC 
 
  307
 
  5
 
 32089
 
 tss
 
 0.906224
 
 0.0213607
 
 RGS7BP (margin: RGS7BP)
 
  8.4164990
 
 7.94e-03
 
 3.19e-02
  
 
  
 3
 
 113251061
 
 113251417
 
  PDF   STATS   ENSEMBL   UCSC 
 
  357
 
  5
 
 29366
 
 island
 
 0.109499
 
 0.0009064
 
 SIDT1 (margin: SIDT1)
 
  8.3783860
 
 7.94e-03
 
 3.19e-02
  
 
  
 1
 
   8271918
 
   8272277
 
  PDF   STATS   ENSEMBL   UCSC 
 
  360
 
  5
 
 2634
 
 island
 
 0.008565
 
 0.6228818
 
 RP11-431K24.3 (margin: RP11-431K24.3; RNU1-7P; RP11-431K24.4)
 
  8.3663883
 
 7.94e-03
 
 3.19e-02
  
 
  
 6
 
  39281421
 
  39281885
 
  PDF   STATS   ENSEMBL   UCSC 
 
  465
 
  5
 
 34325
 
 gene;island
 
 0.090577
 
 0.9702917
 
 KCNK17; KCNK16 (margin: KCNK17; KCNK16)
 
  8.3585339
 
 7.94e-03
 
 3.19e-02
  
 
  
 14
 
  61116211
 
  61116506
 
  PDF   STATS   ENSEMBL   UCSC 
 
  296
 
  5
 
 12439
 
 tss
 
 0.004213
 
 0.5544231
 
 SIX1 (margin: RP11-1042B17.5; SIX1)
 
  8.3524061
 
 7.94e-03
 
 3.19e-02
  
 
  
 8
 
  13424080
 
  13424269
 
  PDF   STATS   ENSEMBL   UCSC 
 
  190
 
  5
 
 39561
 
 tss
 
 0.481329
 
 0.0043589
 
 C8orf48; RP11-145O15.3 (margin: C8orf48; RP11-145O15.3)
 
  8.3439752
 
 7.94e-03
 
 3.19e-02
  
 
  
 11
 
  64014518
 
  64014598
 
  PDF   STATS   ENSEMBL   UCSC 
 
   81
 
  5
 
 7390
 
 tss;island
 
 0.461853
 
 0.0132093
 
 RP11-783K16.5; PPP1R14B; RP11-783K16.13 (margin: VEGFB; FKBP2; RP11-783K16.5; PLCB3; PPP1R14B; RP11-783K16.13)
 
  8.3350929
 
 7.94e-03
 
 3.19e-02
  
 
  
 11
 
   2170870
 
   2171184
 
  PDF   STATS   ENSEMBL   UCSC 
 
  315
 
  5
 
 7059
 
 tss
 
 0.031741
 
 0.7533327
 
 IGF2-AS; IGF2; INS-IGF2 (margin: IGF2-AS; IGF2; INS-IGF2; INS)
 
  8.3298164
 
 7.94e-03
 
 3.19e-02
  
 
  
 13
 
  53425635
 
  53426032
 
  PDF   STATS   ENSEMBL   UCSC 
 
  398
 
  5
 
 11764
 
 island
 
 0.386905
 
 0.0015809
 
  (margin: PCDH8)
 
  8.2799760
 
 7.94e-03
 
 3.19e-02
  
 
  
 1
 
  31158026
 
  31158299
 
  PDF   STATS   ENSEMBL   UCSC 
 
  274
 
  5
 
 2964
 
 island
 
 0.875772
 
 0.0235539
 
  (margin: )
 
  8.2517766
 
 7.94e-03
 
 3.19e-02
  
 
  
 1
 
 109941043
 
 109941201
 
  PDF   STATS   ENSEMBL   UCSC 
 
  159
 
  5
 
 1802
 
 tss;island
 
 0.946062
 
 0.0376738
 
 SORT1; PSMA5 (margin: SORT1; PSMA5)
 
  8.2352069
 
 7.94e-03
 
 3.19e-02
  
 
  
 6
 
  87647370
 
  87647644
 
  PDF   STATS   ENSEMBL   UCSC 
 
  275
 
  5
 
 34408
 
 gene;island
 
 0.926751
 
 0.0153160
 
 HTR1E (margin: HTR1E)
 
  8.2130131
 
 7.94e-03
 
 3.19e-02
  
 
  
 1
 
   2222253
 
   2222674
 
  PDF   STATS   ENSEMBL   UCSC 
 
  422
 
  5
 
 72
 
 gene;island
 
 0.114408
 
 0.9540614
 
 SKI (margin: SKI)
 
  8.1975602
 
 7.94e-03
 
 3.19e-02
  
 
  
 17
 
  78800574
 
  78800806
 
  PDF   STATS   ENSEMBL   UCSC 
 
  233
 
  5
 
 16875
 
 gene;island
 
 0.109899
 
 0.9726206
 
 RPTOR (margin: RPTOR)
 
  8.1913743
 
 7.94e-03
 
 3.19e-02
  
 
  
 1
 
  32714023
 
  32714308
 
  PDF   STATS   ENSEMBL   UCSC 
 
  286
 
  5
 
 392
 
 gene;island
 
 0.871854
 
 0.0284450
 
 FAM167B (margin: FAM167B; LCK; MTMR9LP)
 
  8.1811033
 
 7.94e-03
 
 3.19e-02
  
 
  
 1
 
  82267923
 
  82268281
 
  PDF   STATS   ENSEMBL   UCSC 
 
  359
 
  5
 
 597
 
 gene;island
 
 0.479268
 
 0.0075037
 
 LPHN2 (margin: LPHN2)
 
  8.1717529
 
 7.94e-03
 
 3.19e-02
  
 
  
 17
 
  80806135
 
  80806435
 
  PDF   STATS   ENSEMBL   UCSC 
 
  301
 
  5
 
 16960
 
 gene
 
 0.078442
 
 0.9685223
 
 TBCD (margin: TBCD; ZNF750)
 
  8.1654564
 
 7.94e-03
 
 3.19e-02
  
 
  
 19
 
  50194120
 
  50194252
 
  PDF   STATS   ENSEMBL   UCSC 
 
  133
 
  5
 
 20961
 
 tss;island
 
 0.202623
 
 0.9772568
 
 PRMT1; ADM5; CPT1C; CTB-33G10.6 (margin: PRMT1; MIR5088; ADM5; CPT1C; CTB-33G10.6)
 
  8.1077477
 
 7.94e-03
 
 3.19e-02
  
 
  
 11
 
 124311131
 
 124311423
 
  PDF   STATS   ENSEMBL   UCSC 
 
  293
 
  5
 
 7782
 
 tss
 
 0.116300
 
 0.9788297
 
 OR8B8 (margin: OR8B8)
 
  8.0792415
 
 7.94e-03
 
 3.19e-02
  
 
  
 11
 
 120530774
 
 120530973
 
  PDF   STATS   ENSEMBL   UCSC 
 
  200
 
  5
 
 7764
 
 tss
 
 0.046716
 
 0.9359603
 
 GRIK4 (margin: GRIK4)
 
  8.0635414
 
 7.94e-03
 
 3.19e-02
  
 
  
 15
 
  99789622
 
  99789855
 
  PDF   STATS   ENSEMBL   UCSC 
 
  234
 
  5
 
 14503
 
 island
 
 0.003573
 
 0.4260381
 
 LRRC28; TTC23 (margin: LRRC28; AC022819.1; HSP90B2P; TTC23)
 
  8.0453028
 
 7.94e-03
 
 3.19e-02
  
 
  
 11
 
    818752
 
    818917
 
  PDF   STATS   ENSEMBL   UCSC 
 
  166
 
  5
 
 7884
 
 island
 
 0.017277
 
 0.5443000
 
 PNPLA2 (margin: RPLP2; SNORA52; PNPLA2; EFCAB4A; PIDD; AP006621.8)
 
  8.0192970
 
 7.94e-03
 
 3.19e-02
  
 
  
 19
 
  11531294
 
  11531519
 
  PDF   STATS   ENSEMBL   UCSC 
 
  226
 
  5
 
 19840
 
 gene;island
 
 0.816227
 
 0.0265451
 
 RGL3; CCDC151 (margin: Y_RNA; RGL3; CCDC151)
 
  7.9674074
 
 7.94e-03
 
 3.19e-02
  
 
  
 5
 
 128796079
 
 128796266
 
  PDF   STATS   ENSEMBL   UCSC 
 
  188
 
  5
 
 33074
 
 island
 
 0.567654
 
 0.0109427
 
 ADAMTS19; ADAMTS19-AS1 (margin: ADAMTS19; ADAMTS19-AS1)
 
  7.9347361
 
 7.94e-03
 
 3.19e-02
  
 
  
 3
 
 187456175
 
 187456579
 
  PDF   STATS   ENSEMBL   UCSC 
 
  405
 
  5
 
 28078
 
 gene;island
 
 0.156983
 
 0.9880829
 
 BCL6 (margin: RP11-211G3.3; RP11-211G3.2; BCL6)
 
  7.9224759
 
 7.94e-03
 
 3.19e-02
  
 
  
 17
 
   6899522
 
   6899888
 
  PDF   STATS   ENSEMBL   UCSC 
 
  367
 
  5
 
 16380
 
 gene
 
 0.240198
 
 0.9779467
 
 ALOX12; AC027763.2; RP11-589P10.7; RP11-589P10.5 (margin: ALOX12; AC027763.2; RP11-589P10.7; RP11-589P10.5)
 
  7.8572518
 
 7.94e-03
 
 3.19e-02
  
 
  
 3
 
  46448753
 
  46449100
 
  PDF   STATS   ENSEMBL   UCSC 
 
  348
 
  5
 
 27748
 
 gene
 
 0.021420
 
 0.8615799
 
 ACKR5; RP11-24F11.2 (margin: ACKR5; RP11-24F11.2)
 
  7.8486509
 
 7.94e-03
 
 3.19e-02
  
 
  
 1
 
  61542546
 
  61542689
 
  PDF   STATS   ENSEMBL   UCSC 
 
  144
 
  5
 
 1661
 
 tss;island
 
 0.675588
 
 0.0099630
 
 NFIA (margin: NFIA; AC096534.1)
 
  7.7936929
 
 7.94e-03
 
 3.19e-02
  
 
  
 17
 
  30592997
 
  30593034
 
  PDF   STATS   ENSEMBL   UCSC 
 
   38
 
  5
 
 17253
 
 tss;island
 
 0.657925
 
 0.0086737
 
 RHBDL3 (margin: RHBDL3)
 
  7.7825555
 
 7.94e-03
 
 3.19e-02
  
 
  
 5
 
 131347439
 
 131347773
 
  PDF   STATS   ENSEMBL   UCSC 
 
  335
 
  5
 
 32266
 
 tss
 
 0.630365
 
 0.0202117
 
 AC034228.2; ACSL6 (margin: AC034228.3; AC034228.2; ACSL6)
 
  7.7646322
 
 7.94e-03
 
 3.19e-02
  
 
  
 1
 
  23751369
 
  23751761
 
  PDF   STATS   ENSEMBL   UCSC 
 
  393
 
  5
 
 1341
 
 tss
 
 0.885921
 
 0.0273623
 
 TCEA3 (margin: TCEA3; ASAP3)
 
  7.7263718
 
 7.94e-03
 
 3.19e-02
  
 
  
 2
 
 242212422
 
 242212528
 
  PDF   STATS   ENSEMBL   UCSC 
 
  107
 
  5
 
 23133
 
 gene;tss;island
 
 0.037830
 
 0.9258619
 
 HDLBP (margin: HDLBP)
 
  7.7263176
 
 7.94e-03
 
 3.19e-02
  
 
  
 7
 
  28996196
 
  28996639
 
  PDF   STATS   ENSEMBL   UCSC 
 
  444
 
  5
 
 36947
 
 gene;island
 
 0.643632
 
 0.0181728
 
 AC005013.5; AC005013.1; TRIL (margin: AC005013.5; AC005013.1; TRIL)
 
  7.7262438
 
 7.94e-03
 
 3.19e-02
  
 
  
 10
 
 116391467
 
 116391843
 
  PDF   STATS   ENSEMBL   UCSC 
 
  377
 
  5
 
 4769
 
 gene;island
 
 0.141263
 
 0.9729664
 
 ABLIM1 (margin: ABLIM1)
 
  7.7248521
 
 7.94e-03
 
 3.19e-02
  
 
  
 19
 
  50831762
 
  50832037
 
  PDF   STATS   ENSEMBL   UCSC 
 
  276
 
  5
 
 20173
 
 gene;island
 
 0.941142
 
 0.0538253
 
 NR1H2; KCNC3 (margin: NR1H2; KCNC3; NAPSB)
 
  7.7087204
 
 7.94e-03
 
 3.19e-02
  
 
  
 1
 
  40420503
 
  40420692
 
  PDF   STATS   ENSEMBL   UCSC 
 
  190
 
  9
 
 1531
 
 tss
 
 0.006049
 
 0.5433334
 
 MFSD2A (margin: MFSD2A; RP3-342P20.2; Y_RNA)
 
  7.7030761
 
 7.77e-03
 
 3.19e-02
  
 
  
 20
 
  37230326
 
  37230484
 
  PDF   STATS   ENSEMBL   UCSC 
 
  159
 
  5
 
 25737
 
 tss
 
 0.032872
 
 0.8748559
 
 ARHGAP40 (margin: ARHGAP40)
 
  7.6857119
 
 7.94e-03
 
 3.19e-02
  
 
  
 21
 
  43786663
 
  43786997
 
  PDF   STATS   ENSEMBL   UCSC 
 
  335
 
  5
 
 26567
 
 tss
 
 0.672652
 
 0.0166230
 
 TFF1 (margin: TFF1; TMPRSS3)
 
  7.6806789
 
 7.94e-03
 
 3.19e-02
  
 
  
 12
 
 121975121
 
 121975669
 
  PDF   STATS   ENSEMBL   UCSC 
 
  549
 
  5
 
 9369
 
 gene;island
 
 0.824954
 
 0.0088347
 
 KDM2B (margin: KDM2B)
 
  7.6387648
 
 7.94e-03
 
 3.19e-02
  
 
  
 7
 
  38468944
 
  38469273
 
  PDF   STATS   ENSEMBL   UCSC 
 
  330
 
  5
 
 36971
 
 gene
 
 0.018347
 
 0.8515830
 
 AMPH (margin: AMPH)
 
  7.6327739
 
 7.94e-03
 
 3.19e-02
  
 
  
 8
 
 110704026
 
 110704289
 
  PDF   STATS   ENSEMBL   UCSC 
 
  264
 
  5
 
 39874
 
 tss
 
 0.641600
 
 0.0108684
 
 SYBU (margin: SYBU)
 
  7.6295572
 
 7.94e-03
 
 3.19e-02
  
 
  
 17
 
  38465281
 
  38465422
 
  PDF   STATS   ENSEMBL   UCSC 
 
  142
 
  5
 
 17329
 
 tss
 
 0.559284
 
 0.0057663
 
 RARA (margin: CDC6; CTD-2267D19.6; RARA)
 
  7.6113027
 
 7.94e-03
 
 3.19e-02
  
 
  
 3
 
 191048308
 
 191048439
 
  PDF   STATS   ENSEMBL   UCSC 
 
  132
 
  5
 
 28084
 
 gene;island
 
 0.001429
 
 0.3244087
 
 CCDC50; UTS2B (margin: CCDC50; UTS2B)
 
  7.5870219
 
 7.94e-03
 
 3.19e-02
  
 
  
 4
 
  96470237
 
  96470349
 
  PDF   STATS   ENSEMBL   UCSC 
 
  113
 
  5
 
 30505
 
 tss
 
 0.792742
 
 0.0078895
 
 RP11-710C12.1; UNC5C (margin: RP11-710C12.1; UNC5C)
 
  7.5664401
 
 7.94e-03
 
 3.19e-02
  
 
  
 17
 
  21279561
 
  21279696
 
  PDF   STATS   ENSEMBL   UCSC 
 
  136
 
  5
 
 17203
 
 tss
 
 0.455491
 
 0.9850824
 
 KCNJ12 (margin: KCNJ12)
 
  7.5616430
 
 7.94e-03
 
 3.19e-02
  
 
  
 8
 
  22560922
 
  22561238
 
  PDF   STATS   ENSEMBL   UCSC 
 
  317
 
  5
 
 40142
 
 island
 
 0.002856
 
 0.3751584
 
 RP11-459E5.1 (margin: RP11-459E5.1; EGR3; PEBP4)
 
  7.5563483
 
 7.94e-03
 
 3.19e-02
  
 
  
 6
 
 106958303
 
 106958645
 
  PDF   STATS   ENSEMBL   UCSC 
 
  343
 
  5
 
 35150
 
 tss;island
 
 0.873249
 
 0.0130015
 
 AIM1 (margin: AIM1)
 
  7.5485975
 
 7.94e-03
 
 3.19e-02
  
 
  
 1
 
 179560700
 
 179560983
 
  PDF   STATS   ENSEMBL   UCSC 
 
  284
 
  5
 
 2119
 
 tss
 
 0.545865
 
 0.0112335
 
 RP11-545A16.4; TDRD5 (margin: RP11-545A16.3; RP11-545A16.4; TDRD5)
 
  7.5348817
 
 7.94e-03
 
 3.19e-02
  
 
  
 11
 
 118401536
 
 118401878
 
  PDF   STATS   ENSEMBL   UCSC 
 
  343
 
  5
 
 8858
 
 island
 
 0.410006
 
 0.9782230
 
 TTC36; TMEM25; RP11-770J1.3 (margin: KMT2A; TTC36; TMEM25; RP11-770J1.3)
 
  7.5180742
 
 7.94e-03
 
 3.19e-02
  
 
  
 5
 
 140345461
 
 140346403
 
  PDF   STATS   ENSEMBL   UCSC 
 
  943
 
 14
 
 31806
 
 gene;island
 
 0.654195
 
 0.0173700
 
 PCDHA1; PCDHA2; PCDHA3; PCDHA4; PCDHA5; PCDHA6; PCDHA7; PCDHA8; PCDHA9; PCDHA10; PCDHA11; PCDHA12; PCDHA13; PCDHAC1; PCDHAC2 (margin: PCDHA1; PCDHA2; PCDHA3; PCDHA4; PCDHA5; PCDHA6; PCDHA7; PCDHA8; PCDHA9; PCDHA10; PCDHA11; PCDHA12; PCDHA13; PCDHAC1; PCDHAC2)
 
  7.4696728
 
 6.74e-03
 
 3.19e-02
  
 
  
 1
 
 197881327
 
 197881540
 
  PDF   STATS   ENSEMBL   UCSC 
 
  214
 
  5
 
 2157
 
 tss;island
 
 0.035328
 
 0.9566167
 
 LHX9 (margin: C1orf53; LHX9)
 
  7.4127766
 
 7.94e-03
 
 3.19e-02
  
 
  
 7
 
    193229
 
    193732
 
  PDF   STATS   ENSEMBL   UCSC 
 
  504
 
  5
 
 36701
 
 gene;island
 
 0.017854
 
 0.6821618
 
 FAM20C; AC093627.12 (margin: FAM20C; AC093627.12)
 
  7.3902622
 
 7.94e-03
 
 3.19e-02
  
 
  
 21
 
  40984760
[truncated: 4,784,472 more chars]
