# Supplementary figures and images for: DMRforPairs: identifying Differentially Methylated Regions between unique samples using array based methylation profiles (part 2 of 2)
Source: BMC Bioinformatics. 2014 May 15;15:141. doi: 10.1186/1471-2105-15-141 (PMC4046028; doi:10.1186/1471-2105-15-141)

RegionID: 10141, chr12:298151–298484–M\_values

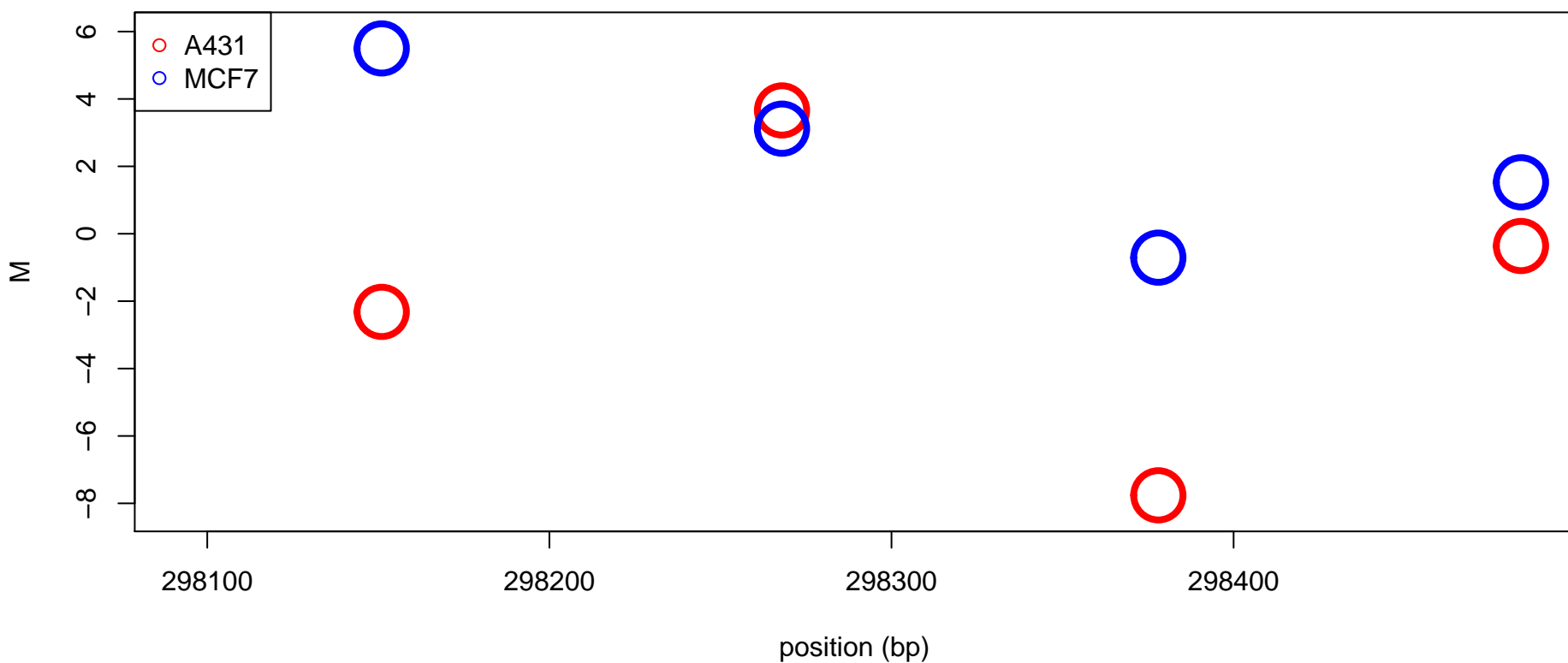

RegionID: 10141, chr12:298151–298484–Beta\_values

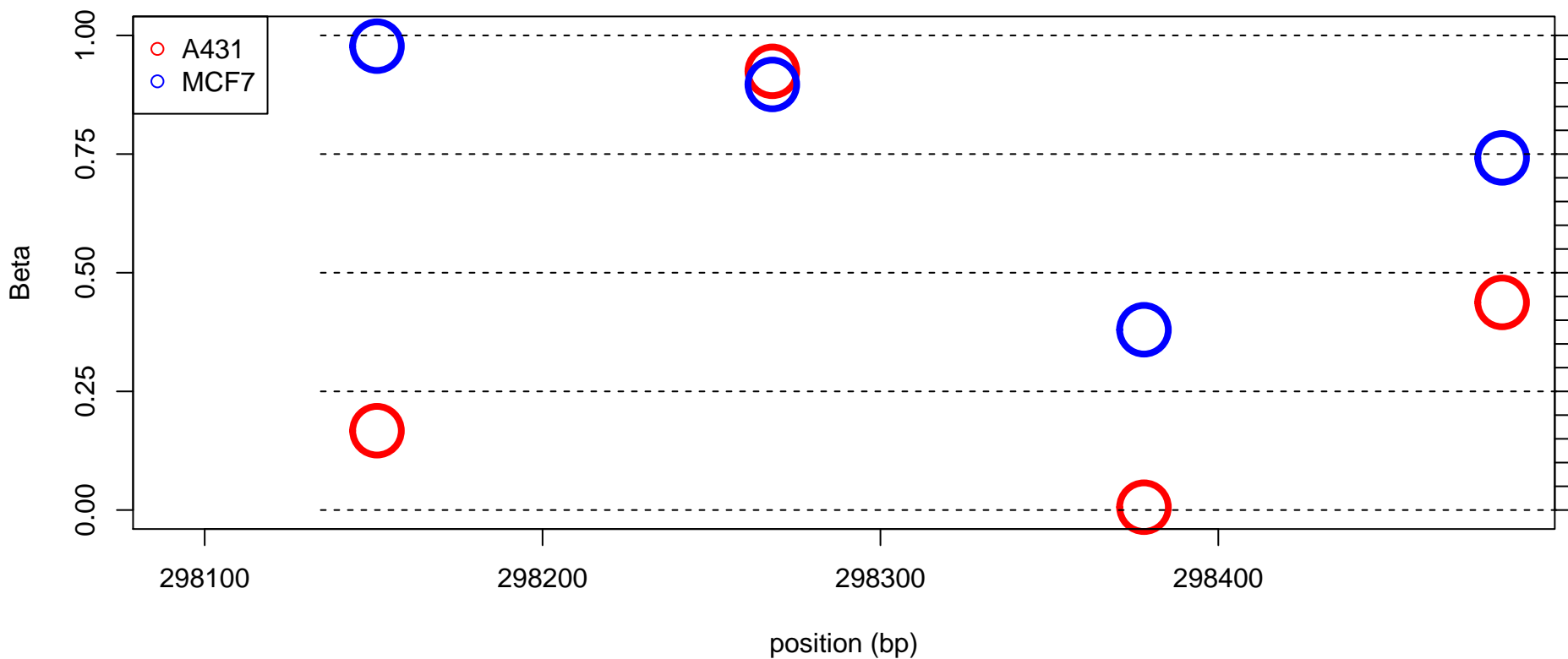

Supplement: Additional file 2 — DMRforPairs output for the comparison of A431-MCF7 and NA17018-NA17105. Please start from the HTML files in each folder. Available via the BMC Bioinformatics website. [file 1471-2105-15-141-S2.zip › 1394847754114233_MOESM2_ESM/A431_MCF7/figures/10141.pdf]

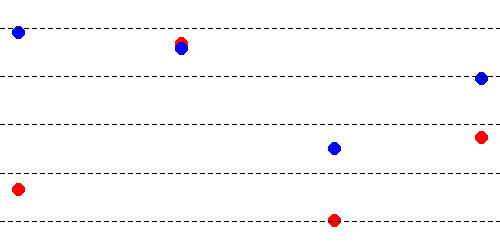

Supplement: Additional file 2 — DMRforPairs output for the comparison of A431-MCF7 and NA17018-NA17105. Please start from the HTML files in each folder. Available via the BMC Bioinformatics website. [file 1471-2105-15-141-S2.zip › 1394847754114233_MOESM2_ESM/A431_MCF7/figures/10141.png]

RegionID: 10155, chr12:2162130-2162491-M\_values

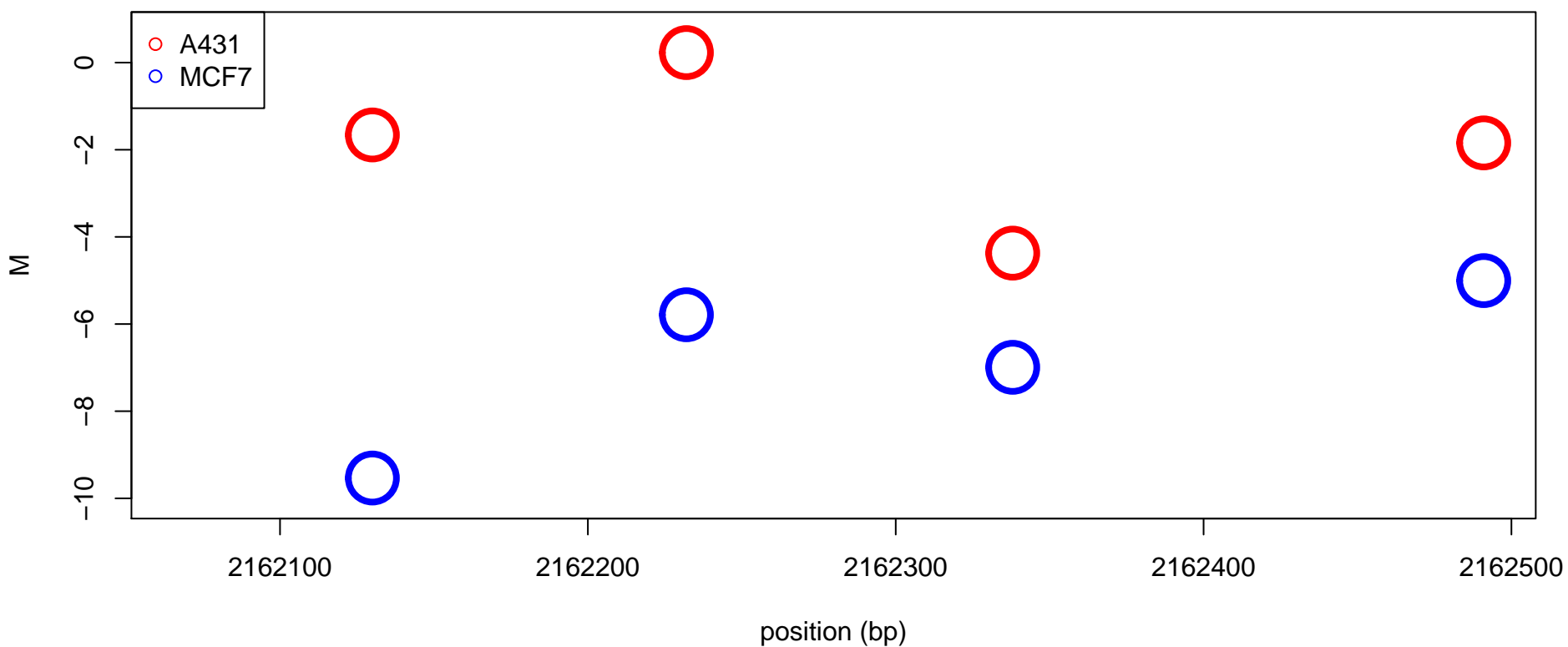

RegionID: 10155, chr12:2162130-2162491-Beta\_values

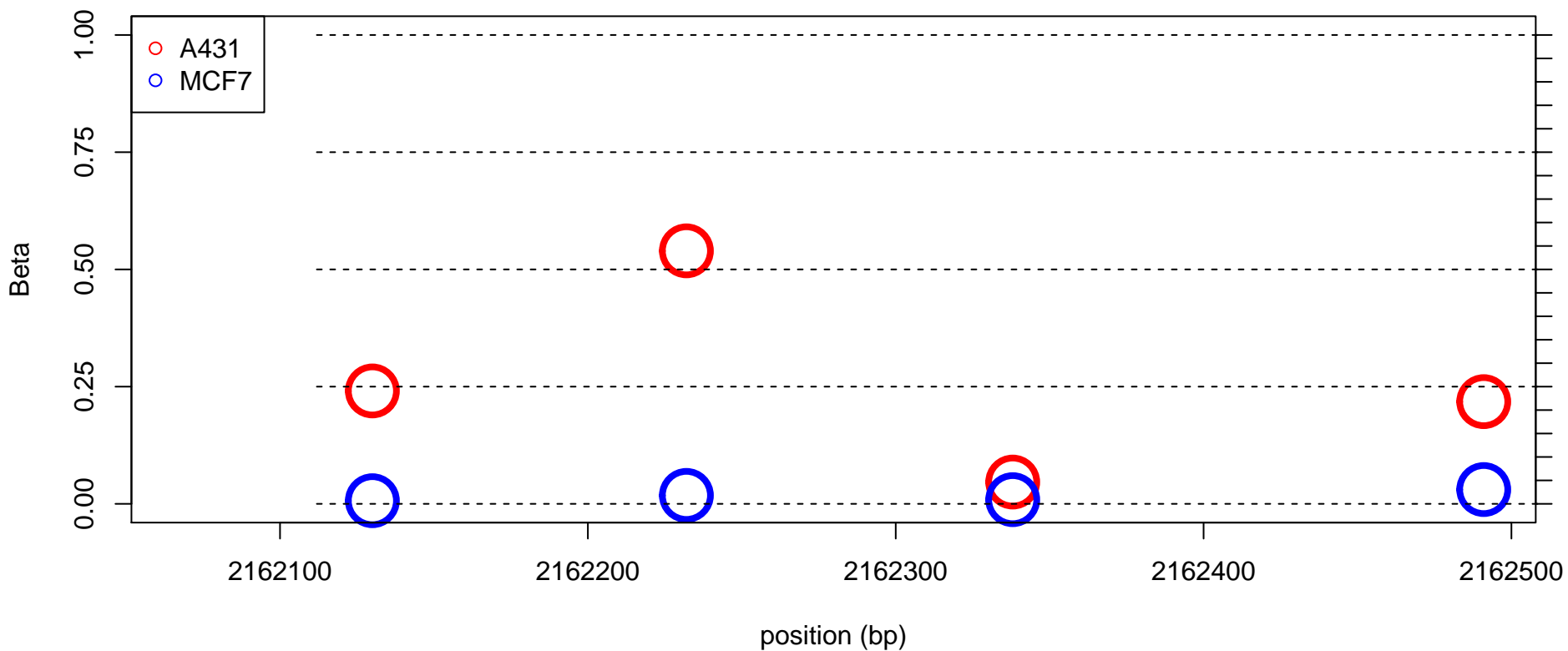

Supplement: Additional file 2 — DMRforPairs output for the comparison of A431-MCF7 and NA17018-NA17105. Please start from the HTML files in each folder. Available via the BMC Bioinformatics website. [file 1471-2105-15-141-S2.zip › 1394847754114233_MOESM2_ESM/A431_MCF7/figures/10155.pdf]

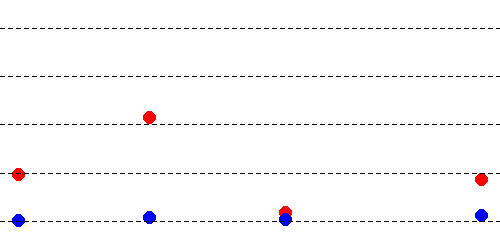

Supplement: Additional file 2 — DMRforPairs output for the comparison of A431-MCF7 and NA17018-NA17105. Please start from the HTML files in each folder. Available via the BMC Bioinformatics website. [file 1471-2105-15-141-S2.zip › 1394847754114233_MOESM2_ESM/A431_MCF7/figures/10155.png]

RegionID: 10168, chr12:4140624-4141193-M\_values

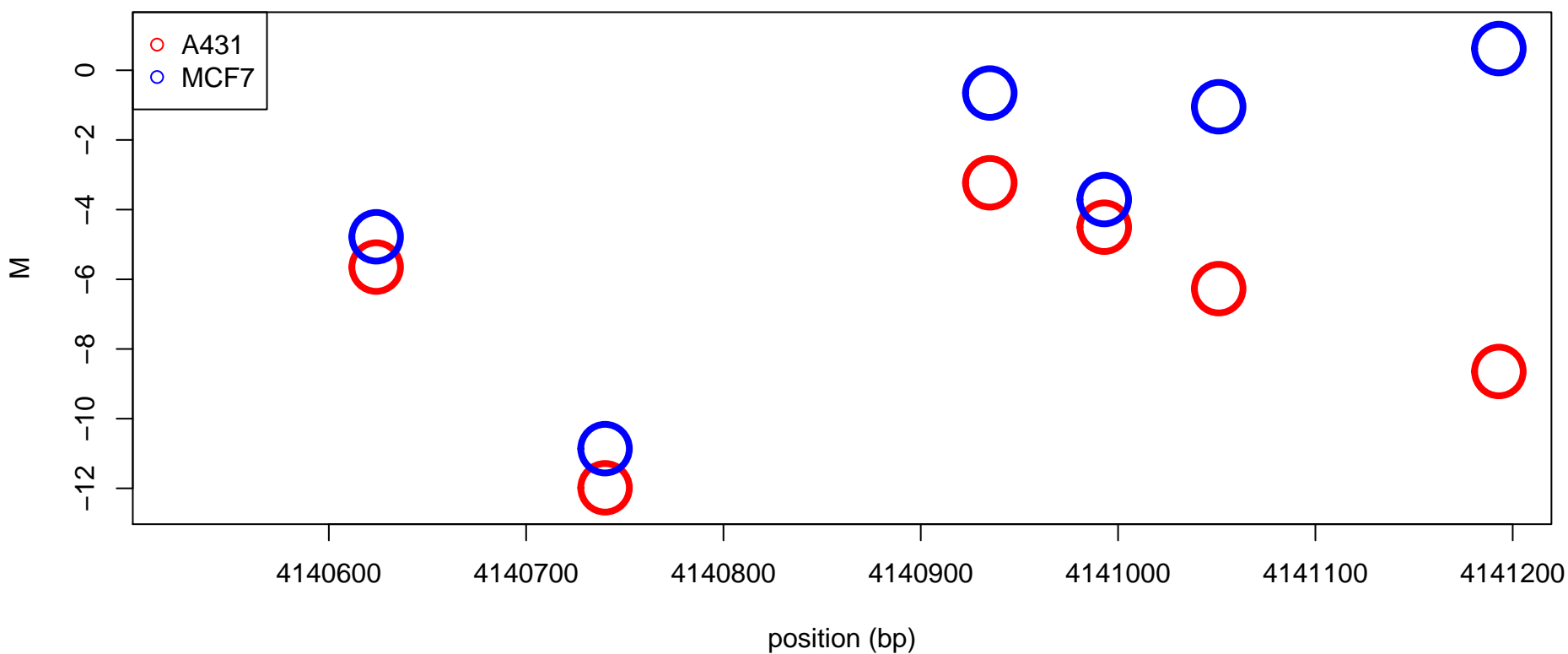

RegionID: 10168, chr12:4140624-4141193-Beta\_values

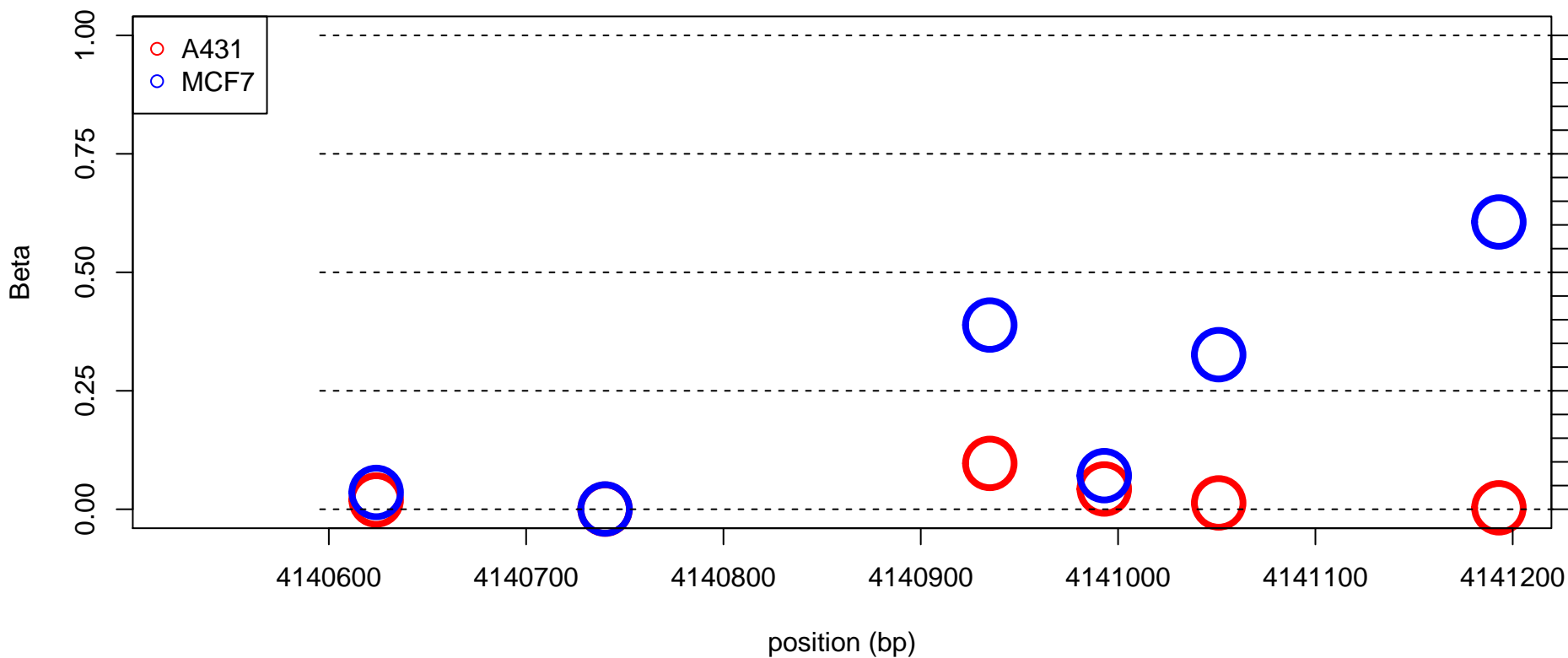

Supplement: Additional file 2 — DMRforPairs output for the comparison of A431-MCF7 and NA17018-NA17105. Please start from the HTML files in each folder. Available via the BMC Bioinformatics website. [file 1471-2105-15-141-S2.zip › 1394847754114233_MOESM2_ESM/A431_MCF7/figures/10168.pdf]

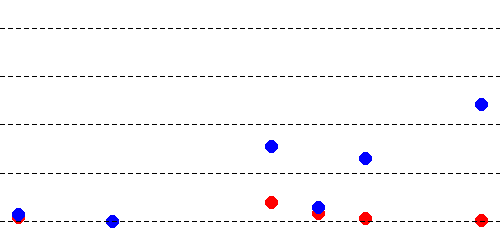

Supplement: Additional file 2 — DMRforPairs output for the comparison of A431-MCF7 and NA17018-NA17105. Please start from the HTML files in each folder. Available via the BMC Bioinformatics website. [file 1471-2105-15-141-S2.zip › 1394847754114233_MOESM2_ESM/A431_MCF7/figures/10168.png]

RegionID: 10171, chr12:4382878-4383281-M\_values

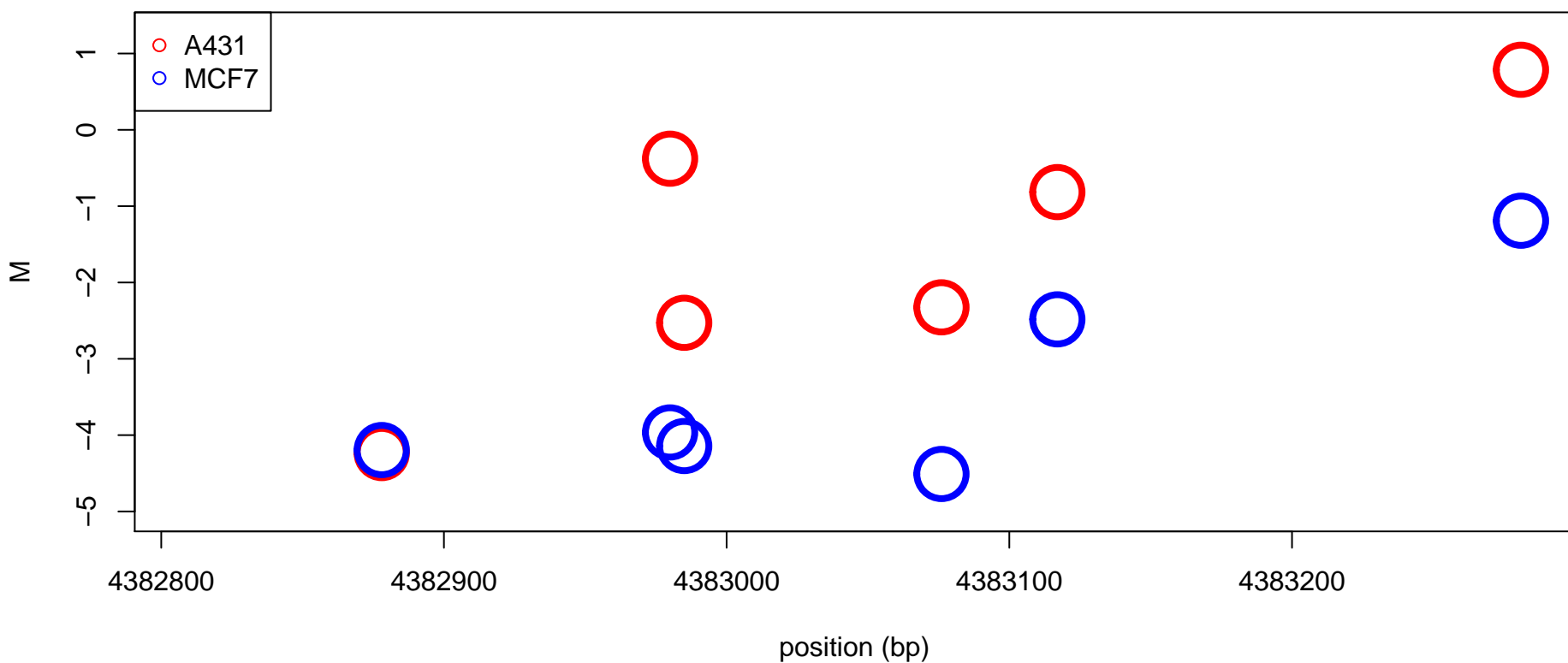

RegionID: 10171, chr12:4382878-4383281-Beta\_values

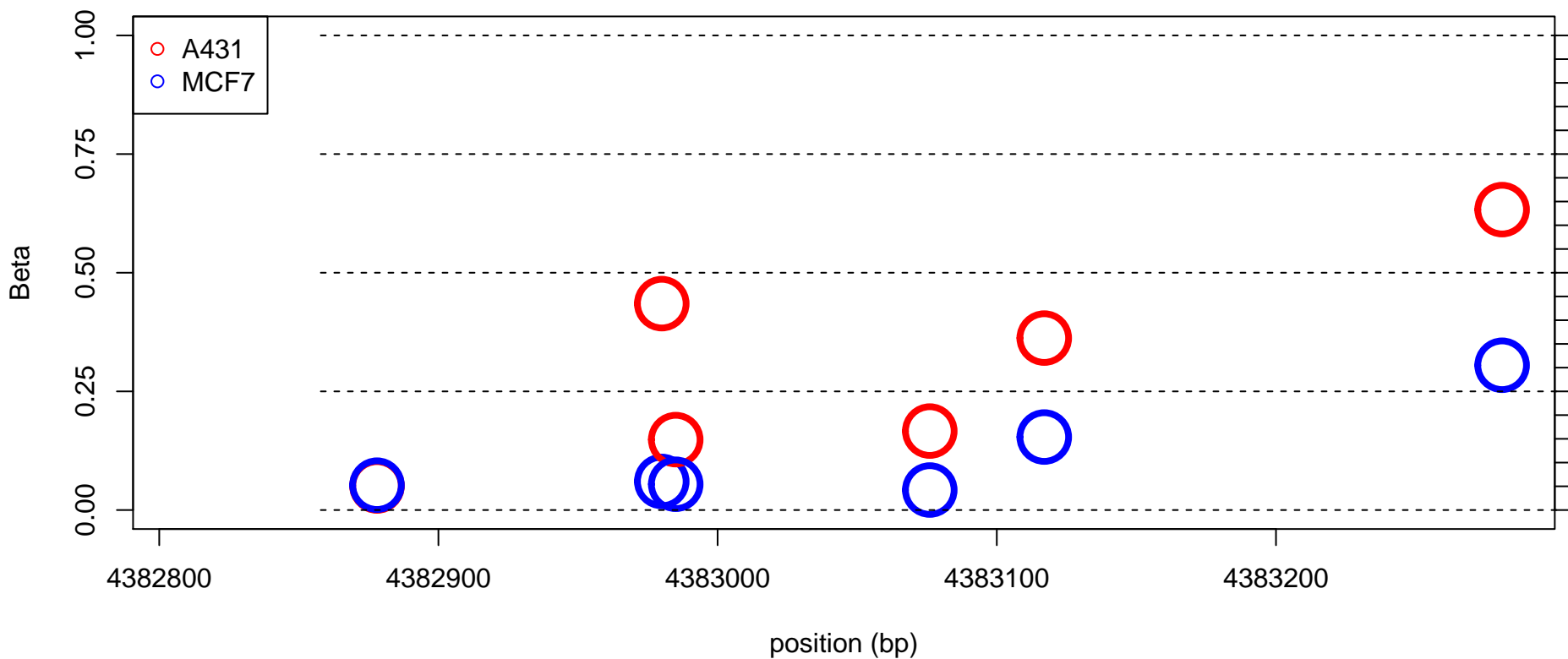

Supplement: Additional file 2 — DMRforPairs output for the comparison of A431-MCF7 and NA17018-NA17105. Please start from the HTML files in each folder. Available via the BMC Bioinformatics website. [file 1471-2105-15-141-S2.zip › 1394847754114233_MOESM2_ESM/A431_MCF7/figures/10171.pdf]

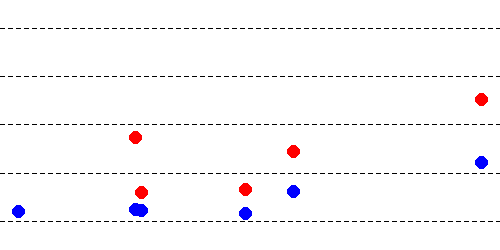

Supplement: Additional file 2 — DMRforPairs output for the comparison of A431-MCF7 and NA17018-NA17105. Please start from the HTML files in each folder. Available via the BMC Bioinformatics website. [file 1471-2105-15-141-S2.zip › 1394847754114233_MOESM2_ESM/A431_MCF7/figures/10171.png]

RegionID: 10175, chr12:4554295-4554971-M\_values

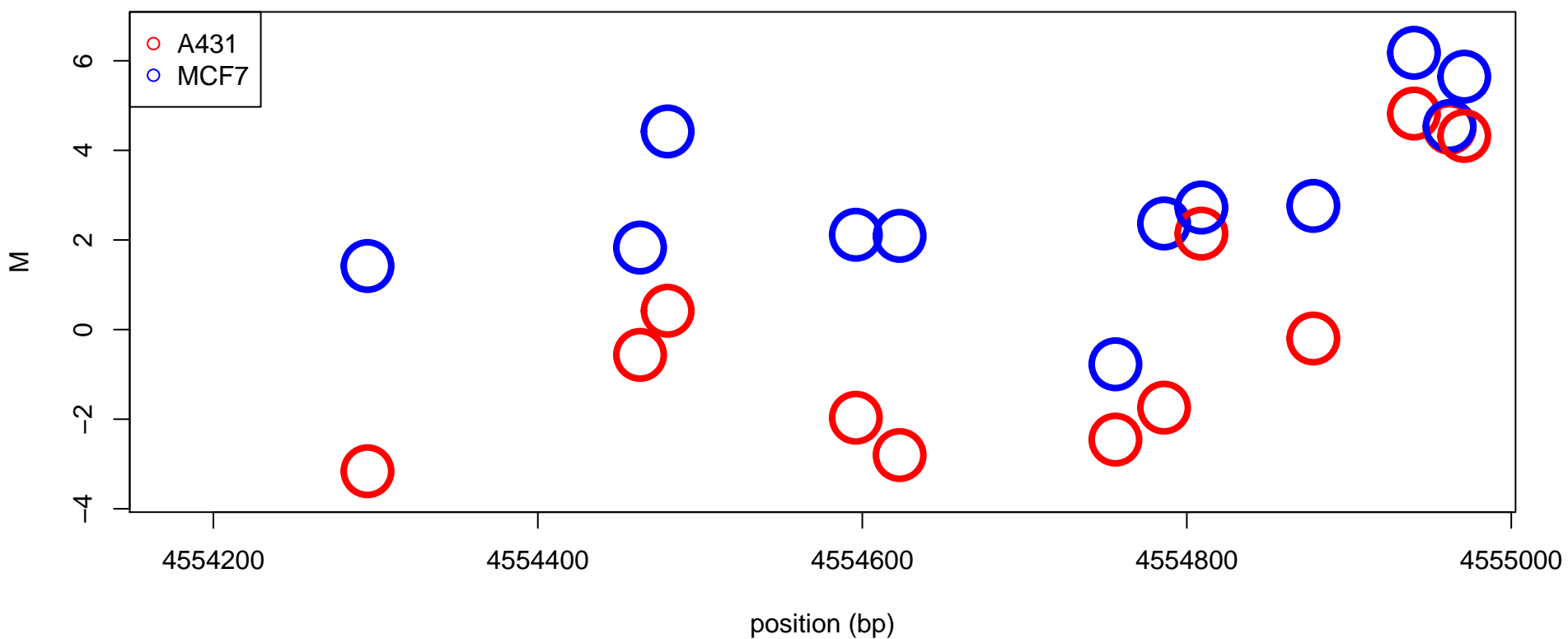

RegionID: 10175, chr12:4554295-4554971-Beta\_values

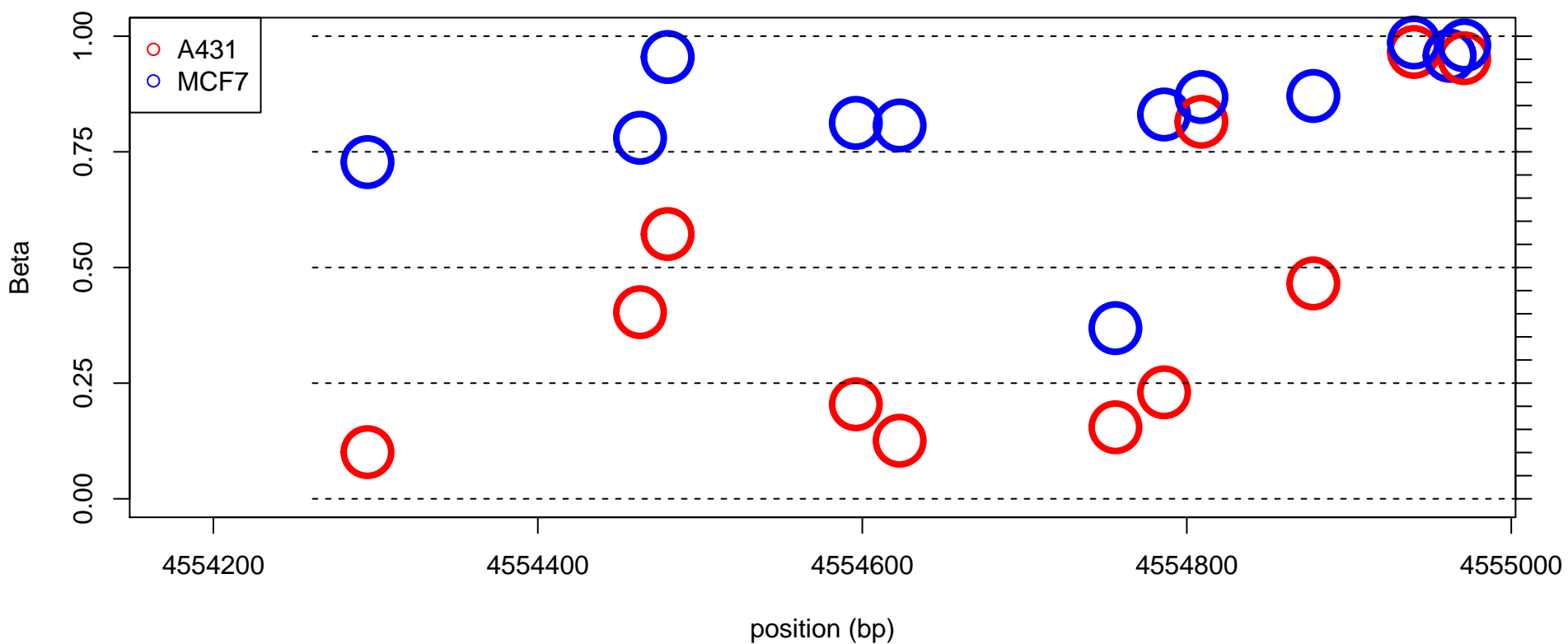

Supplement: Additional file 2 — DMRforPairs output for the comparison of A431-MCF7 and NA17018-NA17105. Please start from the HTML files in each folder. Available via the BMC Bioinformatics website. [file 1471-2105-15-141-S2.zip › 1394847754114233_MOESM2_ESM/A431_MCF7/figures/10175.pdf]

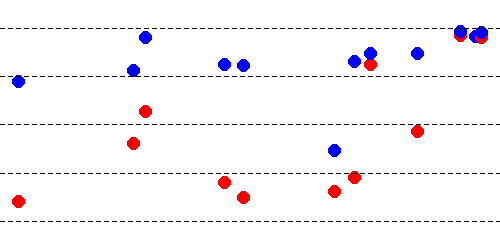

Supplement: Additional file 2 — DMRforPairs output for the comparison of A431-MCF7 and NA17018-NA17105. Please start from the HTML files in each folder. Available via the BMC Bioinformatics website. [file 1471-2105-15-141-S2.zip › 1394847754114233_MOESM2_ESM/A431_MCF7/figures/10175.png]

RegionID: 10180, chr12:5018715-5019091-M\_values

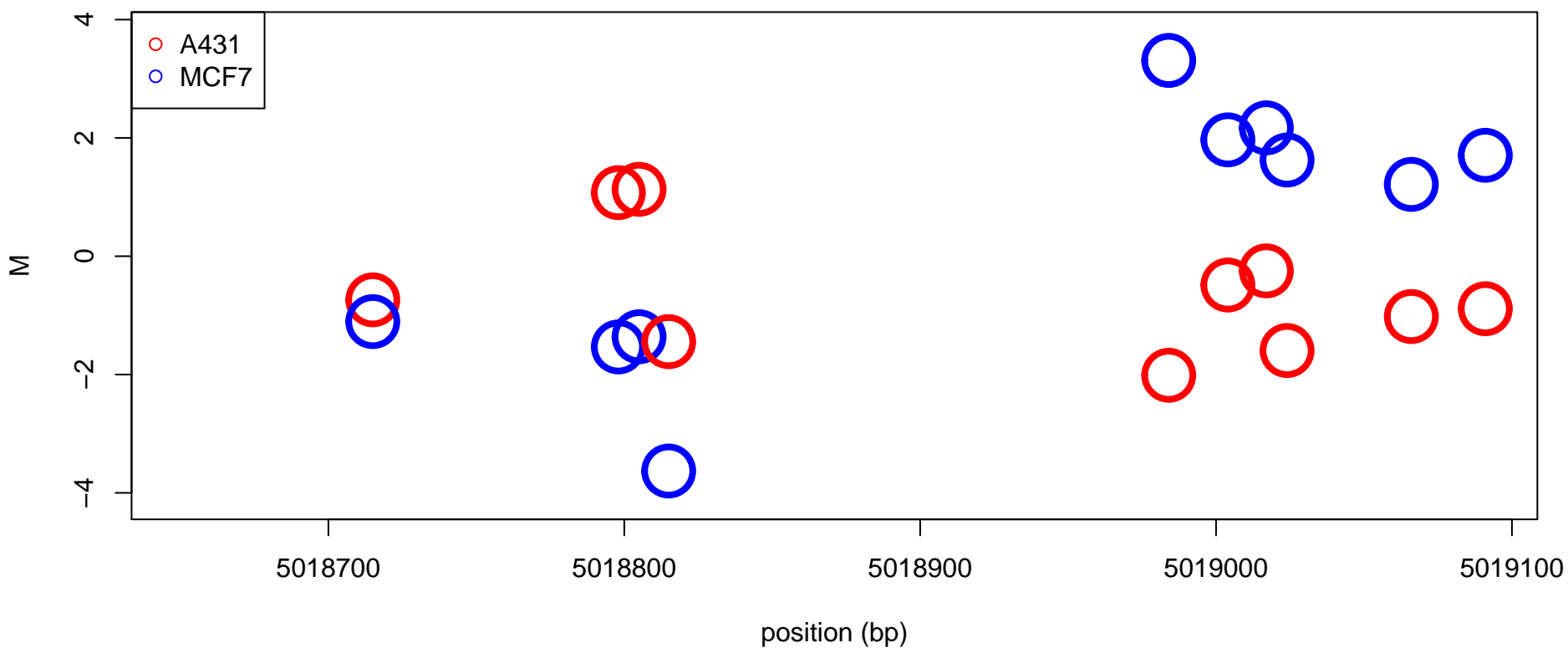

RegionID: 10180, chr12:5018715-5019091-Beta\_values

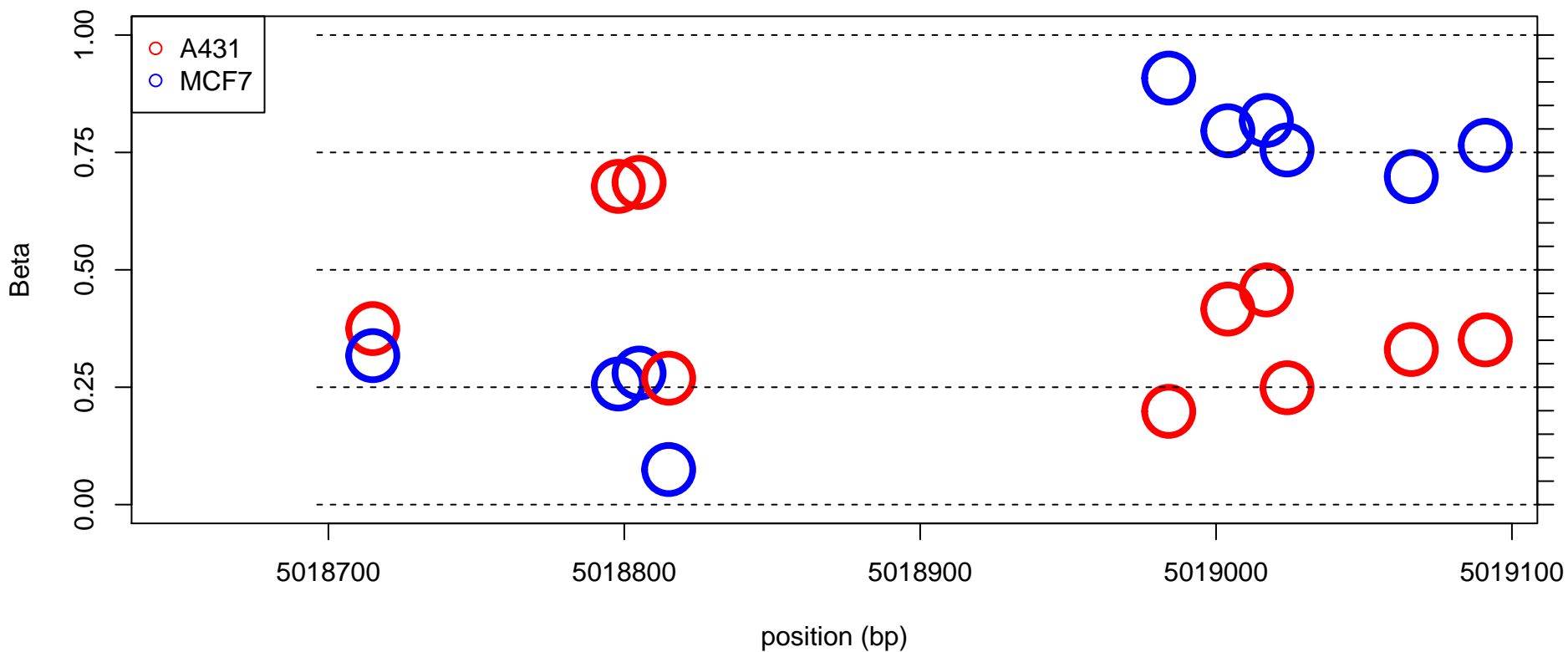

Supplement: Additional file 2 — DMRforPairs output for the comparison of A431-MCF7 and NA17018-NA17105. Please start from the HTML files in each folder. Available via the BMC Bioinformatics website. [file 1471-2105-15-141-S2.zip › 1394847754114233_MOESM2_ESM/A431_MCF7/figures/10180.pdf]

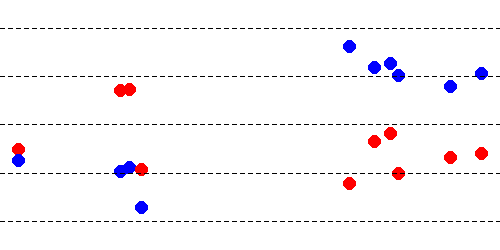

Supplement: Additional file 2 — DMRforPairs output for the comparison of A431-MCF7 and NA17018-NA17105. Please start from the HTML files in each folder. Available via the BMC Bioinformatics website. [file 1471-2105-15-141-S2.zip › 1394847754114233_MOESM2_ESM/A431_MCF7/figures/10180.png]

RegionID: 10182, chr12:5541220-5541403-M\_values

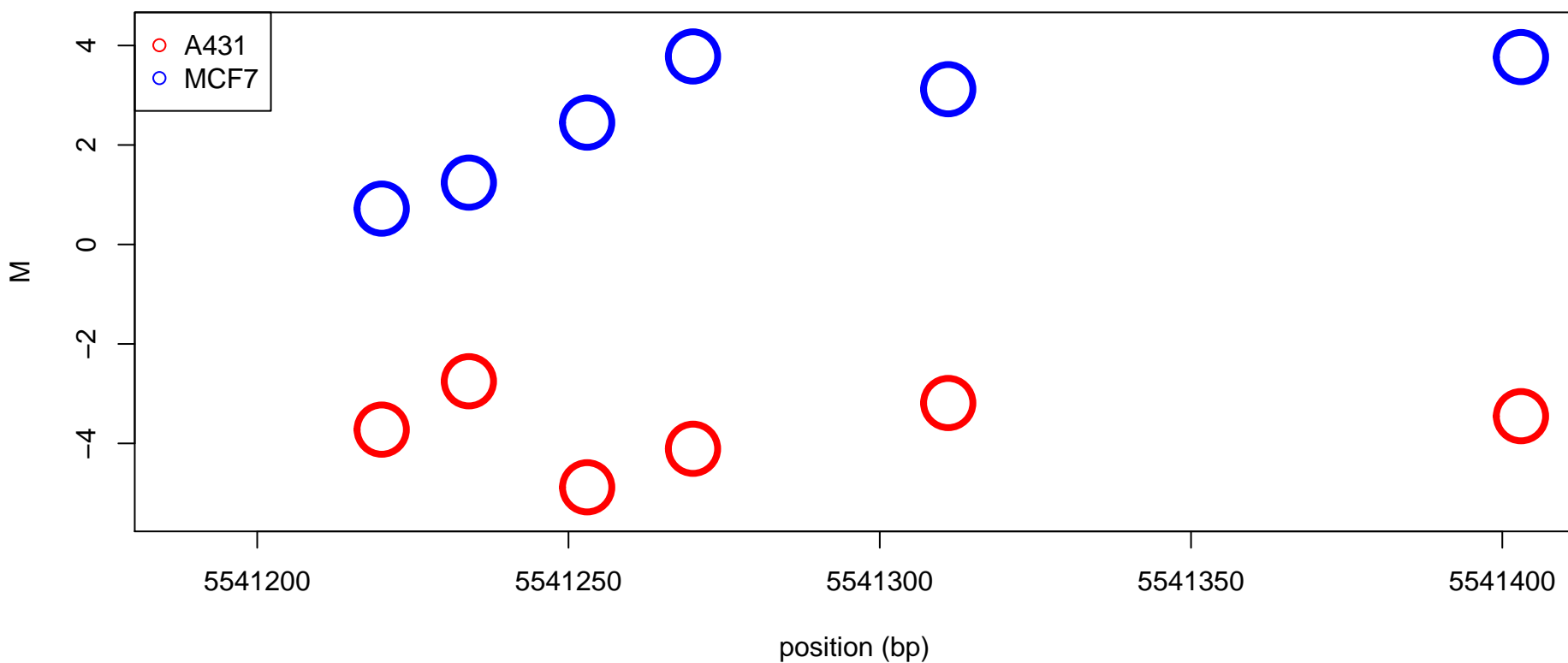

RegionID: 10182, chr12:5541220-5541403-Beta\_values

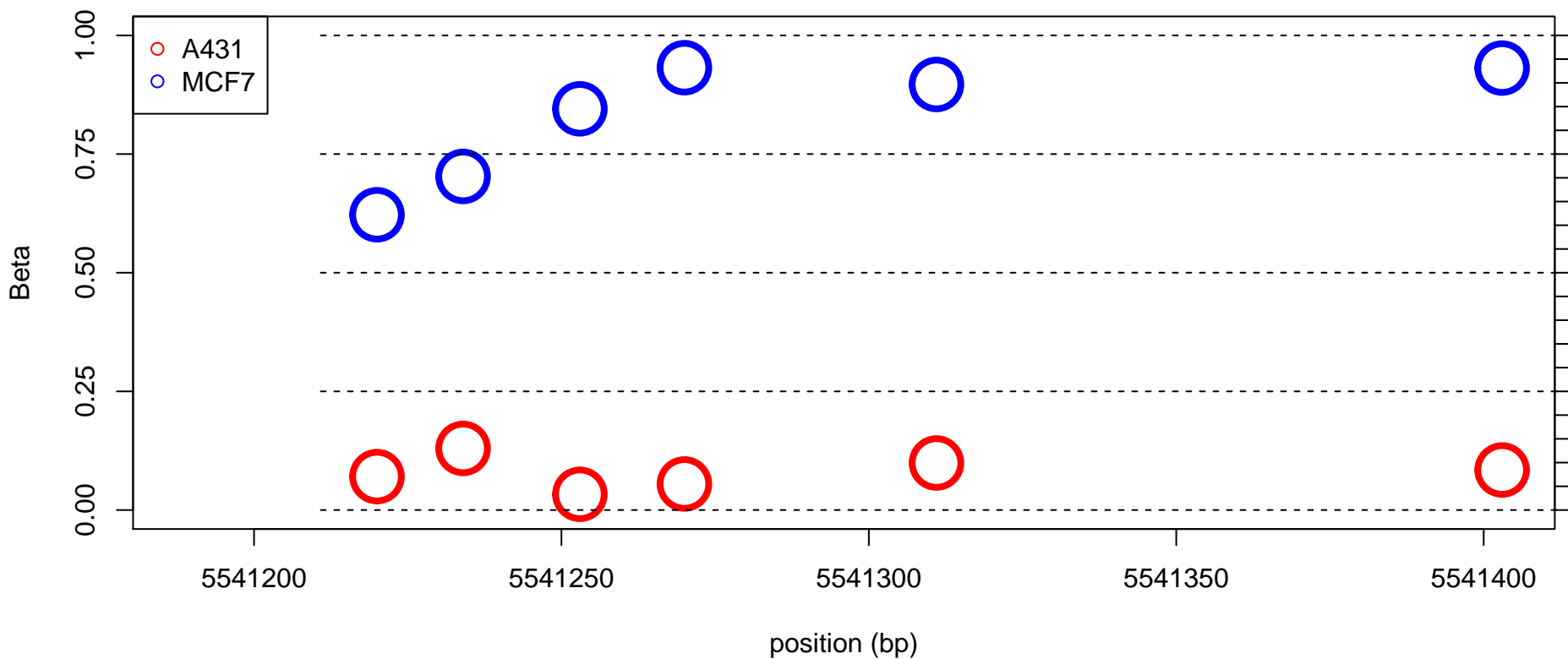

Chromosome 12

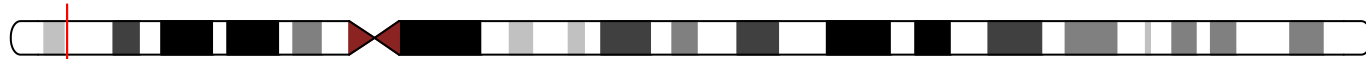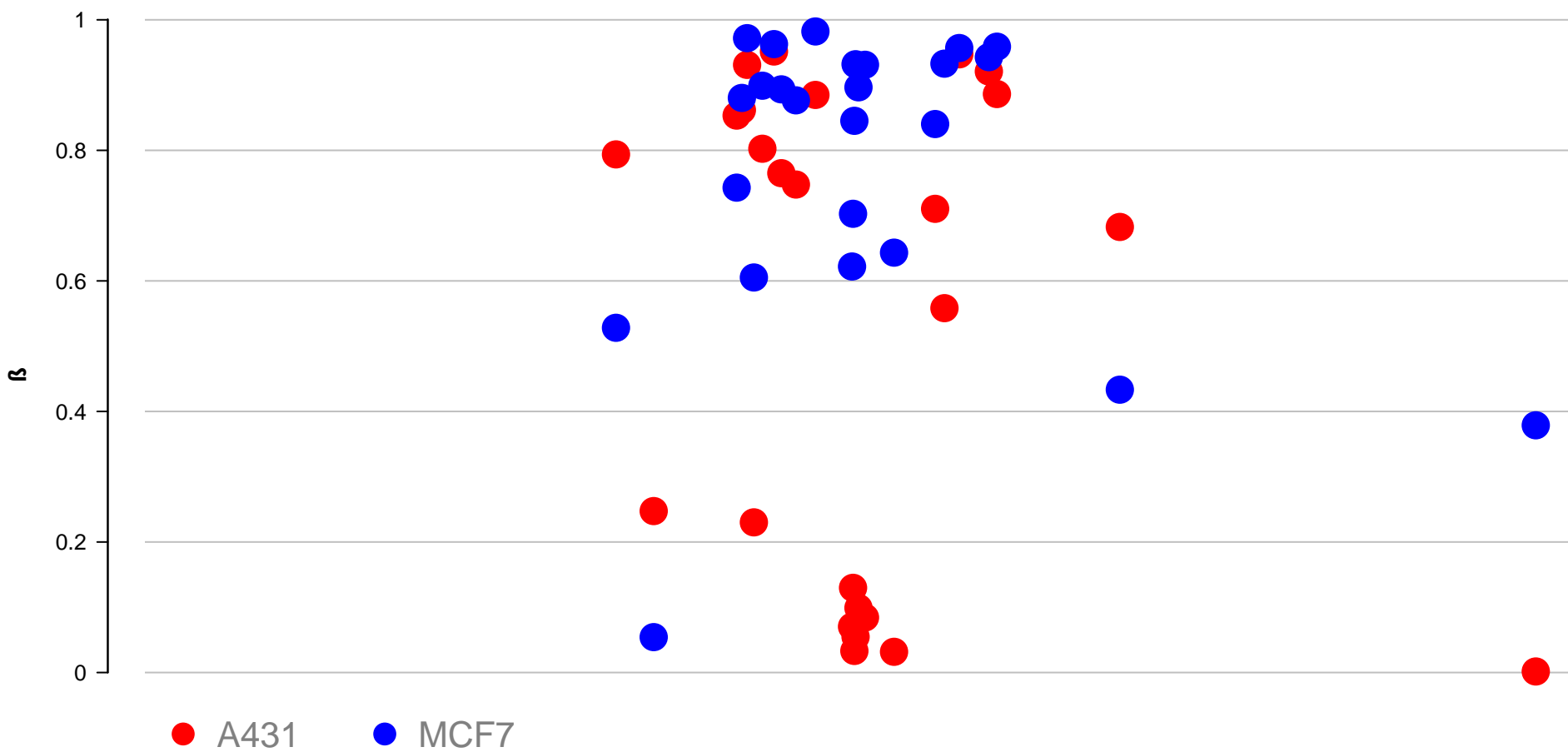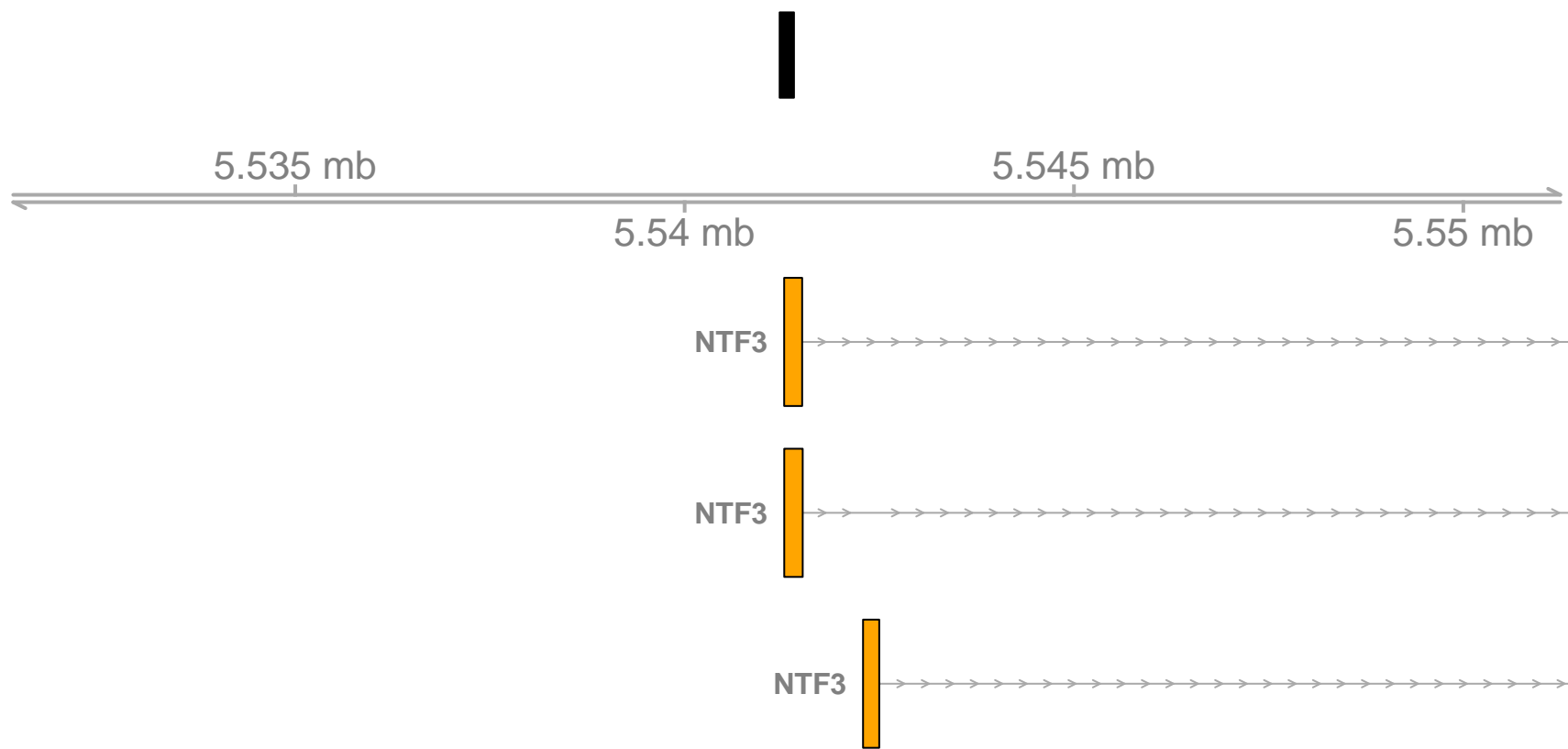

Supplement: Additional file 2 — DMRforPairs output for the comparison of A431-MCF7 and NA17018-NA17105. Please start from the HTML files in each folder. Available via the BMC Bioinformatics website. [file 1471-2105-15-141-S2.zip › 1394847754114233_MOESM2_ESM/A431_MCF7/figures/10182.pdf]

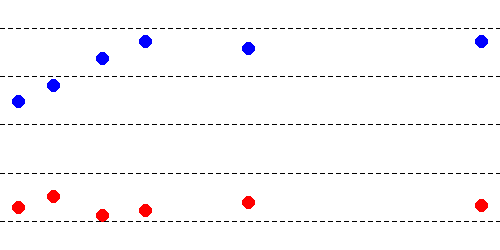

Supplement: Additional file 2 — DMRforPairs output for the comparison of A431-MCF7 and NA17018-NA17105. Please start from the HTML files in each folder. Available via the BMC Bioinformatics website. [file 1471-2105-15-141-S2.zip › 1394847754114233_MOESM2_ESM/A431_MCF7/figures/10182.png]

RegionID: 1019, chr1:220864119–220864321–M\_values

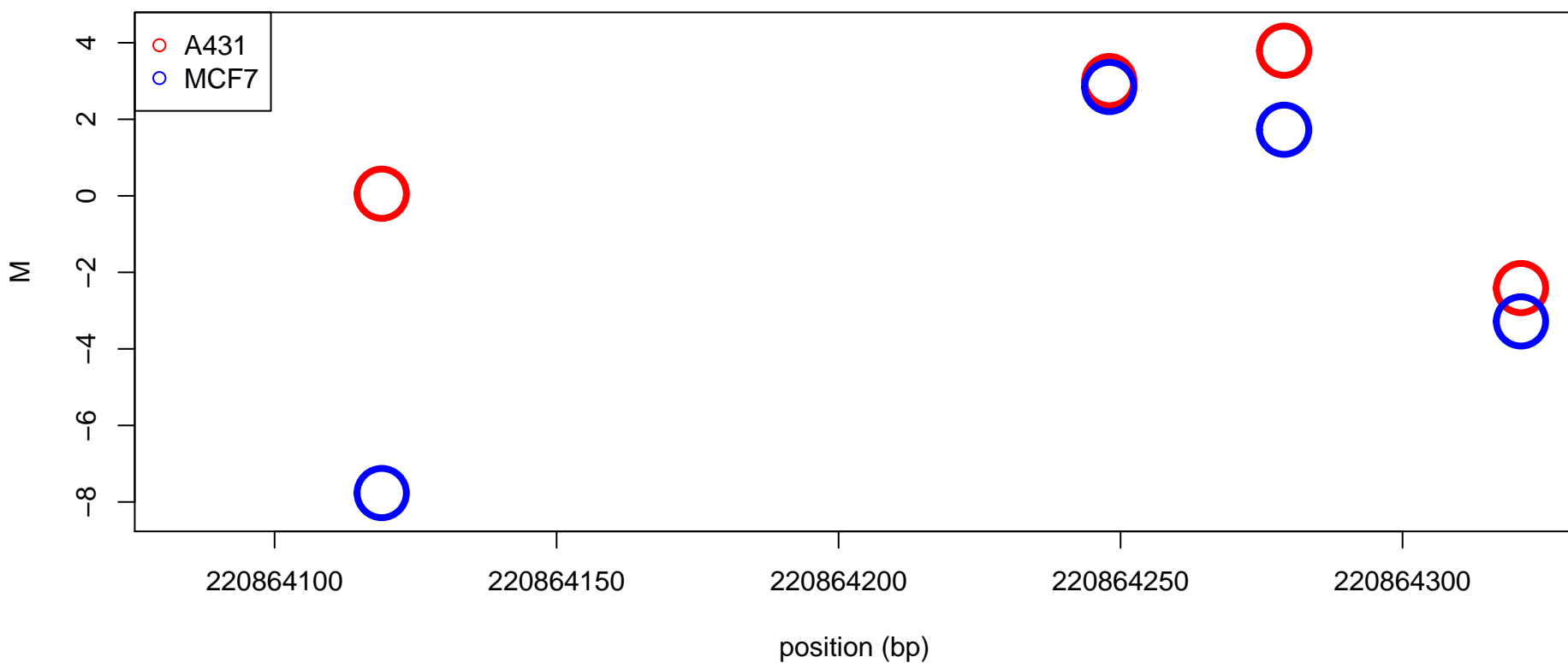

RegionID: 1019, chr1:220864119–220864321–Beta\_values

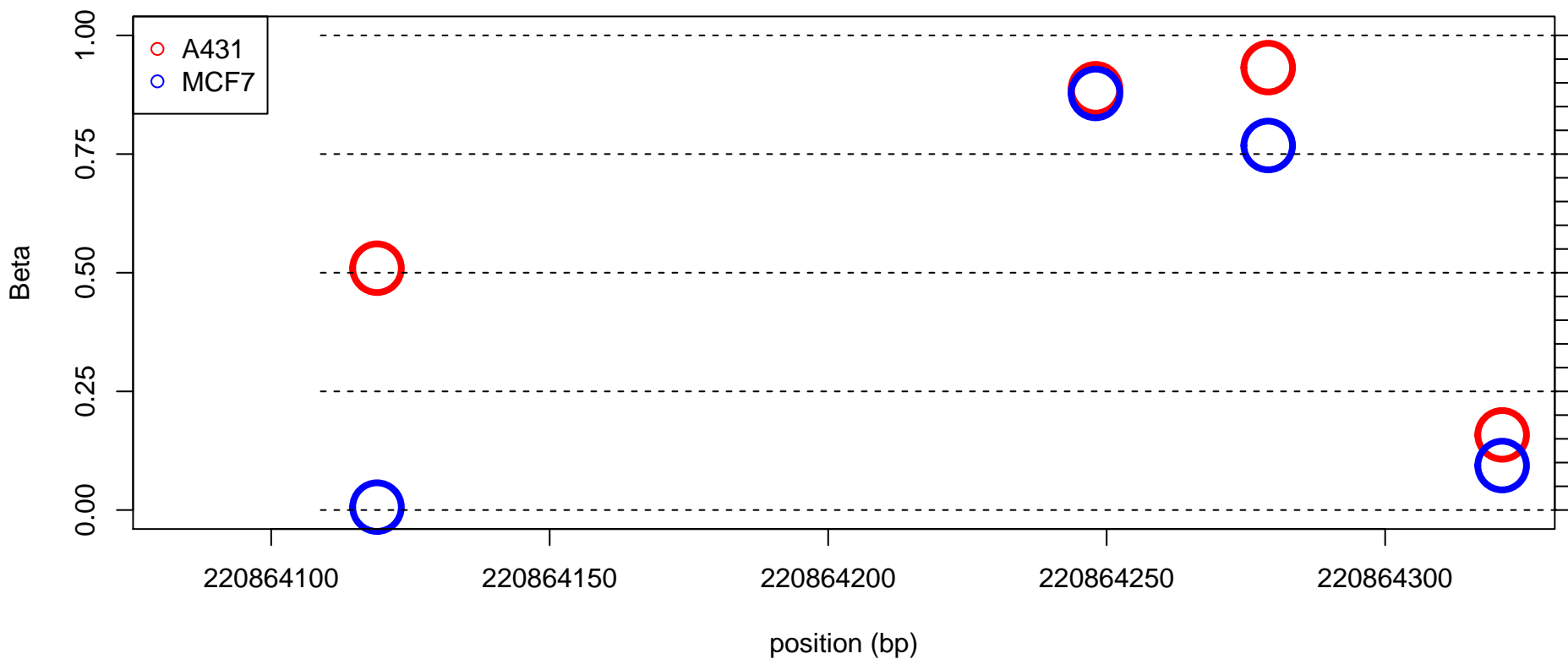

Supplement: Additional file 2 — DMRforPairs output for the comparison of A431-MCF7 and NA17018-NA17105. Please start from the HTML files in each folder. Available via the BMC Bioinformatics website. [file 1471-2105-15-141-S2.zip › 1394847754114233_MOESM2_ESM/A431_MCF7/figures/1019.pdf]

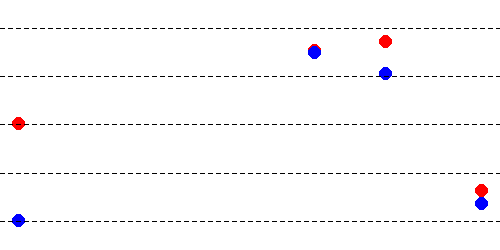

Supplement: Additional file 2 — DMRforPairs output for the comparison of A431-MCF7 and NA17018-NA17105. Please start from the HTML files in each folder. Available via the BMC Bioinformatics website. [file 1471-2105-15-141-S2.zip › 1394847754114233_MOESM2_ESM/A431_MCF7/figures/1019.png]

RegionID: 102, chr1:3056542–3056951–M\_values

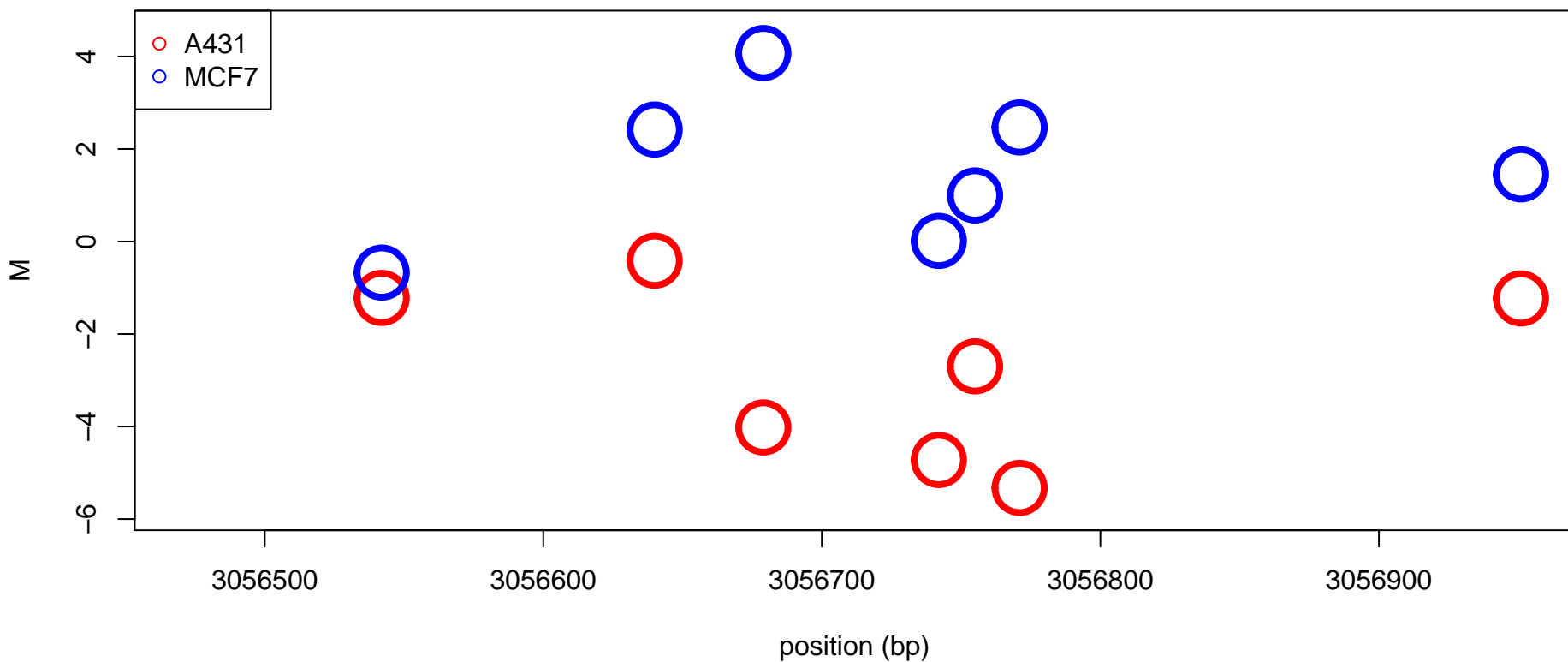

RegionID: 102, chr1:3056542–3056951–Beta\_values

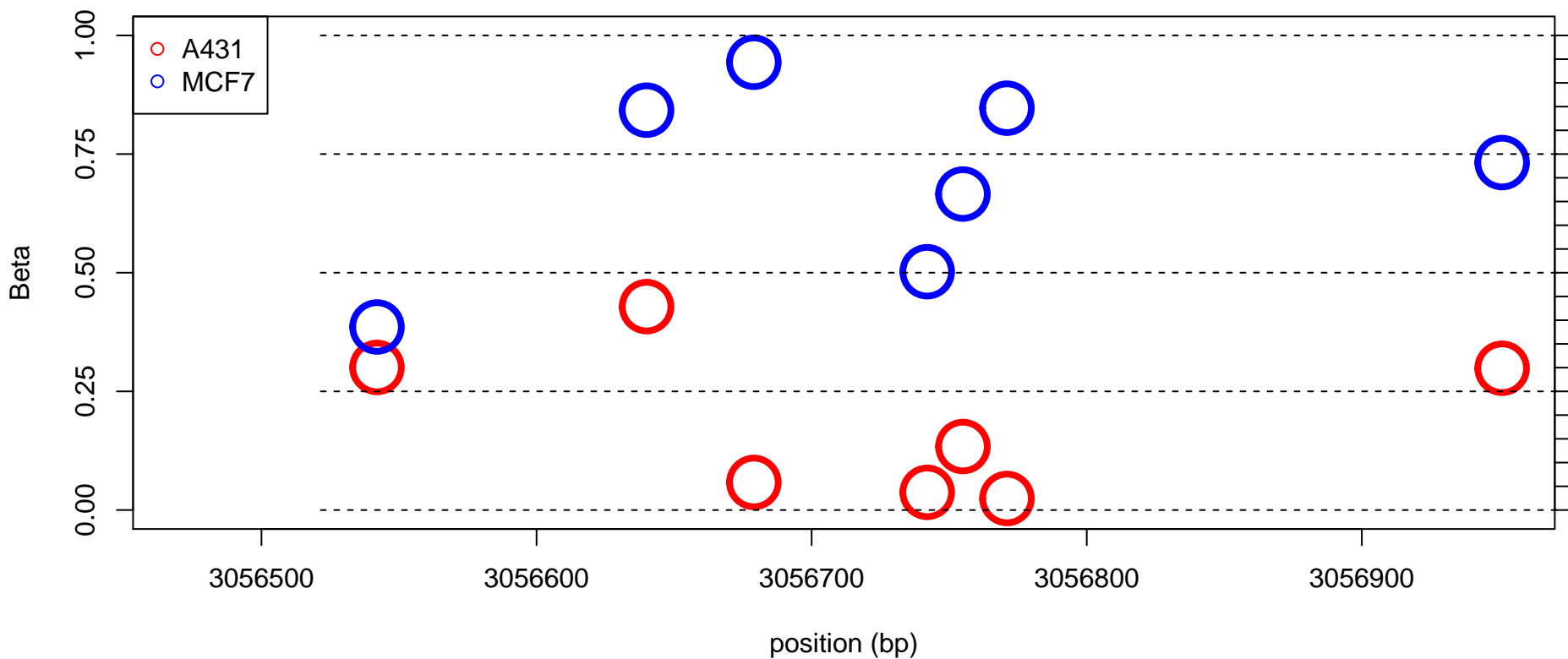

Chromosome 1

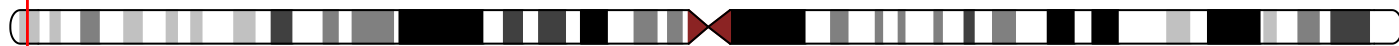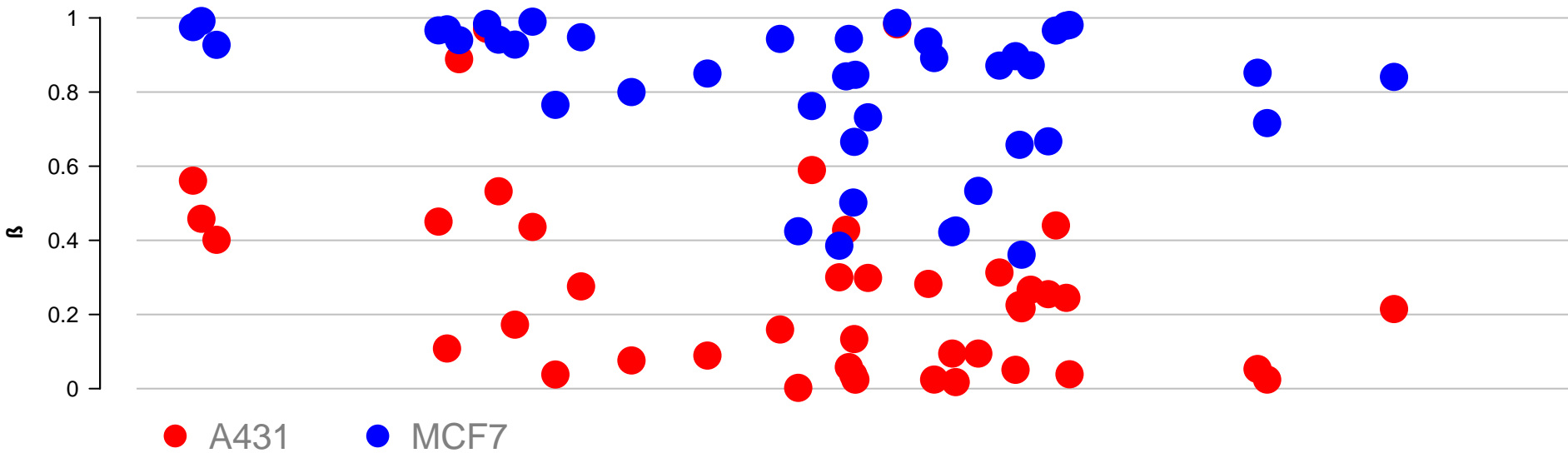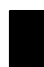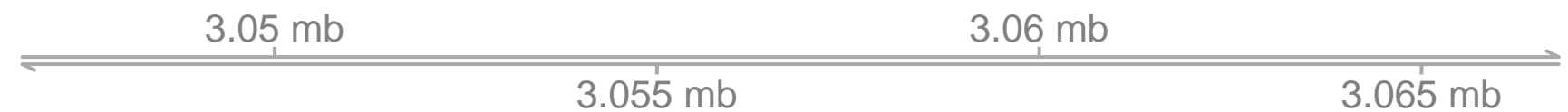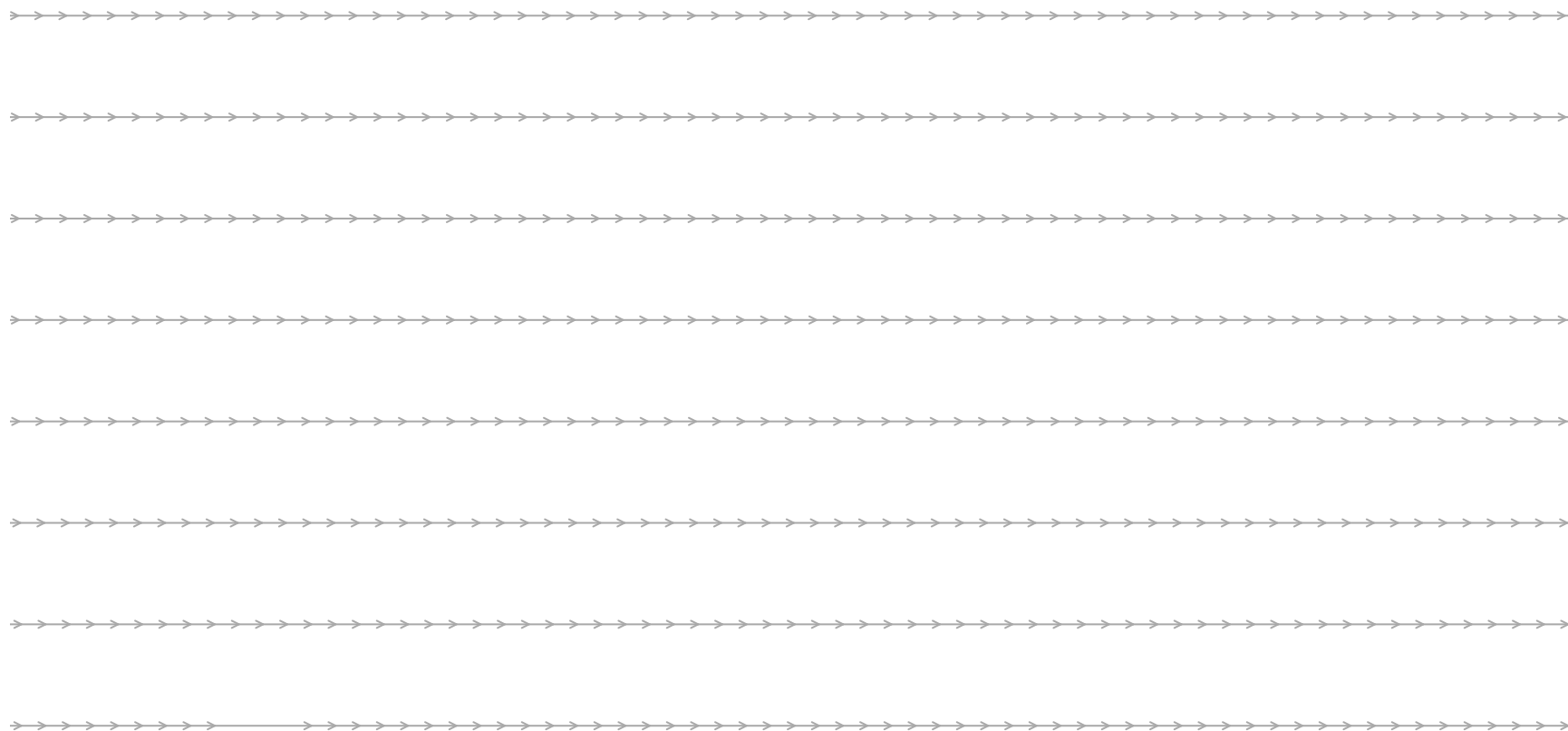

RP1-163G9.2

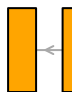

Supplement: Additional file 2 — DMRforPairs output for the comparison of A431-MCF7 and NA17018-NA17105. Please start from the HTML files in each folder. Available via the BMC Bioinformatics website. [file 1471-2105-15-141-S2.zip › 1394847754114233_MOESM2_ESM/A431_MCF7/figures/102.pdf]

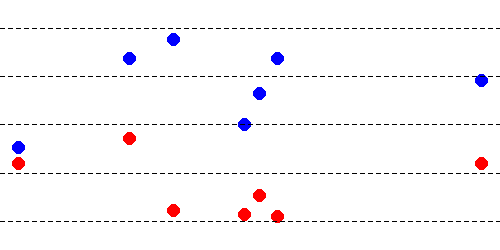

Supplement: Additional file 2 — DMRforPairs output for the comparison of A431-MCF7 and NA17018-NA17105. Please start from the HTML files in each folder. Available via the BMC Bioinformatics website. [file 1471-2105-15-141-S2.zip › 1394847754114233_MOESM2_ESM/A431_MCF7/figures/102.png]

RegionID: 1020, chr1:220921855–220922217–M\_values

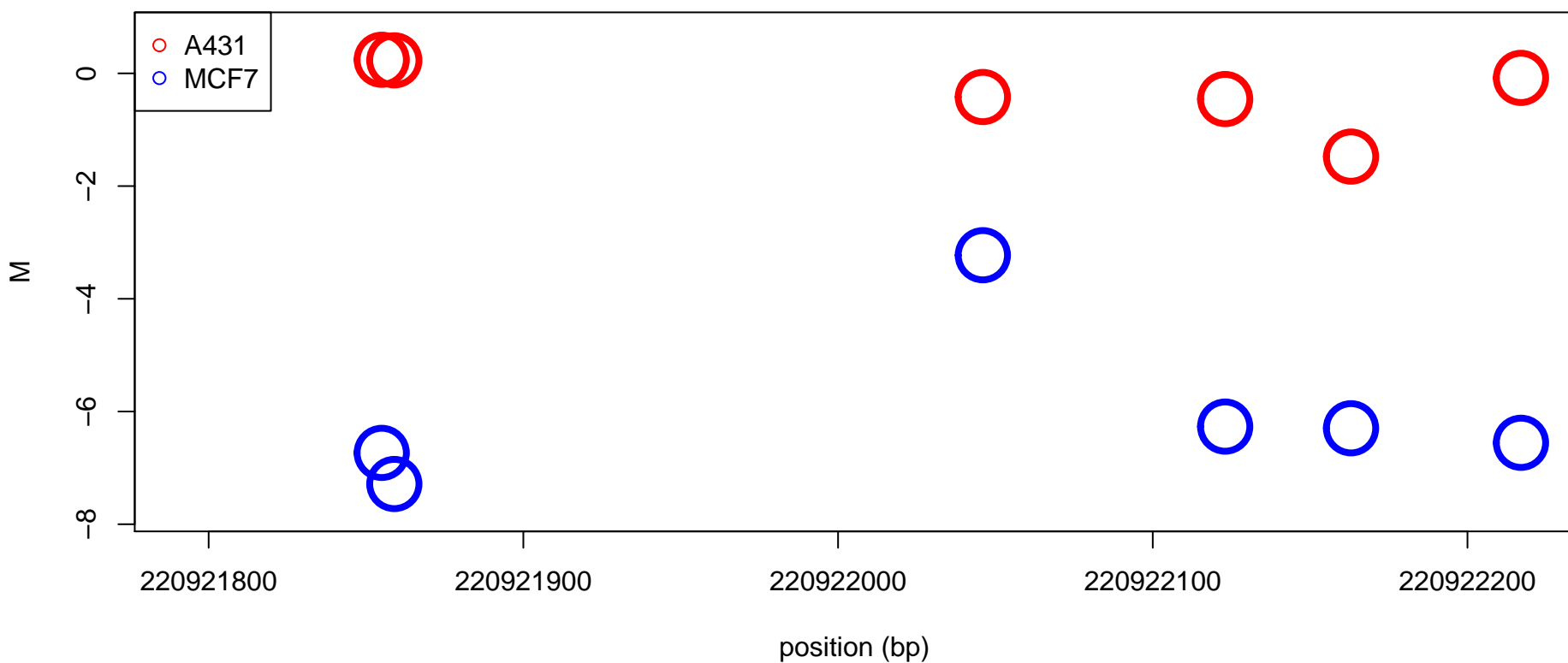

RegionID: 1020, chr1:220921855–220922217–Beta\_values

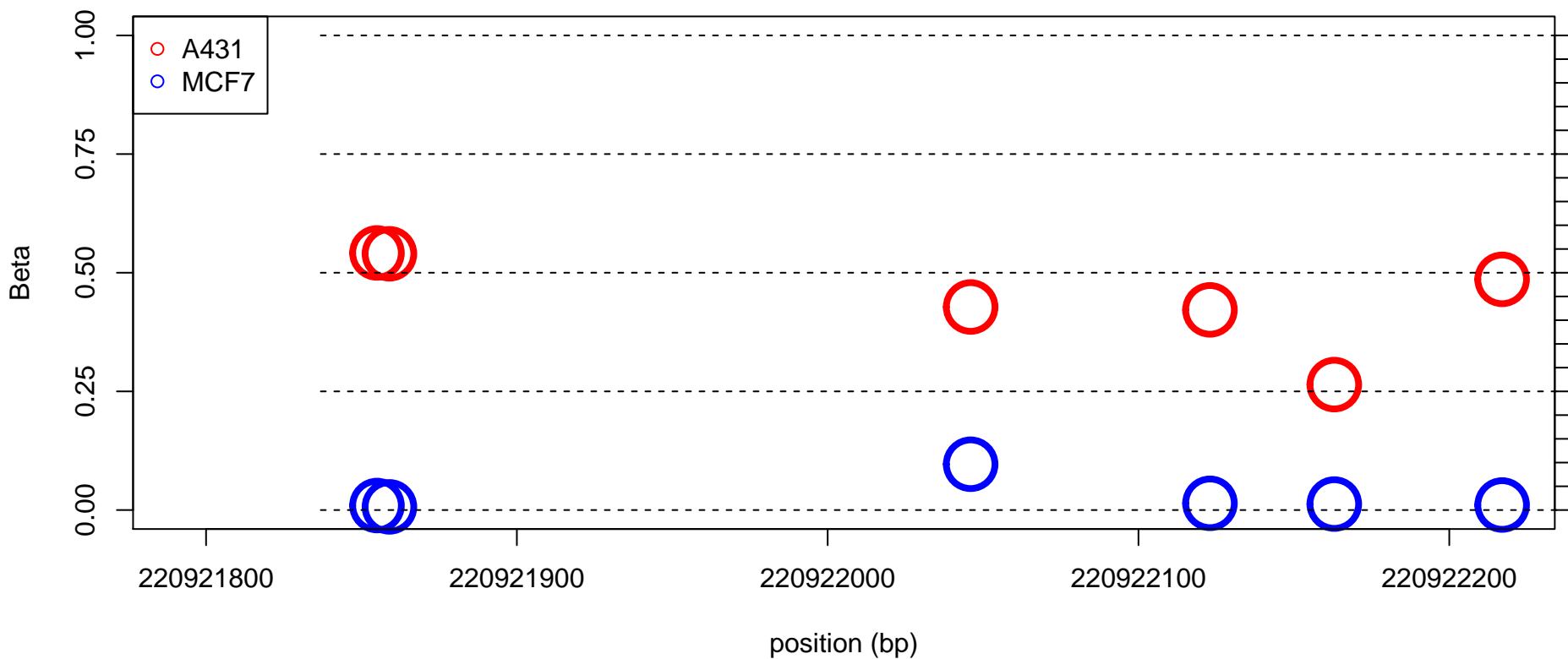

Chromosome 1

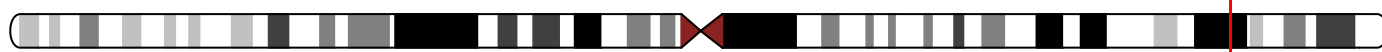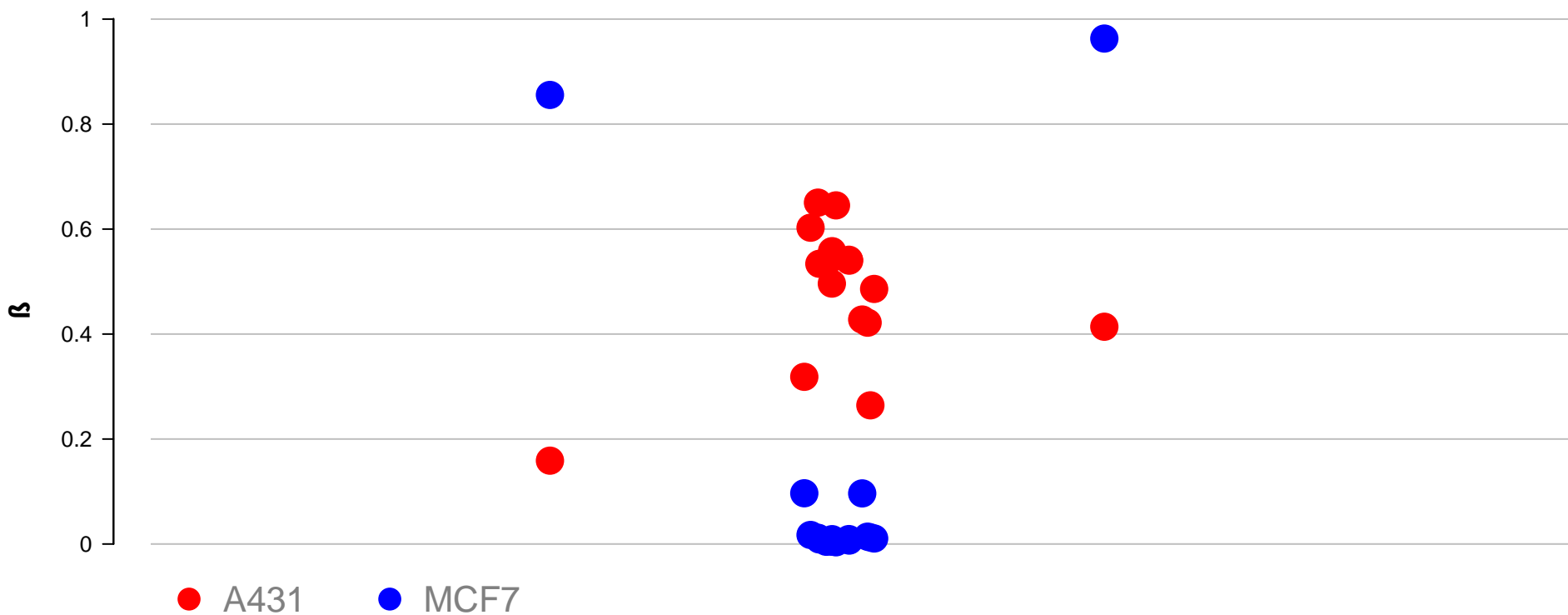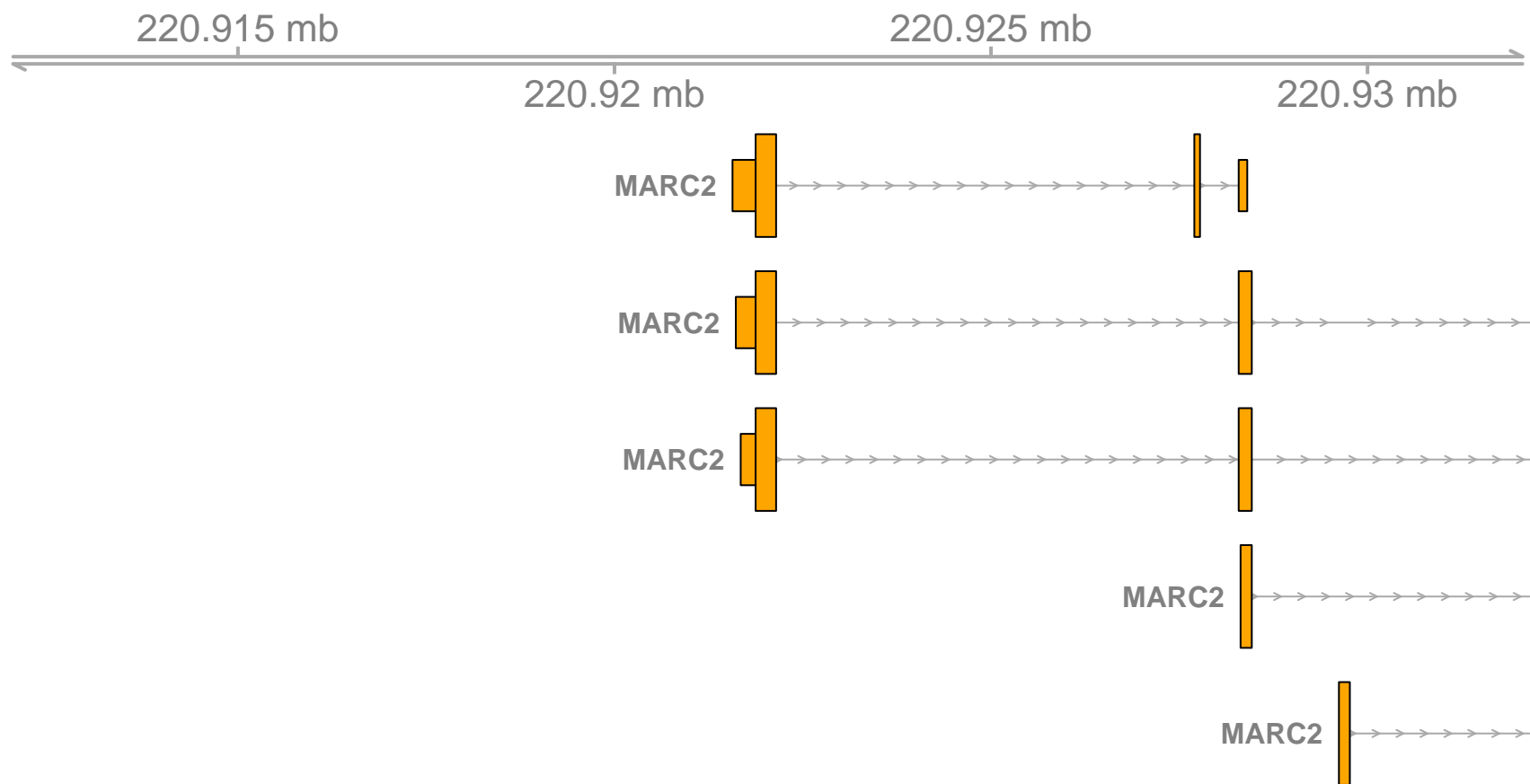

Supplement: Additional file 2 — DMRforPairs output for the comparison of A431-MCF7 and NA17018-NA17105. Please start from the HTML files in each folder. Available via the BMC Bioinformatics website. [file 1471-2105-15-141-S2.zip › 1394847754114233_MOESM2_ESM/A431_MCF7/figures/1020.pdf]

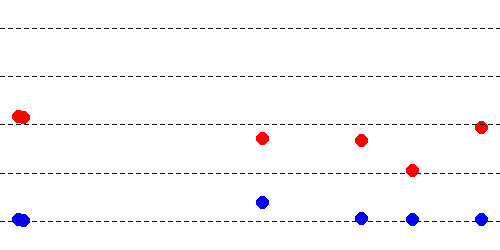

Supplement: Additional file 2 — DMRforPairs output for the comparison of A431-MCF7 and NA17018-NA17105. Please start from the HTML files in each folder. Available via the BMC Bioinformatics website. [file 1471-2105-15-141-S2.zip › 1394847754114233_MOESM2_ESM/A431_MCF7/figures/1020.png]

RegionID: 10202, chr12:6756377-6756815-M\_values

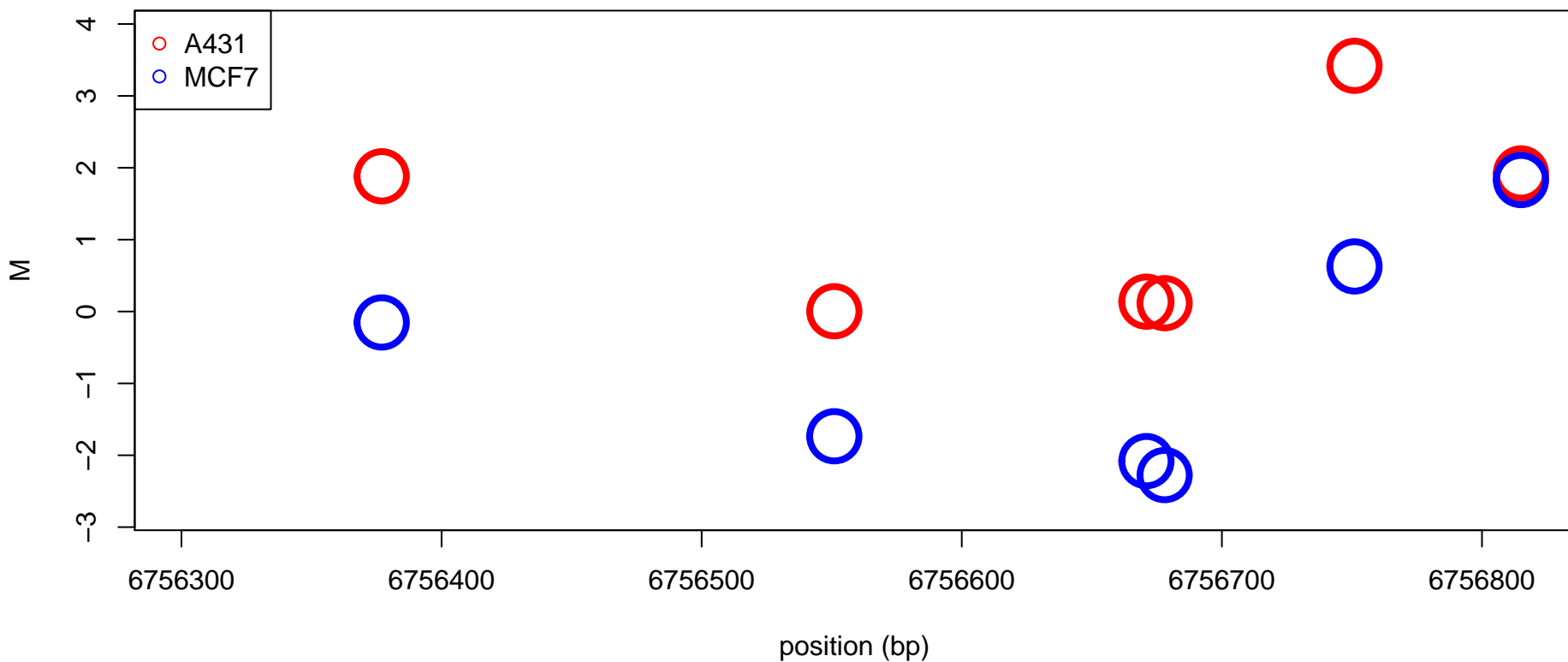

RegionID: 10202, chr12:6756377-6756815-Beta\_values

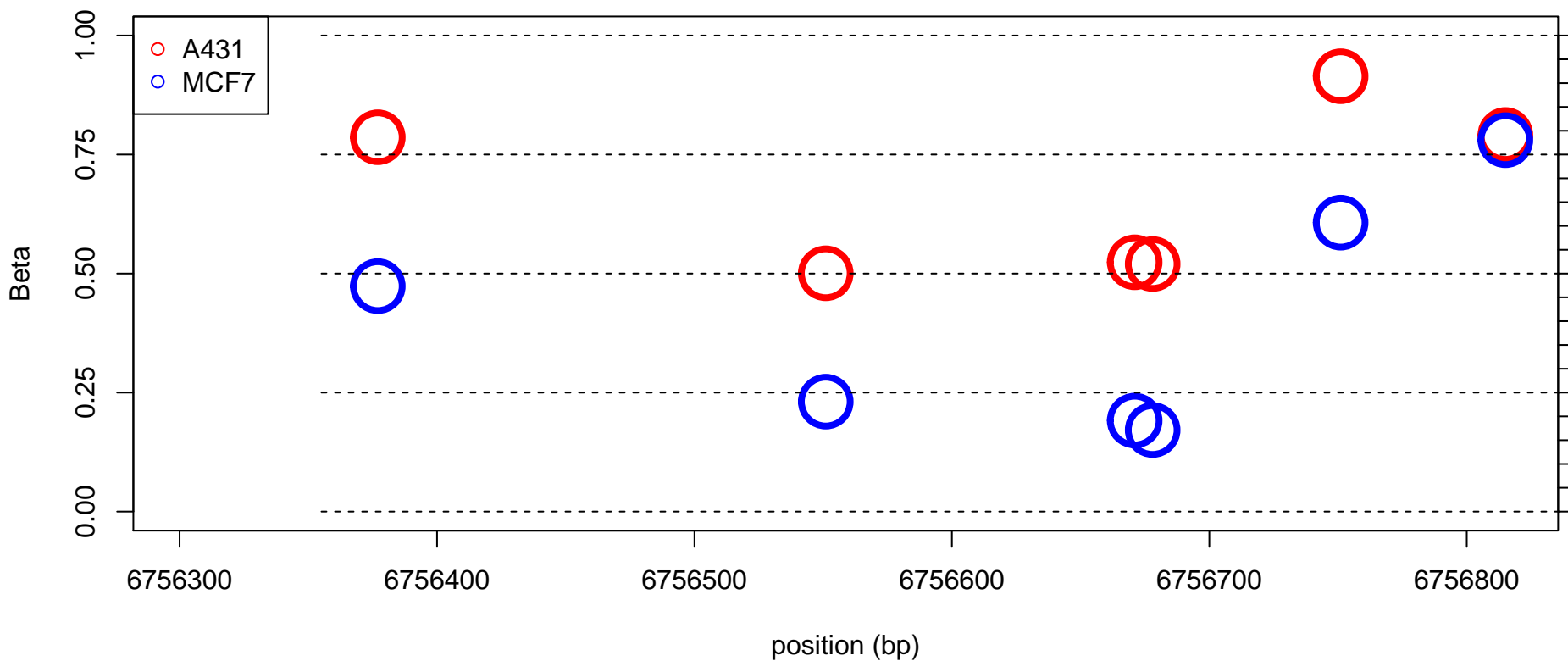

Supplement: Additional file 2 — DMRforPairs output for the comparison of A431-MCF7 and NA17018-NA17105. Please start from the HTML files in each folder. Available via the BMC Bioinformatics website. [file 1471-2105-15-141-S2.zip › 1394847754114233_MOESM2_ESM/A431_MCF7/figures/10202.pdf]

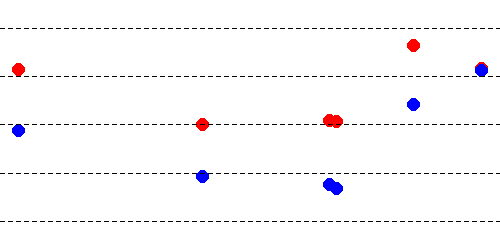

Supplement: Additional file 2 — DMRforPairs output for the comparison of A431-MCF7 and NA17018-NA17105. Please start from the HTML files in each folder. Available via the BMC Bioinformatics website. [file 1471-2105-15-141-S2.zip › 1394847754114233_MOESM2_ESM/A431_MCF7/figures/10202.png]

RegionID: 10206, chr12:6809792-6809939-M\_values

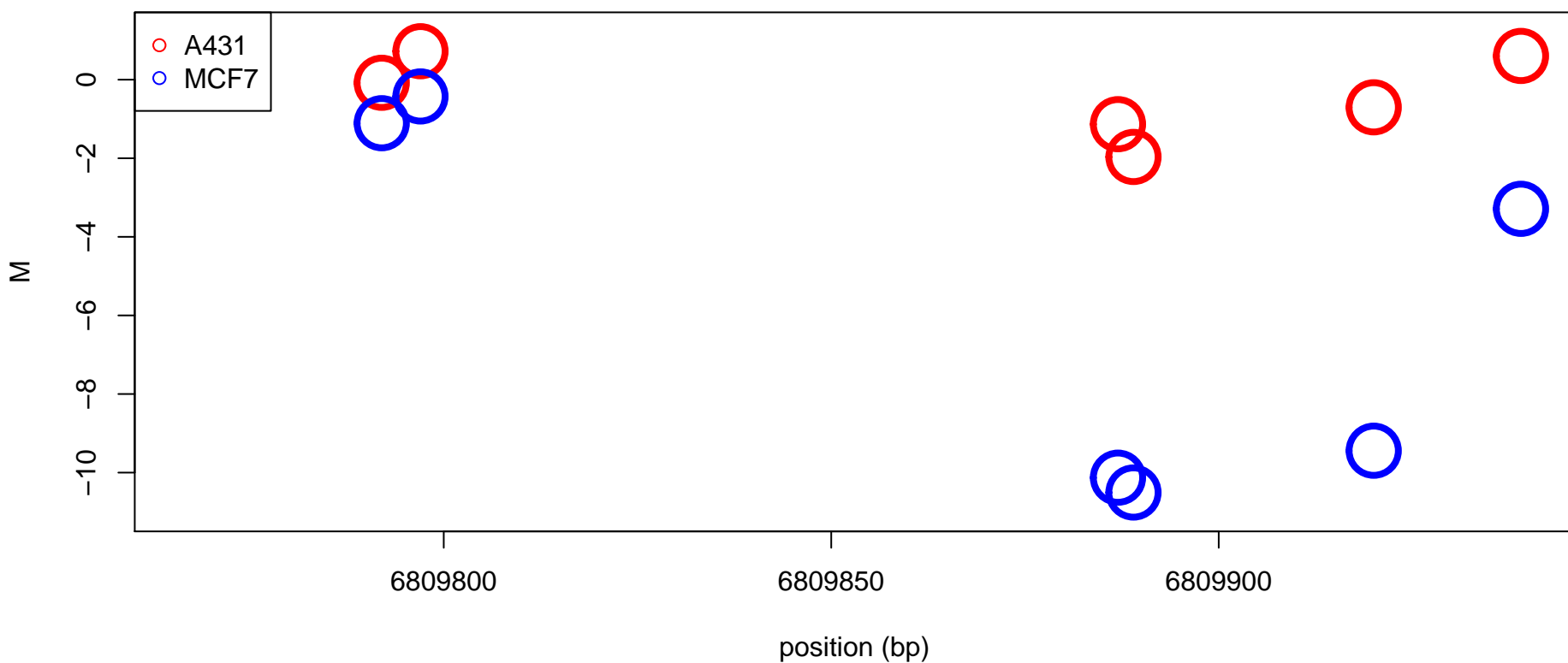

RegionID: 10206, chr12:6809792-6809939-Beta\_values

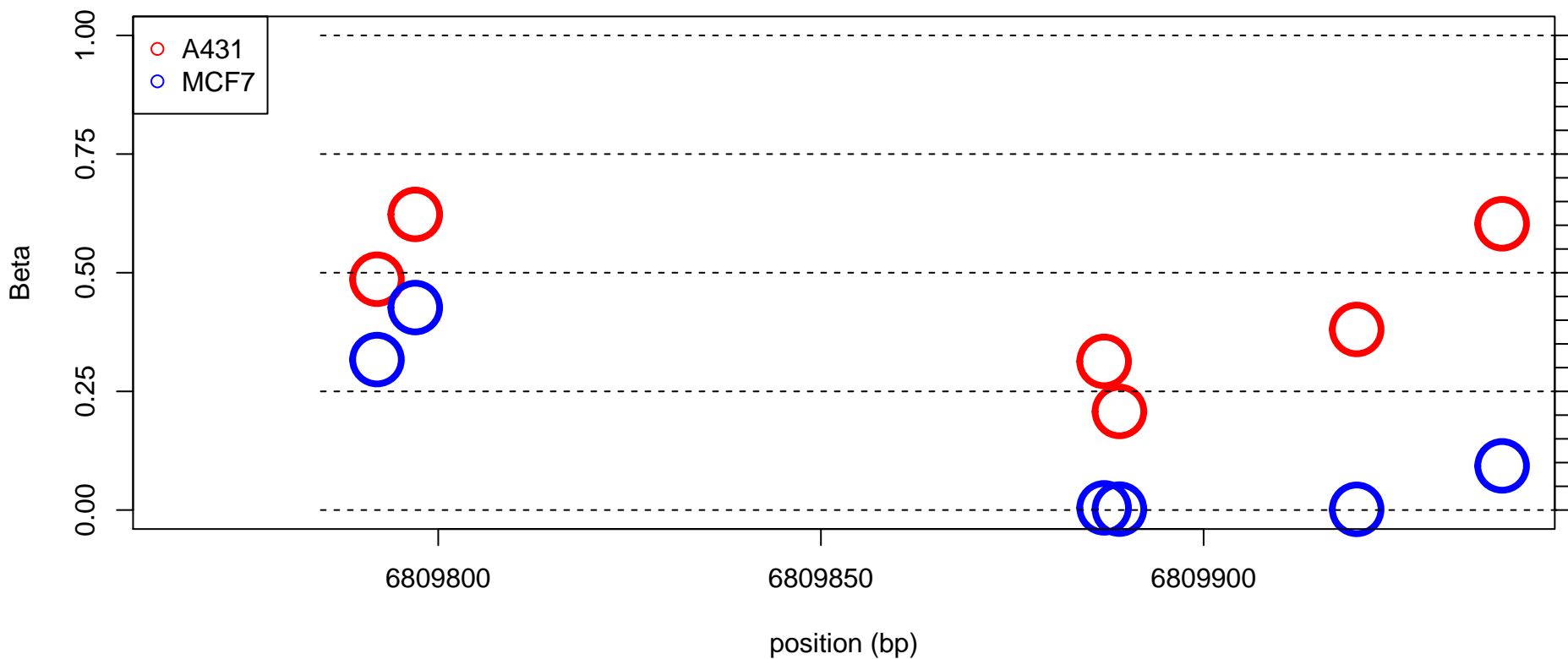

Supplement: Additional file 2 — DMRforPairs output for the comparison of A431-MCF7 and NA17018-NA17105. Please start from the HTML files in each folder. Available via the BMC Bioinformatics website. [file 1471-2105-15-141-S2.zip › 1394847754114233_MOESM2_ESM/A431_MCF7/figures/10206.pdf]

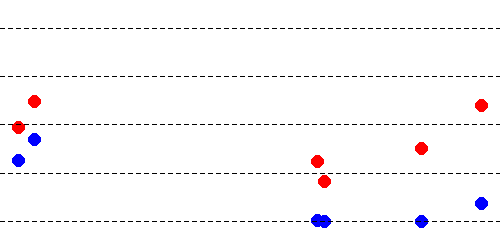

Supplement: Additional file 2 — DMRforPairs output for the comparison of A431-MCF7 and NA17018-NA17105. Please start from the HTML files in each folder. Available via the BMC Bioinformatics website. [file 1471-2105-15-141-S2.zip › 1394847754114233_MOESM2_ESM/A431_MCF7/figures/10206.png]

RegionID: 1022, chr1:221057558–221057808–M\_values

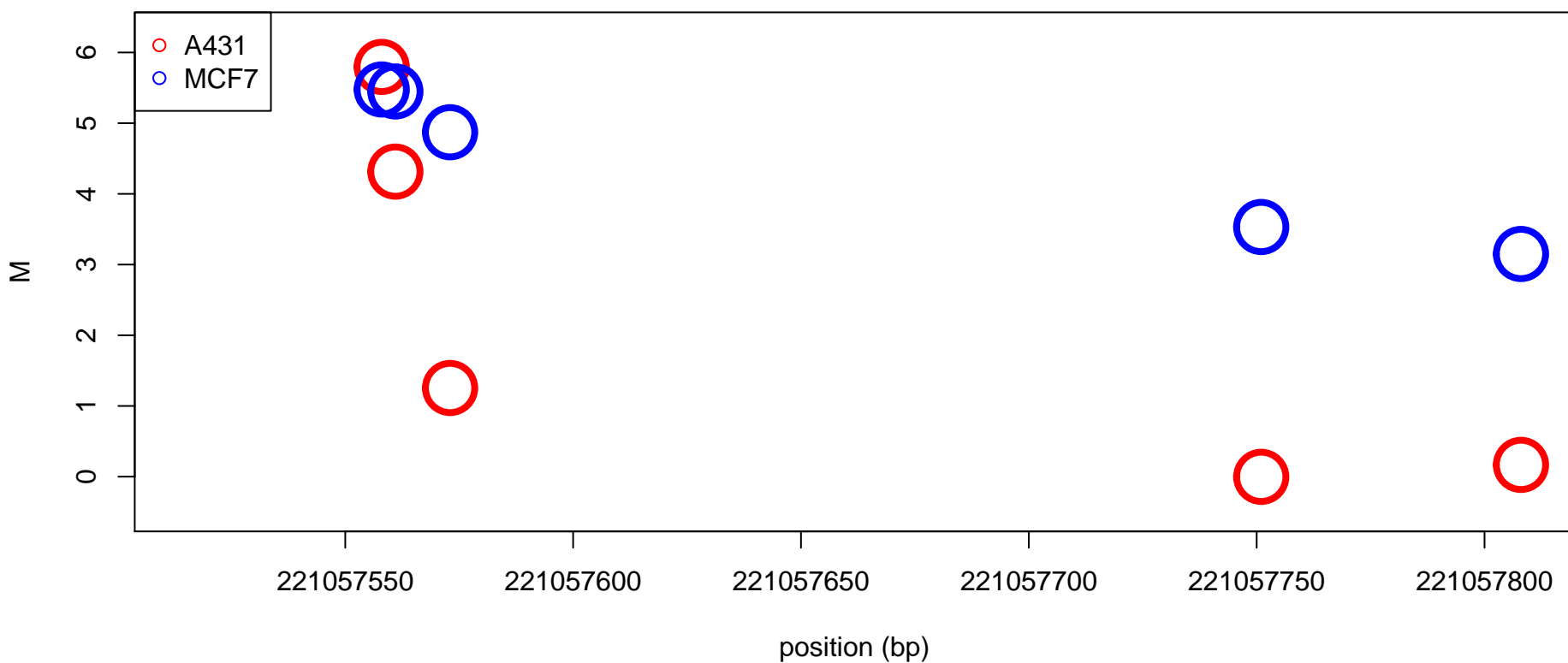

RegionID: 1022, chr1:221057558–221057808–Beta\_values

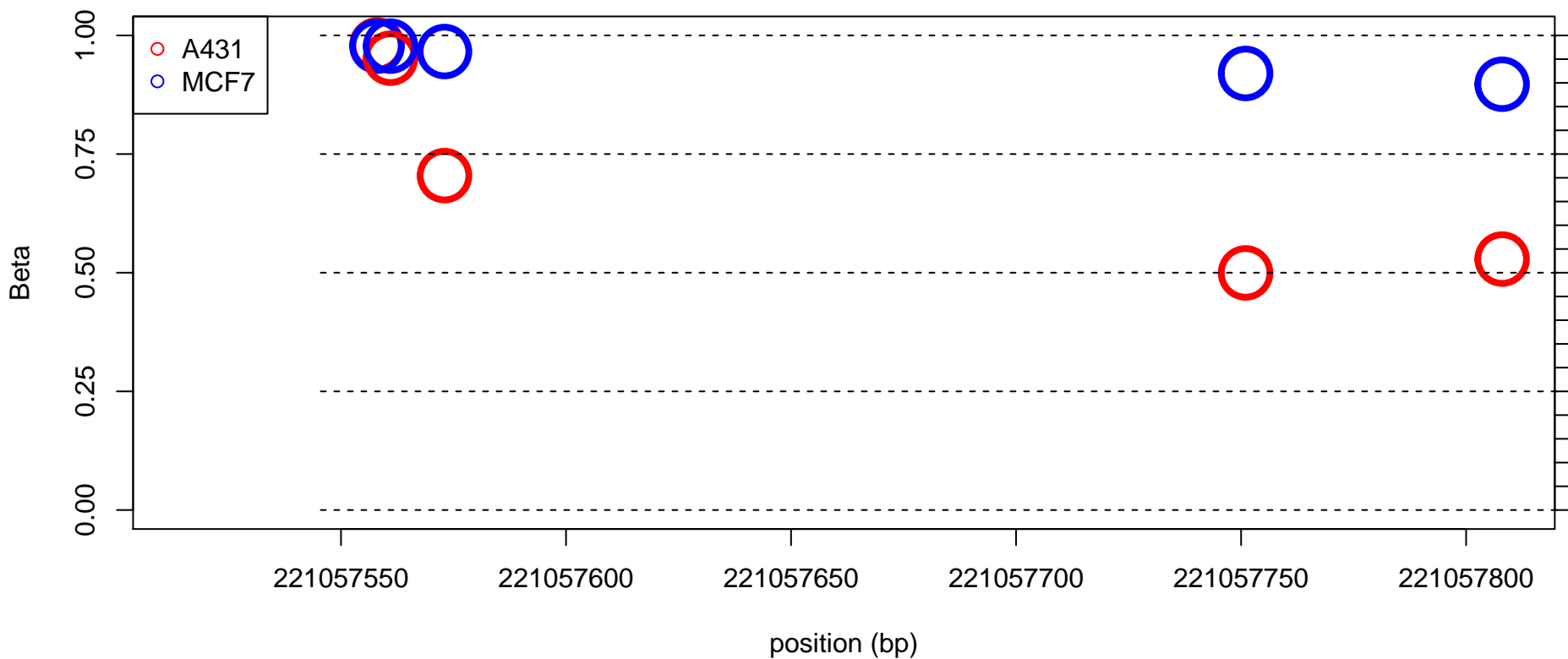

Supplement: Additional file 2 — DMRforPairs output for the comparison of A431-MCF7 and NA17018-NA17105. Please start from the HTML files in each folder. Available via the BMC Bioinformatics website. [file 1471-2105-15-141-S2.zip › 1394847754114233_MOESM2_ESM/A431_MCF7/figures/1022.pdf]

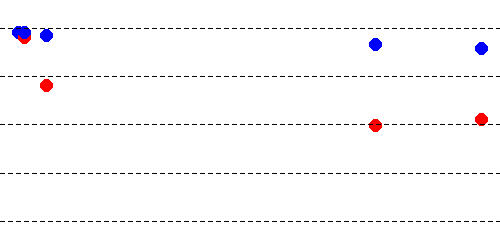

Supplement: Additional file 2 — DMRforPairs output for the comparison of A431-MCF7 and NA17018-NA17105. Please start from the HTML files in each folder. Available via the BMC Bioinformatics website. [file 1471-2105-15-141-S2.zip › 1394847754114233_MOESM2_ESM/A431_MCF7/figures/1022.png]

RegionID: 10221, chr12:7055452-7056008-M\_values

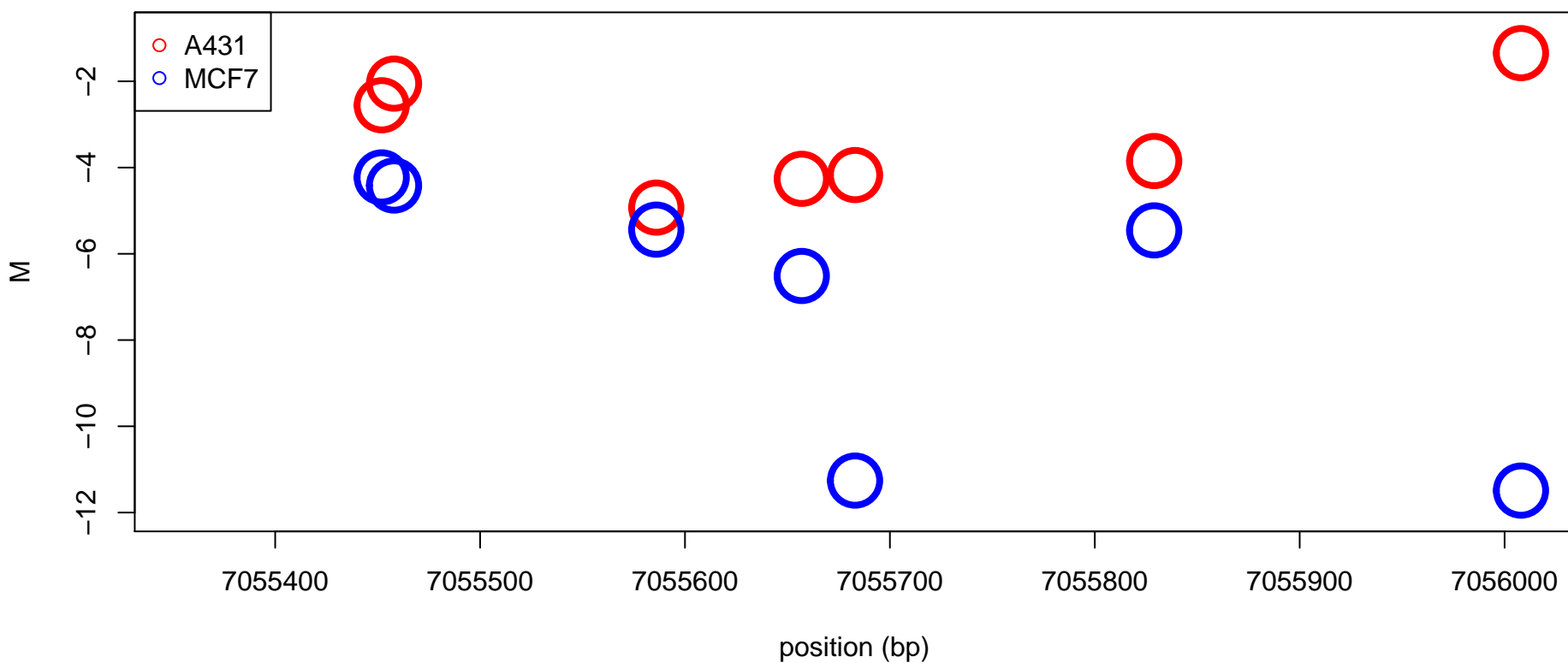

RegionID: 10221, chr12:7055452-7056008-Beta\_values

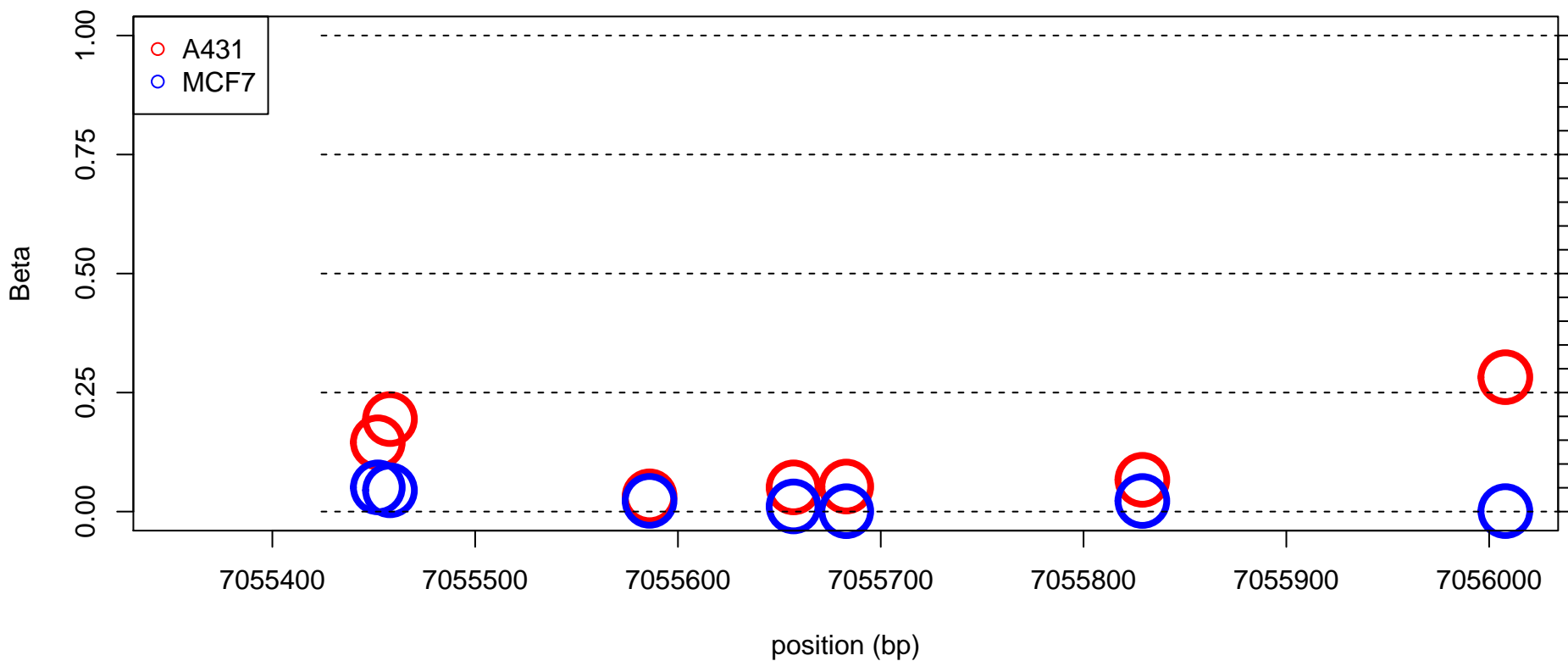

Chromosome 12

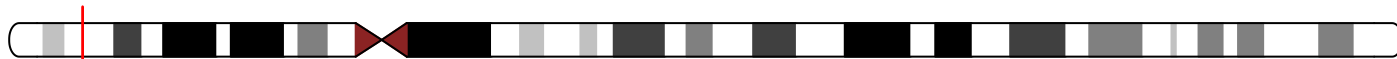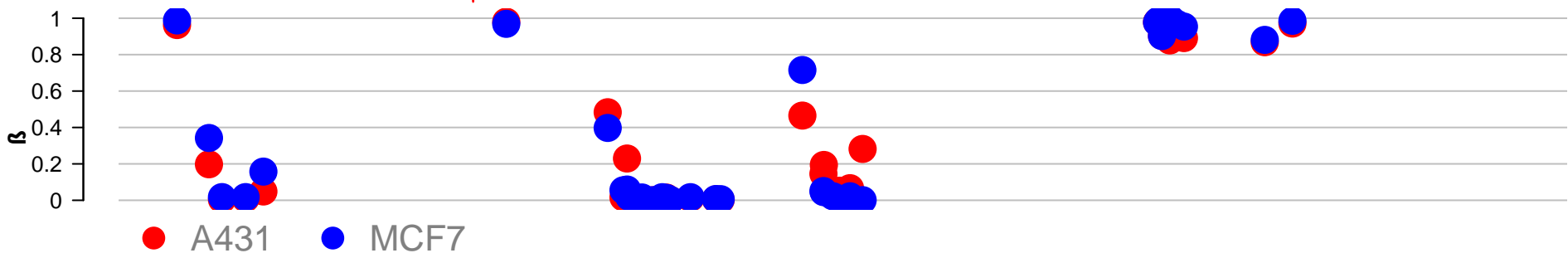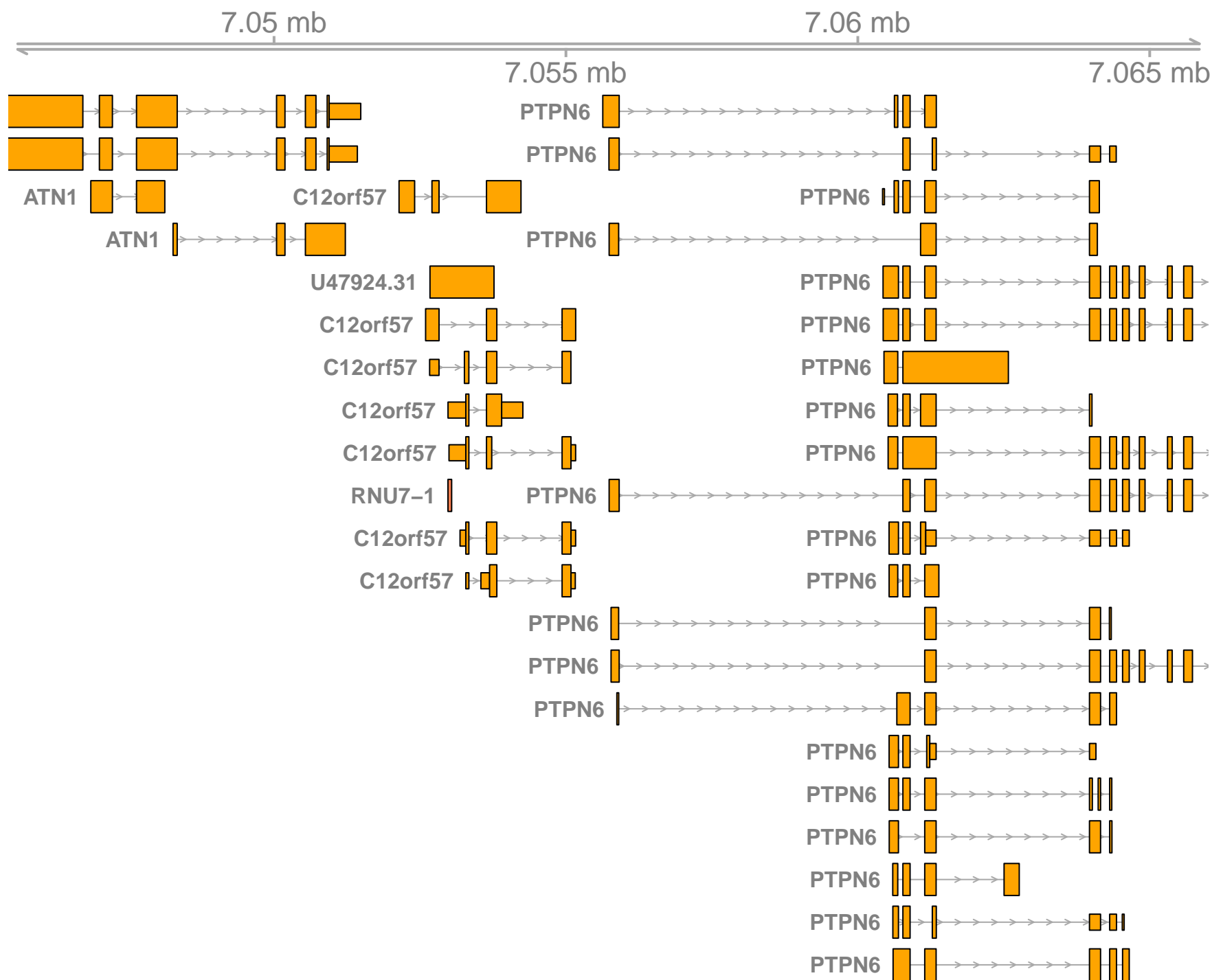

Supplement: Additional file 2 — DMRforPairs output for the comparison of A431-MCF7 and NA17018-NA17105. Please start from the HTML files in each folder. Available via the BMC Bioinformatics website. [file 1471-2105-15-141-S2.zip › 1394847754114233_MOESM2_ESM/A431_MCF7/figures/10221.pdf]

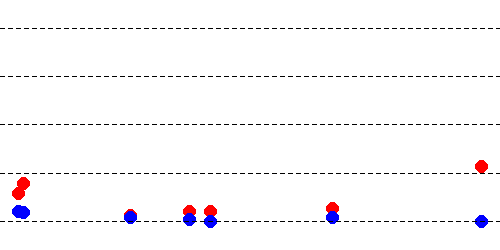

Supplement: Additional file 2 — DMRforPairs output for the comparison of A431-MCF7 and NA17018-NA17105. Please start from the HTML files in each folder. Available via the BMC Bioinformatics website. [file 1471-2105-15-141-S2.zip › 1394847754114233_MOESM2_ESM/A431_MCF7/figures/10221.png]

RegionID: 10228, chr12:7858592-7858848-M\_values

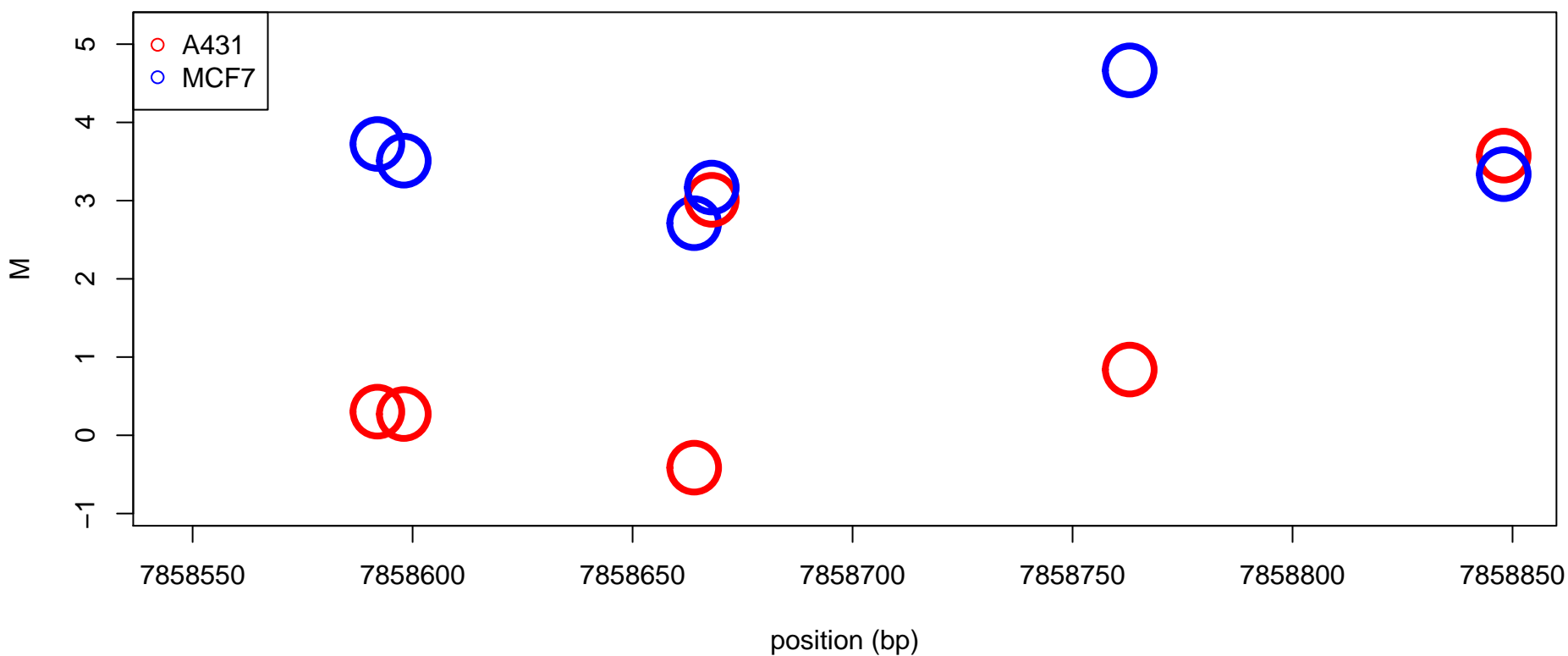

RegionID: 10228, chr12:7858592-7858848-Beta\_values

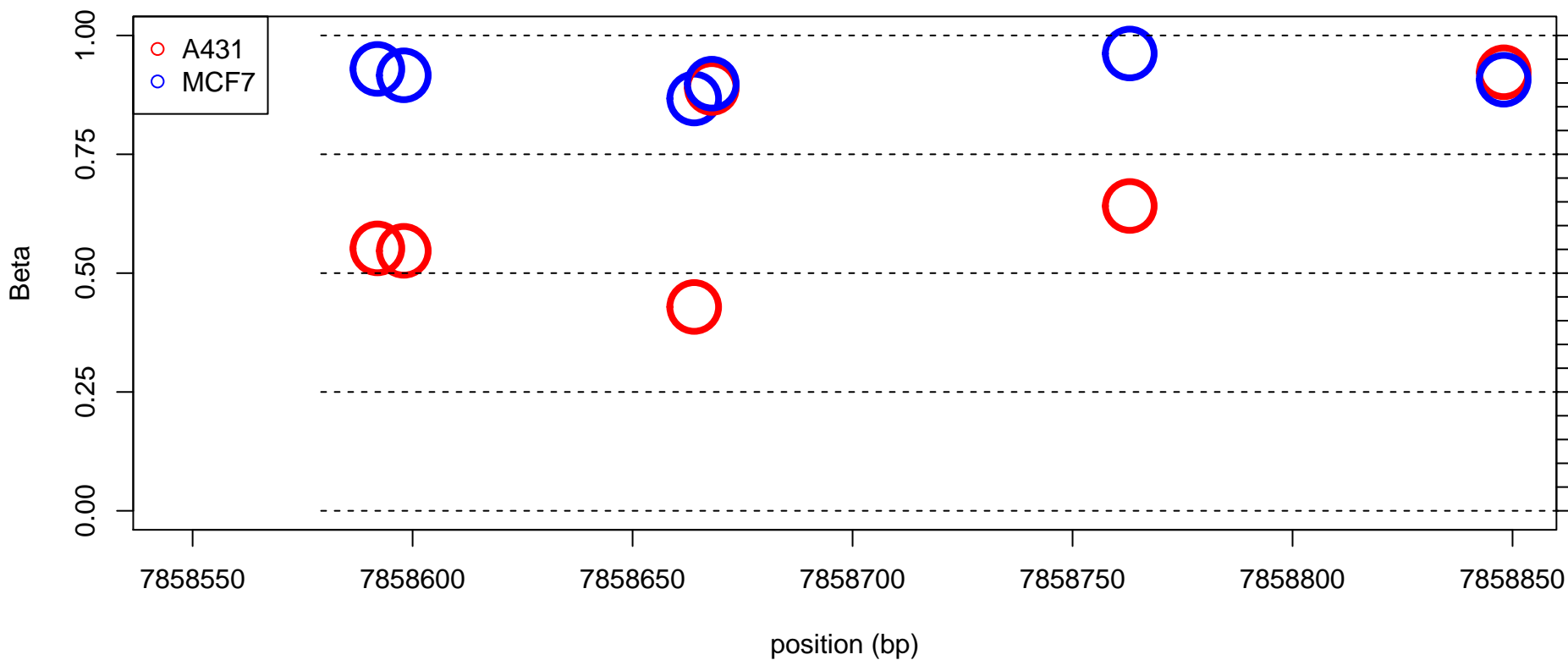

Supplement: Additional file 2 — DMRforPairs output for the comparison of A431-MCF7 and NA17018-NA17105. Please start from the HTML files in each folder. Available via the BMC Bioinformatics website. [file 1471-2105-15-141-S2.zip › 1394847754114233_MOESM2_ESM/A431_MCF7/figures/10228.pdf]

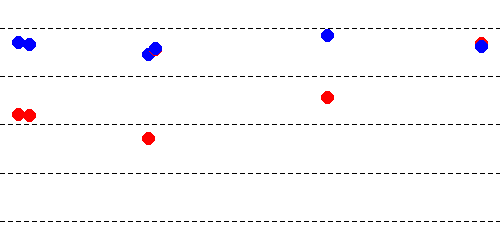

Supplement: Additional file 2 — DMRforPairs output for the comparison of A431-MCF7 and NA17018-NA17105. Please start from the HTML files in each folder. Available via the BMC Bioinformatics website. [file 1471-2105-15-141-S2.zip › 1394847754114233_MOESM2_ESM/A431_MCF7/figures/10228.png]

RegionID: 10229, chr12:8025394–8025646–M\_values

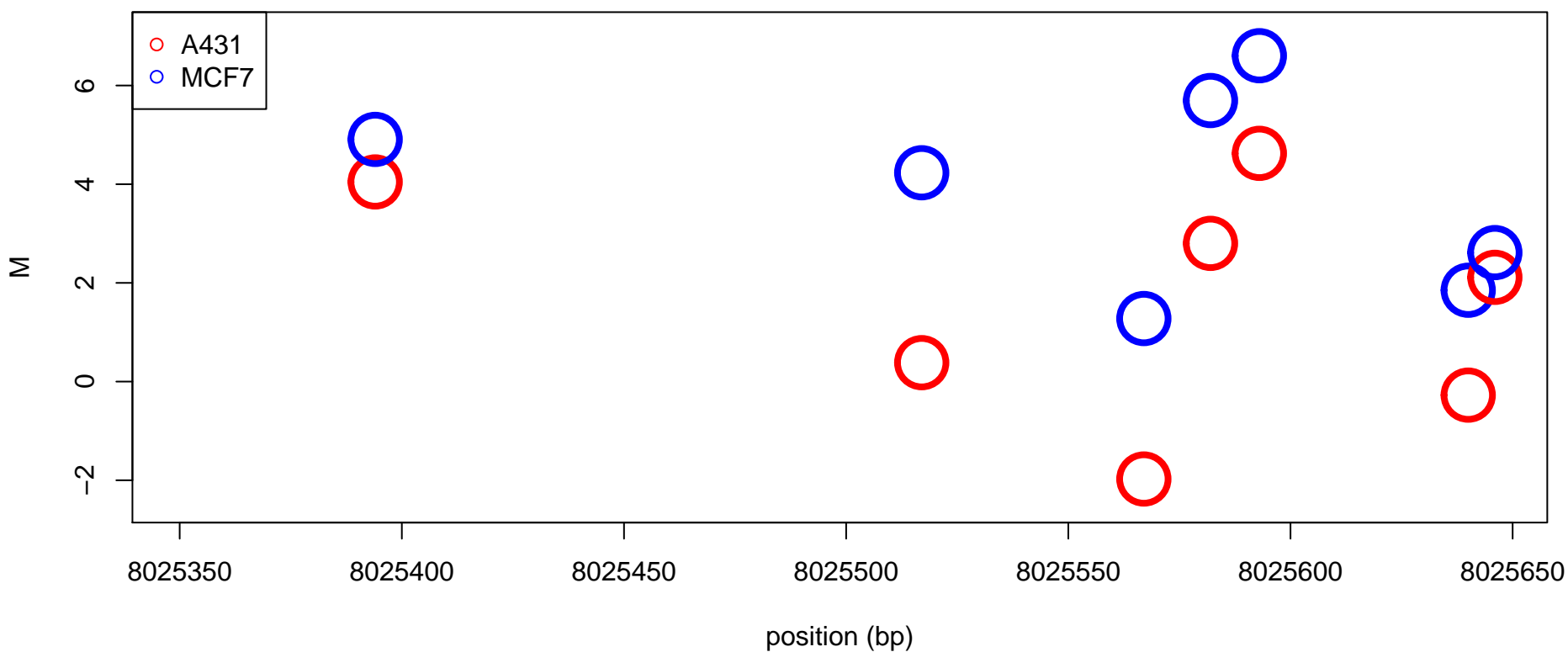

RegionID: 10229, chr12:8025394–8025646–Beta\_values

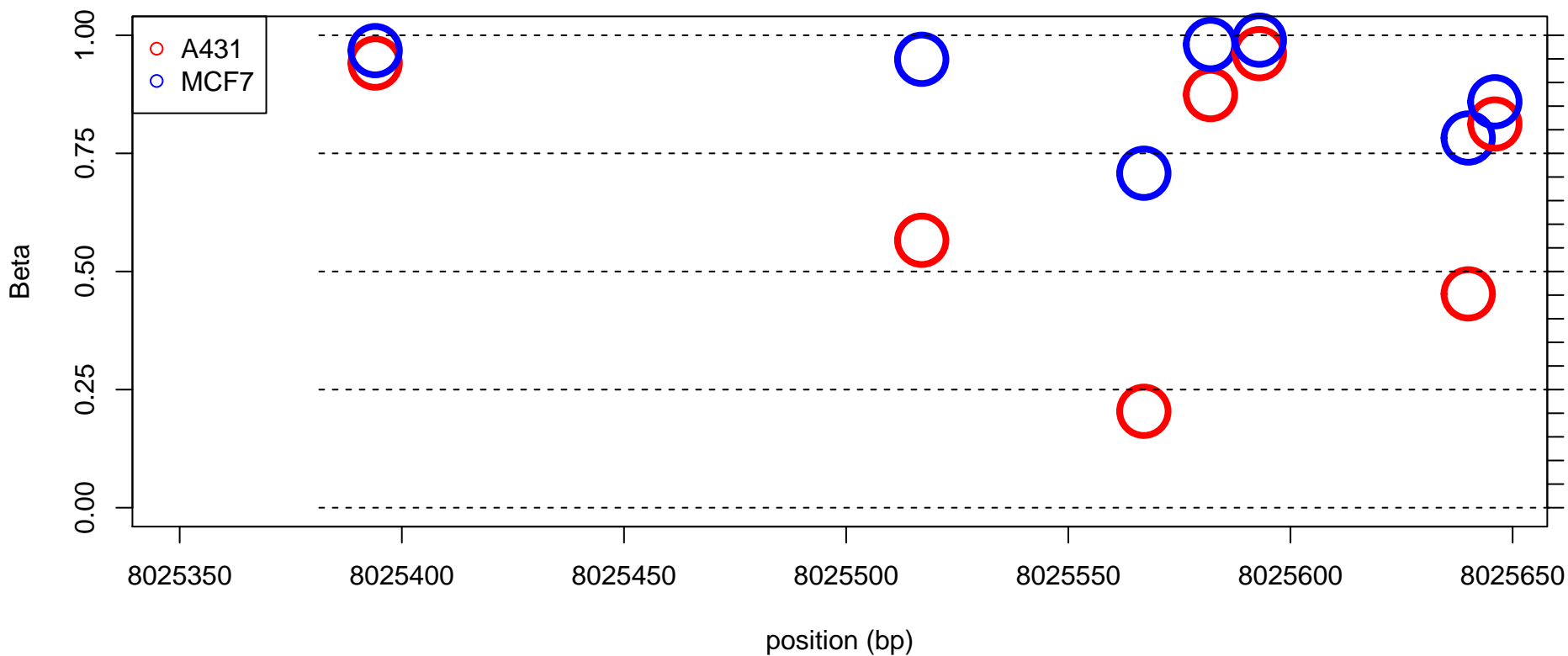

Supplement: Additional file 2 — DMRforPairs output for the comparison of A431-MCF7 and NA17018-NA17105. Please start from the HTML files in each folder. Available via the BMC Bioinformatics website. [file 1471-2105-15-141-S2.zip › 1394847754114233_MOESM2_ESM/A431_MCF7/figures/10229.pdf]

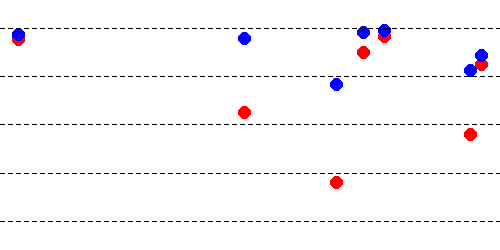

Supplement: Additional file 2 — DMRforPairs output for the comparison of A431-MCF7 and NA17018-NA17105. Please start from the HTML files in each folder. Available via the BMC Bioinformatics website. [file 1471-2105-15-141-S2.zip › 1394847754114233_MOESM2_ESM/A431_MCF7/figures/10229.png]
