# Supplementary figures and images for: DMRforPairs: identifying Differentially Methylated Regions between unique samples using array based methylation profiles (part 1 of 2)
Source: BMC Bioinformatics. 2014 May 15;15:141. doi: 10.1186/1471-2105-15-141 (PMC4046028; doi:10.1186/1471-2105-15-141)

RegionID: 100, chr1:3028813–3029070–M\_values

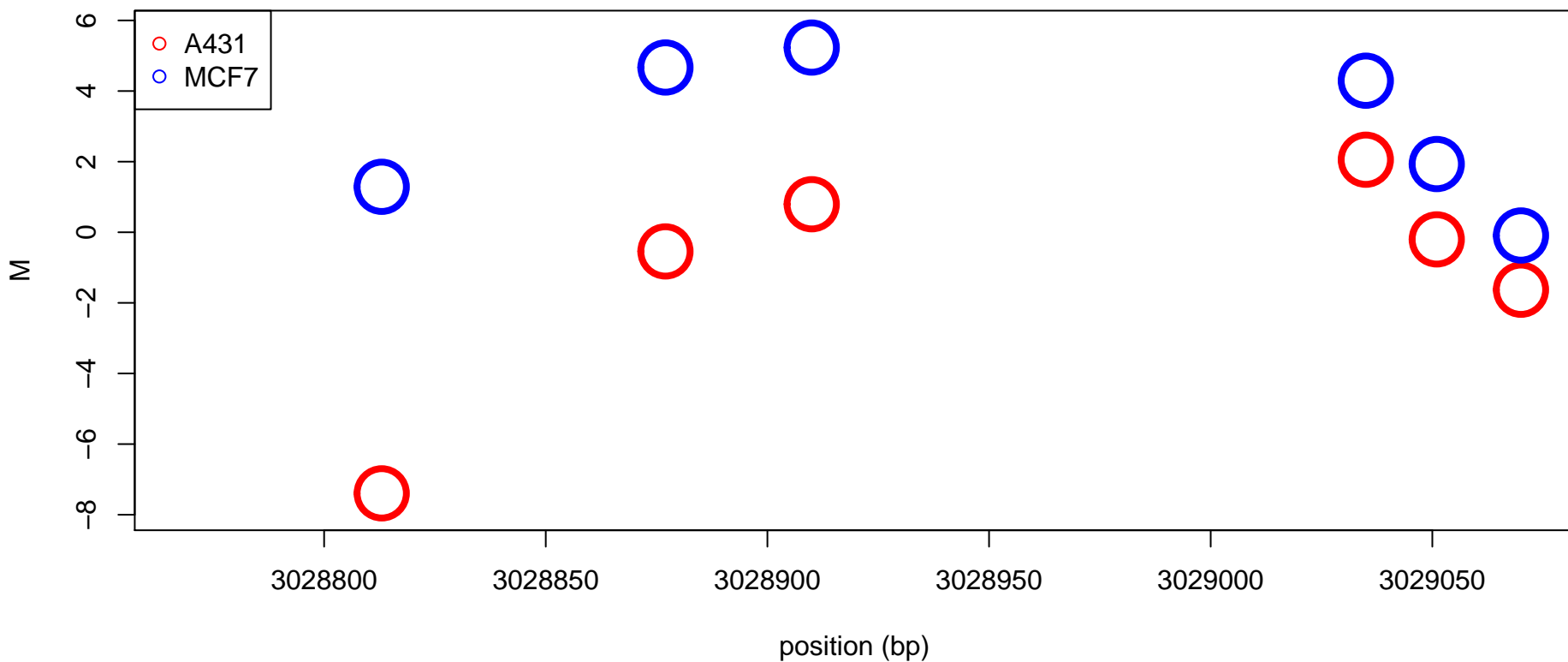

RegionID: 100, chr1:3028813–3029070–Beta\_values

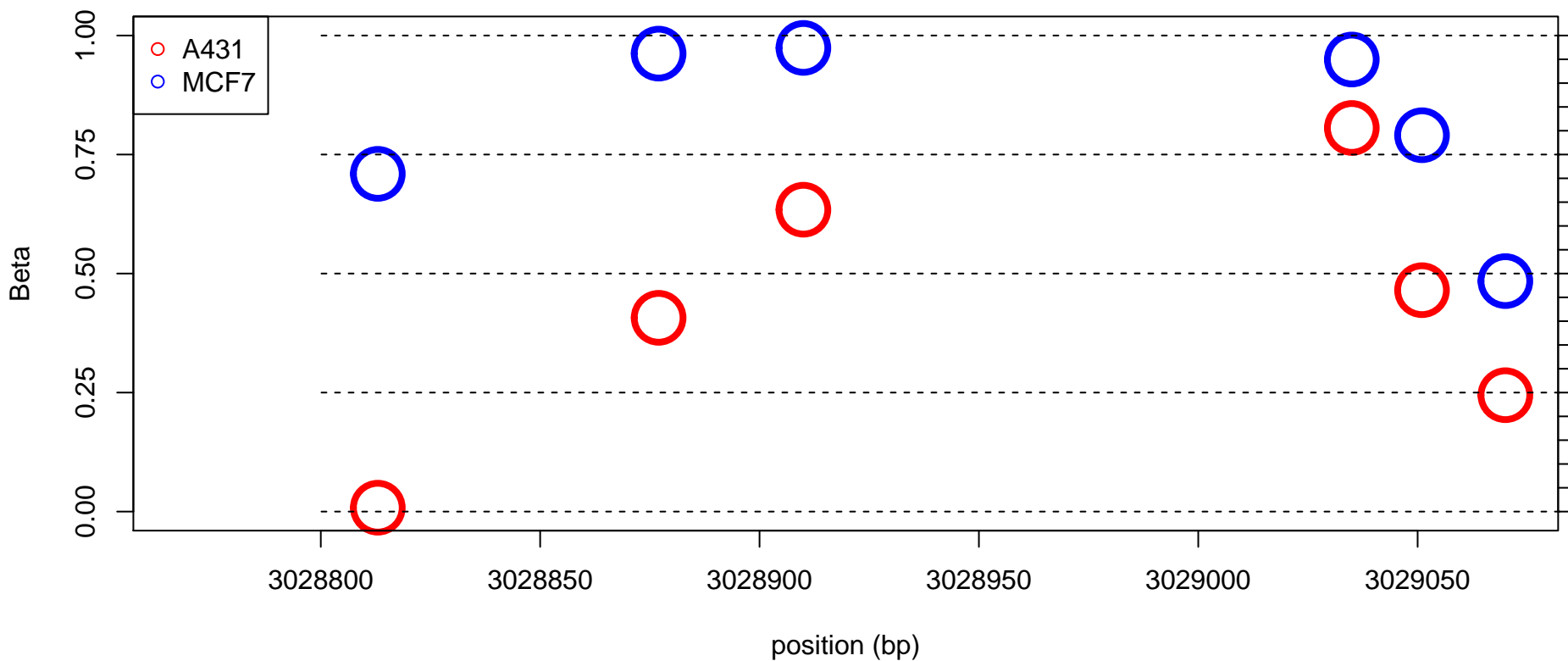

Supplement: Additional file 2 — DMRforPairs output for the comparison of A431-MCF7 and NA17018-NA17105. Please start from the HTML files in each folder. Available via the BMC Bioinformatics website. [file 1471-2105-15-141-S2.zip › 1394847754114233_MOESM2_ESM/A431_MCF7/figures/100.pdf]

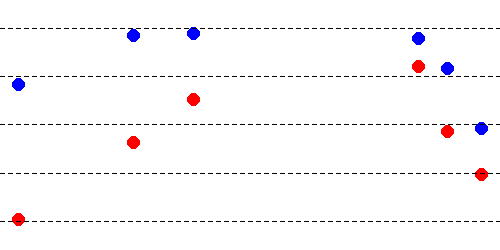

Supplement: Additional file 2 — DMRforPairs output for the comparison of A431-MCF7 and NA17018-NA17105. Please start from the HTML files in each folder. Available via the BMC Bioinformatics website. [file 1471-2105-15-141-S2.zip › 1394847754114233_MOESM2_ESM/A431_MCF7/figures/100.png]

RegionID: 10008, chr12:111126960–111127139–M\_values

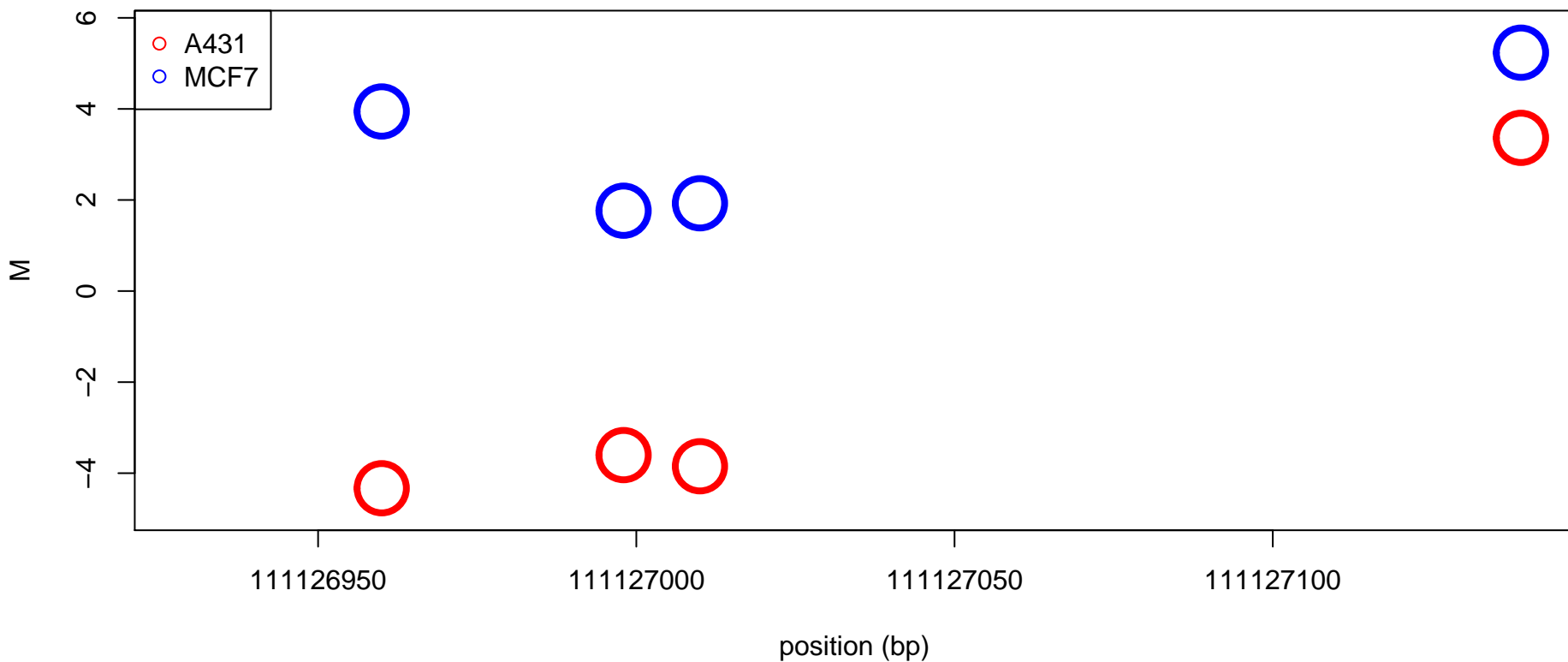

RegionID: 10008, chr12:111126960–111127139–Beta\_values

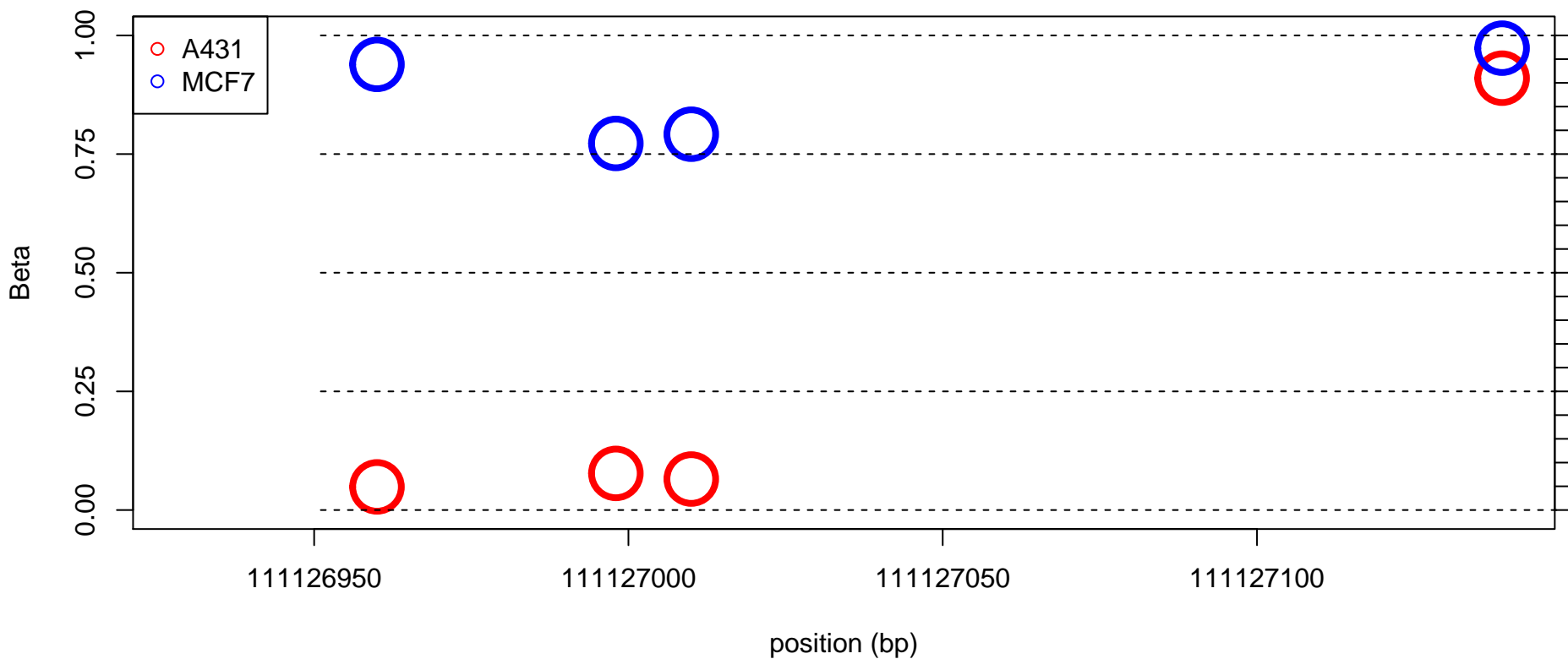

Supplement: Additional file 2 — DMRforPairs output for the comparison of A431-MCF7 and NA17018-NA17105. Please start from the HTML files in each folder. Available via the BMC Bioinformatics website. [file 1471-2105-15-141-S2.zip › 1394847754114233_MOESM2_ESM/A431_MCF7/figures/10008.pdf]

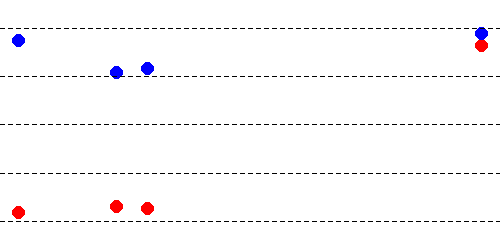

Supplement: Additional file 2 — DMRforPairs output for the comparison of A431-MCF7 and NA17018-NA17105. Please start from the HTML files in each folder. Available via the BMC Bioinformatics website. [file 1471-2105-15-141-S2.zip › 1394847754114233_MOESM2_ESM/A431_MCF7/figures/10008.png]

RegionID: 10010, chr12:111181439–111181819–M\_values

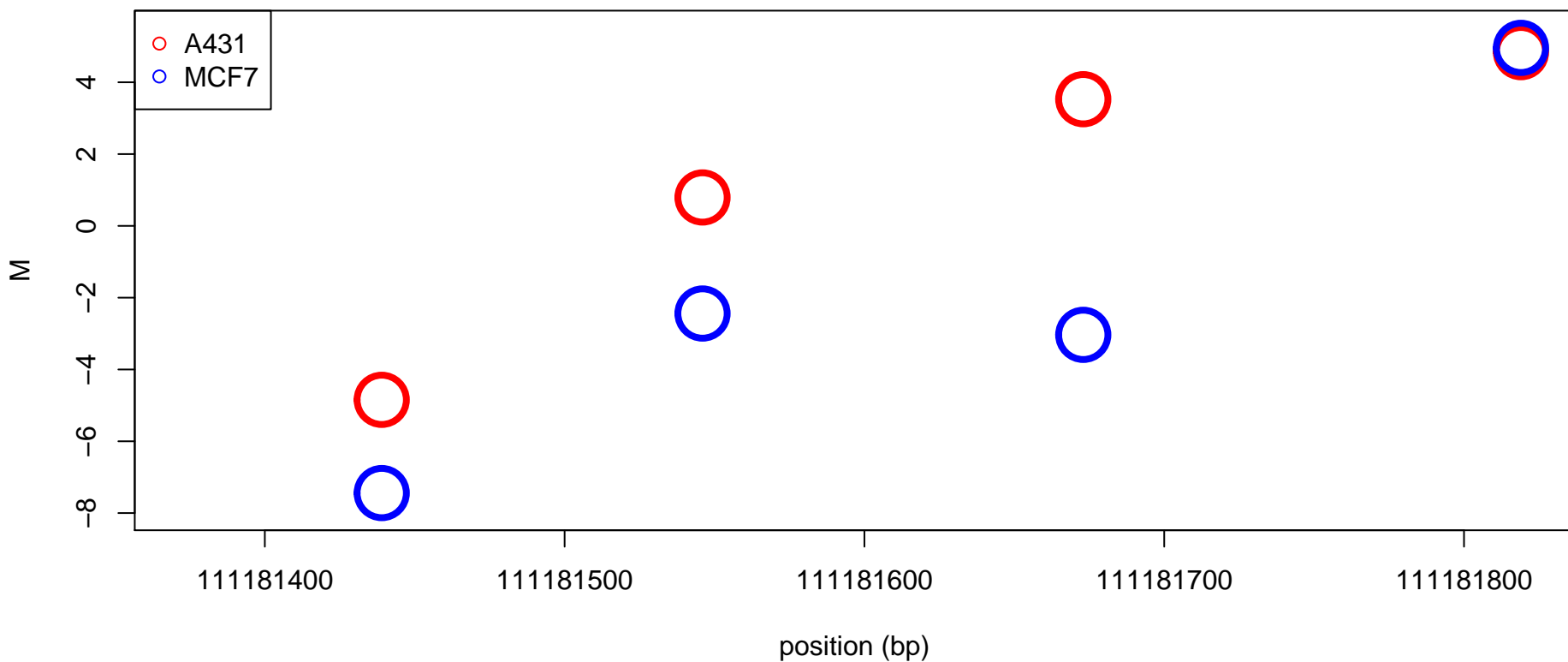

RegionID: 10010, chr12:111181439–111181819–Beta\_values

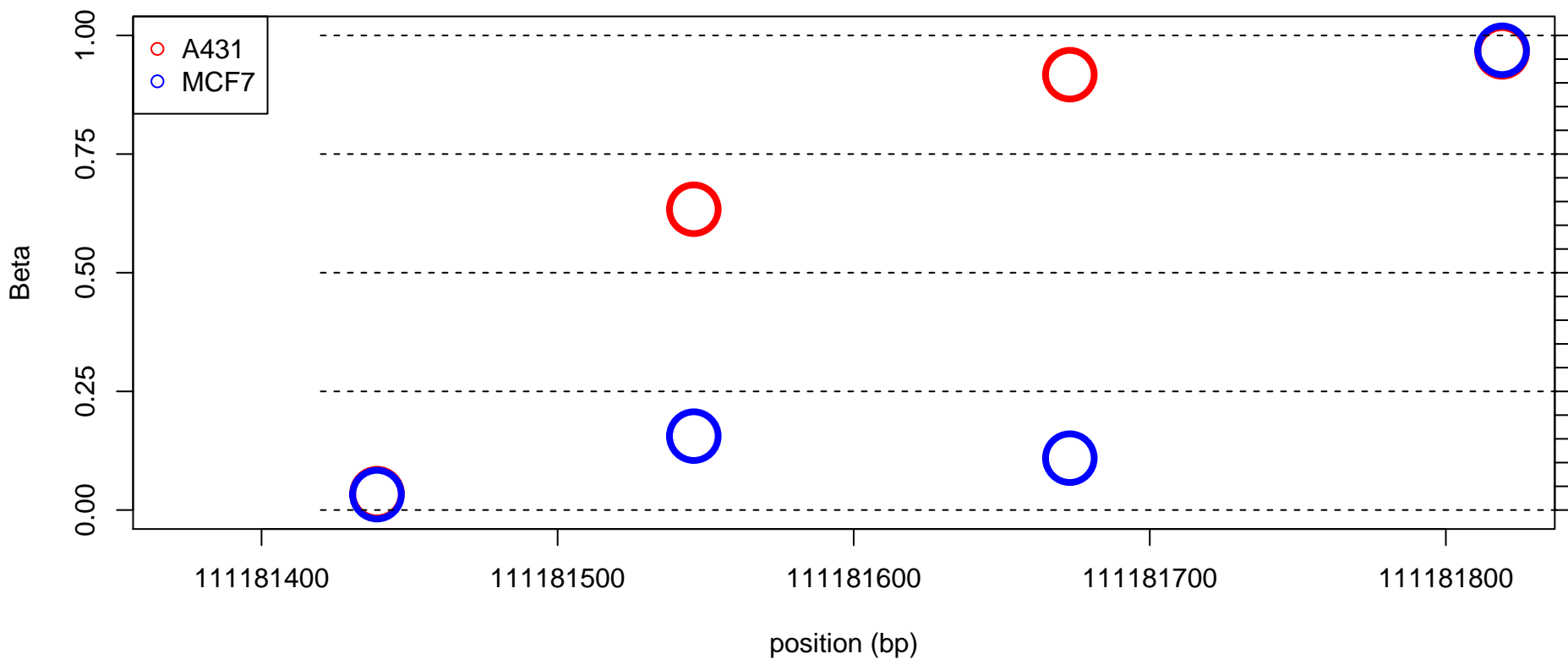

Supplement: Additional file 2 — DMRforPairs output for the comparison of A431-MCF7 and NA17018-NA17105. Please start from the HTML files in each folder. Available via the BMC Bioinformatics website. [file 1471-2105-15-141-S2.zip › 1394847754114233_MOESM2_ESM/A431_MCF7/figures/10010.pdf]

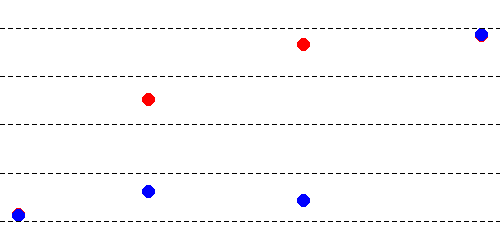

Supplement: Additional file 2 — DMRforPairs output for the comparison of A431-MCF7 and NA17018-NA17105. Please start from the HTML files in each folder. Available via the BMC Bioinformatics website. [file 1471-2105-15-141-S2.zip › 1394847754114233_MOESM2_ESM/A431_MCF7/figures/10010.png]

RegionID: 10011, chr12:111284527-111284806-M\_values

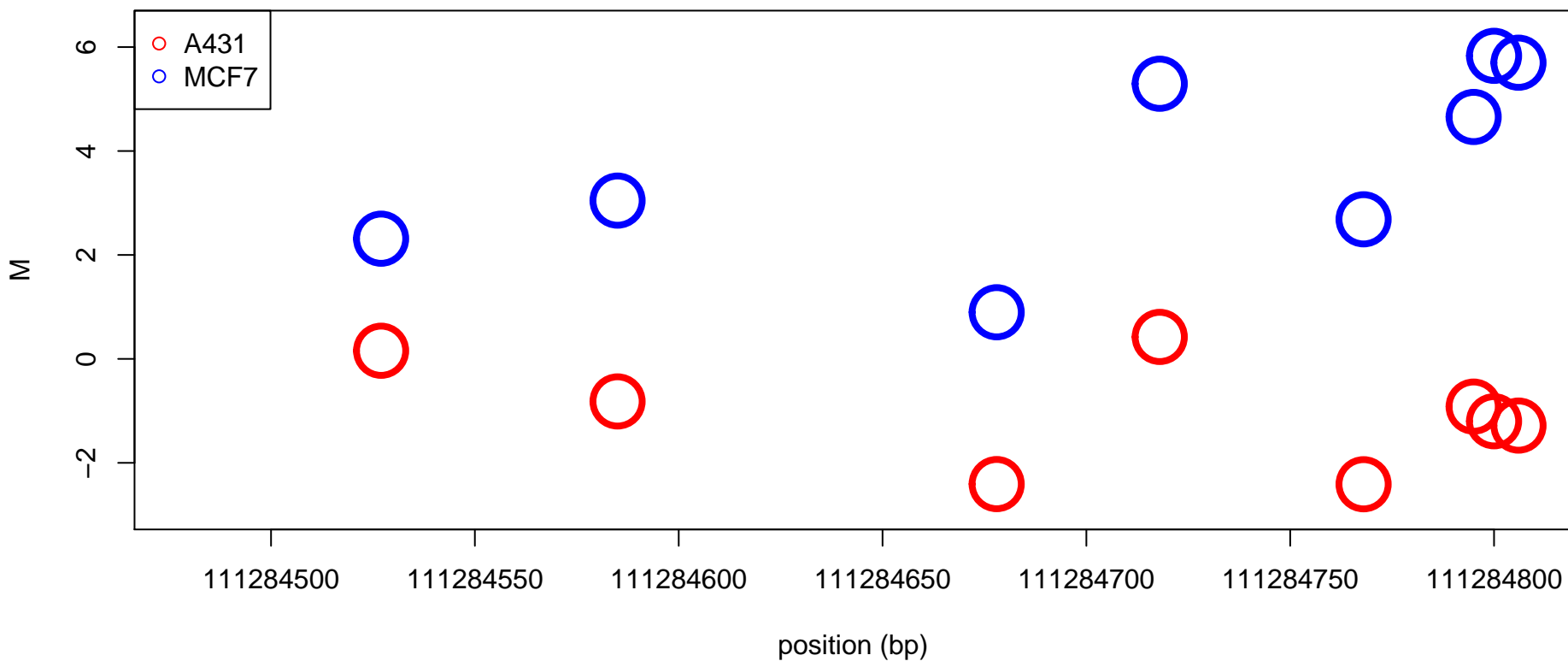

RegionID: 10011, chr12:111284527-111284806-Beta\_values

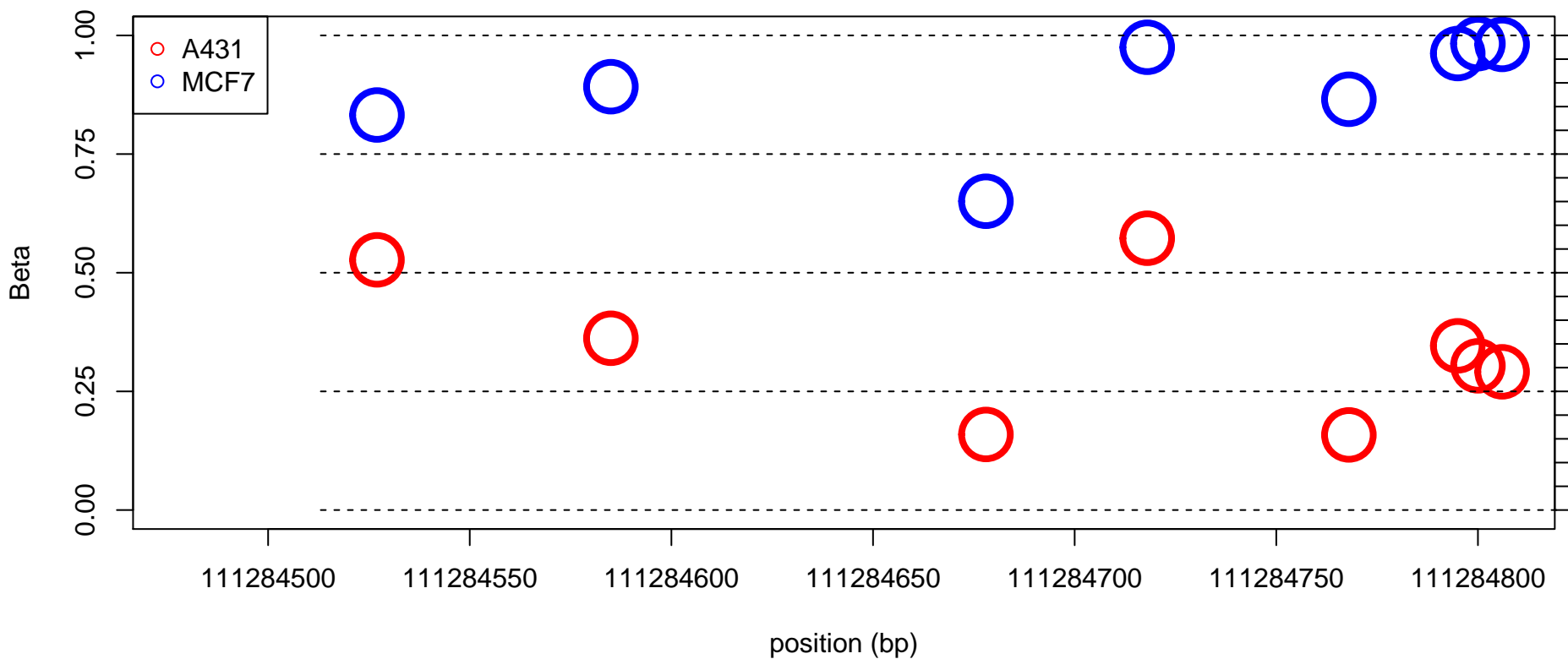

Chromosome 12

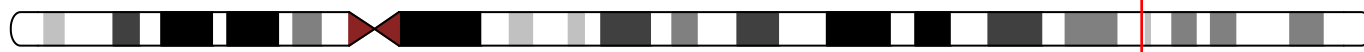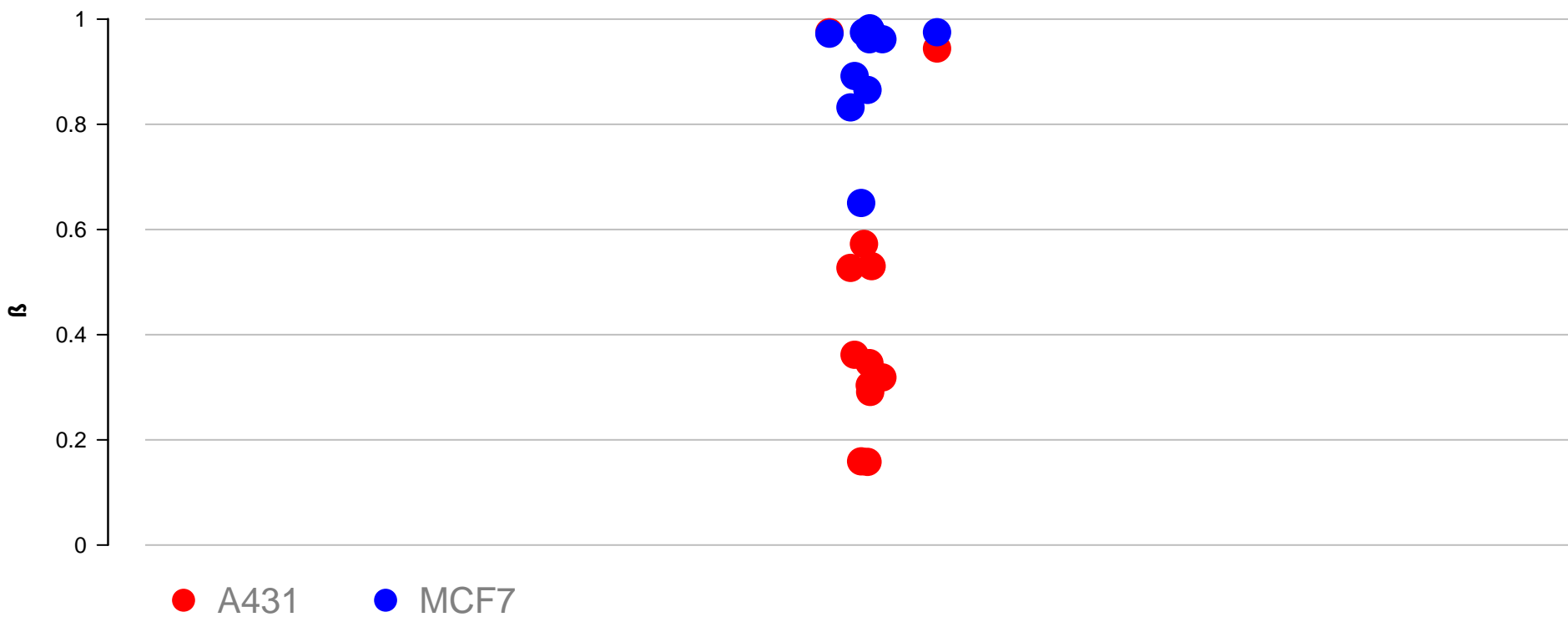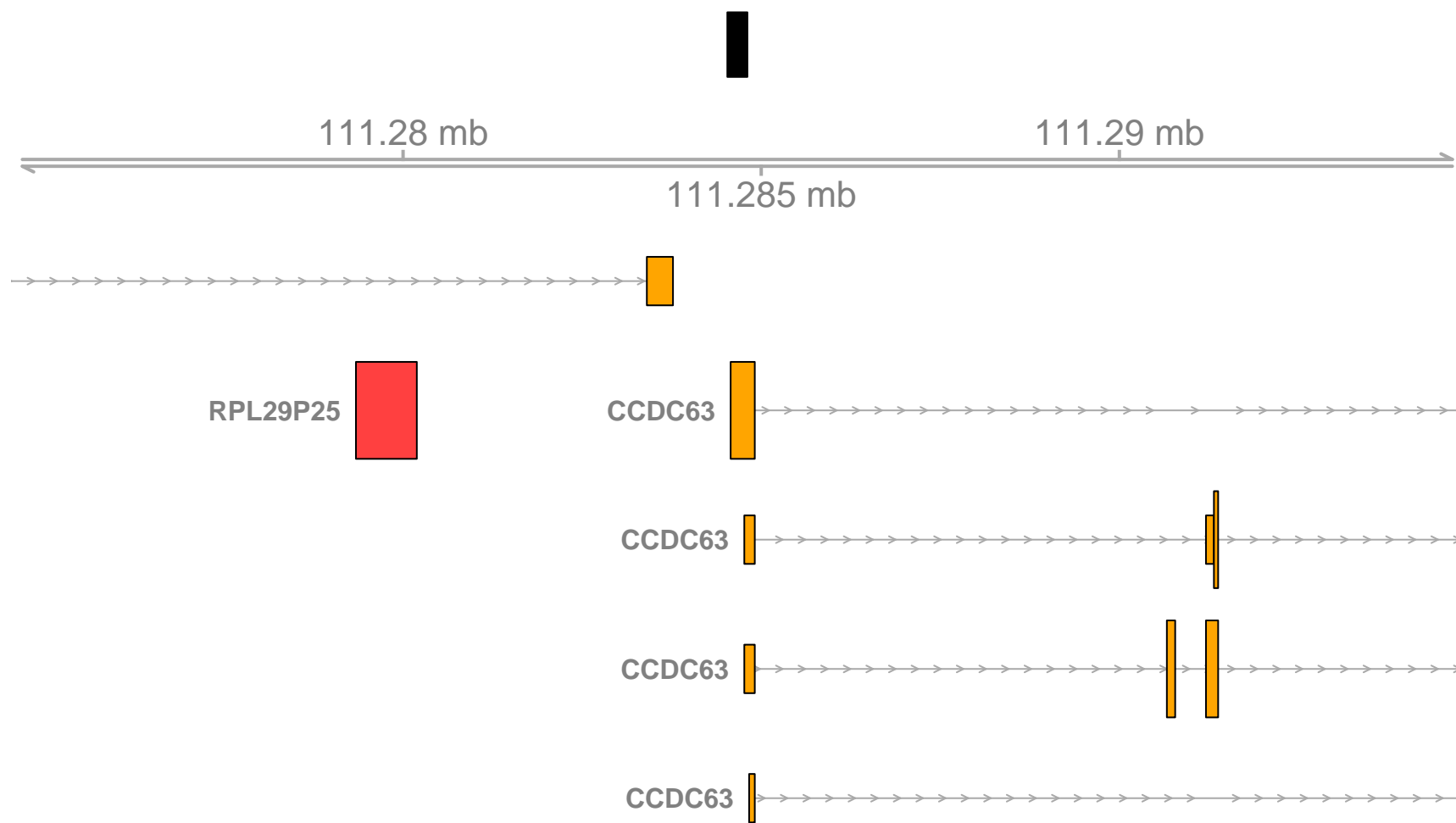

Supplement: Additional file 2 — DMRforPairs output for the comparison of A431-MCF7 and NA17018-NA17105. Please start from the HTML files in each folder. Available via the BMC Bioinformatics website. [file 1471-2105-15-141-S2.zip › 1394847754114233_MOESM2_ESM/A431_MCF7/figures/10011.pdf]

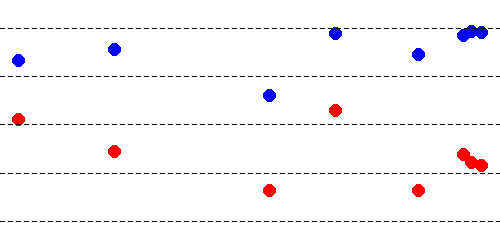

Supplement: Additional file 2 — DMRforPairs output for the comparison of A431-MCF7 and NA17018-NA17105. Please start from the HTML files in each folder. Available via the BMC Bioinformatics website. [file 1471-2105-15-141-S2.zip › 1394847754114233_MOESM2_ESM/A431_MCF7/figures/10011.png]

RegionID: 10013, chr12:111471192–111471489–M\_values

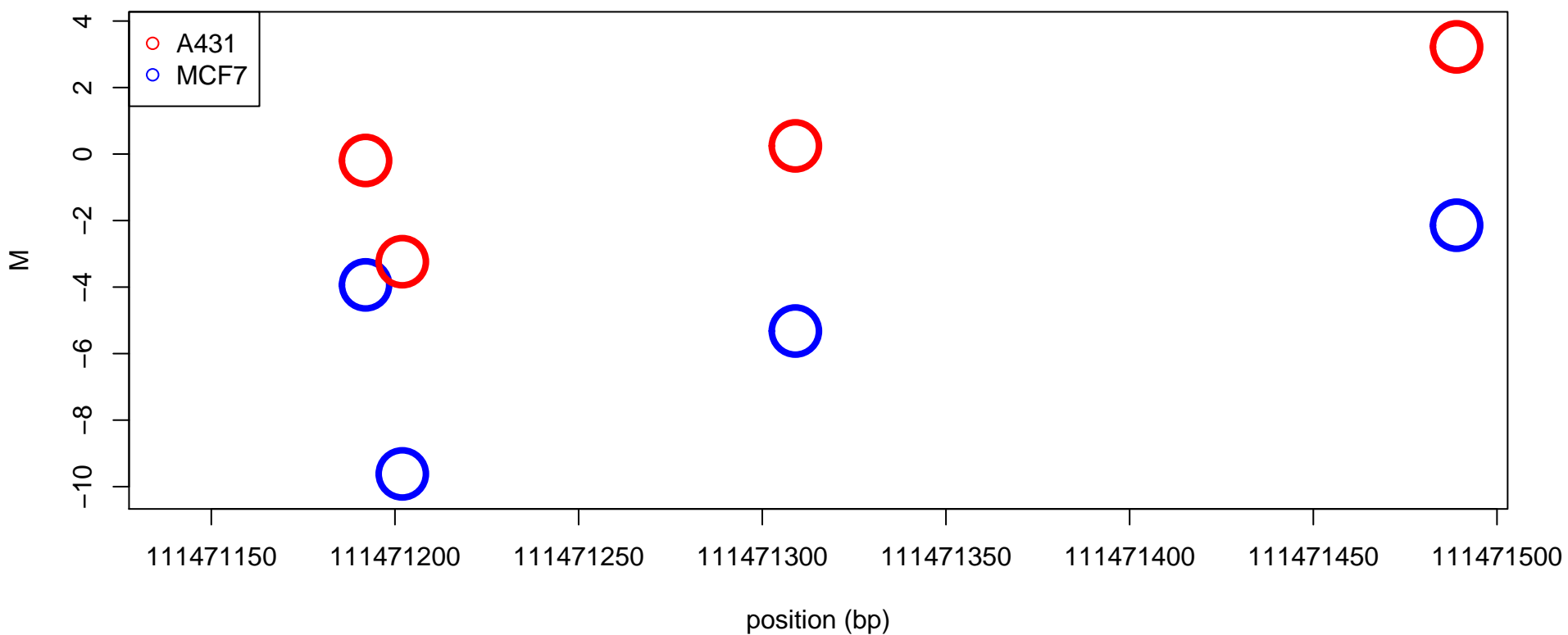

RegionID: 10013, chr12:111471192–111471489–Beta\_values

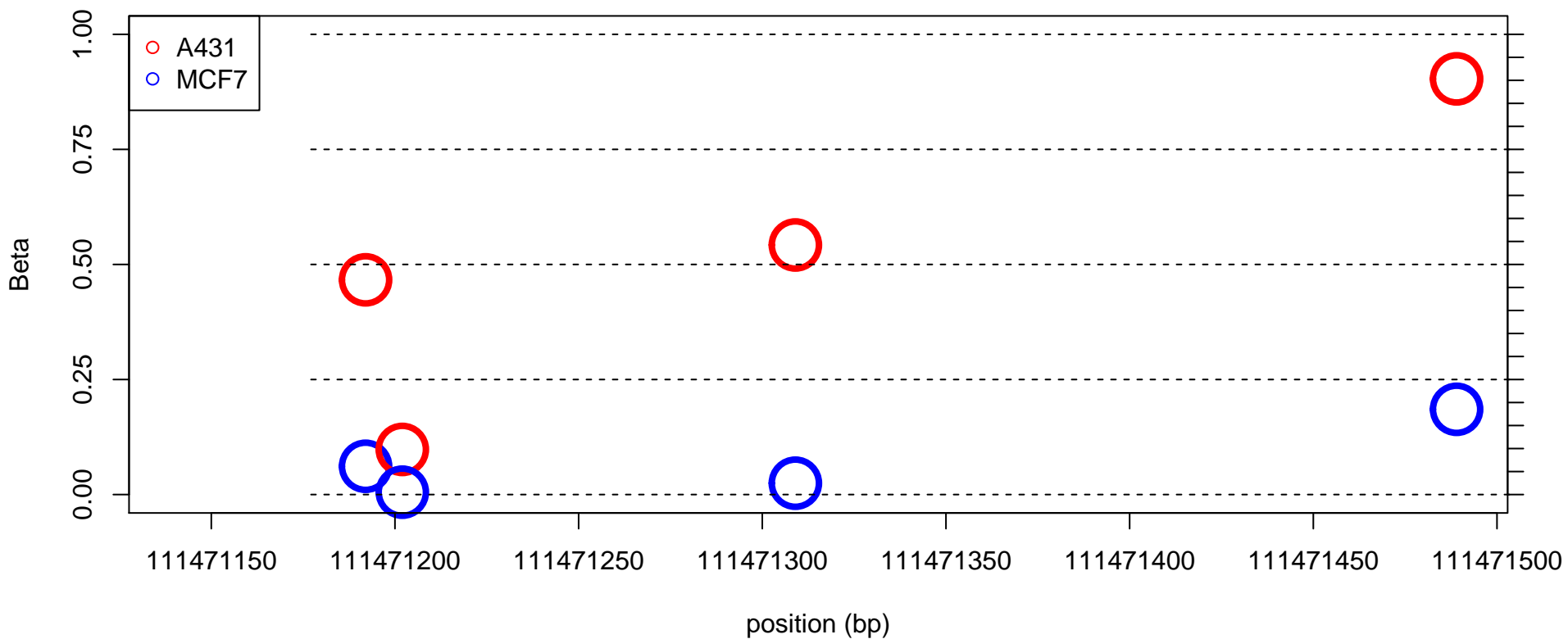

Supplement: Additional file 2 — DMRforPairs output for the comparison of A431-MCF7 and NA17018-NA17105. Please start from the HTML files in each folder. Available via the BMC Bioinformatics website. [file 1471-2105-15-141-S2.zip › 1394847754114233_MOESM2_ESM/A431_MCF7/figures/10013.pdf]

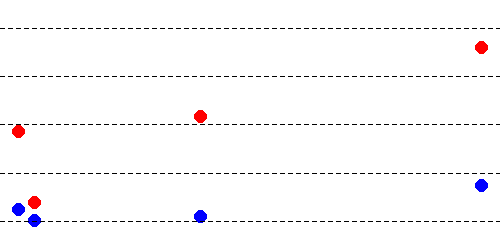

Supplement: Additional file 2 — DMRforPairs output for the comparison of A431-MCF7 and NA17018-NA17105. Please start from the HTML files in each folder. Available via the BMC Bioinformatics website. [file 1471-2105-15-141-S2.zip › 1394847754114233_MOESM2_ESM/A431_MCF7/figures/10013.png]

RegionID: 1002, chr1:213124368–213124685–M\_values

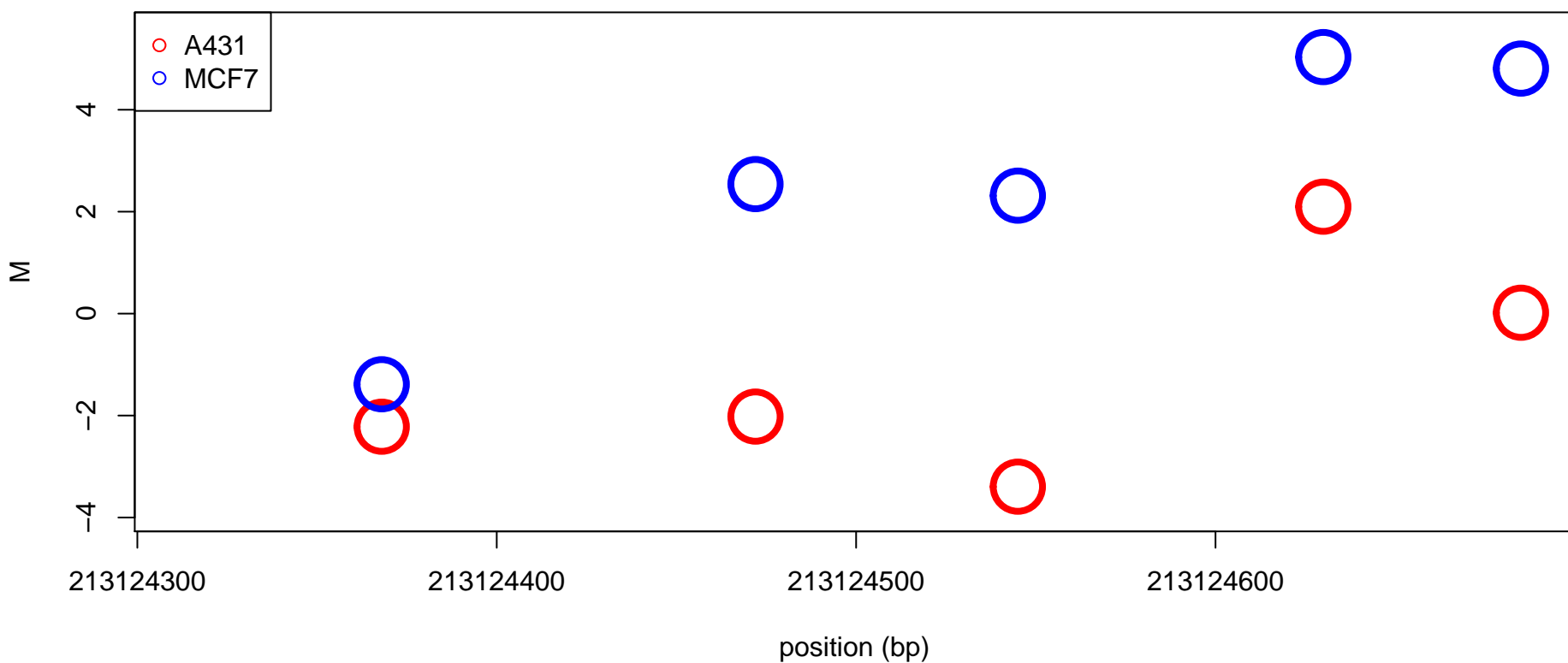

RegionID: 1002, chr1:213124368–213124685–Beta\_values

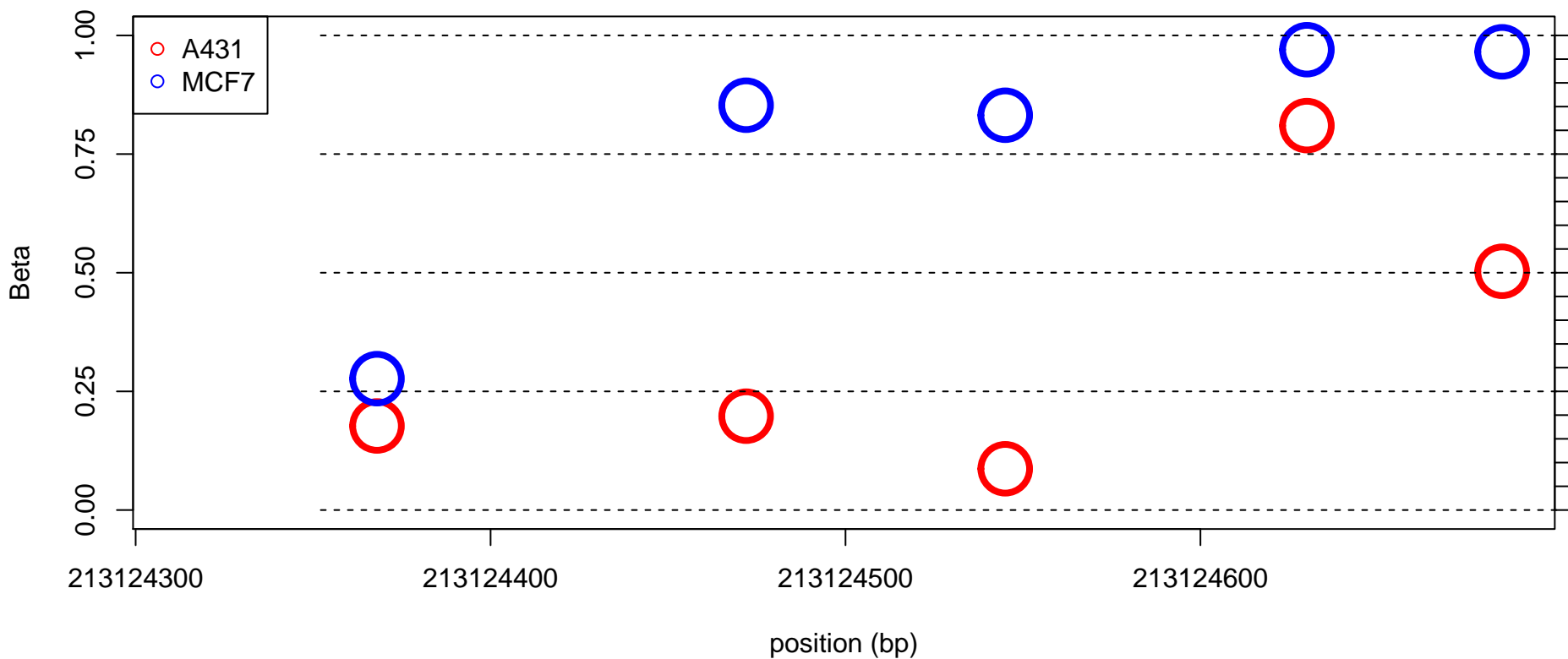

Supplement: Additional file 2 — DMRforPairs output for the comparison of A431-MCF7 and NA17018-NA17105. Please start from the HTML files in each folder. Available via the BMC Bioinformatics website. [file 1471-2105-15-141-S2.zip › 1394847754114233_MOESM2_ESM/A431_MCF7/figures/1002.pdf]

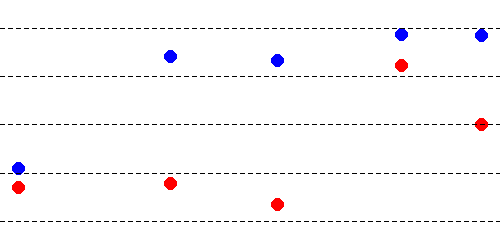

Supplement: Additional file 2 — DMRforPairs output for the comparison of A431-MCF7 and NA17018-NA17105. Please start from the HTML files in each folder. Available via the BMC Bioinformatics website. [file 1471-2105-15-141-S2.zip › 1394847754114233_MOESM2_ESM/A431_MCF7/figures/1002.png]

RegionID: 10023, chr12:113229245–113229534–M\_values

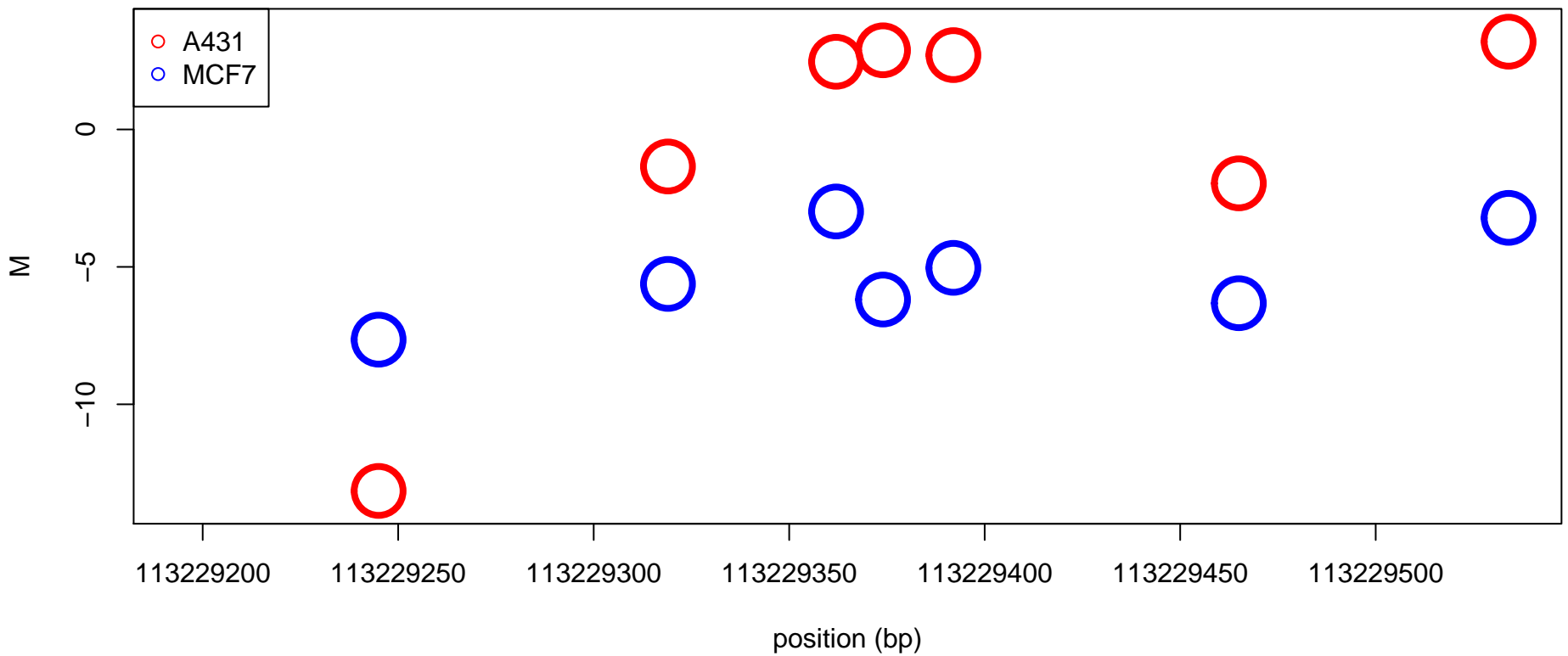

RegionID: 10023, chr12:113229245–113229534–Beta\_values

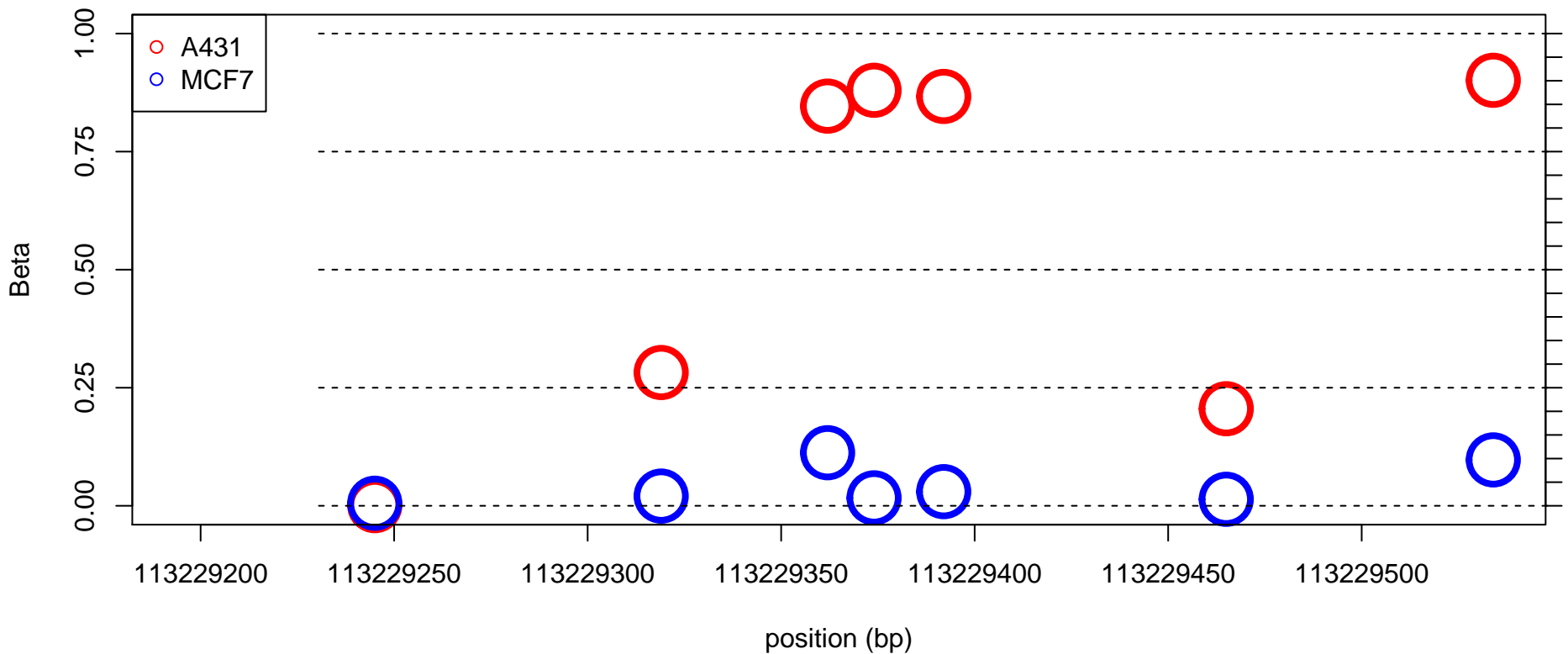

Supplement: Additional file 2 — DMRforPairs output for the comparison of A431-MCF7 and NA17018-NA17105. Please start from the HTML files in each folder. Available via the BMC Bioinformatics website. [file 1471-2105-15-141-S2.zip › 1394847754114233_MOESM2_ESM/A431_MCF7/figures/10023.pdf]

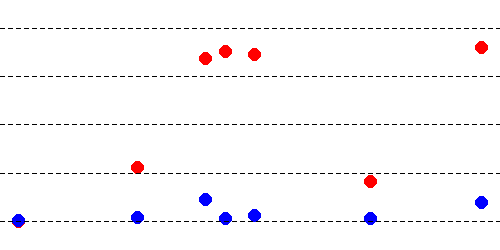

Supplement: Additional file 2 — DMRforPairs output for the comparison of A431-MCF7 and NA17018-NA17105. Please start from the HTML files in each folder. Available via the BMC Bioinformatics website. [file 1471-2105-15-141-S2.zip › 1394847754114233_MOESM2_ESM/A431_MCF7/figures/10023.png]

RegionID: 10024, chr12:113415883–113416268–M\_values

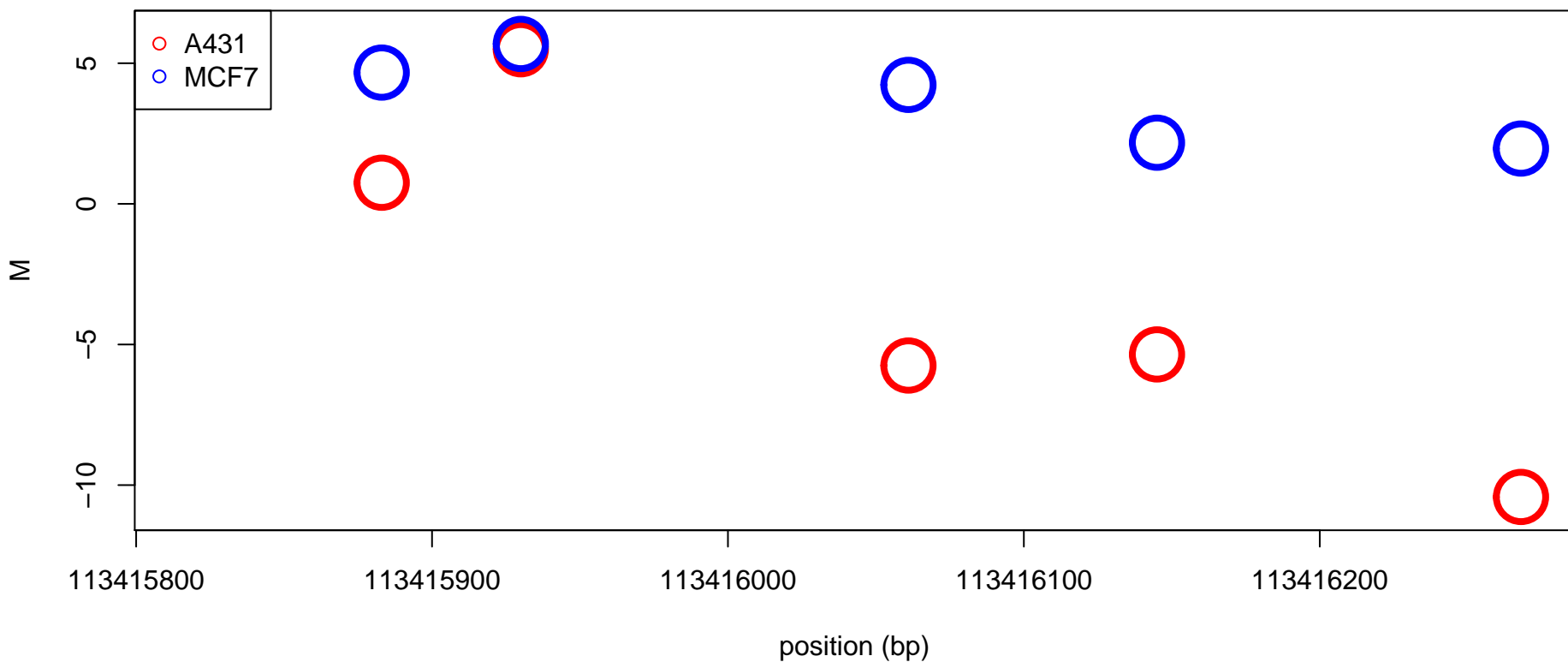

RegionID: 10024, chr12:113415883–113416268–Beta\_values

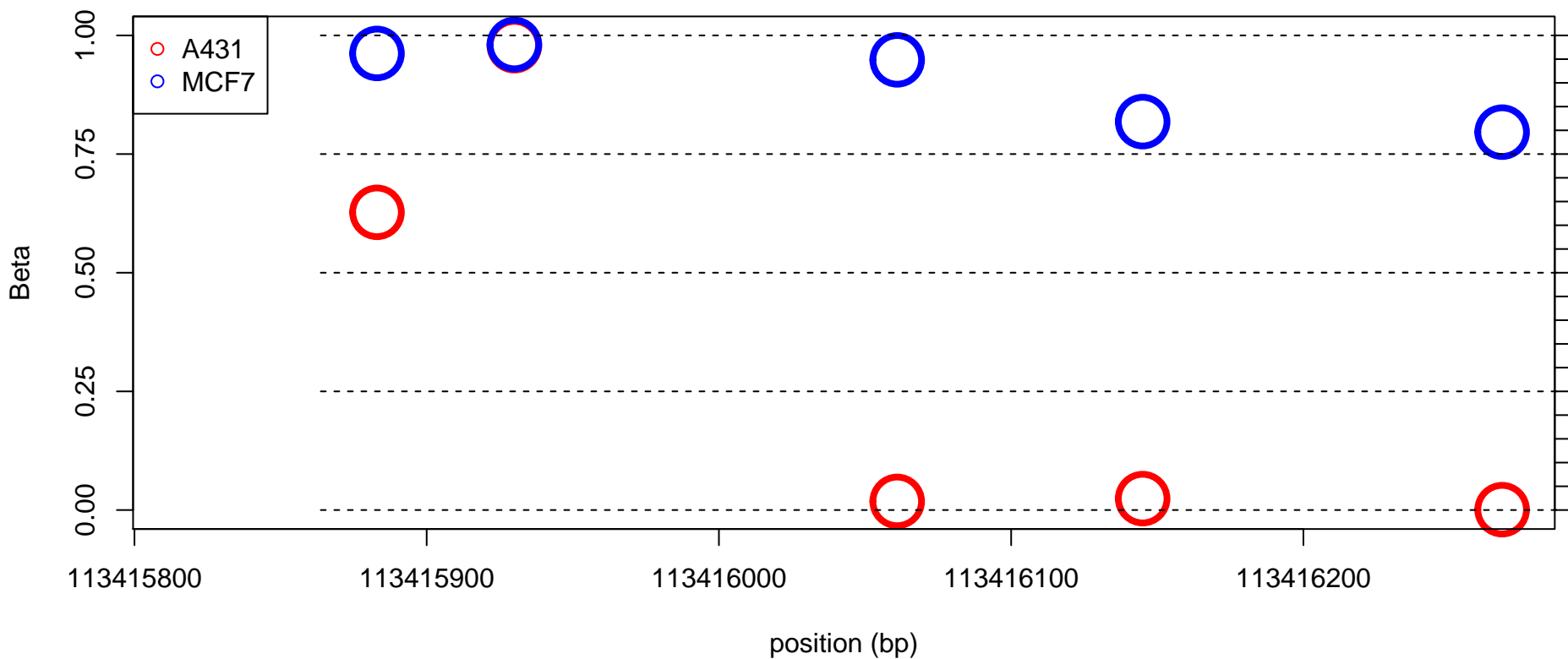

Supplement: Additional file 2 — DMRforPairs output for the comparison of A431-MCF7 and NA17018-NA17105. Please start from the HTML files in each folder. Available via the BMC Bioinformatics website. [file 1471-2105-15-141-S2.zip › 1394847754114233_MOESM2_ESM/A431_MCF7/figures/10024.pdf]

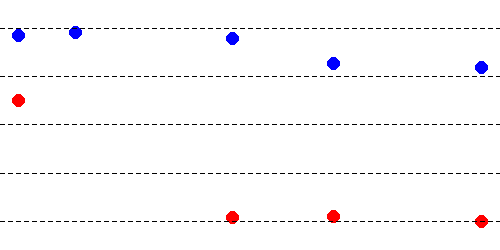

Supplement: Additional file 2 — DMRforPairs output for the comparison of A431-MCF7 and NA17018-NA17105. Please start from the HTML files in each folder. Available via the BMC Bioinformatics website. [file 1471-2105-15-141-S2.zip › 1394847754114233_MOESM2_ESM/A431_MCF7/figures/10024.png]

RegionID: 10026, chr12:113587240–113587581–M\_values

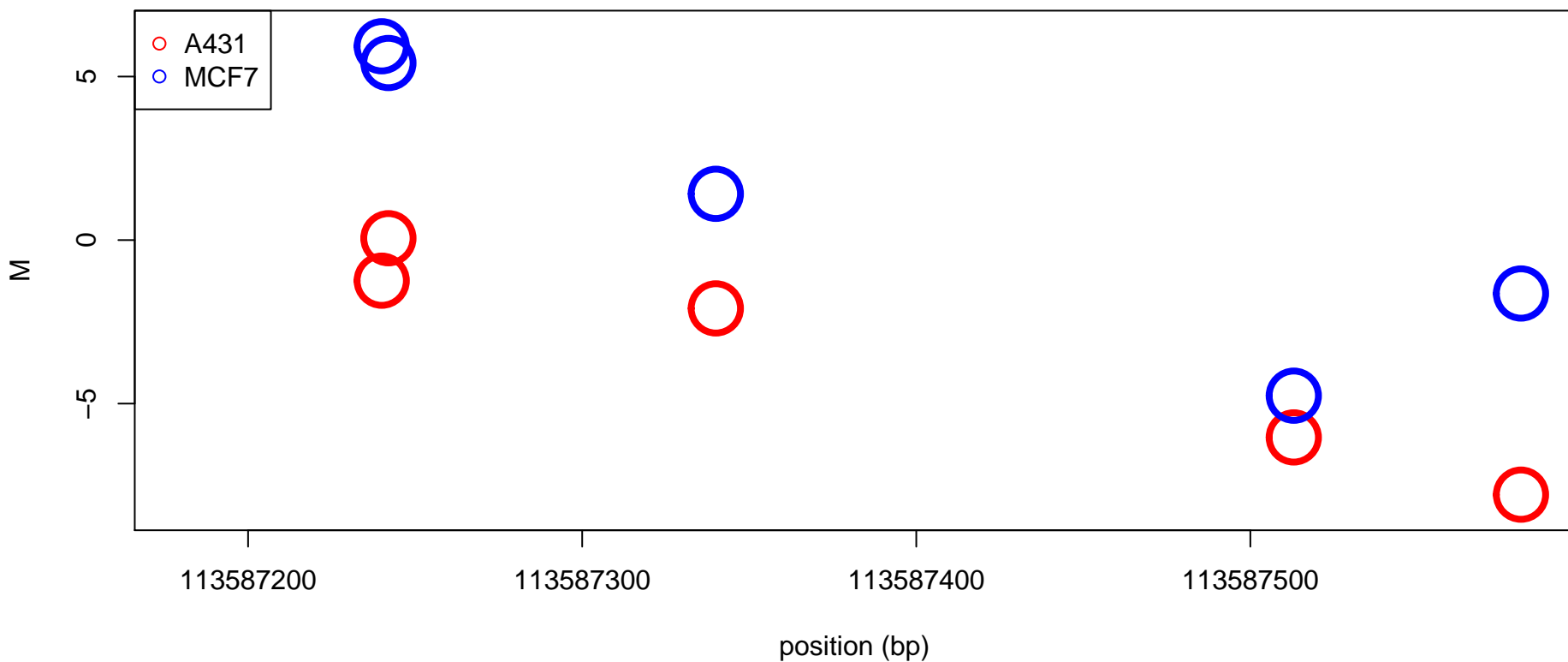

RegionID: 10026, chr12:113587240–113587581–Beta\_values

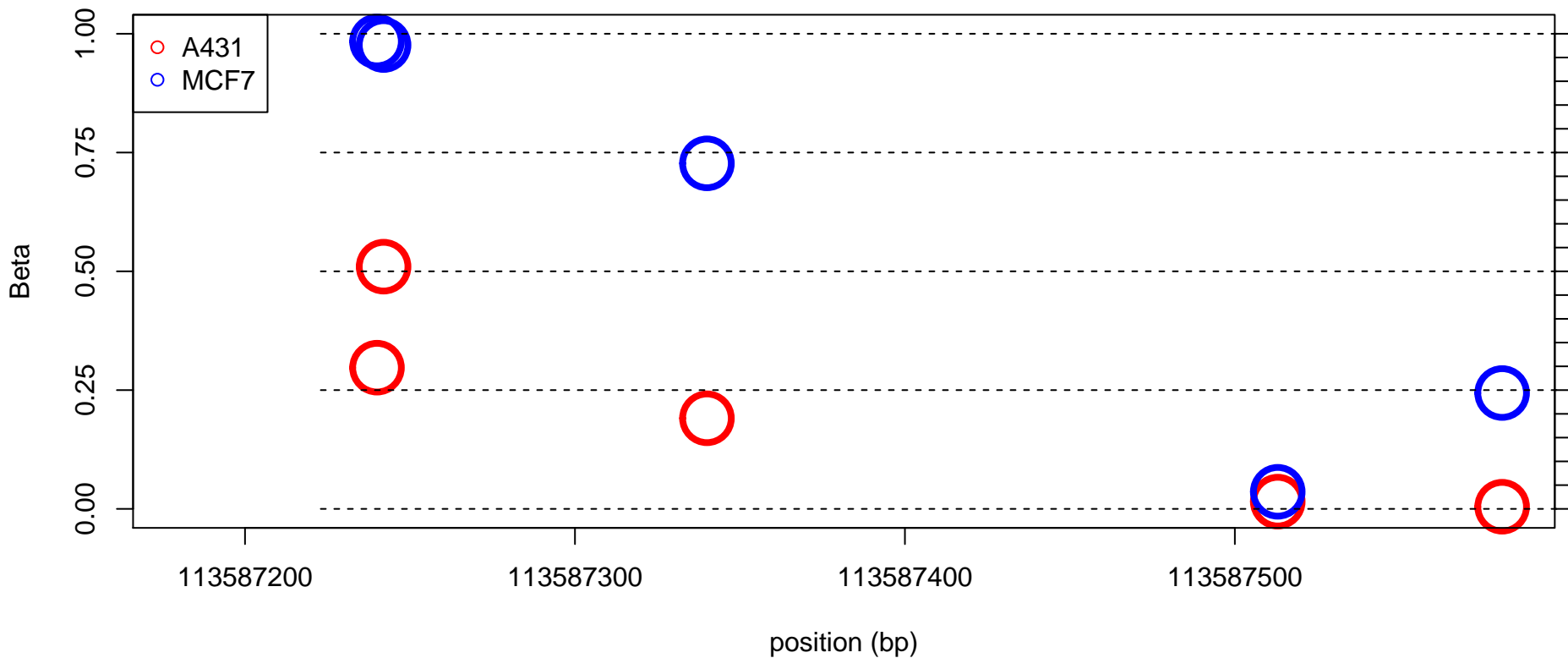

Supplement: Additional file 2 — DMRforPairs output for the comparison of A431-MCF7 and NA17018-NA17105. Please start from the HTML files in each folder. Available via the BMC Bioinformatics website. [file 1471-2105-15-141-S2.zip › 1394847754114233_MOESM2_ESM/A431_MCF7/figures/10026.pdf]

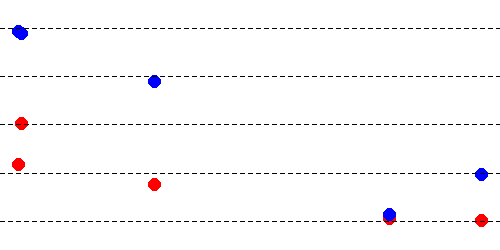

Supplement: Additional file 2 — DMRforPairs output for the comparison of A431-MCF7 and NA17018-NA17105. Please start from the HTML files in each folder. Available via the BMC Bioinformatics website. [file 1471-2105-15-141-S2.zip › 1394847754114233_MOESM2_ESM/A431_MCF7/figures/10026.png]

RegionID: 10030, chr12:113909932-113910034-M\_values

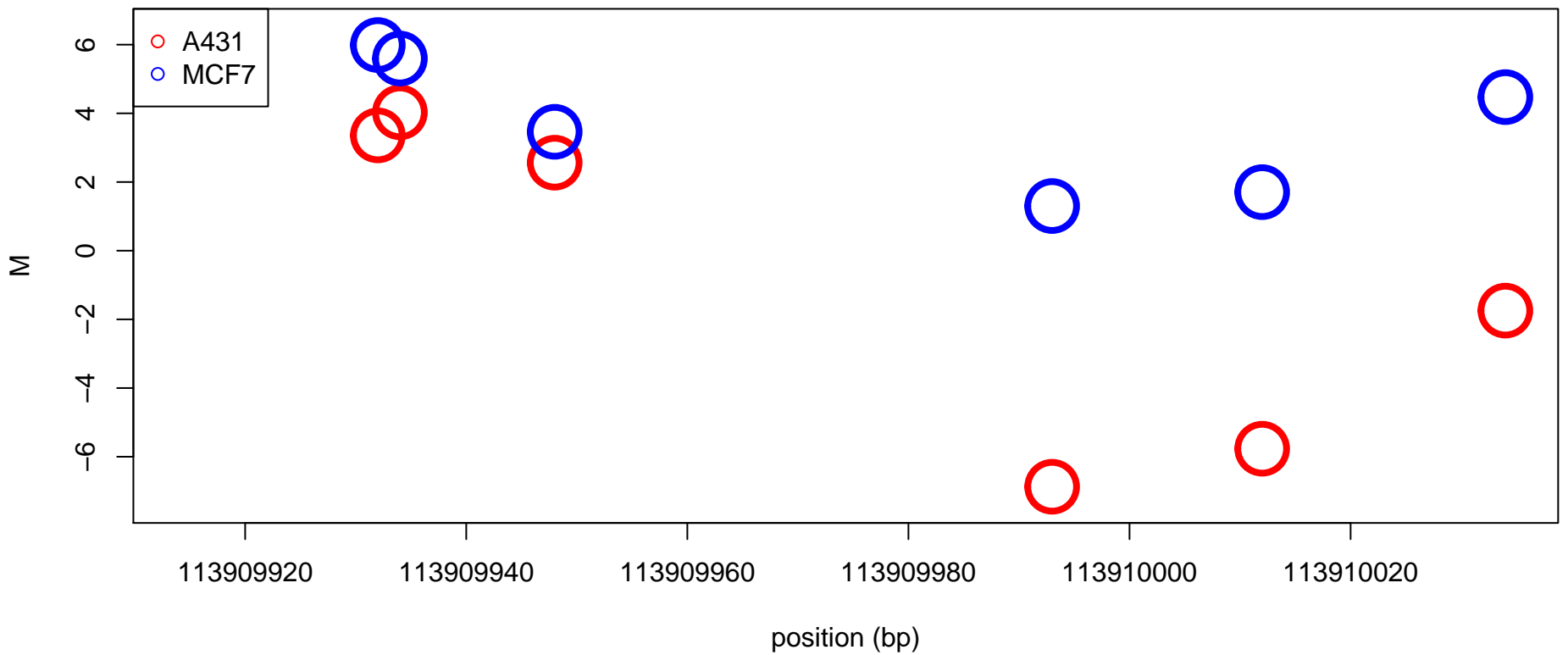

RegionID: 10030, chr12:113909932-113910034-Beta\_values

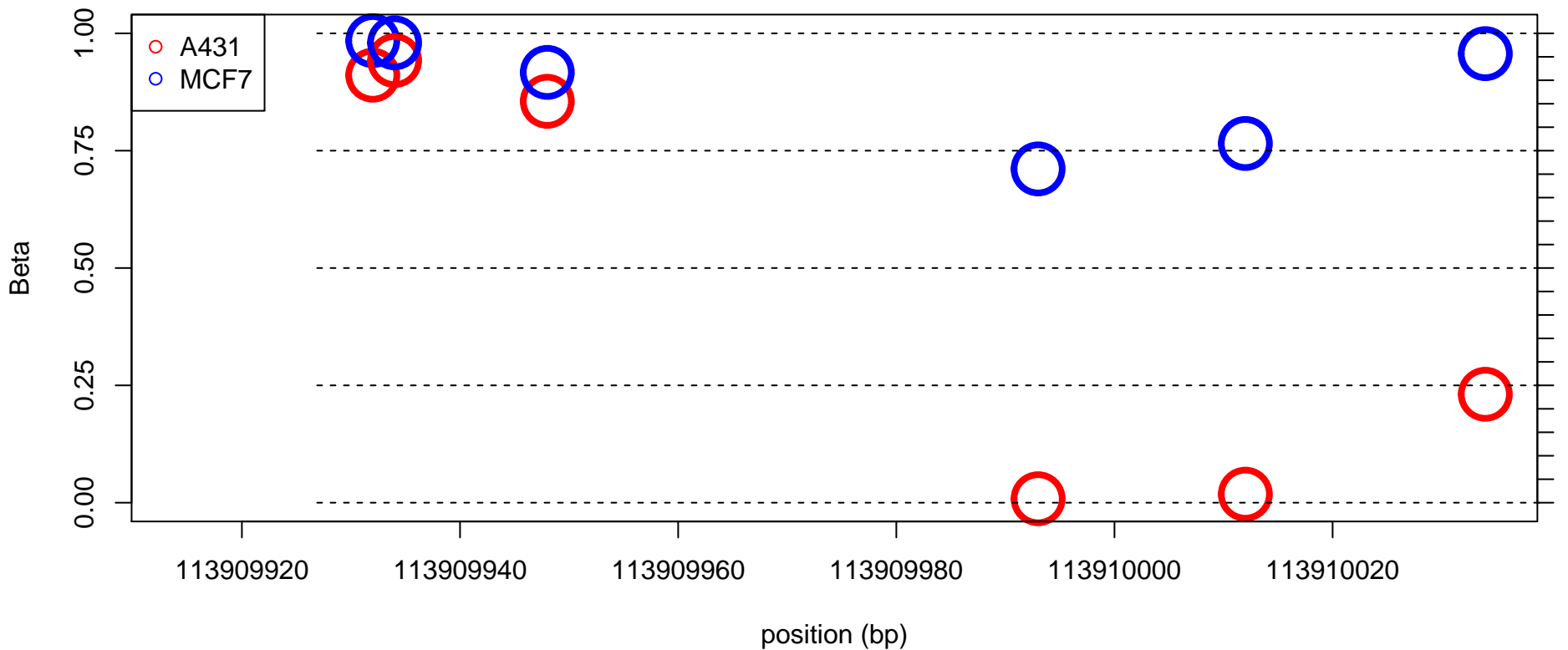

Supplement: Additional file 2 — DMRforPairs output for the comparison of A431-MCF7 and NA17018-NA17105. Please start from the HTML files in each folder. Available via the BMC Bioinformatics website. [file 1471-2105-15-141-S2.zip › 1394847754114233_MOESM2_ESM/A431_MCF7/figures/10030.pdf]

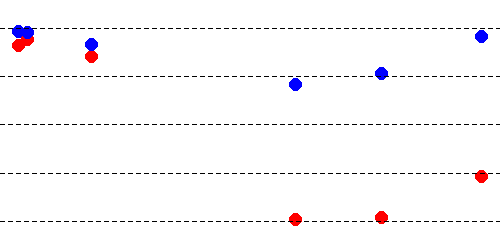

Supplement: Additional file 2 — DMRforPairs output for the comparison of A431-MCF7 and NA17018-NA17105. Please start from the HTML files in each folder. Available via the BMC Bioinformatics website. [file 1471-2105-15-141-S2.zip › 1394847754114233_MOESM2_ESM/A431_MCF7/figures/10030.png]

RegionID: 10032, chr12:114841708–114842031–M\_values

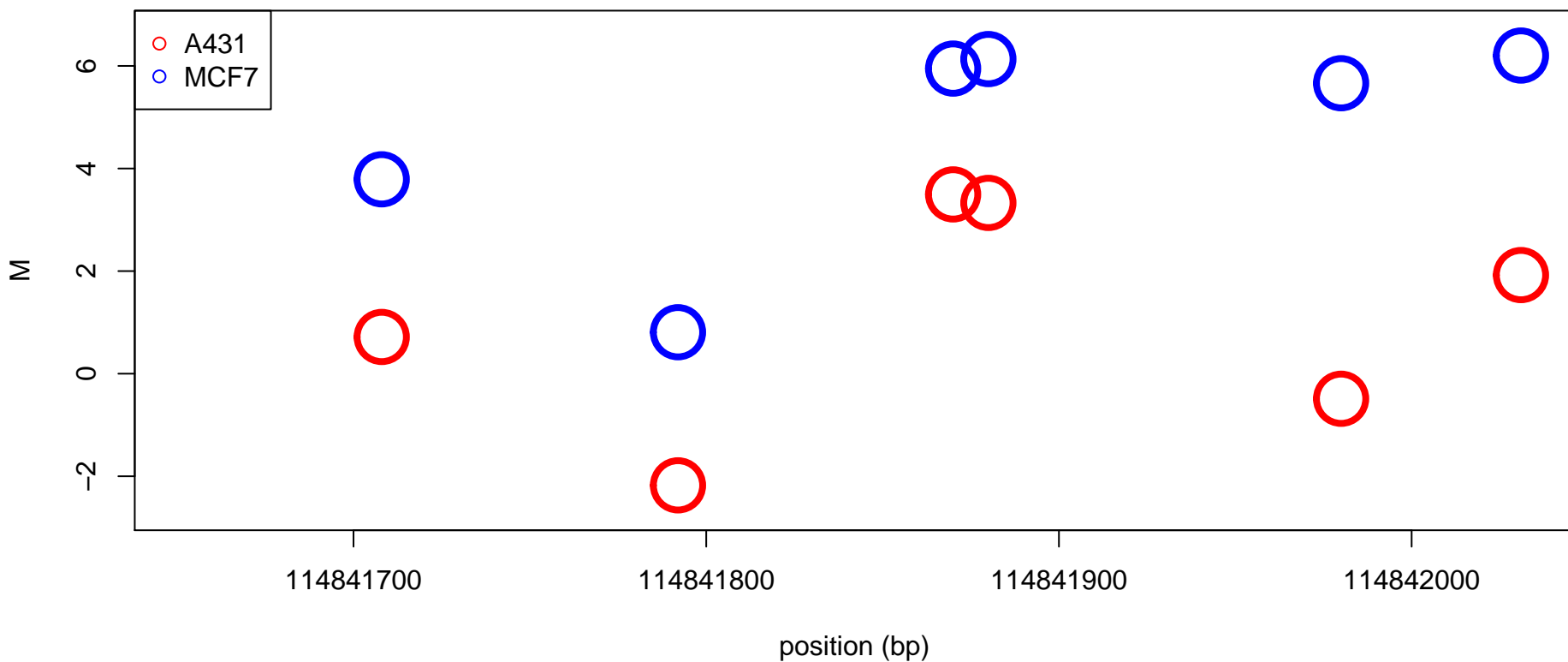

RegionID: 10032, chr12:114841708–114842031–Beta\_values

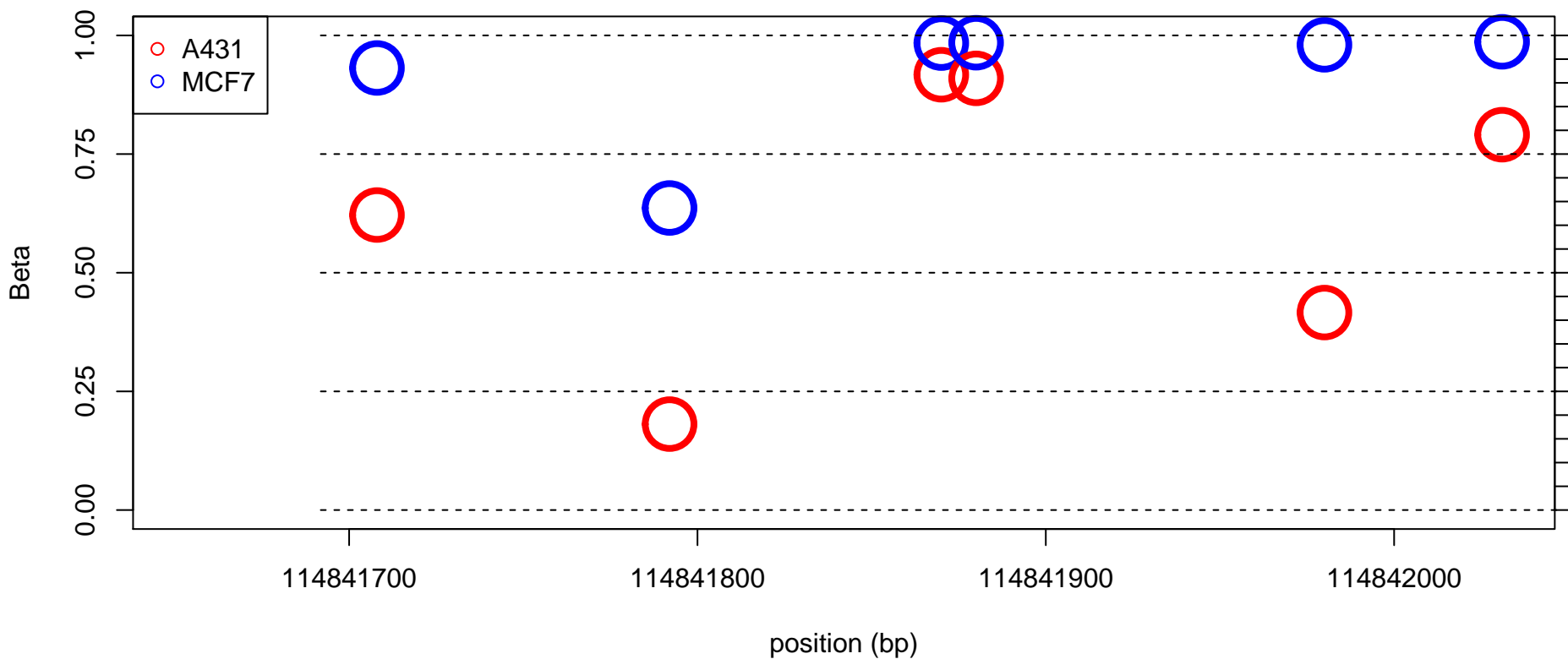

Supplement: Additional file 2 — DMRforPairs output for the comparison of A431-MCF7 and NA17018-NA17105. Please start from the HTML files in each folder. Available via the BMC Bioinformatics website. [file 1471-2105-15-141-S2.zip › 1394847754114233_MOESM2_ESM/A431_MCF7/figures/10032.pdf]

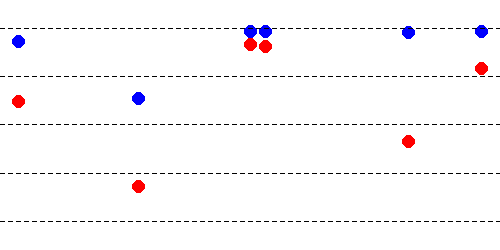

Supplement: Additional file 2 — DMRforPairs output for the comparison of A431-MCF7 and NA17018-NA17105. Please start from the HTML files in each folder. Available via the BMC Bioinformatics website. [file 1471-2105-15-141-S2.zip › 1394847754114233_MOESM2_ESM/A431_MCF7/figures/10032.png]

RegionID: 10033, chr12:114846313–114846503–M\_values

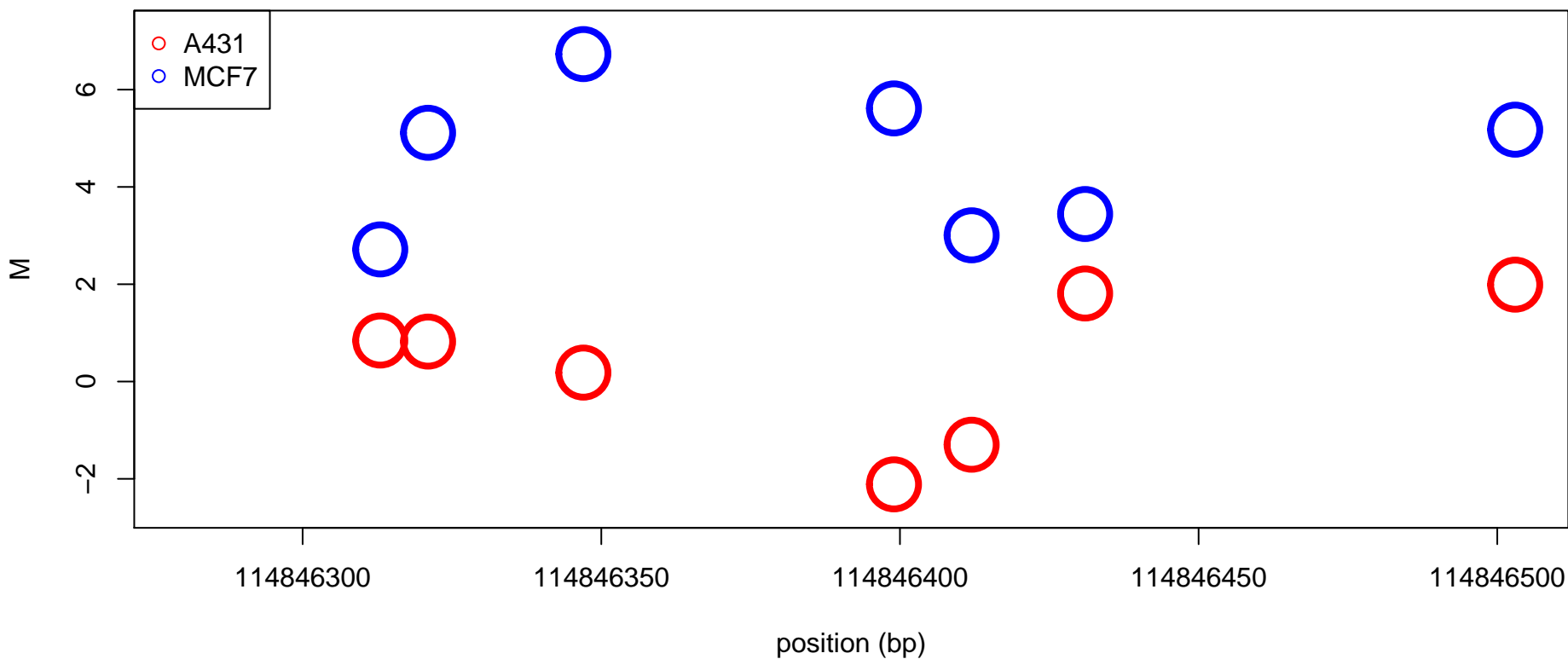

RegionID: 10033, chr12:114846313–114846503–Beta\_values

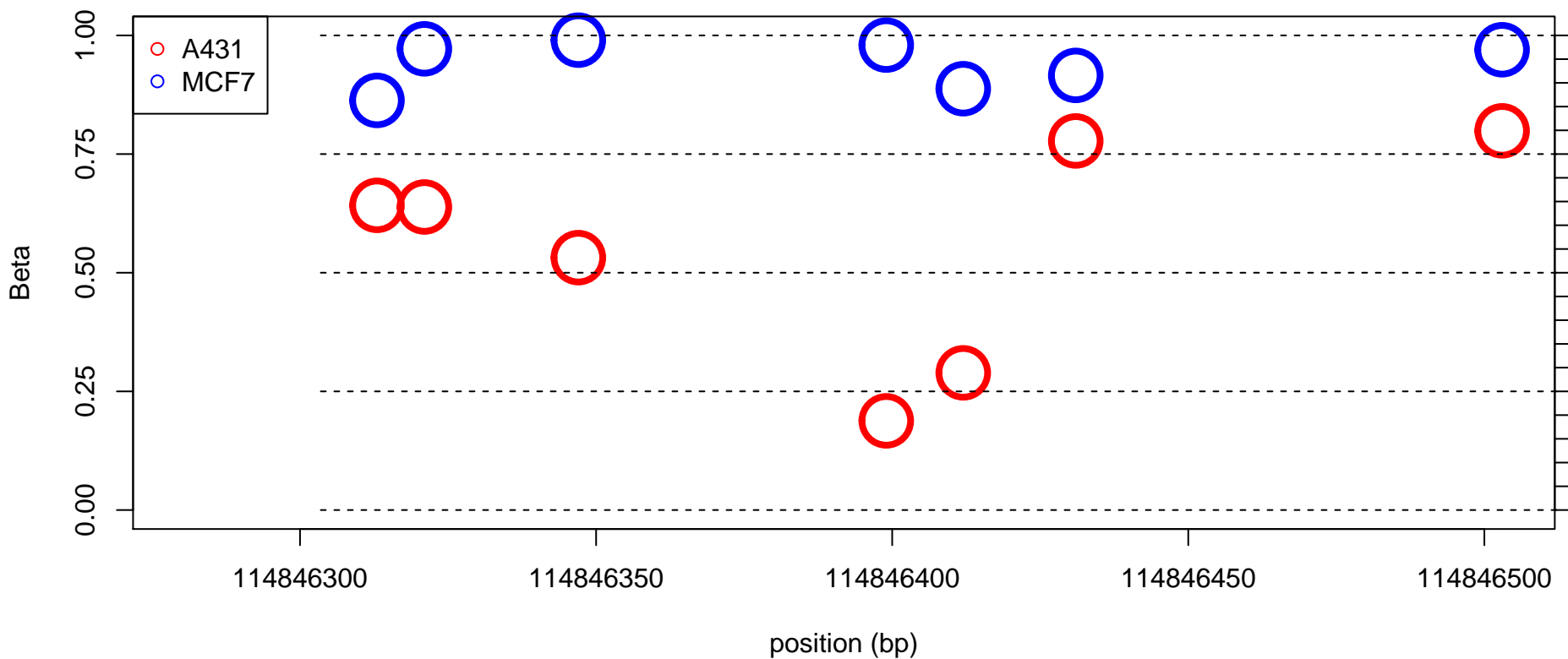

Chromosome 12

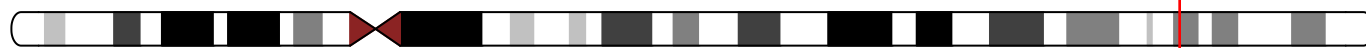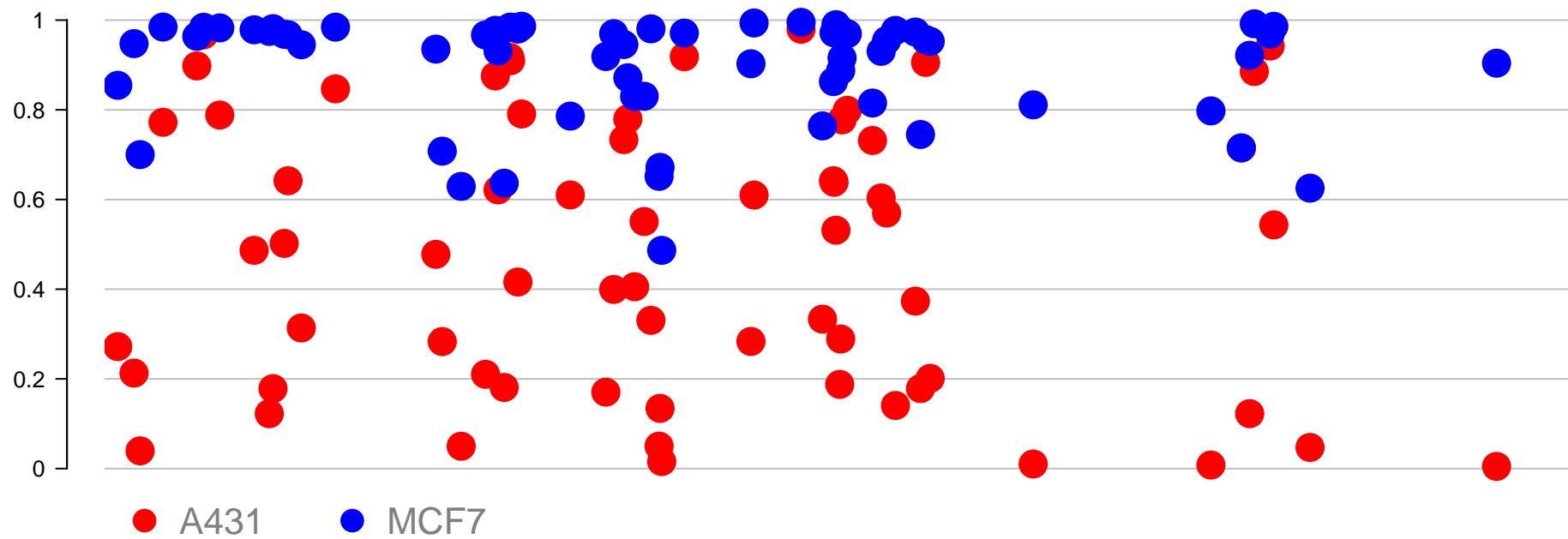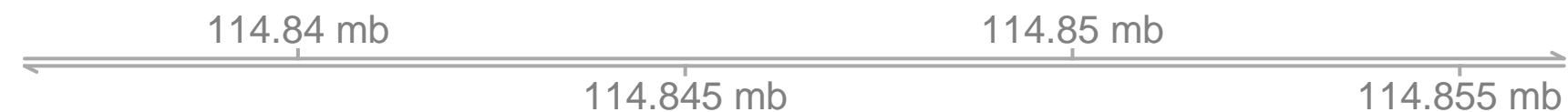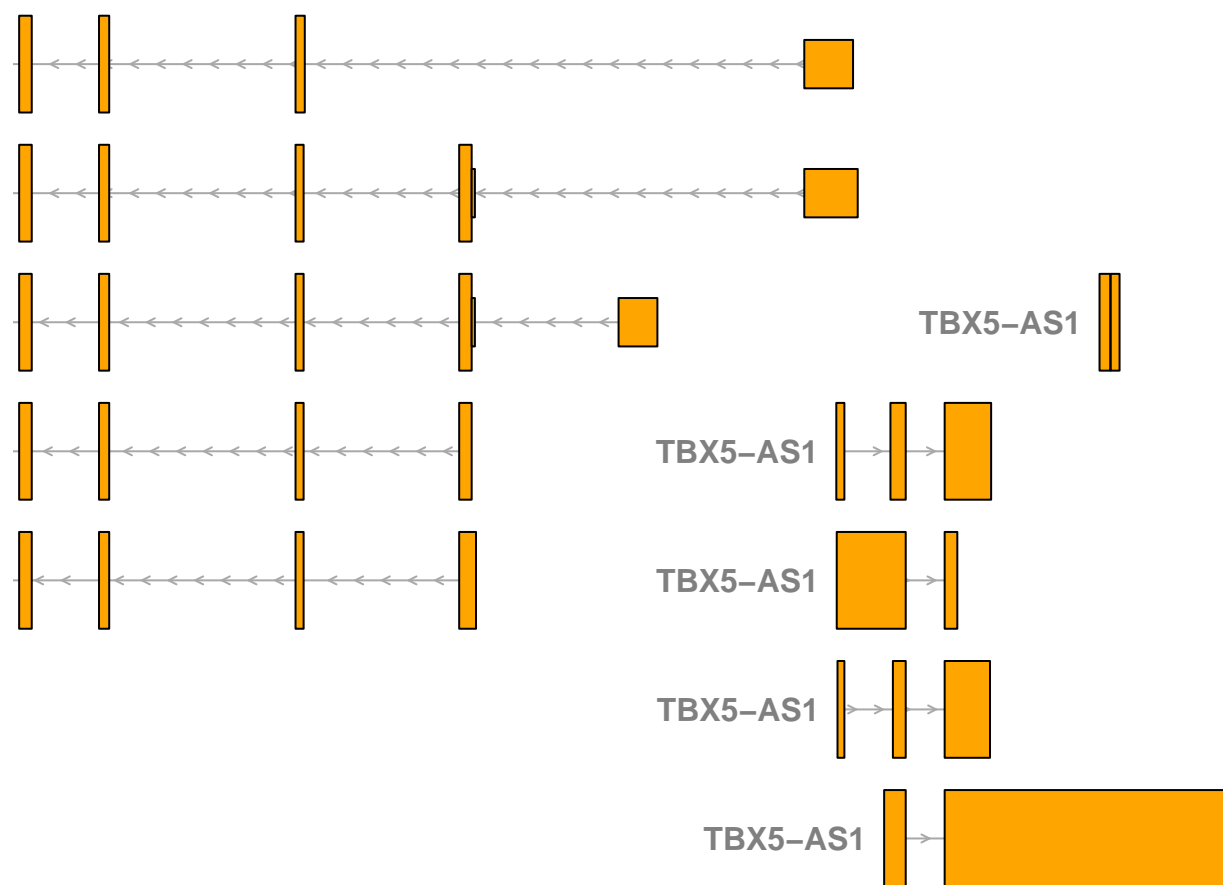

Supplement: Additional file 2 — DMRforPairs output for the comparison of A431-MCF7 and NA17018-NA17105. Please start from the HTML files in each folder. Available via the BMC Bioinformatics website. [file 1471-2105-15-141-S2.zip › 1394847754114233_MOESM2_ESM/A431_MCF7/figures/10033.pdf]

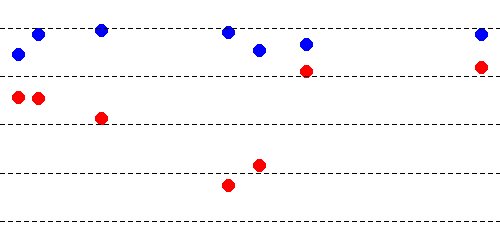

Supplement: Additional file 2 — DMRforPairs output for the comparison of A431-MCF7 and NA17018-NA17105. Please start from the HTML files in each folder. Available via the BMC Bioinformatics website. [file 1471-2105-15-141-S2.zip › 1394847754114233_MOESM2_ESM/A431_MCF7/figures/10033.png]

RegionID: 10034, chr12:114846849–114847164–M\_values

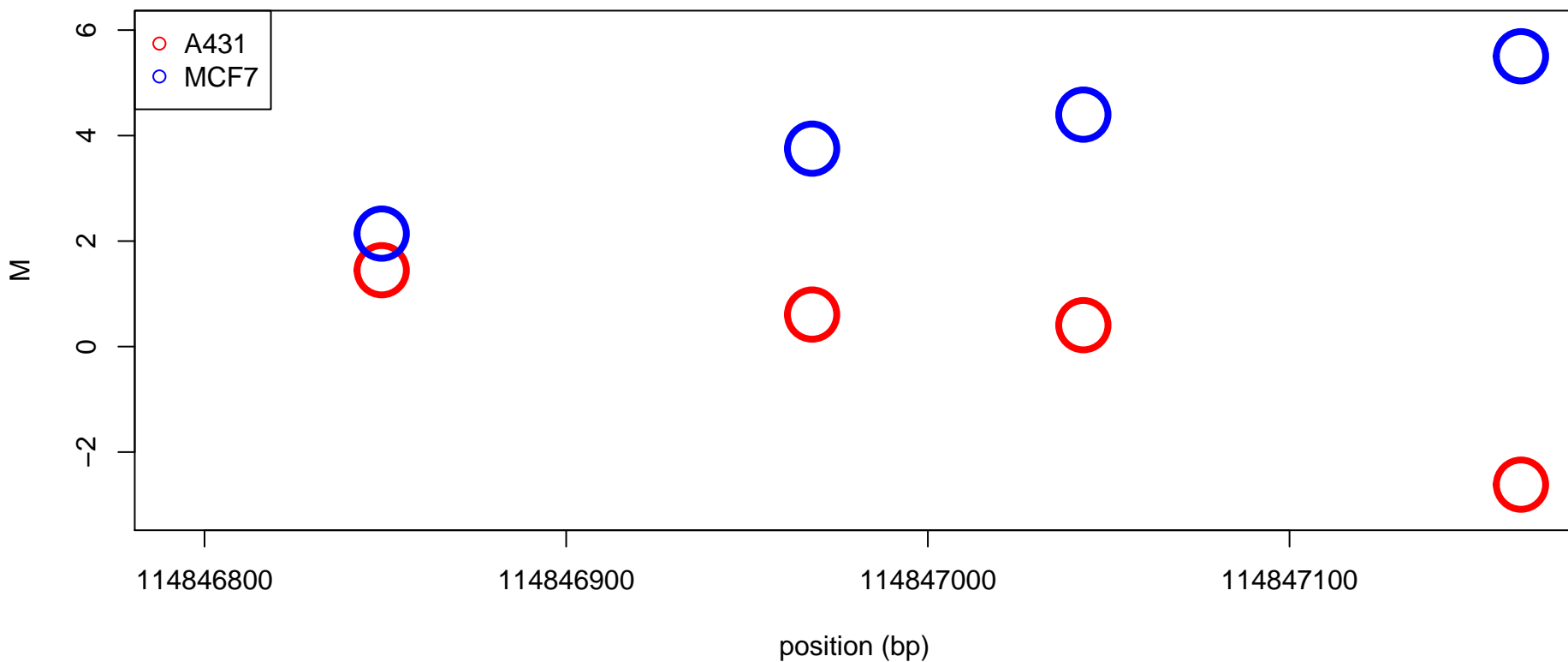

RegionID: 10034, chr12:114846849–114847164–Beta\_values

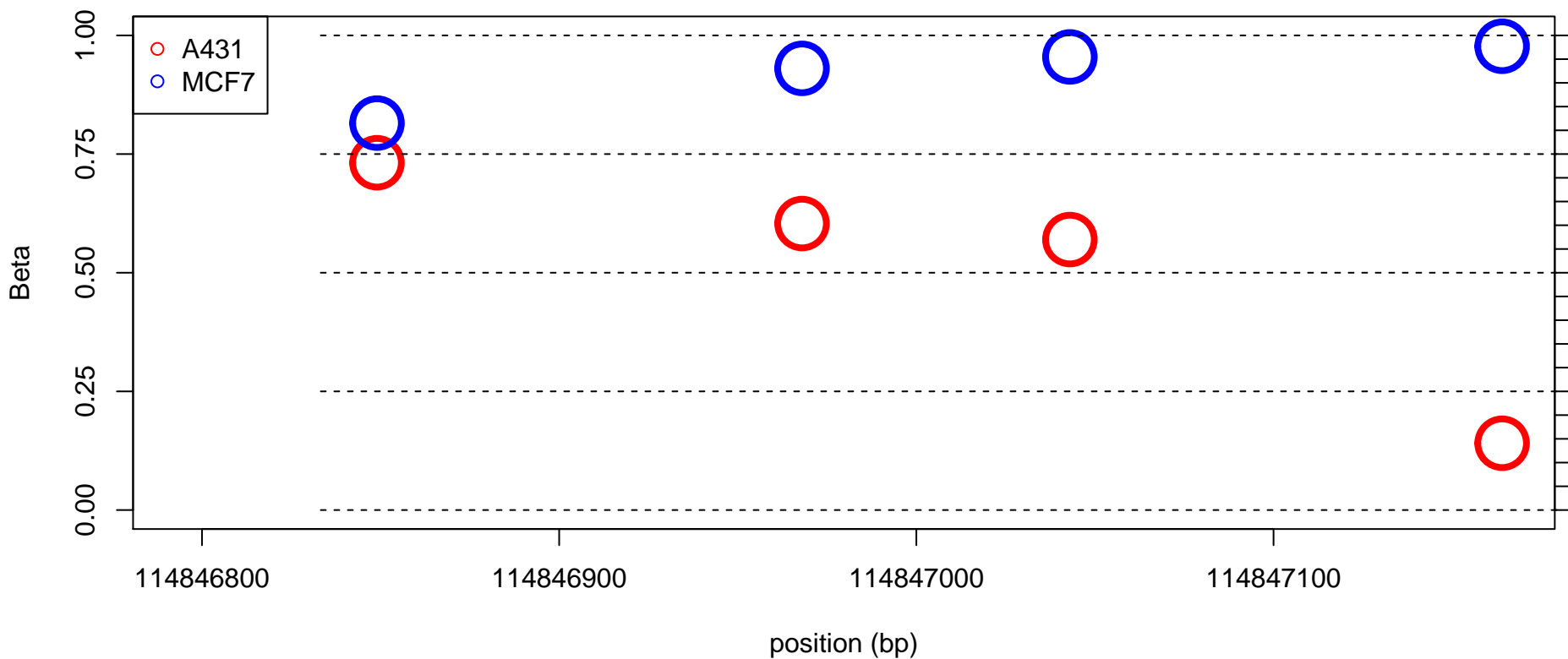

Supplement: Additional file 2 — DMRforPairs output for the comparison of A431-MCF7 and NA17018-NA17105. Please start from the HTML files in each folder. Available via the BMC Bioinformatics website. [file 1471-2105-15-141-S2.zip › 1394847754114233_MOESM2_ESM/A431_MCF7/figures/10034.pdf]

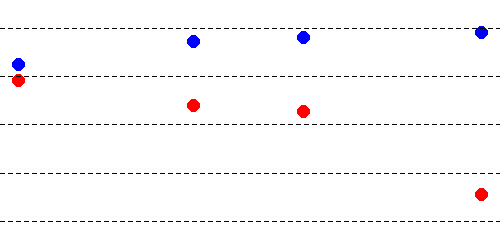

Supplement: Additional file 2 — DMRforPairs output for the comparison of A431-MCF7 and NA17018-NA17105. Please start from the HTML files in each folder. Available via the BMC Bioinformatics website. [file 1471-2105-15-141-S2.zip › 1394847754114233_MOESM2_ESM/A431_MCF7/figures/10034.png]

RegionID: 10035, chr12:114847438–114847641–M\_values

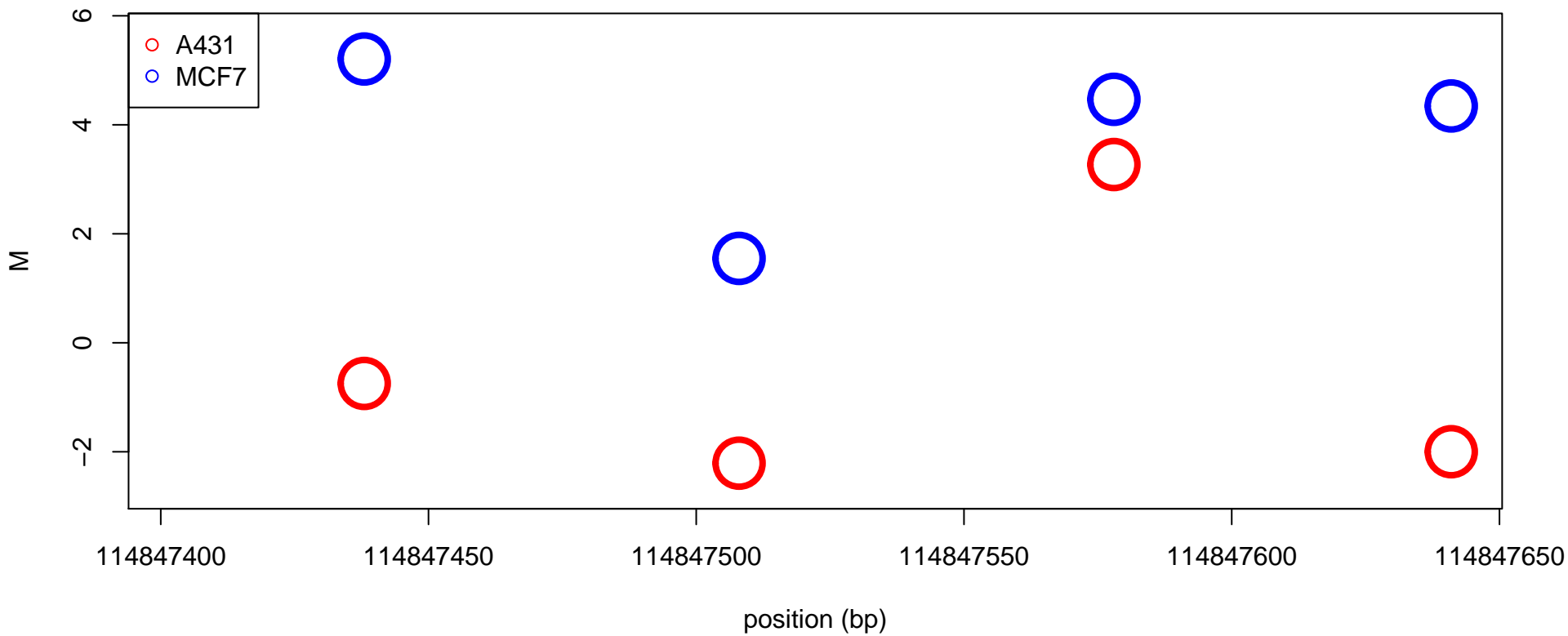

RegionID: 10035, chr12:114847438–114847641–Beta\_values

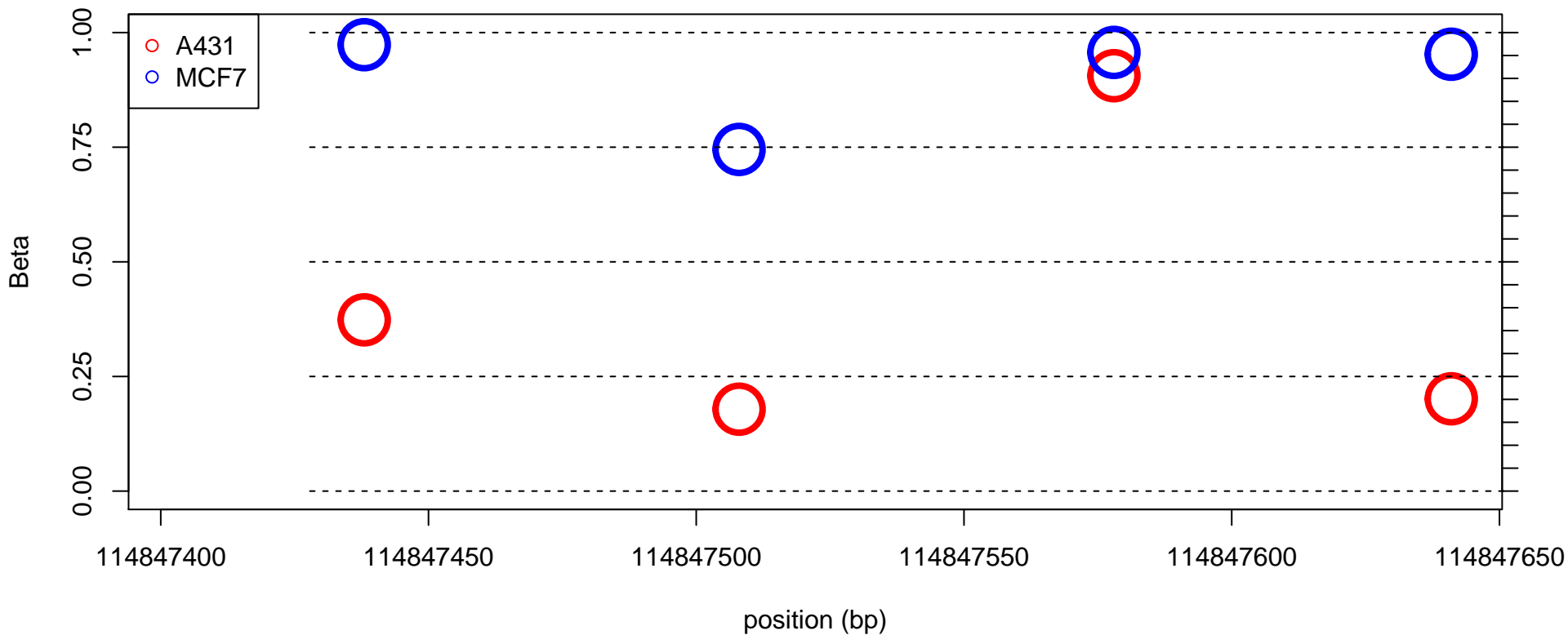

Supplement: Additional file 2 — DMRforPairs output for the comparison of A431-MCF7 and NA17018-NA17105. Please start from the HTML files in each folder. Available via the BMC Bioinformatics website. [file 1471-2105-15-141-S2.zip › 1394847754114233_MOESM2_ESM/A431_MCF7/figures/10035.pdf]

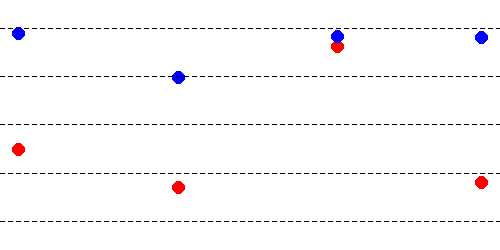

Supplement: Additional file 2 — DMRforPairs output for the comparison of A431-MCF7 and NA17018-NA17105. Please start from the HTML files in each folder. Available via the BMC Bioinformatics website. [file 1471-2105-15-141-S2.zip › 1394847754114233_MOESM2_ESM/A431_MCF7/figures/10035.png]

RegionID: 10036, chr12:115122028–115122304–M\_values

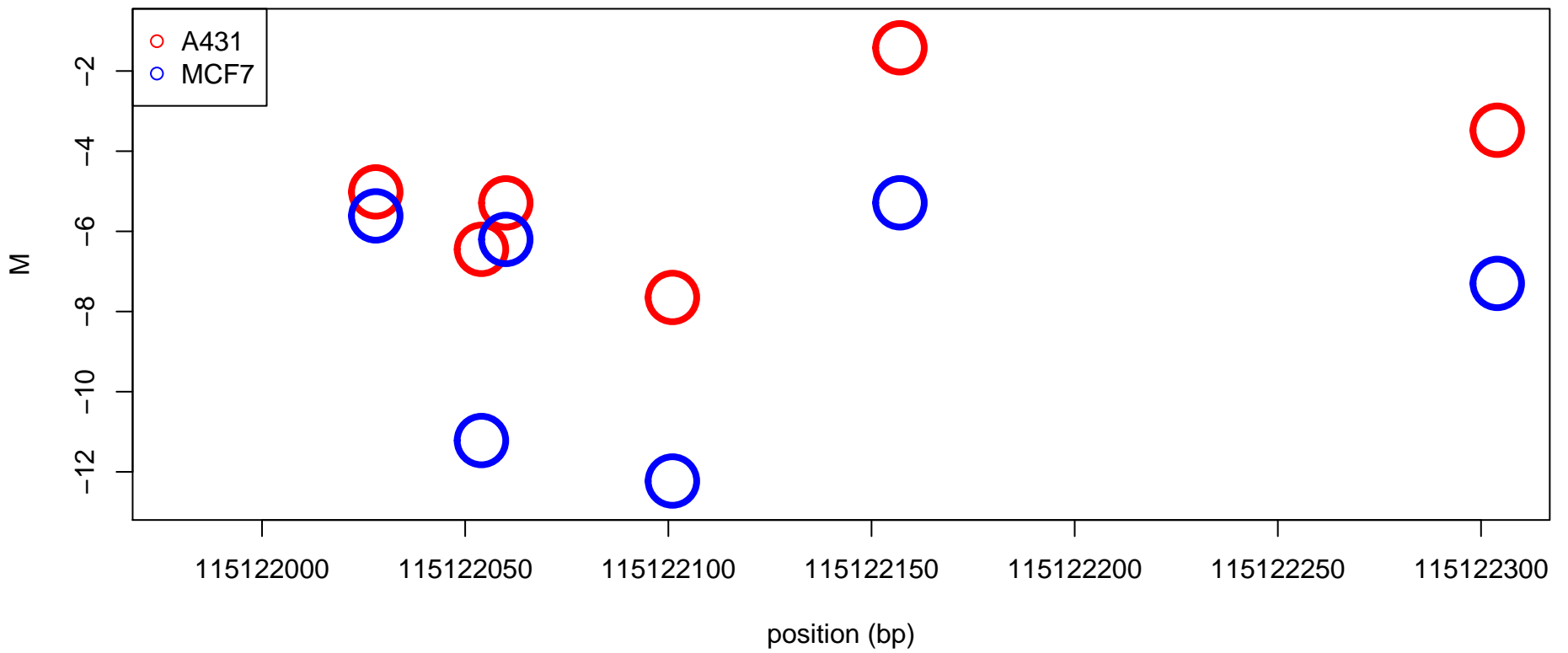

RegionID: 10036, chr12:115122028–115122304–Beta\_values

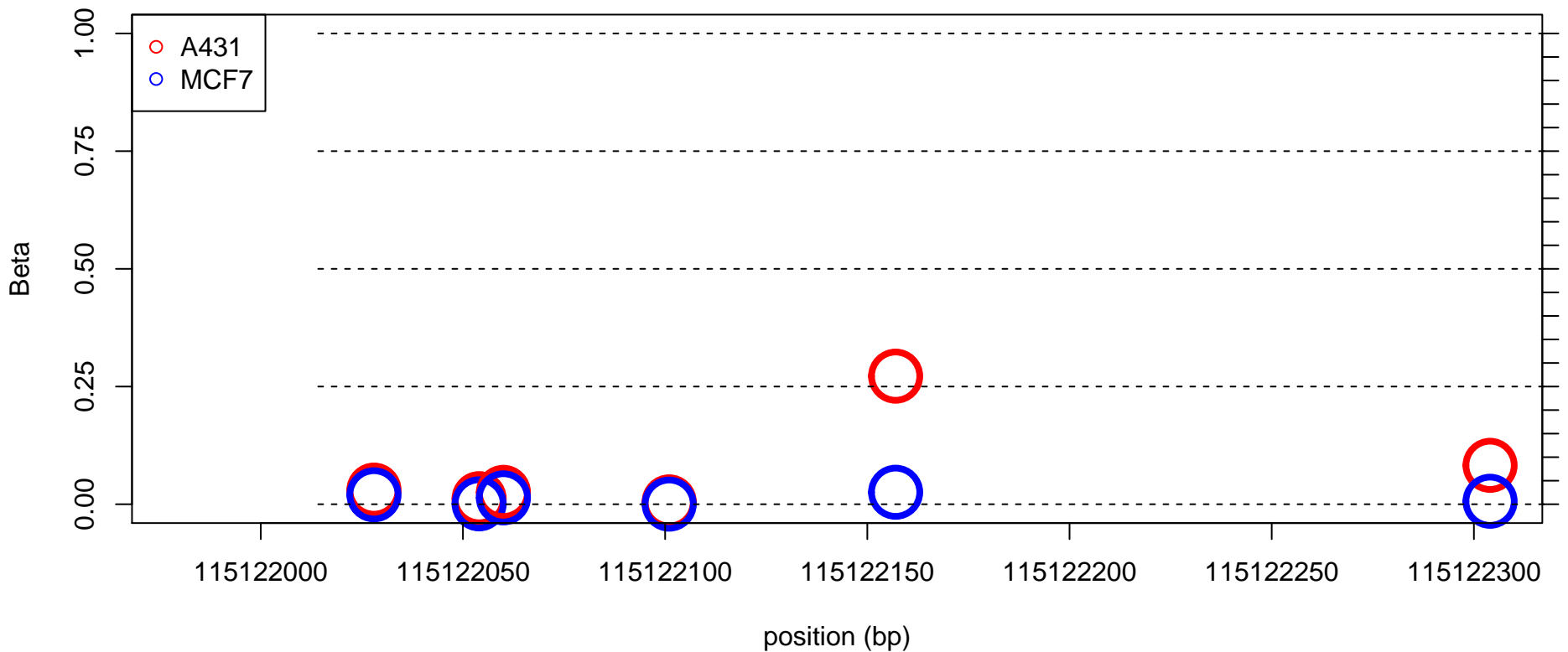

Supplement: Additional file 2 — DMRforPairs output for the comparison of A431-MCF7 and NA17018-NA17105. Please start from the HTML files in each folder. Available via the BMC Bioinformatics website. [file 1471-2105-15-141-S2.zip › 1394847754114233_MOESM2_ESM/A431_MCF7/figures/10036.pdf]

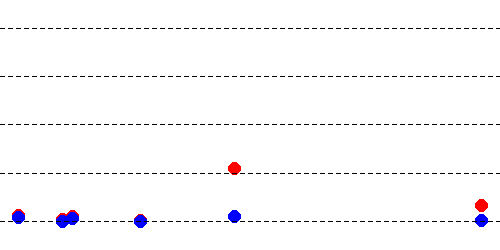

Supplement: Additional file 2 — DMRforPairs output for the comparison of A431-MCF7 and NA17018-NA17105. Please start from the HTML files in each folder. Available via the BMC Bioinformatics website. [file 1471-2105-15-141-S2.zip › 1394847754114233_MOESM2_ESM/A431_MCF7/figures/10036.png]

RegionID: 10037, chr12:116970856–116971165–M\_values

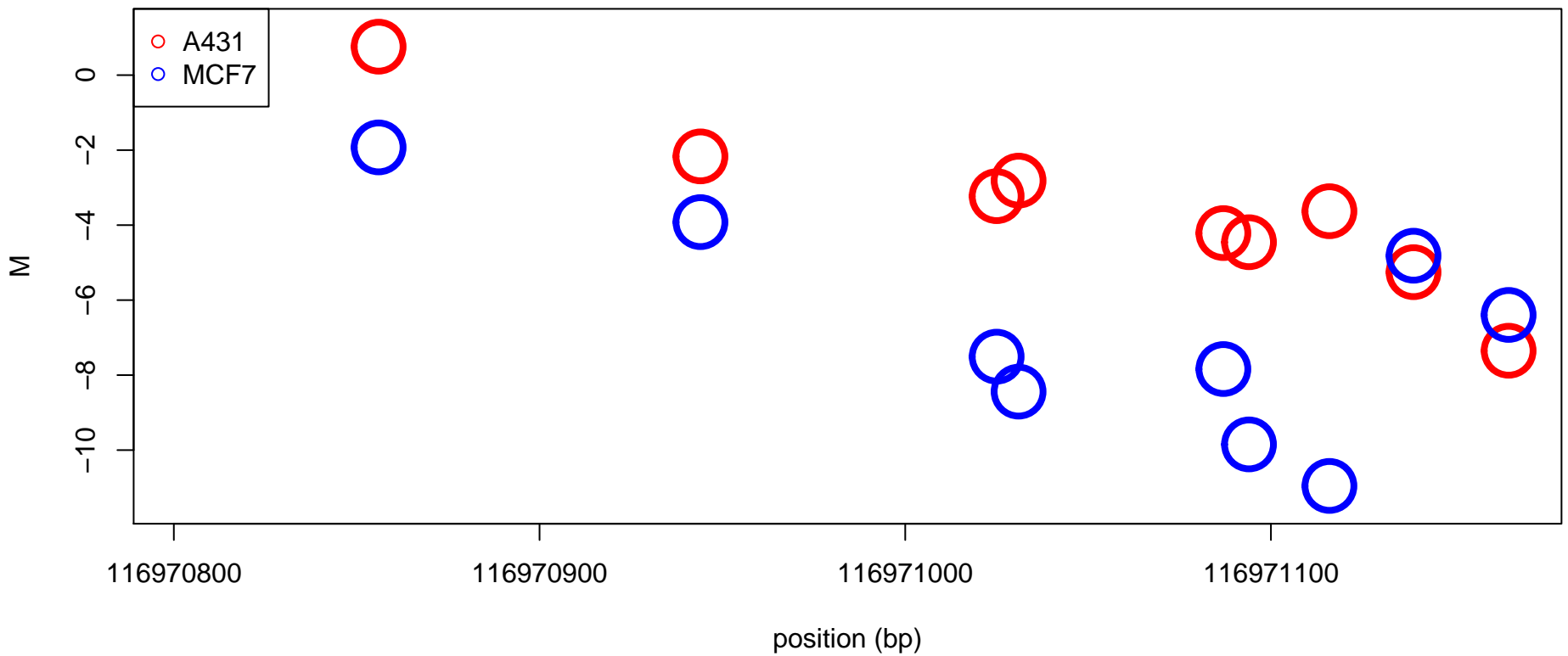

RegionID: 10037, chr12:116970856–116971165–Beta\_values

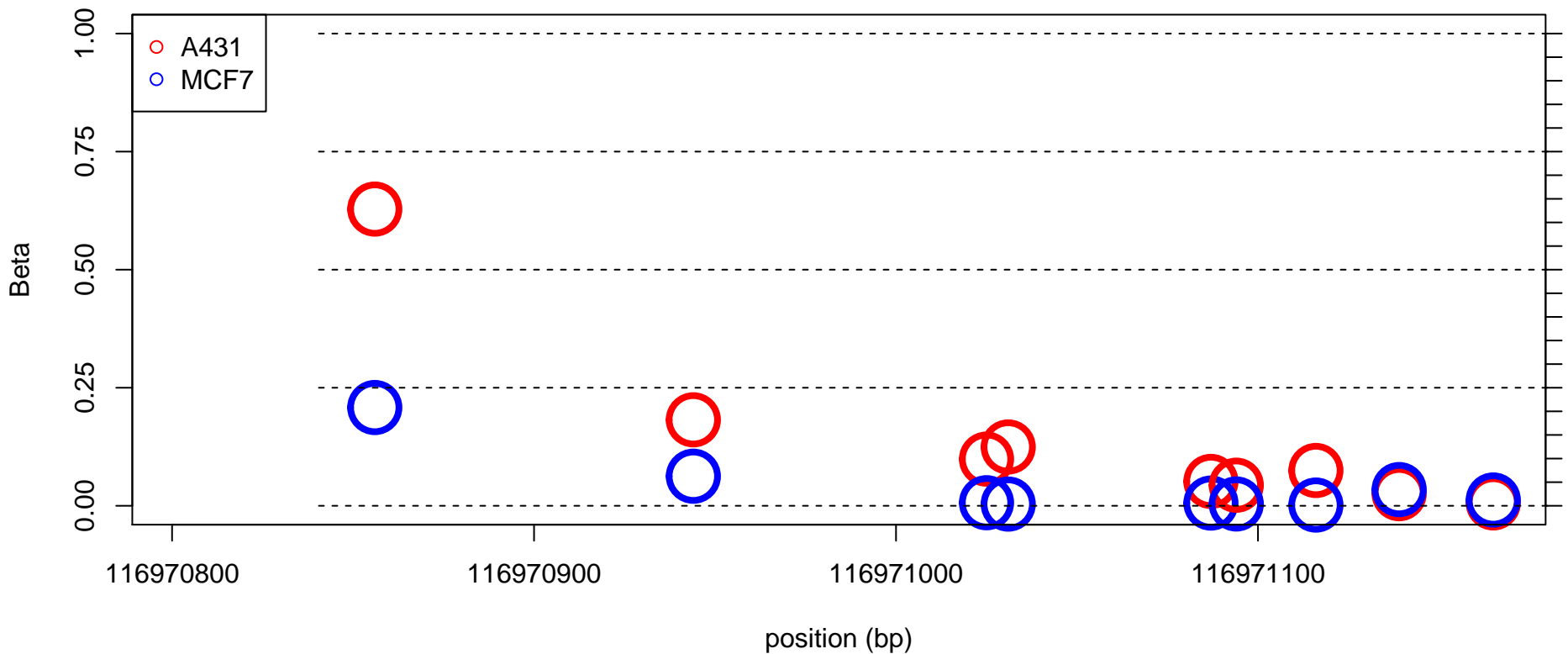

Supplement: Additional file 2 — DMRforPairs output for the comparison of A431-MCF7 and NA17018-NA17105. Please start from the HTML files in each folder. Available via the BMC Bioinformatics website. [file 1471-2105-15-141-S2.zip › 1394847754114233_MOESM2_ESM/A431_MCF7/figures/10037.pdf]

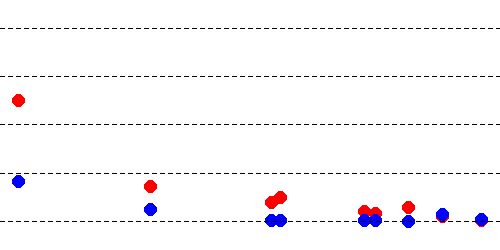

Supplement: Additional file 2 — DMRforPairs output for the comparison of A431-MCF7 and NA17018-NA17105. Please start from the HTML files in each folder. Available via the BMC Bioinformatics website. [file 1471-2105-15-141-S2.zip › 1394847754114233_MOESM2_ESM/A431_MCF7/figures/10037.png]

RegionID: 10038, chr12:116996773–116997095–M\_values

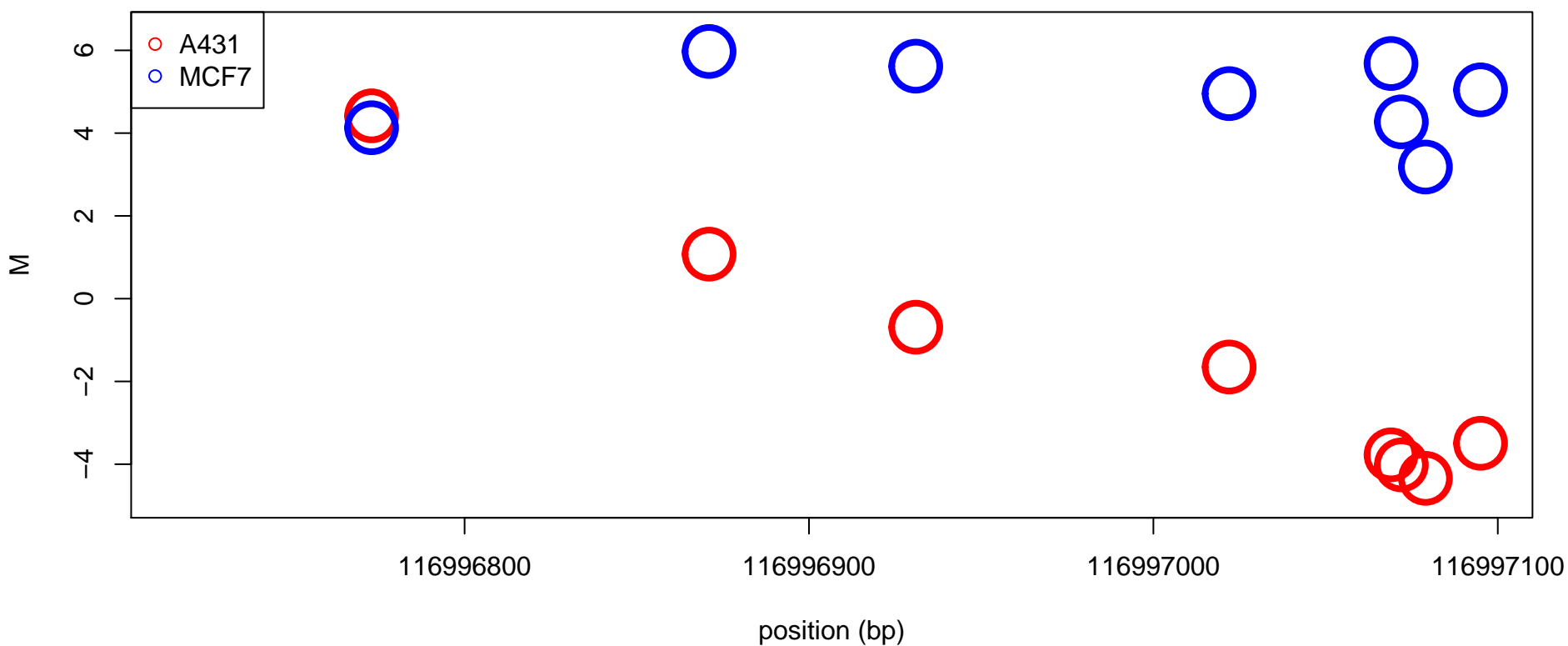

RegionID: 10038, chr12:116996773–116997095–Beta\_values

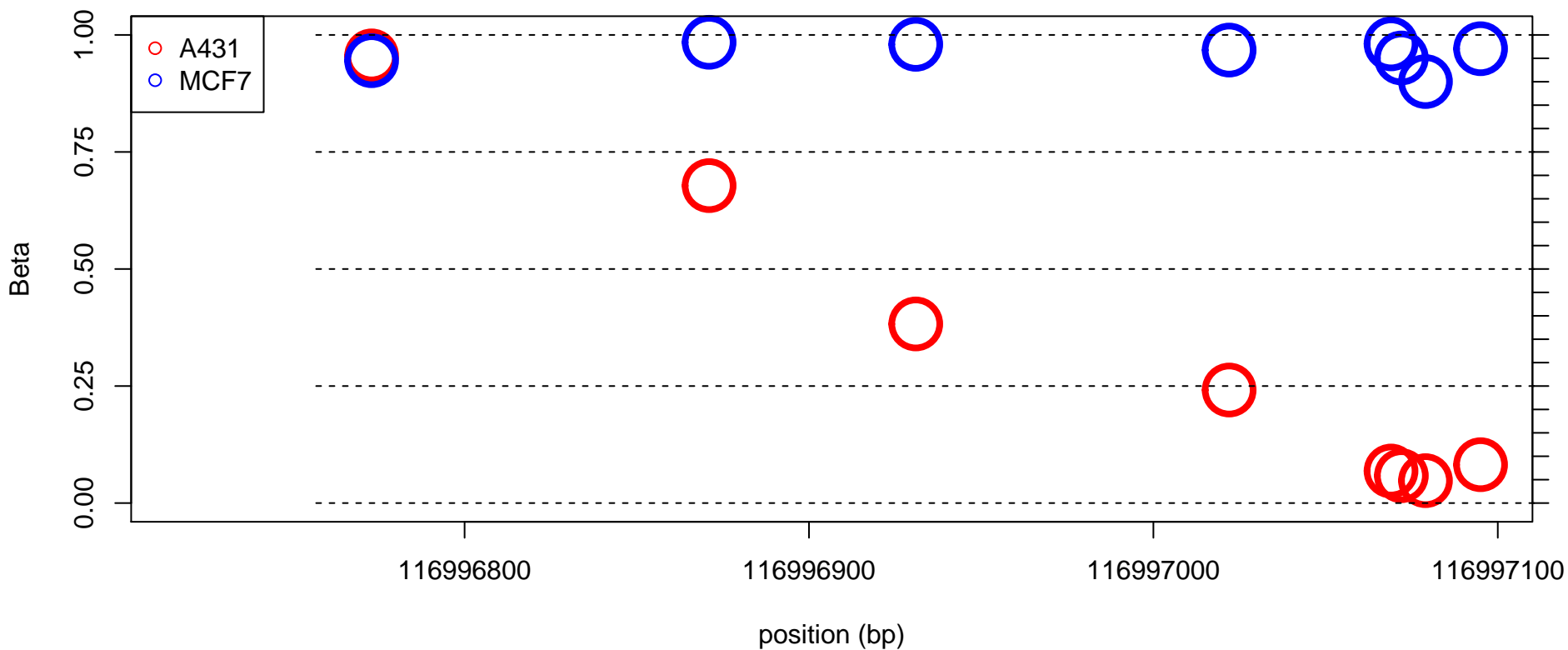

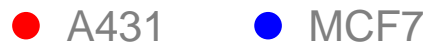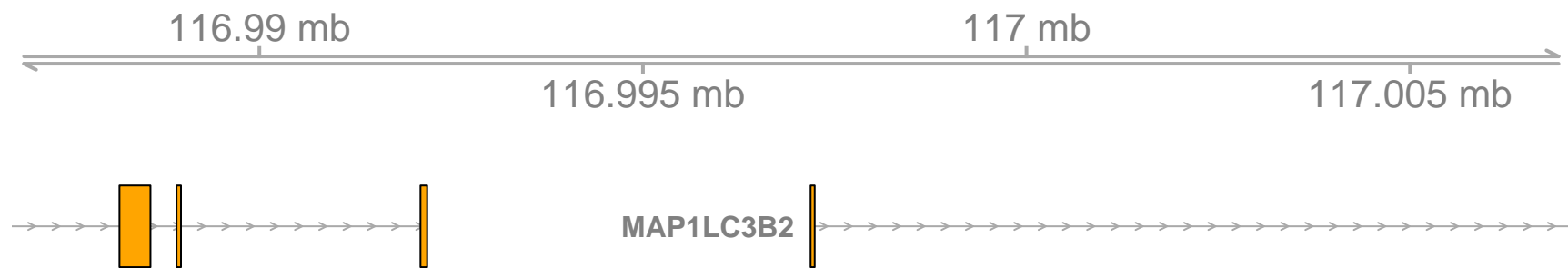

Supplement: Additional file 2 — DMRforPairs output for the comparison of A431-MCF7 and NA17018-NA17105. Please start from the HTML files in each folder. Available via the BMC Bioinformatics website. [file 1471-2105-15-141-S2.zip › 1394847754114233_MOESM2_ESM/A431_MCF7/figures/10038.pdf]

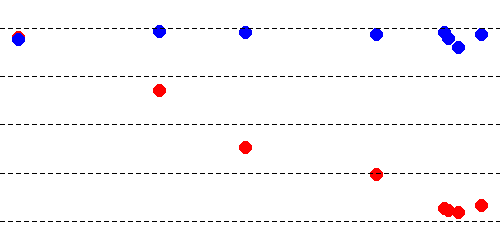

Supplement: Additional file 2 — DMRforPairs output for the comparison of A431-MCF7 and NA17018-NA17105. Please start from the HTML files in each folder. Available via the BMC Bioinformatics website. [file 1471-2105-15-141-S2.zip › 1394847754114233_MOESM2_ESM/A431_MCF7/figures/10038.png]

RegionID: 10041, chr12:117319785–117320316–M\_values

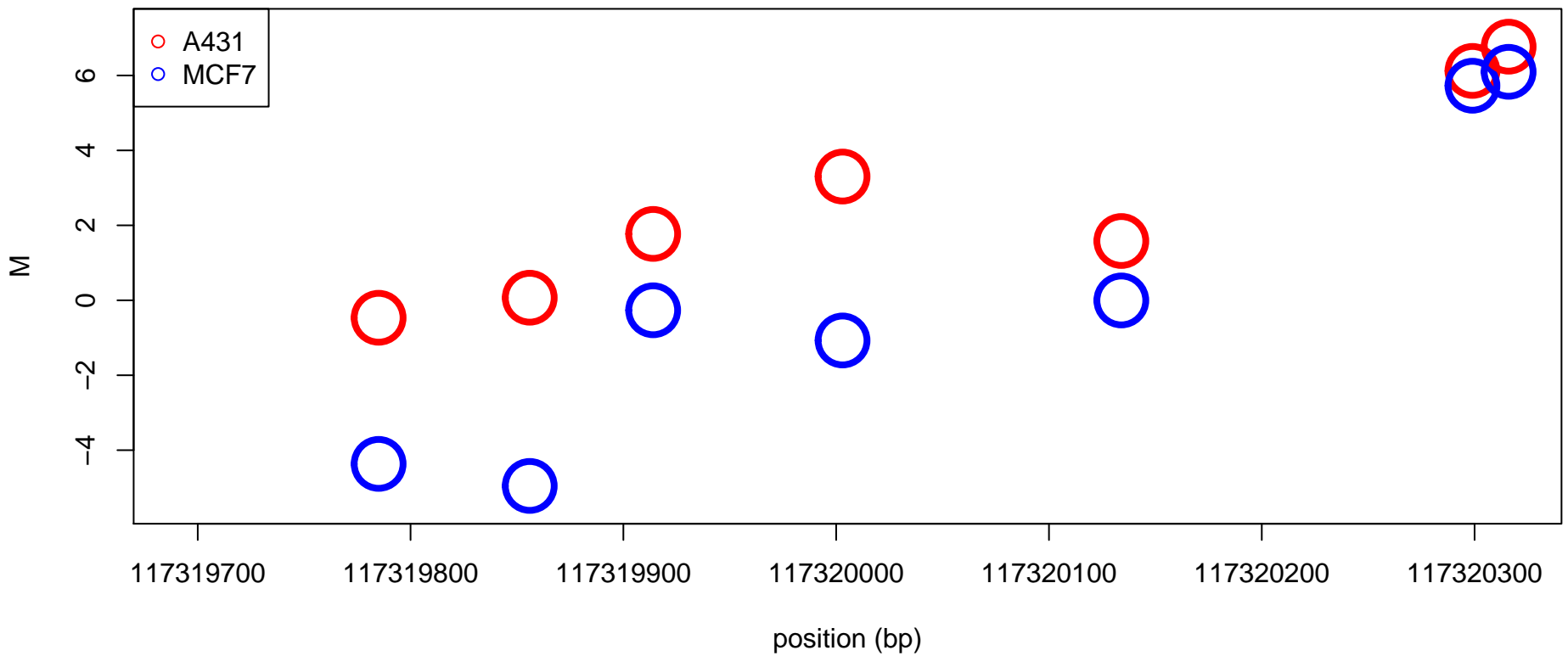

RegionID: 10041, chr12:117319785–117320316–Beta\_values

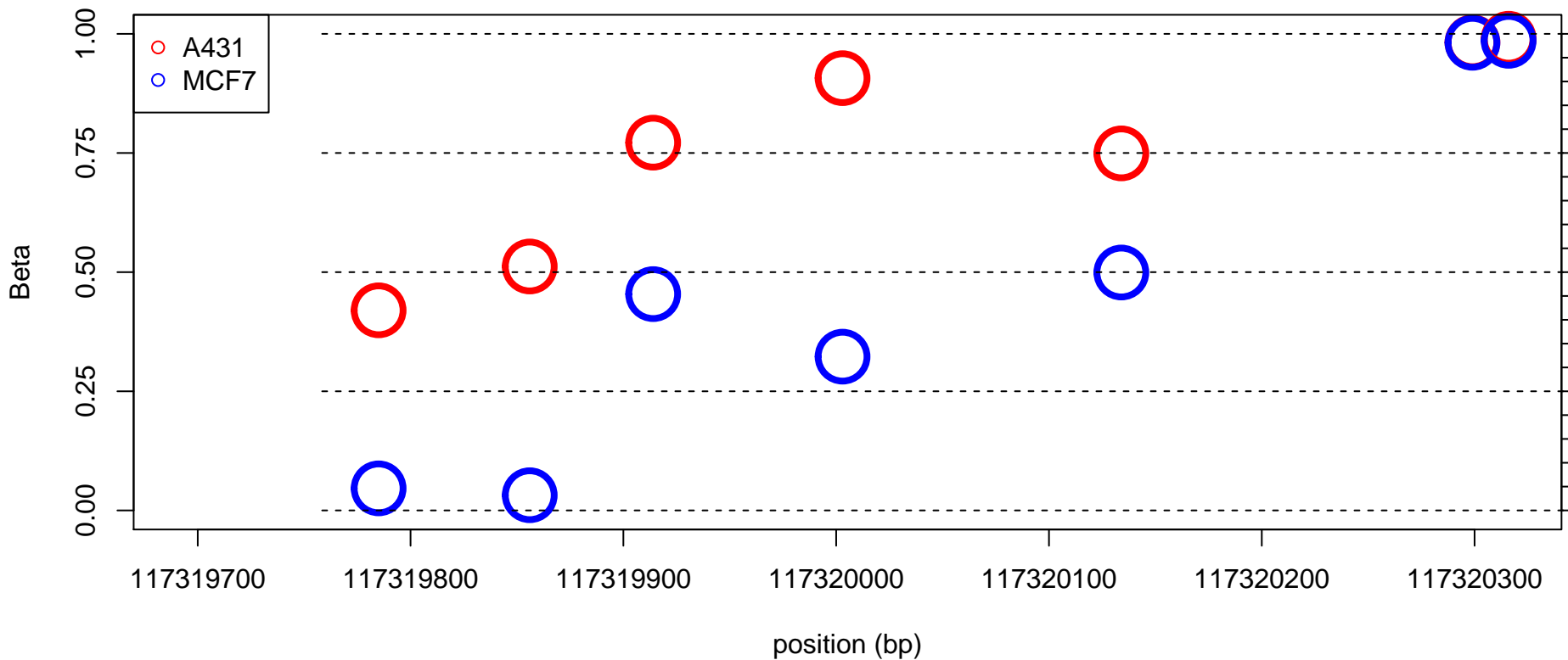

Supplement: Additional file 2 — DMRforPairs output for the comparison of A431-MCF7 and NA17018-NA17105. Please start from the HTML files in each folder. Available via the BMC Bioinformatics website. [file 1471-2105-15-141-S2.zip › 1394847754114233_MOESM2_ESM/A431_MCF7/figures/10041.pdf]

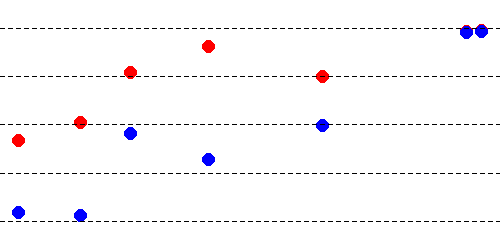

Supplement: Additional file 2 — DMRforPairs output for the comparison of A431-MCF7 and NA17018-NA17105. Please start from the HTML files in each folder. Available via the BMC Bioinformatics website. [file 1471-2105-15-141-S2.zip › 1394847754114233_MOESM2_ESM/A431_MCF7/figures/10041.png]

RegionID: 10043, chr12:117537264–117537710–M\_values

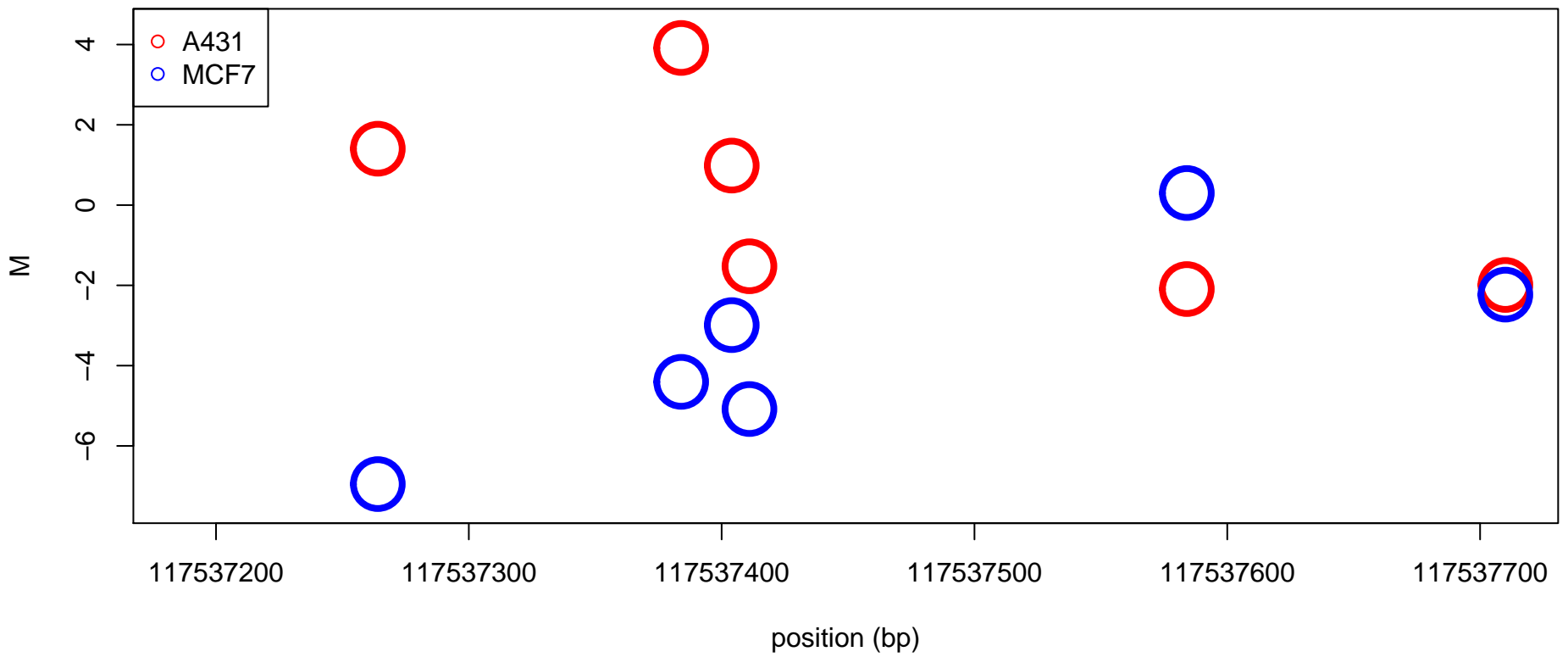

RegionID: 10043, chr12:117537264–117537710–Beta\_values

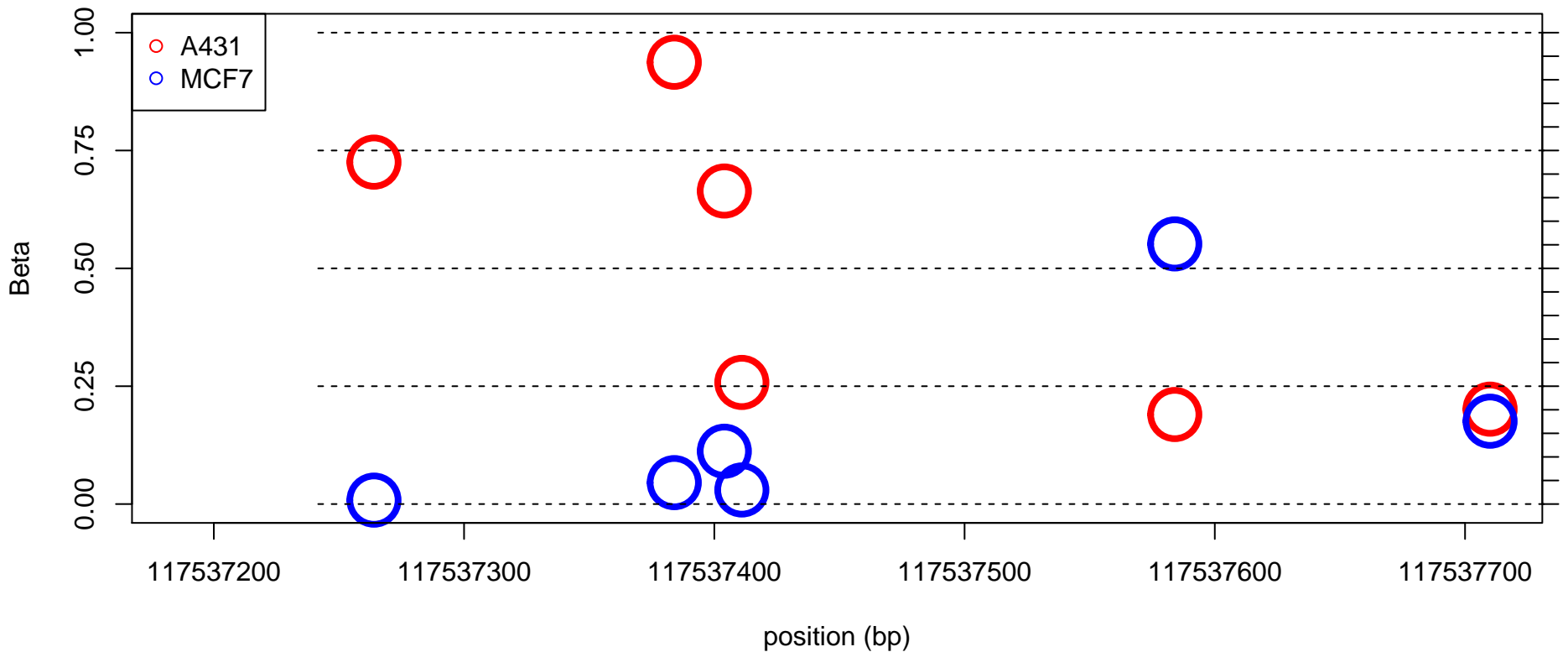

Supplement: Additional file 2 — DMRforPairs output for the comparison of A431-MCF7 and NA17018-NA17105. Please start from the HTML files in each folder. Available via the BMC Bioinformatics website. [file 1471-2105-15-141-S2.zip › 1394847754114233_MOESM2_ESM/A431_MCF7/figures/10043.pdf]

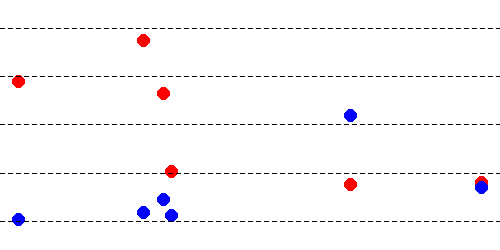

Supplement: Additional file 2 — DMRforPairs output for the comparison of A431-MCF7 and NA17018-NA17105. Please start from the HTML files in each folder. Available via the BMC Bioinformatics website. [file 1471-2105-15-141-S2.zip › 1394847754114233_MOESM2_ESM/A431_MCF7/figures/10043.png]

RegionID: 10044, chr12:117628347-117628589-M\_values

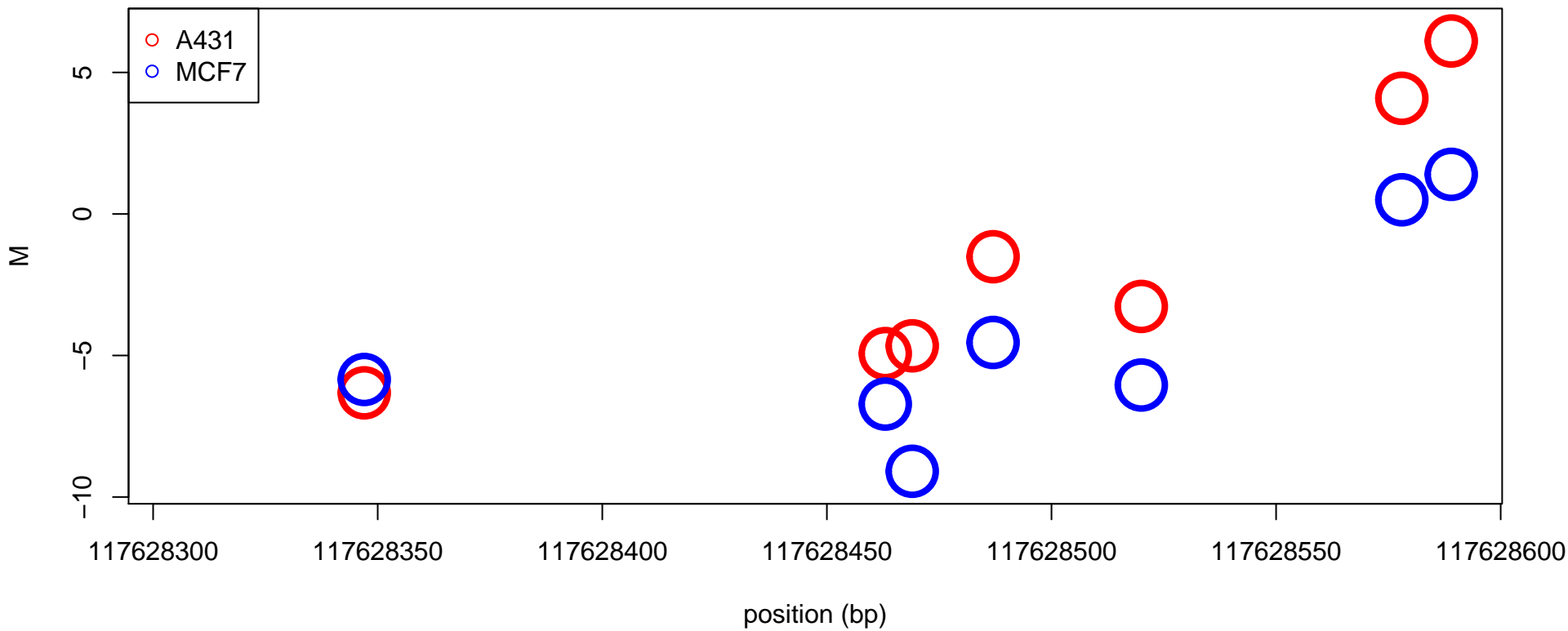

RegionID: 10044, chr12:117628347-117628589-Beta\_values

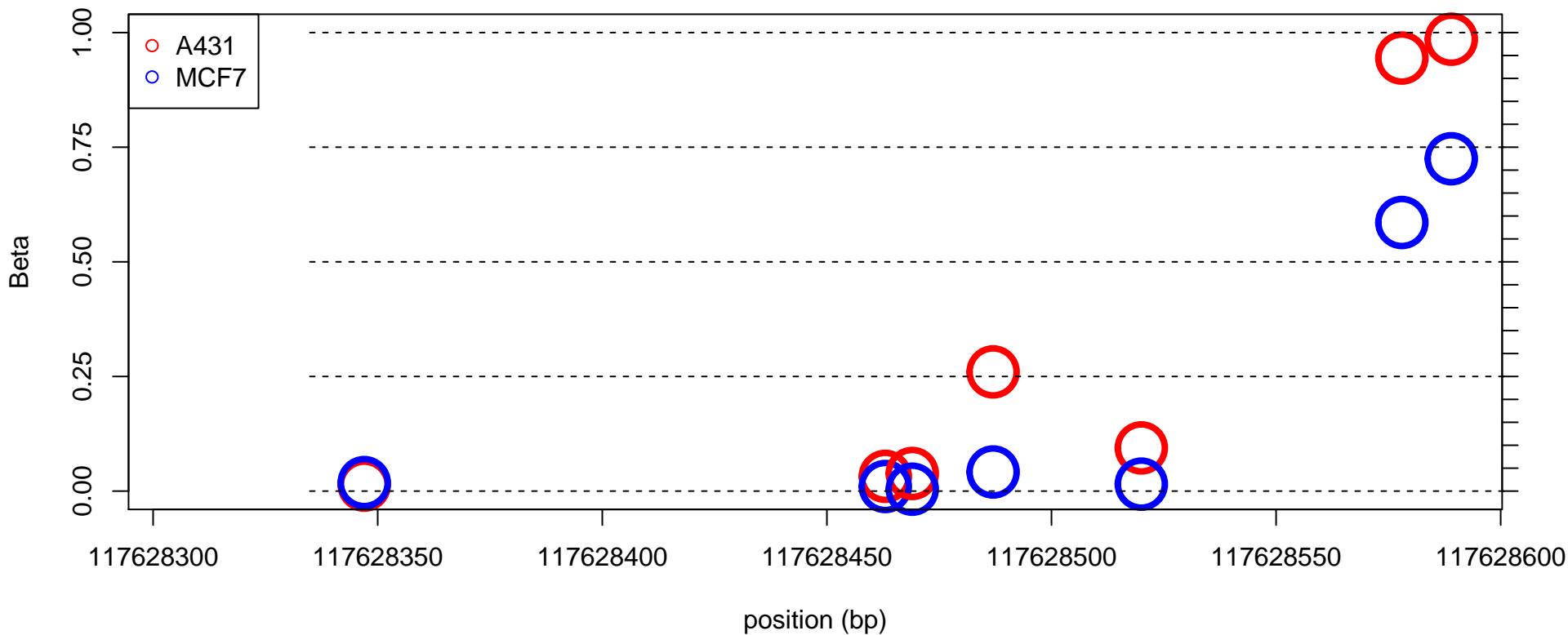

Supplement: Additional file 2 — DMRforPairs output for the comparison of A431-MCF7 and NA17018-NA17105. Please start from the HTML files in each folder. Available via the BMC Bioinformatics website. [file 1471-2105-15-141-S2.zip › 1394847754114233_MOESM2_ESM/A431_MCF7/figures/10044.pdf]

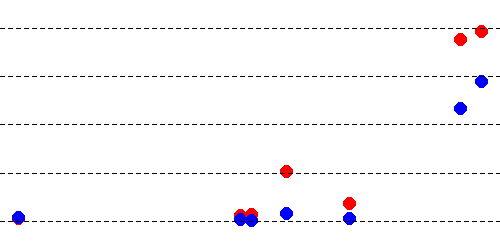

Supplement: Additional file 2 — DMRforPairs output for the comparison of A431-MCF7 and NA17018-NA17105. Please start from the HTML files in each folder. Available via the BMC Bioinformatics website. [file 1471-2105-15-141-S2.zip › 1394847754114233_MOESM2_ESM/A431_MCF7/figures/10044.png]

RegionID: 10045, chr12:118407009–118407487–M\_values

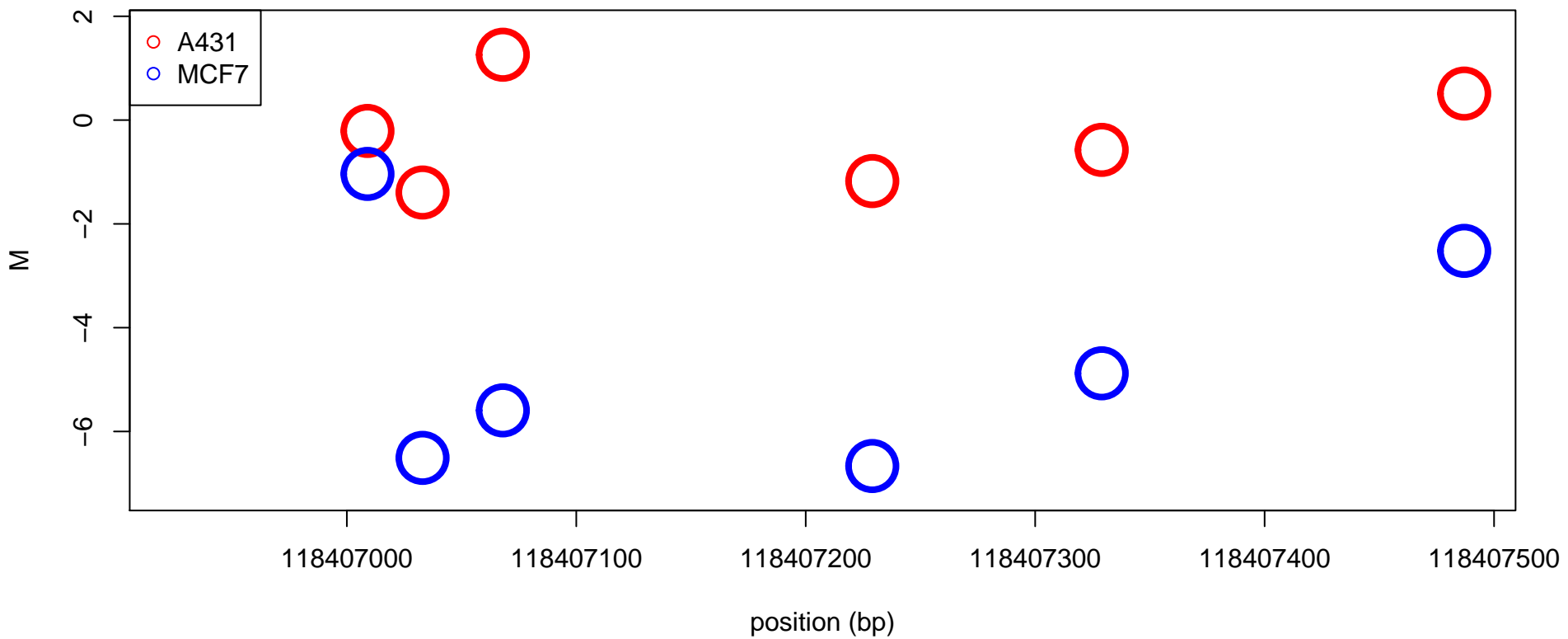

RegionID: 10045, chr12:118407009–118407487–Beta\_values

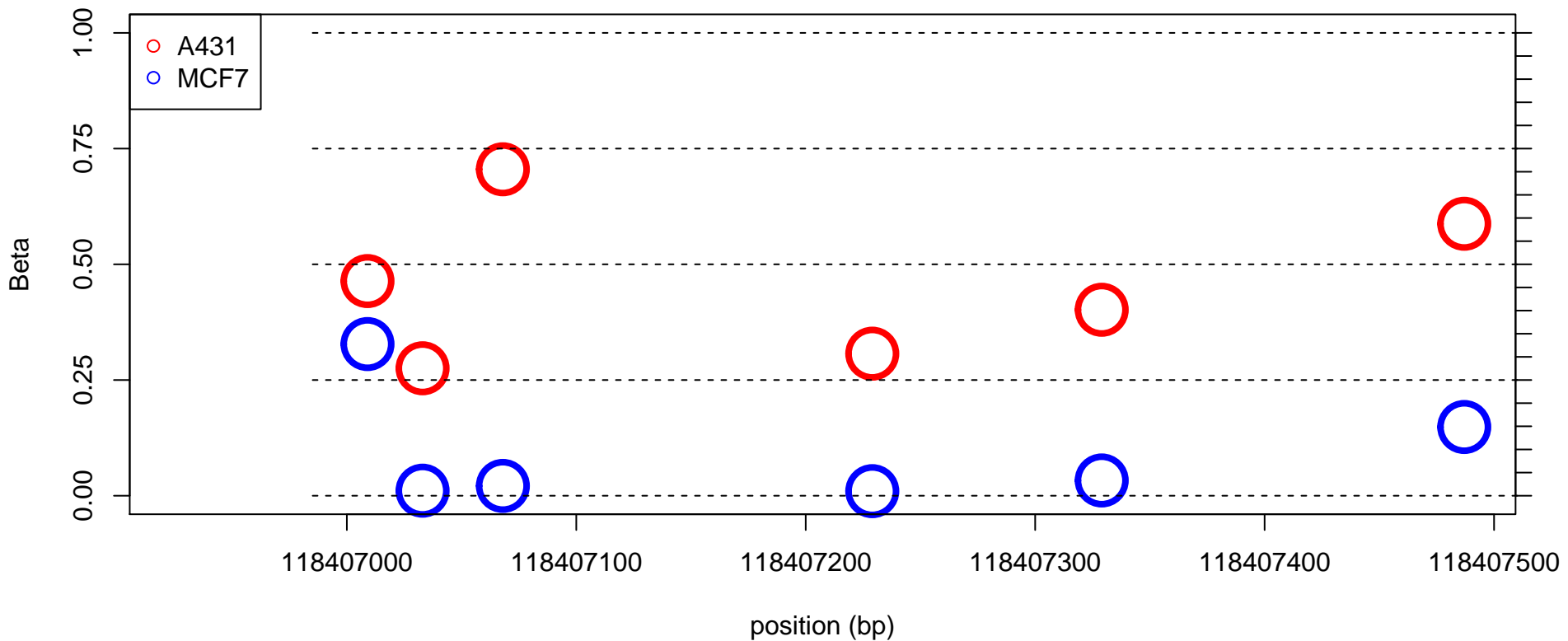

Chromosome 12

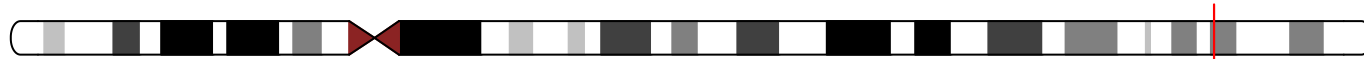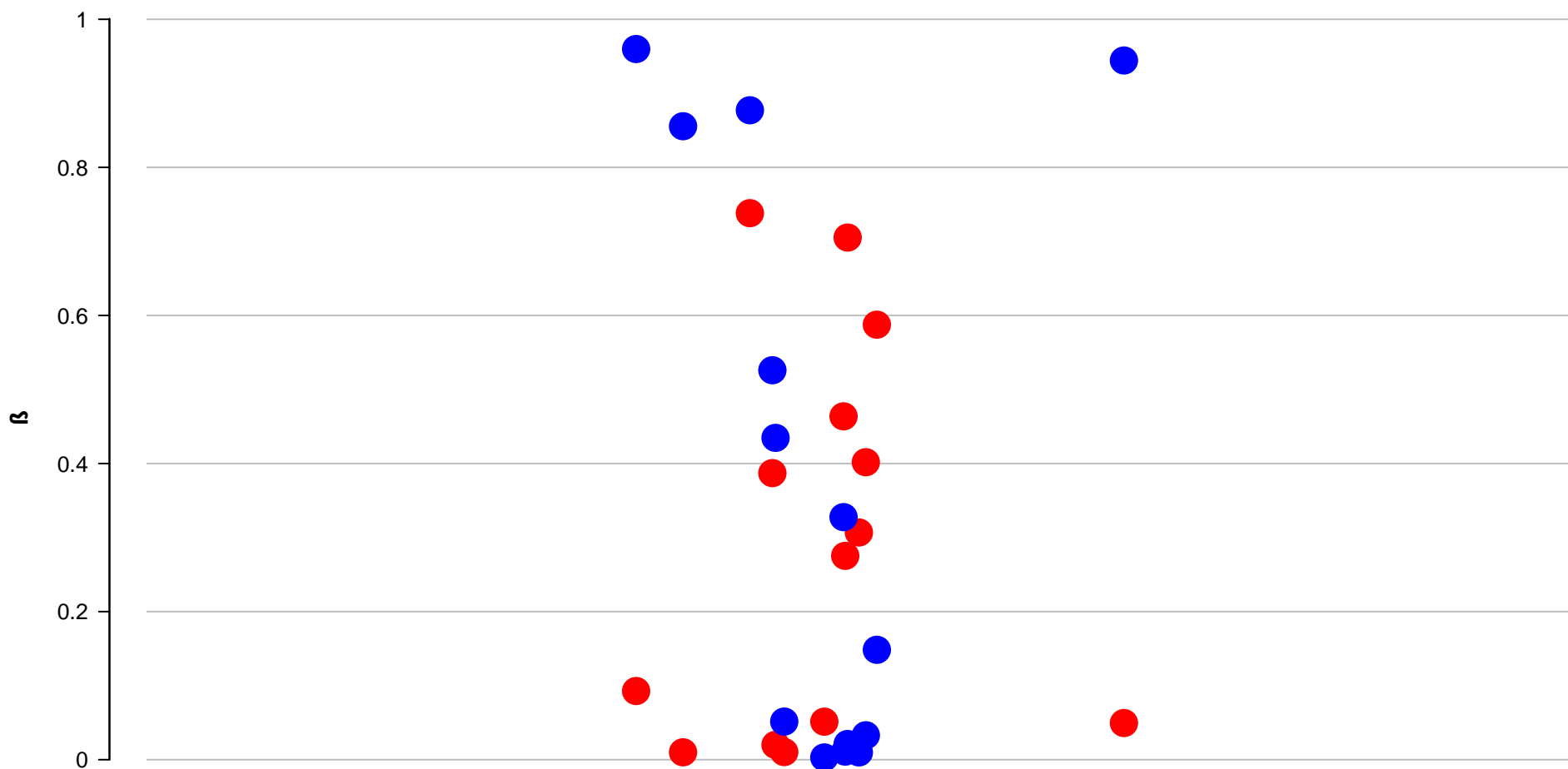

A431 MCF7

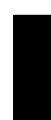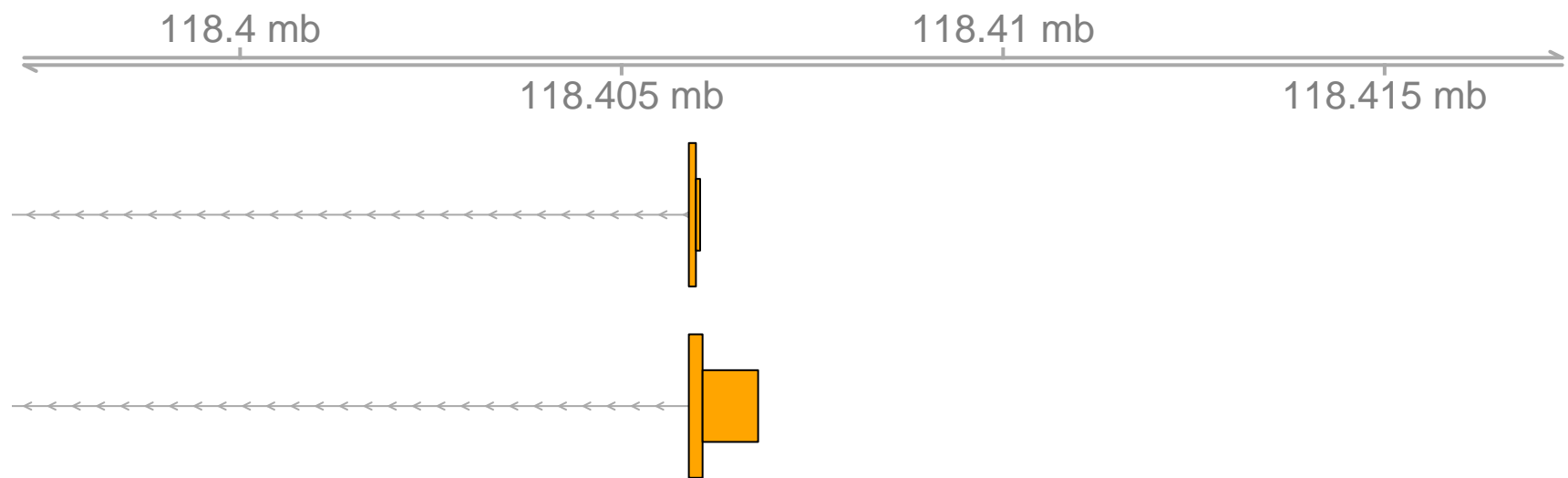

Supplement: Additional file 2 — DMRforPairs output for the comparison of A431-MCF7 and NA17018-NA17105. Please start from the HTML files in each folder. Available via the BMC Bioinformatics website. [file 1471-2105-15-141-S2.zip › 1394847754114233_MOESM2_ESM/A431_MCF7/figures/10045.pdf]

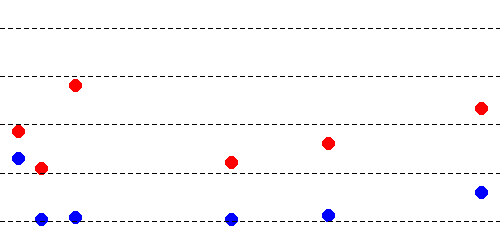

Supplement: Additional file 2 — DMRforPairs output for the comparison of A431-MCF7 and NA17018-NA17105. Please start from the HTML files in each folder. Available via the BMC Bioinformatics website. [file 1471-2105-15-141-S2.zip › 1394847754114233_MOESM2_ESM/A431_MCF7/figures/10045.png]

RegionID: 10049, chr12:119419106–119419331–M\_values

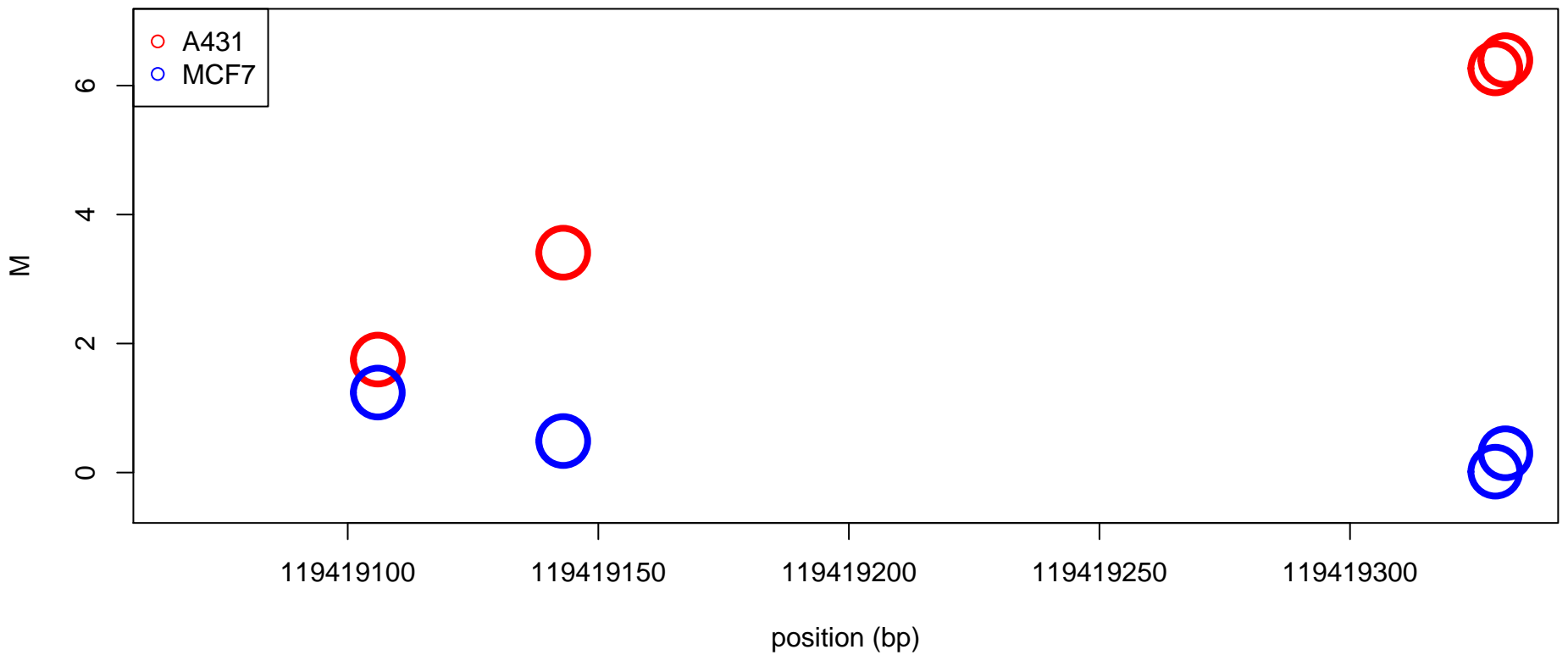

RegionID: 10049, chr12:119419106–119419331–Beta\_values

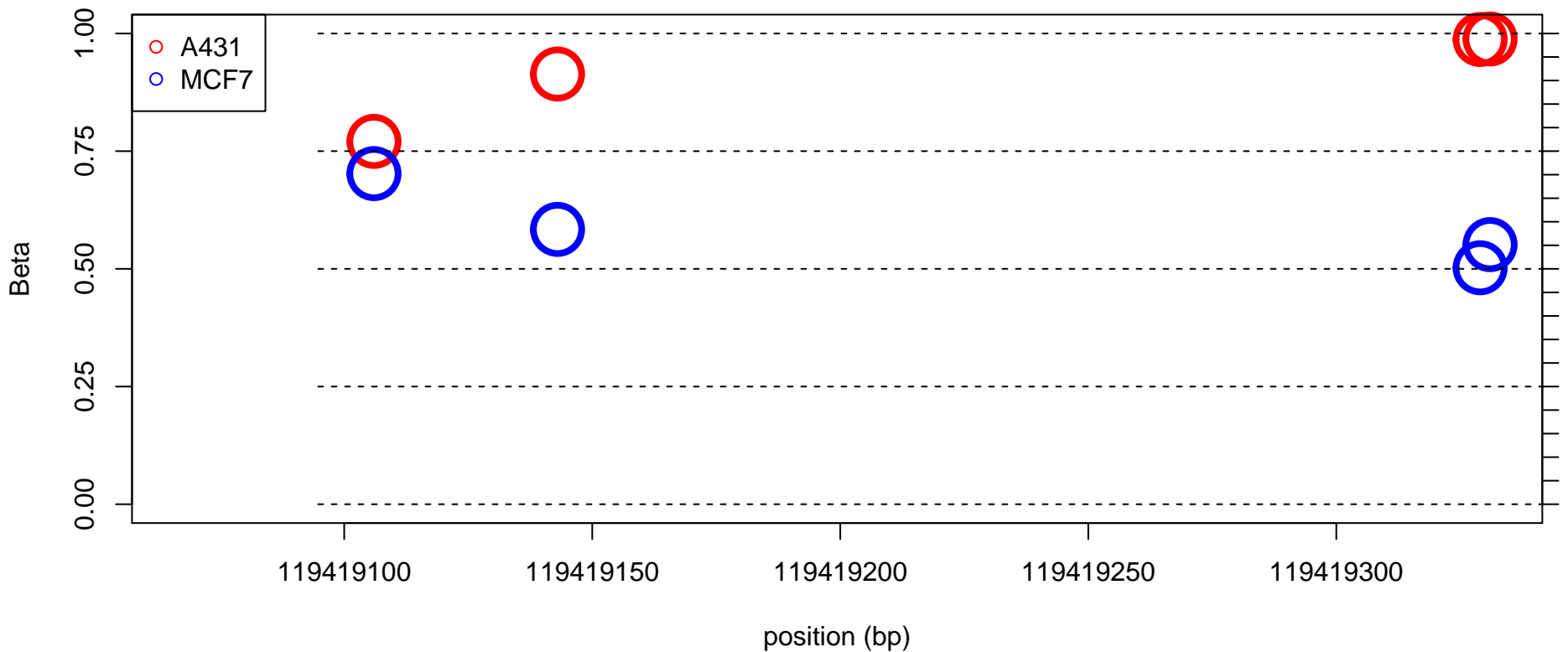

Supplement: Additional file 2 — DMRforPairs output for the comparison of A431-MCF7 and NA17018-NA17105. Please start from the HTML files in each folder. Available via the BMC Bioinformatics website. [file 1471-2105-15-141-S2.zip › 1394847754114233_MOESM2_ESM/A431_MCF7/figures/10049.pdf]

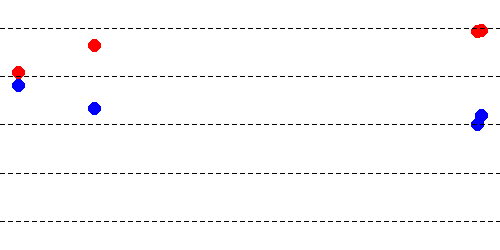

Supplement: Additional file 2 — DMRforPairs output for the comparison of A431-MCF7 and NA17018-NA17105. Please start from the HTML files in each folder. Available via the BMC Bioinformatics website. [file 1471-2105-15-141-S2.zip › 1394847754114233_MOESM2_ESM/A431_MCF7/figures/10049.png]

RegionID: 1005, chr1:214170114-214170376-M\_values

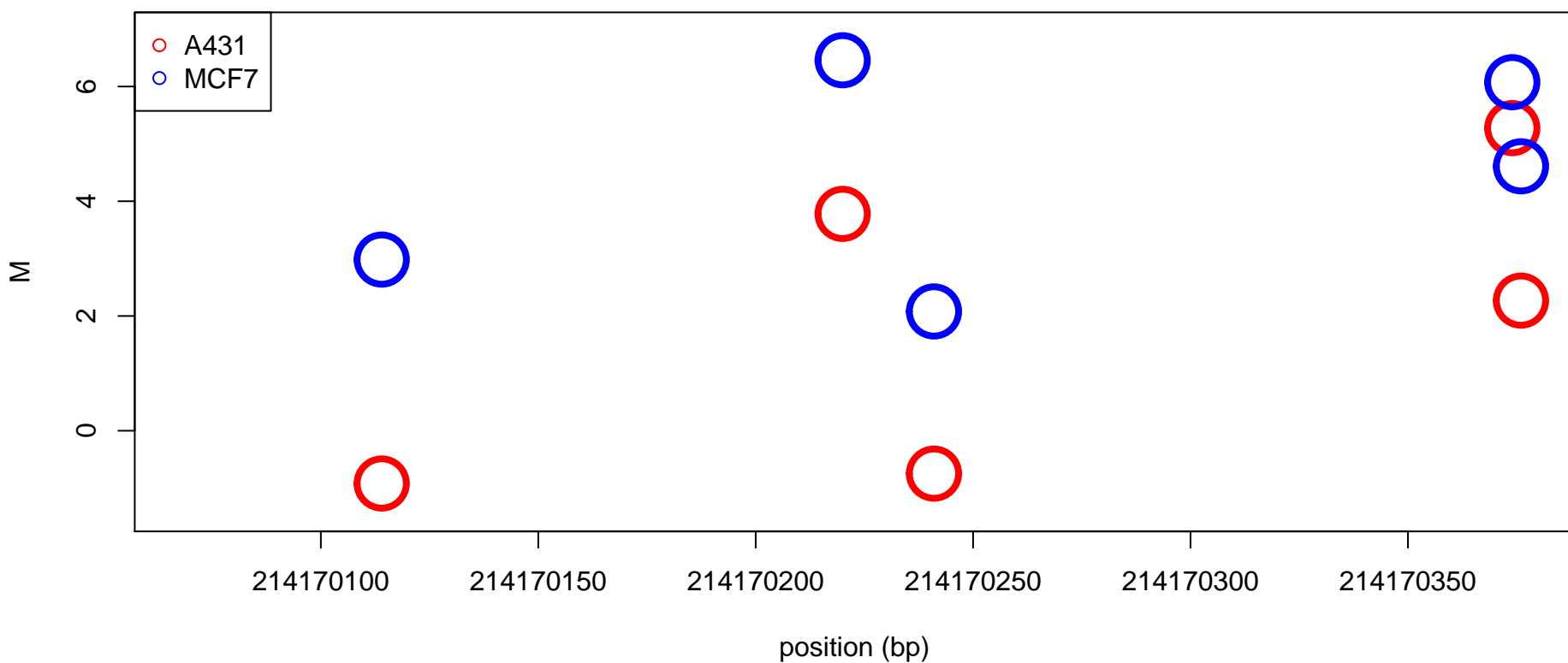

RegionID: 1005, chr1:214170114-214170376-Beta\_values

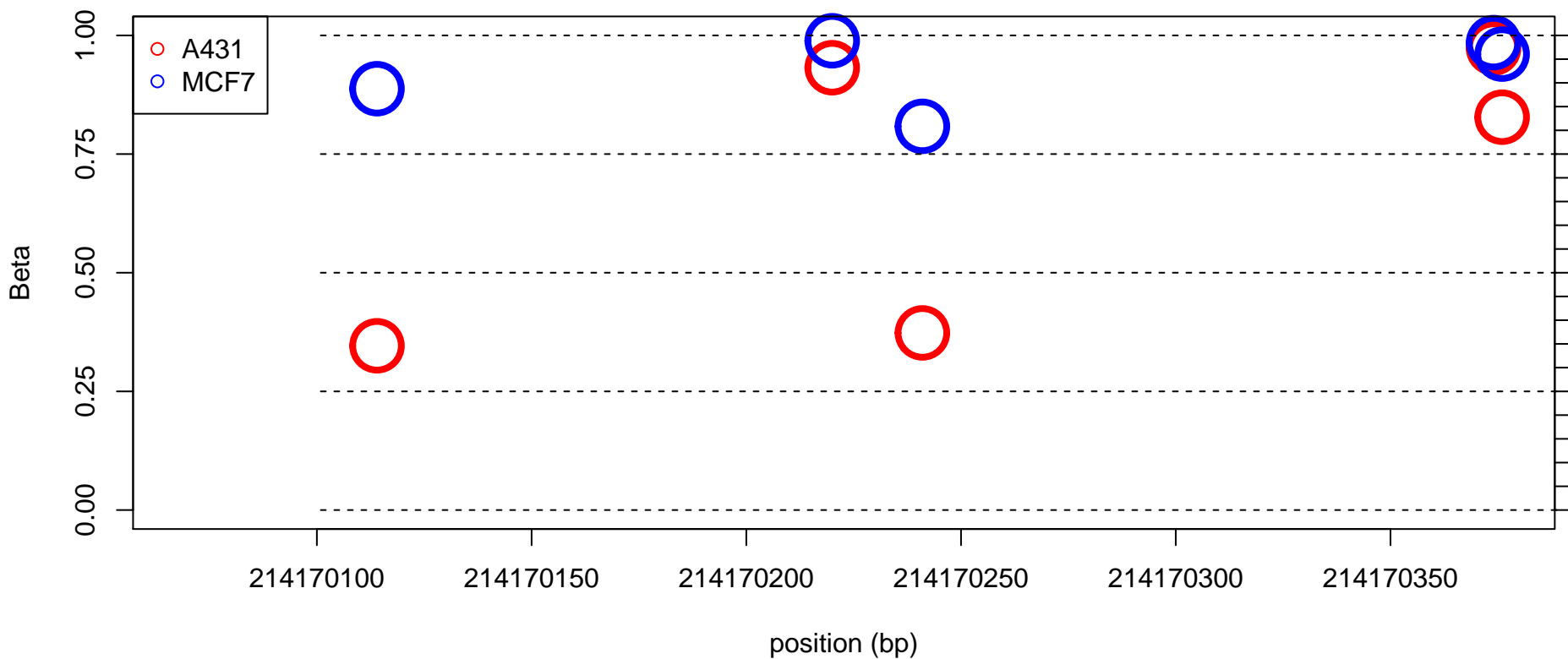

Supplement: Additional file 2 — DMRforPairs output for the comparison of A431-MCF7 and NA17018-NA17105. Please start from the HTML files in each folder. Available via the BMC Bioinformatics website. [file 1471-2105-15-141-S2.zip › 1394847754114233_MOESM2_ESM/A431_MCF7/figures/1005.pdf]

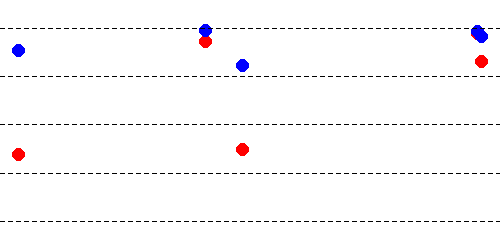

Supplement: Additional file 2 — DMRforPairs output for the comparison of A431-MCF7 and NA17018-NA17105. Please start from the HTML files in each folder. Available via the BMC Bioinformatics website. [file 1471-2105-15-141-S2.zip › 1394847754114233_MOESM2_ESM/A431_MCF7/figures/1005.png]

RegionID: 10050, chr12:119772354-119772421-M\_values

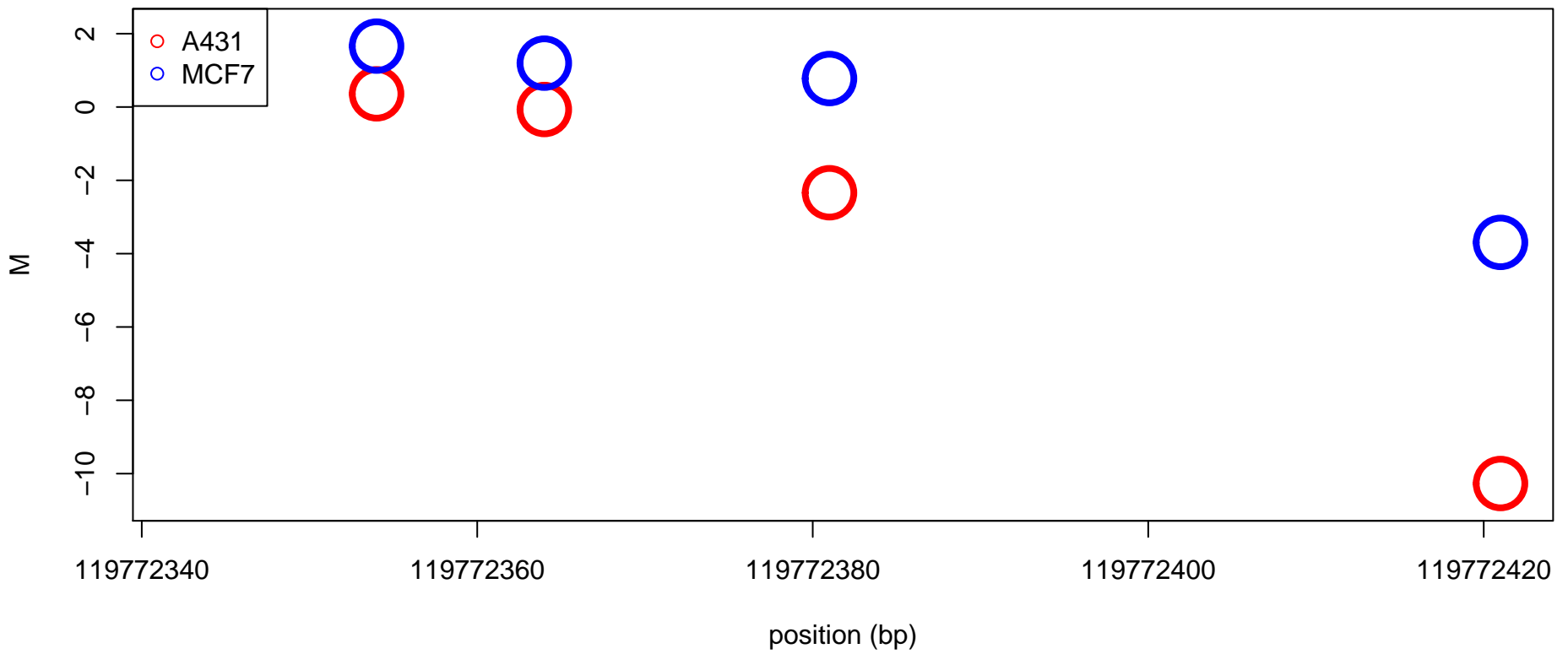

RegionID: 10050, chr12:119772354-119772421-Beta\_values

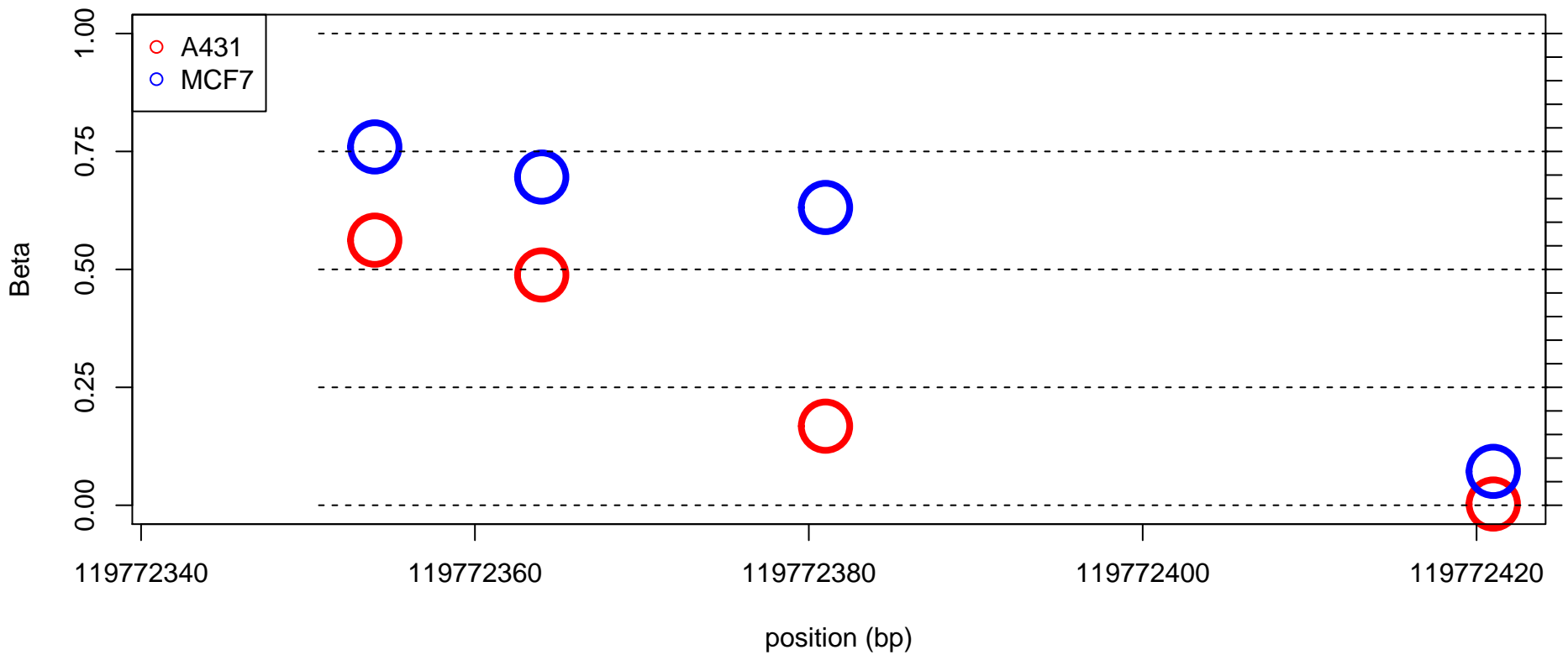

Supplement: Additional file 2 — DMRforPairs output for the comparison of A431-MCF7 and NA17018-NA17105. Please start from the HTML files in each folder. Available via the BMC Bioinformatics website. [file 1471-2105-15-141-S2.zip › 1394847754114233_MOESM2_ESM/A431_MCF7/figures/10050.pdf]

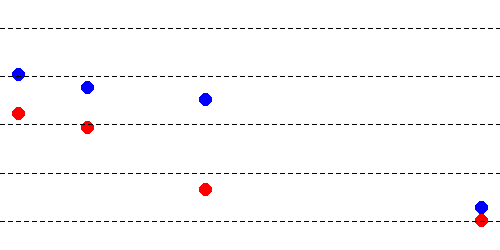

Supplement: Additional file 2 — DMRforPairs output for the comparison of A431-MCF7 and NA17018-NA17105. Please start from the HTML files in each folder. Available via the BMC Bioinformatics website. [file 1471-2105-15-141-S2.zip › 1394847754114233_MOESM2_ESM/A431_MCF7/figures/10050.png]

RegionID: 10056, chr12:120703729–120704006–M\_values

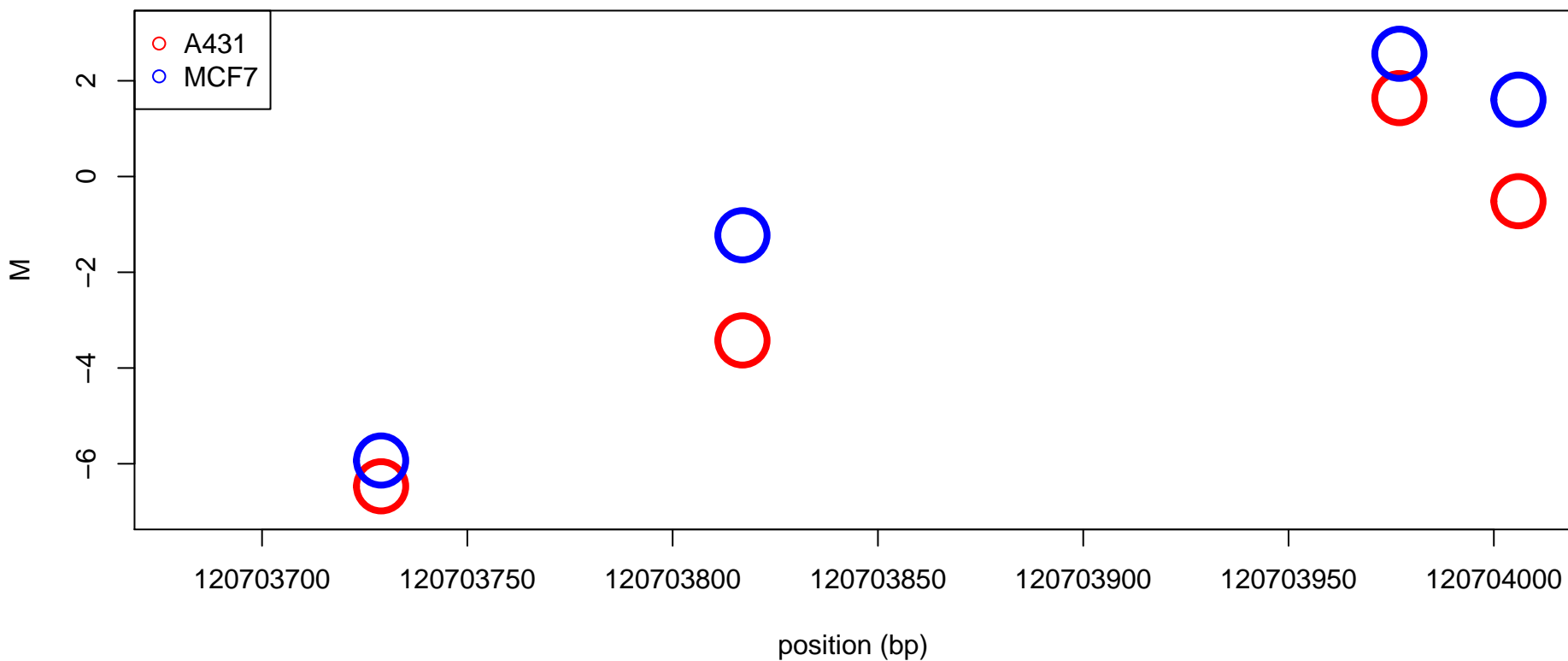

RegionID: 10056, chr12:120703729–120704006–Beta\_values

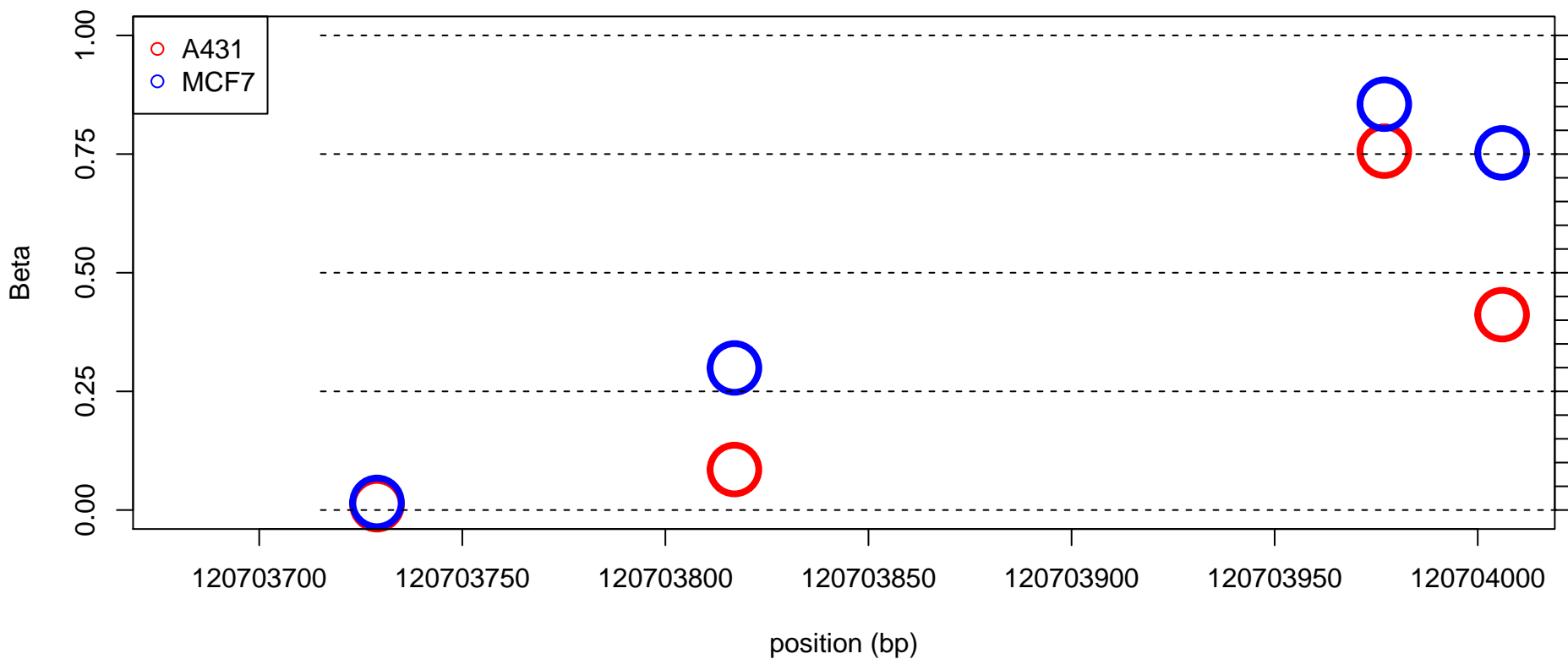

Supplement: Additional file 2 — DMRforPairs output for the comparison of A431-MCF7 and NA17018-NA17105. Please start from the HTML files in each folder. Available via the BMC Bioinformatics website. [file 1471-2105-15-141-S2.zip › 1394847754114233_MOESM2_ESM/A431_MCF7/figures/10056.pdf]

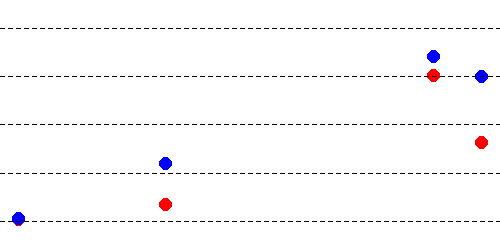

Supplement: Additional file 2 — DMRforPairs output for the comparison of A431-MCF7 and NA17018-NA17105. Please start from the HTML files in each folder. Available via the BMC Bioinformatics website. [file 1471-2105-15-141-S2.zip › 1394847754114233_MOESM2_ESM/A431_MCF7/figures/10056.png]

RegionID: 10058, chr12:120875539–120875898–M\_values

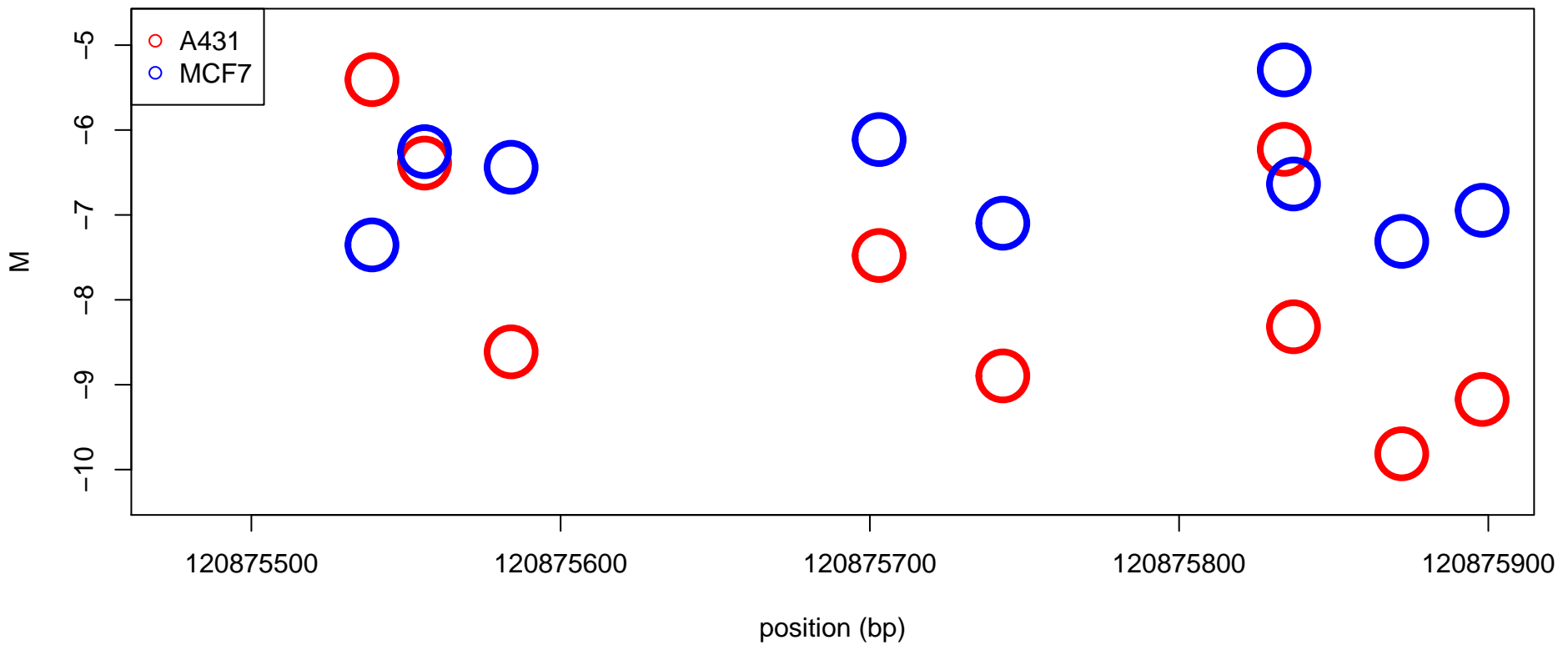

RegionID: 10058, chr12:120875539–120875898–Beta\_values

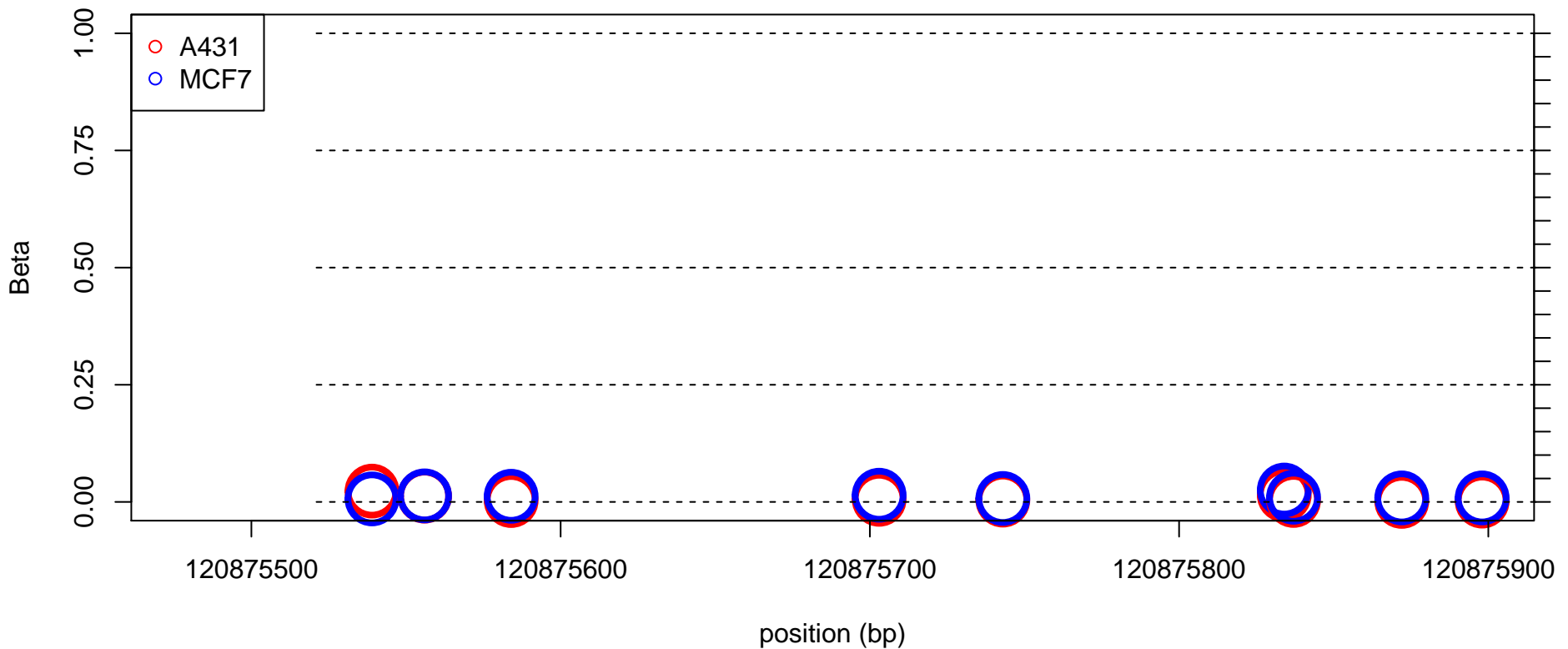

Supplement: Additional file 2 — DMRforPairs output for the comparison of A431-MCF7 and NA17018-NA17105. Please start from the HTML files in each folder. Available via the BMC Bioinformatics website. [file 1471-2105-15-141-S2.zip › 1394847754114233_MOESM2_ESM/A431_MCF7/figures/10058.pdf]

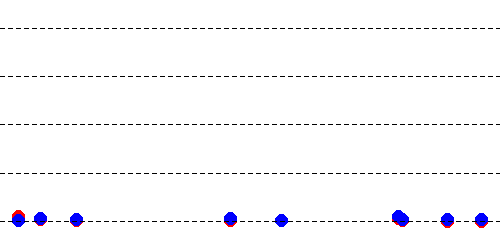

Supplement: Additional file 2 — DMRforPairs output for the comparison of A431-MCF7 and NA17018-NA17105. Please start from the HTML files in each folder. Available via the BMC Bioinformatics website. [file 1471-2105-15-141-S2.zip › 1394847754114233_MOESM2_ESM/A431_MCF7/figures/10058.png]

RegionID: 10061, chr12:120933726–120933845–M\_values

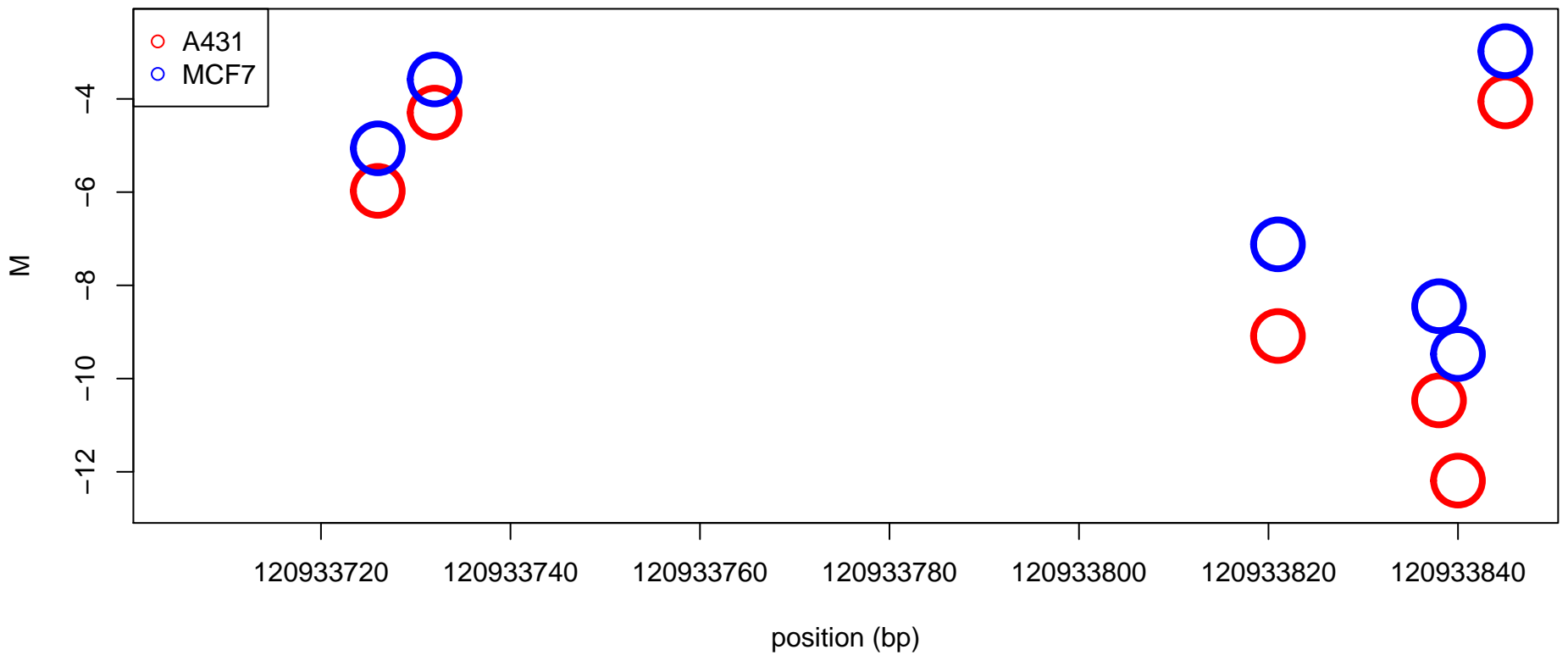

RegionID: 10061, chr12:120933726–120933845–Beta\_values

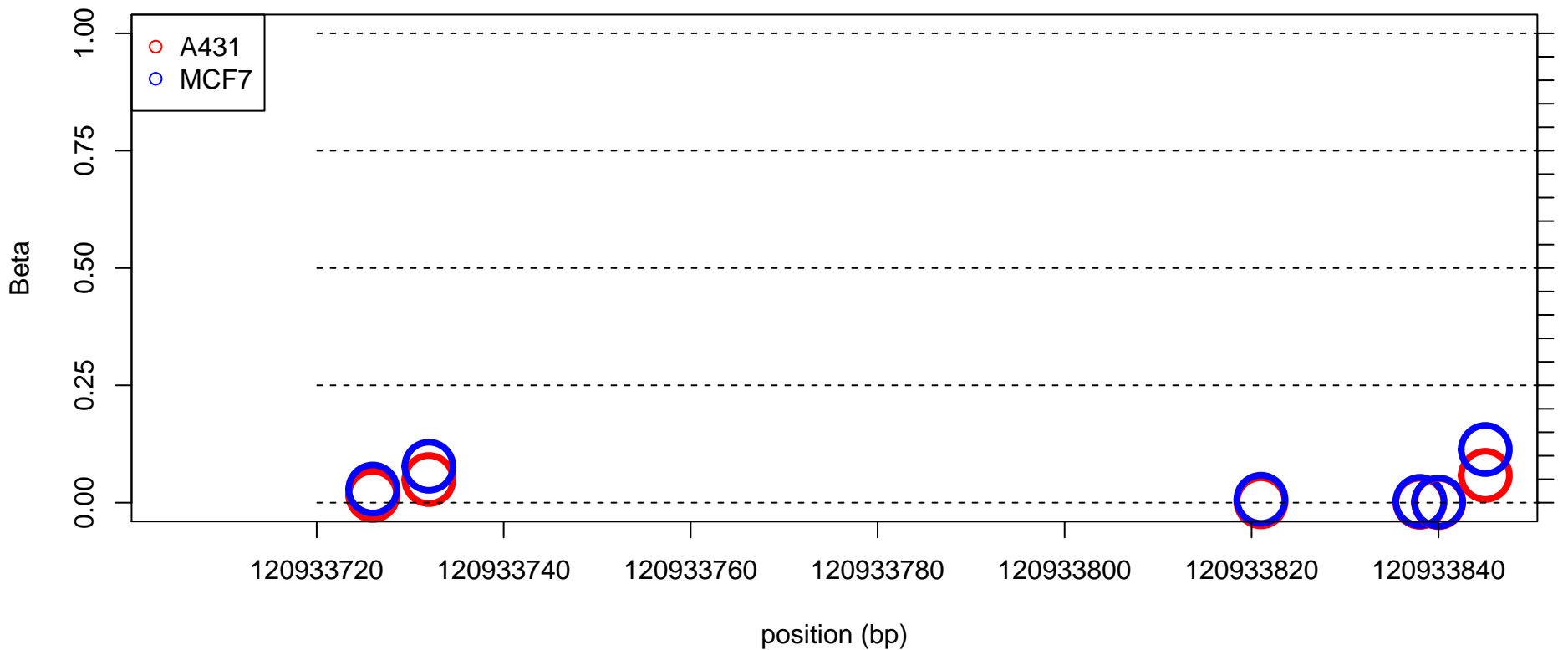

Supplement: Additional file 2 — DMRforPairs output for the comparison of A431-MCF7 and NA17018-NA17105. Please start from the HTML files in each folder. Available via the BMC Bioinformatics website. [file 1471-2105-15-141-S2.zip › 1394847754114233_MOESM2_ESM/A431_MCF7/figures/10061.pdf]

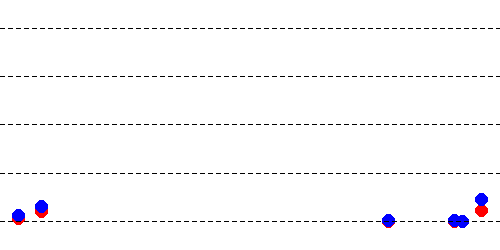

Supplement: Additional file 2 — DMRforPairs output for the comparison of A431-MCF7 and NA17018-NA17105. Please start from the HTML files in each folder. Available via the BMC Bioinformatics website. [file 1471-2105-15-141-S2.zip › 1394847754114233_MOESM2_ESM/A431_MCF7/figures/10061.png]

RegionID: 10086, chr12:123215151-123215684-M\_values

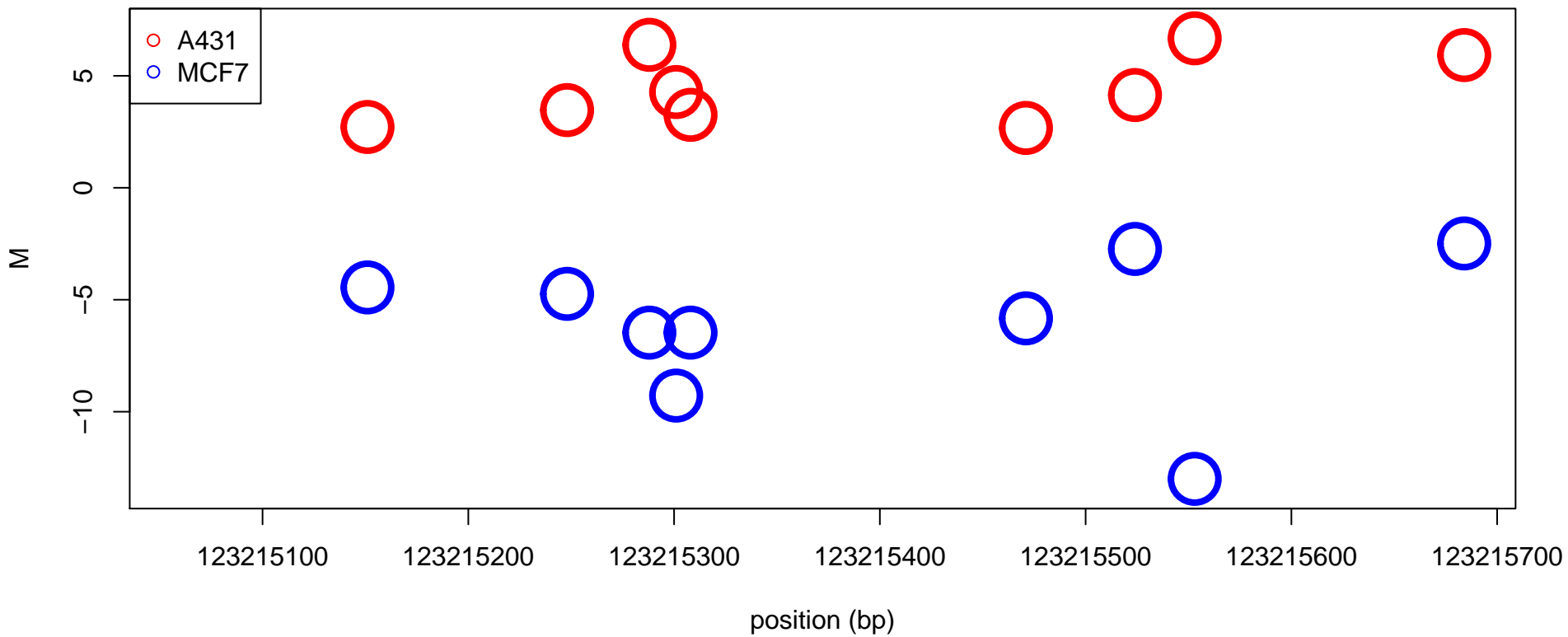

RegionID: 10086, chr12:123215151-123215684-Beta\_values

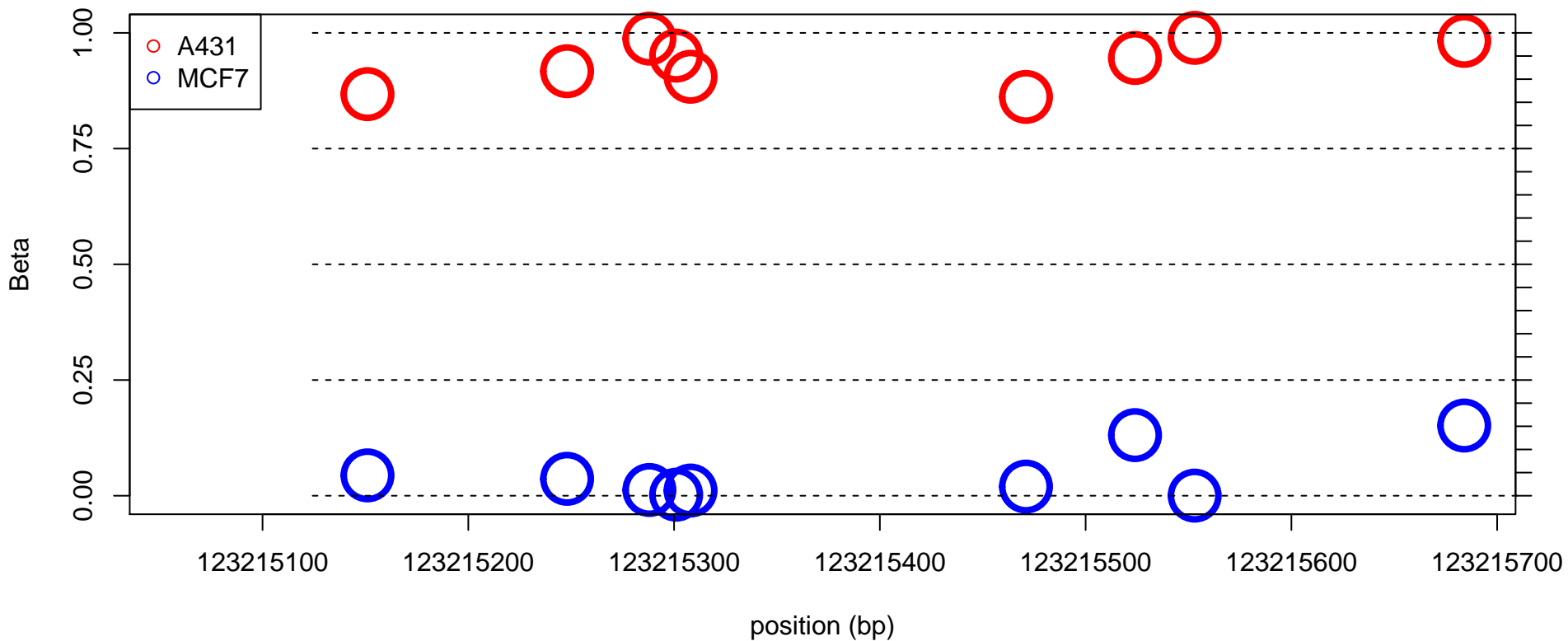

Chromosome 12

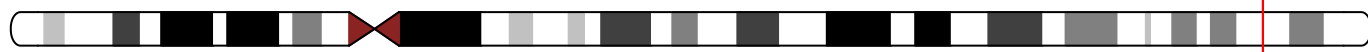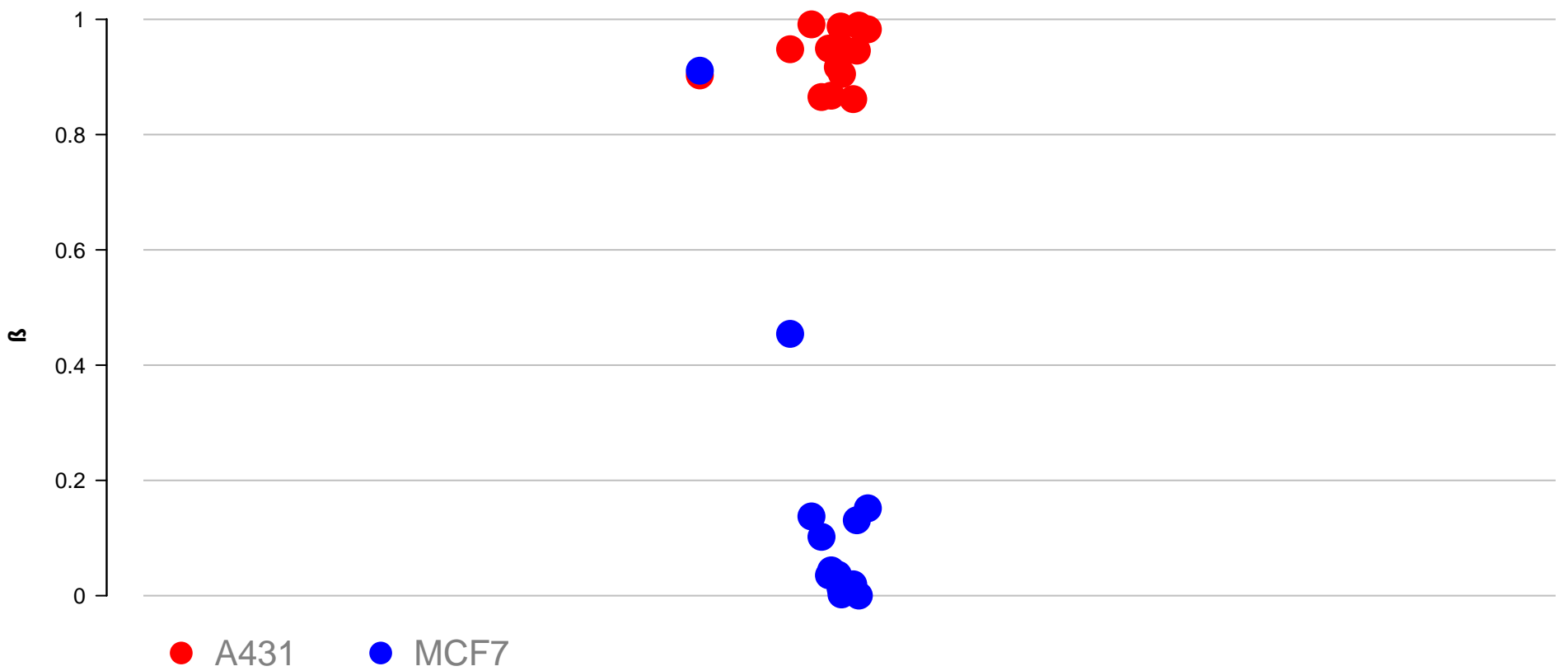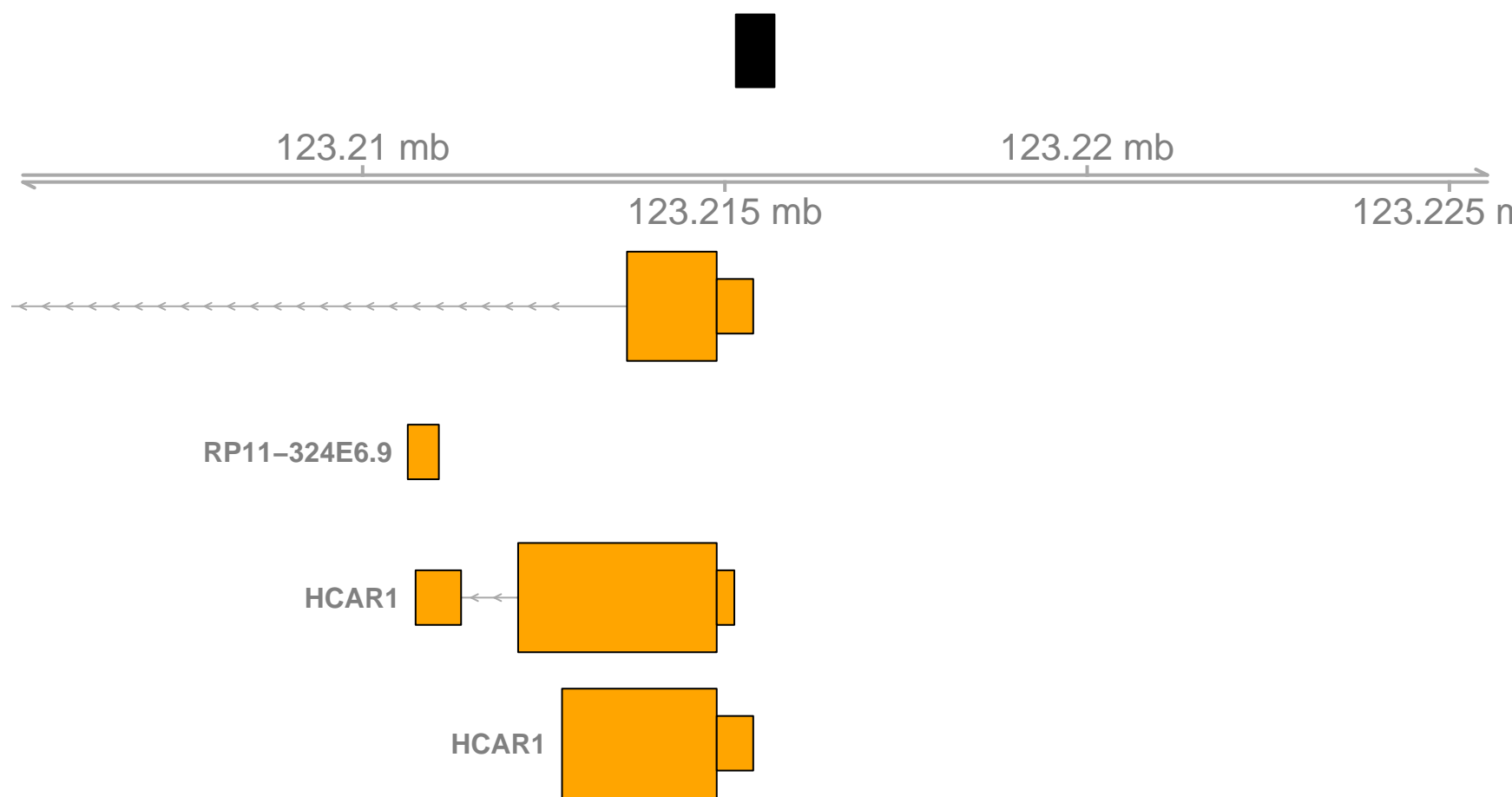

Supplement: Additional file 2 — DMRforPairs output for the comparison of A431-MCF7 and NA17018-NA17105. Please start from the HTML files in each folder. Available via the BMC Bioinformatics website. [file 1471-2105-15-141-S2.zip › 1394847754114233_MOESM2_ESM/A431_MCF7/figures/10086.pdf]

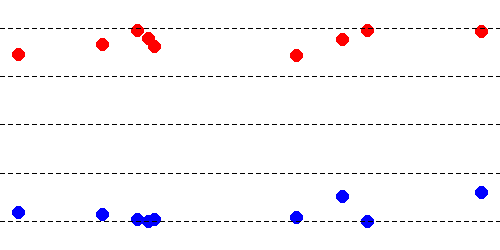

Supplement: Additional file 2 — DMRforPairs output for the comparison of A431-MCF7 and NA17018-NA17105. Please start from the HTML files in each folder. Available via the BMC Bioinformatics website. [file 1471-2105-15-141-S2.zip › 1394847754114233_MOESM2_ESM/A431_MCF7/figures/10086.png]

RegionID: 10089, chr12:123319216–123319366–M\_values

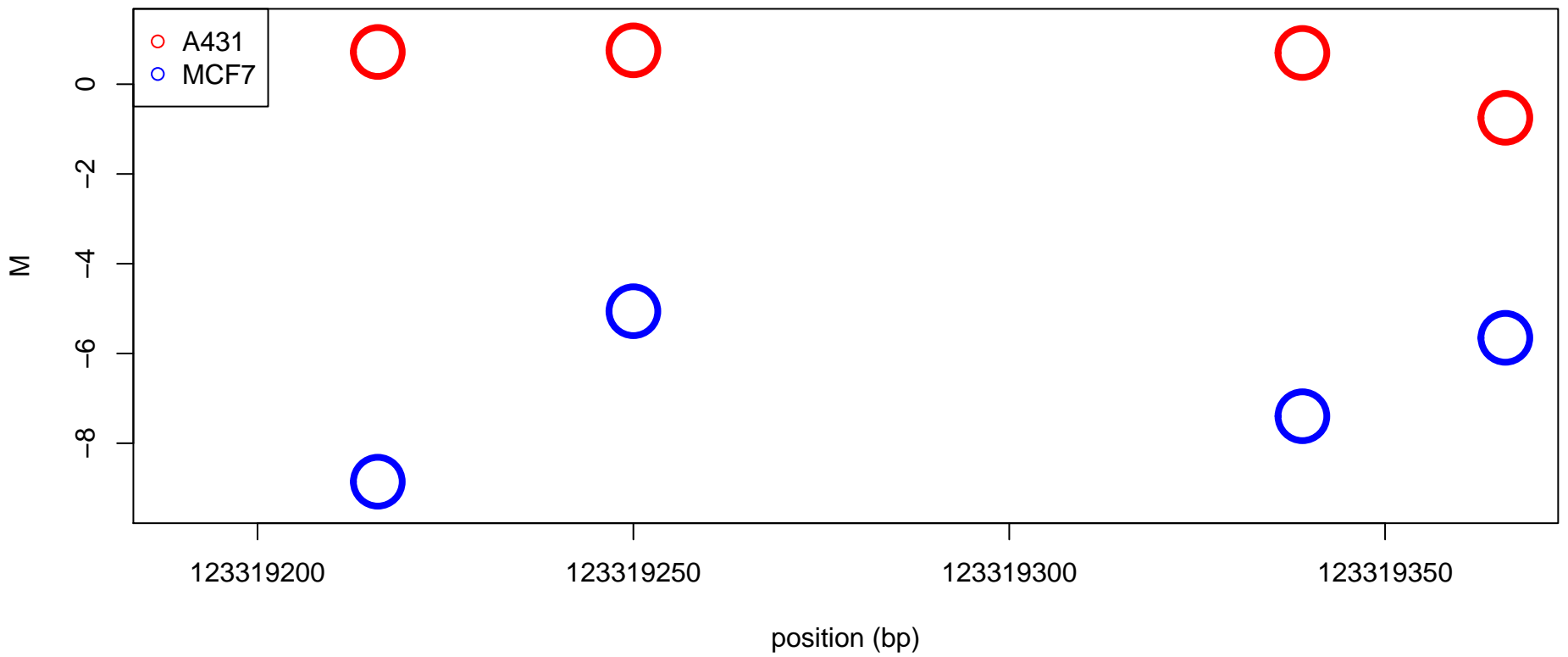

RegionID: 10089, chr12:123319216–123319366–Beta\_values

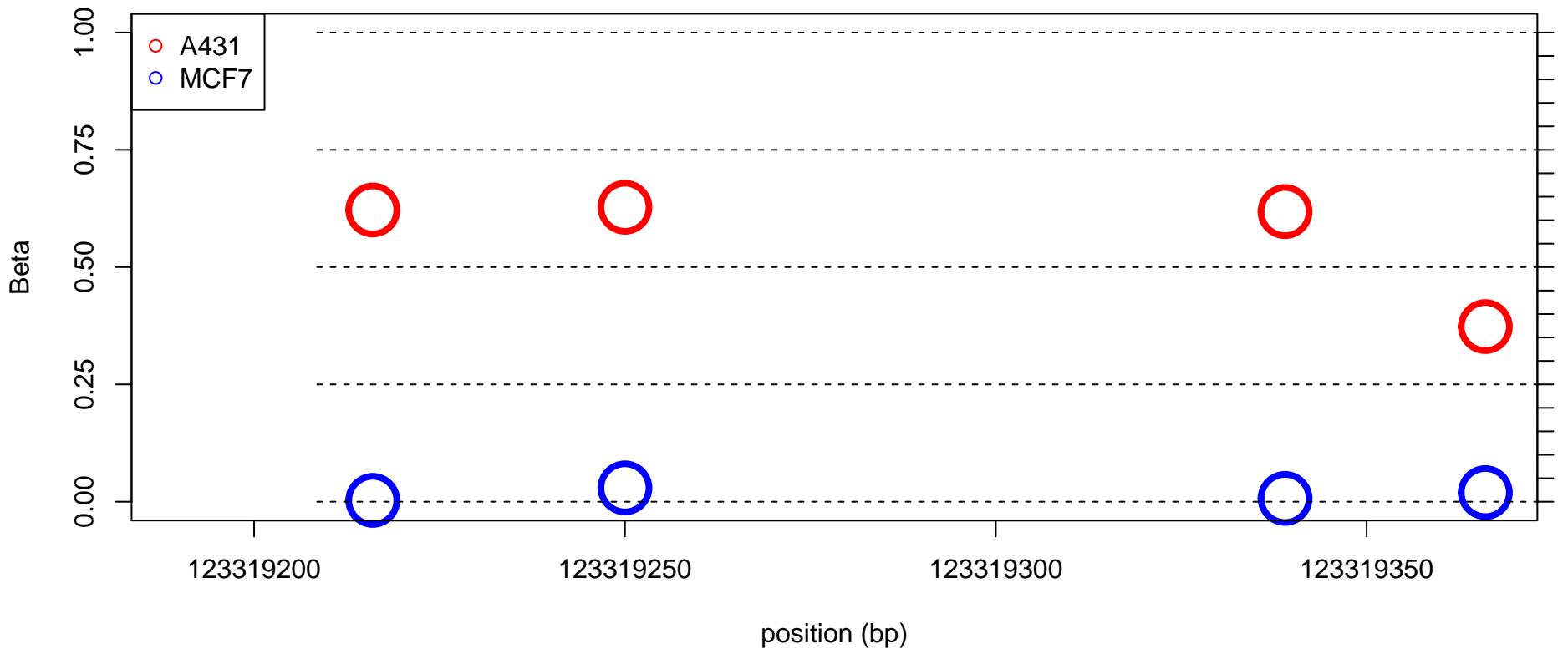

Supplement: Additional file 2 — DMRforPairs output for the comparison of A431-MCF7 and NA17018-NA17105. Please start from the HTML files in each folder. Available via the BMC Bioinformatics website. [file 1471-2105-15-141-S2.zip › 1394847754114233_MOESM2_ESM/A431_MCF7/figures/10089.pdf]

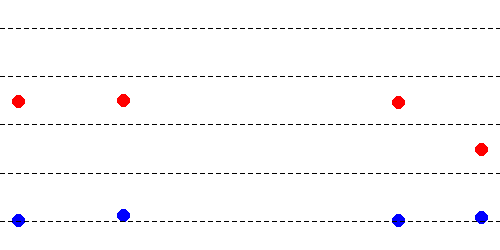

Supplement: Additional file 2 — DMRforPairs output for the comparison of A431-MCF7 and NA17018-NA17105. Please start from the HTML files in each folder. Available via the BMC Bioinformatics website. [file 1471-2105-15-141-S2.zip › 1394847754114233_MOESM2_ESM/A431_MCF7/figures/10089.png]

RegionID: 1009, chr1:215256254–215256978–M\_values

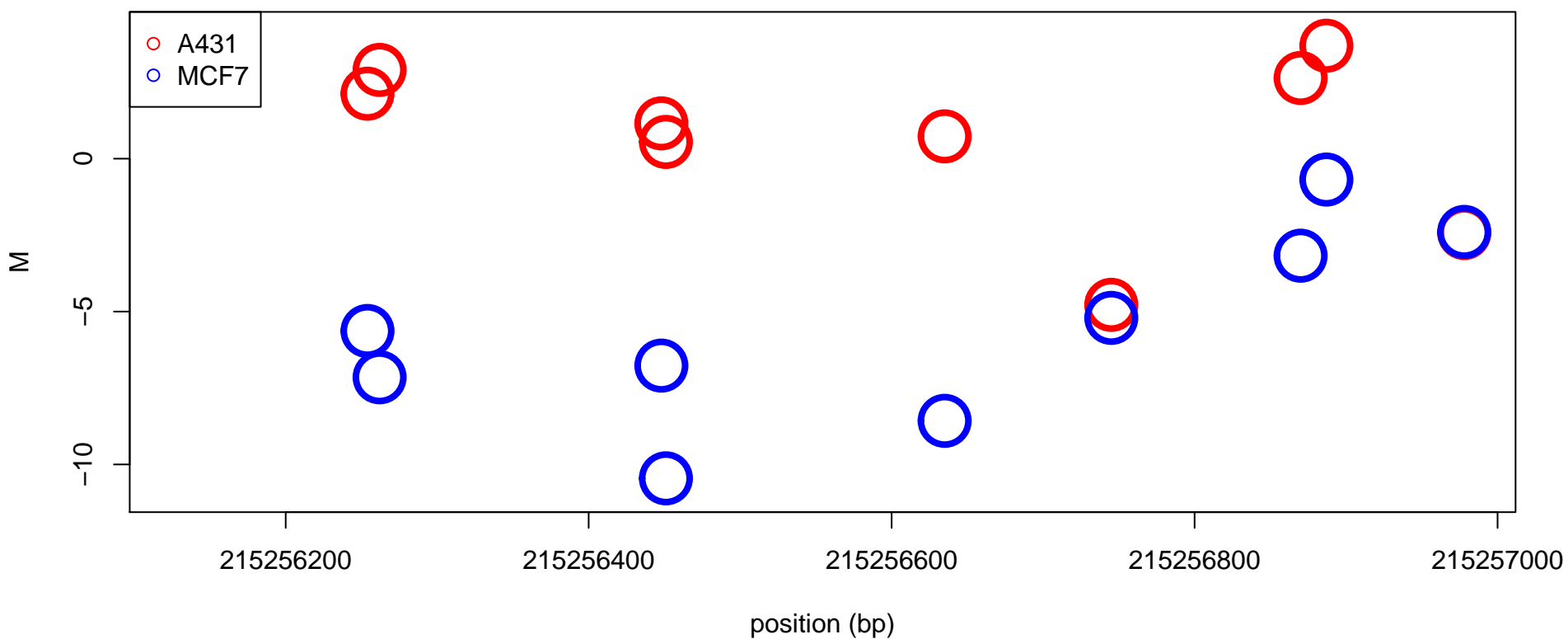

RegionID: 1009, chr1:215256254–215256978–Beta\_values

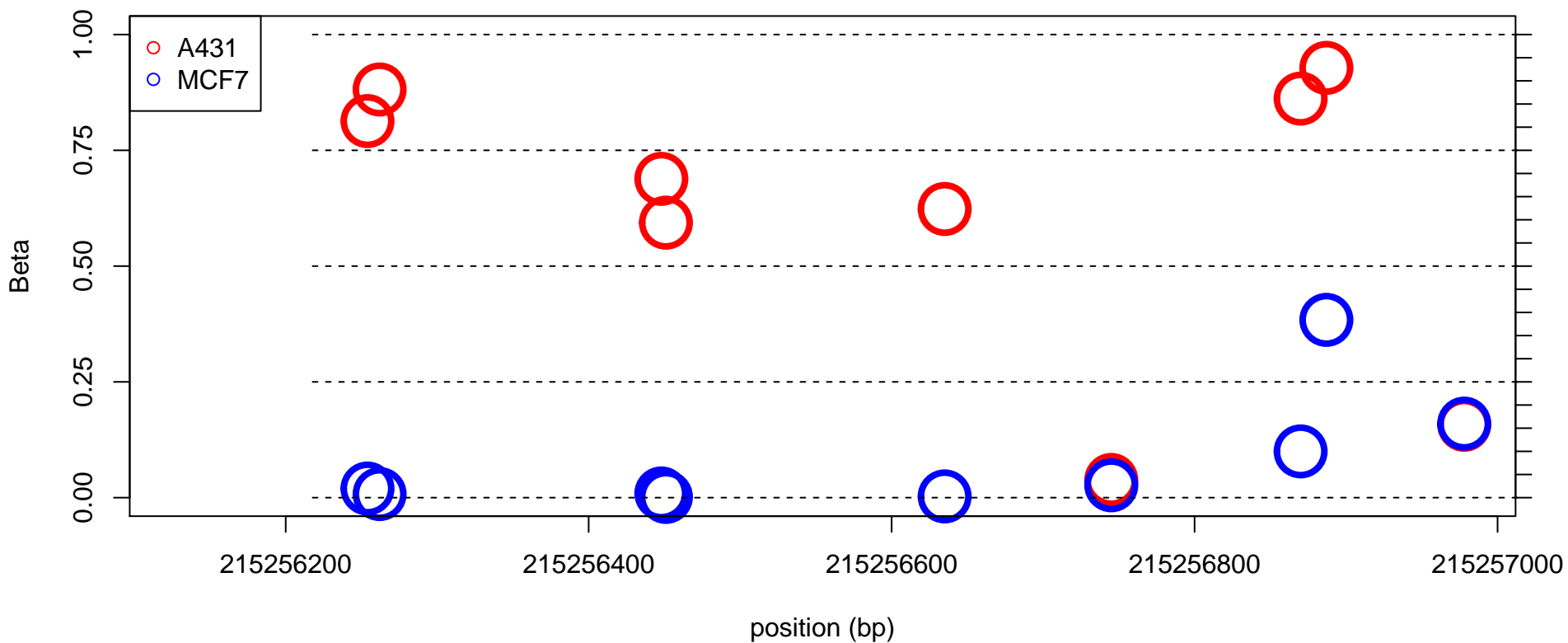

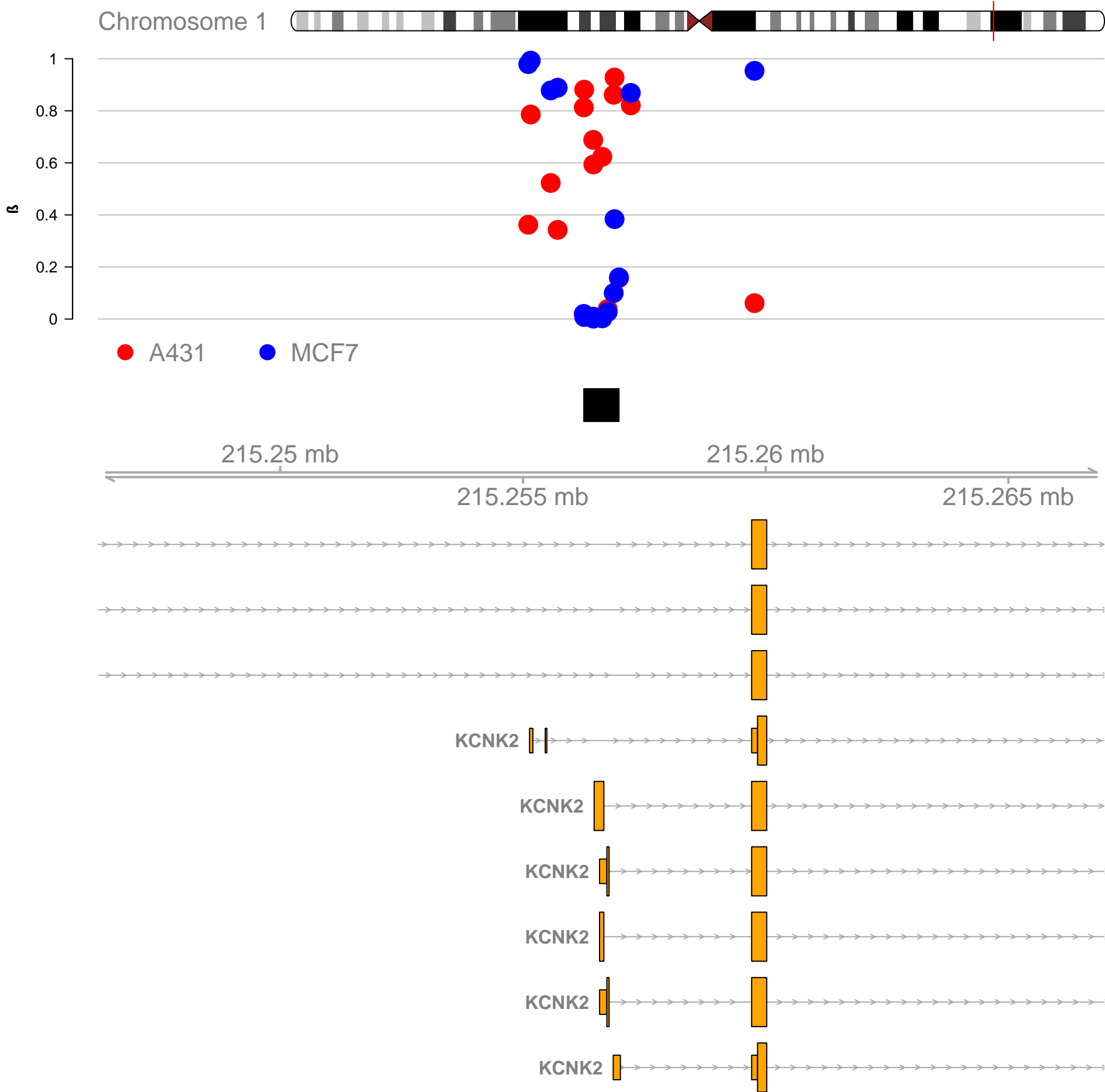

Supplement: Additional file 2 — DMRforPairs output for the comparison of A431-MCF7 and NA17018-NA17105. Please start from the HTML files in each folder. Available via the BMC Bioinformatics website. [file 1471-2105-15-141-S2.zip › 1394847754114233_MOESM2_ESM/A431_MCF7/figures/1009.pdf]

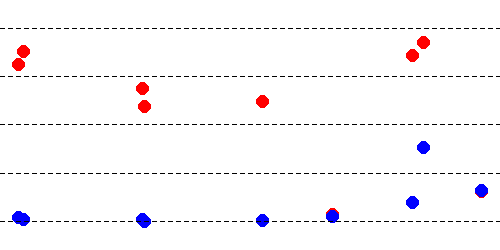

Supplement: Additional file 2 — DMRforPairs output for the comparison of A431-MCF7 and NA17018-NA17105. Please start from the HTML files in each folder. Available via the BMC Bioinformatics website. [file 1471-2105-15-141-S2.zip › 1394847754114233_MOESM2_ESM/A431_MCF7/figures/1009.png]

RegionID: 10094, chr12:123459803–123459832–M\_values

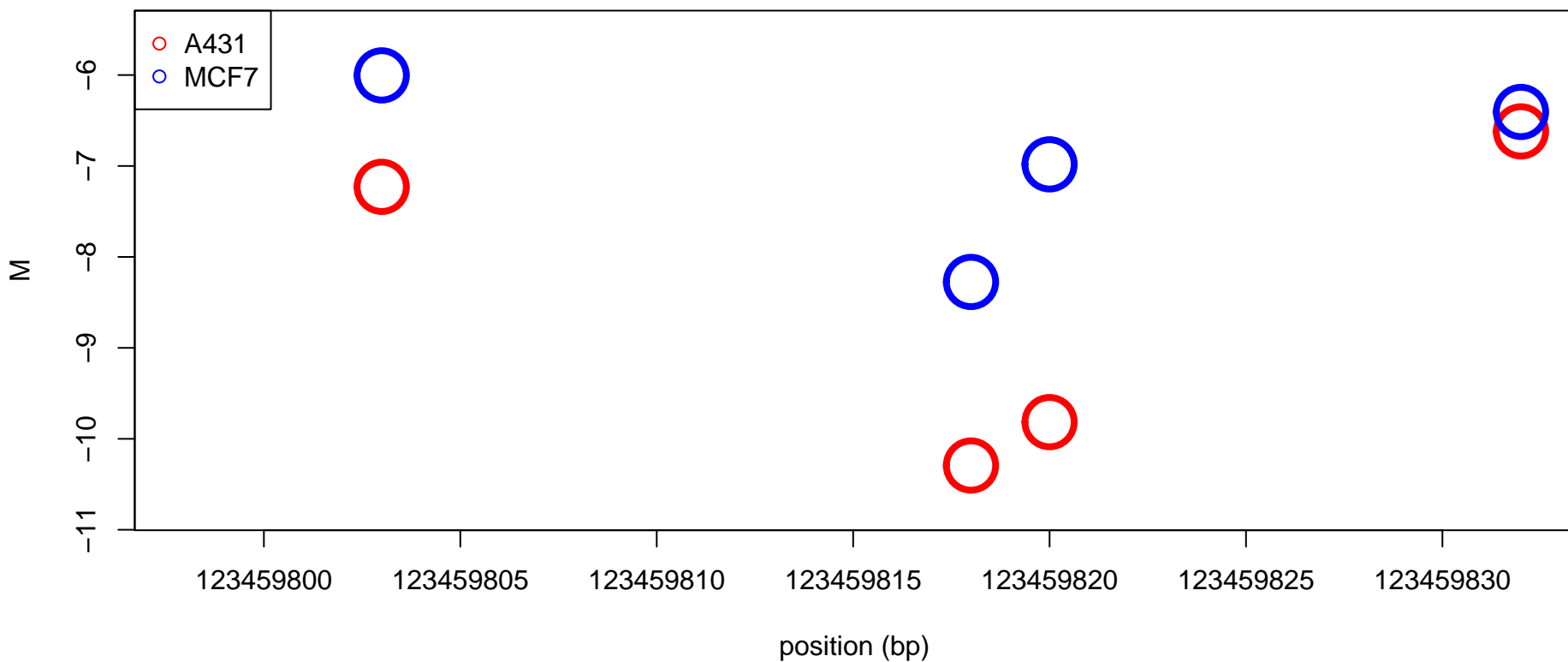

RegionID: 10094, chr12:123459803–123459832–Beta\_values

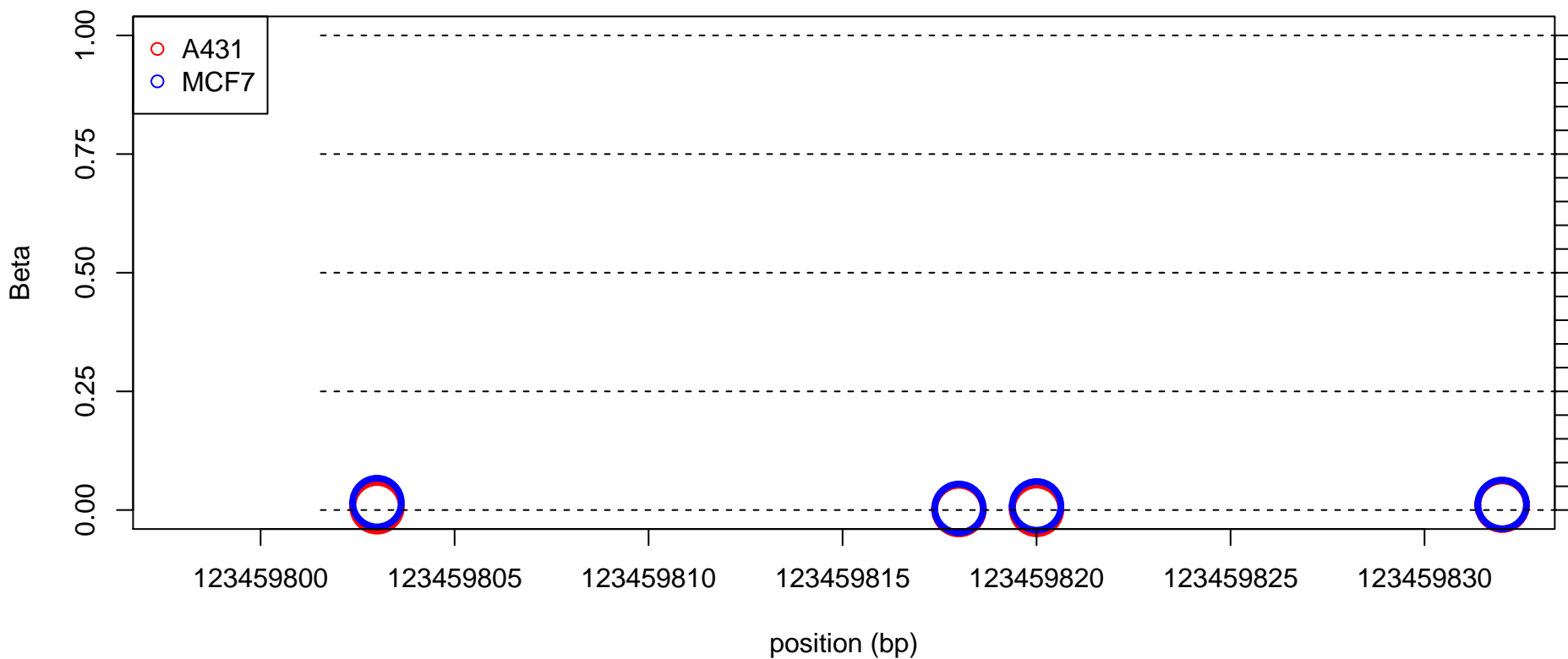

Supplement: Additional file 2 — DMRforPairs output for the comparison of A431-MCF7 and NA17018-NA17105. Please start from the HTML files in each folder. Available via the BMC Bioinformatics website. [file 1471-2105-15-141-S2.zip › 1394847754114233_MOESM2_ESM/A431_MCF7/figures/10094.pdf]

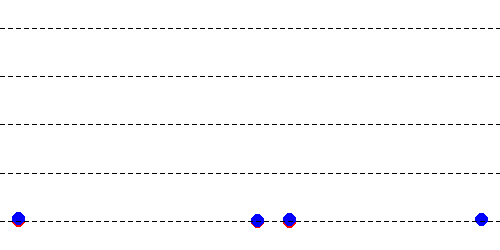

Supplement: Additional file 2 — DMRforPairs output for the comparison of A431-MCF7 and NA17018-NA17105. Please start from the HTML files in each folder. Available via the BMC Bioinformatics website. [file 1471-2105-15-141-S2.zip › 1394847754114233_MOESM2_ESM/A431_MCF7/figures/10094.png]

RegionID: 10095, chr12:123464511–123464823–M\_values

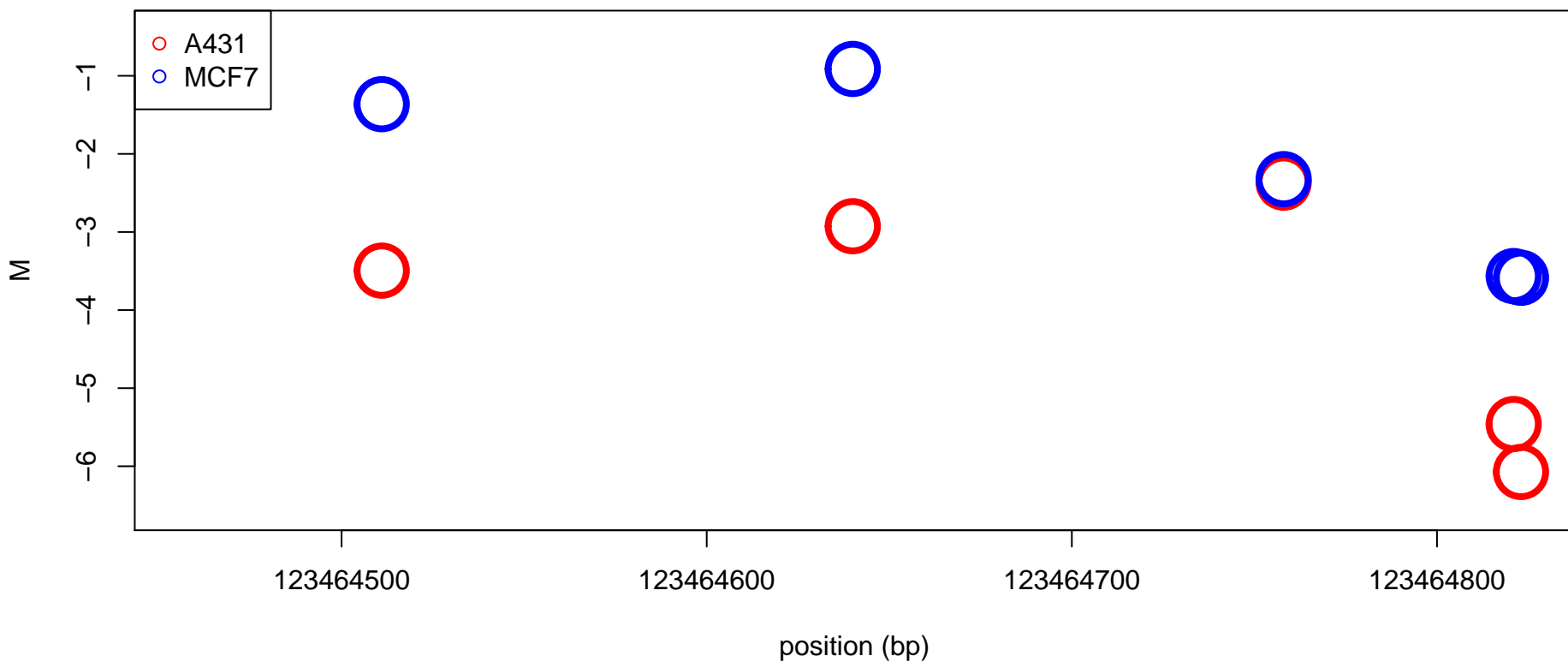

RegionID: 10095, chr12:123464511–123464823–Beta\_values

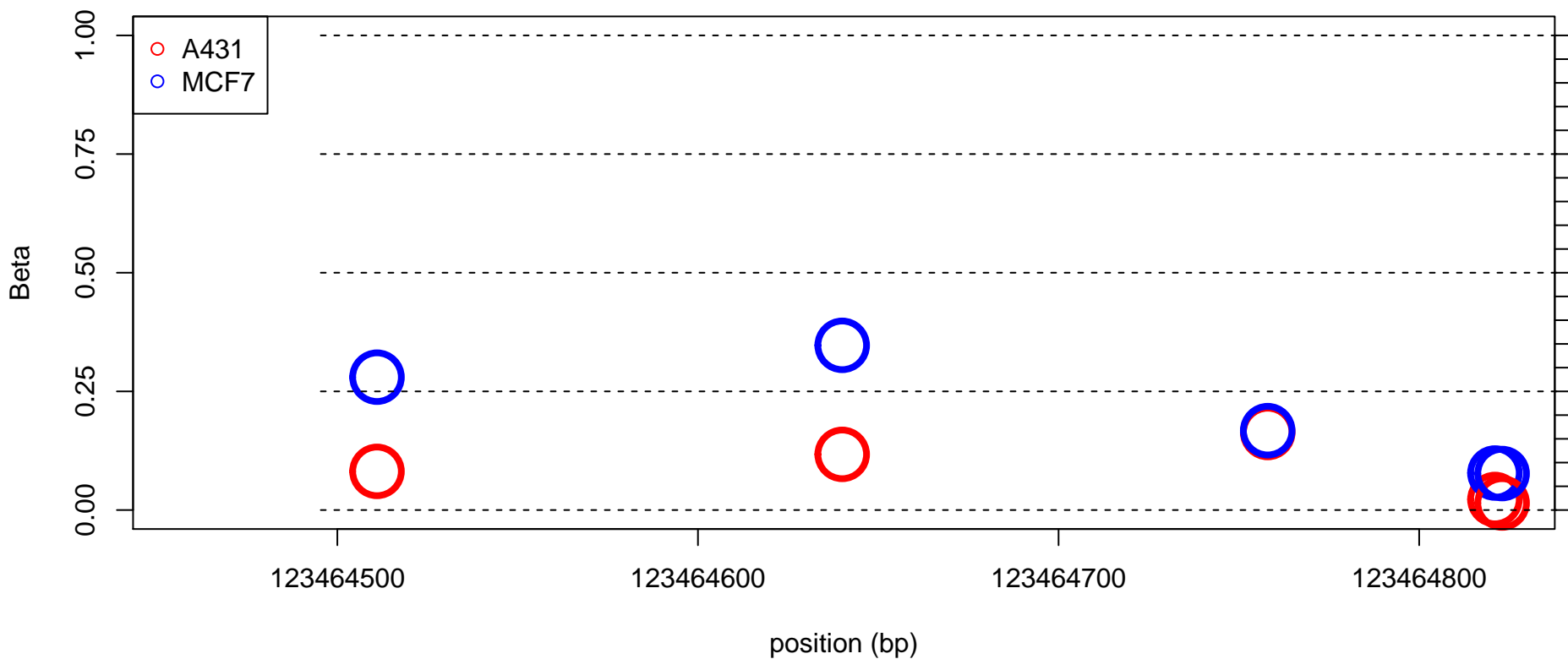

Supplement: Additional file 2 — DMRforPairs output for the comparison of A431-MCF7 and NA17018-NA17105. Please start from the HTML files in each folder. Available via the BMC Bioinformatics website. [file 1471-2105-15-141-S2.zip › 1394847754114233_MOESM2_ESM/A431_MCF7/figures/10095.pdf]

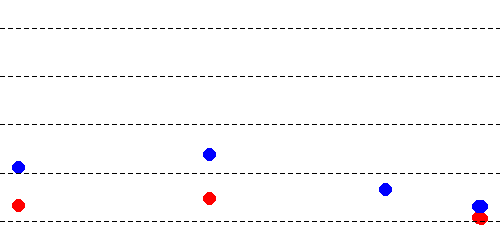

Supplement: Additional file 2 — DMRforPairs output for the comparison of A431-MCF7 and NA17018-NA17105. Please start from the HTML files in each folder. Available via the BMC Bioinformatics website. [file 1471-2105-15-141-S2.zip › 1394847754114233_MOESM2_ESM/A431_MCF7/figures/10095.png]

RegionID: 101, chr1:3044741-3045166-M\_values

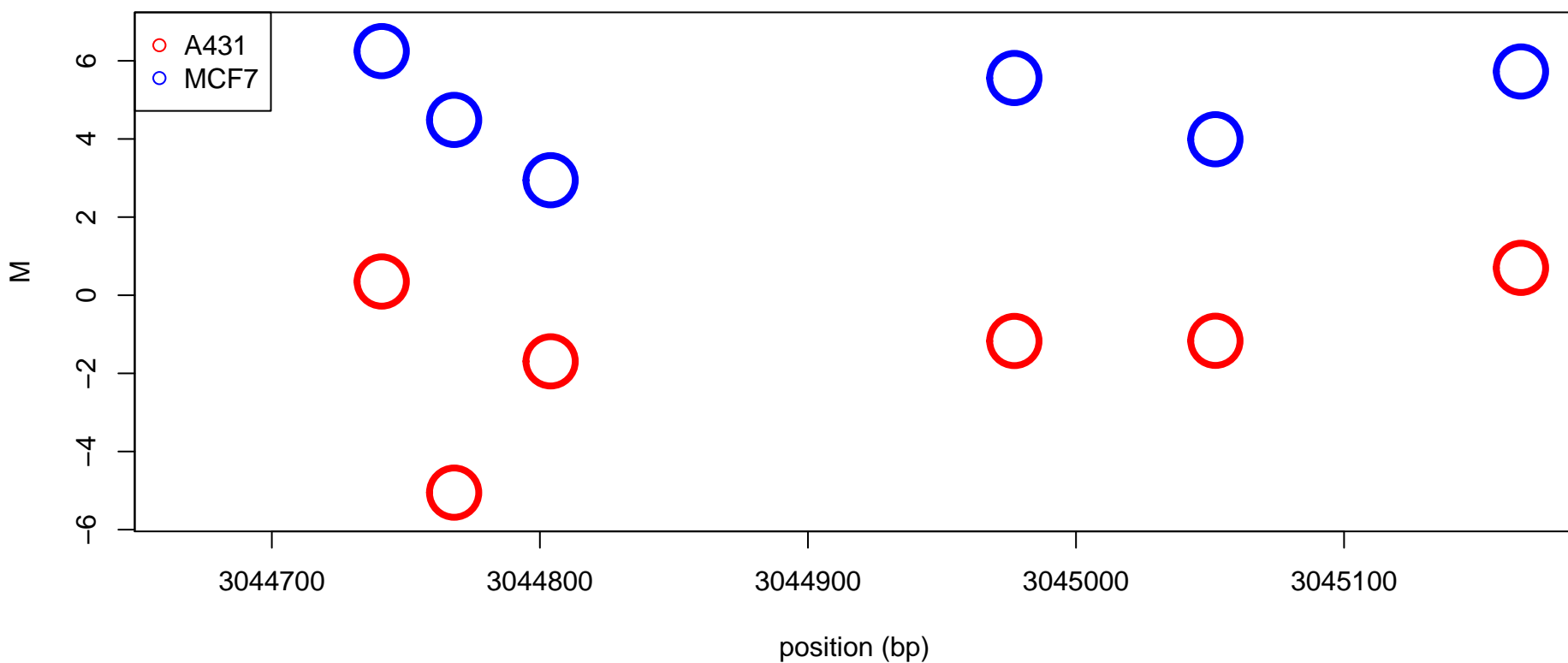

RegionID: 101, chr1:3044741-3045166-Beta\_values

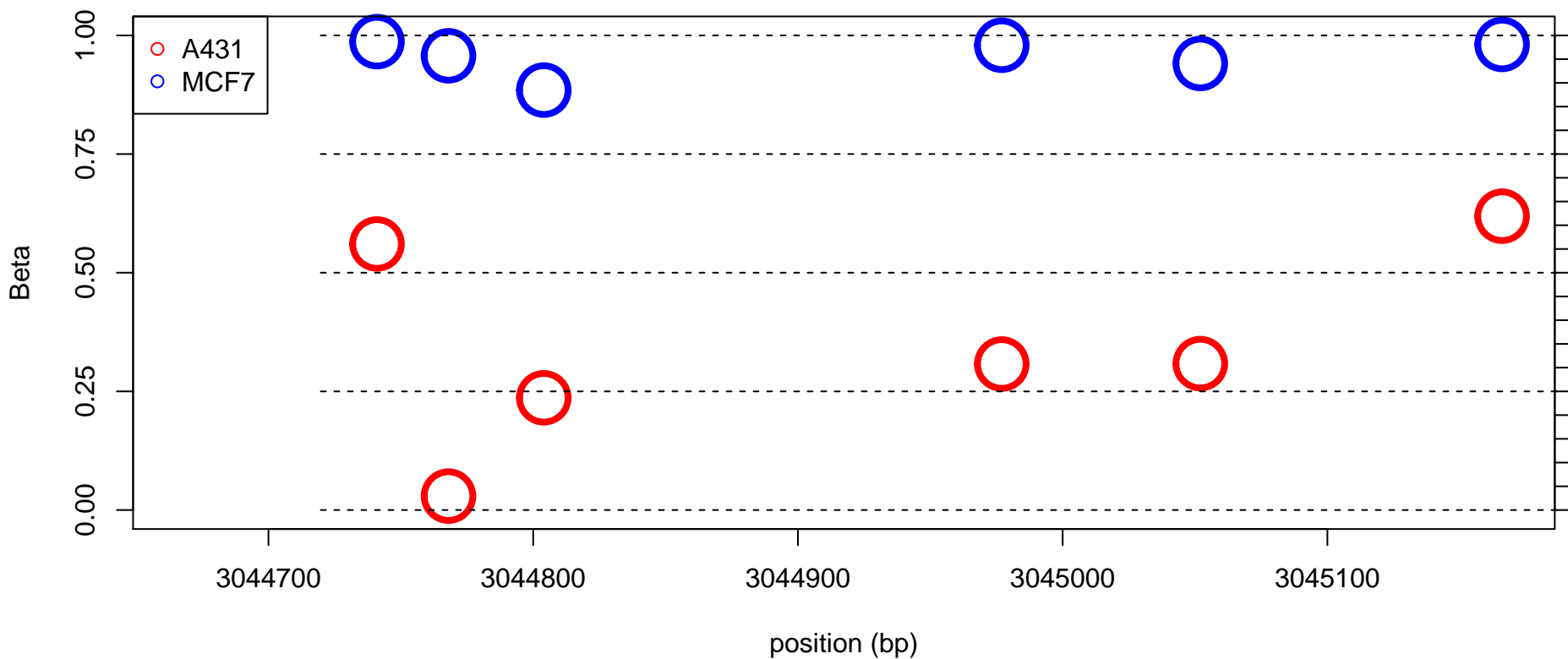

Chromosome 1

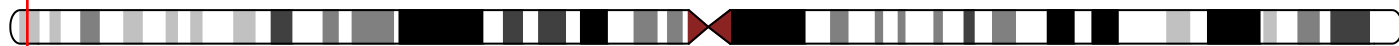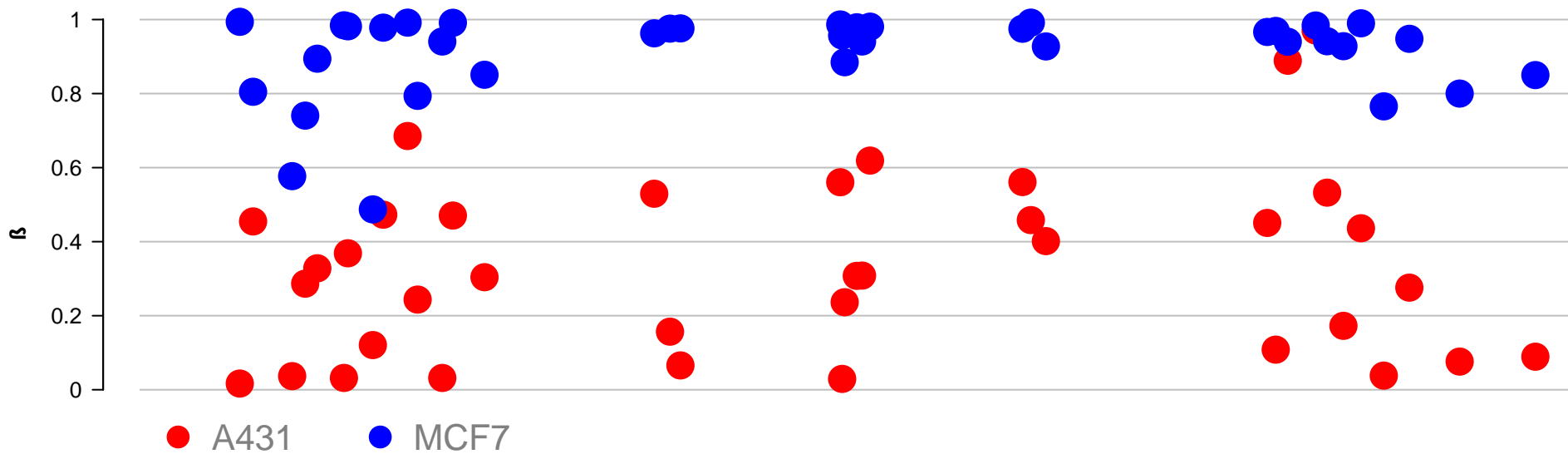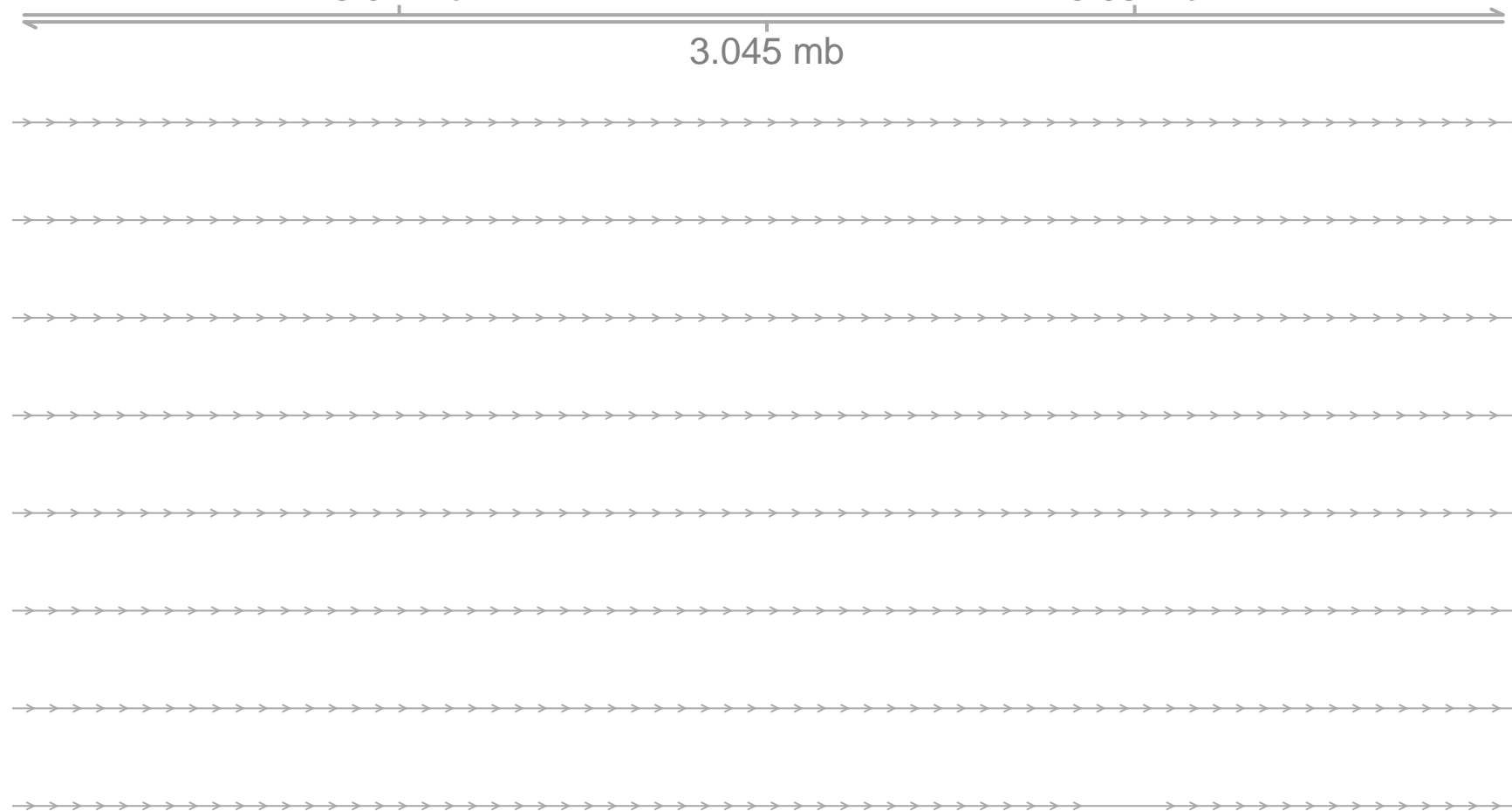

RP1-163G9.2

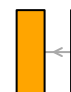

Supplement: Additional file 2 — DMRforPairs output for the comparison of A431-MCF7 and NA17018-NA17105. Please start from the HTML files in each folder. Available via the BMC Bioinformatics website. [file 1471-2105-15-141-S2.zip › 1394847754114233_MOESM2_ESM/A431_MCF7/figures/101.pdf]

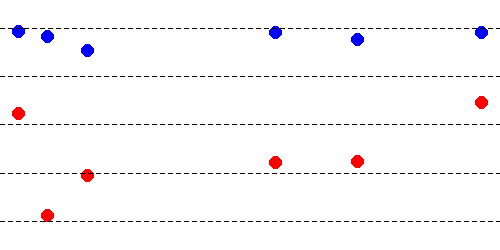

Supplement: Additional file 2 — DMRforPairs output for the comparison of A431-MCF7 and NA17018-NA17105. Please start from the HTML files in each folder. Available via the BMC Bioinformatics website. [file 1471-2105-15-141-S2.zip › 1394847754114233_MOESM2_ESM/A431_MCF7/figures/101.png]

RegionID: 10105, chr12:124155451-124155620-M\_values

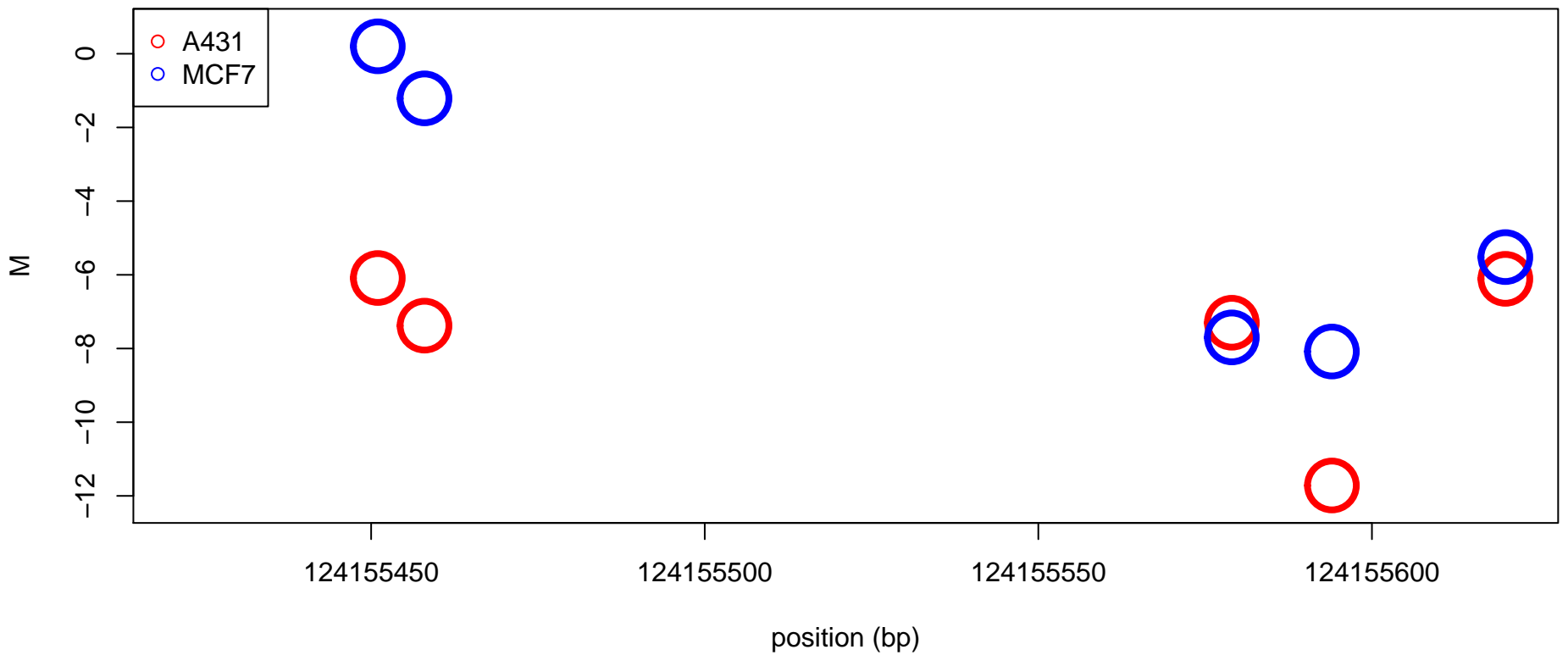

RegionID: 10105, chr12:124155451-124155620-Beta\_values

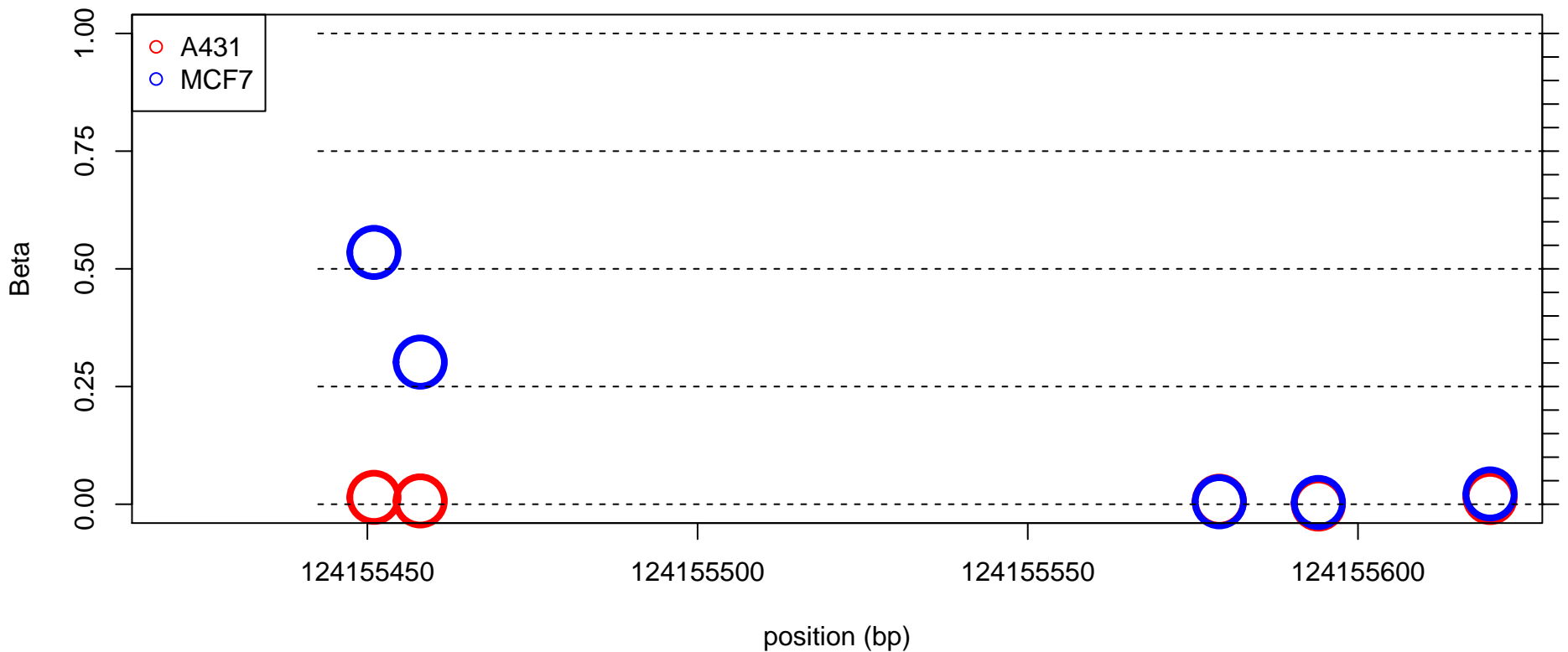

Supplement: Additional file 2 — DMRforPairs output for the comparison of A431-MCF7 and NA17018-NA17105. Please start from the HTML files in each folder. Available via the BMC Bioinformatics website. [file 1471-2105-15-141-S2.zip › 1394847754114233_MOESM2_ESM/A431_MCF7/figures/10105.pdf]

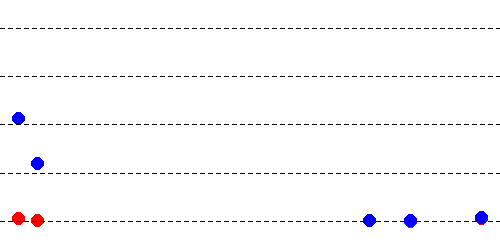

Supplement: Additional file 2 — DMRforPairs output for the comparison of A431-MCF7 and NA17018-NA17105. Please start from the HTML files in each folder. Available via the BMC Bioinformatics website. [file 1471-2105-15-141-S2.zip › 1394847754114233_MOESM2_ESM/A431_MCF7/figures/10105.png]

RegionID: 10107, chr12:124246391–124246525–M\_values

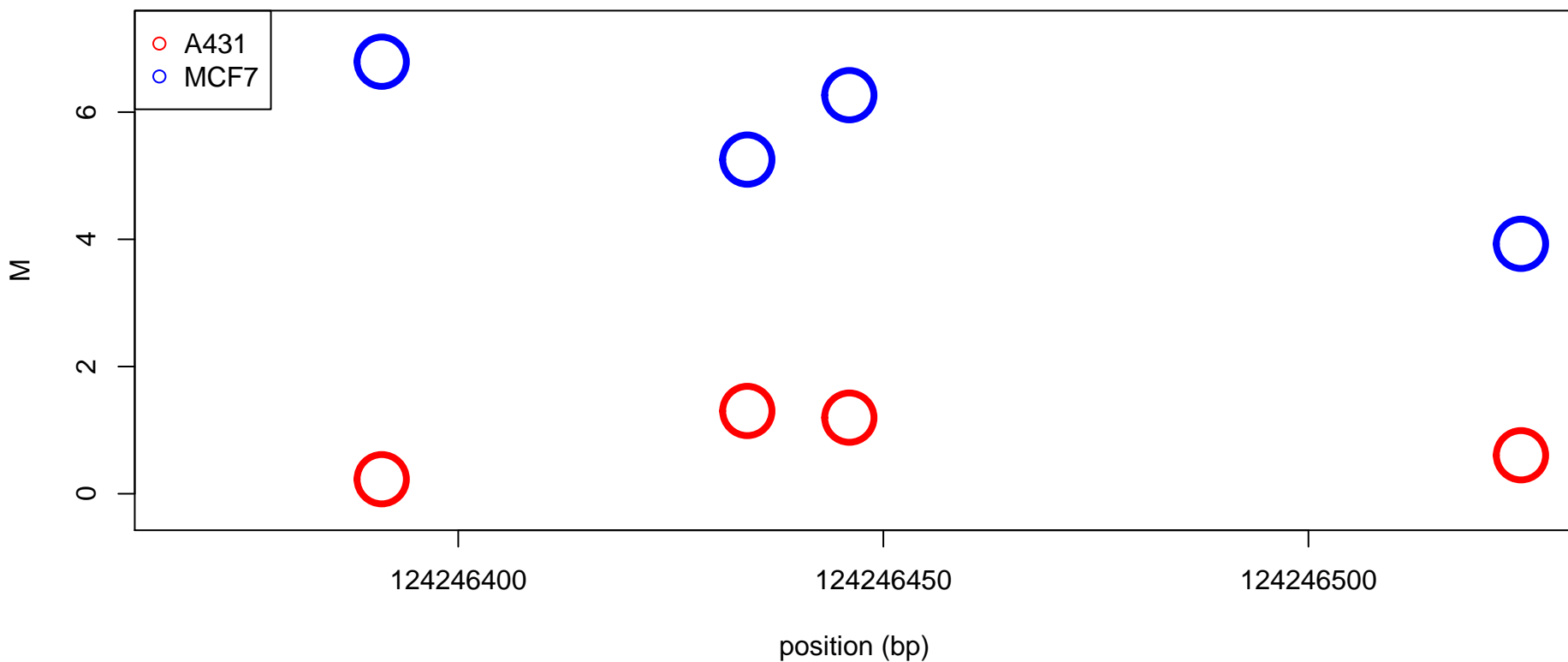

RegionID: 10107, chr12:124246391–124246525–Beta\_values

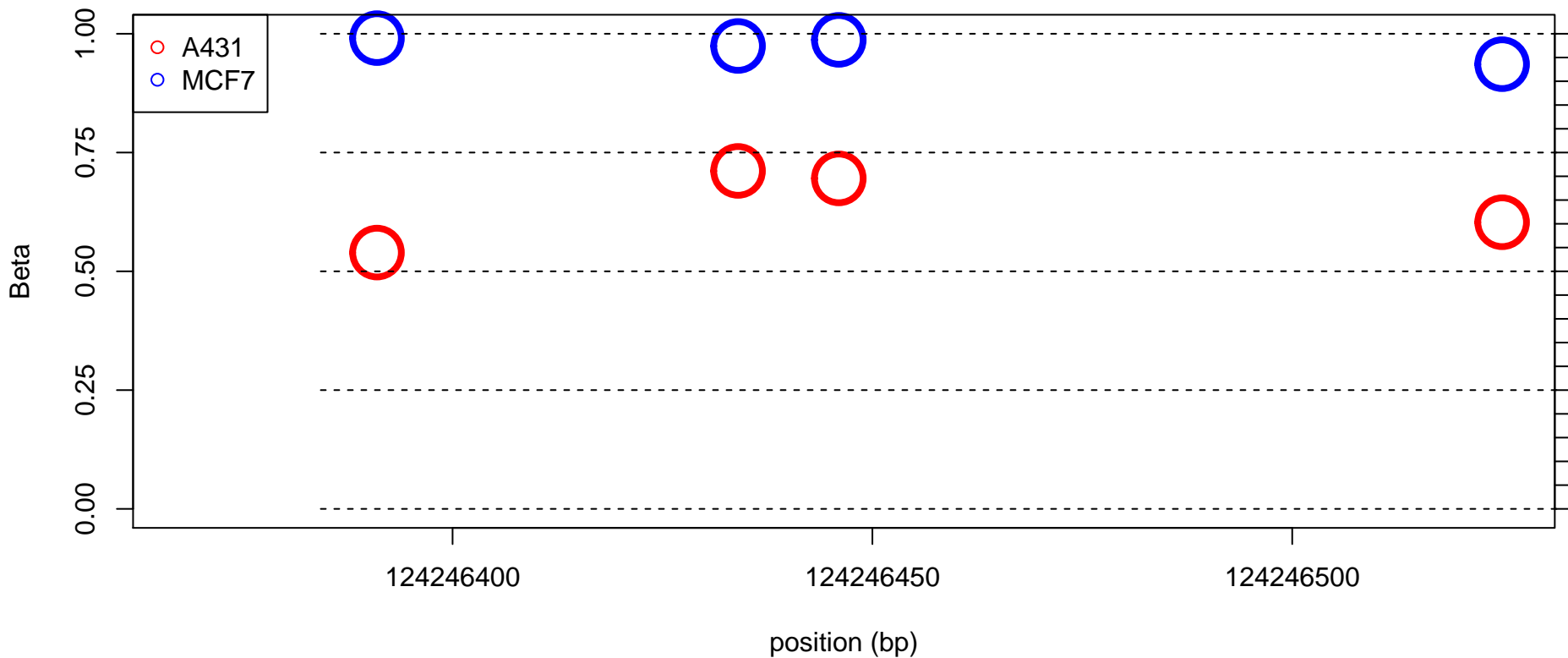

Supplement: Additional file 2 — DMRforPairs output for the comparison of A431-MCF7 and NA17018-NA17105. Please start from the HTML files in each folder. Available via the BMC Bioinformatics website. [file 1471-2105-15-141-S2.zip › 1394847754114233_MOESM2_ESM/A431_MCF7/figures/10107.pdf]

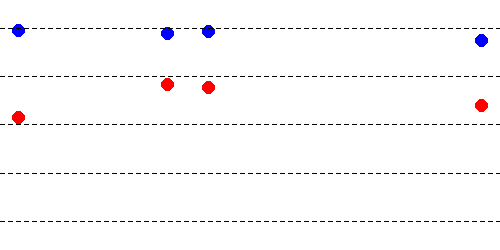

Supplement: Additional file 2 — DMRforPairs output for the comparison of A431-MCF7 and NA17018-NA17105. Please start from the HTML files in each folder. Available via the BMC Bioinformatics website. [file 1471-2105-15-141-S2.zip › 1394847754114233_MOESM2_ESM/A431_MCF7/figures/10107.png]

RegionID: 10110, chr12:125399699–125399964–M\_values

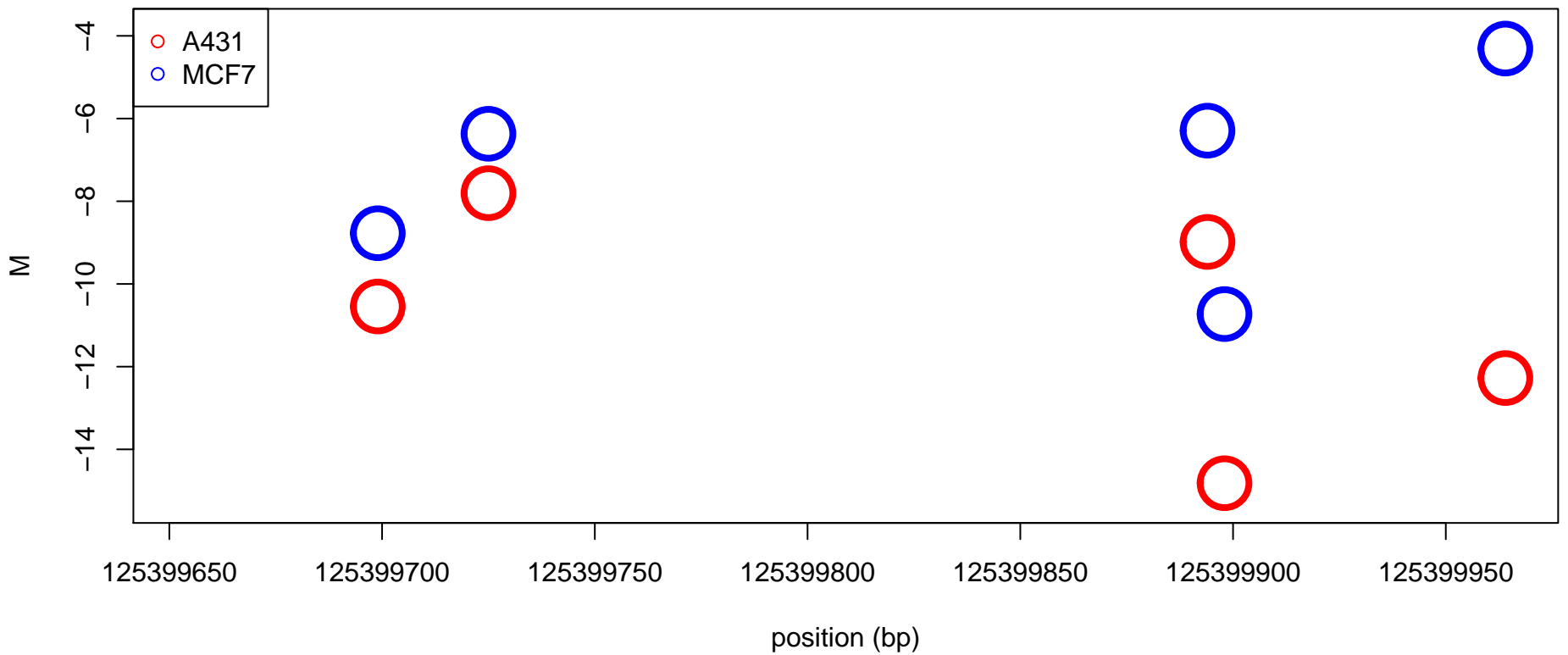

RegionID: 10110, chr12:125399699–125399964–Beta\_values

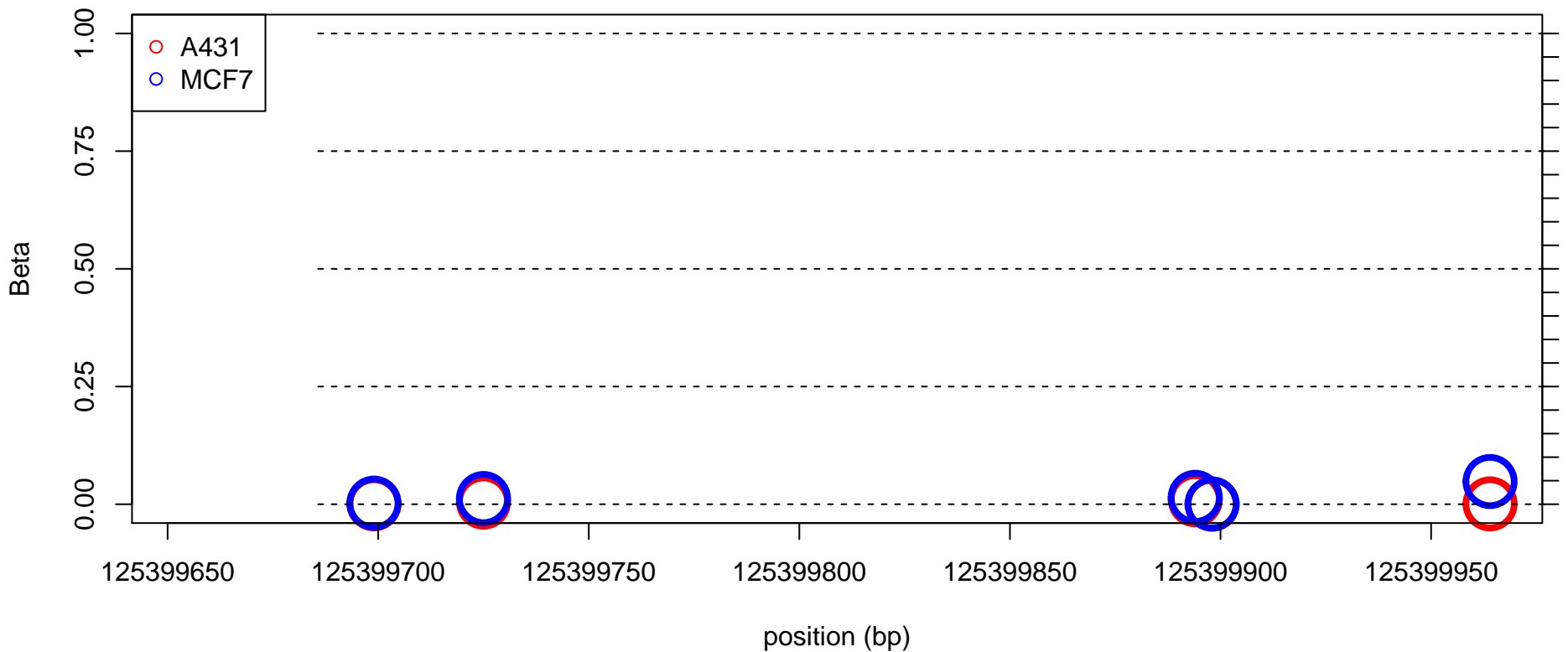

Supplement: Additional file 2 — DMRforPairs output for the comparison of A431-MCF7 and NA17018-NA17105. Please start from the HTML files in each folder. Available via the BMC Bioinformatics website. [file 1471-2105-15-141-S2.zip › 1394847754114233_MOESM2_ESM/A431_MCF7/figures/10110.pdf]

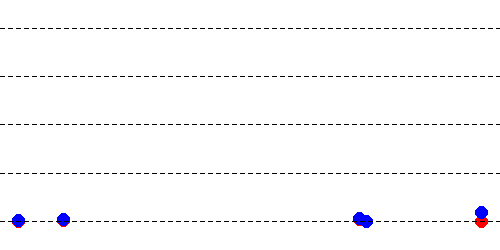

Supplement: Additional file 2 — DMRforPairs output for the comparison of A431-MCF7 and NA17018-NA17105. Please start from the HTML files in each folder. Available via the BMC Bioinformatics website. [file 1471-2105-15-141-S2.zip › 1394847754114233_MOESM2_ESM/A431_MCF7/figures/10110.png]

RegionID: 10115, chr12:129309117–129309376–M\_values

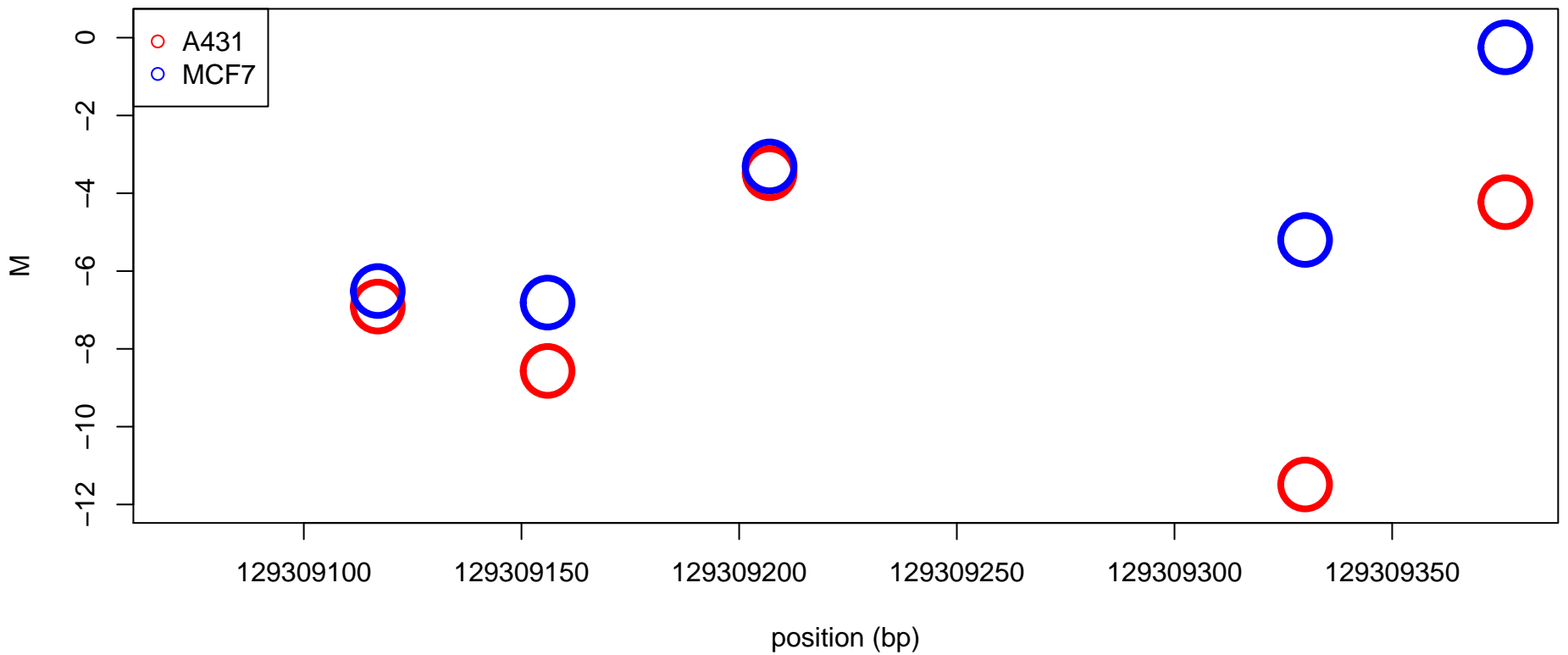

RegionID: 10115, chr12:129309117–129309376–Beta\_values

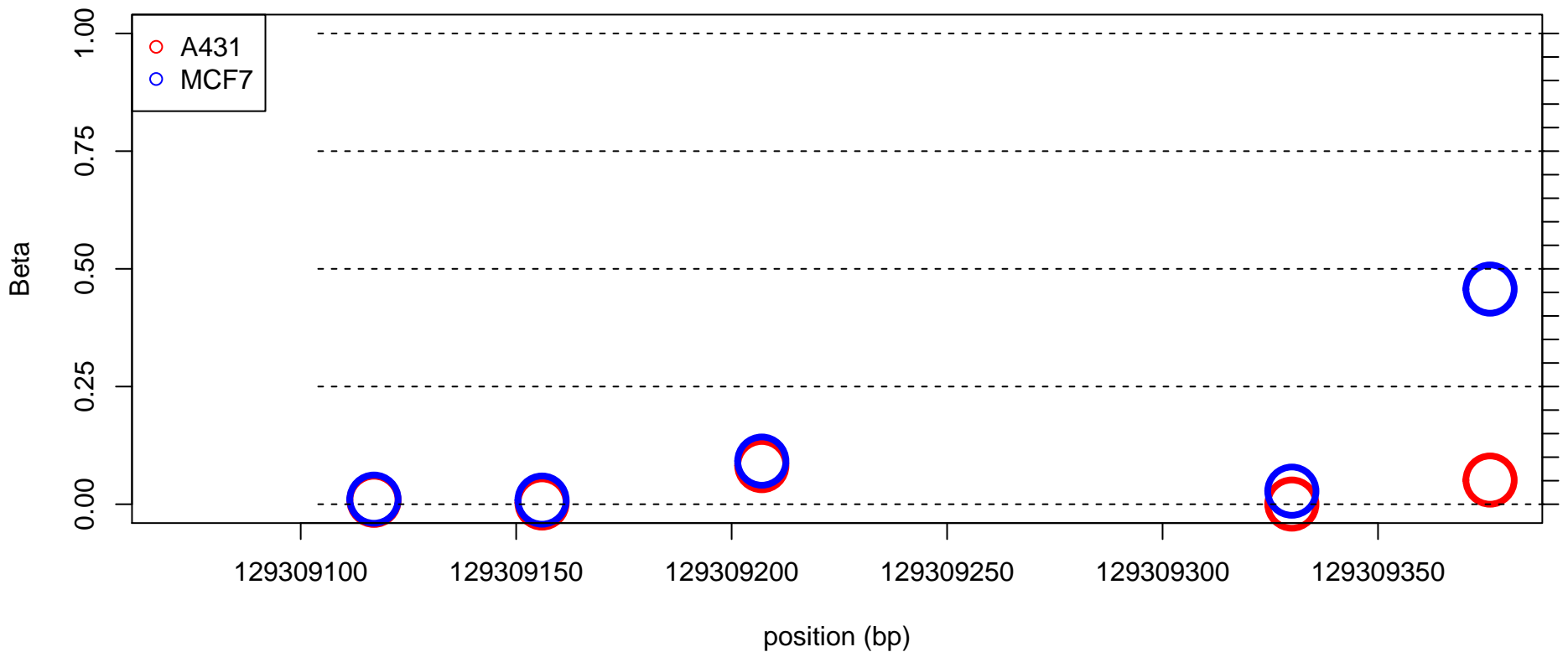

Supplement: Additional file 2 — DMRforPairs output for the comparison of A431-MCF7 and NA17018-NA17105. Please start from the HTML files in each folder. Available via the BMC Bioinformatics website. [file 1471-2105-15-141-S2.zip › 1394847754114233_MOESM2_ESM/A431_MCF7/figures/10115.pdf]

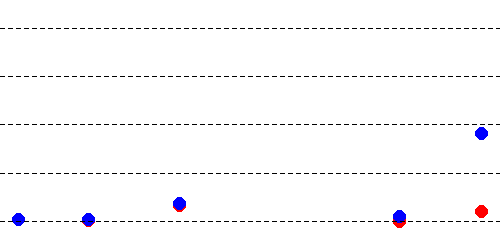

Supplement: Additional file 2 — DMRforPairs output for the comparison of A431-MCF7 and NA17018-NA17105. Please start from the HTML files in each folder. Available via the BMC Bioinformatics website. [file 1471-2105-15-141-S2.zip › 1394847754114233_MOESM2_ESM/A431_MCF7/figures/10115.png]

RegionID: 10116, chr12:129337674–129337910–M\_values

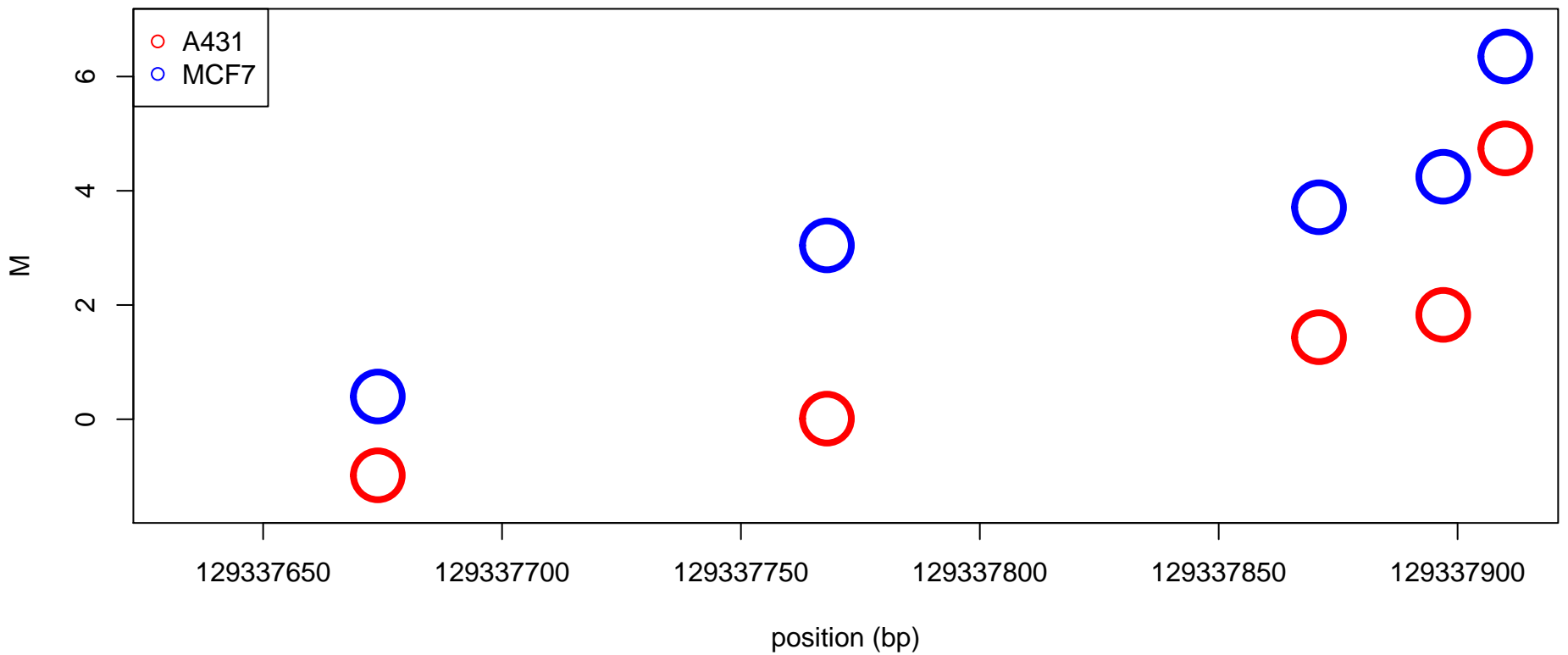

RegionID: 10116, chr12:129337674–129337910–Beta\_values

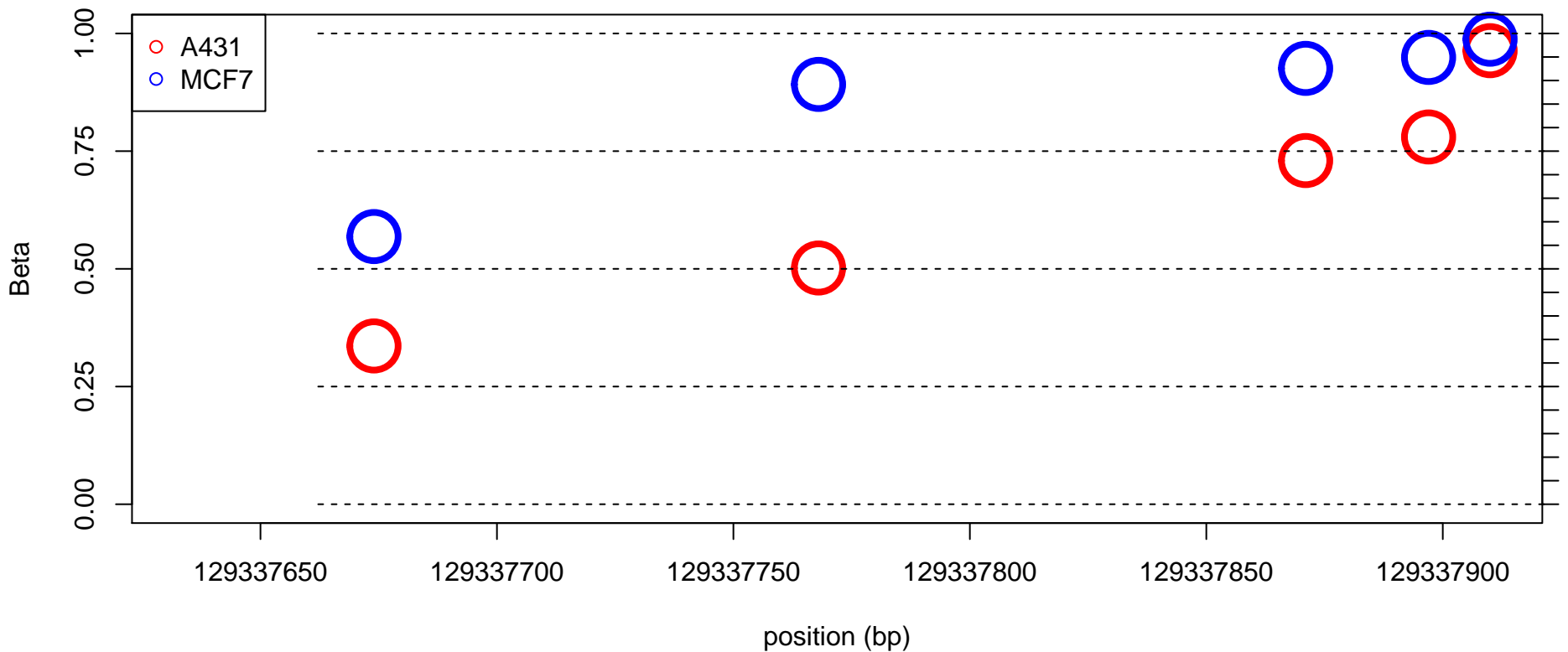

Supplement: Additional file 2 — DMRforPairs output for the comparison of A431-MCF7 and NA17018-NA17105. Please start from the HTML files in each folder. Available via the BMC Bioinformatics website. [file 1471-2105-15-141-S2.zip › 1394847754114233_MOESM2_ESM/A431_MCF7/figures/10116.pdf]

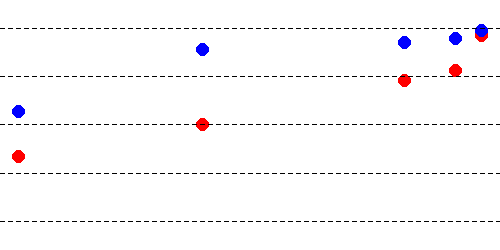

Supplement: Additional file 2 — DMRforPairs output for the comparison of A431-MCF7 and NA17018-NA17105. Please start from the HTML files in each folder. Available via the BMC Bioinformatics website. [file 1471-2105-15-141-S2.zip › 1394847754114233_MOESM2_ESM/A431_MCF7/figures/10116.png]

RegionID: 10117, chr12:130526916–130527212–M\_values

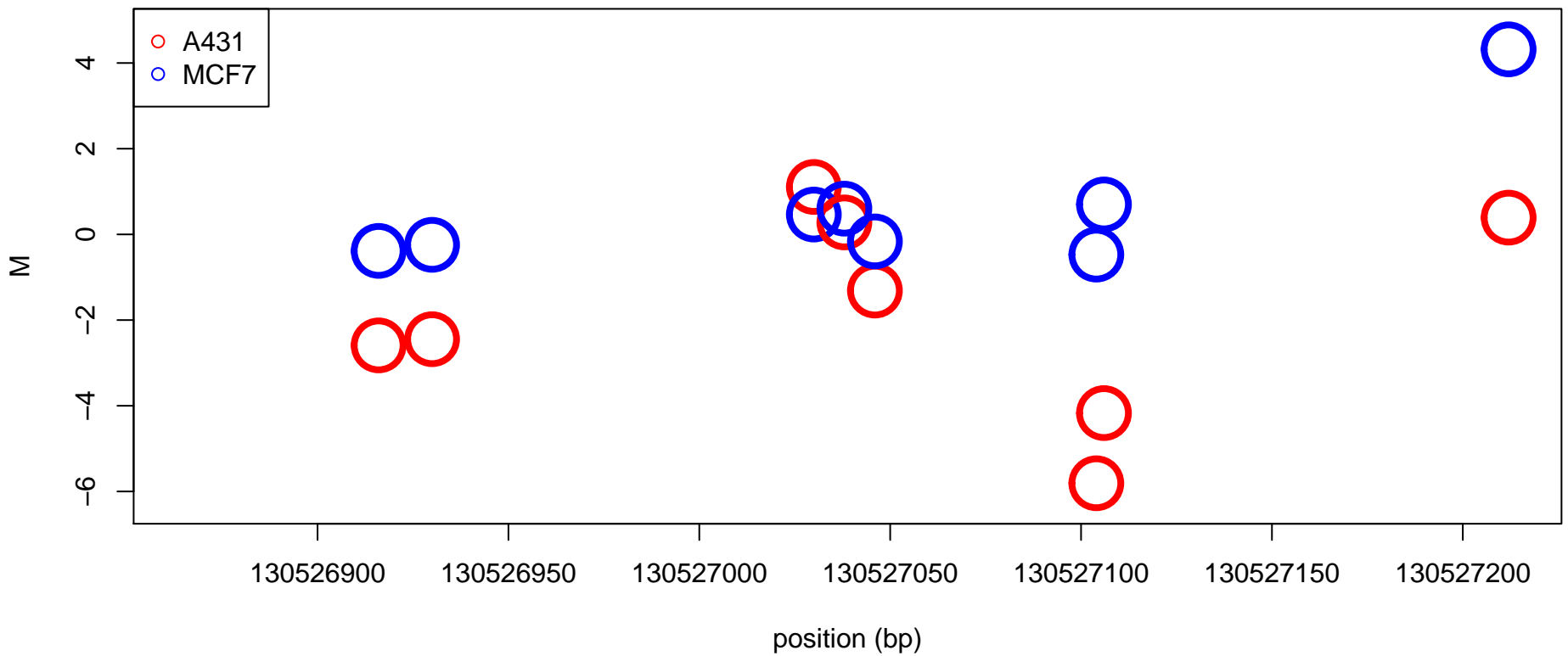

RegionID: 10117, chr12:130526916–130527212–Beta\_values

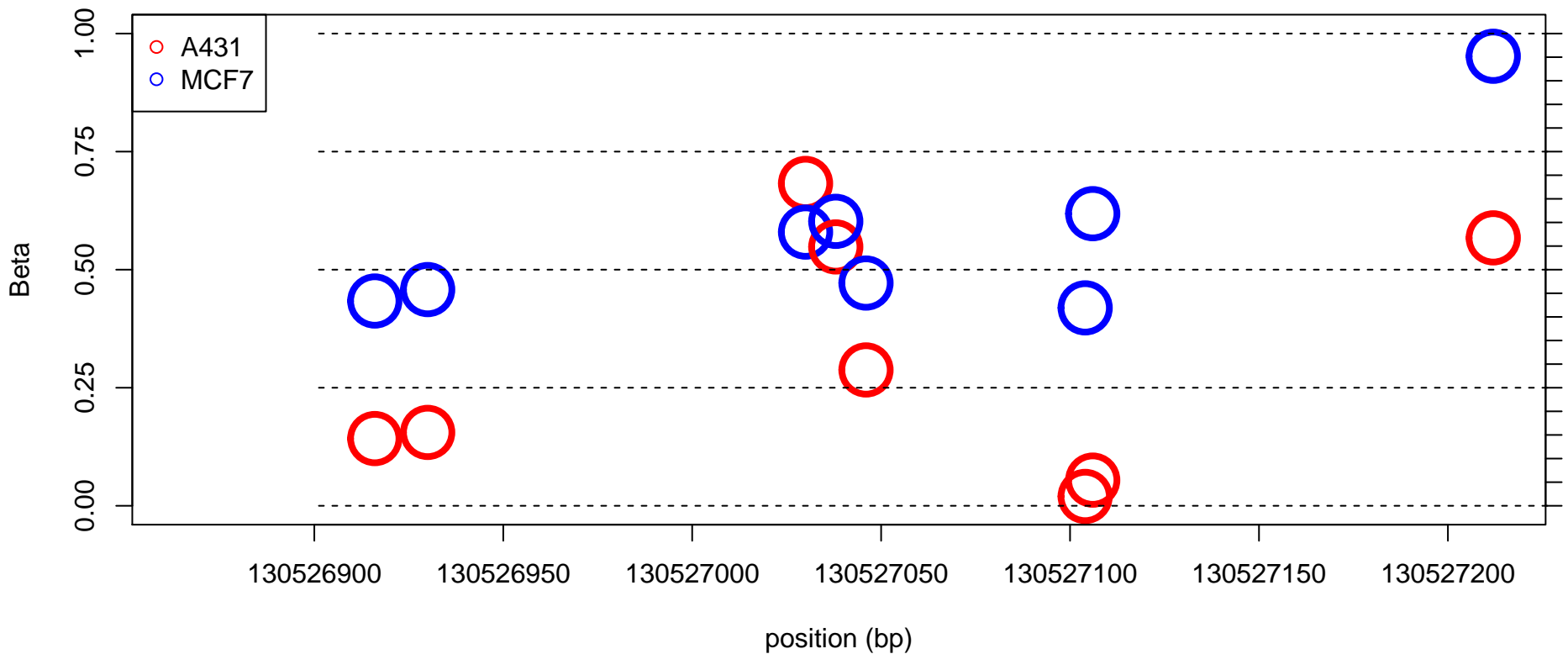

Supplement: Additional file 2 — DMRforPairs output for the comparison of A431-MCF7 and NA17018-NA17105. Please start from the HTML files in each folder. Available via the BMC Bioinformatics website. [file 1471-2105-15-141-S2.zip › 1394847754114233_MOESM2_ESM/A431_MCF7/figures/10117.pdf]

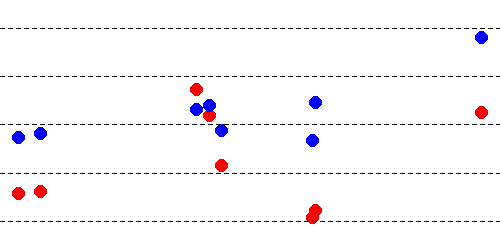

Supplement: Additional file 2 — DMRforPairs output for the comparison of A431-MCF7 and NA17018-NA17105. Please start from the HTML files in each folder. Available via the BMC Bioinformatics website. [file 1471-2105-15-141-S2.zip › 1394847754114233_MOESM2_ESM/A431_MCF7/figures/10117.png]

RegionID: 10118, chr12:130646022-130646497-M\_values

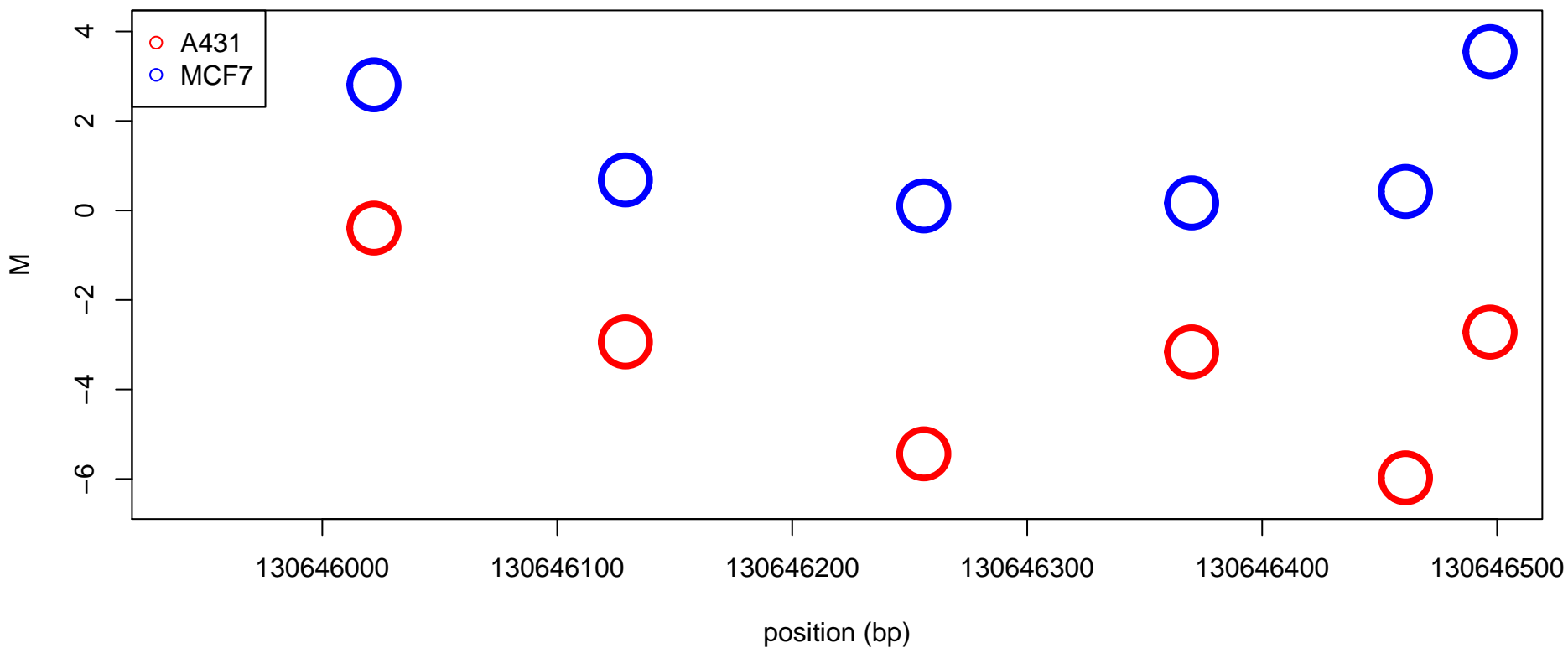

RegionID: 10118, chr12:130646022-130646497-Beta\_values

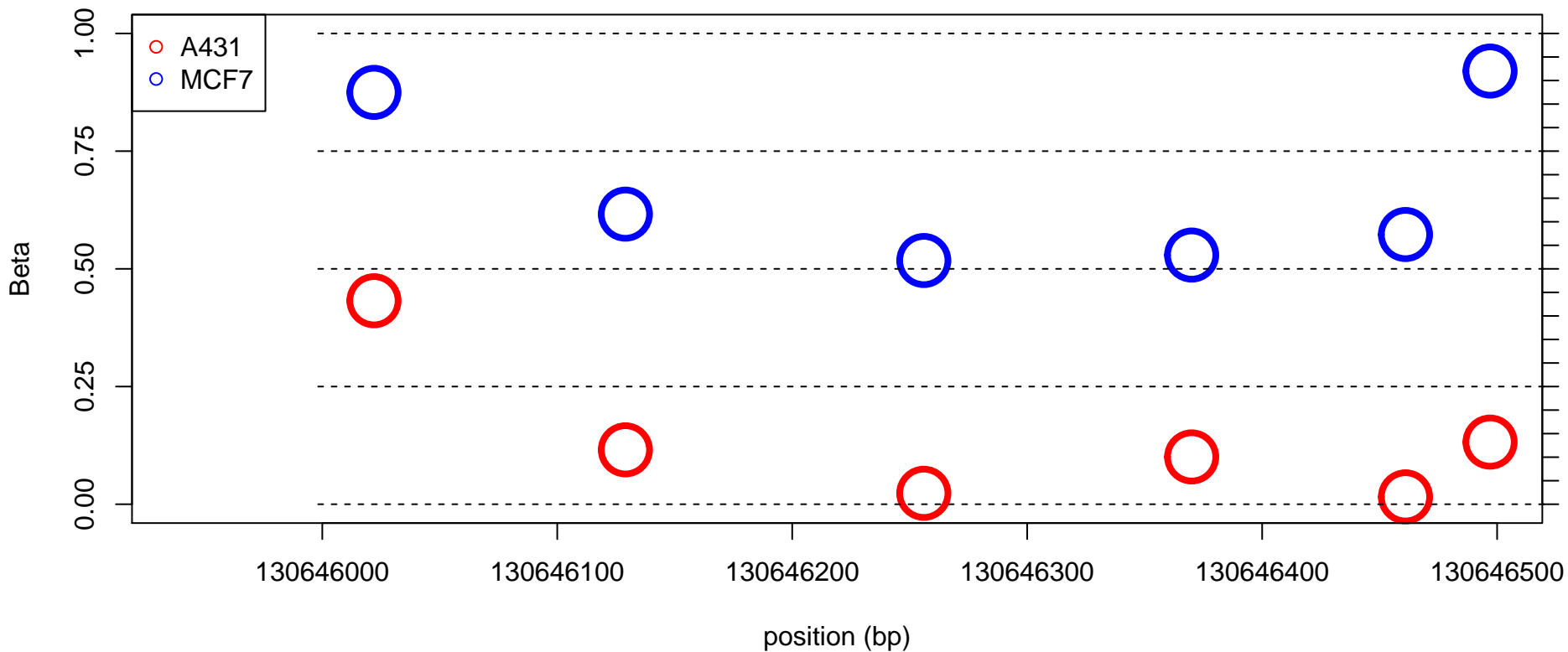

Chromosome 12

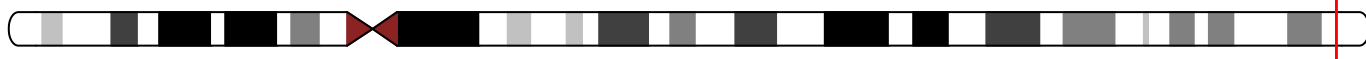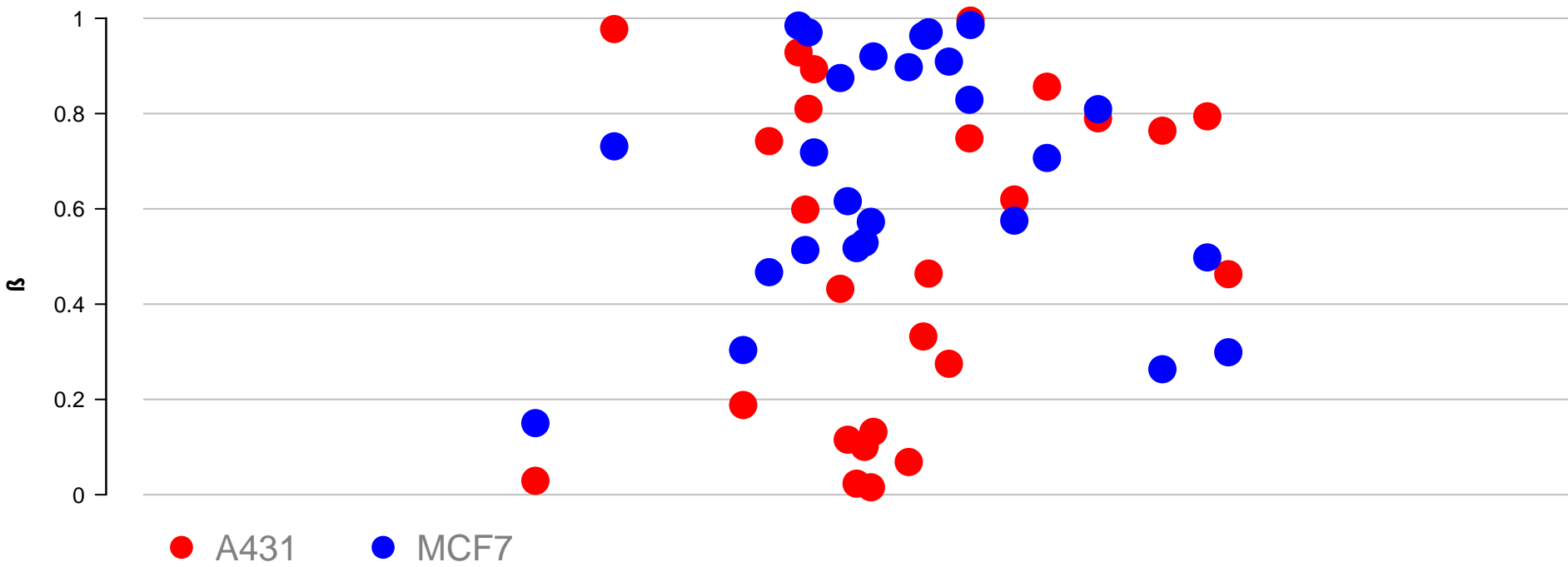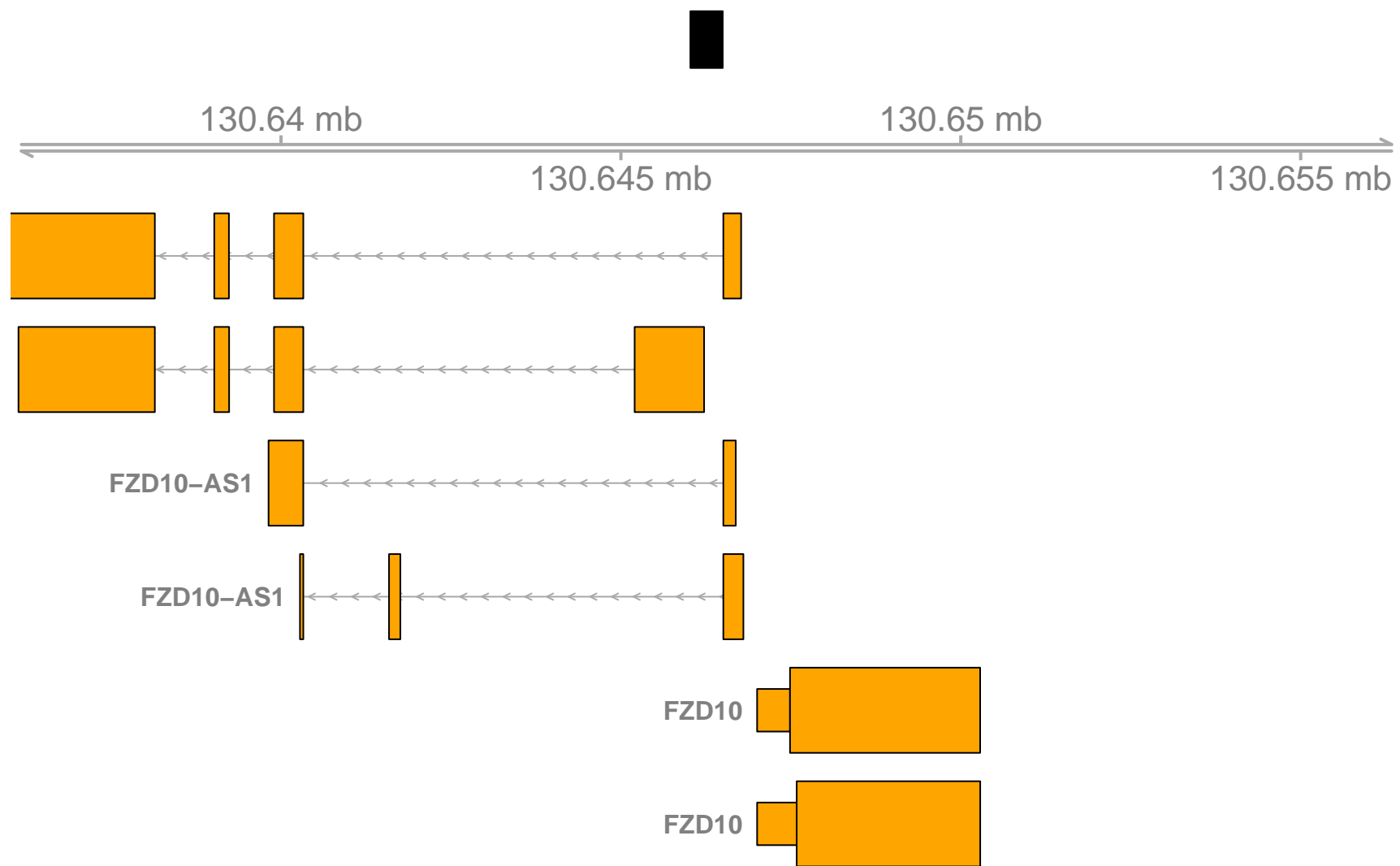

Supplement: Additional file 2 — DMRforPairs output for the comparison of A431-MCF7 and NA17018-NA17105. Please start from the HTML files in each folder. Available via the BMC Bioinformatics website. [file 1471-2105-15-141-S2.zip › 1394847754114233_MOESM2_ESM/A431_MCF7/figures/10118.pdf]

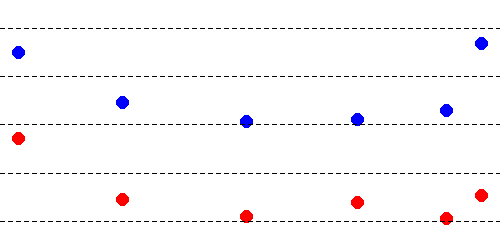

Supplement: Additional file 2 — DMRforPairs output for the comparison of A431-MCF7 and NA17018-NA17105. Please start from the HTML files in each folder. Available via the BMC Bioinformatics website. [file 1471-2105-15-141-S2.zip › 1394847754114233_MOESM2_ESM/A431_MCF7/figures/10118.png]

RegionID: 10119, chr12:130821453–130821607–M\_values

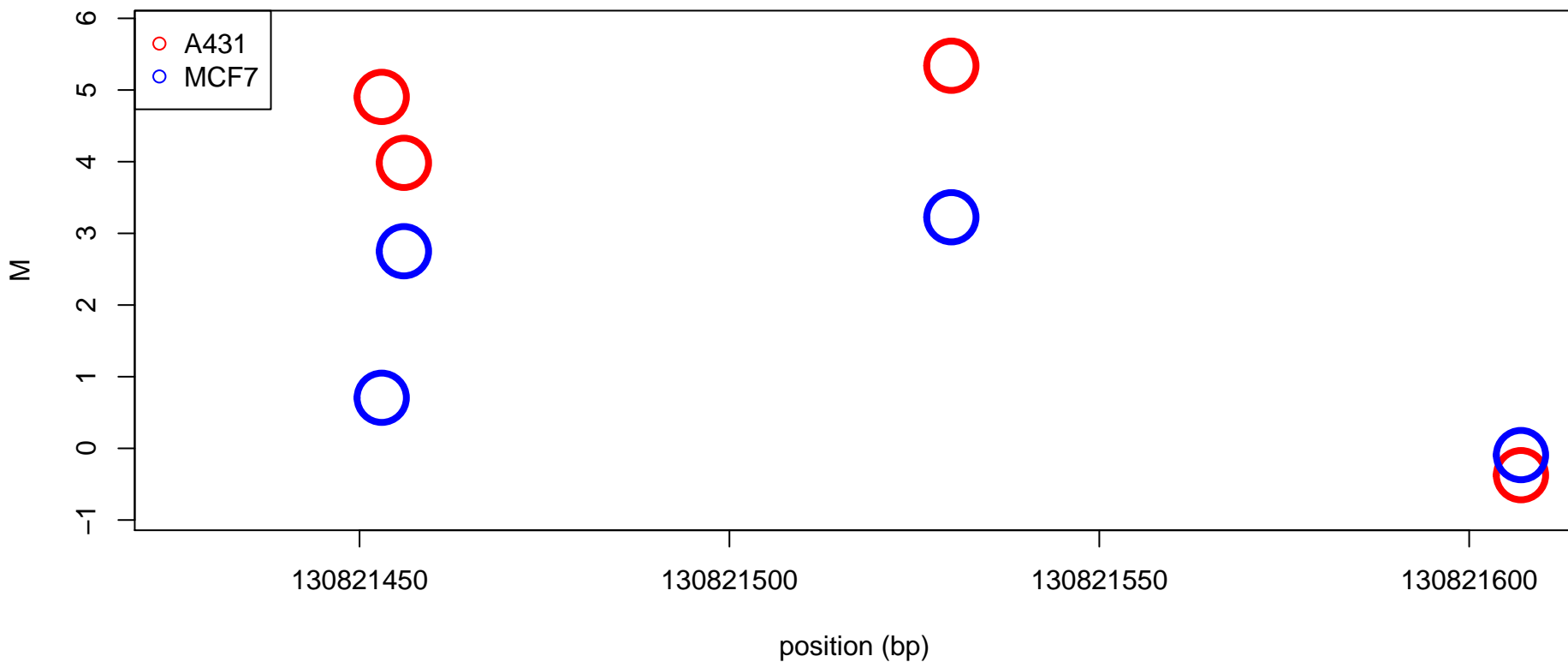

RegionID: 10119, chr12:130821453–130821607–Beta\_values

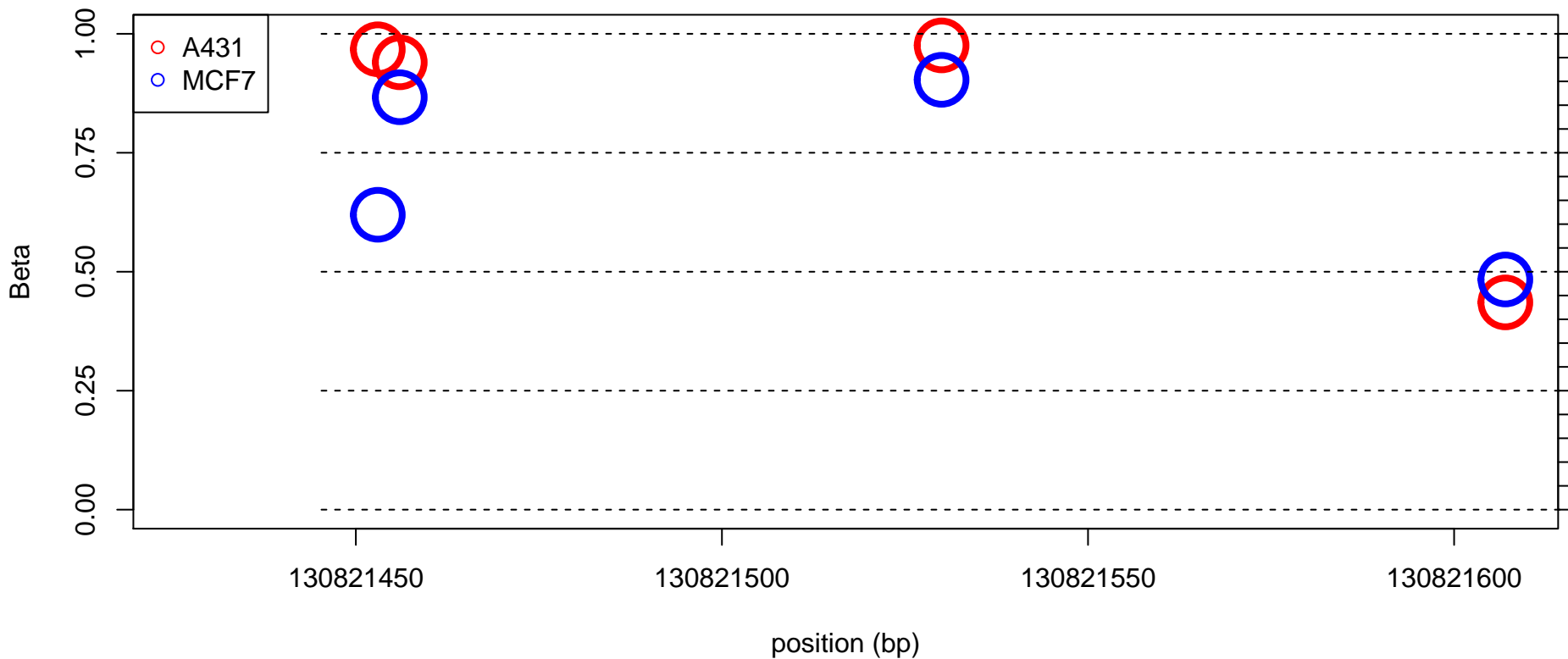

Supplement: Additional file 2 — DMRforPairs output for the comparison of A431-MCF7 and NA17018-NA17105. Please start from the HTML files in each folder. Available via the BMC Bioinformatics website. [file 1471-2105-15-141-S2.zip › 1394847754114233_MOESM2_ESM/A431_MCF7/figures/10119.pdf]

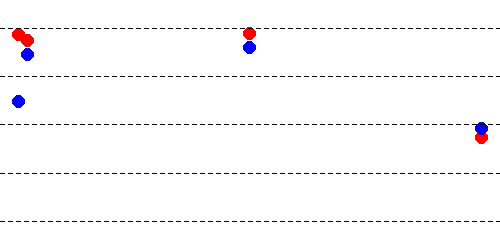

Supplement: Additional file 2 — DMRforPairs output for the comparison of A431-MCF7 and NA17018-NA17105. Please start from the HTML files in each folder. Available via the BMC Bioinformatics website. [file 1471-2105-15-141-S2.zip › 1394847754114233_MOESM2_ESM/A431_MCF7/figures/10119.png]

RegionID: 10120, chr12:131323926–131324319–M\_values

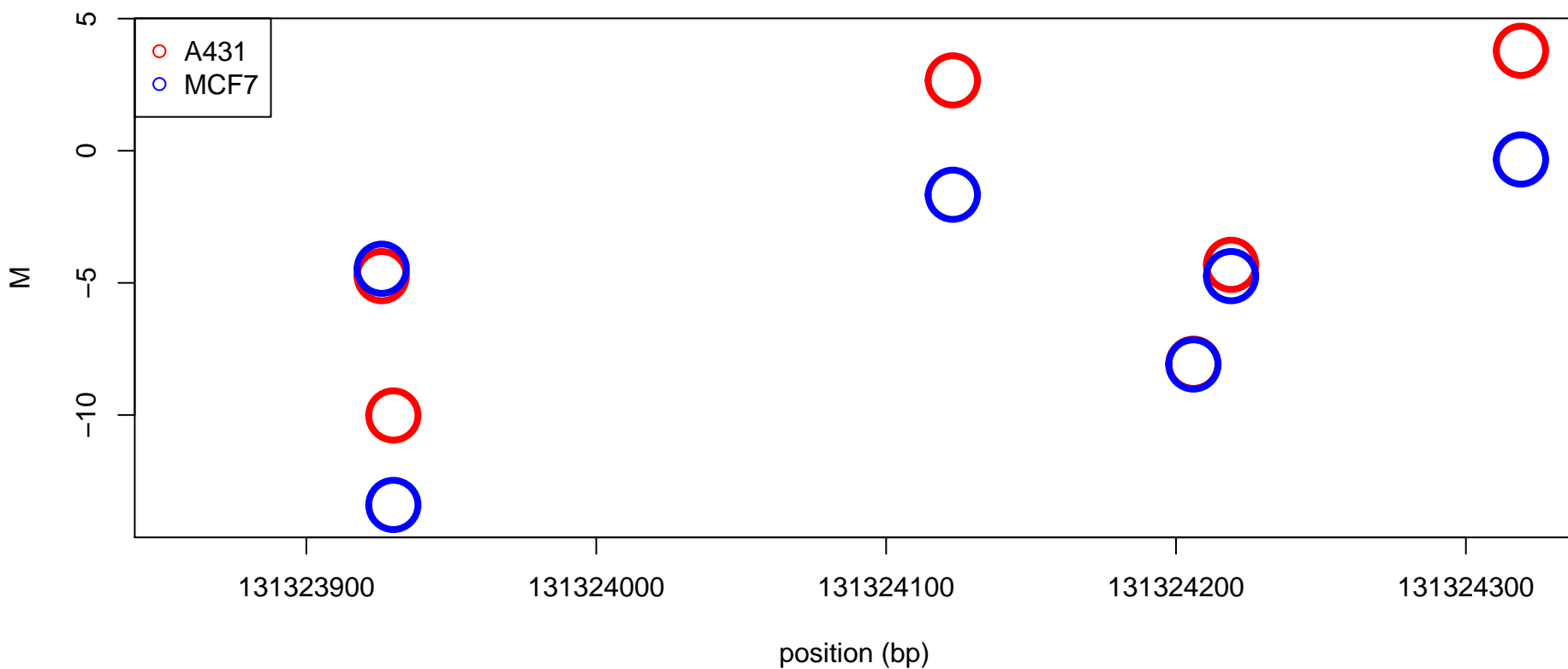

RegionID: 10120, chr12:131323926–131324319–Beta\_values

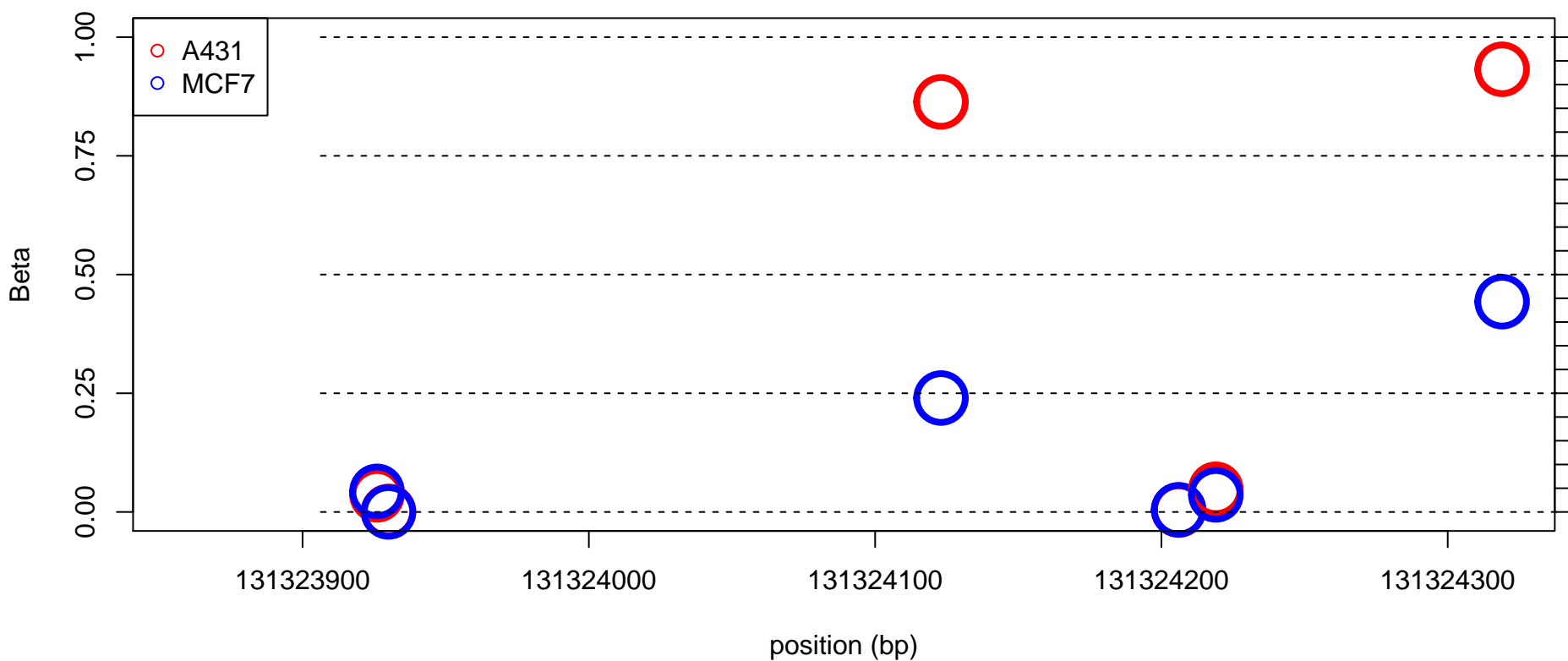

Supplement: Additional file 2 — DMRforPairs output for the comparison of A431-MCF7 and NA17018-NA17105. Please start from the HTML files in each folder. Available via the BMC Bioinformatics website. [file 1471-2105-15-141-S2.zip › 1394847754114233_MOESM2_ESM/A431_MCF7/figures/10120.pdf]

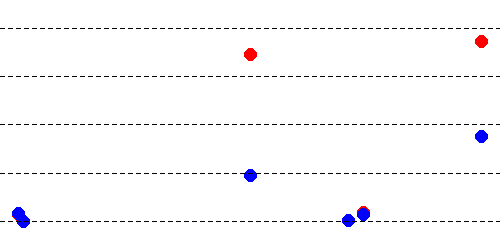

Supplement: Additional file 2 — DMRforPairs output for the comparison of A431-MCF7 and NA17018-NA17105. Please start from the HTML files in each folder. Available via the BMC Bioinformatics website. [file 1471-2105-15-141-S2.zip › 1394847754114233_MOESM2_ESM/A431_MCF7/figures/10120.png]

RegionID: 10123, chr12:132312702-132312875-M\_values

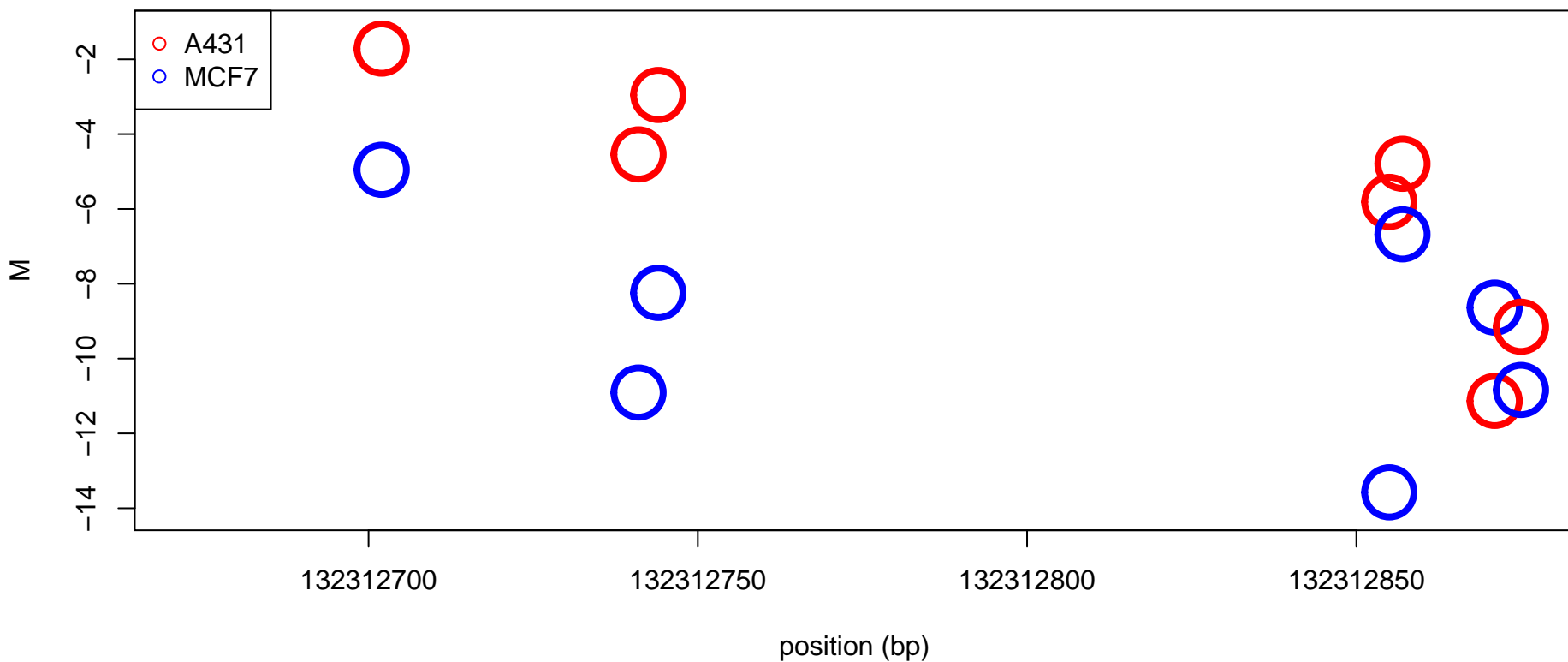

RegionID: 10123, chr12:132312702-132312875-Beta\_values

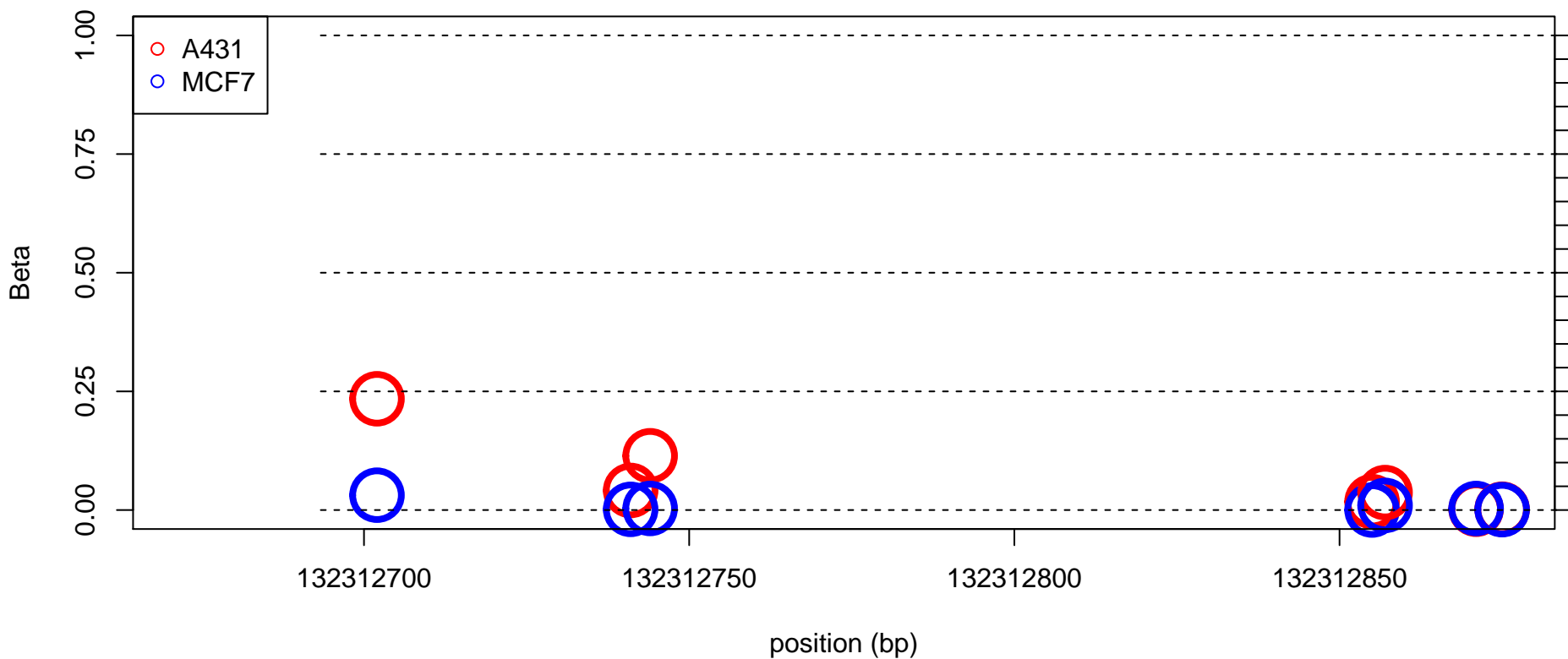

Supplement: Additional file 2 — DMRforPairs output for the comparison of A431-MCF7 and NA17018-NA17105. Please start from the HTML files in each folder. Available via the BMC Bioinformatics website. [file 1471-2105-15-141-S2.zip › 1394847754114233_MOESM2_ESM/A431_MCF7/figures/10123.pdf]

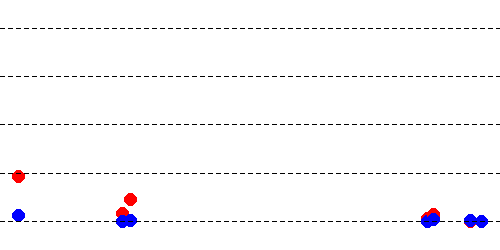

Supplement: Additional file 2 — DMRforPairs output for the comparison of A431-MCF7 and NA17018-NA17105. Please start from the HTML files in each folder. Available via the BMC Bioinformatics website. [file 1471-2105-15-141-S2.zip › 1394847754114233_MOESM2_ESM/A431_MCF7/figures/10123.png]

RegionID: 10129, chr12:132690643–132690782–M\_values

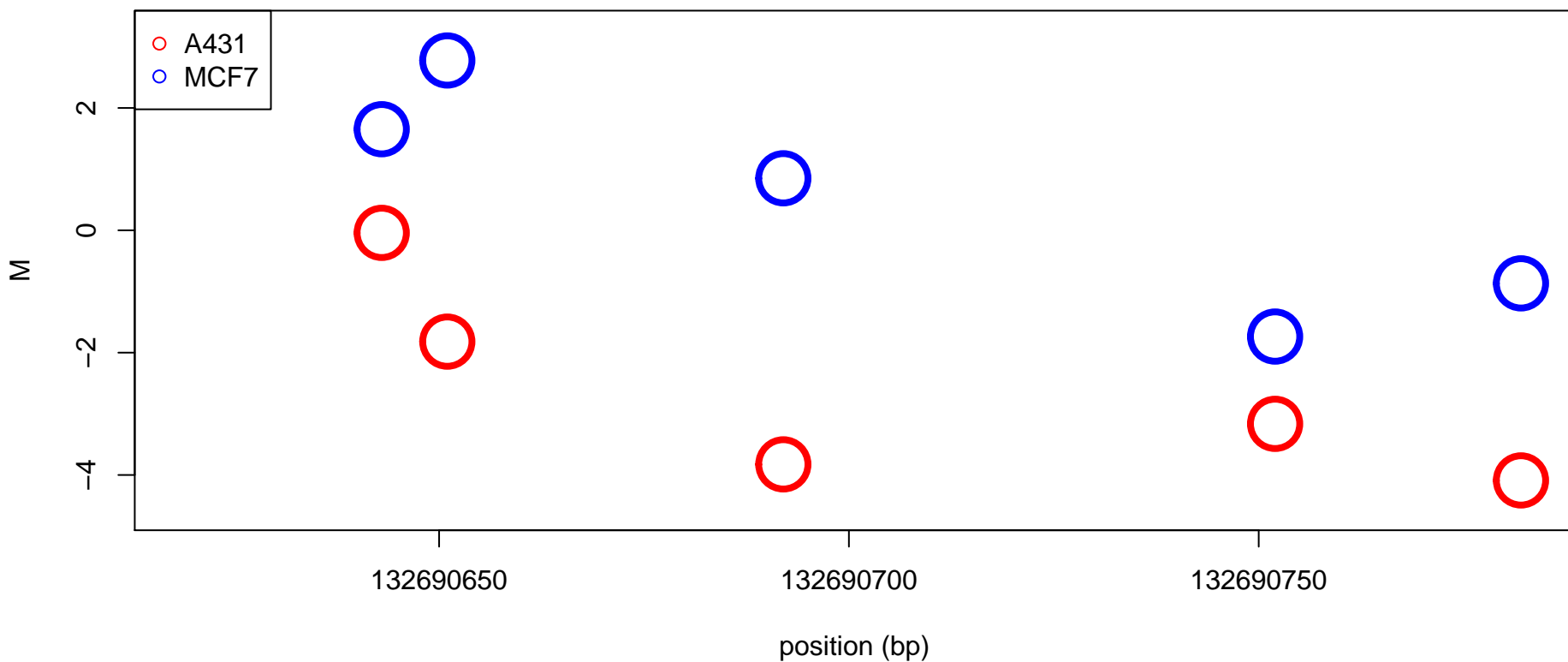

RegionID: 10129, chr12:132690643–132690782–Beta\_values

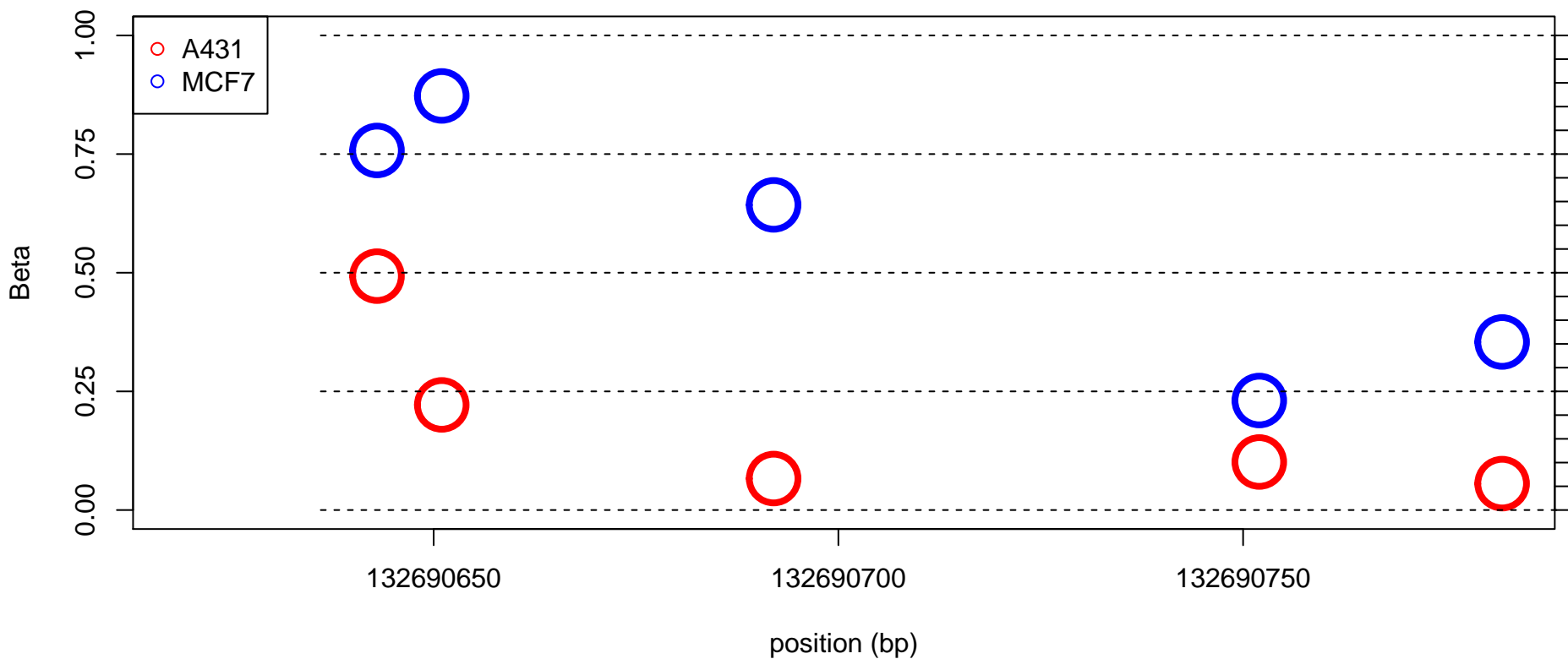

Supplement: Additional file 2 — DMRforPairs output for the comparison of A431-MCF7 and NA17018-NA17105. Please start from the HTML files in each folder. Available via the BMC Bioinformatics website. [file 1471-2105-15-141-S2.zip › 1394847754114233_MOESM2_ESM/A431_MCF7/figures/10129.pdf]

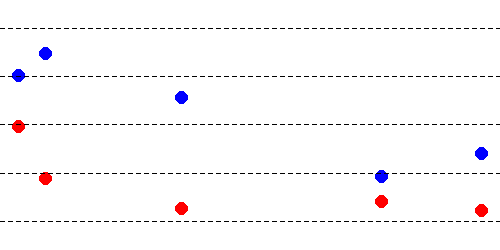

Supplement: Additional file 2 — DMRforPairs output for the comparison of A431-MCF7 and NA17018-NA17105. Please start from the HTML files in each folder. Available via the BMC Bioinformatics website. [file 1471-2105-15-141-S2.zip › 1394847754114233_MOESM2_ESM/A431_MCF7/figures/10129.png]

RegionID: 1013, chr1:219347252-219347458-M\_values

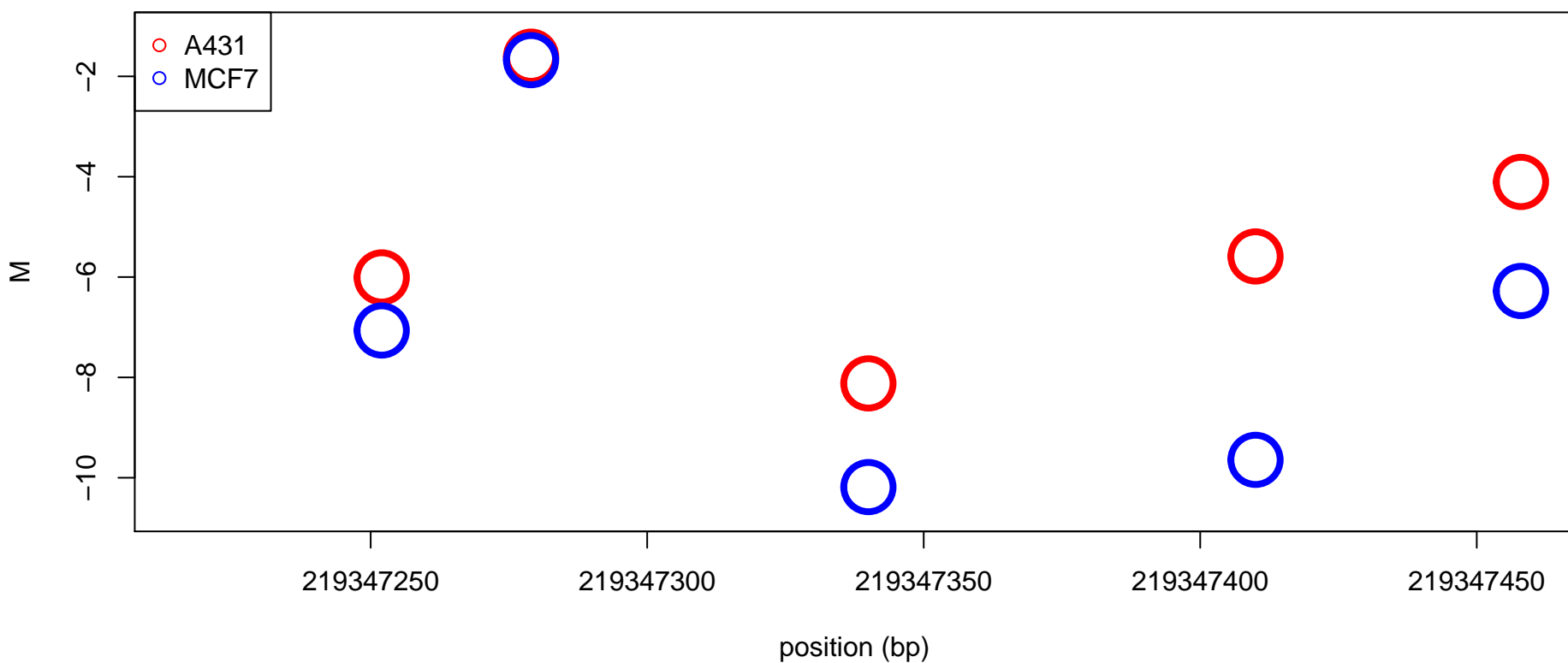

RegionID: 1013, chr1:219347252-219347458-Beta\_values

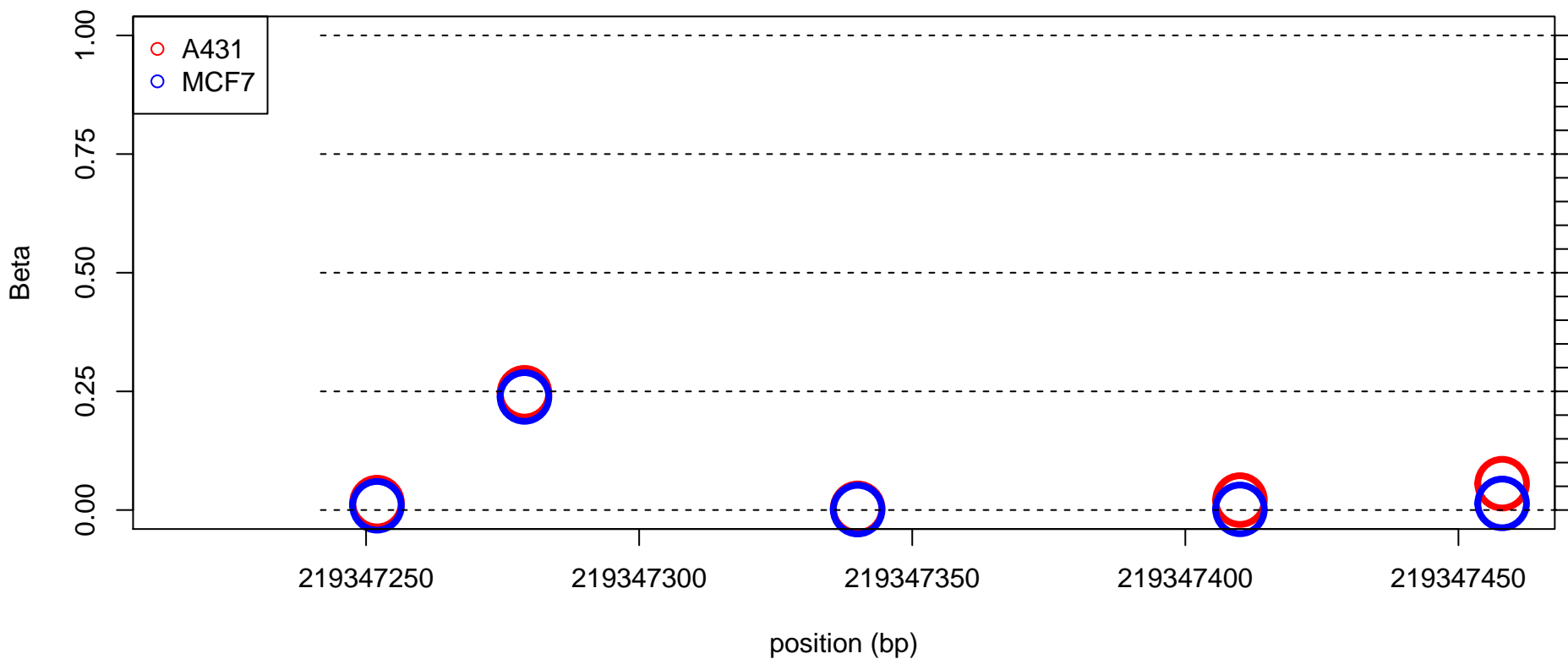

Supplement: Additional file 2 — DMRforPairs output for the comparison of A431-MCF7 and NA17018-NA17105. Please start from the HTML files in each folder. Available via the BMC Bioinformatics website. [file 1471-2105-15-141-S2.zip › 1394847754114233_MOESM2_ESM/A431_MCF7/figures/1013.pdf]

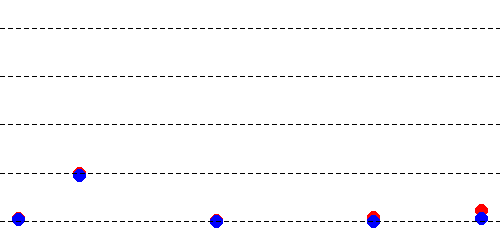

Supplement: Additional file 2 — DMRforPairs output for the comparison of A431-MCF7 and NA17018-NA17105. Please start from the HTML files in each folder. Available via the BMC Bioinformatics website. [file 1471-2105-15-141-S2.zip › 1394847754114233_MOESM2_ESM/A431_MCF7/figures/1013.png]

RegionID: 10130, chr12:133065724-133066069-M\_values

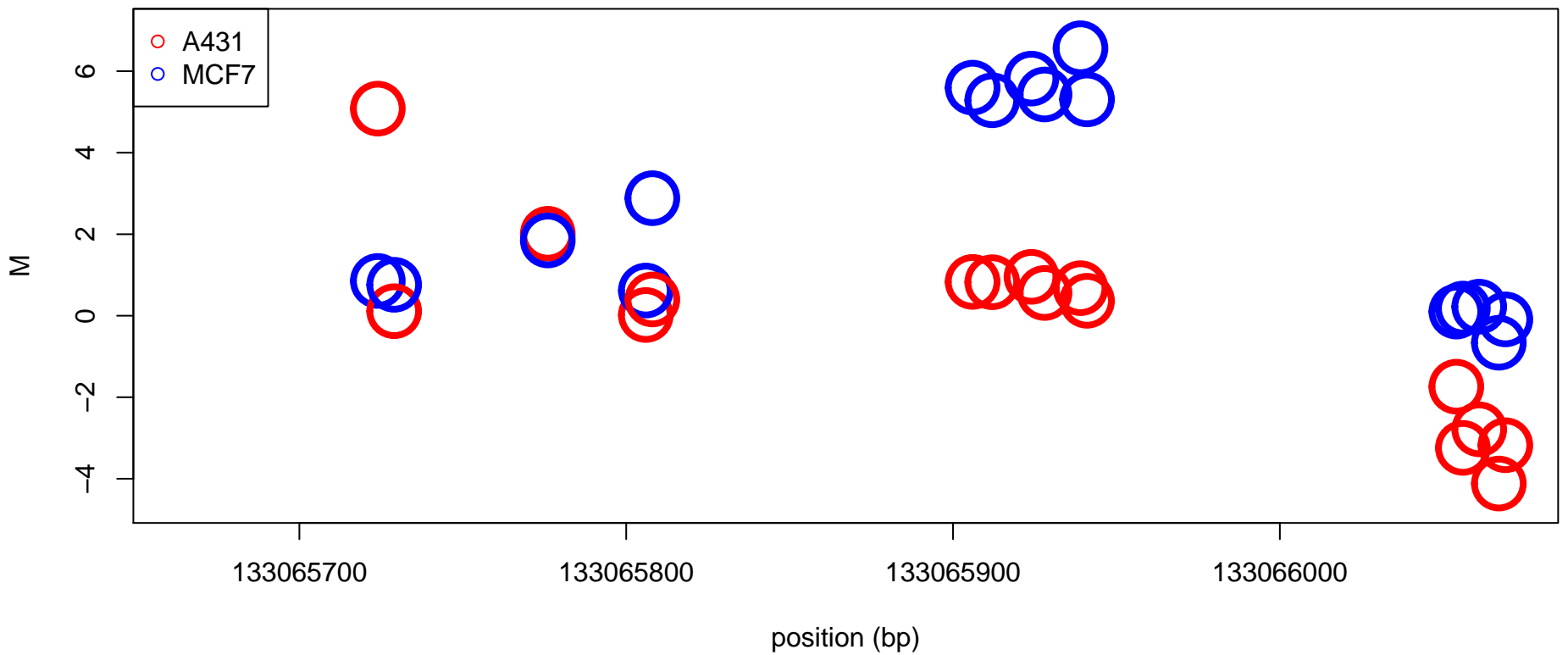

RegionID: 10130, chr12:133065724-133066069-Beta\_values

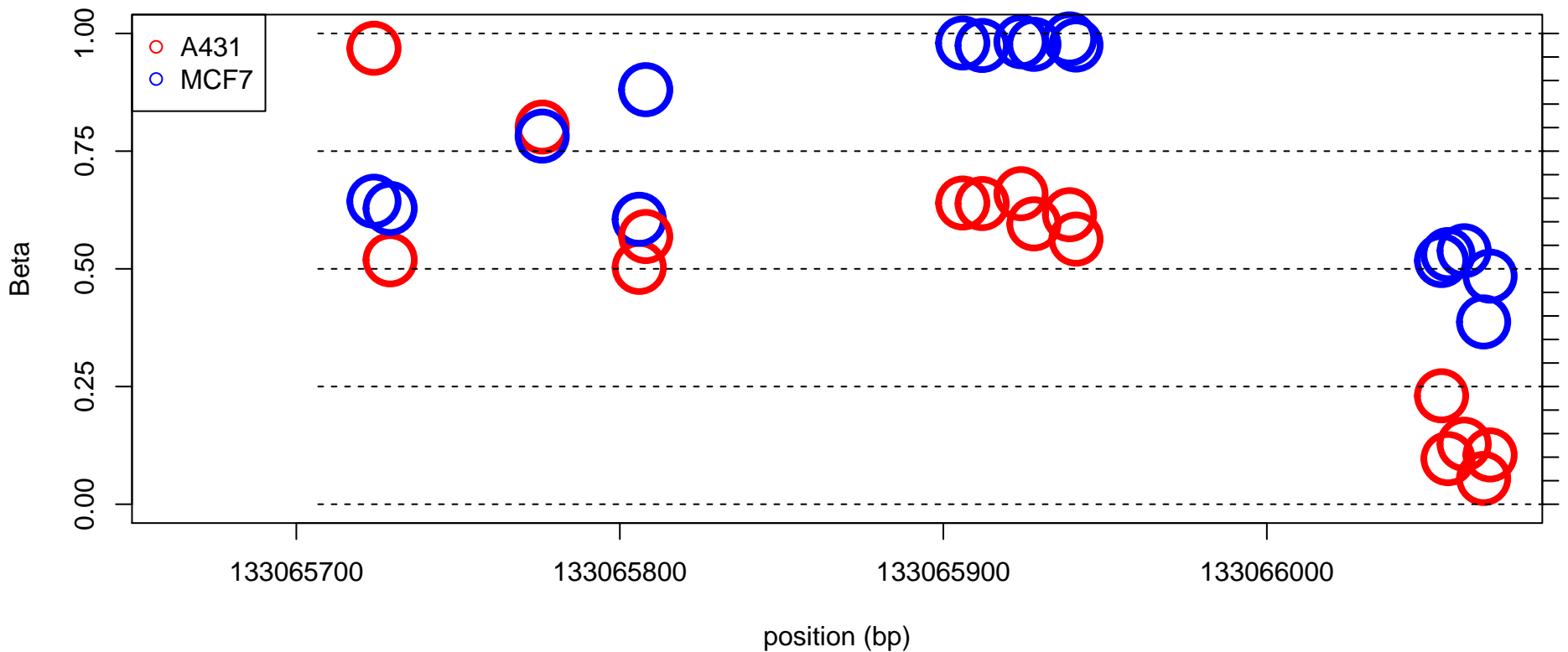

Supplement: Additional file 2 — DMRforPairs output for the comparison of A431-MCF7 and NA17018-NA17105. Please start from the HTML files in each folder. Available via the BMC Bioinformatics website. [file 1471-2105-15-141-S2.zip › 1394847754114233_MOESM2_ESM/A431_MCF7/figures/10130.pdf]

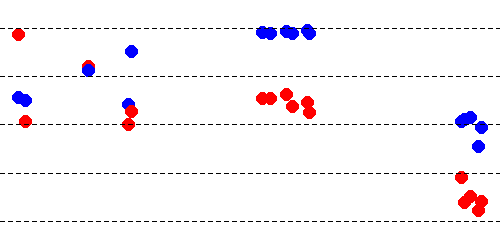

Supplement: Additional file 2 — DMRforPairs output for the comparison of A431-MCF7 and NA17018-NA17105. Please start from the HTML files in each folder. Available via the BMC Bioinformatics website. [file 1471-2105-15-141-S2.zip › 1394847754114233_MOESM2_ESM/A431_MCF7/figures/10130.png]

RegionID: 10132, chr12:133263907-133264322-M\_values

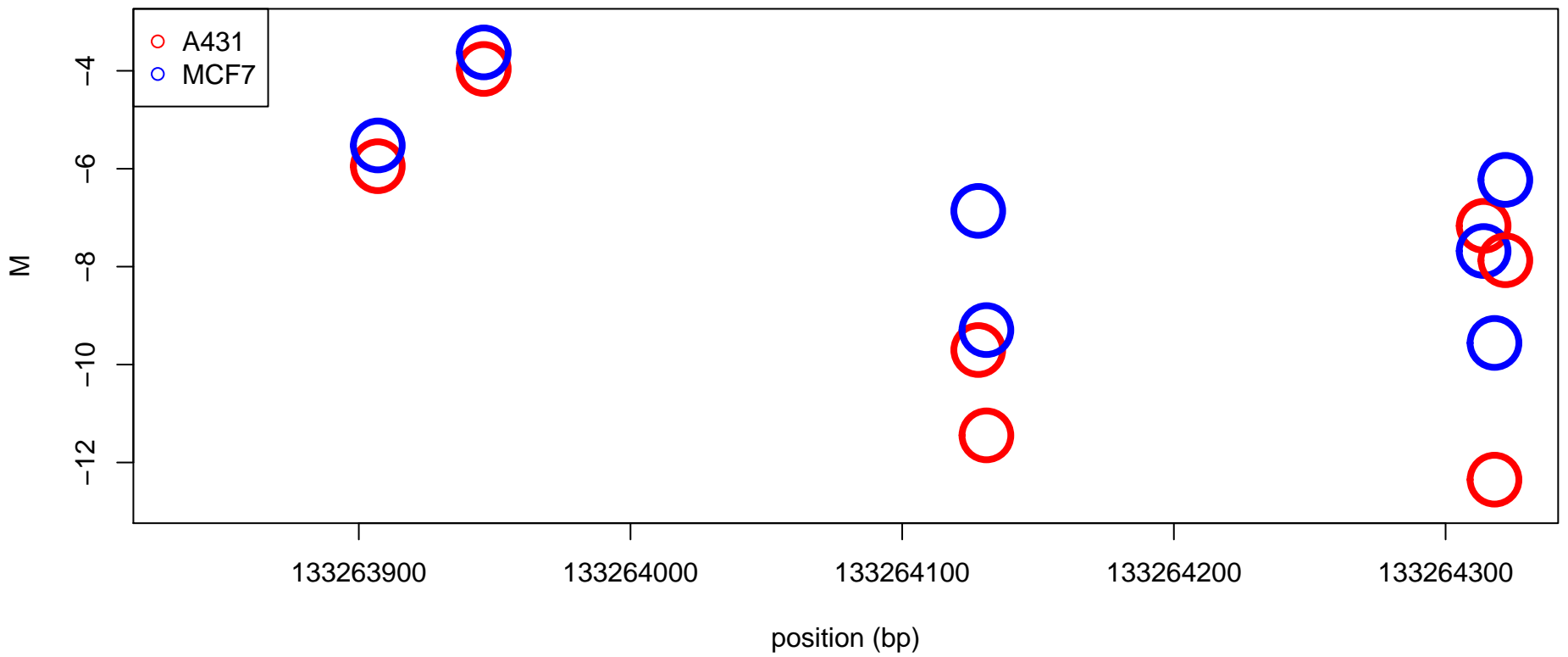

RegionID: 10132, chr12:133263907-133264322-Beta\_values

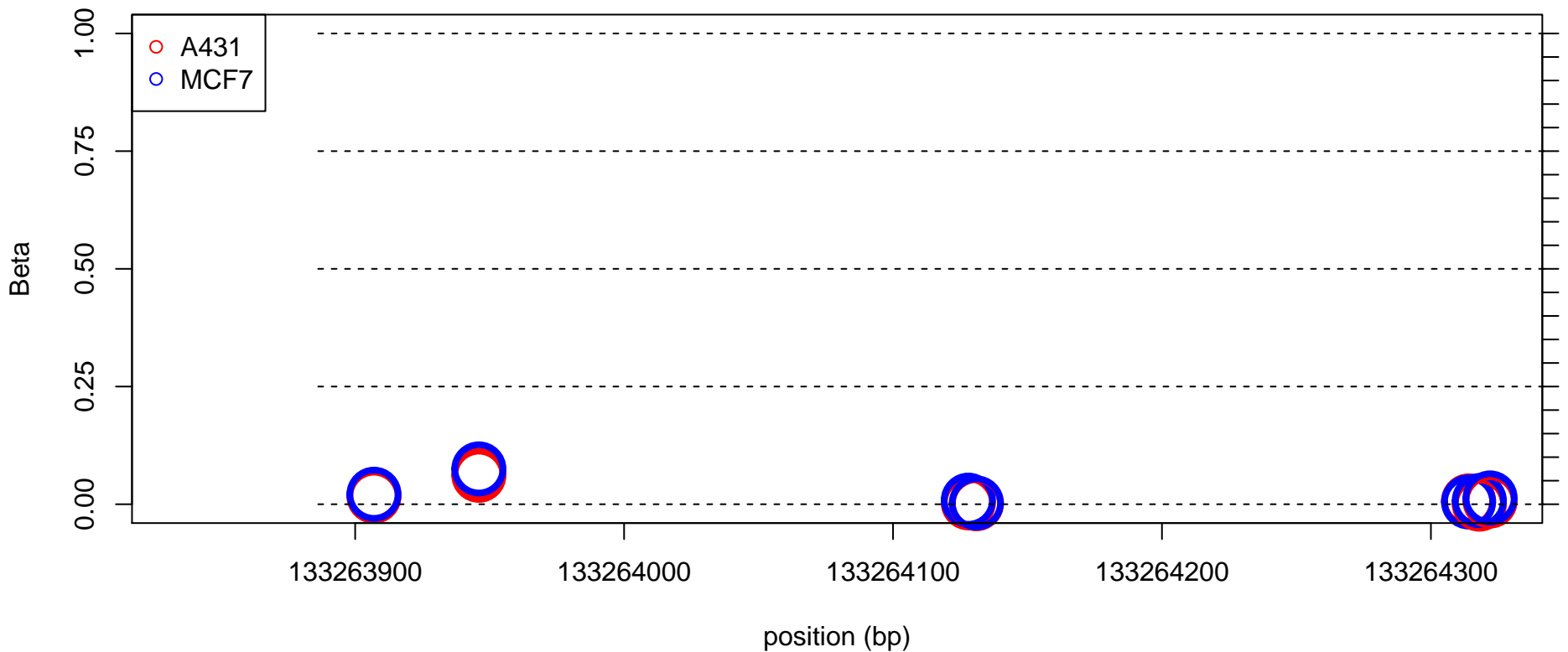

Supplement: Additional file 2 — DMRforPairs output for the comparison of A431-MCF7 and NA17018-NA17105. Please start from the HTML files in each folder. Available via the BMC Bioinformatics website. [file 1471-2105-15-141-S2.zip › 1394847754114233_MOESM2_ESM/A431_MCF7/figures/10132.pdf]

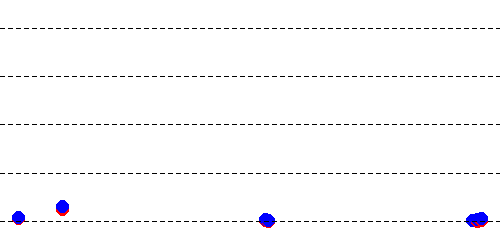

Supplement: Additional file 2 — DMRforPairs output for the comparison of A431-MCF7 and NA17018-NA17105. Please start from the HTML files in each folder. Available via the BMC Bioinformatics website. [file 1471-2105-15-141-S2.zip › 1394847754114233_MOESM2_ESM/A431_MCF7/figures/10132.png]

RegionID: 10133, chr12:133286734–133286970–M\_values

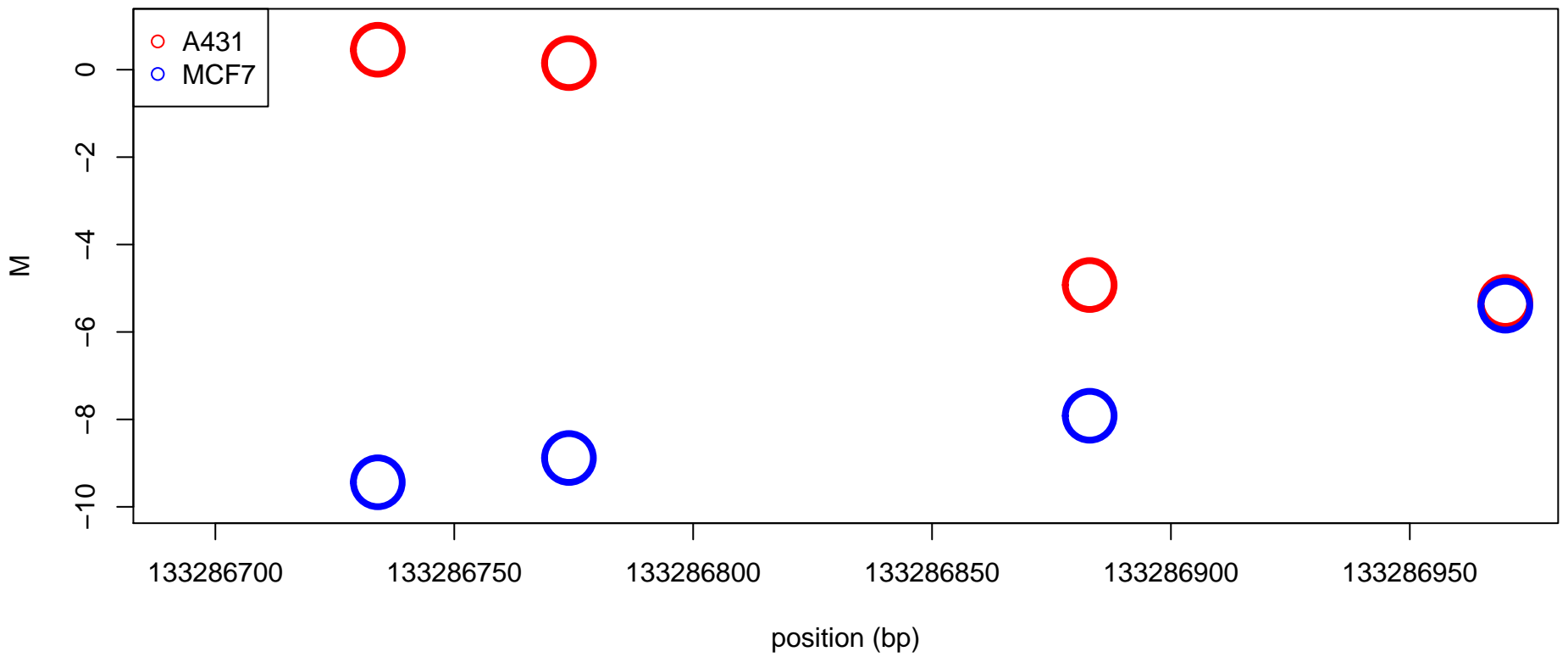

RegionID: 10133, chr12:133286734–133286970–Beta\_values

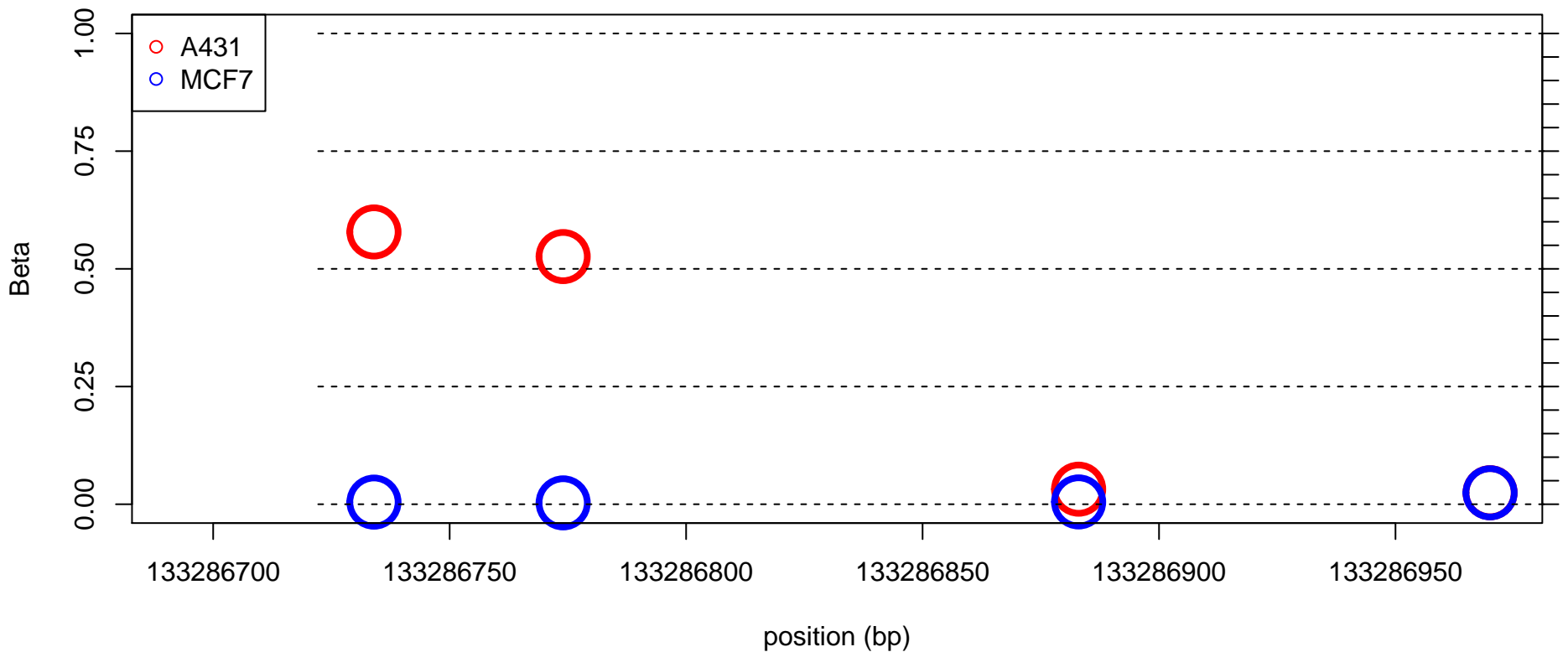

Supplement: Additional file 2 — DMRforPairs output for the comparison of A431-MCF7 and NA17018-NA17105. Please start from the HTML files in each folder. Available via the BMC Bioinformatics website. [file 1471-2105-15-141-S2.zip › 1394847754114233_MOESM2_ESM/A431_MCF7/figures/10133.pdf]

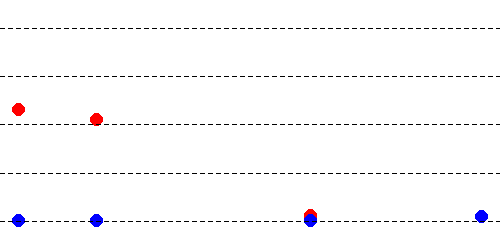

Supplement: Additional file 2 — DMRforPairs output for the comparison of A431-MCF7 and NA17018-NA17105. Please start from the HTML files in each folder. Available via the BMC Bioinformatics website. [file 1471-2105-15-141-S2.zip › 1394847754114233_MOESM2_ESM/A431_MCF7/figures/10133.png]

RegionID: 10135, chr12:133532874–133533354–M\_values

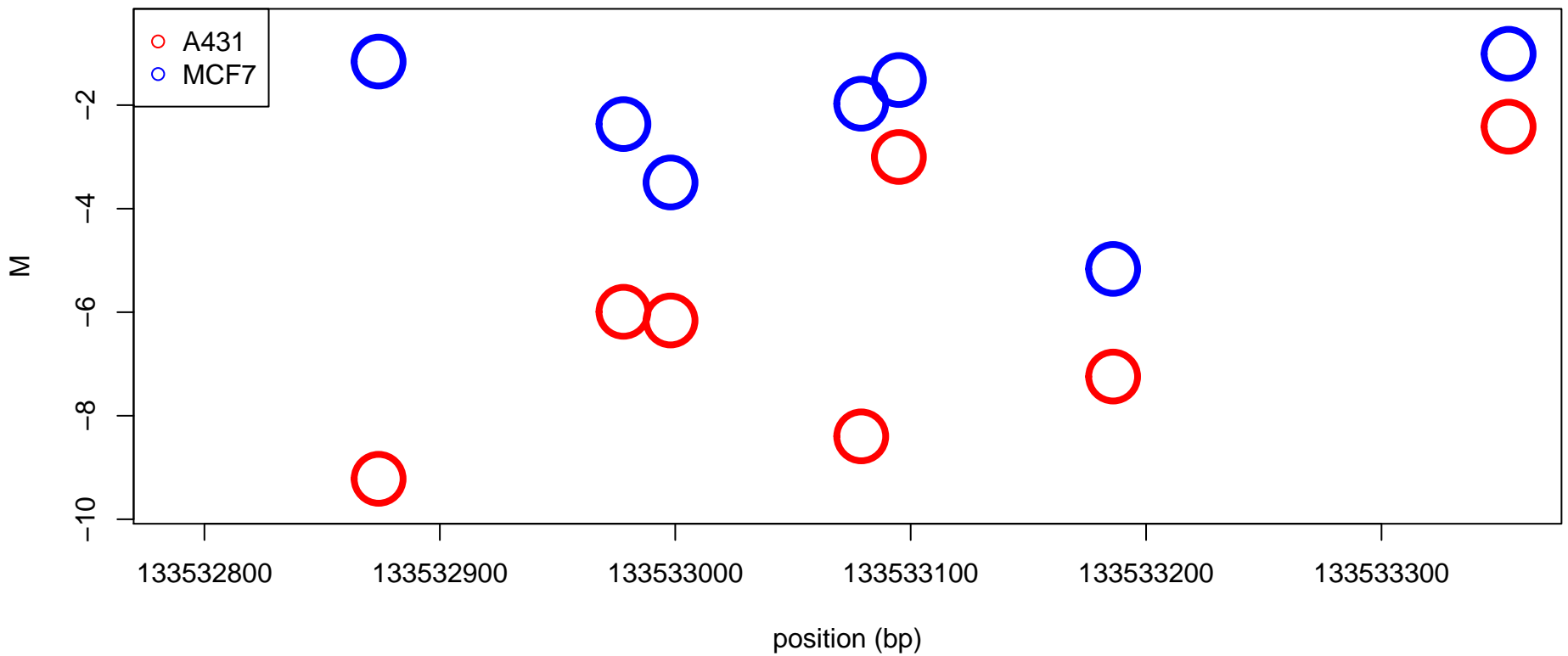

RegionID: 10135, chr12:133532874–133533354–Beta\_values

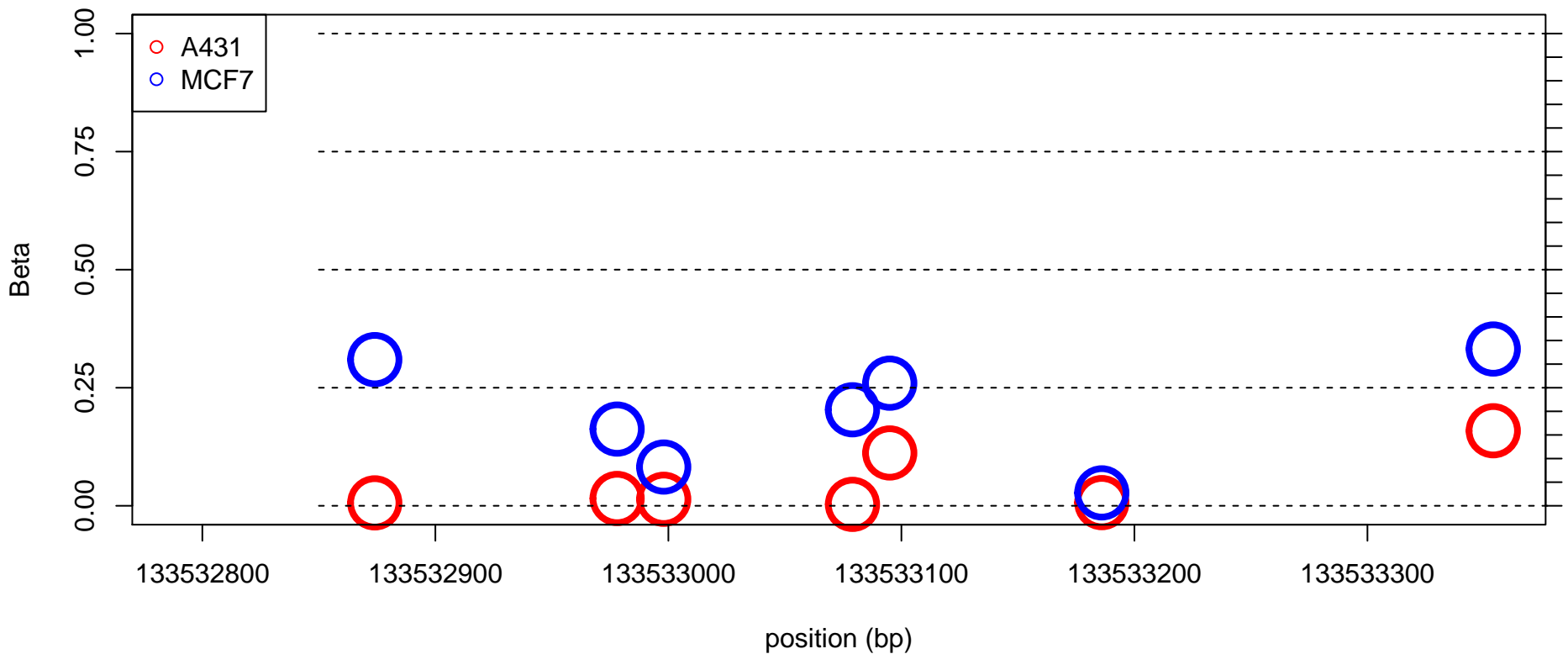

Chromosome 12

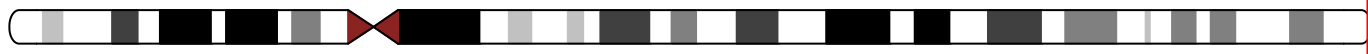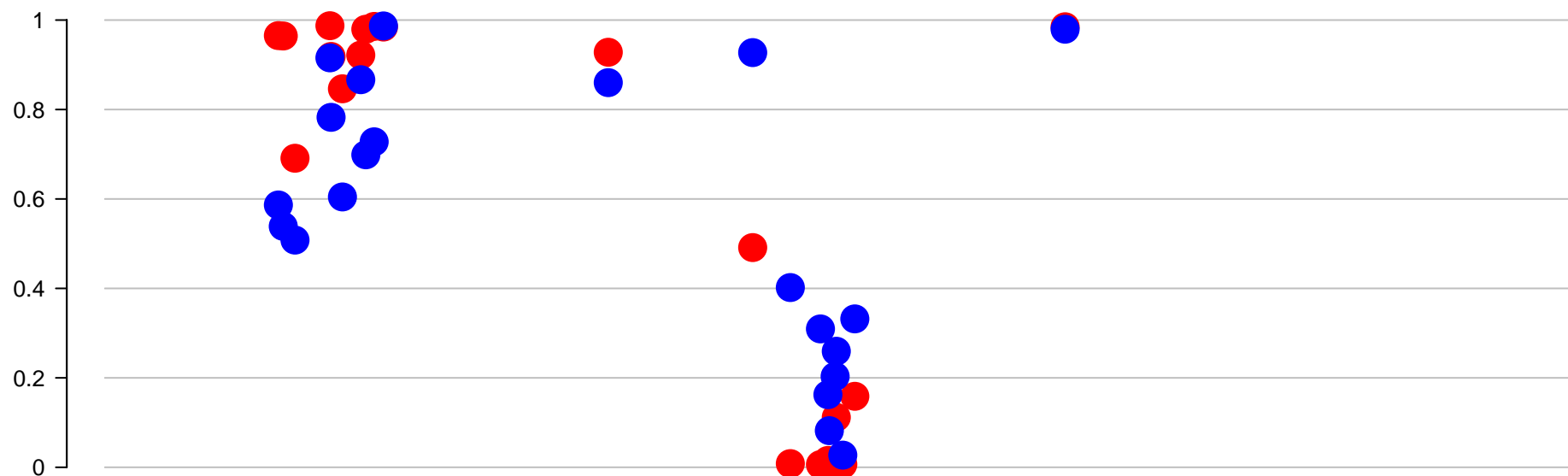

● A431 ● MCF7

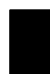

133.525 mb

133.535 mb

133.53 mb

133.54 mb

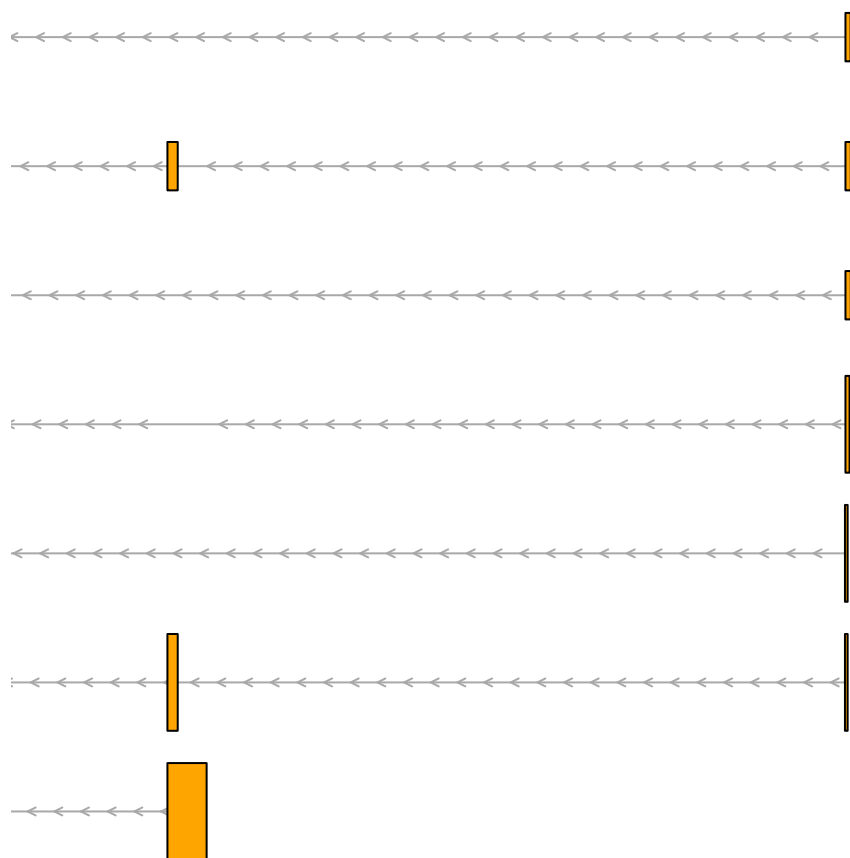

Supplement: Additional file 2 — DMRforPairs output for the comparison of A431-MCF7 and NA17018-NA17105. Please start from the HTML files in each folder. Available via the BMC Bioinformatics website. [file 1471-2105-15-141-S2.zip › 1394847754114233_MOESM2_ESM/A431_MCF7/figures/10135.pdf]

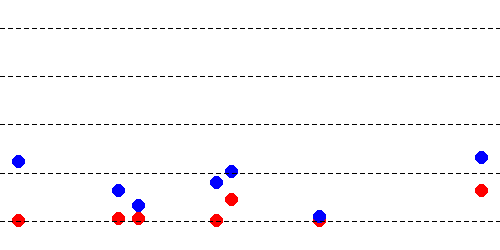

Supplement: Additional file 2 — DMRforPairs output for the comparison of A431-MCF7 and NA17018-NA17105. Please start from the HTML files in each folder. Available via the BMC Bioinformatics website. [file 1471-2105-15-141-S2.zip › 1394847754114233_MOESM2_ESM/A431_MCF7/figures/10135.png]

RegionID: 1014, chr1:220101698–220101962–M\_values

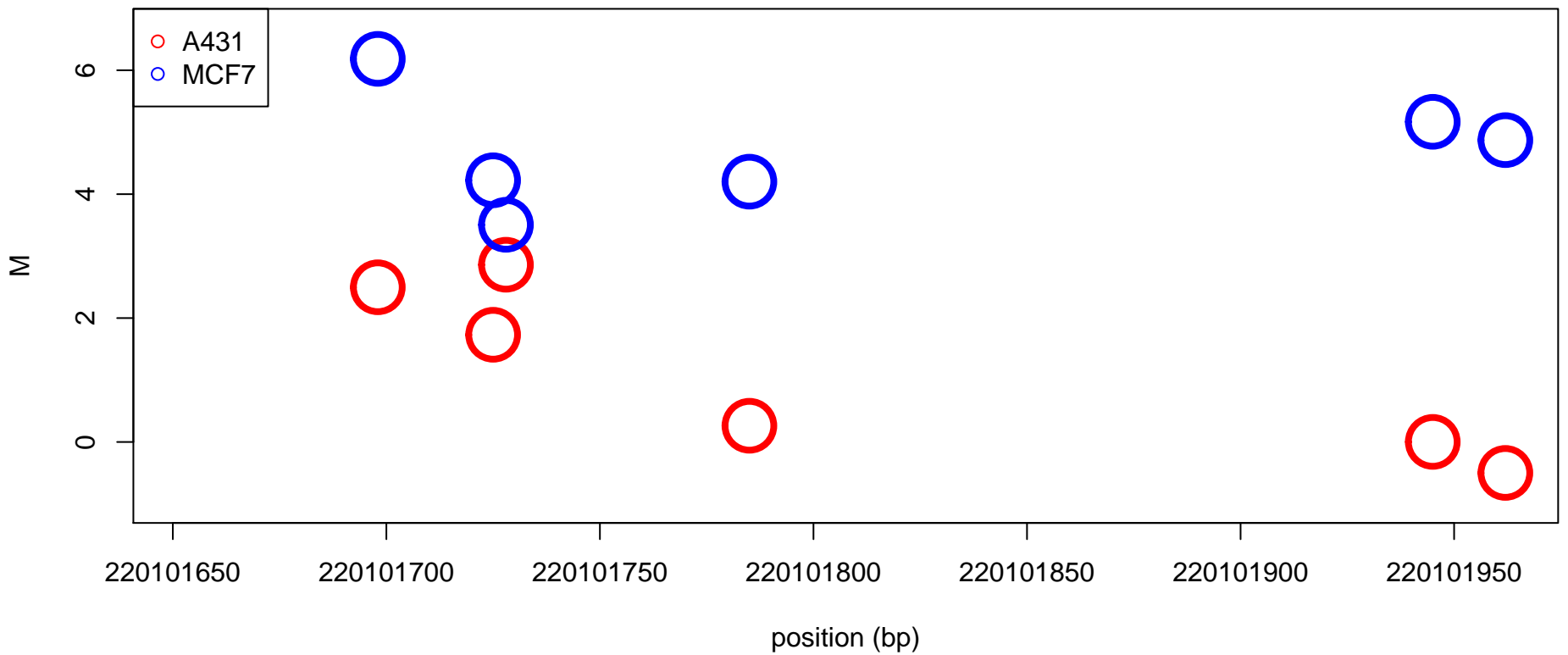

RegionID: 1014, chr1:220101698–220101962–Beta\_values

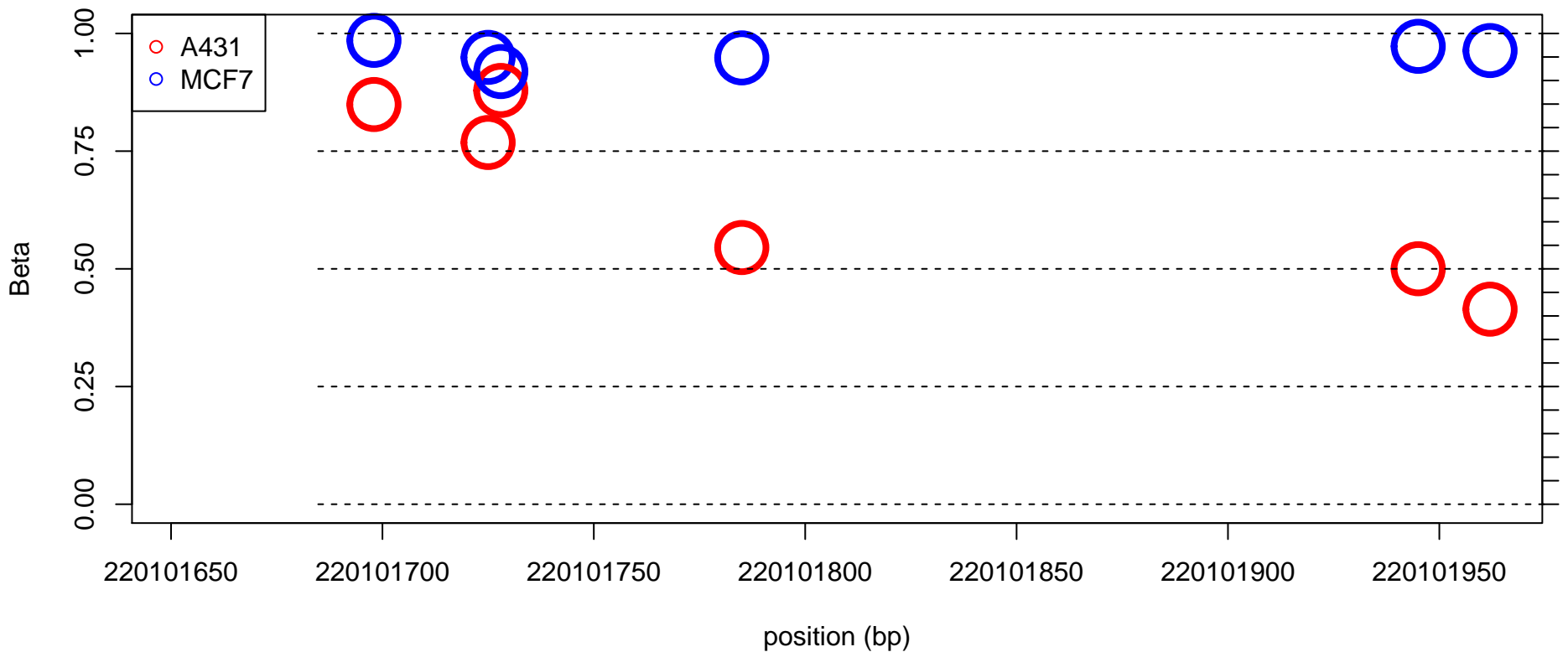

Chromosome 1

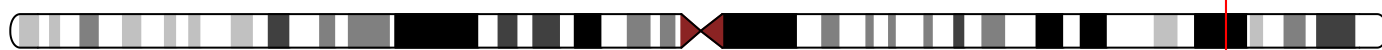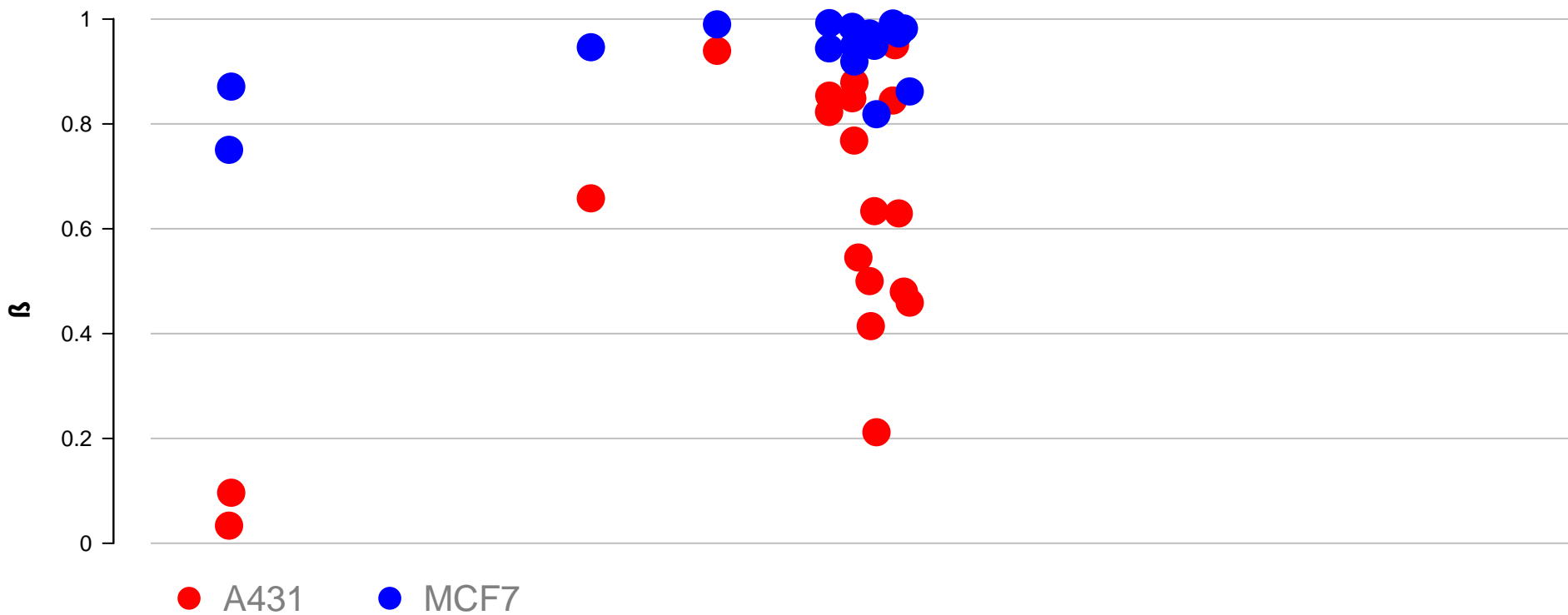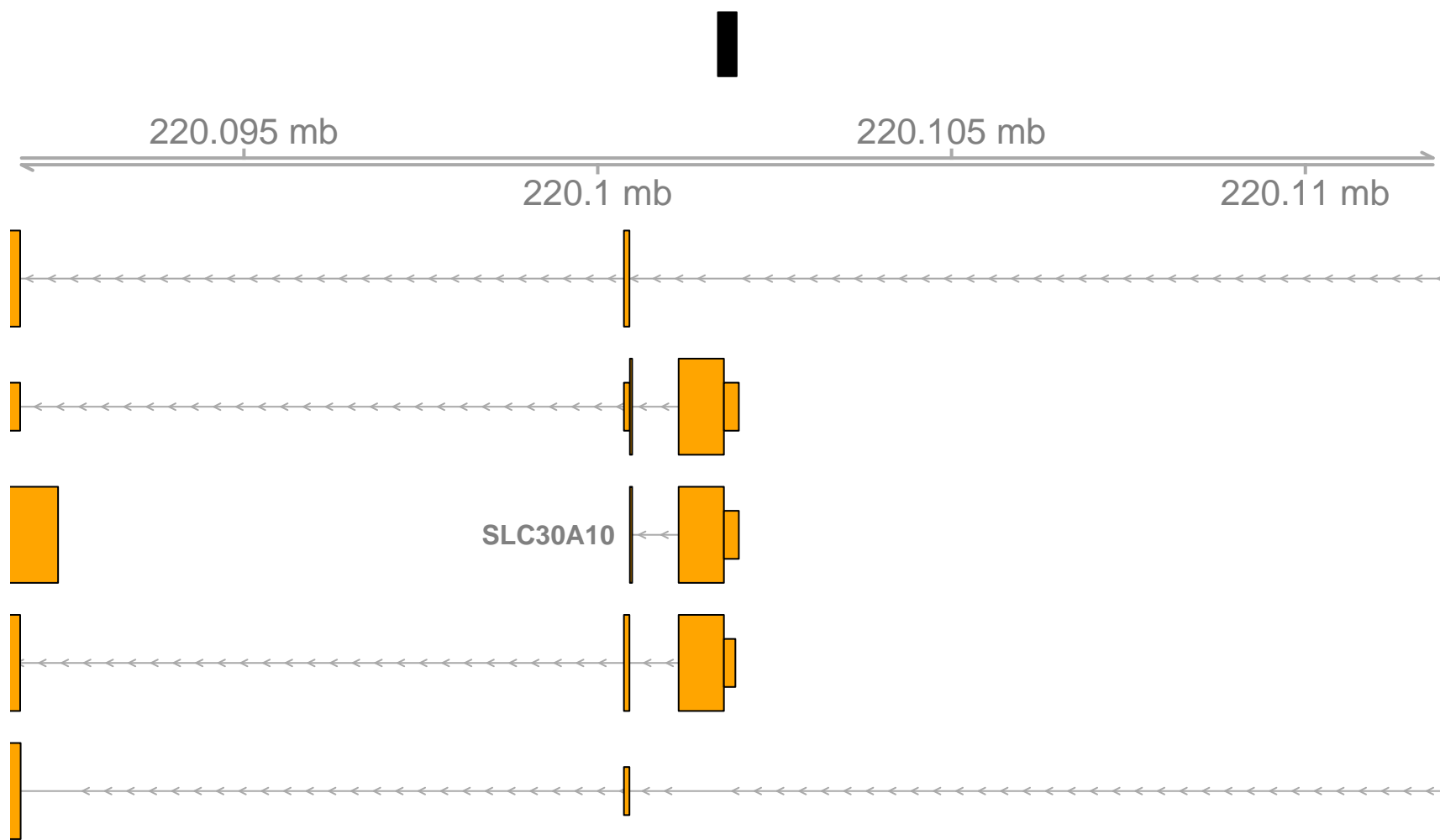

Supplement: Additional file 2 — DMRforPairs output for the comparison of A431-MCF7 and NA17018-NA17105. Please start from the HTML files in each folder. Available via the BMC Bioinformatics website. [file 1471-2105-15-141-S2.zip › 1394847754114233_MOESM2_ESM/A431_MCF7/figures/1014.pdf]

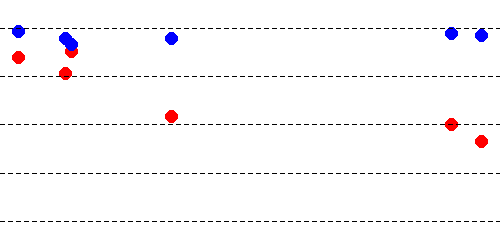

Supplement: Additional file 2 — DMRforPairs output for the comparison of A431-MCF7 and NA17018-NA17105. Please start from the HTML files in each folder. Available via the BMC Bioinformatics website. [file 1471-2105-15-141-S2.zip › 1394847754114233_MOESM2_ESM/A431_MCF7/figures/1014.png]
